# Genome-Wide Transcriptomic Analysis of the Effects of Infection with the Hemibiotrophic Fungus *Colletotrichum lindemuthianum* on Common Bean

**DOI:** 10.3390/plants11151995

**Published:** 2022-07-31

**Authors:** Juan C. Alvarez-Diaz, Richard Laugé, Etienne Delannoy, Stéphanie Huguet, Christine Paysant-Le Roux, Ariane Gratias, Valérie Geffroy

**Affiliations:** 1 Université Paris-Saclay, CNRS, INRAE, Université Evry, Institute of Plant Sciences Paris-Saclay (IPS2), 91190 Gif sur Yvette, France; juan-camilo.alvarez-diaz@universite-paris-saclay.fr (J.C.A.-D.); etienne.delannoy@inrae.fr (E.D.); pops.ips2@universite-paris-saclay.fr (S.H.); christine.paysant-le-roux@inrae.fr (C.P.-L.R.); ariane.gratias-weill@universite-paris-saclay.fr (A.G.); 2 Université Paris-Cité, CNRS, INRAE, Institute of Plant Sciences Paris-Saclay (IPS2), 91190 Gif sur Yvette, France; 3 Université Paris-Saclay, INRAE UR 1290 BIOGER, Av. Lucien Bretignières, BP 01, 78850 Thiverval Grignon, France; richard.lauge@inrae.fr

**Keywords:** *Phaseolus vulgaris*, *Colletotrichum lindemuthianum*, RNA-seq, transcriptome, *Co*-resistance genes, resistance response, PR10, Bet vI, NLR

## Abstract

Bean anthracnose caused by the hemibiotrophic fungus *Colletotrichum lindemuthianum* is one of the most important diseases of common bean (*Phaseolus vulgaris*) in the world. In the present study, the whole transcriptome of common bean infected with *C. lindemuthianum* during compatible and incompatible interactions was characterized at 48 and 72 hpi, corresponding to the biotrophy phase of the infection cycle. Our results highlight the prominent role of pathogenesis-related (PR) genes from the PR10/Bet vI family as well as a complex interplay of different plant hormone pathways including Ethylene, Salicylic acid (SA) and Jasmonic acid pathways. Gene Ontology enrichment analysis reveals that infected common bean seedlings responded by down-regulation of photosynthesis, ubiquitination-mediated proteolysis and cell wall modifications. In infected common bean, SA biosynthesis seems to be based on the PAL pathway instead of the ICS pathway, contrarily to what is described in *Arabidopsis*. Interestingly, ~30 NLR were up-regulated in both contexts. Overall, our results suggest that the difference between the compatible and incompatible reaction is more a question of timing and strength, than a massive difference in differentially expressed genes between these two contexts. Finally, we used RT-qPCR to validate the expression patterns of several genes, and the results showed an excellent agreement with deep sequencing.

## 1. Introduction

Common bean (*Phaseolus vulgaris*) is the most important grain legume for human consumption worldwide and constitutes a major source of protein for populations in Africa and Latin America. This is a diploid species (2n = 22) with a relatively small genome estimated to be ~600 Mb [1,2]. The Andean common bean landrace G19833 (Chaucha Chuga) was used to obtain the first reference genome of *P. vulgaris* [3]. The resulting annotation includes ~27,000 protein-coding loci, including ~400 Nucleotide-binding leucine-rich repeat (NLR) genes [4], the major class of disease resistance genes in plants [5]. Surprisingly, 50% of common bean NLR are methylated in the three sequence contexts (CHH, CHG, CG), like transposable elements, suggesting the existence of a transcriptional gene silencing mechanism in the absence of pathogen [6].

Anthracnose, caused by the fungus *Colletotrichum lindemuthianum*, is the most important disease of common bean throughout the world, especially in tropical areas of Latin America and Eastern Africa where common bean is one of the major staple crops [7]. It causes important yield losses and significantly reduces the quality of bean seeds and pods, especially when susceptible cultivars are planted under cool and humid environmental conditions that favor anthracnose development [7]. Fungi are characterized by a wide variety of infection strategies, from strict biotrophy to necrotrophy. *C. lindemuthianum* is a hemibiotrophic fungus characterized by two successive phases, biotrophy followed by necrotrophy. During their infection cycle, genuine hemibiotrophic fungi such as *C. lindemuthianum* have first a biotrophic phase where they develop a succession of specialized infection structures, i.e., an appressorium, to penetrate the host plant epidermis, then biotrophic hyphae to feed on living host cells, followed by a second necrotrophic phase where necrotrophic hyphae spread for host tissue colonization [8]. Histological studies of infection on common bean have shown that the transition switch between biotrophy and necrotrophy occurs between 72h and 96h after infection [9,10].

Because chemical control is not only expensive but also harmful to human health and the environment, genetic resistance represents the most reliable and cost-effective control strategy [7,11]. The genetics of anthracnose resistance in common bean has been studied for a long time, since this host–pathogen interaction was the first report of race-cultivar specificity [12]. A large diversity of virulence have been reported for *C. lindemuthianum* strains [13,14,15]. Likewise, many dominant resistance (*R*) genes conditioning resistance against different strains of the fungus have been described [16], strongly suggesting a gene-for-gene-type resistance [17] for this interaction. As expected since NLR is the prevalent class of R gene, many anthracnose *R* genes are co-located with NLR clusters [16]. For example, the *Co-9 R* gene from the genotype BAT93, conferring resistance against strain C531, is co-located with a NLR gene cluster at one end of chromosome 4 [13,18]. However, a different situation appears for the *Co-x*/*Co-1* allelic series, with *Co-x* encoding a CRINCKLY4 kinase [14,19,20].

Even if many anthracnose *R* genes have been identified, the molecular pathways underlying the common bean/*C. lindemuthianum* interaction are not fully characterized. The rapid development of high-throughput sequencing technology has helped to bridge the gap between model species and crops. In particular, transcriptome sequencing (RNA sequencing, RNA-seq) has contributed to important discoveries in various fields such as host–pathogen interactions. RNA-seq has been applied to common bean in interaction with bacteria [21], BCMV [22] and the fungus *Fusarium oxysporum* [23] but also to common bean (carrying *Co-1* gene)/*C. lindemuthianum* interaction [24]. In order to identify host plant response upon *C. lindemuthianum* infection, we characterized the transcriptome of common bean BAT93 (*Co-9*) after infection with two strains of *C. lindemuthianum* leading to compatible or incompatible interactions. The results described here improve our fundamental knowledge of molecular responses to the common bean/*C. lindemuthianum* interaction.

## 2. Results

### 2.1. Disease Development

**Figure 1:**
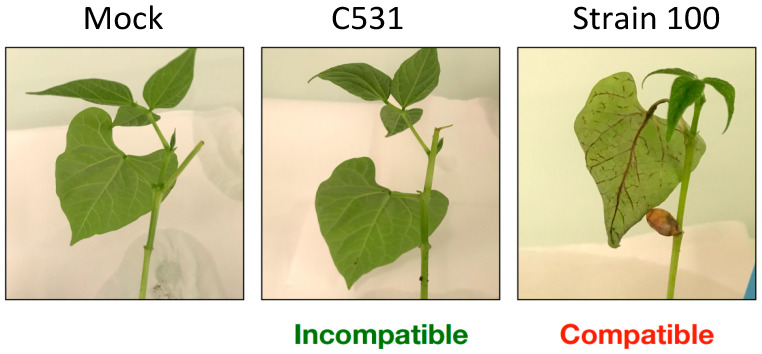
Infection characteristics of *C. lindemuthianum* on common bean plants. Typical symptoms on cotyledonary leaves at 6 days post-infection on representative BAT93 plants.

BAT93 cotyledonary leaves were spray-inoculated with *C. lindemuthianum* strain C531 (Incompatible interaction) or strain 100 (Compatible interaction), or with water as control (Mock). In compatible interaction, the first symptoms appeared at 96 h post-inoculation (hpi), as small lesions on the veins of the abaxial surface of the leaves. At six days post-infection, these small lesions further developed into large necrotic lesions (Figure 1). In contrast, no symptoms were observed in both Mock and incompatible interactions, at six days post-infection (Figure 1). In particular, no hypersensitive response was visible in incompatible interaction.

### 2.2. Quality Control of Transcriptome Analysis 

For each tested conditions (Incompatible, compatible, Mock), cotyledonary leaves were sampled at 48 and 72 hpi, before the appearance of the first symptoms, during four independent biological replicates leading to 24 RNA-seq libraries. The number of cleaned and trimmed reads per library ranged from 11 to 23 million with an average of ~16 million (Table S1). Reads were mapped to the reference genome of G19833 (v2) [3], with an average rate of 91% of uniquely mapped reads (Table S1). Samples from Mock and inoculated plants formed distinct groups after principal component analysis (PCA), confirming the similarity between replicates (Figure S1).

### 2.3. Differentially Expressed Genes in Response to C. lindemuthianum in Common Bean

**Figure 2:**
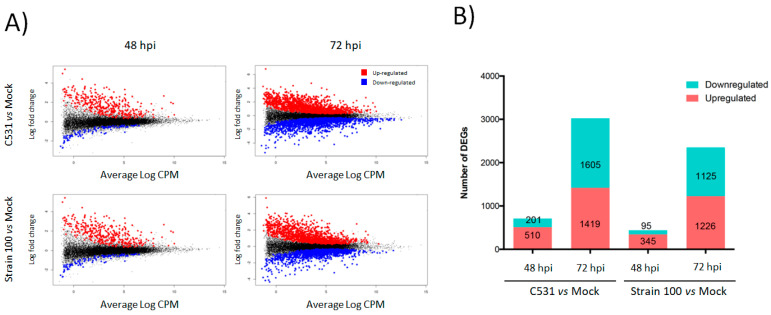
Global overview of the *P. vulgaris* transcriptomic response to inoculation with *C. lindemuthianum* under compatible (Strain 100) and incompatible interaction (C531). (**A**) LogCPM expression vs. Log2 fold change plots (MA-plots) were calculated for inoculated vs. Mock condition at each time point. Significant DEGs with adjusted *p*-value < 0.05 are plotted in red (Up-regulated) and blue (Down-regulated). (**B**) Numbers of significant DEGs in inoculated plants vs. Mock condition at each time point.

In order to study the transcriptional dynamics after infection with *C. lindemuthianum* in common bean, we performed a differential gene expression analysis during incompatible and compatible interactions. To do this, fold change expression of genes modulated after inoculation with the strain C531 or strain 100, were compared to Mock plant at two-time points, 48 and 72 hpi. After inoculation with *C. lindemuthianum*, a total of 3891 genes out of 27,433 genes (Counts per million mapped reads (CPM) > 10) were significantly differentially expressed (DEGs) in at least one of the performed pairwise comparisons (Table S2). In both conditions of infection, the number of DEGs (up- and down-regulated) was higher at 72 h than at 48 h (Figure 2A). Indeed, in plants inoculated with strain 100 (compatible interaction) compared to Mock, 440 and 2351 DEGs were identified at 48 and 72 hpi, respectively. Similarly, in plants inoculated with strain C531 (Incompatible interaction) compared to Mock, differential expression analysis identified 711 and 3024 DEGs at 48 and 72 hpi, respectively (Figure 2B). Moreover, the number of DEGs was globally higher in incompatible interaction than in compatible interaction, both at 48 and 72 hpi (Figure 2B).

**Figure 3:**
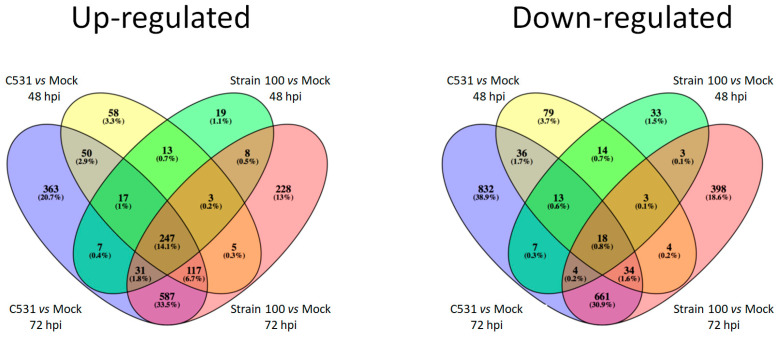
Venn diagram illustrating the comparison between down-regulated and up-regulated DEGs between BAT93 inoculated plants (Strain 100, compatible interaction, and C531, incompatible interaction) vs. Mock at 48 hpi and 72 hpi.

Among the up-regulated DEGs, an important proportion (14.1%) (*n* = 247) are common between the two conditions of infection (compatible and incompatible) and the two time points (48 and 72 hpi) (Figure 3). On the contrary, most down-regulated genes are specific to each group, with only 0.8% (*n* = 18) of the down-regulated DEGs shared between the four pairwise comparisons. Interestingly, almost one third of DEGs (33.5% and 30.9% of the up-regulated and down-regulated DEGs, respectively) are common between compatible and incompatible interaction at 72 hpi (Figure 3).

### 2.4. Gene Ontology (GO) Enrichment in Down-Regulated and Up-Regulated DEGs

**Figure 4:**
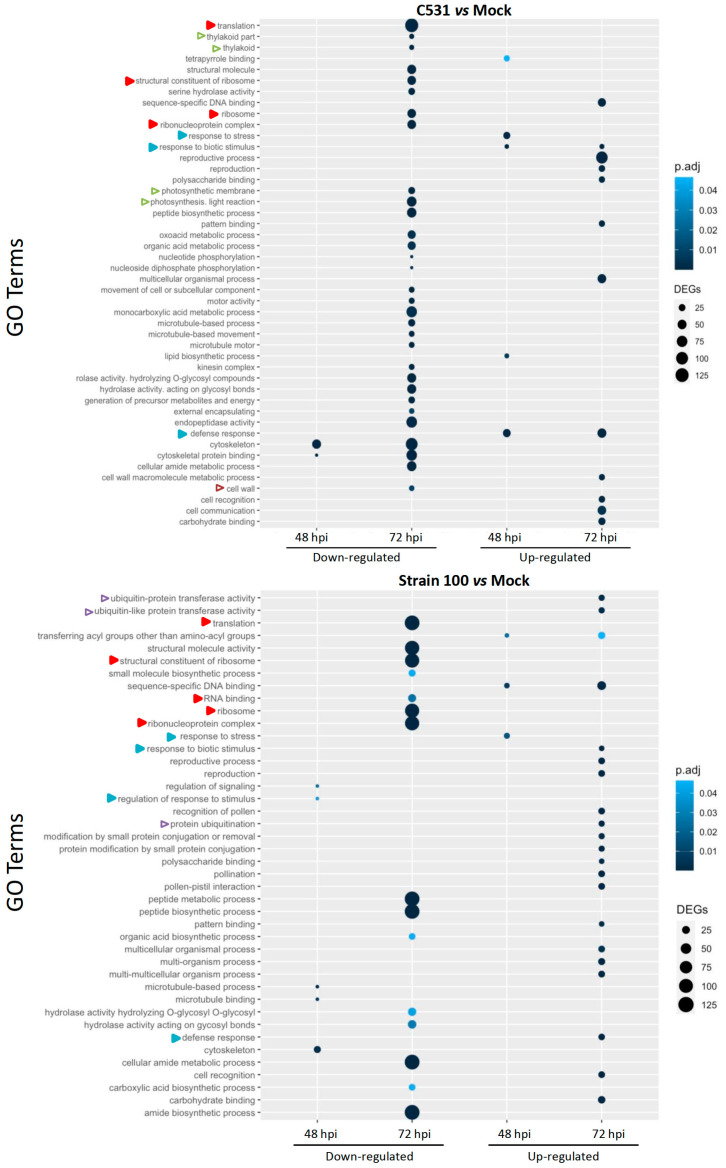
Gene Ontology enrichment analysis on DEGs from BAT93 plants, inoculated with *C. lindemuthianum* strain C531, incompatible interaction, or strain 100, compatible interaction, vs. Mock. Empty and full arrows represent enriched GOs that are specific to one infection condition or common between the two infection conditions, respectively. Color of the arrows represent different cellular processes: photosynthesis in green, translation in red, ubiquitination in purple, cell wall in brown and plant defense in blue.

The Gene Ontology (GO) system is an international standardized gene functional classification system dynamically updated. To determine the functions of DEGs involved in the response of common bean against *C. lindemuthianum*, we performed a GO enrichment analysis. Globally, GO terms analysis highlighted a diversity of biological processes at 72 hpi in both contexts of infection, while few were identified at 48 hpi (Figure 4). In the incompatible context (strain C531), down-regulated DEGs exhibit enrichment in a diversity of GO terms, whereas in the compatible context (strain 100), down-regulated GO terms are less diverse but contain more DEGs per GO term (Figure 4). GO terms specifically enriched in the resistant or susceptible context were identified. The incompatible context is specifically linked to down-regulation of photosynthetic process (thylakoid part, thylakoid, photosynthetic membrane, photosynthesis, light reaction) and cell-wall modification. The compatible context seems to be specifically associated with up-regulation of ubiquitination pathway, ubiquitin-like transferase activity and protein ubiquitination. However, some GO terms are enriched in both contexts, for example, down-regulated DEGs related to cytoskeleton and microtubules, as well as the translational process (translation, ribosome, peptide biosynthesis…) or metabolic process. We can hypothesize that these DEGs could be involved in the general defense response of common bean against *C. lindemuthianum*.

Interestingly, DEGs associated with defense response (response to stress, response to biotic stress or stimulus) are found up-regulated in both the compatible and incompatible context. However, the enrichment in defense response GO terms is observed earlier (48 hpi) in the resistant context.

### 2.5. Clustering Analysis and Gene Expression Profiles

**Figure 5:**
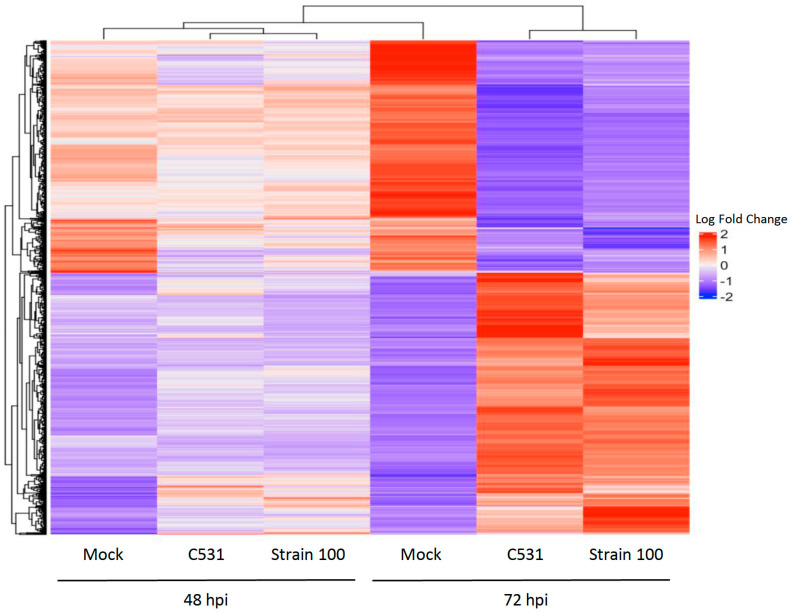
Hierarchical clustering analysis of gene expression patterns in BAT93. Heat map showing the expression levels of significant DEGs in inoculated plants vs. Mock. Up-regulated and down-regulated genes are represented in red and blue, respectively.

In order to study the expression patterns over time during incompatible and compatible interactions in common bean, we performed a hierarchical clustering analysis. A total of 3891 genes differentially expressed in at least one comparison between inoculated versus Mock were employed for clustering. Heat map representation of the clustering analysis of DEGs showed that expression patterns between inoculated and Mock conditions are quite similar at 48 hpi. However, gene expression clusters became differentiated between Mock and infected conditions at 72 hpi, showing two distinct groups of DEGs, relatively similar in both conditions of infection (Figure 5).

### 2.6. Detailed Differences between Incompatible and Compatible Interaction in Response to C. lindemuthianum in Common Bean

**Figure 6:**
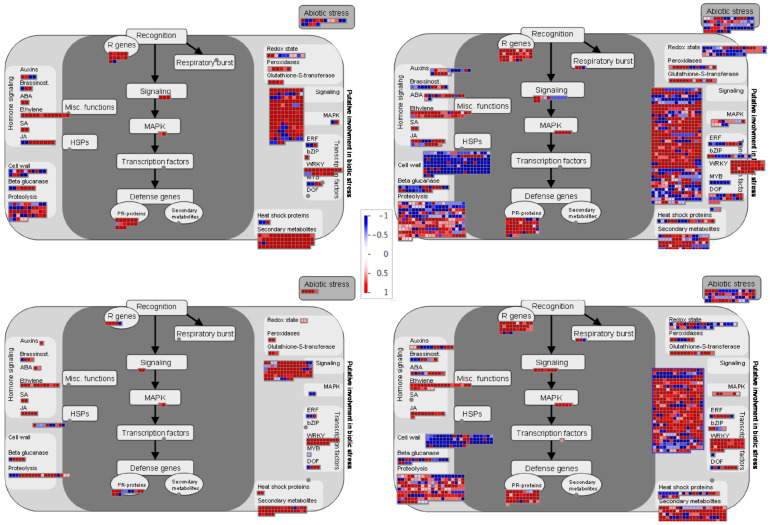
MapMan overview of the biotic stress pathway of DEGs in BAT93 inoculated plants during compatible and incompatible interactions at 48 hpi and 72 hpi. DEGs are represented by squares colored in red (Up-regulated) or blue (Down-regulated) following the scale bar displaying changes in gene expression values in LogFC (Center).

To have a global overview and to visualize the different pathways regulated during incompatible and compatible interaction in common bean, we used MapMan visualization. In agreement with clustering and GO analysis, the DEGs correspond to same pathways in both incompatible and compatible interactions. However, at 48 hpi genes related to proteolysis, signaling, secondary metabolism and cell wall are significantly enriched in resistant plants compared to susceptible plants (Figure 6). The enrichment in these pathways is even higher at 72 hpi, with more DEGs in the incompatible context compared to compatible context (Figure 6). Notably, resistance was associated with a higher repression of genes involved in the cell wall modification, both at 48 h and 72 h. Additionally, *R* genes and pathogenesis-related (PRs) proteins were enriched in the incompatible context at 48 hpi.

### 2.7. Expression of Pathogenesis-Related (PRs) Proteins and NLRs in Response to C. lindemuthianum

To investigate the behavior of genes known to be involved in the resistance/defense response, we examined the expression profiles of NLRs and PRs. We observed that a total of 48 NLRs were differentially expressed after inoculation with *C. lindemuthianum* in at least one comparison (Table 1). In resistant plants, 10 NLRs were differentially expressed (8 up- and 2 down-regulated) at 48 hpi. In susceptible plants, 8 NLRs were significantly differentially expressed (3 up- and 5 down-regulated) at 48 hpi (Table 1). Thus, at 48 hpi, more NLRs are up-regulated in the resistant plants. At 72 hpi, 37 NLRs (all up-regulated) and 32 NLRs (28 up and 4 down-regulated) were significantly differentially expressed during incompatible and compatible interaction, respectively (Table 1). Strikingly, roughly speaking, the same set of NLRs were up-regulated in both contexts of infection, with more NLRs up-regulated at 72 hpi compared to 48 hpi.

Concerning PR proteins, 25 DEGs corresponding to PR were identified (Table 2). Notably, most of them correspond to PR10/Bet v I (15 DEGs), 6 correspond to PR5 and the 4 remaining are PR1. At 48 hpi, 14 PRs (13 up- and 1 down-regulated) and 8 PRs (all up-regulated) were differentially expressed during incompatible and compatible interaction, respectively (Table 2). Eight of them were induced in both the susceptible and resistant contexts but with higher Log2FC in resistant context. At 72 hpi, 21 PRs were induced during incompatible (16 up- and 5 down-regulated) and 18 PRs during compatible interaction (16 up- and 2 down-regulated) (Table 2). Similarly to what was observed at 48 hpi, most of them were common to both resistant and susceptible contexts but with higher Log2FC in resistant context. In addition, for a given context (resistant or susceptible), higher Log2FC were observed for common DEGs at 48 hpi compared to 72 hpi. All these results are consistent with the MapMan and the GO enrichment terms analysis, suggesting that although some *R* and PR genes can be common to both resistant and susceptible contexts, resistant plants develop an earlier and stronger defense response.

### 2.8. Defense-Related Plant Hormones

Many genes involved in the ethylene pathway are induced after *C. lindemuthianum* infection, both in compatible and incompatible interaction, especially at 72 hpi (Table 3). Conversely, few genes involved in the Salicylic Acid (SA) pathway are differentially expressed, except the gene dimethylxanthine methyltransferase (Phvul.008G057600) and two PALs (Phvul.001G177700 and Phvul.001G177800) that were strongly up-regulated (Table 3). Concerning Jasmonic Acid (JA) pathway, a more marked contrast was observed between resistant and susceptible plants: at 72 hpi, 11 genes of this pathway are found differentially expressed in the resistant plants whereas only 6 DEGS are detected in susceptible plants (Table 3). Interestingly, half of the DEGs correspond to down-regulated genes in the resistant plants, suggesting a repression of the JA pathway in resistant plants at 72 hpi.

### 2.9. Validation of RNA-Seq Results with Quantitative Real-Time PCR (RT-qPCR)

**Figure 7:**
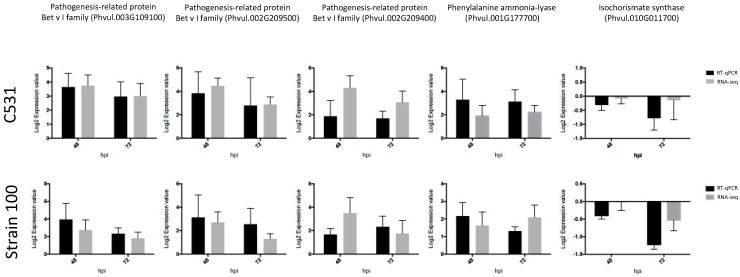
Validation of RNA-seq results by RT-qPCR in BAT93 plants in response to inoculation with *C. lindemuthianum.* Expression data were normalized using two housekeeping genes (*Act* and *UBI*) and calibrated relative to Mock.

To confirm the normalized gene count values obtained from RNA-seq data, we performed RT-qPCR using five *C. lindemuthianum* responsive common bean genes. First, we selected three PRs Bet v I (Phvul.003G109100; Phvul.002G209500 and Phvul002G209400) up-regulated after *C. lindemuthianum* infection (Table 2). Interestingly, these PRs have also been described as induced after infection with various other pathogens in common bean [22,24,25,26]. By examining the expression of these genes by RT-qPCR, we observed that RT-qPCR and RNA-seq data exhibit very similar expression profiles in all the tested conditions (Figure 7). Thus, we confirmed the up-regulation of these three PRs after infection by *C. lindemuthianum* (Figure 7).

In addition to PR genes, we also paid particular attention to two genes involved in the biosynthesis of SA, a plant hormone reported to play important role in disease resistance [27]. Indeed, PR1, considered as a marker for SA biosynthesis [28], was identified in our DEG genes (Table 2). SA can be derived from two possible pathways: the isochorismate synthase (ICS) and phenylalanine ammonia-lyase (PAL) pathway. Our RNA-seq data revealed that the expression of ICS (Phvul.005022900) was not significantly modified after *C. lindemuthianum* infection, whereas the PAL genes (Phvul.001G177700 and Phvul.001G177800) were strongly up-regulated, especially at 72 hpi (Table 3). RT-qPCR analysis on these genes gave similar expression profiles as the RNA-seq data, thus confirming the differential expression between ICS and PAL (Figure 6). This strongly suggests that in common bean infected by C. *lindemuthianum*, the SA biosynthesis should be mediated by the PAL pathway.

In conclusion, all five selected genes showed similar trend in RT-qPCR and RNA-seq data, supporting the RNA-seq data.

## 3. Discussion

In this work, we used RNA-Seq data to investigate the transcriptomic response of common bean after infection with the hemibiotrophic fungus *C. lindemuthianum* during compatible and incompatible interaction. The time points selected (48 and 72 hpi) correspond to the central and late biotrophy phase of the infection cycle. RT-qPCR was used to validate the expression patterns of several selected genes and the results showed an excellent agreement with deep sequencing.

### 3.1. PR Proteins and Hemibiotrophic Pathogens

Since their identification and description in tobacco, PR proteins have been proven to be hallmarks of the plant defense response under pathogen attack [29,30]. These molecules, currently classified in 17 families according to the protein domains they contain, have been shown to display various potential antimicrobial activities such as proteinases or cell-wall-degrading enzymatic capacities. Furthermore, two distinct and antagonistic pathways, one salicylic acid (SA)-dependent and one ethylene/jasmonic acid (JA)-dependent, have been shown, mainly in the *A. thaliana* plant model, to globally govern their regulation [31,32]. RNA sequence analyses involving genuine hemibiotrophic fungal plant pathogen species, either of the *Colletotrichum* genus, such as *Colletotrichum higginsianum* on *Arabidopsis thaliana* [33], *Colletotrichum graminicola* on maize [34], or the rice blast fungus *Magnaporthe oryzae* on rice [35] have been published. These studies showed induction of genes belonging to various PR protein families. However, no clear common specific pattern could be drawn in relation to the fact that (i) they all involve genuine hemibiotrophic pathogens, that (ii) they involve different fungal species of the same genus (*Colletotrichum*) or fungal from different genera (*Colletotrichum* versus *Magnaporthe*), that (iii) the host plants belong to the monocot clade: maize and rice versus Arabidopsis which is a dicot. For example, in the *A. thaliana/C. higginsianum* interaction PR4 and PDF1.2 (PR-12) are quickly up-regulated, whereas PR2 and PR5 are hardly (PR5), if any (PR2), slightly induced in the late necrotrophic phase of the interaction, while in maize/*C. graminicola* PR1, PR5 and PR10 were up-regulated and in *M. oryzae*/rice multiple PR proteins belonging to many families are induced, with a predominance of PR1 members.

Likewise, we identified DEGs for 25 PR encoding genes (Table 2), including 15 PR10/Bet vI, 3 PR1 and 6 PR5. Most are induced during the biotrophic phase early (48 hpi and 72 hpi Table 2 upper third) or lately (72 hpi Table 2 middle third). Interestingly, the lower third of Table 2 groups genes are down-regulated at the end of the biotrophic phase, especially in incompatible interaction. This biological fact has rarely been reported and/or discussed in literature [36].

The study of Narusaka et al. [33] demonstrated that the *C. higginsianum/A. thaliana* interaction displays induction of PR proteins and that it leans more towards the JA pathway (PDF1.2 and PR4) than towards the SA pathway (PR5 and PR2). In our study, we observed the induction of PR protein families of different plant hormone-dependent defense pathways, such as Ethylene but also JA and SA (Figure 6). Therefore, PR proteins induction in the *C. lindemuthianum*/common bean interaction does not seem to rely on one preferential pathway. In addition, even within the numerous members of the PR 10 (Bet vI) family, individual members show variable up- versus down-regulation (Table 2). The same holds true for the defense-related plant hormone genes, in particular from the JA pathway (Table 3).

Interestingly, out of 25 PR encoding genes (Table 2), 18 PR were common with Padder [24]. This suggests that the *Co-1/Co-x* [24] (encoding a CRINCKLY4 kinase) and the *Co-9*-mediated resistance (co-located with a NLR cluster) activate similar pathways. This reinforces the hypothesis that *Co-x* encodes a virulence target involved in classical NLR-mediated resistance [19].

Another noticeable result is the globally earlier and/or stronger induction of the same PR proteins in the incompatible interaction with the C531 strain (resistance of the plant) versus the compatible interaction with the strain 100 (susceptibility of the plant). This is in agreement with previous work done with non-PR proteins defense-related genes on the same pathosystem showing that PAL or chalcone isomerase display an earlier and stronger induction in incompatible interactions [37,38], or with works done on other pathosystems involving hemibiotrophic fungi such as *Cladosporium fulvum* on tomato using apoplastic PR proteins as markers [39,40]. Altogether, our results suggest that the difference between the compatible and incompatible reaction is more a question of timing and strength, than a massive difference in differentially expressed genes between these two contexts.

### 3.2. Importance of PR10/Bet vI in Common Bean Defense

Our RNA-seq results point out the importance of PR10/Bet vI proteins in the *P. vulgaris/C. lindemuthianum* interaction, with 15 DEGs corresponding to this PR family (Table 2). Interestingly, this is in agreement with other studies in common bean, showing an up-regulation of several PR10/Bet vI proteins after infection with various pathogens including not only *C. lindemuthianum* but also another fungus such as *Fusarium oxysporum* and the virus BCMV [22,24,25]. Bet vI proteins are related to the major allergen from birch pollen Bet vI and belong to the widespread PR10/Bet vI family [41]. They constitute a multigene family in plant genome with for example 46, 36, 25 and 14 members in common bean, *Medicago truncatula*, Arabidopsis and rice, respectively. These cytoplasmic PR proteins are regulated by various abiotic and biotic stresses [42]. This up-regulation of PR10/Bet vI genes has been reported after infection with various pathogens including viruses, bacteria and fungi, as well as in a large range of plant species including monocots and dicots [43,44,45,46,47,48,49]. Even if the most commonly reported biological function of PR10/Bet vI proteins is anti-pathogen activity, the mechanism of action remains unknown. Interestingly, some PR10/Bet vI proteins exhibit ribonuclease activity that could be responsible for anti-viral or anti-fungal activity [44,50]. More recently, the functional scope of the diverse PR10/Bet v1 protein superfamily has greatly expanded, in part by understanding their structural conformation. Indeed, despite low sequence identities suggesting a high evolutionary rate, the conserved secondary and tertiary structure of several Bet vI variants highlighted the presence of a large hydrophobic cavity. This important feature of PR10s allows the binding, storage and transport of various ligands, including phytohormones, proteins, fatty acids, phenolics (including flavonoids) and several classes of alkaloids [51,52,53,54,55,56,57,58,59,60,61]. These specific interactions with pathway intermediates can thus modulate the corresponding biochemical pathways leading anti-pathogen activity in an indirect manner [62].

### 3.3. In Infected Common Bean, SA Accumulation Is Based on the PAL Pathway

Our RNA-seq analysis, confirmed by RT-qPCR experiments, showed that after infection two PAL genes were strongly up-regulated while ICS expression was not significantly modified. This strongly suggests that in common bean infected with *C. lindemuthianum*, SA accumulation (data not shown) is mediated by the PAL pathway. These results in common bean contrast with those obtained in Arabidopsis in which ICS pathway is responsible for pathogen-induced SA accumulation [27]. Interestingly, soybean shows equally important roles for the ICS and PAL pathway in its SA accumulation [63]. Consequently, the importance of both pathways differs between plant species, rendering it hard to make generalizations about SA production that cover the entire plant kingdom [27].

### 3.4. Up-Regulation of NLR after Pathogen Infection

After *C. lindemuthianum* infection, a group of ~30 NLR genes were found differentially expressed in both compatible and incompatible interaction (Table 2). In a previous study, we showed that in common bean, half of the NLR genes (197/364) present an atypical pattern of DNA methylation, reminiscent of the methylation pattern observed on repeated sequences [4]. Moreover, among these methylated NLR genes, 38% (76/197) were also abundantly targeted by 24 nt siRNA. This led us to propose the existence of a transcriptional gene silencing mechanism of NLRs through the RdDM (RNA-directed DNA methylation) pathway in common bean that could be essential to down-regulate their expression during normal growth condition, and thus to avoid the fitness cost of resistance, in absence of pathogen attack. Since DNA methylation dynamically responds to biotic stress, we also proposed that this methylation could be withdrawn in the presence of the pathogen allowing NLR expression only when needed [4,6]. Surprisingly, among the NLRs up-regulated after infection, only three correspond to genes marked by the two RdDM epigenetic marks, i.e., DNA methylation and 24 nt siRNA ([4]; data not shown). This suggests that, contradictory to our expectations, biotic stress is not sufficient to de-repress NLR genes marked by RdDM marks. Alternatively, since ~2/3 of the DEG NLRs do not present any epigenetic marks without infection (data not shown), the induction of expression after *C. lindemuthianum* attack could be mediated by another mechanism acting at the post-transcriptional level. Indeed, seminal works in the Fabaceae and Solanaceae have demonstrated complex networks of small RNAs targeting NLR mRNAs, triggered by microRNAs (miRNAs) functioning as ‘master regulators’ [64,65,66]. The up-regulation of NLR after infection would be due to pathogen suppressors of RNA silencing that hijack plant sRNA pathways at diverse steps, and suppress plant immunity [67]. However, plants may have sneakily co-opted this pathogen interference: after infection, suppressors would diminish silencing at all levels, including the miRNA/phasiRNA silencing cascade, leading to an increase in NLR gene transcript levels. In that context, the NLR up-regulated in the present study could correspond to NLR silenced by the miRNA/phasi system in the absence of pathogen, and up-regulated after *C. lindemuthianum* infection due to its suppressor of silencing.

### 3.5. C. lindemuthianum Resistance in Common Bean Involved Down-Regulation of Photosynthesis, Ubiquitination-Mediated Proteolysis and Cell Wall Modifications

Our results clearly show a repression of photosynthetic processes in incompatible interaction. Such a decrease in photosynthetic activity during the incompatible interaction has already been reported in other studies [68,69,70]. In particular, in common bean, a similar response was observed after infection with various types of pathogens such as the bacteria *Xanthomonas phaseoli*, the Bean Common Mosaic Virus (BCMV) and the soybean cyst nematode [21,22,71]. In Arabidopsis, it has been shown that photosynthesis suppression after a biotic stress is mediated by MPK3/MPK6, two kinases that seem to be critical for ETI [72]. Thus, inhibition of photosynthesis is part of the plant immunity by orchestrating the trade-off between plant growth and plant defense [72,73,74].

The cell wall constitutes a physical barrier against pathogens and thus plays an important role in plant immunity. However, plant pathogens, especially the hemibiotrophic fungus *Colletotrichum*, can produce many types of cell-wall-degrading enzymes, and in several plant diseases these enzymes play a major role in the infection process development of symptoms [75,76]. In common bean, repression of genes related to cell wall modification was observed in resistant plants after infection by the fungus *Colletotrichum* (our study) but also by *Xanthomonas phaseoli* [21]. This could prevent the propagation of pathogen, by stabilizing the cell wall.

Ubiquitination-mediated proteolysis is a key for (positive and negative) regulation of oxidative burst, hormone signaling, gene induction and programmed cell death, involved in plant immunity [77,78]. In our study, expression of genes related to ubiquitination is up-regulated in common bean infected by *C. lindemuthianum*. Induction of U-box domain-containing proteins has also been reported in both *Vitis vinifera* and *Nicotiana benthamiana* in response to the grapevine leafroll-associated virus 3 (GLRaV-3) [79]. Thus, activation of the ubiquitination system (UBS) could represent a mechanism to cope with pathogens attack. This is emphasized by the identification of pathogen effectors targeting of the UBS [77,80].

## 4. Materials and Methods

### 4.1. Biological Material and Plant Inoculation

BAT93 is a common bean Mesoamerican breeding line developed at the Centro International de Agricultura Tropical (CIAT, Cali, Colombia). BAT93 possess the *Co-9 R* gene of resistance against strain C531 (incompatible interaction) of *C. lindemuthianum* [13], while it is susceptible to strain 100 (compatible interaction) [19]. La Victoire is a french cultivar of Andean origin developed by the seed company “Tezier” (Valence-sur-Rhône, France), highly susceptible to both strains 100 and C531 and included as control. Plants were sowed in soil and grown for 7 days in a growth chamber at 23 °C, 75% of humidity and with 8 h dark and 16 h light photoperiods under fluorescent tubes (166lE). Seven days post-sowing seedlings were inoculated with one of the two strains of *C. lindemuthianum*, C531 or strain 100, as previously described in Richard et al. [81]. Briefly, both sides of the two cotyledonary leaves were spray-inoculated with a spore suspension (2 × 10^6^ spores.mL^−1^) or with water alone (Mock). For each condition (BAT93 compatible; BAT93 incompatible; BAT93 Mock), four cotyledonary leaves from 4 inoculated plants were sampled, pooled and flash frozen in liquid nitrogen at 48 and 72 h post-infection (hpi). To confirm the virulence of the fungal strains, symptoms were observed on BAT93 and La Victoire at 7 days post-inoculation (dpi). Four biological replicates were independently prepared.

### 4.2. RNA Isolation, Library Preparation and Sequencing

Total RNA was extracted from cotyledonary leaves using mirVana^®^ miRNA isolation kit (ThermoScientific, Vilnius, Lithuania) following the manufacturer’s instructions and was further purified using the RNA Clean & Concentrator Kits (Zymo Research^®^, Irvine, CA, USA). RNA-seq libraries were constructed by the POPS platform (IPS2) using the TruSeq Stranded mRNA library prep kit (Illumina^®^, San Diego, CA, USA) according to the supplier’s instructions. In total, 24 libraries were constructed from inoculated and Mock samples (BA93 compatible; BAT93 incompatible; BAT93 Mock), at two time points post-infection (48 and 72 hpi) and in four independent biological replicates. The libraries were sequenced in paired-end (PE) mode with 75 bases for each read on a NextSeq500 to generate between 10 and 20 million PE reads per sample. Libraries were sequenced on an Illumina NextSeq500 using a paired-end (PE) read length of 2 × 75 bp. Lane distribution and barcoding gave approximately 10–20 million PE reads per sample.

Adapter sequences and bases with a Q-Score below 20 were trimmed out from reads using Trimmomatic (v0.36, [82]) and reads shorter than 30 bases after trimming were discarded. Reads corresponding to rRNA sequences were removed using sortMeRNA (v2.1, [83]) against the silva-bac-16s-id90, silva-bac-23s-id98, silva-euk-18s-id95 and silva-euk-28s-id98 databases.

Filtered reads were then mapped and counted using STAR (v2.7.3a, [84]) with the following parameters --alignIntronMin 5 --alignIntronMax 60000 --alignMatesGapMax 6000 --alignEndsType Local --outFilterMultimapNmax 20 --outFilterMultimapScoreRange 0 --outSAMprimaryFlag AllBestScore --mismatchNoverLmax 0.6 on the common bean reference genome G19833 (v2) [3] and its associated GTF annotation file.

### 4.3. Analysis of Differentially Expressed Genes

Genes with less than 1 read per million in at least half of the samples were discarded. The resulting raw count matrix was fed into edgeR [85] for differential expression testing by fitting a negative binomial generalized log-linear model (GLM) including a genotype factor, an inoculation factor and its interaction as well as a replicate factor to the TMM-normalized read counts for each gene. The distribution of the resulting *p*-values followed the quality criterion described by Rigaill et al. [86]. Genes with an adjusted *p*-value (FDR, [87]) below 0.05 were considered as differentially expressed. Normalized CPMs were used for hierarchical clustering and heat map generation using R (v3.6.1) [88].

### 4.4. Gene Ontology Enrichment

Gene Ontology (GO) enrichment analysis of significant DEGs for each pairwise comparisons (compatible or incompatible versus Mock) at the two time points (48 hpi and 72 hpi) were performed by using ClusterProfiler (v4.0, [89]). Hypergeometric tests were performed on DEGs from the different pairwise comparisons and *p*-values were adjusted with the Benjamini-Hochberg procedure to obtain false discovery rates (FDRs). GO terms represented by a minimum of 10 DEGs and a False Discovery Rate (FDR) < 0.05 were considered significantly enriched. Enriched GO terms belonging to biological processes (BP), molecular function (MF) or cellular component (CC) were summarized using REVIGO, a computational approach that summarizes long GO lists by reducing functional redundancies (http://revigo.irb.hr, accessed 7 April 2022) [90]. Functional annotation of significant DEGs were visualized using MapMan (v3.6.0) [91] (https://mapman.gabipd.org/, accessed 7 April 2022). Lists of DEGs associated with plant hormone signaling were extracted using corresponding MapMan Bin codes. PRs used in this study were retrieved from Phytozome (v13) database, using G19833 (v2), searching “Pathogenic related protein” as keyword. The complete set of G19833 NLRs was retrieved according to Richard et al. [4].

### 4.5. Real-Time Quantitative PCR (RT-qPCR)

To validate the RNA-seq results, RT-qPCR assays were performed on five selected genes representing contrasting pattern of expression (induction or not) and relevant according to previous publications: 3 PR10/Bet vI (Phvul.003G109100; Phvul.002G209500; Phvul.002G209400), PAL (Phvul.001G177700) and ICS (Phvul.010G011700). Expression analysis by reverse transcription followed by quantitative PCR, was performed as previously described in Richard et al. [19], except for RNA, that are the same as the ones used for the present RNA-seq analysis, extracted according to what is described in “*RNA isolation, library preparation and sequencing*” section. RT-qPCR specific primer-pairs were designed from these selected genes using Primer3 (v0.4.0) and checked for their specificity using primer BLAST [92] (Table S3). Gene expression was normalized with two reference genes (*PvIDE* and *PvAct11)* [93] (Table S3). Gene expression in Mock treatment was used to calibrate gene expression data (RT-qPCR or RNA-seq) in infected plants for each gene and time point. Relative gene expression in inoculated leaves compared with Mock leaves was calculated using the method 2^−ΔΔCt^ on four biological replicates and three technical replicates [94].

## 5. Conclusions

In the present study, the whole transcriptome of common bean infected with the hemibiotrophic fungus *C. lindemuthianum* was characterized at 48 and 72 hpi, corresponding to central and late biotrophy phase of the infection cycle. Our results highlight the prominent role of PR10/Bet vI in this interaction. A bibliographic survey pointed out that PR10/Bet vI are found up-regulated in various plant–pathogen interactions. Consequently, the importance of PR10/Bet vI in common bean and more generally in legume species may have been overlooked in part because of the supremacy of PR1 from Arabidopsis in the field of plant–pathogen interaction. Similarly, based on data from Arabidopsis, publications often generalize that the ICS pathway is responsible for basal and pathogen-induced SA accumulation in plants. However, in common bean, our results suggest that SA biosynthesis is based on the PAL pathway instead of the ICS pathway. Different plant hormone-dependent defense pathways, such as Ethylene but also JA and SA, were involved in the defense response, and common bean response to infection also involved down-regulation of photosynthesis, ubiquitination-mediated proteolysis and cell wall modifications. Importantly, our results suggest that the difference between the compatible and incompatible reaction is more a question of timing and strength, than a massive difference in differentially expressed genes between these two contexts.

## Figures and Tables

**Table 1:** List of significantly differentially expressed NLRs in compatible and incompatible interaction, at 48 hpi and 72 hpi.

										Incompatible				Compatible			
										C531 vs. Mock 48 hpi		C531 vs. Mock 72 hpi		Strain 100 vs. Mock 48 hpi		Strain 100 vs. Mock 72 hpi	
Gene ID	Annotation							Methylation Status	24nt siRNA	LogFC	FDR	LogFC	FDR	LogFC	FDR	LogFC	FDR
Phvul.002G098200	PTHR23155:SF414 LEUCINE-RICH REPEAT-CONTAINING PROTEIN							UM	None	1.96	4.56E-03	1.95	9.53E-04	1.45	7.98E-02	1.78	3.97E-03
Phvul.011G201900	PTHR23155:SF414 LEUCINE-RICH REPEAT-CONTAINING PROTEIN							UM	None	1.85	1.08E-02	0.39	6.31E-01	0.37	8.23E-01	0.57	4.26E-01
Phvul.002G133400	PTHR23155:SF633 LEUCINE-RICH REPEAT-CONTAINING PROTEIN							UM	None	1.62	2.71E-03	1.46	2.05E-03	1.58	5.42E-03	1.33	7.01E-03
Phvul.008G020700	PTHR23155:SF497 LEUCINE-RICH REPEAT-CONTAINING PROTEIN							UM	None	1.45	1.74E-02	2.38	1.40E-06	1.52	1.67E-02	1.94	1.36E-04
Phvul.005G117900	PTHR23155:SF497 LEUCINE-RICH REPEAT-CONTAINING PROTEIN							UM	None	1.41	4.96E-02	1.93	3.73E-04	1.33	9.20E-02	1.73	2.47E-03
Phvul.011G014400	PTHR23155:SF402 DISEASE RESISTANCE PROTEIN RPP13-RELATED							UM	None	1.08	5.96E-03	1.05	2.16E-03	0.92	3.83E-02	1.10	2.06E-03
Phvul.010G024100	PF00931//PF01582 NB-ARC domain (NB-ARC)//TIR domain (TIR)							UM	None	0.95	8.43E-03	0.70	2.90E-02	0.64	1.60E-01	0.43	2.22E-01
Phvul.009G233700	PTHR23155:SF402 DISEASE RESISTANCE PROTEIN RPP13-RELATED							UM	None	0.84	3.76E-02	1.27	6.95E-05	0.82	5.69E-02	1.35	2.94E-05
Phvul.007G254700	PTHR23155:SF563 LEUCINE-RICH REPEAT-CONTAINING PROTEIN							UM	None	1.74	6.79E-02	1.75	1.53E-02	1.25	2.87E-01	2.04	6.30E-03
Phvul.002G171400	PTHR11945:SF169 MADS-BOX FAMILY PROTEIN							UM	None	1.54	7.79E-02	1.78	1.06E-02	0.74	5.56E-01	1.78	1.36E-02
Phvul.008G020900	PTHR23155:SF497 LEUCINE-RICH REPEAT-CONTAINING PROTEIN							UM	None	2.17	6.08E-02	1.74	3.75E-02	1.89	1.59E-01	1.40	1.15E-01
Phvul.008G020750	PTHR23155:SF497 LEUCINE-RICH REPEAT-CONTAINING PROTEIN							N.A	N.A	1.33	1.35E-01	1.71	8.52E-03	1.32	1.83E-01	0.92	2.23E-01
Phvul.003G129700	PTHR11017:SF169 DISEASE RESISTANCE PROTEIN-RELATED							UM	None	1.32	3.43E-01	2.33	1.07E-02	1.49	3.04E-01	2.63	5.49E-03
Phvul.010G028200	PF00931//PF13676 NB-ARC domain (NB-ARC) TIR domain (TIR_2)							UM	None	1.24	1.03E-01	1.21	3.57E-02	0.81	4.07E-01	1.10	7.08E-02
Phvul.001G132301	PTHR23155:SF563 LEUCINE-RICH REPEAT-CONTAINING PROTEIN							N.A	N.A	1.17	3.09E-01	1.66	4.42E-02	0.94	5.13E-01	2.21	6.15E-03
Phvul.011G014500	PTHR23155:SF402 DISEASE RESISTANCE PROTEIN RPP13-RELATED							UM	None	0.92	1.00E-01	1.24	3.83E-03	0.94	1.12E-01	1.43	1.11E-03
Phvul.002G133600	PTHR23155:SF633 LEUCINE-RICH REPEAT-CONTAINING PROTEIN							CG-Meth	None	0.68	3.15E-01	1.24	6.35E-03	0.55	4.80E-01	1.36	4.14E-03
Phvul.004G048000	PTHR23155:SF554 LEUCINE-RICH REPEAT-CONTAINING PROTEIN							C-Meth	Yes	0.60	6.64E-01	1.94	1.71E-02	0.41	8.17E-01	2.54	2.22E-03
Phvul.004G013300	K00122 Formate dehydrogenase (FDH)							C-Meth	Yes	0.59	2.29E-01	0.81	1.61E-02	0.18	8.26E-01	0.61	9.14E-02
Phvul.003G247200	PTHR23155:SF543 LEUCINE-RICH REPEAT-CONTAINING PROTEIN							UM	None	0.57	1.32E-01	1.01	3.58E-04	0.50	2.52E-01	1.02	4.75E-04
Phvul.011G136130	K14488 SAUR family protein (SAUR)							N.A	N.A	0.47	3.47E-01	0.75	2.44E-02	0.14	8.59E-01	0.44	2.35E-01
Phvul.004G140700	PF05729//PF13676 NACHT domain (NACHT)//TIR domain							UM	None	0.42	3.74E-01	0.81	8.70E-03	0.38	4.65E-01	0.85	7.76E-03
Phvul.003G072500	PTHR12565:SF107 TRANSCRIPTION FACTOR BPE							UM	None	0.35	3.25E-01	0.50	4.40E-02	0.20	6.79E-01	0.61	1.35E-02
Phvul.010G054400	PF01582 TIR domain (TIR)							UM	None	0.33	8.02E-01	2.10	1.81E-03	0.60	6.28E-01	1.89	7.60E-03
Phvul.001G134100	PTHR23155:SF563 LEUCINE-RICH REPEAT-CONTAINING PROTEIN							C-Meth	Yes	0.32	7.09E-01	0.94	4.28E-02	0.26	8.04E-01	0.74	1.37E-01
Phvul.004G140800	PTHR11017:SF171 LEUCINE-RICH REPEAT-CONTAINING PROTEIN							C-Meth	None	0.31	6.65E-01	1.14	4.47E-03	0.23	7.93E-01	1.01	1.78E-02
Phvul.002G131000	PTHR23155:SF505 LEUCINE-RICH REPEAT-CONTAINING PROTEIN							C-Meth	None	0.31	3.72E-01	0.48	3.37E-02	0.14	7.72E-01	0.54	1.91E-02
Phvul.011G202900	PTHR23155:SF414 LEUCINE-RICH REPEAT-CONTAINING PROTEIN							C-Meth	None	0.30	5.76E-01	0.79	2.01E-02	0.24	7.14E-01	0.27	5.26E-01
Phvul.001G018800	PTHR23155:SF543 LEUCINE-RICH REPEAT-CONTAINING PROTEIN							UM	None	0.30	3.21E-01	0.51	1.53E-02	0.36	2.55E-01	0.47	3.37E-02
Phvul.011G172100	PTHR23155:SF554 LEUCINE-RICH REPEAT-CONTAINING PROTEIN							C-Meth	None	0.27	7.89E-01	0.79	1.77E-01	−0.56	5.97E-01	1.21	2.48E-02
Phvul.002G021700	PTHR23155:SF543 LEUCINE-RICH REPEAT-CONTAINING PROTEIN							UM	None	0.25	6.57E-01	0.79	1.48E-02	0.24	7.03E-01	0.78	2.15E-02
Phvul.010G101200	PTHR11017:SF174 LEUCINE-RICH REPEAT-CONTAINING PROTEIN							C-Meth	None	0.23	7.79E-01	1.07	1.51E-02	0.11	9.26E-01	1.29	4.25E-03
Phvul.002G166400	PTHR23155:SF506 LEUCINE-RICH REPEAT-CONTAINING PROTEIN							UM	None	0.22	5.45E-01	0.55	1.51E-02	0.29	4.27E-01	0.66	3.80E-03
Phvul.003G247651	PTHR23155:SF543 LEUCINE-RICH REPEAT-CONTAINING PROTEIN							N.A	N.A	0.21	6.91E-01	0.78	1.01E-02	0.36	4.79E-01	0.98	1.48E-03
Phvul.011G202966	PTHR23155:SF414 LEUCINE-RICH REPEAT-CONTAINING PROTEIN							N.A	N.A	0.21	5.19E-01	0.49	1.49E-02	0.08	8.70E-01	0.42	4.95E-02
Phvul.011G014301	PTHR23155:SF402 DISEASE RESISTANCE PROTEIN RPP13-RELATED							N.A	N.A	0.16	5.14E-01	0.36	2.02E-02	0.16	5.40E-01	0.34	3.65E-02
Phvul.011G195100	PTHR23155:SF414 LEUCINE-RICH REPEAT-CONTAINING PROTEIN							C-Meth	None	0.12	9.27E-01	1.56	1.68E-02	0.75	3.86E-01	1.29	6.74E-02
Phvul.002G323200	PTHR11017:SF162 LEUCINE-RICH REPEAT-CONTAINING PROTEIN							UM	None	0.03	9.71E-01	0.33	3.24E-01	−0.05	9.45E-01	0.62	4.08E-02
Phvul.011G193600	PF00931//PF13855 NB-ARC domain (NB-ARC)//Leucine rich repeat							C-Meth	None	0.00	9.96E-01	−0.44	9.41E-02	−0.13	8.05E-01	−0.53	4.40E-02
Phvul.010G026400	PF00931//PF13676 NB-ARC domain (NB-ARC)//TIR domain							C-Meth	None	−0.04	9.39E-01	0.58	2.51E-02	−0.15	7.78E-01	0.44	1.12E-01
Phvul.011G074800	1.3.99.12 2-methylacyl-CoA dehydrogenase/Branched-chain acyl-CoA							UM	None	−0.09	9.24E-01	0.90	2.50E-02	−0.08	9.37E-01	0.56	2.06E-01
Phvul.002G075400	KOG4308 LRR-containing protein							N.A	N.A	−0.13	7.13E-01	−0.31	1.48E-01	−0.17	6.38E-01	−0.52	1.26E-02
Phvul.011G193100	PTHR23155:SF414 LEUCINE-RICH REPEAT-CONTAINING PROTEIN							C-Meth		−0.20	5.50E-01	−0.39	7.92E-02	−0.27	4.25E-01	−0.44	4.87E-02
Phvul.011G198400	PF00931//PF13855 NB-ARC domain (NB-ARC)//Leucine rich repeat							C-Meth		−0.28	4.49E-01	−0.19	5.43E-01	−0.62	4.66E-02	−0.18	5.54E-01
Phvul.008G072300	PTHR23155:SF590 LEUCINE-RICH REPEAT-CONTAINING PROTEIN							UM	None	−0.45	4.26E-01	0.03	9.67E-01	−1.00	3.95E-02	−0.02	9.76E-01
Phvul.002G075000	PTHR23155:SF505 LEUCINE-RICH REPEAT-CONTAINING PROTEIN							UM	None	−0.47	6.08E-01	−0.67	3.39E-01	−1.51	4.62E-02	−0.63	3.63E-01
Phvul.011G191600	PF13191//PF13855 AAA ATPase domain//Leucine rich repeat							C-Meth	None	−0.60	3.96E-02	−0.03	9.38E-01	−0.65	3.26E-02	−0.18	5.65E-01
Phvul.005G005000	PTHR32472 P-LOOP CONTAINING NUCLEOSIDE TRIPHOSPHATE HYDROLASES SUPERFAMILY PROTEIN							UM	None	−1.81	3.97E-02	−1.50	1.40E-01	−2.54	4.87E-03	−3.78	2.70E-03
N.A: Not available; UM: Unmethylated; C-Meth: CHG or CHH gene-body methylated; CG-Meth: CG gene-body methylated: Underlined (purple): Methylation due to a TE inserted in an intron; Underlined (green): Methylation due to an associated repeat located in an intron.																	
FDR																	
	1.00E-10	1.00E-08	0.05	0.00													
Log2FC																	
	−2.50	−1.50	−1.00	−0.5	0.00	0.5	1.00	1.50	2.50								

**Table 2:** List of significantly differentially expressed pathogenesis-related proteins (PRs) in compatible and incompatible interaction, at 48 hpi and 72 hpi.

				Incompatible				Compatible			
				C531 vs. Mock 48 hpi		C531 vs. Mock 72 hpi		Strain 100 vs. Mock 48 hpi		Strain 100 vs. Mock 72 hpi	
Gene ID	Annotation			LogFC	FDR	LogFC	FDR	LogFC	FDR	LogFC	FDR
Phvul.002G209500 *	PF00407—Pathogenesis-related protein Bet v I family (PR10)			4.53	2.34E-19	2.80	3.29E-08	3.06	2.94E-08	1.91	3.09E-04
**Phvul.003G109603**	PF00407—Pathogenesis-related protein Bet v I family (PR10)			4.31	4.94E-04	2.99	3.98E-03	3.51	1.48E-02	2.21	4.48E-02
**Phvul.003G109200**	PF00407—Pathogenesis-related protein Bet v I family (PR10)			4.18	8.46E-10	2.94	1.54E-05	2.69	5.41E-04	2.30	1.31E-03
**Phvul.002G209400**	PF00407—Pathogenesis-related protein Bet v I family (PR10)			4.15	6.68E-10	2.84	9.35E-06	3.02	1.03E-04	2.14	1.57E-03
**Phvul.003G109800**	PF00407—Pathogenesis-related protein Bet v I family (PR10)			3.63	2.12E-03	2.84	1.29E-03	1.68	4.46E-01	1.92	6.28E-02
**Phvul.003G109000**	PTHR31339—PECTIN LYASE-LIKE SUPERFAMILY PROTEIN (Bet v 1) (PR10)			3.46	7.35E-05	2.52	1.14E-03	2.81	3.85E-03	2.85	3.11E-04
**Phvul.003G109100**	PTHR22847:SF361—JOUBERIN Bet v I family (PR10)			3.44	6.18E-09	2.62	1.13E-05	2.66	7.18E-05	1.83	3.14E-03
Phvul.002G155500	PF00314—Thaumatin family (Thaumatin) Pathogenesis-related (PR5)			2.23	9.47E-07	1.89	2.78E-05	1.84	2.63E-04	1.55	1.02E-03
Phvul.006G196900 **	K13449—pathogenesis-related protein 1 (PR1)			2.21	2.31E-05	1.28	1.19E-02	1.88	1.00E-03	0.99	6.97E-02
Phvul.002G286600	KOG4837—Uncharacterized conserved protein Pathogenesis-related (PR5)			1.97	2.35E-01	2.15	2.43E-02	1.71	3.75E-01	2.28	2.30E-02
Phvul.006G197500	PTHR10334—CYSTEINE-RICH SECRETORY PROTEIN (PR1)			1.37	3.91E-02	0.41	5.58E-01	0.90	2.96E-01	0.63	3.13E-01
**Phvul.011G183900**	PF00407—Pathogenesis-related protein Bet v I family (PR10)			0.95	5.62E-02	0.94	2.09E-02	0.73	2.20E-01	0.97	2.33E-02
**Phvul.011G183766**	PF00407—Pathogenesis-related protein Bet v I family (PR10)			0.94	1.02E-01	0.86	6.71E-02	0.78	2.45E-01	1.03	2.97E-02
**Phvul.011G183700**	PF00407—Pathogenesis-related protein Bet v I family (PR10)			0.88	4.05E-02	0.86	1.79E-02	0.70	1.66E-01	0.83	2.80E-02
**Phvul.011G183832**	PF00407—Pathogenesis-related protein Bet v I family (PR10)			0.83	8.87E-02	0.89	2.25E-02	0.71	2.08E-01	0.92	2.39E-02
Phvul.002G286500	PF00314—Thaumatin family (Thaumatin) (PR5)			0.79	4.56E-01	2.01	3.32E-03	0.51	7.00E-01	2.15	2.57E-03
**Phvul.011G184200**	PF00407—Pathogenesis-related protein Bet v I family (PR10)			0.77	1.00E-02	0.86	9.60E-04	0.62	7.75E-02	0.78	4.18E-03
**Phvul.011G183400**	PF00407—Pathogenesis-related protein Bet v I family (PR10)			0.53	2.24E-02	0.27	2.33E-01	0.40	1.49E-01	0.30	1.78E-01
Phvul.006G197200	PTHR10334—CYSTEINE-RICH SECRETORY PROTEIN (PR1)			0.20	9.41E-01	2.34	4.93E-02	0.20	9.54E-01	2.62	3.09E-02
**Phvul.011G183000**	PF00407—Pathogenesis-related protein Bet v I family (PR10)			0.15	9.29E-01	0.76	3.94E-01	−0.39	8.07E-01	1.82	2.40E-02
Phvul.008G166500	[NAD(+)], CYTOPLASMIC Pathogenesis-related (PR5)			−0.72	1.21E-01	−1.06	1.65E-02	−0.52	3.45E-01	−0.27	5.96E-01
Phvul.009G082100	Pathogenesis-related protein p14a (PR1-like)			−0.76	2.41E-01	−1.73	1.89E-04	−0.65	3.79E-01	−1.44	2.73E-03
**Phvul.011G182900**	PF00407—Pathogenesis-related protein Bet v I family (PR10)			−0.89	2.05E-02	−1.05	3.12E-03	−0.82	5.06E-02	−0.60	1.16E-01
Phvul.001G016700	PTHR31048:SF10—PATHOGENESIS-RELATED PROTEIN 5-RELATED (PR5)			−0.93	2.16E-01	−2.26	1.62E-04	−0.64	4.84E-01	−1.27	3.64E-02
Phvul.011G034200	PROTEIN AGD2-RELATED Pathogenesis-related (PR5)			−0.98	3.90E-01	−2.19	6.17E-03	−0.60	6.76E-01	−1.48	7.49E-02
*; **: Bet v I (PR10) and PR1 are presented in **bold** and underlined, respectively, while PR5 encoding genes appears in normal character.											
FDR											
	1.00E-10	1.00E-08	0.05	0.00							
Log2FC											
	−2.50	−1.50	−1.00	−0.5	0.00	0.5	1.00	1.50	2.50		

**Table 3:** List of defense-related plant hormones genes differentially expressed in at least one pairwise comparison, in compatible and incompatible interaction, at 48 hpi and 72 hpi.

				Incompatible				Compatible			
				C531 vs. Mock 48 hpi		C531 vs. Mock 72 hpi		Strain 100 vs. Mock 48 hpi		Strain 100 vs. Mock 72 hpi	
Gene ID	Annotation			LogFC	FDR	LogFC	FDR	LogFC	FDR	LogFC	FDR
**Auxins**											
Phvul.009G103800	K14488—SAUR family protein (SAUR)			2.45	4.37E-02	0.26	8.47E-01	2.01	1.59E-01	0.16	9.15E-01
Phvul.006G186600	PTHR11772//PTHR11772:SF19—ASPARAGINE SYNTHETASE			1.10	9.08E-02	1.03	5.09E-02	0.92	2.24E-01	1.13	3.62E-02
Phvul.009G001800	K14488—SAUR family protein (SAUR)			1.02	1.72E-01	2.39	3.71E-05	1.12	1.52E-01	2.41	4.03E-05
Phvul.006G142300	PTHR23130:SF80-AUXIN-INDUCED IN ROOT CULTURES PROTEIN 12			0.85	4.61E-02	1.38	5.42E-05	0.91	3.76E-02	1.44	3.37E-05
Phvul.009G001500	K14488—SAUR family protein (SAUR)			0.65	5.15E-01	1.31	5.46E-02	−0.08	9.65E-01	1.59	2.06E-02
Phvul.011G108500	PTHR10641//PTHR10641—MYB-LIKE DNA-BINDING PROTEIN MYB			0.51	6.36E-01	1.28	4.44E-02	0.50	6.76E-01	1.12	9.11E-02
Phvul.010G117500	K14488—SAUR family protein (SAUR)			0.41	5.20E-01	1.10	3.96E-03	0.19	8.32E-01	0.99	1.52E-02
Phvul.001G147300	K14487—auxin responsive GH3 gene family (GH3)			0.34	8.10E-01	1.57	3.47E-02	−0.34	8.43E-01	0.79	3.60E-01
Phvul.007G219500	PTHR12899//PTHR12899:SF4—39S RIBOSOMAL PROTEIN L18			0.34	6.94E-01	0.99	4.84E-02	0.47	5.83E-01	1.41	4.67E-03
Phvul.011G037300	PTHR23334—CCAAT/ENHANCER BINDING PROTEIN			0.11	9.10E-01	−1.39	1.16E-02	−0.19	8.46E-01	−0.40	4.98E-01
Phvul.002G209300	PTHR23335:SF12—CALMODULIN-BINDING TRANSCRIPTION ACTIVATOR 1			0.05	8.70E-01	0.26	9.24E-02	0.01	9.80E-01	0.44	2.91E-03
Phvul.011G037900	PTHR24015:SF364—ATPASE EXPRESSION PROTEIN 3			−0.22	8.14E-01	−1.18	1.58E-02	−0.10	9.41E-01	−0.93	7.20E-02
Phvul.002G147300	PTHR31933:SF3—O-FUCOSYLTRANSFERASE FAMILY PROTEIN			−0.26	3.65E-01	−0.44	2.80E-02	−0.29	3.46E-01	−0.39	6.39E-02
Phvul.008G286600	PF03188//PF04526—Eukaryotic cytochrome b561 (Cytochrom B561)			−0.30	6.39E-01	−0.94	5.06E-02	−0.23	7.54E-01	−1.05	3.26E-02
Phvul.010G125400	PF02519—Auxin responsive protein (Auxin inducible)			−0.36	6.75E-01	−0.93	7.57E-02	−0.18	8.70E-01	−1.17	2.67E-02
Phvul.003G190900	PF03634—TCP family transcription factor (TCP)			−0.37	2.34E-01	−0.61	7.03E-03	−0.31	3.93E-01	−0.42	8.54E-02
Phvul.003G127801	PTHR31374:SF22—AUXIN-RESPONSIVE PROTEIN-LIKE PROTEIN			−0.50	4.07E-01	−1.12	7.57E-03	−0.26	7.45E-01	−0.95	3.01E-02
Phvul.009G188100	PF03634—TCP family transcription factor (TCP)			−0.50	2.34E-01	−0.65	4.12E-02	−0.37	4.60E-01	−0.54	1.06E-01
Phvul.006G113100	PF13639//PF13947//PF14380—Ring finger domain (zf-RING 2)			−1.02	1.63E-02	−0.84	6.39E-02	−0.62	2.45E-01	−0.02	9.76E-01
**Brassinosteroids**											
Phvul.003G187200	PF00069//PF00560//PF08263—Protein kinase domain (Pkinase)			2.12	7.49E-03	1.99	2.30E-03	2.14	9.73E-03	2.09	2.40E-03
Phvul.002G207000	K13416—brassinosteroid insensitive 1-associated receptor kinase 1			1.39	1.98E-02	1.80	2.92E-04	1.47	1.75E-02	1.87	2.77E-04
Phvul.002G158800	PTHR14155—RING FINGER DOMAIN-CONTAINING			0.51	1.13E-01	0.12	7.38E-01	0.70	2.17E-02	0.30	2.96E-01
Phvul.009G184500	PF00069//PF00560//PF08263—Protein kinase domain (Pkinase)			0.43	6.22E-01	1.05	4.16E-02	0.70	3.92E-01	1.17	2.65E-02
Phvul.006G208100	K13416—brassinosteroid insensitive 1-associated receptor kinase 1			0.28	5.13E-01	0.56	4.35E-02	0.23	6.55E-01	0.60	3.50E-02
Phvul.002G216900	Squalene monooxygenase/Squalene epoxidase			0.04	9.81E-01	−1.82	8.58E-03	−0.44	7.44E-01	−1.24	8.65E-02
Phvul.004G067300	no data			0.00	9.97E-01	−0.79	1.24E-02	−0.16	8.18E-01	−0.88	6.79E-03
Phvul.009G056400	K14503—brassinosteroid resistant 1/2 (BZR1 2)			−0.21	6.34E-01	−0.52	5.11E-02	−0.05	9.43E-01	−0.59	3.16E-02
Phvul.007G223700	PTHR10015:SF164—HEAT STRESS TRANSCRIPTION FACTOR A-3			−0.37	2.47E-01	−0.54	2.36E-02	−0.26	5.06E-01	−0.47	6.13E-02
Phvul.011G031700	PTHR31388:SF37—PEROXIDASE 4-RELATED			−0.50	3.99E-01	−1.06	7.79E-03	−0.45	4.93E-01	−1.05	1.09E-02
Phvul.009G020000	Cycloartenol 24-C-methyltransferase/Sterol C-methyltransferase			−0.52	2.28E-01	−0.66	4.08E-02	−0.09	9.05E-01	−0.45	1.97E-01
Phvul.002G291800	PF00651—BTB/POZ domain (BTB)			−0.83	2.68E-01	−2.01	1.17E-03	−0.32	7.56E-01	−0.82	1.84E-01
Phvul.010G064300	K02728—20S proteasome subunit alpha 3 (PSMA4)			−1.57	4.87E-02	−2.23	2.24E-03	−1.70	3.67E-02	−2.21	2.72E-03
**Abscisic acid**											
Phvul.011G096800	KOG0725—Reductases with broad range of substrate specificities			5.47	1.30E-02	3.10	2.02E-02	2.86	3.51E-01	2.10	1.43E-01
Phvul.009G218800	PTHR11926:SF242—UDP-GLYCOSYLTRANSFERASE 71B2-RELATED			1.54	5.86E-03	1.45	2.20E-03	1.35	3.08E-02	1.14	2.62E-02
Phvul.004G138600	PF02893—GRAM domain (GRAM)			1.02	4.17E-01	1.83	3.34E-02	1.33	2.91E-01	2.11	1.89E-02
Phvul.008G209900	K00423—L-ascorbate oxidase (E1.10.3.3)			0.91	9.81E-02	1.05	1.53E-02	0.81	1.94E-01	1.06	1.90E-02
Phvul.001G153200	PF04570—zinc-finger of the FCS-type, C2-C2 (zf-FLZ)			0.56	4.41E-01	1.28	6.08E-03	0.30	7.60E-01	0.83	1.02E-01
Phvul.001G087100	Pleckstrin-homology domain (PH domain)			0.54	7.67E-02	0.54	3.23E-02	0.37	3.44E-01	0.75	2.35E-03
Phvul.011G097200	KOG0725—Reductases with broad range of substrate specificities			0.48	1.39E-01	0.73	2.86E-03	0.62	4.72E-02	0.53	4.50E-02
Phvul.002G122200	PTHR24286:SF10—ABSCISIC ACID 8’-HYDROXYLASE 1-RELATED			0.15	8.58E-01	1.06	6.51E-03	0.33	6.45E-01	1.13	5.44E-03
Phvul.003G191100	PTHR12300:SF52—HVA22-LIKE PROTEIN A-RELATED			−0.30	4.45E-01	−0.74	5.03E-03	−0.42	2.80E-01	−0.55	5.38E-02
Phvul.002G086700	PTHR12300:SF51—HVA22-LIKE PROTEIN C			−0.39	5.77E-01	−1.03	1.99E-02	−0.16	8.70E-01	−0.84	7.18E-02
Phvul.001G007300	PTHR12300:SF26—HVA22-LIKE PROTEIN G-RELATED			−0.48	1.91E-01	−1.07	9.85E-05	−0.51	1.90E-01	−0.75	9.87E-03
Phvul.008G190500	PTHR12300:SF27—HVA22-LIKE PROTEIN F			−0.54	3.84E-01	−1.32	3.11E-03	−0.58	3.77E-01	−1.28	5.12E-03
**Ethylene**											
Phvul.007G273000	PTHR31190:SF15—ETHYLENE-RESPONSIVE TRANSCRIPTION FACTOR 1B			3.66	1.42E-02	2.21	1.88E-02	3.00	1.19E-01	2.77	3.24E-03
Phvul.001G160100	PTHR31190:SF26—ETHYLENE-RESPONSIVE TRANSCRIPTION FACTOR			2.62	1.16E-03	1.51	2.97E-02	1.94	5.00E-02	0.23	8.06E-01
Phvul.002G326600	PTHR18934:SF112—DEA(D/H)-BOX RNA HELICASE FAMILY PROTEIN			2.29	5.13E-04	2.25	2.29E-04	2.31	8.01E-04	2.44	8.86E-05
Phvul.010G003300	no data			1.95	1.13E-02	2.72	3.41E-05	2.02	9.54E-03	3.37	2.34E-07
Phvul.007G127800	PTHR31153:SF3—HISTONE H1FLK-LIKE PROTEIN-RELATED			1.94	9.04E-05	1.34	4.47E-03	1.65	2.51E-03	1.06	3.46E-02
Phvul.004G081900	PTHR13690:SF86—TRANSCRIPTION FACTOR VIP1			1.90	3.97E-04	1.82	1.62E-04	1.40	3.11E-02	1.43	5.37E-03
Phvul.004G081900	PTHR13690:SF86—TRANSCRIPTION FACTOR VIP1			1.90	3.97E-04	1.82	1.62E-04	1.40	3.11E-02	1.43	5.37E-03
Phvul.003G020100	PTHR10209- OXIDOREDUCTASE, 2OG-FE II OXYGENASE			1.67	8.68E-03	1.42	9.56E-03	1.10	1.76E-01	1.29	2.38E-02
Phvul.003G020000	2,4-dihydroxy-1,4-benzoxazin-3-one-glucoside dioxygenase/			1.13	1.11E-01	1.65	2.01E-03	0.82	3.46E-01	1.52	6.29E-03
Phvul.009G066980	PTHR31729:SF0—ETHYLENE-RESPONSIVE TRANSCRIPTION FACTOR			1.06	7.49E-03	0.79	2.74E-02	1.13	4.87E-03	0.81	2.84E-02
Phvul.003G020200	PTHR10209—OXIDOREDUCTASE, 2OG-FE II OXYGENASE FAMILY PROTEIN			1.05	1.92E-01	1.18	5.48E-02	0.97	2.76E-01	1.47	1.85E-02
Phvul.008G127500	PTHR15898:SF1—GLUCOSE-INDUCED DEGRADATION PROTEIN 4			1.01	7.54E-02	1.27	4.09E-03	0.82	2.26E-01	0.88	6.61E-02
Phvul.009G003400	PTHR10209:SF107 FE(II)-DEPENDENT OXYGENASE-LIKE PROTEIN-RELATED			0.93	2.96E-01	1.50	1.11E-02	1.02	2.85E-01	1.34	3.11E-02
Phvul.006G183100	PF00847—AP2 domain (AP2)			0.86	1.59E-02	1.05	5.42E-04	0.75	6.43E-02	0.57	9.01E-02
Phvul.003G020300	PTHR10209//PTHR10209:SF148—OXIDOREDUCTASE, 2OG-FE II			0.81	1.34E-01	0.94	2.76E-02	0.75	2.20E-01	0.94	3.16E-02
Phvul.011G125200	no data			0.73	3.90E-02	0.65	2.54E-02	0.70	6.39E-02	0.63	3.61E-02
Phvul.001G225300	PF00582—Universal stress protein family (Usp)			0.69	1.23E-01	0.75	2.57E-02	0.41	4.92E-01	0.55	1.32E-01
Phvul.007G241600	K06228—fused (FU)			0.63	1.18E-01	0.86	5.28E-03	0.80	4.05E-02	0.79	1.58E-02
Phvul.002G055800	K09286—EREBP-like factor (EREBP)			0.54	7.35E-02	0.84	2.93E-04	0.79	3.33E-03	0.85	3.09E-04
Phvul.002G293000	K11135—Pin2-interacting protein X1 (PINX1)			0.52	5.51E-01	1.22	2.66E-02	0.59	5.20E-01	1.33	2.07E-02
Phvul.004G120700	Deacetoxyvindoline 4-hydroxylase/Desacetyoxyvindoline-17-hydroxylase			0.42	3.34E-01	0.65	2.94E-02	0.20	7.33E-01	0.50	1.15E-01
Phvul.002G055700	PTHR31190:SF30—ETHYLENE-RESPONSIVE TRANSCRIPTION FACTOR 15			0.41	2.09E-01	0.69	3.15E-03	0.70	1.35E-02	0.68	5.09E-03
Phvul.003G223624	K09286—EREBP-like factor (EREBP)			0.33	3.16E-01	0.73	9.51E-04	0.59	3.47E-02	0.88	5.54E-05
Phvul.003G223686	PTHR31190:SF30—ETHYLENE-RESPONSIVE TRANSCRIPTION FACTOR 15			0.32	4.19E-01	0.81	1.53E-03	0.57	9.77E-02	0.69	1.12E-02
Phvul.003G150900	K19044—E3 ubiquitin-protein ligase XBAT32/33 (XBAT32 33)			0.09	7.77E-01	0.36	4.76E-02	0.08	8.30E-01	0.36	5.50E-02
Phvul.002G209300	PTHR23335:SF12—CALMODULIN-BINDING TRANSCRIPTION ACTIVATOR 1			0.05	8.70E-01	0.26	9.24E-02	0.01	9.80E-01	0.44	2.91E-03
Phvul.006G179800	PF00847—AP2 domain (AP2)			−0.07	9.15E-01	0.64	3.41E-02	0.21	7.26E-01	0.84	5.53E-03
Phvul.004G121000	Deacetoxyvindoline 4-hydroxylase			−0.30	5.72E-01	−0.77	2.09E-02	−0.24	7.02E-01	−0.40	2.64E-01
Phvul.003G241700	PTHR31729:SF0- ETHYLENE-RESPONSIVE TRANSCRIPTION FACTOR			−0.34	4.94E-01	−0.65	4.84E-02	−0.20	7.54E-01	−0.63	6.26E-02
**Salicylic acid**											
Phvul.008G057600	Caffeine synthase/Dimethylxanthine methyltransferase			5.07	2.40E-05	2.95	1.04E-03	4.41	1.11E-03	1.77	7.16E-02
Phvul.001G177700	K10775—Phenylalanine ammonia-lyase (PAL)			1.61	6.78E-03	2.19	2.35E-05	1.55	1.37E-02	2.45	3.25E-06
Phvul.001G177800	K10775—Phenylalanine ammonia-lyase (PAL)			1.59	2.83E-02	2.27	1.72E-04	−0.13	1.00E+00	2.28	2.39E-04
Phvul.010G011700	5.4.4.2—Isochorismate synthase/Isochorismate synthetase			−0.02	9.77E-01	−0.60	6.19E-02	−0.08	1.00E+00	−0.61	6.64E-02
**Jasmonic acid**											
Phvul.010G134700	K15718—linoleate 9S-lipoxygenase (LOX1 5)			1.70	1.99E-02	2.25	2.48E-04	1.52	6.13E-02	1.84	3.96E-03
Phvul.006G016900	no data			1.43	1.85E-04	1.44	5.50E-05	1.18	5.46E-03	1.23	1.02E-03
Phvul.001G000800	PTHR22893//PTHR22893:SF62- NADH OXIDOREDUCTASE-RELATED			0.98	1.58E-01	1.03	4.90E-02	0.98	1.91E-01	1.01	6.17E-02
Phvul.005G156800	K15718—linoleate 9S-lipoxygenase (LOX1 5)			0.88	6.18E-01	−2.52	2.72E-02	0.19	9.46E-01	−1.01	4.37E-01
Phvul.009G262900	PTHR11771:SF38—LIPOXYGENASE 3, CHLOROPLASTIC-RELATED			0.86	1.27E-01	1.20	4.91E-03	0.70	2.92E-01	1.04	2.11E-02
Phvul.003G021000	PTHR33077:SF7—PROTEIN TIFY 6A-RELATED			0.70	6.11E-02	0.34	3.54E-01	0.56	2.06E-01	0.81	1.25E-02
Phvul.003G111500	Delta(4)-3-oxosteroid 5-beta-reductase			0.55	9.25E-02	0.64	1.21E-02	0.54	1.28E-01	0.42	1.30E-01
Phvul.005G156900	K15718—linoleate 9S-lipoxygenase (LOX1 5)			0.31	8.49E-01	−1.89	2.13E-02	−0.15	9.44E-01	−0.86	3.63E-01
Phvul.003G129200	PTHR33077:SF13—PROTEIN TIFY 10A-RELATED			0.19	8.87E-01	0.29	7.73E-01	0.39	7.54E-01	1.72	9.46E-03
Phvul.009G225300	PTHR33077:SF13—PROTEIN TIFY 10A-RELATED			0.18	6.26E-01	0.62	5.05E-03	0.24	5.20E-01	0.78	4.37E-04
Phvul.003G131600	PTHR22893:SF67—12-OXOPHYTODIENOATE REDUCTASE 3			−0.39	2.89E-01	−0.58	2.82E-02	−0.19	7.10E-01	−0.25	4.15E-01
Phvul.002G175500	PTHR11771:SF60—LIPOXYGENASE 6, CHLOROPLASTIC			−0.47	3.22E-01	−0.71	4.38E-02	−0.39	4.76E-01	−0.33	4.10E-01
Phvul.004G072000	PTHR31942:SF29—MLO-LIKE PROTEIN 12-RELATED			−0.60	9.00E-02	−0.62	3.84E-02	−0.36	4.23E-01	−0.59	5.63E-02
Phvul.010G032300	PTHR11771//PTHR11771:SF59—LIPOXYGENASE			−1.26	4.20E-03	−0.48	3.32E-01	−0.34	6.23E-01	−0.14	8.13E-01
FDR											
	1.00E-10	1.00E-08	0.05	0.00							
Log2FC											
	−2.50	−1.50	−1.00	−0.5	0.00	0.5	1.00	1.50	2.50		

## Data Availability

Not applicable.

## Supplementary Materials

The following supporting information can be downloaded at: https://www.mdpi.com/article/10.3390/plants11151995/s1, Figure S1: Statistical analysis of RNA-seq data; Table S1: Summary of the transcriptomic dataset; Table S2: List of DEGs in BAT93 in response to inoculation with *C. lindemuthianum* during compatible and incompatible interaction; Table S3: List of primer sequences used in this study.
