# Supplementary material for: Genome-Wide Transcriptomic Analysis of the Effects of Infection with the Hemibiotrophic Fungus Colletotrichum lindemuthianum on Common Bean
Source: Plants (Basel). 2022 Jul 31;11(15):1995. doi: 10.3390/plants11151995 (PMC9370732; doi:10.3390/plants11151995)
Supplement: Supplementary file 1 [file plants-11-01995-s001.zip › plants-1825820-supplementary.pdf]

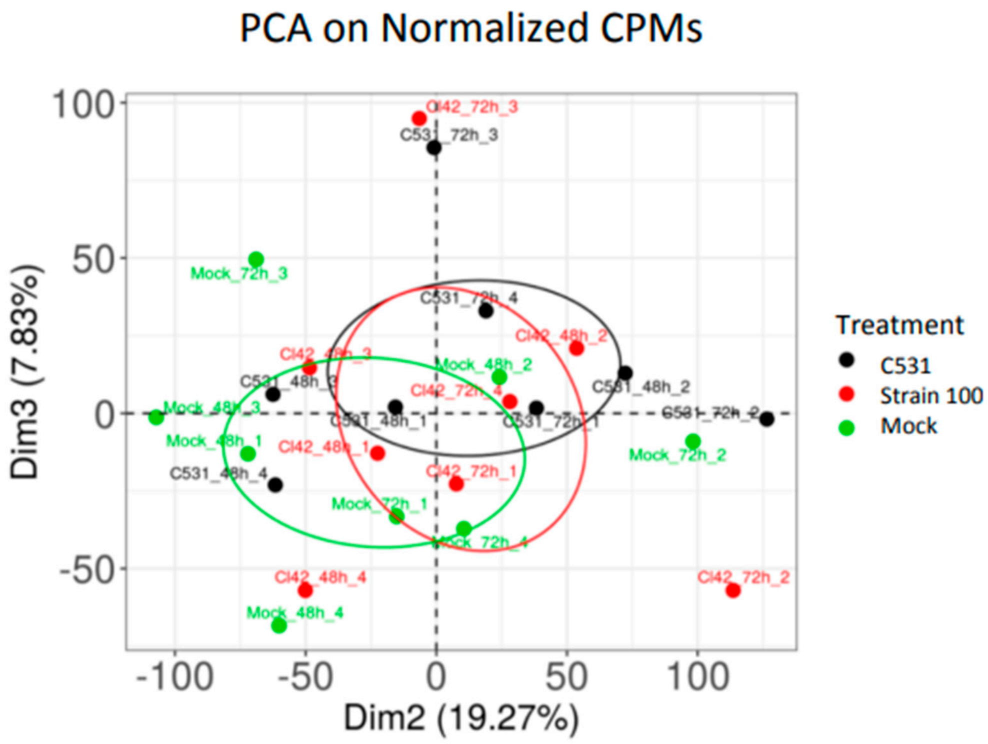

Figure S1. Statistical analysis of RNA-seq data. Principal component analysis (PCA) on normalized RNA-seq reads counts (CPMs) on BAT93 plants, inoculated with one of two strains of *C. lindemuthianum* (C531 or Strain 100) and Mock.

Table S1. Summary of the transcriptomic dataset

Sample name	Replicate	Total cleaned Reads	Uniquely mapped %	Multi mapped %	Unmapped %
C531 48h	1	20000000	92.42	5.46	2.13
	2	11838256	91.33	6.49	2.19
	3	12704009	91.14	7.06	1.81
	4	18730662	85.7	5.5	8.93
C531 72h	1	17620342	93.44	4.41	2.19
	2	15876064	92.6	4.89	2.53
	3	11894538	92.92	5.23	1.87
	4	18912720	88.34	3.03	8.77
Strain 100 48h	1	12473628	91.86	6.15	2.01
	2	13147627	91.49	6.51	2.02
	3	19402849	89.85	7.73	2.45
	4	19469459	87.88	6.31	5.89
Strain 100 72h	1	20000000	93.28	4.82	1.91
	2	12979160	92.83	5.18	2.00
	3	19019870	90.53	6.83	2.67
	4	23268905	87.07	3.04	9.93
Mock 48h	1	15515578	91.04	6.89	2.09
	2	20000000	91.8	6.17	2.05
	3	22219228	89.95	7.78	2.29
	4	19704865	87.06	5.67	7.39
Mock 72h	1	17241729	91.92	5.98	2.12
	2	13133542	92.2	5.05	2.77
	3	19142366	89.83	8.3	1.89
	4	18067151	88.85	3.54	7.72

Table S3. List of primer sequences used in this study.

Gene name	Gene ID	Primer name	Primer Sequence (5'-3')	Product length (bp)	Reference
PR1	Phvul.003G109100	PvPR1-fal F	TGGTCCTAACGGAGGATCAC	98	This study
		PvPR1-fal R	TGGCTTTTCCAGCTTTGAGT		
Bet v I	Phvul.002G209400	Bet_v1_9400 F	TGCCAGACACTGCAGAGAAG	270	This study
		Bet_v1_9400 R	AGGAAACACTGAAAGCCAACT		
Bet v I	Phvul.002G209500	Bet_v1_9500 F	TCCTTGTTTCCTTTGGCCTCA	131	This study
		Bet_v1_9500 R	ACACCTTGCCACATCCCAAA		
PAL	Phvul.001G177800	PvPAL_2 F	TTAAGGCGAGCAGTGAGTGG	117	This study
		PvPAL_2 R	GCAGAGCACCACCTTGTTTG		
ICS	Phvul.010G011700	PvICS F	CTTCACCCTAGTCCAGCTGT	97	This study
		PvICS R	GTCCAGCATACATCCCTCGA		

Reference genes

IDE	Phvul.001G133200	IDE F	GCAACCAACCTTTCATCAGC	156	[93]
		IDE R	AGAAATGCCTCAACCCTTTG		
Actin-11	Phvul.008G011000	Act11-R	TGCATACGTTGGTGATGAGG	190	[93]
		Act11-R	AGCCTTGGGGTTAAGAGGAG		
