# Supplementary material for: Genome-Wide Transcriptomic Analysis of the Effects of Infection with the Hemibiotrophic Fungus Colletotrichum lindemuthianum on Common Bean
Source: Plants (Basel). 2022 Jul 31;11(15):1995. doi: 10.3390/plants11151995 (PMC9370732; doi:10.3390/plants11151995)
Supplement: Supplementary file 1 [file plants-11-01995-s001.zip › Table S2.pdf]

Table S2. List of DEGs in BAT93 in response to inoculation with *C. lindemuthianum* during compatible and incompatible interaction

Gene ID	Annotation	C531 vs Mock 48 hpi		C531 vs Mock 72 hpi		Strain 100 vs Mock 48 hpi		Strain 100 vs Mock 72 hpi	
		Lof2FC	FDR	Lof2FC	FDR	Lof2FC	FDR	Lof2FC	FDR
Phvul.011G096800	KOG0725 - Reductases with broad range of substrate specificities (1 of 51)	5.47	1.30E-02	3.10	2.02E-02	2.86	3.51E-01	2.10	1.43E-01
Phvul.004G125200	PF00069//PF01657 - Protein kinase domain (Pkinase) // Salt stress response/antifungal (Stress-antifung) (1 of 9)	5.14	5.36E-03	2.39	3.86E-02	4.98	1.39E-02	1.31	3.33E-01
Phvul.008G057600	2.1.1.160 - Caffeine synthase / Dimethylxanthine methyltransferase (1 of 1)	5.07	2.40E-05	2.95	1.04E-03	4.41	1.11E-03	1.77	7.16E-02
Phvul.008G008700	no data	4.93	3.73E-03	2.42	1.05E-02	5.41	6.71E-04	3.03	9.47E-04
Phvul.001G169300	PTHR32468:SF10 - CATION/H(+) ANTIPORTER 20 (1 of 1)	4.61	4.95E-05	2.91	4.01E-04	2.01	3.99E-01	2.84	9.12E-04
Phvul.002G209500	PF00407 - Pathogenesis-related protein Bet v I family (Bet v 1)	4.53	2.34E-19	2.80	3.29E-08	3.06	2.94E-08	1.91	3.09E-04
Phvul.011G051600	PTHR10218:SF226 - EXTRA-LARGE GUANINE NUCLEOTIDE-BINDING PROTEIN 2 (1 of 3)	4.39	1.15E-03	2.76	2.00E-02	3.43	3.77E-02	2.69	3.43E-02
Phvul.002G186000	PTHR11999//PTHR11999:SF105 - GROUP II PYRIDOXAL-5-PHOSPHATE DECARBOXYLASE // SUBFAMILY NOT NAMED (1 o	4.39	8.78E-08	4.72	1.10E-08	3.34	2.12E-04	3.80	4.45E-06
Phvul.003G109603	PF00407 - Pathogenesis-related protein Bet v I family (Bet v 1)	4.31	4.94E-04	2.99	3.98E-03	3.51	1.48E-02	2.21	4.48E-02
Phvul.002G185900	PTHR11999//PTHR11999:SF105 - GROUP II PYRIDOXAL-5-PHOSPHATE DECARBOXYLASE // SUBFAMILY NOT NAMED (1 o	4.23	6.68E-10	4.36	2.74E-10	3.46	5.79E-06	3.19	6.12E-06
Phvul.003G109200	PTHR22814//PTHR22814:SF89 - COPPER TRANSPORT PROTEIN ATOX1-RELATED Pathogenesis-related	4.18	8.46E-10	2.94	1.54E-05	2.69	5.41E-04	2.30	1.31E-03
Phvul.002G209400	PF00407 - Pathogenesis-related protein Bet v I family (Bet v 1)	4.15	6.68E-10	2.84	9.35E-06	3.02	1.03E-04	2.14	1.57E-03
Phvul.009G061450	KOG0156 - Cytochrome P450 CYP2 subfamily (1 of 118)	4.10	3.00E-04	2.78	5.20E-03	3.56	3.74E-03	2.36	2.39E-02
Phvul.007G227900	K03531 - cell division protein FtsZ (ftsZ) (1 of 3)	4.06	3.31E-03	2.38	3.12E-02	3.52	2.50E-02	0.85	5.29E-01
Phvul.002G185800	PTHR11999//PTHR11999:SF105 - GROUP II PYRIDOXAL-5-PHOSPHATE DECARBOXYLASE // SUBFAMILY NOT NAMED (1 o	4.04	7.82E-03	3.01	7.57E-03	3.16	9.57E-02	1.77	1.82E-01
Phvul.008G086800	1.11.1.7 - Peroxidase / Lactoperoxidase (1 of 96)	3.97	2.31E-05	3.48	5.51E-06	3.83	1.03E-04	2.79	3.22E-04
Phvul.010G008800	PF00582 - Universal stress protein family (Usp) (1 of 40)	3.82	5.67E-02	3.16	2.43E-02	2.89	2.43E-01	2.77	6.52E-02
Phvul.004G147500	PTHR22849//PTHR22849:SF9 - WDSAM1 PROTEIN // SUBFAMILY NOT NAMED (1 of 2)	3.80	2.55E-06	2.40	3.44E-04	3.26	5.41E-04	2.49	2.56E-04
Phvul.009G108300	3.2.2.9 - Adenosylhomocysteine nucleosidase / S-adenosylhomocysteine/5'-methylthioadenosine nucleosidase (1 of 2)	3.77	2.07E-03	3.83	5.42E-04	3.32	1.12E-02	2.31	4.42E-02
Phvul.010G090000	K18726 - FAS-associated factor 2 (FAF2, UBXD8) (1 of 3)	3.75	6.67E-04	2.13	1.89E-02	3.70	1.22E-03	2.99	1.12E-03
Phvul.010G063900	PF00931//PF01582 - NB-ARC domain (NB-ARC) // TIR domain (TIR) (1 of 28)	3.69	2.96E-09	2.99	3.57E-07	3.48	1.95E-07	2.61	1.02E-05
Phvul.005G071400	no data	3.68	6.68E-10	3.10	1.71E-07	2.62	1.03E-04	2.73	5.58E-06
Phvul.007G273000	PTHR31190:SF15 - ETHYLENE-RESPONSIVE TRANSCRIPTION FACTOR 1B (1 of 3)	3.66	1.42E-02	2.21	1.88E-02	3.00	1.19E-01	2.77	3.24E-03
Phvul.003G074000	PTHR33873:SF1 - TRANSCRIPTION FACTOR VOZ1 (1 of 2)	3.66	1.20E-03	1.71	8.83E-02	2.54	6.48E-02	1.56	1.29E-01
Phvul.002G032866	PTHR10366//PTHR10366:SF375 - NAD DEPENDENT EPIMERASE/DEHYDRATASE // SUBFAMILY NOT NAMED (1 of 9)	3.64	5.07E-04	2.55	4.62E-03	3.32	3.60E-03	2.65	5.44E-03
Phvul.003G109800	PF00407 - Pathogenesis-related protein Bet v I family (Bet v 1)	3.63	2.12E-03	2.84	1.29E-03	1.68	4.46E-01	1.92	6.28E-02
Phvul.005G010000	PTHR10366//PTHR10366:SF352 - NAD DEPENDENT EPIMERASE/DEHYDRATASE // SUBFAMILY NOT NAMED (1 of 5)	3.57	7.46E-03	3.87	7.80E-04	2.87	6.55E-02	3.68	2.58E-03
Phvul.011G182200	PTHR31236:SF2 - DEHYDRATION-RESPONSIVE PROTEIN RD22 (1 of 4)	3.55	6.53E-07	3.02	1.48E-05	2.59	1.13E-03	1.61	2.83E-02
Phvul.009G241900	PF05678 - VQ motif (VQ) (1 of 39)	3.48	3.66E-05	2.78	1.24E-04	3.64	4.51E-05	3.00	3.75E-05
Phvul.007G049700	PTHR32099:SF4 - CYSTEINE-RICH REPEAT SECRETORY PROTEIN 1-RELATED (1 of 5)	3.48	4.70E-02	6.81	1.54E-05	3.41	6.69E-02	5.91	5.64E-04
Phvul.003G109000	PTHR31339:SF15 - PECTIN LYASE-LIKE SUPERFAMILY PROTEIN-RELATED Pathogenesis-related	3.46	7.35E-05	2.52	1.14E-03	2.81	3.85E-03	2.85	3.11E-04
Phvul.006G012501	KOG0531 - Protein phosphatase 1, regulatory subunit, and related proteins (1 of 5)	3.45	5.55E-03	2.49	2.07E-02	2.96	3.23E-02	3.23	4.68E-03
Phvul.003G109100	PTHR22847:SF361 - JOUBERIN (1 of 1) Pathogenesis-related	3.44	6.18E-09	2.62	1.13E-05	2.66	7.18E-05	1.83	3.14E-03
Phvul.007G068800	no data	3.44	1.28E-01	4.25	1.88E-04	2.35	4.22E-01	2.58	4.99E-02
Phvul.005G163100	PF00560//PF07714//PF12819 - Leucine Rich Repeat (LRR 1) // Protein tyrosine kinase (Pkinase Tyr) // Carbohydrate-bir	3.44	4.56E-05	2.68	5.15E-05	3.37	2.63E-04	2.55	1.78E-04
Phvul.009G244000	PTHR24298:SF61 - CYTOCHROME P450 81D1-RELATED (1 of 13)	3.41	2.07E-03	2.48	3.47E-03	1.53	4.78E-01	1.90	4.53E-02
Phvul.010G073900	K02953 - small subunit ribosomal protein S13e (RP-S13e, RPS13) (1 of 2)	3.40	2.98E-02	1.45	2.23E-01	3.01	9.26E-02	1.08	3.87E-01
Phvul.005G010200	PTHR10366//PTHR10366:SF352 - NAD DEPENDENT EPIMERASE/DEHYDRATASE // SUBFAMILY NOT NAMED (1 of 5)	3.40	4.01E-02	3.51	1.14E-02	3.29	6.65E-02	3.27	3.37E-02
Phvul.004G021400	KOG0156 - Cytochrome P450 CYP2 subfamily (1 of 118)	3.36	1.21E-02	1.20	2.87E-01	3.41	1.56E-02	2.21	3.69E-02
Phvul.003G255400	no data	3.31	3.07E-04	3.23	1.10E-04	3.17	9.60E-04	3.38	6.81E-05
Phvul.005G077501	no data	3.31	1.41E-02	1.76	1.50E-01	2.27	1.91E-01	2.15	8.86E-02
Phvul.004G071700	PTHR22883:SF80 - PROTEIN S-ACYLTRANSFERASE 5-RELATED (1 of 3)	3.30	4.42E-05	2.72	2.22E-04	3.10	3.70E-04	2.45	1.71E-03
Phvul.005G121600	no data	3.23	1.26E-03	2.94	8.71E-04	3.17	2.08E-03	2.82	2.25E-03
Phvul.006G058700	PTHR13301//PTHR13301:SF54 - X-BOX TRANSCRIPTION FACTOR-RELATED // SUBFAMILY NOT NAMED (1 of 6)	3.21	9.88E-03	3.36	6.95E-05	3.21	1.58E-02	2.98	8.68E-04
Phvul.008G076600	PTHR10366:SF425 - BRASSINOSTEROID METABOLIC PATHWAY PROTEIN BEN1 (1 of 6)	3.20	2.62E-05	2.74	1.16E-04	2.85	5.41E-04	2.62	3.60E-04
Phvul.009G080000	PF03106 - WRKY DNA -binding domain (WRKY) (1 of 91)	3.20	2.84E-02	3.34	8.05E-04	3.55	1.37E-02	4.04	3.67E-05
Phvul.008G216600	K05933 - aminocyclopropanecarboxylate oxidase (E1.14.17.4) (1 of 9)	3.17	9.47E-07	4.02	1.55E-09	2.57	3.05E-04	3.16	1.38E-06
Phvul.003G051700	PTHR33136:SF3 - PROTEIN RALF-LIKE 32 (1 of 1)	3.13	4.95E-05	2.82	9.09E-05	2.56	3.08E-03	2.74	2.01E-04
Phvul.002G039232	2.3.1.74 - Naringenin-chalcone synthase / Flavonone synthase (1 of 14)	3.11	2.37E-04	2.42	1.58E-03	2.65	4.13E-03	2.62	1.29E-03
Phvul.010G063800	PTHR23155//PTHR23155:SF562 - LEUCINE-RICH REPEAT-CONTAINING PROTEIN // SUBFAMILY NOT NAMED (1 of 7)	3.11	1.71E-05	2.84	3.89E-05	2.81	2.90E-04	2.77	1.03E-04
Phvul.009G244200	1.14.13.89 - Isoflavone 2'-hydroxylase / Isoflavone 2'-monooxygenase (1 of 12)	3.10	9.08E-03	1.23	1.57E-01	2.67	5.06E-02	0.45	6.76E-01
Phvul.011G144200	no data	3.09	5.15E-05	2.18	2.53E-03	2.03	2.45E-02	1.41	6.23E-02
Phvul.002G285800	K10684 - ubiquitin-like 1-activating enzyme E1 A (UBLE1A, SAE1) (1 of 1)	3.09	9.15E-03	2.45	1.43E-02	2.28	1.20E-01	1.66	1.18E-01
Phvul.002G215100	PF07714//PF08263//PF13855 - Protein tyrosine kinase (Pkinase Tyr) // Leucine rich repeat N-terminal domain (LRRNT 2	3.09	2.43E-03	2.63	1.50E-03	2.82	1.55E-02	3.01	1.66E-04
Phvul.004G021700	KOG0156 - Cytochrome P450 CYP2 subfamily (1 of 118)	3.05	2.27E-03	2.40	5.88E-03	2.47	2.98E-02	1.94	3.37E-02
Phvul.005G009500	KOG0254 - Predicted transporter (major facilitator superfamily) (1 of 73)	3.05	1.68E-02	2.08	2.72E-02	2.25	1.62E-01	1.81	7.07E-02
Phvul.009G211000	PF02095 - Extensin-like protein repeat (Extensin 1) (1 of 3)	3.05	6.68E-10	2.22	1.16E-05	2.02	4.36E-04	1.61	2.80E-03
Phvul.002G038600	2.3.1.74 - Naringenin-chalcone synthase / Flavonone synthase (1 of 14)	3.04	8.00E-04	1.96	1.77E-02	2.58	9.49E-03	2.07	1.61E-02
Phvul.010G021001	PTHR22835:SF262 - GDSL ESTERASE/LIPASE 5-RELATED (1 of 3)	3.02	1.11E-03	2.95	2.62E-04	2.46	1.85E-02	1.96	2.38E-02
Phvul.009G258600	K13066 - caffeic acid 3-O-methyltransferase (E2.1.1.68, COMT) (1 of 18)	3.01	6.74E-07	2.96	3.11E-07	2.49	2.63E-04	2.54	1.47E-05
Phvul.010G035500	no data	3.00	2.20E-02	2.03	1.25E-02	3.31	8.63E-03	1.44	1.18E-01
Phvul.008G287300	PTHR11732//PTHR11732:SF220 - ALDO/KETO REDUCTASE // SUBFAMILY NOT NAMED (1 of 3)	2.98	1.30E-02	3.57	3.89E-05	2.67	5.59E-02	3.62	4.07E-05
Phvul.006G030200	PF14009 - Domain of unknown function (DUF4228) (DUF4228) (1 of 38)	2.97	1.28E-02	2.74	6.91E-03	2.77	3.45E-02	2.60	1.62E-02
Phvul.002G038700	2.3.1.74 - Naringenin-chalcone synthase / Flavonone synthase (1 of 14)	2.96	1.72E-03	2.33	5.06E-03	2.20	4.68E-02	2.33	7.55E-03
Phvul.002G184300	2.3.1.74 - Naringenin-chalcone synthase / Flavonone synthase (1 of 14)	2.93	2.07E-03	2.41	4.52E-03	2.59	1.12E-02	2.41	6.71E-03
Phvul.002G199800	1.3.3.8 - Tetrahydroberberine oxidase / THB oxidase (1 of 37)	2.93	9.73E-03	3.91	9.35E-06	2.60	4.87E-02	3.01	2.63E-03
Phvul.009G116500	PTHR22595:SF34 - BASIC ENDOCHITINASE B (1 of 8)	2.90	1.49E-07	2.10	2.85E-05	2.25	3.96E-04	1.40	1.06E-02
Phvul.001G155400	PTHR23050//PTHR23050:SF211 - CALCIUM BINDING PROTEIN // SUBFAMILY NOT NAMED (1 of 2)	2.90	4.08E-06	1.97	9.99E-04	2.83	3.07E-05	2.05	9.20E-04
Phvul.007G092400	no data	2.89	3.38E-02	0.88	4.42E-01	2.66	8.25E-02	0.33	8.06E-01
Phvul.005G116551	PTHR13301:SF53 - CELLULOSE SYNTHASE-LIKE PROTEIN B1-RELATED (1 of 10)	2.89	1.47E-02	2.97	7.33E-04	2.41	9.01E-02	2.35	1.25E-02
Phvul.003G064300	2.4.2.18 - Anthranilate phosphoribosyltransferase / Phosphoribosyl-anthranilate pyrophosphorylase (1 of 2)	2.87	8.28E-05	2.02	2.04E-03	2.32	4.07E-03	1.69	1.52E-02
Phvul.006G078400	PTHR11945:SF153 - AGAMOUS-LIKE MADS-BOX PROTEIN AGL36-RELATED (1 of 2)	2.85	1.30E-01	3.45	3.53E-05	1.77	5.09E-01	3.19	2.62E-04
Phvul.005G079600	K18178 - cytochrome c oxidase assembly factor 5 (COA5, PET191) (1 of 1)	2.83	1.35E-03	2.51	1.78E-03	2.62	5.41E-03	2.52	3.04E-03
Phvul.011G144100	no data	2.83	2.63E-02	1.69	6.35E-02	1.56	3.90E-01	0.82	4.30E-01
Phvul.004G025900	no data	2.83	2.47E-02	1.45	1.55E-01	3.36	3.74E-03	2.57	4.18E-03
Phvul.002G009700	PF05678 - VQ motif (VQ) (1 of 39)	2.82	4.42E-05	2.33	1.72E-04	2.22	3.99E-03	1.69	1.04E-02
Phvul.002G039000	2.3.1.74 - Naringenin-chalcone synthase / Flavonone synthase (1 of 14)	2.81	3.38E-03	2.12	1.24E-02	2.31	3.11E-02	2.28	1.30E-02
Phvul.002G154600	PTHR11206:SF127 - MATE EFFLUX FAMILY PROTEIN (1 of 2)	2.79	9.79E-04	1.75	2.20E-02	1.95	8.23E-02	1.76	2.67E-02
Phvul.005G024800	PF05678 - VQ motif (VQ) (1 of 39)	2.78	3.50E-03	3.31	2.78E-05	2.96	1.76E-03	3.65	3.43E-06
Phvul.002G215000	PF00139//PF07714 - Legume lectin domain (Lectin legB) // Protein tyrosine kinase (Pkinase Tyr) (1 of 5)	2.78	3.65E-03	2.52	2.22E-03	2.76	3.93E-03	3.11	2.17E-04
Phvul.002G039300	2.3.1.74 - Naringenin-chalcone synthase / Flavonone synthase (1 of 14)	2.78	1.00E-02	2.24	1.22E-02	2.88	9.92E-03	2.11	2.86E-02
Phvul.004G098000	no data	2.77	5.52E-04	1.87	1.12E-02	1.80	6.89E-02	0.65	4.60E-01
Phvul.002G317000	PF05678 - VQ motif (VQ) (1 of 39)	2.76	2.20E-03	2.71	8.94E-04	2.49	1.10E-02	2.43	4.49E-03
Phvul.004G021800	KOG0156 - Cytochrome P450 CYP2 subfamily (1 of 118)	2.75	6.19E-03	2.37	3.49E-03	2.98	3.74E-03	1.98	2.08E-02
Phvul.010G066500	PTHR23026 - NADPH NITROREDUCTASE (1 of 1)	2.74	1.41E-08	2.81	4.71E-09	2.16	1.00E-04	1.81	2.07E-04
Phvul.003G192000	PF03106 - WRKY DNA -binding domain (WRKY) (1 of 91)	2.73	1.85E-02	1.78	1.03E-02	2.71	3.16E-02	1.74	1.69E-02
Phvul.007G260400	PTHR27007:SF56 - L-TYPE LECTIN-DOMAIN CONTAINING RECEPTOR KINASE S.5-RELATED (1 of 3)	2.72	1.24E-02	2.17	5.82E-03	2.19	9.19E-02	2.27	5.72E-03
Phvul.007G048900	PTHR27002:SF122 - CYSTEINE-RICH RECEPTOR-LIKE PROTEIN KINASE 28-RELATED (1 of 17)	2.72	5.96E-03	2.60	6.04E-03	1.84	1.47E-01	3.12	1.13E-03
Phvul.003G239800	no data	2.71	1.76E-03	2.80	2.87E-04	2.28	1.88E-02	3.25	3.02E-05
Phvul.009G032900	no data	2.71	1.15E-03	2.60	4.12E-04	2.55	3.60E-03	2.98	6.96E-05
Phvul.002G046100	PF00069//PF00954//PF01453 - Protein kinase domain (Pkinase) // S-locus glycoprotein domain (S locus glycop) // D-ma	2.70	1.04E-01	2.18	6.92E-02	1.99	3.31E-01	2.71	2.50E-02
Phvul.006G079700	PTHR24298:SF1 - CYTOCHROME P450, FAMILY 79, SUBFAMILY C, POLYPEPTIDE 1-RELATED (1 of 6)	2.70	1.85E-02	2.56	7.14E-03	2.13	1.17E-01	2.58	9.50E-03
Phvul.008G015800	PTHR11732//PTHR11732:SF220 - ALDO/KETO REDUCTASE // SUBFAMILY NOT NAMED (1 of 3)	2.70	3.07E-04	2.42	1.67E-04	2.56	1.22E-03	1.77	1.03E-02
Phvul.005G053100	PTHR11260:SF204 - GLUTATHIONE S-TRANSFERASE U21-RELATED (1 of 4)	2.65	5.52E-04	2.37	5.15E-04	2.59	1.34E-03	2.60	1.78E-04
Phvul.009G231600	PTHR12565//PTHR12565:SF180 - STEROL REGULATORY ELEMENT-BINDING PROTEIN // SUBFAMILY NOT NAMED (1 of 2)	2.65	2.99E-04	2.66	1.02E-04	2.42	1.91E-03	3.11	9.31E-06
Phvul.005G011100	K14802 - phospholipid-transporting ATPase [EC:3.6.3.1] (DRS2, ATP8A) (1 of 5)	2.64	5.25E-03	2.02	1.21E-02	2.39	2.05E-02	1.42	1.00E-01
Phvul.010G089900	K12175 - COP9 signalosome complex subunit 1 (GPS1, COPS1, CSN1) (1 of 1)	2.64	3.34E-02	2.57	9.52E-03	2.72	3.23E-02	2.54	1.37E-02
Phvul.005G004400	1.3.3.8 - Tetrahydroberberine oxidase / THB oxidase (1 of 37)	2.64	8.60E-04	2.33	1.00E-03	2.45	3.70E-03	2.30	1.81E-03
Phvul.007G049200	PF01657//PF14244 - Salt stress response/antifungal (Stress-antifung) // gag-polypeptide of LTR copia-type (UBN2 3) (1	2.63	3.34E-02	3.05	3.53E-03	2.61	4.76E-02	3.79	3.87E-04
Phvul.008G112200	no data	2.63	1.66E-03	3.11	4.36E-05	2.28	1.48E-02	3.02	1.30E-04

Phvul.001G183300	PTHR10209:SF152 - 1-AMINOCYCLOPROPANE-1-CARBOXYLATE OXIDASE-RELATED (1 of 2)	2.62	3.26E-02	1.25	2.79E-01	2.12	1.42E-01	1.29	2.71E-01
Phvul.005G057200	K03242 - translation initiation factor 2 subunit 3 (EIF2S3) (1 of 3)	2.62	8.32E-03	3.14	5.70E-04	3.07	6.71E-04	2.71	6.69E-03
Phvul.001G160100	PTHR31190:SF26 - ETHYLENE-RESPONSIVE TRANSCRIPTION FACTOR ERF096 (1 of 6)	2.62	1.16E-03	1.51	2.97E-02	1.94	5.00E-02	0.23	8.06E-01
Phvul.002G260800	PF04791 - LMBR1-like membrane protein (LMBR1) (1 of 4)	2.61	2.07E-03	1.88	5.60E-03	2.44	8.14E-03	2.40	4.37E-04
Phvul.005G164100	PTHR27003:SF105 - LEUCINE-RICH REPEAT PROTEIN KINASE-RELATED (1 of 25)	2.60	1.00E-02	2.15	1.94E-03	2.42	2.98E-02	1.92	8.59E-03
Phvul.006G194600	PTHR27002:SF93 - RECEPTOR-LIKE SERINE/THREONINE-PROTEIN KINASE SD1-6-RELATED (1 of 2)	2.56	1.39E-02	2.38	7.64E-03	2.45	3.23E-02	2.62	4.84E-03
Phvul.001G145900	PTHR21495:SF71 - DIRIGENT PROTEIN 1-RELATED (1 of 10)	2.55	4.67E-04	2.15	8.56E-04	1.99	2.62E-02	1.66	1.74E-02
Phvul.007G008600	K01859 - chalcone isomerase (E5.5.1.6) (1 of 4)	2.55	1.47E-06	2.61	5.99E-07	2.22	1.46E-04	2.31	1.28E-05
Phvul.001G158300	no data	2.55	7.11E-02	3.10	3.33E-03	3.06	2.17E-02	2.91	1.01E-02
Phvul.009G075100	PTHR11825:SF54 - BRANCHED-CHAIN-AMINO-ACID AMINOTRANSFERASE 1, MITOCHONDRIAL (1 of 1)	2.55	8.77E-03	2.18	1.23E-02	1.94	9.16E-02	1.91	3.45E-02
Phvul.009G116600	PTHR22595:SF34 - BASIC ENDOCHITINASE B (1 of 8)	2.53	9.47E-07	2.23	1.19E-05	1.96	6.53E-04	1.55	4.06E-03
Phvul.007G092500	K03231 - elongation factor 1-alpha (EEF1A) (1 of 3)	2.53	1.98E-02	1.91	2.32E-02	1.68	2.77E-01	1.53	9.29E-02
Phvul.005G119200	PTHR22849:SF0 - WD REPEAT, SAM AND U-BOX DOMAIN-CONTAINING PROTEIN 1 (1 of 2)	2.53	4.40E-04	1.98	2.69E-03	2.25	3.93E-03	1.44	4.08E-02
Phvul.008G232700	6.3.2.17 - Tetrahydrofolate synthase / Tetrahydrofolylpolyglutamate synthase (1 of 2)	2.52	9.96E-03	3.66	7.37E-06	2.48	1.67E-02	3.52	2.04E-05
Phvul.007G260500	PTHR27007:SF56 - L-TYPE LECTIN-DOMAIN CONTAINING RECEPTOR KINASE S.5-RELATED (1 of 3)	2.51	3.49E-02	2.11	1.14E-02	1.70	2.82E-01	1.60	7.60E-02
Phvul.004G115600	PF00560//PF13855 - Leucine Rich Repeat (LRR 1) // Leucine rich repeat (LRR 8) (1 of 35)	2.50	1.41E-02	2.68	1.18E-03	1.87	1.28E-01	2.00	2.37E-02
Phvul.010G063600	PTHR21493:SF117 - ALPHA/BETA-HYDROLASES SUPERFAMILY PROTEIN (1 of 3)	2.50	6.67E-04	2.91	1.60E-05	2.04	1.48E-02	2.44	5.03E-04
Phvul.002G039100	2.3.1.74 - Naringenin-chalcone synthase / Flavonone synthase (1 of 14)	2.49	1.16E-02	1.66	4.74E-02	2.02	7.56E-02	1.98	2.30E-02
Phvul.011G060300	PTHR14155:SF75 - E3 UBIQUITIN LIGASE BIG BROTHER (1 of 3)	2.48	6.54E-02	4.18	4.33E-05	2.77	4.21E-02	3.48	9.10E-04
Phvul.002G039166	2.3.1.74 - Naringenin-chalcone synthase / Flavonone synthase (1 of 14)	2.48	9.55E-03	2.44	2.92E-03	2.51	1.19E-02	2.54	3.96E-03
Phvul.008G139900	no data	2.46	1.04E-03	2.96	1.54E-05	1.77	4.74E-02	2.30	1.12E-03
Phvul.004G103900	no data	2.46	4.99E-04	2.22	8.40E-04	1.41	1.26E-01	0.85	2.56E-01
Phvul.002G057500	PTHR19139:SF134 - AQUAPORIN TIP2-3-RELATED (1 of 1)	2.46	8.28E-03	2.69	7.24E-04	2.44	1.17E-02	1.84	2.61E-02
Phvul.007G252500	PTHR27009:SF21 - PR5-LIKE RECEPTOR KINASE-RELATED (1 of 19)	2.45	1.98E-02	1.70	3.41E-02	1.87	1.64E-01	1.33	1.27E-01
Phvul.009G103800	K14488 - SAUR family protein (SAUR) (1 of 75)	2.45	4.37E-02	0.26	8.47E-01	2.01	1.59E-01	0.16	9.15E-01
Phvul.008G287200	PTHR11732//PTHR11732:SF220 - ALDO/KETO REDUCTASE // SUBFAMILY NOT NAMED (1 of 3)	2.45	6.79E-02	2.65	2.51E-03	2.82	3.23E-02	2.71	3.17E-03
Phvul.005G173600	PTHR19241:SF219 - ABC TRANSPORTER G FAMILY MEMBER 38 (1 of 18)	2.45	3.29E-03	2.14	3.92E-03	2.21	1.60E-02	2.23	4.36E-03
Phvul.009G045601	no data	2.44	3.07E-04	2.39	2.84E-04	2.53	2.79E-04	3.00	3.43E-06
Phvul.001G088200	KOG2306 - Uncharacterized conserved protein (1 of 2)	2.43	3.08E-03	1.97	7.86E-03	1.90	4.53E-02	1.79	2.02E-02
Phvul.009G256400	3.2.1.39 - Glucan endo-1,3-beta-D-glucosidase / Laminarinase (1 of 65)	2.43	1.59E-04	1.85	2.30E-03	1.99	5.68E-03	1.17	7.22E-02
Phvul.008G044900	PTHR23070:SF44 - AAA-TYPE ATPASE FAMILY PROTEIN-RELATED (1 of 3)	2.43	3.05E-02	2.90	1.84E-03	2.52	3.04E-02	3.75	8.86E-05
Phvul.005G162000	PTHR27003:SF44 - LRR RECEPTOR-LIKE SERINE/THREONINE-PROTEIN KINASE MEE39-RELATED (1 of 2)	2.43	1.72E-02	3.05	2.70E-04	2.00	9.57E-02	2.46	5.77E-03
Phvul.L001846	1.14.13.21 - Flavonoid 3'-monooxygenase / Flavonoid 3'-hydroxylase (1 of 8)	2.43	9.88E-03	1.47	7.33E-02	2.16	3.32E-02	1.30	1.23E-01
Phvul.011G168400	PTHR27002:SF129 - INACTIVE G-TYPE LECTIN S-RECEPTOR-LIKE SERINE/THREONINE-PROTEIN KINASE SRK-RELATED (1 of	2.42	1.24E-01	2.69	2.10E-02	2.61	1.07E-01	2.79	1.91E-02
Phvul.006G176600	PTHR23315:SF131 - ARMADILLO/BETA-CATENIN-LIKE REPEAT-CONTAINING PROTEIN (1 of 2)	2.42	3.22E-03	1.65	2.24E-02	2.33	8.46E-03	1.61	3.60E-02
Phvul.011G144000	K00565 - mRNA (guanine-N7-)-methyltransferase (RNMT) (1 of 2)	2.42	2.12E-03	1.37	4.81E-02	1.53	1.42E-01	0.17	8.59E-01
Phvul.006G188500	1.10.3.1 - Catechol oxidase / Tyrosinase (1 of 3)	2.41	1.69E-02	1.74	4.50E-02	2.19	6.26E-02	2.42	5.78E-03
Phvul.009G211100	no data	2.40	2.55E-02	2.49	2.32E-02	1.99	1.26E-01	2.11	7.87E-02
Phvul.002G004000	PTHR32246:SF20 - BON1-ASSOCIATED PROTEIN 1-RELATED (1 of 9)	2.40	1.17E-02	2.50	1.71E-03	2.70	3.74E-03	3.07	1.30E-04
Phvul.009G146700	no data	2.40	2.53E-03	2.90	2.10E-06	2.51	1.84E-03	2.51	1.03E-04
Phvul.002G318100	K12396 - AP-3 complex subunit delta-1 (AP3D1) (1 of 1)	2.38	1.83E-04	2.71	6.05E-06	2.35	3.76E-04	3.20	2.02E-07
Phvul.008G037300	K02183 - calmodulin (CALM) (1 of 9)	2.38	2.62E-05	2.47	4.61E-06	2.50	3.07E-05	2.85	2.02E-07
Phvul.010G003400	no data	2.38	3.45E-02	2.53	3.49E-03	1.97	1.42E-01	2.50	5.00E-03
Phvul.011G077900	PTHR10252:SF44 - DNA POLYMERASE EPSILON SUBUNIT C-RELATED (1 of 1)	2.37	3.82E-03	2.28	1.73E-03	2.04	3.11E-02	1.83	1.59E-02
Phvul.003G045600	PTHR14304 - P30 DBC PROTEIN (1 of 1)	2.37	1.85E-02	2.11	1.40E-02	1.58	2.19E-01	2.30	1.03E-02
Phvul.009G143800	K15104 - solute carrier family 25 (mitochondrial oxoglutarate transporter), member 11 (SLC25A11, OGC) (1 of 3)	2.37	1.26E-03	1.92	9.27E-03	2.31	2.73E-03	1.91	1.30E-02
Phvul.009G240688	no data	2.37	1.79E-02	1.53	7.28E-02	1.98	8.72E-02	2.10	1.12E-02
Phvul.005G030800	1.6.5.2//1.6.5.3 - NAD(P)H dehydrogenase (quinone) / Vitamin-K reductase // NADH:ubiquinone reductase (H(+)-translo	2.36	2.72E-03	2.03	4.30E-03	2.05	2.25E-02	1.59	3.63E-02
Phvul.004G112400	1.1.1.330 - Very-long-chain 3-oxoacyl-CoA reductase / Very-long-chain beta-ketoacyl-CoA reductase (1 of 18)	2.36	3.42E-06	2.43	4.38E-07	1.89	8.72E-04	1.74	4.27E-04
Phvul.007G267800	PTHR22849//PTHR22849:SF24 - WDSAM1 PROTEIN // SUBFAMILY NOT NAMED (1 of 1)	2.35	4.90E-02	3.09	4.18E-03	2.70	1.98E-02	2.64	2.30E-02
Phvul.007G267200	PTHR19957:SF69 - SYNTAXIN-121-RELATED (1 of 4)	2.35	7.56E-04	2.73	2.67E-05	2.25	2.37E-03	3.10	2.77E-06
Phvul.006G172000	no data	2.35	2.15E-01	3.06	5.41E-03	3.58	2.16E-02	3.64	9.20E-04
Phvul.010G021700	PTHR31851:SF16 - MEMBRANE PROTEIN OF ER BODY 2 (1 of 4)	2.34	1.18E-01	3.20	9.72E-03	2.20	2.19E-01	2.18	1.13E-01
Phvul.011G152700	PF00954//PF01453//PF07714//PF08276 - S-locus glycoprotein domain (S locus glycop) // D-mannose binding lectin (B le	2.33	1.03E-01	2.56	1.05E-02	1.98	2.45E-01	1.70	1.10E-01
Phvul.010G134000	PF13962 - Domain of unknown function (PGG) (1 of 69)	2.33	1.05E-01	2.57	8.06E-03	1.29	5.27E-01	2.61	9.38E-03
Phvul.008G044701	2.7.11.1 - Non-specific serine/threonine protein kinase / Threonine-specific protein kinase (1 of 1198)	2.32	1.57E-02	2.64	9.53E-04	2.21	3.45E-02	2.45	3.96E-03
Phvul.006G084900	PF05757 - Oxygen evolving enhancer protein 3 (PsbQ) (PsbQ) (1 of 4)	2.31	8.77E-03	3.12	2.35E-05	2.14	2.72E-02	3.22	1.76E-05
Phvul.001G040700	no data	2.31	1.00E-02	3.35	9.56E-06	1.70	1.45E-01	2.61	1.56E-03
Phvul.002G165800	PTHR23070//PTHR23070:SF12 - BCS1 AAA-TYPE ATPASE // SUBFAMILY NOT NAMED (1 of 3)	2.31	4.80E-02	0.70	5.56E-01	2.75	1.73E-02	1.00	3.54E-01
Phvul.007G050400	PF01657//PF07714 - Salt stress response/antifungal (Stress-antifung) // Protein tyrosine kinase (Pkinase Tyr) (1 of 40)	2.30	9.49E-04	2.11	9.20E-04	1.69	3.80E-02	1.72	1.08E-02
Phvul.002G014700	1.14.13.89 - Isoflavone 2'-hydroxylase / Isoflavone 2'-monooxygenase (1 of 12)	2.30	2.83E-03	2.10	2.02E-03	1.96	2.68E-02	2.02	4.71E-03
Phvul.001G039700	no data	2.30	1.83E-04	1.40	1.72E-02	2.15	1.28E-03	1.68	4.73E-03
Phvul.009G240666	PF12796//PF13962 - Ankyrin repeats (3 copies) (Ank 2) // Domain of unknown function (PGG) (1 of 27)	2.29	5.96E-03	2.79	7.60E-05	1.78	7.07E-02	2.27	2.39E-03
Phvul.002G326600	PTHR18934:SF112 - DEA(D/H)-BOX RNA HELICASE FAMILY PROTEIN (1 of 1)	2.29	5.13E-04	2.25	2.29E-04	2.31	8.01E-04	2.44	8.86E-05
Phvul.001G071100	K14787 - multiple RNA-binding domain-containing protein 1 (MRD1, RBM19) (1 of 1)	2.29	1.07E-03	2.79	6.05E-06	2.19	3.33E-03	2.86	3.61E-06
Phvul.010G045000	no data	2.29	2.14E-02	2.52	1.27E-03	1.63	2.02E-01	2.13	1.15E-02
Phvul.008G123900	PTHR31140:SF1 - B3 DOMAIN-CONTAINING TRANSCRIPTION FACTOR ABI3 (1 of 1)	2.27	1.08E-02	2.37	9.07E-04	2.19	2.22E-02	2.72	1.46E-04
Phvul.002G046500	PF00069//PF00954//PF01453 - Protein kinase domain (Pkinase) // S-locus glycoprotein domain (S locus glycop) // D-ma	2.27	1.02E-02	3.05	3.15E-05	2.45	6.11E-03	3.30	8.17E-06
Phvul.001G019300	PTHR33095:SF7 - AR781 (1 of 3)	2.26	1.65E-05	2.26	4.61E-06	2.06	2.63E-04	2.37	1.38E-06
Phvul.002G038800	2.3.1.74 - Naringenin-chalcone synthase / Flavonone synthase (1 of 14)	2.26	1.43E-02	2.27	3.97E-03	2.29	1.71E-02	2.21	8.61E-03
Phvul.011G096300	PF12937 - F-box-like (F-box-like) (1 of 53)	2.25	4.36E-04	2.00	7.83E-04	1.92	6.12E-03	2.33	1.12E-04
Phvul.004G049200	K01180 - endo-1,3(4)-beta-glucanase (E3.2.1.6) (1 of 6)	2.25	1.08E-02	2.58	6.59E-04	2.04	3.66E-02	2.35	2.79E-03
Phvul.006G074600	PTHR11709:SF68 - LACCASE-13-RELATED (1 of 2)	2.24	1.55E-03	2.13	4.14E-04	1.86	2.24E-02	1.88	2.91E-03
Phvul.001G145600	PTHR21495:SF71 - DIRIGENT PROTEIN 1-RELATED (1 of 10)	2.24	9.91E-02	2.41	1.99E-02	0.98	6.43E-01	2.95	6.15E-03
Phvul.007G091000	2.1.1.154 - Isoliquiritigenin 2'-O-methyltransferase / CHMT (1 of 8)	2.23	2.57E-02	0.29	7.90E-01	1.54	2.47E-01	-0.52	6.20E-01
Phvul.002G155500	PF00314 - Thaumatin family (Thaumatin) Pathogenesis-related	2.23	9.47E-07	1.89	2.78E-05	1.84	2.63E-04	1.55	1.02E-03
Phvul.002G211500	PTHR10209//PTHR10209:SF167 - OXIDOREDUCTASE, 2OG-FE II OXYGENASE FAMILY PROTEIN // SUBFAMILY NOT NAME	2.23	2.73E-02	2.36	5.02E-03	1.42	2.77E-01	1.65	6.08E-02
Phvul.006G196900	K13449 - pathogenesis-related protein 1 (PR1)	2.21	2.31E-05	1.28	1.19E-02	1.88	1.00E-03	0.99	6.97E-02
Phvul.008G044600	PF00560//PF07714//PF13855 - Leucine Rich Repeat (LRR 1) // Protein tyrosine kinase (Pkinase Tyr) // Leucine rich repe	2.21	8.77E-03	2.13	4.04E-03	1.70	9.20E-02	2.25	4.02E-03
Phvul.004G162500	PTHR33374:SF6 - ARABINOGALACTAN PEPTIDE 16-RELATED (1 of 3)	2.20	5.97E-04	2.35	8.15E-05	2.02	3.45E-03	2.39	8.33E-05
Phvul.004G088400	PTHR31951:SF22 - ECA1 GAMETOGENESIS RELATED FAMILY PROTEIN-RELATED (1 of 4)	2.19	1.17E-01	2.85	4.93E-03	1.63	3.55E-01	2.63	1.27E-02
Phvul.001G226000	PF07887 - Calmodulin binding protein-like (Calmodulin bind) (1 of 10)	2.19	3.42E-03	2.55	1.13E-04	2.06	8.94E-03	2.70	6.81E-05
Phvul.005G166500	PTHR19241:SF246 - ABC TRANSPORTER G FAMILY MEMBER 29 (1 of 2)	2.19	1.50E-04	2.35	8.57E-06	1.77	7.02E-03	1.64	3.16E-03
Phvul.005G102500	PF03007//PF06974 - Wax ester synthase-like Acyl-CoA acyltransferase domain (WES acyltransf) // Protein of unknown f	2.18	9.96E-03	2.54	6.31E-04	1.28	2.52E-01	2.01	1.03E-02
Phvul.001G258900	PF06522 - NADH-ubiquinone reductase complex 1 MLRQ subunit (B12D) (1 of 3)	2.18	1.45E-02	2.57	8.43E-04	1.80	7.93E-02	2.50	2.29E-03
Phvul.007G138200	2.7.10.2//2.7.11.1 - Non-specific protein-tyrosine kinase / Cytoplasmic protein tyrosine kinase // Non-specific serine/thr	2.18	6.07E-06	1.56	8.06E-04	1.91	2.79E-04	1.37	4.76E-03
Phvul.006G086200	no data	2.17	4.94E-03	2.46	1.39E-04	2.41	1.73E-03	2.93	5.58E-06
Phvul.011G048800	PTHR30546//PTHR30546:SF8 - FLAVODOXIN-RELATED PROTEIN WRBA-RELATED // SUBFAMILY NOT NAMED (1 of 1)	2.17	1.16E-03	2.46	4.42E-05	2.40	3.54E-04	2.68	1.20E-05
Phvul.008G020900	PTHR23155//PTHR23155:SF497 - LEUCINE-RICH REPEAT-CONTAINING PROTEIN // SUBFAMILY NOT NAMED (1 of 6)	2.17	6.08E-02	1.74	3.75E-02	1.89	1.59E-01	1.40	1.15E-01
Phvul.002G077100	PTHR23213:SF239 - FORMIN-LIKE PROTEIN 4-RELATED (1 of 3)	2.16	1.08E-02	1.45	3.04E-02	1.85	5.77E-02	1.00	1.79E-01
Phvul.003G072900	PTHR11668//PTHR11668:SF257 - SERINE/THREONINE PROTEIN PHOSPHATASE // SUBFAMILY NOT NAMED (1 of 2)	2.16	2.63E-03	2.02	9.53E-04	1.96	1.30E-02	2.07	1.02E-03
Phvul.001G048500	K04733 - interleukin-1 receptor-associated kinase 4 (IRAK4) (1 of 48)	2.15	1.55E-02	1.95	1.79E-03	1.84	7.29E-02	1.05	1.34E-01
Phvul.006G133200	K08497 - protein transport protein SEC20 (SEC20) (1 of 2)	2.14	2.28E-01	2.24	3.90E-02	1.60	4.84E-01	1.72	1.37E-01
Phvul.001G048800	PF13947 - Wall-associated receptor kinase galacturonan-binding (GUB WAK bind) (1 of 65)	2.14	4.58E-02	2.18	1.17E-03	1.87	1.37E-01	1.34	7.08E-02
Phvul.007G128100	PF07279 - Protein of unknown function (DUF1442) (DUF1442) (1 of 8)	2.13	5.96E-03	1.35	4.24E-02	1.71	6.31E-02	0.94	1.99E-01
Phvul.005G169200	PTHR15907:SF46 - PROTEIN PLANT CADMIUM RESISTANCE 8 (1 of 1)	2.13	1.42E-02	1.61	3.49E-02	1.57	1.51E-01	1.83	2.07E-02
Phvul.005G162500	PF00560//PF07714//PF12819 - Leucine Rich Repeat (LRR 1) // Protein tyrosine kinase (Pkinase Tyr) // Carbohydrate-bir	2.13	4.94E-04	2.16	2.32E-06	1.94	4.87E-03	1.93	3.69E-05
Phvul.003G124000	PF03106 - WRKY DNA -binding domain (WRKY) (1 of 91)	2.13	2.53E-04	1.80	1.43E-03	1.87	3.70E-03	1.77	2.79E-03
Phvul.002G134600	K13806 - sn1-specific diacylglycerol lipase (DAGL) (1 of 1)	2.12	5.99E-07	2.12	3.11E-07	2.09	5.71E-06	1.71	4.87E-05
Phvul.008G236500	PTHR10641//PTHR10641:SF503 - MYB-LIKE DNA-BINDING PROTEIN MYB // SUBFAMILY NOT NAMED (1 of 1)	2.12	5.74E-02	2.08	1.36E-02	1.35	3.52E-01	1.59	7.50E-02
Phvul.002G170200	PF02365 - No apical meristem (NAM) protein (NAM) (1 of 91)	2.12	1.43E-02	0.66	4.33E-01	1.13	3.55E-01	0.19	8.50E-01
Phvul.008G049700	PTHR24177:SF14 - ANKYRIN REPEAT-CONTAINING PROTEIN (1 of 1)	2.12	4.87E-02	1.79	4.67E-02	2.01	8.45E-02	1.69	6.68E-02
Phvul.003G187200	PF00069//PF00560//PF08263//PF13855 - Protein kinase domain (Pkinase) // Leucine Rich Repeat (LRR 1) // Leucine ric	2.12	7.49E-03	1.99	2.30E-03	2.14	9.73E-03	2.09	2.40E-03

Phvul.008G045200	PF00069//PF00560//PF13855 - Protein kinase domain (Pkinase) // Leucine Rich Repeat (LRR 1) // Leucine rich repeat (L	2.11	9.61E-04	2.81	2.37E-06	1.79	1.13E-02	3.14	2.24E-07
Phvul.004G114700	PF00560//PF08263 - Leucine Rich Repeat (LRR 1) // Leucine rich repeat N-terminal domain (LRRNT 2) (1 of 17)	2.11	8.90E-02	3.21	5.60E-04	1.54	3.24E-01	2.82	4.14E-03
Phvul.003G070900	3.5.4.6 - AMP deaminase / Myoadenylate deaminase (1 of 2)	2.10	2.69E-02	1.84	2.14E-02	1.86	9.42E-02	1.99	1.44E-02
Phvul.003G177700	no data	2.10	3.97E-04	1.91	8.79E-04	1.42	7.20E-02	1.69	5.75E-03
Phvul.008G289500	K10775 - phenylalanine ammonia-lyase (E4.3.1.24) (1 of 6)	2.09	3.73E-03	2.06	1.27E-03	1.81	2.55E-02	1.80	7.39E-03
Phvul.001G130200	PTHR20961//PTHR20961:SF12 - GLYCOSYLTRANSFERASE // SUBFAMILY NOT NAMED (1 of 1)	2.08	7.50E-02	2.22	1.14E-02	1.88	1.55E-01	2.16	2.00E-02
Phvul.007G278900	PTHR23050 - CALCIUM BINDING PROTEIN (1 of 17)	2.07	5.13E-04	2.10	1.73E-04	1.87	3.99E-03	1.66	5.78E-03
Phvul.011G176950	3.2.1.39 - Glucan endo-1,3-beta-D-glucosidase / Laminarinase (1 of 65)	2.07	4.77E-03	2.75	2.36E-05	1.74	3.65E-02	2.80	2.04E-05
Phvul.008G086900	1.11.1.7 - Peroxidase / Lactoperoxidase (1 of 96)	2.05	1.55E-02	2.94	1.08E-06	1.09	3.83E-01	1.94	2.54E-03
Phvul.010G161100	PTHR31429:SF3 - WRKY TRANSCRIPTION FACTOR 40-RELATED (1 of 4)	2.05	2.92E-04	2.36	7.75E-06	1.71	6.04E-03	2.13	7.67E-05
Phvul.006G001500	PF00560//PF08263//PF13855 - Leucine Rich Repeat (LRR 1) // Leucine rich repeat N-terminal domain (LRRNT 2) // Leuc	2.05	1.35E-02	2.64	2.38E-04	1.53	1.25E-01	2.50	8.40E-04
Phvul.008G243700	PTHR21493//PTHR21493:SF2 - CGI-141-RELATED/LIPASE CONTAINING PROTEIN // SUBFAMILY NOT NAMED (1 of 9)	2.04	1.23E-01	1.77	1.76E-02	1.94	2.04E-01	1.71	3.23E-02
Phvul.006G153100	PTHR13683:SF268 - EUKARYOTIC ASPARTYL PROTEASE FAMILY PROTEIN (1 of 7)	2.04	1.59E-04	2.13	2.85E-05	1.89	1.01E-03	1.89	2.93E-04
Phvul.011G178400	PTHR11260:SF174 - GLUTATHIONE S-TRANSFERASE U19-RELATED (1 of 2)	2.02	1.73E-06	1.59	6.88E-05	1.82	1.03E-04	1.15	6.88E-03
Phvul.003G149500	PTHR18966:SF204 - GLUTAMATE RECEPTOR 2.5-RELATED (1 of 3)	2.02	2.19E-03	2.44	2.07E-05	1.92	5.95E-03	2.24	1.36E-04
Phvul.009G133500	PTHR33133:SF5 - EXPRESSED PROTEIN (1 of 2)	2.02	1.30E-01	2.53	7.83E-03	1.48	3.56E-01	2.46	1.52E-02
Phvul.008G238200	no data	2.00	1.08E-03	1.64	2.87E-03	1.75	8.50E-03	1.84	1.32E-03
Phvul.005G167700	2.7.11.1//3.2.1.14 - Non-specific serine/threonine protein kinase / Threonine-specific protein kinase // Chitinase / Poly-	1.99	1.20E-02	1.80	8.00E-03	1.52	1.19E-01	1.52	3.54E-02
Phvul.004G155900	PTHR31713:SF15 - PROTEIN SAR DEFICIENT 1 (1 of 2)	1.99	7.51E-02	2.19	9.34E-03	2.53	1.39E-02	2.19	1.20E-02
Phvul.009G045700	no data	1.98	2.97E-02	1.82	1.13E-02	1.78	7.33E-02	2.23	2.30E-03
Phvul.002G168700	PTHR33133:SF7 - F26K24.10 PROTEIN (1 of 2)	1.98	1.35E-03	2.70	1.76E-06	2.14	5.77E-04	2.60	4.64E-06
Phvul.009G154100	PTHR11785//PTHR11785:SF370 - AMINO ACID TRANSPORTER // SUBFAMILY NOT NAMED (1 of 1)	1.97	9.91E-02	1.97	2.95E-02	1.64	2.52E-01	1.92	4.18E-02
Phvul.001G042200	PTHR11941:SF96 - 3-HYDROXYISOBUTYRYL-COA HYDROLASE-LIKE PROTEIN 2, MITOCHONDRIAL (1 of 1)	1.97	1.23E-01	2.75	3.27E-03	1.36	3.88E-01	3.71	1.10E-04
Phvul.002G286600	KOG4837 - Uncharacterized conserved protein (1 of 2) Pathogenesis-related	1.97	2.35E-01	2.15	2.43E-02	1.71	3.75E-01	2.28	2.30E-02
Phvul.005G059000	PTHR10836//PTHR10836:SF46 - GLYCERALDEHYDE 3-PHOSPHATE DEHYDROGENASE // SUBFAMILY NOT NAMED (1 of 1)	1.97	4.39E-02	1.42	6.56E-02	1.78	1.09E-01	1.54	5.01E-02
Phvul.002G098200	PTHR31521:SF2 - EXPRESSED PROTEIN (1 of 1)	1.96	4.56E-03	1.95	9.53E-04	1.45	7.98E-02	1.78	3.97E-03
Phvul.010G155100	no data	1.96	4.32E-02	1.88	1.68E-02	1.96	5.42E-02	1.97	1.65E-02
Phvul.010G003300	no data	1.95	1.13E-02	2.72	3.41E-05	2.02	9.54E-03	3.37	2.34E-07
Phvul.008G290700	PTHR33179:SF7 - GENOMIC DNA, CHROMOSOME 3, P1 CLONE:MKA23 (1 of 2)	1.95	1.66E-03	2.13	1.12E-04	1.94	2.92E-03	2.24	6.81E-05
Phvul.004G070500	no data	1.95	8.43E-03	1.94	2.53E-03	1.59	6.08E-02	2.29	5.03E-04
Phvul.009G131900	PTHR27005:SF32 - F16F4.7 PROTEIN-RELATED (1 of 2)	1.95	4.90E-02	2.49	7.46E-03	1.30	3.20E-01	2.93	1.57E-03
Phvul.003G146700	PTHR11214//PTHR11214:SF141 - BETA-1,3-N-ACETYLGLUCOSAMINYLTRANSFERASE // SUBFAMILY NOT NAMED (1 of 1)	1.94	1.02E-01	2.69	5.83E-04	1.98	1.26E-01	2.98	1.10E-04
Phvul.007G127800	PTHR31153:SF3 - HISTONE H1FLK-LIKE PROTEIN-RELATED (1 of 4)	1.94	9.04E-05	1.34	4.47E-03	1.65	2.51E-03	1.06	3.46E-02
Phvul.006G102300	K03116 - sec-independent protein translocase protein TatA (tatA) (1 of 1)	1.93	8.29E-07	1.92	6.83E-07	1.59	2.76E-04	1.62	3.69E-05
Phvul.003G219200	PTHR31731:SF7 - BIFUNCTIONAL INHIBITOR/LIPID-TRANSFER PROTEIN/SEED STORAGE 2S ALBUMIN SUPERFAMILY PROT	1.93	8.28E-05	1.59	2.91E-04	1.48	9.49E-03	0.32	5.76E-01
Phvul.009G152800	PTHR31719:SF3 - GRAB1-LIKE PROTEIN (1 of 3)	1.93	2.87E-02	1.46	5.23E-02	1.76	7.20E-02	1.56	4.68E-02
Phvul.011G007701	no data	1.93	2.63E-02	1.19	1.01E-01	1.87	4.73E-02	0.03	9.78E-01
Phvul.005G136700	K08472 - mlo protein (MLO) (1 of 20)	1.93	5.96E-03	2.62	2.21E-05	1.14	2.19E-01	1.84	4.98E-03
Phvul.005G023600	PTHR10638:SF39 - AMINE OXIDASE-RELATED (1 of 2)	1.93	6.19E-03	1.97	1.15E-03	1.46	7.60E-02	1.60	1.13E-02
Phvul.002G026400	2.5.1.29 - Geranylgeranyl diphosphate synthase / Geranylgeranyl-PP synthetase (1 of 5)	1.92	2.26E-02	0.35	6.89E-01	1.07	3.52E-01	0.58	4.61E-01
Phvul.009G244100	PTHR24298:SF61 - CYTOCHROME P450 81D1-RELATED (1 of 13)	1.91	5.53E-03	1.94	9.87E-04	1.43	8.91E-02	1.42	2.25E-02
Phvul.001G004900	PF11961 - Domain of unknown function (DUF3475) (DUF3475) (1 of 15)	1.91	1.11E-03	2.05	1.11E-04	1.77	4.50E-03	1.87	6.44E-04
Phvul.009G118800	PTHR21704:SF19 - EXPRESSED PROTEIN-RELATED (1 of 1)	1.91	1.43E-01	2.78	3.12E-03	2.04	1.44E-01	2.53	1.18E-02
Phvul.008G094500	PF00069//PF00139 - Protein kinase domain (Pkinase) // Legume lectin domain (Lectin legB) (1 of 32)	1.90	1.66E-03	2.60	5.63E-06	1.83	4.15E-03	2.48	1.78E-05
Phvul.005G155800	PTHR22595//PTHR22595:SF61 - CHITINASE-RELATED // SUBFAMILY NOT NAMED (1 of 2)	1.90	8.30E-06	2.27	2.61E-08	1.87	4.51E-05	1.95	3.43E-06
Phvul.004G081900	PTHR13690:SF86 - TRANSCRIPTION FACTOR VIP1 (1 of 1)	1.90	3.97E-04	1.82	1.62E-04	1.40	3.11E-02	1.43	5.37E-03
Phvul.008G011900	PTHR19375//PTHR19375:SF227 - HEAT SHOCK PROTEIN 70KDA // SUBFAMILY NOT NAMED (1 of 5)	1.89	6.47E-02	2.04	6.34E-03	1.92	7.54E-02	1.53	6.06E-02
Phvul.010G137800	PTHR31953:SF16 - BETA-FRUCTOFURANOSIDASE, INSOLUBLE ISOENZYME CWINV1-RELATED (1 of 3)	1.89	2.70E-03	2.80	1.14E-06	1.77	8.50E-03	2.94	5.79E-07
Phvul.011G022400	1.1.1.195 - Cinnamyl-alcohol dehydrogenase / CAD (1 of 20)	1.88	6.57E-04	1.66	9.68E-04	1.42	3.02E-02	1.23	2.24E-02
Phvul.006G084800	PTHR31384:SF2 - AUXIN RESPONSE FACTOR 8 (1 of 2)	1.87	3.61E-02	2.11	2.85E-03	1.96	3.64E-02	1.75	2.06E-02
Phvul.006G162800	PF12819//PF13855 - Carbohydrate-binding protein of the ER (Malectin like) // Leucine rich repeat (LRR 8) (1 of 1)	1.87	1.27E-02	2.15	1.07E-03	1.21	2.21E-01	1.42	4.79E-02
Phvul.008G149600	PF00069//PF00560//PF08263//PF13855 - Protein kinase domain (Pkinase) // Leucine Rich Repeat (LRR 1) // Leucine ric	1.86	5.64E-03	2.39	4.64E-05	1.69	1.99E-02	2.64	8.89E-06
Phvul.011G201900	PTHR23155//PTHR23155:SF414 - LEUCINE-RICH REPEAT-CONTAINING PROTEIN // SUBFAMILY NOT NAMED (1 of 124)	1.85	1.08E-02	0.39	6.31E-01	0.37	8.23E-01	0.57	4.26E-01
Phvul.003G228300	PF16913 - Purine nucleobase transmembrane transport (PUNUT) (1 of 13)	1.84	3.33E-02	2.10	2.01E-03	1.59	1.16E-01	2.08	3.56E-03
Phvul.008G169800	PF01344//PF12937 - Kelch motif (Kelch 1) // F-box-like (F-box-like) (1 of 1)	1.83	7.07E-02	2.36	3.10E-03	1.97	5.54E-02	2.76	7.83E-04
Phvul.003G217400	no data	1.83	4.00E-02	1.70	1.40E-02	1.79	5.86E-02	0.81	2.98E-01
Phvul.003G295200	PTHR23024:SF157 - CARBOXYLESTERASE 12-RELATED (1 of 3)	1.83	6.24E-02	1.78	1.05E-02	1.55	1.83E-01	1.49	4.42E-02
Phvul.010G129000	2.7.11.1 - Non-specific serine/threonine protein kinase / Threonine-specific protein kinase (1 of 1198)	1.83	4.64E-02	2.12	2.50E-03	1.73	8.89E-02	1.94	8.78E-03
Phvul.002G265400	PTHR24073:SF462 - RAS-RELATED PROTEIN RABG1-RELATED (1 of 1)	1.82	4.07E-02	2.54	4.40E-04	1.53	1.32E-01	3.65	7.78E-07
Phvul.008G140800	no data	1.82	4.16E-02	2.37	1.38E-03	1.64	1.01E-01	2.37	2.68E-03
Phvul.007G050500	PF01657//PF07714 - Salt stress response/antifungal (Stress-antifung) // Protein tyrosine kinase (Pkinase Tyr) (1 of 40)	1.81	8.28E-05	1.77	2.83E-05	1.81	2.12E-04	1.52	5.07E-04
Phvul.001G155150	PF00689//PF00702 - Cation transporting ATPase, C-terminus (Cation ATPase C) // haloacid dehalogenase-like hydrolase	1.81	5.68E-03	2.42	2.24E-05	1.62	2.60E-02	2.19	1.78E-04
Phvul.007G109000	PTHR13683:SF268 - EUKARYOTIC ASPARTYL PROTEASE FAMILY PROTEIN (1 of 7)	1.81	2.68E-03	1.32	1.87E-02	0.99	2.53E-01	1.03	8.91E-02
Phvul.002G318500	PF00069//PF14380 - Protein kinase domain (Pkinase) // Wall-associated receptor kinase C-terminal (WAK assoc) (1 of 2	1.80	5.62E-02	1.69	2.48E-02	1.49	1.99E-01	1.90	1.12E-02
Phvul.005G027032	no data	1.80	1.41E-08	1.42	1.16E-05	1.37	1.69E-04	0.81	2.14E-02
Phvul.008G011500	PTHR13871//PTHR13871:SF40 - THIOREDOXIN // SUBFAMILY NOT NAMED (1 of 2)	1.80	8.58E-02	1.94	1.85E-02	1.52	2.17E-01	2.13	1.45E-02
Phvul.009G259000	K13066 - caffeic acid 3-O-methyltransferase (E2.1.1.68, COMT) (1 of 18)	1.79	1.44E-04	2.65	2.15E-09	1.54	3.30E-03	2.13	3.25E-06
Phvul.004G018900	K10529 - alpha-dioxygenase (DOX1) (1 of 2)	1.78	1.43E-01	1.59	1.29E-01	0.47	8.23E-01	2.25	2.86E-02
Phvul.004G107200	no data	1.77	5.01E-03	2.27	4.39E-05	1.79	5.77E-03	2.38	2.46E-05
Phvul.009G262300	PTHR22835:SF262 - GDSL ESTERASE/LIPASE 5-RELATED (1 of 3)	1.76	2.47E-04	1.86	1.40E-05	1.64	1.58E-03	1.74	7.90E-05
Phvul.003G142100	2.3.1.119//2.3.1.199 - Very-long-chain 3-oxoacyl-CoA synthase / Very-long-chain beta-ketoacyl-CoA synthase (1 of 6)	1.76	2.69E-02	3.32	1.60E-06	1.49	1.07E-01	2.77	9.45E-05
Phvul.008G093700	PTHR11431//PTHR11431:SF44 - FERRITIN // SUBFAMILY NOT NAMED (1 of 2)	1.76	1.35E-01	2.19	1.98E-02	1.64	1.98E-01	2.75	5.71E-03
Phvul.006G179200	no data	1.75	1.38E-01	3.12	2.69E-04	1.60	2.19E-01	3.13	3.96E-04
Phvul.003G022500	no data	1.75	2.55E-02	2.38	6.59E-05	1.78	3.23E-02	2.53	2.81E-05
Phvul.007G254700	PTHR23155//PTHR23155:SF563 - LEUCINE-RICH REPEAT-CONTAINING PROTEIN // SUBFAMILY NOT NAMED (1 of 29)	1.74	6.79E-02	1.75	1.53E-02	1.25	2.87E-01	2.04	6.30E-03
Phvul.009G085000	PTHR24128:SF24 - ANKYRIN REPEAT FAMILY PROTEIN-RELATED (1 of 9)	1.73	2.07E-01	2.95	9.08E-03	0.73	7.37E-01	3.28	3.96E-03
Phvul.002G270700	PTHR11183:SF56 - UDP-GLUCURONATE:XYLAN ALPHA-GLUCURONOSYLTRANSFERASE 3-RELATED (1 of 3)	1.73	6.20E-02	1.49	5.37E-02	1.25	2.59E-01	1.65	3.76E-02
Phvul.005G011200	PTHR27002:SF129 - INACTIVE G-TYPE LECTIN S-RECEPTOR-LIKE SERINE/THREONINE-PROTEIN KINASE SRK-RELATED (1 of	1.73	9.02E-05	1.52	2.34E-04	1.17	3.77E-02	1.41	1.10E-03
Phvul.007G040900	PF04450 - Peptidase of plants and bacteria (BSP) (1 of 3)	1.73	4.94E-04	2.07	5.70E-06	1.56	3.31E-03	2.40	2.24E-07
Phvul.001G182300	K13496 - UDP-glucosyl transferase 73C [EC:2.4.1.-] Glc - brassinosteroid (UGT73C) (1 of 11)	1.72	1.21E-01	2.19	8.77E-03	1.57	2.25E-01	2.12	1.66E-02
Phvul.008G012200	PF04535 - Domain of unknown function (DUF588) (DUF588) (1 of 39)	1.72	7.08E-02	2.93	1.50E-04	1.32	2.46E-01	2.45	2.34E-03
Phvul.008G044400	PF00069//PF13855 - Protein kinase domain (Pkinase) // Leucine rich repeat (LRR 8) (1 of 6)	1.72	1.24E-01	2.57	1.35E-03	1.24	3.76E-01	2.23	9.59E-03
Phvul.007G170900	PTHR13683:SF379 - ASPARTYL PROTEASE-LIKE PROTEIN (1 of 3)	1.71	1.88E-04	1.13	8.61E-03	1.53	2.20E-03	1.32	2.80E-03
Phvul.004G111900	PTHR10283//PTHR10283:SF94 - SOLUTE CARRIER FAMILY 13 MEMBER // SUBFAMILY NOT NAMED (1 of 4)	1.71	1.55E-02	1.72	3.73E-03	1.60	3.58E-02	1.41	2.50E-02
Phvul.010G134700	K15718 - linoleate 9S-lipoxygenase (LOX1 5) (1 of 19)	1.70	1.99E-02	2.25	2.48E-04	1.52	6.13E-02	1.84	3.96E-03
Phvul.006G086100	PTHR11615//PTHR11615:SF151 - NITRATE, FROMATE, IRON DEHYDROGENASE // SUBFAMILY NOT NAMED (1 of 3)	1.70	5.20E-02	2.40	7.44E-04	1.40	1.70E-01	3.30	3.43E-06
Phvul.003G022400	PTHR22814//PTHR22814:SF47 - COPPER TRANSPORT PROTEIN ATOX1-RELATED // SUBFAMILY NOT NAMED (1 of 8)	1.70	1.72E-01	2.32	7.91E-03	2.21	5.45E-02	2.68	2.75E-03
Phvul.007G049400	PTHR27002:SF122 - CYSTEINE-RICH RECEPTOR-LIKE PROTEIN KINASE 28-RELATED (1 of 17)	1.69	4.26E-02	3.11	1.48E-05	1.50	1.12E-01	3.07	3.16E-05
Phvul.003G101100	PF01535//PF13041 - PPR repeat (PPR) // PPR repeat family (PPR 2) (1 of 197)	1.69	1.14E-01	2.22	4.45E-03	1.37	2.76E-01	1.98	1.58E-02
Phvul.002G114100	PTHR22595:SF34 - BASIC ENDOCHITINASE B (1 of 8)	1.69	2.61E-03	0.69	1.52E-01	1.58	9.54E-03	0.63	2.00E-01
Phvul.003G244600	no data	1.68	4.77E-03	1.26	1.76E-02	0.98	2.21E-01	0.74	2.09E-01
Phvul.001G048900	PF07714//PF13947 - Protein tyrosine kinase (Pkinase Tyr) // Wall-associated receptor kinase galacturonan-binding (GUE	1.68	4.08E-01	2.57	2.59E-02	0.95	7.46E-01	1.70	1.79E-01
Phvul.009G066300	PF02458 - Transferase family (Transferase) (1 of 94)	1.68	3.92E-01	2.65	4.94E-02	1.79	4.17E-01	3.67	7.63E-03
Phvul.003G287800	2.2.1.7 - 1-deoxy-D-xylulose-5-phosphate synthase / DXP-synthase (1 of 6)	1.68	2.45E-02	1.65	7.61E-03	1.23	1.86E-01	1.46	2.70E-02
Phvul.003G020100	PTHR10209//PTHR10209:SF148 - OXIDOREDUCTASE, 2OG-FE II OXYGENASE FAMILY PROTEIN // SUBFAMILY NOT NAME	1.67	8.68E-03	1.42	9.56E-03	1.10	1.76E-01	1.29	2.38E-02
Phvul.006G043100	no data	1.66	2.14E-01	3.09	1.46E-03	2.04	1.20E-01	3.60	2.07E-04
Phvul.004G025800	no data	1.66	5.25E-03	2.34	1.18E-05	1.70	5.42E-03	2.67	6.20E-07
Phvul.003G158600	PF00704//PF07714 - Glycosyl hydrolases family 18 (Glyco hydro 18) // Protein tyrosine kinase (Pkinase Tyr) (1 of 2)	1.66	1.89E-01	2.13	2.73E-02	1.46	3.13E-01	1.75	9.96E-02
Phvul.010G062500	PTHR21495:SF77 - DIRIGENT PROTEIN 19 (1 of 6)	1.66	1.64E-03	1.84	1.13E-04	1.60	3.98E-03	1.72	5.20E-04
Phvul.003G011000	PTHR36346:SF1 - EXPRESSED PROTEIN (1 of 2)	1.66	2.26E-02	1.52	1.54E-02	1.71	2.68E-02	1.79	6.00E-03
Phvul.003G141900	no data	1.65	5.65E-02	1.37	4.93E-02	1.22	2.55E-01	1.01	1.79E-01
Phvul.007G081200	no data	1.65	2.50E-03	1.35	6.56E-03	1.24	5.59E-02	0.99	6.37E-02
Phvul.010G116000	PTHR10891:SF624 - CALCIUM-BINDING PROTEIN KIC (1 of 2)	1.65	2.17E-03	1.02	4.67E-02	1.75	1.76E-03	1.16	2.72E-02

Phvul.011G194501	PF01657 - Salt stress response/antifungal (Stress-antifung) (1 of 85)	1.65	7.07E-02	1.51	1.99E-02	1.07	3.92E-01	1.22	7.75E-02
Phvul.008G078500	PTHR10891:SF589 - CALCIUM-BINDING PROTEIN CML42-RELATED (1 of 2)	1.64	3.82E-05	1.44	1.53E-04	1.64	1.03E-04	1.48	1.34E-04
Phvul.010G059000	PF00069//PF00139 - Protein kinase domain (Pkinase) // Legume lectin domain (Lectin legB) (1 of 32)	1.64	2.60E-03	2.27	5.00E-06	1.61	5.11E-03	3.07	6.95E-10
Phvul.011G007800	PF00069//PF00560 - Protein kinase domain (Pkinase) // Leucine Rich Repeat (LRR 1) (1 of 2)	1.63	2.03E-01	2.29	1.87E-02	1.48	3.18E-01	2.35	2.09E-02
Phvul.005G108900	PF02458 - Transferase family (Transferase) (1 of 94)	1.63	1.96E-01	2.10	2.19E-02	1.12	4.66E-01	2.27	1.83E-02
Phvul.003G107100	PTHR24056//PTHR24056:SF176 - CELL DIVISION PROTEIN KINASE // SUBFAMILY NOT NAMED (1 of 2)	1.63	8.62E-02	0.91	2.46E-01	1.90	4.21E-02	1.75	1.22E-02
Phvul.003G174500	no data	1.63	1.73E-03	1.72	2.73E-04	1.58	3.85E-03	1.83	1.30E-04
Phvul.001G259000	PTHR10836:SF59 - GLYCERALDEHYDE-3-PHOSPHATE DEHYDROGENASE GAPC1, CYTOSOLIC (1 of 1)	1.63	3.42E-06	1.90	2.61E-08	1.21	2.92E-03	1.55	8.20E-06
Phvul.003G053900	no data	1.63	2.52E-02	1.25	4.95E-02	1.57	4.59E-02	1.41	3.20E-02
Phvul.008G185000	PTHR10579//PTHR10579:SF55 - CALCIUM-ACTIVATED CHLORIDE CHANNEL REGULATOR // SUBFAMILY NOT NAMED (1 o	1.63	4.76E-02	0.67	3.67E-01	1.41	1.46E-01	0.99	1.30E-01
Phvul.011G050100	PTHR22924 - LEGHEMOGLOBIN-RELATED (1 of 7)	1.62	7.36E-02	1.75	1.07E-02	1.53	1.25E-01	1.36	6.50E-02
Phvul.002G133400	PTHR23155//PTHR23155:SF633 - LEUCINE-RICH REPEAT-CONTAINING PROTEIN // SUBFAMILY NOT NAMED (1 of 2)	1.62	2.71E-03	1.46	2.05E-03	1.58	5.42E-03	1.33	7.01E-03
Phvul.008G029700	2.7.11.1 - Non-specific serine/threonine protein kinase / Threonine-specific protein kinase (1 of 1198)	1.62	2.62E-05	1.04	3.03E-03	1.52	2.25E-04	0.89	1.74E-02
Phvul.005G164500	PF00560//PF07714//PF12819 - Leucine Rich Repeat (LRR 1) // Protein tyrosine kinase (Pkinase Tyr) // Carbohydrate-bir	1.62	3.26E-04	1.25	2.47E-03	1.37	6.10E-03	0.68	1.41E-01
Phvul.003G224800	PTHR31692:SF10 - EXPANSIN-LIKE B1 (1 of 4)	1.62	2.13E-01	2.65	3.70E-03	1.40	3.37E-01	1.65	9.82E-02
Phvul.001G155201	PF00122//PF00690 - E1-E2 ATPase (E1-E2 ATPase) // Cation transporter/ATPase, N-terminus (Cation ATPase N) (1 of 1)	1.61	8.27E-02	2.18	1.56E-03	1.78	6.20E-02	2.15	2.73E-03
Phvul.003G052700	PTHR11699:SF180 - ALDEHYDE DEHYDROGENASE FAMILY 2 MEMBER B7, MITOCHONDRIAL (1 of 4)	1.61	2.63E-02	1.19	2.78E-02	1.26	1.64E-01	1.37	1.34E-02
Phvul.001G177700	K10775 - phenylalanine ammonia-lyase (E4.3.1.24) (1 of 6)	1.61	6.78E-03	2.19	2.35E-05	1.55	1.37E-02	2.45	3.25E-06
Phvul.008G194300	PTHR10774//PTHR10774:SF58 - EXTENDED SYNAPTOTAGMIN-RELATED // SUBFAMILY NOT NAMED (1 of 6)	1.61	3.23E-02	1.30	4.82E-02	1.55	5.51E-02	1.51	2.48E-02
Phvul.009G240000	3.6.3.5 - Zinc-exporting ATPase / Zn(2+)-exporting ATPase (1 of 2)	1.61	2.40E-02	1.01	7.09E-02	0.77	4.76E-01	1.41	1.00E-02
Phvul.008G048466	PTHR19317//PTHR19317:SF15 - PRENYLATED RAB ACCEPTOR 1-RELATED // SUBFAMILY NOT NAMED (1 of 3)	1.60	3.74E-02	2.46	3.06E-04	1.60	5.40E-02	2.65	1.12E-04
Phvul.005G079000	PTHR33510:SF2 - PROTEIN TIC 20-IV, CHLOROPLASTIC (1 of 2)	1.59	2.08E-02	0.66	2.70E-01	1.26	1.49E-01	1.14	3.15E-02
Phvul.011G203450	PF05678 - VQ motif (VQ) (1 of 39)	1.59	1.26E-01	2.03	4.09E-03	1.65	1.42E-01	2.49	4.71E-04
Phvul.008G140400	PTHR31251:SF4 - SQUAMOSA PROMOTER-BINDING-LIKE PROTEIN 8 (1 of 2)	1.59	1.12E-02	2.51	6.05E-06	1.31	7.30E-02	2.73	1.20E-06
Phvul.001G047800	PTHR22884//PTHR22884:SF351 - SET DOMAIN PROTEINS // SUBFAMILY NOT NAMED (1 of 1)	1.59	2.93E-02	0.78	2.08E-01	1.57	4.73E-02	0.60	3.55E-01
Phvul.002G297100	no data	1.59	9.17E-05	1.82	5.25E-06	1.50	5.41E-04	1.82	4.60E-06
Phvul.001G177800	K10775 - phenylalanine ammonia-lyase (E4.3.1.24) (1 of 6)	1.59	2.83E-02	2.27	1.72E-04	1.46	6.55E-02	2.28	2.39E-04
Phvul.010G033600	KOG3399 - Predicted Yippee-type zinc-binding protein (1 of 11)	1.59	9.41E-04	1.50	6.88E-04	1.33	1.35E-02	1.32	4.73E-03
Phvul.003G138200	K13065 - shikimate O-hydroxycinnamoyltransferase (E2.3.1.133, HCT) (1 of 15)	1.58	2.16E-02	1.77	1.29E-03	1.50	4.68E-02	1.56	6.85E-03
Phvul.007G029800	PTHR31042:SF10 - CORE-2/I-BRANCHING BETA-1,6-N-ACETYLGLUCOSAMINYLTRANSFERASE-LIKE PROTEIN (1 of 2)	1.58	8.52E-02	2.73	1.39E-04	0.99	4.25E-01	2.97	3.46E-05
Phvul.011G143300	K01601 - ribulose-bisphosphate carboxylase large chain (rbcL) (1 of 4)	1.58	1.05E-01	1.76	1.15E-02	0.43	7.98E-01	1.17	1.21E-01
Phvul.003G104400	PTHR13778:SF5 - GALACTURONOSYLTRANSFERASE-LIKE 4-RELATED (1 of 2)	1.58	1.59E-02	1.63	2.26E-03	1.63	1.73E-02	1.91	4.38E-04
Phvul.010G044900	no data	1.58	1.30E-01	1.91	1.14E-02	1.48	2.11E-01	1.86	1.87E-02
Phvul.002G207300	PTHR24078 - DNAJ HOMOLOG SUBFAMILY C MEMBER (1 of 123)	1.57	1.46E-01	2.84	6.91E-04	2.29	2.24E-02	2.80	1.04E-03
Phvul.005G084500	PTHR31719:SF34 - NAC DOMAIN-CONTAINING PROTEIN 19-RELATED (1 of 2)	1.57	2.08E-02	1.04	8.34E-02	1.54	3.45E-02	0.95	1.24E-01
Phvul.008G155000	PF01535//PF13041 - PPR repeat (PPR) // PPR repeat family (PPR 2) (1 of 197)	1.57	3.04E-02	1.37	2.68E-02	1.38	9.65E-02	1.30	4.39E-02
Phvul.007G105500	PTHR22950//PTHR22950:SF251 - AMINO ACID TRANSPORTER // SUBFAMILY NOT NAMED (1 of 1)	1.57	1.08E-02	1.51	4.13E-03	1.41	3.95E-02	1.23	2.91E-02
Phvul.002G044100	2.3.1.57 - Diamine N-acetyltransferase / Spermidine N(1)-acetyltransferase (1 of 1)	1.57	3.08E-03	1.30	6.84E-03	1.65	2.74E-03	1.20	1.80E-02
Phvul.008G238500	PTHR22849:SF43 - U-BOX DOMAIN-CONTAINING PROTEIN 54-RELATED (1 of 2)	1.57	9.32E-03	1.36	1.12E-02	1.77	3.31E-03	1.60	3.42E-03
Phvul.001G170800	PTHR26374:SF198 - ZINC FINGER PROTEIN ZAT11 (1 of 4)	1.57	2.16E-01	2.57	1.05E-02	1.99	9.65E-02	3.08	2.04E-03
Phvul.011G143700	PF00076 - RNA recognition motif. (a.k.a. RRM, RBD, or RNP domain) (RRM 1) (1 of 252)	1.57	2.29E-03	0.78	1.12E-01	1.37	1.76E-02	0.81	1.07E-01
Phvul.001G194900	PTHR31415:SF4 - HARPIN-INDUCED PROTEIN-LIKE-RELATED (1 of 9)	1.57	5.76E-03	2.00	7.43E-05	1.91	4.36E-04	2.16	2.04E-05
Phvul.007G048500	PTHR27002:SF122 - CYSTEINE-RICH RECEPTOR-LIKE PROTEIN KINASE 28-RELATED (1 of 17)	1.56	1.43E-02	1.85	7.58E-04	1.36	5.57E-02	2.16	1.07E-04
Phvul.002G309300	PTHR13832//PTHR13832:SF357 - PROTEIN PHOSPHATASE 2C // SUBFAMILY NOT NAMED (1 of 1)	1.56	1.08E-01	1.48	2.95E-02	1.60	1.29E-01	1.60	2.01E-02
Phvul.002G046800	PTHR23500:SF70 - SUGAR TRANSPORT PROTEIN 13 (1 of 4)	1.56	5.02E-02	1.41	2.28E-02	1.21	2.06E-01	2.23	2.93E-04
Phvul.005G042200	4.1.99.18 - Cyclic pyranopterin phosphate synthase / Molybdenum cofactor biosynthesis protein 1 (1 of 3)	1.56	3.63E-01	3.19	4.86E-03	1.72	3.36E-01	3.47	3.16E-03
Phvul.006G002700	K11838 - ubiquitin carboxyl-terminal hydrolase 7 (USP7, UBP15) (1 of 4)	1.55	4.69E-02	2.08	7.66E-04	1.39	1.17E-01	1.95	2.57E-03
Phvul.008G048532	PTHR19317//PTHR19317:SF15 - PRENYLATED RAB ACCEPTOR 1-RELATED // SUBFAMILY NOT NAMED (1 of 3)	1.55	3.08E-02	1.70	4.92E-03	1.41	7.97E-02	1.60	1.22E-02
Phvul.002G044500	PF01535//PF13041//PF14432 - PPR repeat (PPR) // PPR repeat family (PPR 2) // DYW family of nucleic acid deaminases	1.55	4.04E-02	0.58	4.97E-01	1.06	3.04E-01	0.58	4.95E-01
Phvul.001G040300	PTHR32133:SF124 - F-BOX ONLY PROTEIN 13 (1 of 2)	1.54	2.49E-01	2.81	2.01E-03	1.84	1.81E-01	2.88	2.28E-03
Phvul.003G278300	PF06749 - Protein of unknown function (DUF1218) (DUF1218) (1 of 14)	1.54	1.90E-02	1.87	6.91E-04	1.50	3.47E-02	1.98	5.00E-04
Phvul.L001078	no data	1.54	1.39E-01	2.26	2.94E-03	1.02	4.43E-01	2.61	8.15E-04
Phvul.003G256600	PTHR23070//PTHR23070:SF24 - BCS1 AAA-TYPE ATPASE // SUBFAMILY NOT NAMED (1 of 1)	1.54	2.52E-02	2.33	3.71E-05	1.67	1.73E-02	2.78	1.23E-06
Phvul.002G171400	PTHR11945:SF169 - MADS-BOX FAMILY PROTEIN (1 of 1)	1.54	7.79E-02	1.78	1.06E-02	0.74	5.56E-01	1.78	1.36E-02
Phvul.002G153400	PTHR18896//PTHR18896:SF15 - PHOSPHOLIPASE D // SUBFAMILY NOT NAMED (1 of 1)	1.54	1.54E-02	1.33	1.59E-02	1.33	5.97E-02	1.55	7.00E-03
Phvul.009G218800	PTHR11926:SF242 - UDP-GLYCOSYLTRANSFERASE 71B2-RELATED (1 of 1)	1.54	5.86E-03	1.45	2.20E-03	1.35	3.08E-02	1.14	2.62E-02
Phvul.008G088700	PTHR31087:SF11 - PROTEIN LURP-ONE-RELATED 1-RELATED (1 of 9)	1.53	3.98E-02	1.24	4.98E-02	1.34	1.21E-01	0.63	3.70E-01
Phvul.005G026966	no data	1.53	3.26E-04	1.73	9.35E-06	1.34	3.85E-03	1.15	5.59E-03
Phvul.002G257500	PTHR33109:SF3 - EPIDERMAL PATTERNING FACTOR-LIKE PROTEIN 1 (1 of 2)	1.53	1.00E-02	1.47	4.75E-03	1.38	3.70E-02	1.53	5.42E-03
Phvul.002G053800	no data	1.53	4.01E-02	0.87	1.84E-01	1.04	2.72E-01	0.81	2.25E-01
Phvul.009G061500	KOG0156 - Cytochrome P450 CYP2 subfamily (1 of 118)	1.53	2.10E-01	2.04	2.05E-02	0.94	5.31E-01	1.82	4.76E-02
Phvul.003G124100	3.5.4.3 - Guanine deaminase / Guanine aminase (1 of 3)	1.53	4.64E-02	0.33	6.86E-01	0.93	3.71E-01	0.61	4.00E-01
Phvul.004G155400	K04730 - interleukin-1 receptor-associated kinase 1 (IRAK1) (1 of 24)	1.52	3.92E-01	3.35	1.06E-03	1.23	5.64E-01	2.61	2.20E-02
Phvul.002G135900	PTHR21493//PTHR21493:SF2 - CGI-141-RELATED/LIPASE CONTAINING PROTEIN // SUBFAMILY NOT NAMED (1 of 9)	1.52	1.68E-01	1.81	8.15E-03	1.29	3.32E-01	1.46	4.97E-02
Phvul.011G043100	PTHR11629//PTHR11629:SF69 - VACUOLAR PROTON ATPASES // SUBFAMILY NOT NAMED (1 of 2)	1.52	3.42E-03	1.55	8.73E-04	1.46	8.24E-03	2.17	2.86E-06
Phvul.007G220400	no data	1.51	1.98E-02	0.77	2.20E-01	1.21	1.19E-01	1.41	1.63E-02
Phvul.003G233700	PF05678 - VQ motif (VQ) (1 of 39)	1.51	2.91E-02	1.97	3.04E-04	1.47	5.04E-02	1.18	4.87E-02
Phvul.006G102200	PTHR22952:SF90 - BASIC LEUCINE ZIPPER 63 (1 of 1)	1.51	6.37E-05	1.18	1.29E-03	1.22	3.74E-03	0.88	2.39E-02
Phvul.006G156500	K15813 - beta-amyrin synthase (LUP4) (1 of 8)	1.50	7.69E-03	2.36	1.40E-06	1.16	8.00E-02	1.58	2.28E-03
Phvul.006G033300	K04733 - interleukin-1 receptor-associated kinase 4 (IRAK4) (1 of 48)	1.50	3.15E-01	2.32	1.31E-02	2.27	8.72E-02	2.25	2.04E-02
Phvul.001G042100	5.4.99.5 - Chorismate mutase / Hydroxyphenylpyruvate synthase (1 of 4)	1.50	5.30E-01	3.41	1.88E-02	0.45	9.03E-01	4.05	8.60E-03
Phvul.003G272900	1.23.1.4 - (-)-lariciresinol reductase / Pinoresinol/lariciresinol reductase (1 of 2)	1.50	4.69E-03	1.60	5.10E-04	1.17	6.17E-02	1.73	2.42E-04
Phvul.003G010900	PTHR10218:SF226 - EXTRA-LARGE GUANINE NUCLEOTIDE-BINDING PROTEIN 2 (1 of 3)	1.50	1.01E-02	2.03	2.10E-05	1.09	1.35E-01	2.10	1.28E-05
Phvul.005G109800	PTHR10641//PTHR10641:SF541 - MYB-LIKE DNA-BINDING PROTEIN MYB // SUBFAMILY NOT NAMED (1 of 4)	1.50	5.65E-02	1.37	1.80E-02	1.81	1.50E-02	0.78	2.35E-01
Phvul.001G105700	no data	1.50	1.31E-01	1.99	5.77E-03	1.40	2.04E-01	1.61	3.50E-02
Phvul.007G048600	PTHR27002:SF122 - CYSTEINE-RICH RECEPTOR-LIKE PROTEIN KINASE 28-RELATED (1 of 17)	1.49	4.58E-02	2.22	2.72E-04	1.22	1.62E-01	2.40	1.12E-04
Phvul.011G041400	PTHR11223 - EXPORTIN 1/5 (1 of 4)	1.49	7.09E-02	1.55	1.71E-02	1.01	3.30E-01	1.90	4.36E-03
Phvul.011G143100	K15803 - (-)-germacrene D synthase (GERD) (1 of 8)	1.49	3.93E-02	0.51	4.51E-01	0.75	4.58E-01	0.06	9.42E-01
Phvul.008G176900	PF05340 - Protein of unknown function (DUF740) (DUF740) (1 of 6)	1.49	6.82E-02	1.16	7.94E-02	1.52	7.78E-02	1.73	5.37E-03
Phvul.009G240750	no data	1.49	4.18E-01	2.20	7.39E-02	3.01	4.73E-02	2.10	9.17E-02
Phvul.005G038400	no data	1.49	5.50E-02	1.67	7.80E-03	1.33	1.29E-01	1.48	2.61E-02
Phvul.006G207066	PTHR31235:SF27 - PEROXIDASE 62-RELATED (1 of 2)	1.49	2.20E-01	1.66	9.32E-02	0.92	5.60E-01	2.24	2.15E-02
Phvul.007G272700	PTHR14155//PTHR14155:SF178 - RING FINGER DOMAIN-CONTAINING // SUBFAMILY NOT NAMED (1 of 4)	1.49	8.24E-02	1.77	3.00E-03	1.46	1.28E-01	1.72	6.29E-03
Phvul.003G257900	PTHR10992//PTHR10992:SF804 - ALPHA/BETA HYDROLASE FOLD-CONTAINING PROTEIN // SUBFAMILY NOT NAMED (1 o	1.48	3.97E-02	1.92	1.13E-03	1.38	8.23E-02	1.94	1.62E-03
Phvul.008G076500	PTHR10366:SF425 - BRASSINOSTEROID METABOLIC PATHWAY PROTEIN BEN1 (1 of 6)	1.48	1.96E-01	2.00	1.09E-02	1.25	3.57E-01	2.08	1.21E-02
Phvul.010G042200	2.4.1.218 - Hydroquinone glucosyltransferase / Hydroquinone:O-glucosyltransferase (1 of 14)	1.48	2.56E-01	2.37	1.07E-02	1.09	4.84E-01	1.83	6.01E-02
Phvul.007G273400	PTHR10641//PTHR10641:SF650 - MYB-LIKE DNA-BINDING PROTEIN MYB // SUBFAMILY NOT NAMED (1 of 2)	1.48	4.85E-03	1.44	1.71E-03	1.34	2.17E-02	1.32	6.03E-03
Phvul.009G187800	PTHR11260:SF221 - GLUTATHIONE S-TRANSFERASE U9 (1 of 1)	1.47	1.16E-02	1.73	1.00E-03	1.24	6.40E-02	1.65	3.04E-03
Phvul.001G103600	PTHR10314:SF90 - S-SULFOCYSTEINE SYNTHASE, CHLOROPLASTIC-RELATED (1 of 1)	1.47	3.03E-01	1.95	7.45E-02	1.16	5.07E-01	3.00	3.87E-03
Phvul.008G011800	PTHR13871//PTHR13871:SF40 - THIOREDOXIN // SUBFAMILY NOT NAMED (1 of 2)	1.47	2.57E-01	2.46	9.11E-03	1.44	3.12E-01	2.75	4.43E-03
Phvul.006G168136	6.5.1.2 - DNA ligase (NAD(+)) / Polynucleotide ligase (NAD+) (1 of 1)	1.47	1.13E-03	1.27	1.89E-03	1.25	1.55E-02	1.29	2.57E-03
Phvul.002G019100	PTHR10015:SF166 - HEAT STRESS TRANSCRIPTION FACTOR B-1 (1 of 2)	1.45	1.37E-01	1.67	1.24E-02	0.79	5.67E-01	1.79	1.03E-02
Phvul.008G020700	PTHR23155//PTHR23155:SF497 - LEUCINE-RICH REPEAT-CONTAINING PROTEIN // SUBFAMILY NOT NAMED (1 of 6)	1.45	1.74E-02	2.38	1.40E-06	1.52	1.67E-02	1.94	1.36E-04
Phvul.008G194600	PTHR21596:SF25 - TRANSCRIPTION REGULATOR-LIKE-RELATED (1 of 3)	1.45	9.03E-02	1.36	3.58E-02	1.12	2.94E-01	1.37	4.22E-02
Phvul.009G138900	PF03106 - WRKY DNA -binding domain (WRKY) (1 of 91)	1.44	1.05E-01	2.49	1.53E-04	1.09	3.02E-01	2.87	1.41E-05
Phvul.002G148200	PTHR31221:SF20 - WRKY TRANSCRIPTION FACTOR 12-RELATED (1 of 10)	1.44	3.34E-01	2.06	3.92E-02	1.91	1.90E-01	1.74	1.06E-01
Phvul.010G070250	K00827 - alanine-glyoxylate transaminase / (R)-3-amino-2-methylpropionate-pyruvate transaminase (AGXT2) (1 of 3)	1.44	8.75E-02	2.72	1.53E-04	1.08	3.02E-01	2.66	2.99E-04
Phvul.011G152500	K03809 - NAD(P)H dehydrogenase (quinone) (wrbA) (1 of 11)	1.44	1.09E-01	1.19	8.49E-02	0.18	9.33E-01	1.60	1.87E-02
Phvul.006G079600	PTHR31956:SF9 - NON-SPECIFIC PHOSPHOLIPASE C1 (1 of 4)	1.44	4.20E-01	1.64	1.75E-01	2.02	2.32E-01	3.00	8.95E-03
Phvul.005G109000	PF02458 - Transferase family (Transferase) (1 of 94)	1.43	2.26E-01	3.38	4.76E-05	1.16	3.93E-01	4.02	2.23E-06
Phvul.005G163000	PTHR27003:SF105 - LEUCINE-RICH REPEAT PROTEIN KINASE-RELATED (1 of 25)	1.43	1.79E-02	1.55	2.05E-03	1.36	3.72E-02	1.43	7.04E-03
Phvul.006G016900	no data	1.43	1.85E-04	1.44	5.50E-05	1.18	5.46E-03	1.23	1.02E-03
Phvul.005G014900	PF01535//PF13041//PF14432 - PPR repeat (PPR) // PPR repeat family (PPR 2) // DYW family of nucleic acid deaminases	1.42	1.79E-01	2.05	9.40E-03	1.03	4.43E-01	1.95	1.87E-02

Phvul.004G106900	PF14111 - Domain of unknown function (DUF4283) (DUF4283) (1 of 81)	1.42	1.70E-03	1.36	1.01E-03	1.23	1.39E-02	1.28	3.04E-03
Phvul.001G048701	PF00069//PF13947 - Protein kinase domain (Pkinase) // Wall-associated receptor kinase galacturonan-binding (GUB WA	1.42	1.11E-01	1.96	1.19E-03	1.55	8.15E-02	1.72	7.68E-03
Phvul.004G123700	PTHR23024:SF209 - CARBOXYLESTERASE 9-RELATED (1 of 2)	1.41	3.38E-02	1.71	1.95E-03	1.58	2.04E-02	1.83	1.35E-03
Phvul.007G193400	PTHR31190:SF26 - ETHYLENE-RESPONSIVE TRANSCRIPTION FACTOR ERF096 (1 of 6)	1.41	4.80E-02	0.83	1.93E-01	1.31	9.81E-02	0.50	4.83E-01
Phvul.008G205600	4.1.3.4 - Hydroxymethylglutaryl-CoA lyase / HMG-CoA lyase (1 of 3)	1.41	7.98E-02	2.30	9.11E-05	1.20	2.09E-01	2.18	3.18E-04
Phvul.002G204500	KOG1441 - Glucose-6-phosphate/phosphate and phosphoenolpyruvate/phosphate antiporter (1 of 38)	1.41	7.67E-02	1.68	6.98E-03	1.00	3.12E-01	1.04	1.33E-01
Phvul.005G008800	K13989 - Derlin-2/3 (DERL2 3) (1 of 3)	1.41	1.33E-02	2.14	6.44E-06	1.23	5.59E-02	2.13	8.20E-06
Phvul.005G117900	PTHR23155//PTHR23155:SF497 - LEUCINE-RICH REPEAT-CONTAINING PROTEIN // SUBFAMILY NOT NAMED (1 of 6)	1.41	4.96E-02	1.93	3.73E-04	1.33	9.20E-02	1.73	2.47E-03
Phvul.010G006000	no data	1.41	5.38E-02	2.13	2.81E-04	1.17	1.63E-01	2.30	1.17E-04
Phvul.002G082300	PTHR12161:SF23 - REGULATOR OF VPS4 ACTIVITY IN THE MVB PATHWAY PROTEIN (1 of 2)	1.41	7.05E-03	1.45	1.25E-03	1.48	5.46E-03	1.69	2.07E-04
Phvul.006G165200	PTHR34777:SF1 - VQ MOTIF-CONTAINING PROTEIN (1 of 2)	1.41	1.38E-01	1.93	1.71E-02	1.66	7.20E-02	2.17	7.87E-03
Phvul.008G029932	2.7.11.1 - Non-specific serine/threonine protein kinase / Threonine-specific protein kinase (1 of 1198)	1.40	3.07E-04	1.19	9.90E-04	1.06	2.14E-02	1.22	1.11E-03
Phvul.011G028000	no data	1.40	1.25E-02	2.01	1.51E-05	1.21	5.00E-02	1.74	2.62E-04
Phvul.005G164300	PF00560//PF07714//PF12819 - Leucine Rich Repeat (LRR 1) // Protein tyrosine kinase (Pkinase Tyr) // Carbohydrate-bir	1.39	2.53E-04	1.34	1.53E-04	1.32	1.22E-03	0.87	2.21E-02
Phvul.004G134500	PF03140 - Plant protein of unknown function (DUF247) (1 of 36)	1.39	1.44E-01	1.17	1.27E-01	1.17	2.80E-01	1.53	4.40E-02
Phvul.002G207000	K13416 - brassinosteroid insensitive 1-associated receptor kinase 1 (BAK1) (1 of 3)	1.39	1.98E-02	1.80	2.92E-04	1.47	1.75E-02	1.87	2.77E-04
Phvul.002G314000	PTHR33168:SF12 - EXPRESSED PROTEIN (1 of 3)	1.38	8.11E-03	1.71	8.14E-05	1.16	5.32E-02	1.19	1.03E-02
Phvul.004G021200	KOG0156 - Cytochrome P450 CYP2 subfamily (1 of 118)	1.38	1.40E-01	1.84	1.11E-02	1.64	8.52E-02	1.91	1.28E-02
Phvul.006G197500	PTHR10334//PTHR10334:SF200 - CYSTEINE-RICH SECRETORY PROTEIN-RELATED Pathogenesis-related	1.37	3.91E-02	0.41	5.58E-01	0.90	2.96E-01	0.63	3.13E-01
Phvul.006G129700	PTHR31388:SF22 - PEROXIDASE 22-RELATED (1 of 10)	1.37	1.07E-03	1.00	1.13E-02	1.02	3.95E-02	0.50	2.66E-01
Phvul.001G066400	PF00069//PF12947//PF13947 - Protein kinase domain (Pkinase) // EGF domain (EGF 3) // Wall-associated receptor kina	1.36	2.55E-02	1.55	2.30E-03	1.05	1.61E-01	1.42	8.28E-03
Phvul.001G069248	3.1.4.46 - Glycerophosphodiester phosphodiesterase / Glycerophosphoryl diester phosphodiesterase (1 of 65)	1.36	1.39E-01	2.26	3.62E-04	1.18	2.77E-01	1.29	7.72E-02
Phvul.005G162600	PTHR27003:SF105 - LEUCINE-RICH REPEAT PROTEIN KINASE-RELATED (1 of 25)	1.35	8.34E-02	1.56	9.48E-03	1.24	1.54E-01	1.35	3.42E-02
Phvul.010G111900	PF03106 - WRKY DNA -binding domain (WRKY) (1 of 91)	1.35	1.03E-02	1.85	4.39E-05	1.37	1.21E-02	2.16	2.48E-06
Phvul.005G171900	KOG0265//KOG0279 - U5 snRNP-specific protein-like factor and related proteins // G protein beta subunit-like protein (1	1.34	1.72E-01	1.77	1.21E-02	1.61	1.04E-01	2.25	1.69E-03
Phvul.010G117200	PTHR24326:SF122 - HOMEOBOX-LEUCINE ZIPPER PROTEIN ATHB-12-RELATED (1 of 3)	1.34	1.09E-01	1.33	3.42E-02	1.32	1.51E-01	1.55	1.47E-02
Phvul.009G061400	KOG0156 - Cytochrome P450 CYP2 subfamily (1 of 118)	1.34	4.92E-02	1.60	2.92E-03	1.21	1.16E-01	1.26	2.80E-02
Phvul.008G090300	K13424 - WRKY transcription factor 33 (WRKY33) (1 of 4)	1.34	4.34E-03	1.57	1.30E-04	1.43	2.92E-03	1.61	1.30E-04
Phvul.005G164400	PF00560//PF07714//PF12819 - Leucine Rich Repeat (LRR 1) // Protein tyrosine kinase (Pkinase Tyr) // Carbohydrate-bir	1.34	6.78E-03	1.63	1.13E-04	1.17	3.58E-02	1.25	5.03E-03
Phvul.009G073000	no data	1.33	9.84E-03	1.39	1.46E-03	1.15	4.74E-02	1.32	3.58E-03
Phvul.008G020750	PTHR23155//PTHR23155:SF497 - LEUCINE-RICH REPEAT-CONTAINING PROTEIN // SUBFAMILY NOT NAMED (1 of 6)	1.33	1.35E-01	1.71	8.52E-03	1.32	1.83E-01	0.92	2.23E-01
Phvul.009G070800	PF13912 - C2H2-type zinc finger (zf-C2H2 6) (1 of 42)	1.33	2.44E-03	1.14	3.84E-03	1.54	4.09E-04	1.26	2.11E-03
Phvul.004G141200	PTHR23180:SF262 - ADP-RIBOSYLATION FACTOR GTPASE-ACTIVATING PROTEIN AGD15-RELATED (1 of 2)	1.33	8.04E-02	1.74	2.82E-03	1.17	1.76E-01	1.84	2.56E-03
Phvul.008G211200	PF09790 - Hyccin (Hyccin) (1 of 2)	1.33	1.15E-02	1.39	9.71E-04	0.99	1.32E-01	1.23	5.82E-03
Phvul.002G275000	PTHR13683:SF227 - ASPARTYL PROTEASE FAMILY PROTEIN (1 of 2)	1.32	1.84E-03	1.88	1.02E-06	1.65	1.03E-04	1.78	3.43E-06
Phvul.003G129700	PTHR11017:SF169 - DISEASE RESISTANCE PROTEIN-RELATED (1 of 1)	1.32	3.43E-01	2.33	1.07E-02	1.49	3.04E-01	2.63	5.49E-03
Phvul.002G211600	PTHR14155//PTHR14155:SF101 - RING FINGER DOMAIN-CONTAINING // SUBFAMILY NOT NAMED (1 of 2)	1.32	9.57E-02	1.97	1.06E-03	0.89	3.71E-01	1.80	4.24E-03
Phvul.003G160600	PTHR22595//PTHR22595:SF47 - CHITINASE-RELATED // SUBFAMILY NOT NAMED (1 of 2)	1.32	1.57E-02	1.78	1.11E-04	1.09	8.23E-02	1.40	4.21E-03
Phvul.008G030000	2.7.11.1 - Non-specific serine/threonine protein kinase / Threonine-specific protein kinase (1 of 1198)	1.31	4.31E-02	1.19	2.11E-02	1.30	6.26E-02	1.22	2.32E-02
Phvul.007G181800	K10664 - E3 ubiquitin-protein ligase ATL6/9/15/31/42/55 [EC:6.3.2.19] (ATL6S) (1 of 10)	1.31	6.16E-03	1.67	2.77E-05	1.51	1.25E-03	1.57	1.30E-04
Phvul.010G057000	PTHR31284:SF9 - HAD SUPERFAMILY, SUBFAMILY IIIB ACID PHOSPHATASE (1 of 2)	1.31	4.26E-02	1.60	2.38E-03	1.29	6.13E-02	1.63	2.77E-03
Phvul.011G108600	PTHR19836//PTHR19836:SF22 - 30S RIBOSOMAL PROTEIN S14 // SUBFAMILY NOT NAMED (1 of 1)	1.31	1.88E-01	1.46	5.11E-02	1.60	1.09E-01	2.16	4.17E-03
Phvul.001G049000	PF07714//PF13947 - Protein tyrosine kinase (Pkinase Tyr) // Wall-associated receptor kinase galacturonan-binding (GUE	1.30	3.35E-01	2.01	1.79E-02	1.63	2.13E-01	1.59	8.82E-02
Phvul.003G114700	no data	1.30	4.17E-03	0.34	5.03E-01	0.63	3.34E-01	0.22	6.92E-01
Phvul.001G014600	no data	1.30	4.87E-03	1.09	7.14E-03	1.12	3.23E-02	1.00	1.88E-02
Phvul.004G024900	PTHR23070:SF44 - AAA-TYPE ATPASE FAMILY PROTEIN-RELATED (1 of 3)	1.30	2.26E-02	1.30	7.57E-03	1.36	2.25E-02	1.76	3.20E-04
Phvul.001G249500	no data	1.30	2.29E-01	0.75	4.00E-01	1.30	2.67E-01	1.73	3.17E-02
Phvul.002G217900	PF00069//PF08263 - Protein kinase domain (Pkinase) // Leucine rich repeat N-terminal domain (LRRNT 2) (1 of 19)	1.29	4.50E-01	2.43	1.41E-02	0.77	7.45E-01	2.62	1.18E-02
Phvul.011G179600	PTHR14879 - CASPASE REGULATOR, RING FINGER DOMAIN-CONTAINING (1 of 1)	1.29	2.80E-02	1.77	2.69E-04	0.96	1.79E-01	1.94	8.86E-05
Phvul.003G098200	K02971 - small subunit ribosomal protein S21e (RP-S21e, RPS21) (1 of 2)	1.29	1.29E-01	1.37	4.19E-02	1.21	1.98E-01	1.42	3.97E-02
Phvul.008G011400	PTHR19375//PTHR19375:SF227 - HEAT SHOCK PROTEIN 70KDA // SUBFAMILY NOT NAMED (1 of 5)	1.29	3.03E-01	2.72	1.03E-04	1.61	1.83E-01	2.79	7.51E-05
Phvul.009G146800	PF08787 - Alginate lyase (Alginate lyase2) (1 of 1)	1.28	7.89E-03	1.32	1.79E-03	0.97	9.26E-02	0.57	2.39E-01
Phvul.003G166800	PTHR33021:SF55 - BLUE COPPER PROTEIN (1 of 6)	1.28	3.17E-01	2.05	2.45E-02	1.71	1.54E-01	2.32	1.09E-02
Phvul.007G101400	no data	1.28	1.72E-01	1.76	5.70E-03	1.17	2.68E-01	0.89	2.27E-01
Phvul.006G020700	K04733 - interleukin-1 receptor-associated kinase 4 (IRAK4) (1 of 48)	1.27	1.40E-02	1.05	1.94E-02	1.19	3.53E-02	0.83	8.67E-02
Phvul.009G190863	PTHR33021:SF55 - BLUE COPPER PROTEIN (1 of 6)	1.27	1.78E-01	0.96	2.18E-01	1.68	4.62E-02	-0.08	9.48E-01
Phvul.008G225500	PTHR12565//PTHR12565:SF181 - STEROL REGULATORY ELEMENT-BINDING PROTEIN // SUBFAMILY NOT NAMED (1 of 3)	1.25	9.48E-02	1.50	1.08E-02	1.37	7.37E-02	1.83	2.68E-03
Phvul.008G012000	PTHR10891//PTHR10891:SF615 - EF-HAND CALCIUM-BINDING DOMAIN CONTAINING PROTEIN // SUBFAMILY NOT NAM	1.25	6.21E-02	1.27	3.05E-03	0.80	3.98E-01	1.23	5.67E-03
Phvul.006G210400	no data	1.25	1.41E-03	1.10	2.51E-03	1.04	1.97E-02	0.92	1.60E-02
Phvul.010G028200	PF00931//PF13676 - NB-ARC domain (NB-ARC) // TIR domain (TIR 2) (1 of 30)	1.24	1.03E-01	1.21	3.57E-02	0.81	4.07E-01	1.10	7.08E-02
Phvul.007G134700	PTHR11260//PTHR11260:SF227 - GLUTATHIONE S-TRANSFERASE, GST, SUPERFAMILY, GST DOMAIN CONTAINING // SUE	1.24	2.55E-01	2.37	2.64E-03	0.91	4.84E-01	1.54	6.68E-02
Phvul.011G025200	K12392 - AP-1 complex subunit beta-1 (AP1B1) (1 of 2)	1.24	9.01E-02	1.35	1.94E-02	1.18	1.42E-01	0.93	1.39E-01
Phvul.011G063100	PTHR14194:SF38 - PROTEIN PLASTID TRANSCRIPTIONALLY ACTIVE 16 (1 of 1)	1.24	2.00E-01	1.60	2.70E-02	0.74	5.67E-01	1.60	3.32E-02
Phvul.010G090100	no data	1.24	2.89E-01	2.09	1.96E-02	2.16	9.50E-03	3.19	1.53E-05
Phvul.002G223500	no data	1.24	1.38E-02	0.89	4.72E-02	0.98	9.73E-02	0.63	1.94E-01
Phvul.005G065300	PF11145 - Protein of unknown function (DUF2921) (DUF2921) (1 of 6)	1.24	8.05E-03	1.52	1.33E-04	1.12	3.11E-02	1.32	1.57E-03
Phvul.001G249600	K16282 - E3 ubiquitin-protein ligase RHA2 [EC:6.3.2.19] (RHA2) (1 of 4)	1.23	6.37E-02	1.45	5.56E-03	0.93	2.43E-01	1.38	1.15E-02
Phvul.006G205300	PTHR31279:SF12 - PHI-1-LIKE PROTEIN (1 of 2)	1.23	2.26E-02	1.25	5.36E-03	0.74	3.16E-01	0.88	7.65E-02
Phvul.001G199700	no data	1.23	4.47E-03	0.67	1.03E-01	1.05	3.23E-02	0.47	2.83E-01
Phvul.001G039900	PF07082 - Protein of unknown function (DUF1350) (DUF1350) (1 of 2)	1.23	3.03E-01	2.08	5.93E-03	1.26	3.27E-01	2.80	2.01E-04
Phvul.004G107100	no data	1.23	2.51E-02	1.80	7.30E-05	1.35	1.44E-02	2.24	9.39E-07
Phvul.008G094900	PTHR11730:SF38 - AMMONIUM TRANSPORTER 2 (1 of 3)	1.23	1.51E-01	1.54	1.61E-02	0.97	3.48E-01	1.50	2.61E-02
Phvul.003G137600	2.4.1.207 - Xyloglucan:xyloglucosyl transferase / Xyloglucan endotransglycosylase (1 of 34)	1.22	8.43E-03	1.23	2.79E-03	1.12	2.99E-02	0.85	5.48E-02
Phvul.004G061900	PTHR23500:SF118 - PLASTIDIC GLUCOSE TRANSPORTER 4 (1 of 3)	1.22	4.56E-02	0.70	2.15E-01	0.93	2.11E-01	0.42	4.92E-01
Phvul.002G263200	no data	1.22	2.11E-01	1.58	2.65E-02	0.97	4.04E-01	1.54	3.82E-02
Phvul.001G126600	no data	1.22	4.00E-02	1.18	1.61E-02	0.94	1.91E-01	0.43	4.60E-01
Phvul.008G289200	no data	1.21	1.85E-02	1.77	4.94E-05	1.27	1.81E-02	1.87	2.25E-05
Phvul.004G172600	PTHR10509//PTHR10509:SF29 - O-METHYLTRANSFERASE-RELATED // SUBFAMILY NOT NAMED (1 of 2)	1.21	1.26E-03	1.23	3.39E-04	0.90	4.62E-02	0.94	1.00E-02
Phvul.004G175900	PF00069//PF00560//PF08263 - Protein kinase domain (Pkinase) // Leucine Rich Repeat (LRR 1) // Leucine rich repeat N	1.21	1.27E-01	1.74	3.15E-03	1.18	1.82E-01	1.49	1.73E-02
Phvul.008G164300	PTHR10209:SF165 - FLAVONOL SYNTHASE 3-RELATED (1 of 1)	1.21	2.99E-04	1.29	3.42E-05	1.08	3.17E-03	1.19	1.78E-04
Phvul.003G268600	PTHR22595:SF34 - BASIC ENDOCHITINASE B (1 of 8)	1.21	1.70E-02	0.90	4.77E-02	0.81	2.06E-01	0.45	3.82E-01
Phvul.004G155600	PF07714//PF14380 - Protein tyrosine kinase (Pkinase Tyr) // Wall-associated receptor kinase C-terminal (WAK assoc) (1	1.21	4.17E-02	1.75	2.82E-04	0.98	1.59E-01	1.72	5.03E-04
Phvul.004G039100	K04730 - interleukin-1 receptor-associated kinase 1 (IRAK1) (1 of 24)	1.20	8.46E-02	1.74	1.18E-03	1.44	3.56E-02	1.80	1.25E-03
Phvul.006G212500	K14815 - mRNA turnover protein 4 (MRT4) (1 of 1)	1.20	4.78E-02	1.26	1.26E-02	1.04	1.35E-01	2.52	2.02E-07
Phvul.005G078100	KOG4579 - Leucine-rich repeat (LRR) protein associated with apoptosis in muscle tissue (1 of 14)	1.20	9.48E-02	1.86	8.58E-04	1.00	2.31E-01	1.87	1.32E-03
Phvul.004G069000	PF03140 - Plant protein of unknown function (DUF247) (1 of 36)	1.20	1.65E-01	1.98	2.44E-03	0.99	3.29E-01	1.49	3.74E-02
Phvul.004G154600	2.7.11.1 - Non-specific serine/threonine protein kinase / Threonine-specific protein kinase (1 of 1198)	1.19	4.47E-01	2.75	6.09E-03	0.55	8.10E-01	2.87	6.31E-03
Phvul.011G056516	K10773 - endonuclease III (NTH) (1 of 3)	1.19	1.92E-02	-0.23	7.09E-01	0.29	7.53E-01	-0.25	6.77E-01
Phvul.009G261400	PTHR14155:SF99 - RING-H2 FINGER PROTEIN ATL3 (1 of 5)	1.19	1.49E-01	1.54	1.38E-02	1.21	1.76E-01	1.77	6.64E-03
Phvul.002G222800	K04718 - sphingosine kinase (SPHK) (1 of 2)	1.19	1.10E-01	1.57	1.59E-03	1.32	8.08E-02	1.23	2.07E-02
Phvul.011G162700	PTHR31165:SF9 - PROTEIN LIGHT-DEPENDENT SHORT HYPOCOTYLS 7-RELATED (1 of 2)	1.19	2.34E-01	1.34	3.00E-02	1.07	3.52E-01	0.36	6.77E-01
Phvul.005G133400	PTHR18952:SF111 - ALPHA CARBONIC ANHYDRASE 3 (1 of 3)	1.19	4.80E-02	1.64	7.09E-04	0.95	1.86E-01	1.13	2.84E-02
Phvul.009G183100	K18875 - enhanced disease susceptibility 1 protein (EDS1) (1 of 2)	1.18	2.12E-02	1.57	2.91E-04	1.17	3.33E-02	2.01	3.43E-06
Phvul.006G112200	PF03106 - WRKY DNA -binding domain (WRKY) (1 of 91)	1.18	4.61E-03	1.36	2.35E-04	1.11	1.37E-02	1.61	1.31E-05
Phvul.001G130900	PTHR19241//PTHR19241:SF247 - ATP-BINDING CASSETTE TRANSPORTER // SUBFAMILY NOT NAMED (1 of 5)	1.18	2.89E-01	2.17	4.81E-03	0.90	4.88E-01	2.06	1.07E-02
Phvul.006G152100	PF14416 - PMR5 N terminal Domain (PMR5N) (1 of 64)	1.18	2.75E-01	1.36	6.16E-02	1.33	2.44E-01	1.51	4.42E-02
Phvul.007G066300	no data	1.18	3.59E-01	3.87	2.47E-05	1.25	3.58E-01	3.37	5.03E-04
Phvul.005G088000	K11251 - histone H2A (H2A) (1 of 14)	1.18	3.00E-02	1.25	4.55E-03	0.65	3.83E-01	0.93	5.19E-02
Phvul.007G035500	PF14009 - Domain of unknown function (DUF4228) (DUF4228) (1 of 38)	1.18	2.31E-01	1.55	2.52E-02	0.81	5.18E-01	2.02	3.28E-03
Phvul.001G132301	PTHR23155//PTHR23155:SF563 - LEUCINE-RICH REPEAT-CONTAINING PROTEIN // SUBFAMILY NOT NAMED (1 of 29)	1.17	3.09E-01	1.66	4.42E-02	0.94	5.13E-01	2.21	6.15E-03
Phvul.003G166700	PTHR33021:SF55 - BLUE COPPER PROTEIN (1 of 6)	1.17	1.07E-01	1.32	2.14E-02	1.35	6.62E-02	1.60	7.63E-03
Phvul.009G043100	PF03106 - WRKY DNA -binding domain (WRKY) (1 of 91)	1.17	5.96E-02	2.18	6.70E-06	1.20	6.26E-02	2.77	1.47E-08
Phvul.002G149300	PTHR11206//PTHR11206:SF80 - MULTIDRUG RESISTANCE PROTEIN // SUBFAMILY NOT NAMED (1 of 2)	1.17	2.37E-02	1.50	6.04E-04	0.99	9.47E-02	1.11	1.75E-02

Phvul.001G145800	PTHR21495:SF71 - DIRIGENT PROTEIN 1-RELATED (1 of 10)	1.17	7.97E-02	1.64	1.31E-03	1.19	9.65E-02	1.38	1.12E-02
Phvul.009G087400	PTHR31429:SF3 - WRKY TRANSCRIPTION FACTOR 40-RELATED (1 of 4)	1.16	3.58E-01	2.43	3.00E-03	2.15	3.22E-02	3.30	3.01E-05
Phvul.009G125000	no data	1.16	5.34E-02	1.57	1.01E-03	1.07	1.07E-01	1.21	1.71E-02
Phvul.008G273801	KOG4579 - Leucine-rich repeat (LRR) protein associated with apoptosis in muscle tissue (1 of 14)	1.16	1.16E-03	1.16	3.62E-04	1.05	7.23E-03	0.90	1.03E-02
Phvul.006G090200	no data	1.16	7.19E-02	1.53	2.88E-03	1.06	1.40E-01	1.51	5.18E-03
Phvul.005G057500	PTHR10044:SF107 - BOI-RELATED E3 UBIQUITIN-PROTEIN LIGASE 3-RELATED (1 of 1)	1.16	2.54E-02	0.96	3.37E-02	0.99	9.77E-02	0.76	1.10E-01
Phvul.010G065600	3.1.1.11 - Pectinesterase / Pectin methylesterase (1 of 92)	1.16	2.23E-01	1.28	8.43E-02	1.30	1.86E-01	1.67	2.59E-02
Phvul.008G066300	PTHR10024//PTHR10024:SF219 - SYNAPTOTAGMIN // SUBFAMILY NOT NAMED (1 of 2)	1.15	6.14E-02	1.88	1.16E-05	1.20	6.36E-02	1.78	4.68E-05
Phvul.008G034000	2.4.1.85 - Cyanohydrin beta-glucosyltransferase / Uridine diphosphoglucose:aldehyde cyanohydrin beta-glucosyltransfe	1.15	4.88E-01	1.58	1.48E-01	1.01	6.03E-01	2.21	3.60E-02
Phvul.010G155900	PF02298 - Plastocyanin-like domain (Cu bind like) (1 of 53)	1.15	3.43E-01	2.50	1.28E-03	1.68	1.18E-01	2.35	3.69E-03
Phvul.011G113700	no data	1.15	6.33E-02	1.31	7.14E-03	1.23	5.33E-02	1.26	1.38E-02
Phvul.008G012600	PTHR19375:SF232 - MEDIATOR OF RNA POLYMERASE II TRANSCRIPTION SUBUNIT 37E-RELATED (1 of 5)	1.15	6.06E-02	1.38	4.06E-03	1.29	3.65E-02	1.38	5.61E-03
Phvul.002G197800	PF07712 - Stress up-regulated Nod 19 (SURNod19) (1 of 15)	1.15	3.34E-01	2.04	1.20E-02	0.86	5.41E-01	1.88	2.78E-02
Phvul.003G217500	PTHR12000:SF3 - LEGUMAIN (1 of 1)	1.14	4.77E-02	1.00	3.97E-02	1.03	1.10E-01	1.04	3.69E-02
Phvul.004G098201	PTHR31232:SF5 - GENOMIC DNA, CHROMOSOME 3, TAC CLONE:K14A17-RELATED (1 of 41)	1.14	1.28E-02	0.99	1.47E-02	0.50	4.48E-01	-0.04	9.54E-01
Phvul.008G134800	no data	1.14	5.65E-02	1.10	2.20E-02	0.90	2.12E-01	1.14	2.30E-02
Phvul.007G024900	KOG2504 - Monocarboxylate transporter (1 of 2)	1.14	3.35E-01	1.83	1.97E-02	0.82	5.79E-01	1.45	8.89E-02
Phvul.001G108101	PTHR24411:SF35 - BTB/POZ AND MATH DOMAIN-CONTAINING PROTEIN 1-RELATED (1 of 4)	1.14	1.20E-01	1.57	4.85E-03	1.03	2.20E-01	1.76	2.60E-03
Phvul.002G113000	PTHR22950//PTHR22950:SF233 - AMINO ACID TRANSPORTER // SUBFAMILY NOT NAMED (1 of 1)	1.14	8.49E-02	1.51	4.02E-03	1.19	8.62E-02	1.54	5.31E-03
Phvul.011G098100	no data	1.13	1.30E-01	1.12	4.49E-02	0.85	3.59E-01	0.93	1.20E-01
Phvul.003G020000	1.14.20.2 - 2,4-dihydroxy-1,4-benzoxazin-3-one-glucoside dioxygenase / DIBOA-Glc dioxygenase (1 of 1)	1.13	1.11E-01	1.65	2.01E-03	0.82	3.46E-01	1.52	6.29E-03
Phvul.005G070600	no data	1.13	1.21E-01	1.23	3.16E-02	0.93	2.81E-01	1.29	3.06E-02
Phvul.001G048600	3.1.4.46 - Glycerophosphodiester phosphodiesterase / Glycerophosphoryl diester phosphodiesterase (1 of 65)	1.12	3.98E-01	1.98	3.25E-03	1.25	3.63E-01	1.79	1.25E-02
Phvul.008G079800	PTHR27003:SF36 - LEUCINE-RICH REPEAT PROTEIN KINASE-LIKE PROTEIN (1 of 10)	1.12	8.20E-02	1.32	9.40E-03	0.87	2.60E-01	1.18	2.82E-02
Phvul.010G148100	K17362 - acyl-coenzyme A thioesterase 13 [EC:3.1.2.-] (ACOT13) (1 of 5)	1.12	2.60E-02	0.98	1.08E-02	0.93	1.28E-01	0.82	4.53E-02
Phvul.004G100014	KOG0472 - Leucine-rich repeat protein (1 of 95)	1.12	8.95E-02	2.26	1.16E-05	1.43	2.22E-02	2.39	4.15E-06
Phvul.011G147800	PTHR31719:SF34 - NAC DOMAIN-CONTAINING PROTEIN 19-RELATED (1 of 2)	1.12	5.08E-01	1.73	8.74E-02	1.30	4.49E-01	2.10	3.99E-02
Phvul.008G242600	PTHR24095:SF177 - ACYL-ACTIVATING ENZYME 5, PEROXISOMAL-RELATED (1 of 2)	1.11	5.66E-02	1.11	2.07E-02	1.06	9.72E-02	1.11	2.69E-02
Phvul.006G015211	PTHR23153//PTHR23153:SF36 - UBX-RELATED // SUBFAMILY NOT NAMED (1 of 1)	1.10	1.10E-01	1.18	3.00E-02	1.19	9.86E-02	1.28	2.22E-02
Phvul.006G186600	PTHR11772//PTHR11772:SF19 - ASPARAGINE SYNTHETASE // SUBFAMILY NOT NAMED (1 of 1)	1.10	9.08E-02	1.03	5.09E-02	0.92	2.24E-01	1.13	3.62E-02
Phvul.008G211800	K09499 - T-complex protein 1 subunit eta (CCT7) (1 of 1)	1.10	5.08E-01	-2.80	9.01E-03	-0.49	8.27E-01	-2.03	6.46E-02
Phvul.003G179500	K01530 - phospholipid-translocating ATPase (E3.6.3.1) (1 of 9)	1.09	1.68E-02	1.17	2.71E-03	0.90	9.20E-02	0.95	2.20E-02
Phvul.003G150400	PTHR10788:SF30 - ALPHA,ALPHA-TREHALOSE-PHOSPHATE SYNTHASE [UDP-FORMING] 11-RELATED (1 of 1)	1.09	1.64E-01	0.80	2.18E-01	0.96	2.76E-01	1.40	2.58E-02
Phvul.007G161100	no data	1.09	9.13E-02	1.45	3.47E-03	0.74	3.60E-01	1.55	2.77E-03
Phvul.004G039501	2.7.11.1 - Non-specific serine/threonine protein kinase / Threonine-specific protein kinase (1 of 1198)	1.09	1.30E-01	0.70	2.41E-01	0.96	2.46E-01	1.18	3.63E-02
Phvul.008G169900	PTHR13683//PTHR13683:SF339 - ASPARTYL PROTEASES // SUBFAMILY NOT NAMED (1 of 1)	1.08	2.43E-01	1.94	3.83E-03	1.48	9.42E-02	2.16	1.83E-03
Phvul.011G014400	PTHR23155:SF402 - DISEASE RESISTANCE PROTEIN RPP13-RELATED (1 of 8)	1.08	5.96E-03	1.05	2.16E-03	0.92	3.83E-02	1.10	2.06E-03
Phvul.002G194700	no data	1.08	4.43E-02	1.32	3.49E-03	1.46	3.30E-03	1.43	2.20E-03
Phvul.011G024700	no data	1.08	1.57E-02	1.13	3.18E-03	1.13	1.51E-02	1.60	3.02E-05
Phvul.009G143700	PTHR23176:SF42 - RHO GTPASE-ACTIVATING PROTEIN REN1 (1 of 1)	1.08	3.02E-01	1.32	7.34E-02	1.06	3.45E-01	1.68	2.46E-02
Phvul.003G268800	PTHR24095:SF176 - ACYL-ACTIVATING ENZYME 2-RELATED (1 of 2)	1.08	2.46E-01	1.58	1.98E-02	1.02	3.20E-01	1.56	2.72E-02
Phvul.010G076500	PTHR11260//PTHR11260:SF290 - GLUTATHIONE S-TRANSFERASE, GST, SUPERFAMILY, GST DOMAIN CONTAINING // SUE	1.07	2.02E-01	1.97	5.30E-03	1.28	1.27E-01	2.35	1.08E-03
Phvul.004G100300	KOG0472 - Leucine-rich repeat protein (1 of 95)	1.07	1.72E-01	1.17	3.09E-02	0.39	7.71E-01	0.86	1.48E-01
Phvul.009G066980	PTHR31729:SF0 - ETHYLENE-RESPONSIVE TRANSCRIPTION FACTOR ERF008-RELATED (1 of 4)	1.06	7.49E-03	0.79	2.74E-02	1.13	4.87E-03	0.81	2.84E-02
Phvul.001G196000	PTHR22849:SF38 - E3 UBIQUITIN-PROTEIN LIGASE PUB22-RELATED (1 of 2)	1.06	1.20E-02	1.06	3.78E-03	1.07	1.65E-02	0.90	2.27E-02
Phvul.008G201700	PF00234 - Protease inhibitor/seed storage/LTP family (Tryp alpha amyl) (1 of 24)	1.06	4.77E-02	0.67	1.54E-01	0.89	1.54E-01	0.40	4.40E-01
Phvul.007G030000	PTHR27009:SF21 - PR5-LIKE RECEPTOR KINASE-RELATED (1 of 19)	1.06	7.57E-02	1.68	2.53E-04	0.97	1.46E-01	1.38	4.69E-03
Phvul.004G002700	PTHR11206:SF87 - MATE EFFLUX FAMILY PROTEIN-RELATED (1 of 5)	1.05	5.32E-03	0.99	2.88E-03	0.74	1.19E-01	0.99	4.25E-03
Phvul.003G020200	PTHR10209//PTHR10209:SF148 - OXIDOREDUCTASE, 2OG-FE II OXYGENASE FAMILY PROTEIN // SUBFAMILY NOT NAME	1.05	1.92E-01	1.18	5.48E-02	0.97	2.76E-01	1.47	1.85E-02
Phvul.005G088600	PF09353 - Domain of unknown function (DUF1995) (DUF1995) (1 of 5)	1.04	1.57E-02	1.26	6.91E-04	1.18	6.12E-03	1.35	3.84E-04
Phvul.006G156800	no data	1.04	1.57E-02	1.01	6.04E-03	0.88	7.65E-02	0.79	4.39E-02
Phvul.003G251000	PTHR10891:SF568 - CALCIUM-BINDING PROTEIN CML41-RELATED (1 of 2)	1.04	1.44E-01	1.40	9.36E-03	0.65	4.80E-01	1.31	2.04E-02
Phvul.006G107900	PTHR22835:SF160 - GDSL ESTERASE/LIPASE LTL1 (1 of 6)	1.04	4.69E-02	1.07	1.31E-02	1.10	4.63E-02	1.00	2.92E-02
Phvul.009G159300	PTHR11638:SF118 - CHAPERONE PROTEIN CLPD, CHLOROPLASTIC (1 of 1)	1.04	2.01E-01	1.57	9.34E-03	0.93	3.14E-01	1.53	1.48E-02
Phvul.008G216700	K05933 - aminocyclopropanecarboxylate oxidase (E1.14.17.4) (1 of 9)	1.03	3.01E-02	1.55	6.42E-05	0.73	2.21E-01	1.32	1.22E-03
Phvul.007G074900	PTHR22814:SF129 - COPPER TRANSPORT FAMILY PROTEIN-RELATED (1 of 9)	1.03	1.59E-02	0.93	1.32E-02	1.07	1.67E-02	1.15	2.72E-03
Phvul.003G062800	PTHR11850//PTHR11850:SF101 - HOMEOBOX PROTEIN TRANSCRIPTION FACTORS // SUBFAMILY NOT NAMED (1 of 3)	1.03	1.49E-01	1.37	1.42E-02	1.00	2.11E-01	0.90	1.58E-01
Phvul.001G124800	PTHR10108//PTHR10108:SF692 - METHYLTRANSFERASE // SUBFAMILY NOT NAMED (1 of 3)	1.03	1.69E-01	1.53	6.74E-03	1.07	1.86E-01	2.32	6.28E-05
Phvul.003G108200	PTHR22904:SF169 - RNA POLYMERASE II-ASSOCIATED PROTEIN 3 (1 of 1)	1.03	1.05E-01	1.25	1.27E-02	0.80	2.96E-01	1.08	4.25E-02
Phvul.008G277226	PF08263 - Leucine rich repeat N-terminal domain (LRRNT 2) (1 of 284)	1.03	1.00E-01	1.54	1.12E-03	0.44	6.27E-01	1.46	3.29E-03
Phvul.004G138600	PF02893 - GRAM domain (GRAM) (1 of 18)	1.02	4.17E-01	1.83	3.34E-02	1.33	2.91E-01	2.11	1.89E-02
Phvul.009G001800	K14488 - SAUR family protein (SAUR) (1 of 75)	1.02	1.72E-01	2.39	3.71E-05	1.12	1.52E-01	2.41	4.03E-05
Phvul.001G194700	PTHR31415:SF4 - HARPIN-INDUCED PROTEIN-LIKE-RELATED (1 of 9)	1.02	1.03E-01	1.84	9.11E-05	1.33	2.55E-02	1.78	2.39E-04
Phvul.006G181300	PTHR10168:SF69 - GLUTAREDOXIN-C9 (1 of 2)	1.02	2.90E-01	2.23	8.05E-04	1.17	2.44E-01	2.44	2.61E-04
Phvul.002G054100	PTHR11926//PTHR11926:SF190 - GLUCOSYL/GLUCURONOSYL TRANSFERASES // SUBFAMILY NOT NAMED (1 of 3)	1.02	8.76E-02	1.48	1.45E-03	1.55	4.47E-03	1.34	6.29E-03
Phvul.003G168800	PTHR31268:SF5 - GALACTINOL--SUCROSE GALACTOSYLTRANSFERASE 6-RELATED (1 of 1)	1.02	1.17E-01	0.65	2.47E-01	1.25	4.88E-02	0.93	8.65E-02
Phvul.008G127500	PTHR15898:SF1 - GLUCOSE-INDUCED DEGRADATION PROTEIN 4 HOMOLOG (1 of 1)	1.01	7.54E-02	1.27	4.09E-03	0.82	2.26E-01	0.88	6.61E-02
Phvul.009G136000	PTHR10219:SF34 - GENOMIC DNA, CHROMOSOME 3, P1 CLONE: MXL8 (1 of 2)	1.01	1.55E-02	0.76	3.94E-02	0.57	3.16E-01	0.57	1.51E-01
Phvul.010G145600	PTHR19241:SF246 - ABC TRANSPORTER G FAMILY MEMBER 29 (1 of 2)	1.01	7.55E-02	1.17	9.92E-03	0.70	3.15E-01	0.91	6.14E-02
Phvul.007G069600	PTHR10106//PTHR10106:SF17 - CYTOCHROME B561-RELATED // SUBFAMILY NOT NAMED (1 of 2)	1.01	4.85E-03	0.84	9.79E-03	0.72	9.93E-02	0.51	1.53E-01
Phvul.L009843	PTHR24089:SF316 - ADP,ATP CARRIER PROTEIN 1, MITOCHONDRIAL (1 of 3)	1.01	2.32E-02	1.32	2.47E-04	0.86	9.16E-02	1.41	1.08E-04
Phvul.002G066300	PTHR13620:SF7 - EMB (1 of 2)	1.01	3.09E-02	0.79	5.44E-02	0.91	8.25E-02	0.80	5.52E-02
Phvul.002G152400	PTHR10795//PTHR10795:SF370 - PROPROTEIN CONVERTASE SUBTILISIN/KEXIN // SUBFAMILY NOT NAMED (1 of 2)	1.01	4.27E-01	3.08	4.67E-05	-0.52	8.18E-01	2.72	8.40E-04
Phvul.007G168000	PTHR27001:SF54 - RECEPTOR-LIKE CYTOPLASMIC KINASE VIA5 (1 of 2)	1.00	2.05E-01	1.36	1.66E-02	0.89	3.20E-01	1.54	8.85E-03
Phvul.007G075000	PTHR22814:SF129 - COPPER TRANSPORT FAMILY PROTEIN-RELATED (1 of 9)	1.00	1.79E-02	1.06	3.47E-03	0.97	3.22E-02	1.12	2.91E-03
Phvul.009G239700	2.7.11.1 - Non-specific serine/threonine protein kinase / Threonine-specific protein kinase (1 of 1198)	1.00	9.48E-02	1.31	4.99E-03	0.78	2.81E-01	1.27	9.93E-03
Phvul.010G009200	no data	1.00	1.73E-01	1.16	2.51E-02	1.44	3.23E-02	1.27	1.66E-02
Phvul.005G181800	PTHR31429:SF3 - WRKY TRANSCRIPTION FACTOR 40-RELATED (1 of 4)	1.00	7.37E-03	1.11	6.50E-04	1.09	3.74E-03	1.06	1.83E-03
Phvul.007G152700	2.7.11.17 - Calcium/calmodulin-dependent protein kinase / Microtubule-associated protein 2 kinase (1 of 14)	1.00	6.19E-02	1.22	3.46E-03	0.88	1.50E-01	0.98	2.80E-02
Phvul.002G292900	PTHR11751:SF380 - 1-AMINOCYCLOPROPANE-1-CARBOXYLATE SYNTHASE 2-RELATED (1 of 2)	1.00	1.71E-01	1.16	2.95E-02	0.77	3.74E-01	0.80	1.63E-01
Phvul.008G088800	PTHR31087:SF11 - PROTEIN LURP-ONE-RELATED 1-RELATED (1 of 9)	0.99	1.09E-01	1.53	1.56E-03	0.79	2.81E-01	0.94	7.22E-02
Phvul.006G156000	K15813 - beta-amyrin synthase (LUP4) (1 of 8)	0.99	1.84E-01	1.43	1.00E-02	0.82	3.44E-01	1.06	7.72E-02
Phvul.003G213300	PF03634 - TCP family transcription factor (TCP) (1 of 27)	0.99	6.77E-02	1.01	1.88E-02	1.00	8.08E-02	1.01	2.51E-02
Phvul.010G019300	no data	0.99	1.41E-02	0.65	6.74E-02	0.76	1.12E-01	0.45	2.44E-01
Phvul.001G263800	PF01823 - MAC/Perforin domain (MACPF) (1 of 9)	0.99	1.71E-01	1.32	1.51E-02	0.95	2.42E-01	1.40	1.46E-02
Phvul.010G102000	PF02458 - Transferase family (Transferase) (1 of 94)	0.99	1.63E-02	1.98	5.31E-09	0.93	4.00E-02	1.14	2.23E-03
Phvul.003G120400	PTHR19241//PTHR19241:SF289 - ATP-BINDING CASSETTE TRANSPORTER // SUBFAMILY NOT NAMED (1 of 1)	0.99	1.10E-01	1.12	2.19E-02	0.70	3.52E-01	0.89	8.77E-02
Phvul.005G078800	KOG4579 - Leucine-rich repeat (LRR) protein associated with apoptosis in muscle tissue (1 of 14)	0.98	1.98E-02	0.67	6.33E-02	0.70	1.98E-01	0.61	1.05E-01
Phvul.001G000800	PTHR22893//PTHR22893:SF62 - NADH OXIDOREDUCTASE-RELATED // SUBFAMILY NOT NAMED (1 of 2)	0.98	1.58E-01	1.03	4.90E-02	0.98	1.91E-01	1.01	6.17E-02
Phvul.008G054300	PTHR31126:SF19 - ATYPICAL DUAL-SPECIFICITY PHOSPHATASE (1 of 2)	0.98	7.98E-02	1.53	5.03E-04	0.99	9.81E-02	1.50	9.84E-04
Phvul.002G026900	K12356 - coniferyl-alcohol glucosyltransferase (UGT72E) (1 of 3)	0.98	3.39E-03	0.57	6.68E-02	1.15	4.36E-04	0.42	2.15E-01
Phvul.L006743	PTHR31636:SF46 - SCARECROW-LIKE PROTEIN 11-RELATED (1 of 2)	0.98	2.52E-02	1.24	6.73E-04	1.10	1.33E-02	1.05	6.36E-03
Phvul.008G108500	PTHR13620 - 3-5 EXONUCLEASE (1 of 10)	0.98	1.78E-01	1.29	1.53E-02	0.70	4.29E-01	1.29	2.22E-02
Phvul.007G136300	PTHR22849//PTHR22849:SF31 - WDSAM1 PROTEIN // SUBFAMILY NOT NAMED (1 of 1)	0.98	1.12E-01	1.30	3.83E-03	0.77	2.96E-01	1.17	1.46E-02
Phvul.008G078400	PTHR31852:SF3 - LATE EMBRYOGENESIS ABUNDANT (LEA) HYDROXYPROLINE-RICH GLYCOPROTEIN FAMILY (1 of 1)	0.98	7.05E-03	1.14	2.82E-04	0.84	3.95E-02	0.93	5.18E-03
Phvul.004G056700	PTHR22950//PTHR22950:SF298 - AMINO ACID TRANSPORTER // SUBFAMILY NOT NAMED (1 of 6)	0.97	1.44E-01	1.05	4.12E-02	0.88	2.47E-01	0.67	2.42E-01
Phvul.001G245300	PF07714//PF08263 - Protein tyrosine kinase (Pkinase Tyr) // Leucine rich repeat N-terminal domain (LRRNT 2) (1 of 13)	0.96	3.02E-02	1.08	3.56E-03	0.62	2.87E-01	0.72	7.40E-02
Phvul.010G016600	PTHR11732:SF223 - ALDO-KETO REDUCTASE FAMILY 4 MEMBER C10 (1 of 9)	0.96	7.84E-02	1.10	1.13E-02	1.16	3.48E-02	0.75	1.12E-01
Phvul.002G277400	PTHR18959 - CALPONIN/TRANSGELIN (1 of 1)	0.96	1.85E-02	0.90	8.91E-03	0.90	4.34E-02	0.87	1.60E-02
Phvul.004G092100	K08912 - light-harvesting complex II chlorophyll a/b binding protein 1 (LHCB1) (1 of 13)	0.96	4.27E-01	2.08	1.14E-02	0.84	5.39E-01	2.50	2.39E-03
Phvul.005G063900	2.1.1.14 - 5-methyltetrahydropteroyltriglutamate--homocysteine S-methyltransferase / Tetrahydropteroylglutamate-ho	0.95	7.66E-02	1.22	3.61E-03	0.89	1.38E-01	0.94	3.47E-02
Phvul.004G136500	PF00069//PF00560//PF08263//PF13855 - Protein kinase domain (Pkinase) // Leucine Rich Repeat (LRR 1) // Leucine ric	0.95	6.86E-02	0.99	1.55E-02	0.77	2.11E-01	1.10	9.52E-03
Phvul.002G315400	PTHR17224//PTHR17224:SF5 - PEPTIDYL-TRNA HYDROLASE // SUBFAMILY NOT NAMED (1 of 1)	0.95	2.12E-01	1.43	7.52E-03	0.91	2.75E-01	1.13	4.85E-02

Phvul.011G183900	PF00407 - Pathogenesis-related protein Bet v I family (Bet v 1)	0.95	5.62E-02	0.94	2.09E-02	0.73	2.20E-01	0.97	2.33E-02
Phvul.011G014450	PTHR23155:SF402 - DISEASE RESISTANCE PROTEIN RPP13-RELATED (1 of 8)	0.95	1.45E-02	1.03	1.71E-03	1.01	1.13E-02	0.93	7.53E-03
Phvul.001G216000	1.1.1.145 - 3-beta-hydroxy-Delta(5)-steroid dehydrogenase / Progesterone reductase (1 of 3)	0.95	1.70E-01	1.14	2.57E-02	0.61	4.88E-01	0.59	3.09E-01
Phvul.010G024100	PF00931//PF01582 - NB-ARC domain (NB-ARC) // TIR domain (TIR) (1 of 28)	0.95	8.43E-03	0.70	2.90E-02	0.64	1.60E-01	0.43	2.22E-01
Phvul.004G155000	K04730 - interleukin-1 receptor-associated kinase 1 (IRAK1) (1 of 24)	0.94	1.52E-01	0.99	4.81E-02	0.94	1.86E-01	0.50	3.84E-01
Phvul.008G140113	PF03007 - Wax ester synthase-like Acyl-CoA acyltransferase domain (WES acyltransf) (1 of 8)	0.94	5.75E-01	2.54	8.70E-03	1.22	4.79E-01	3.02	2.53E-03
Phvul.004G107001	PF14111 - Domain of unknown function (DUF4283) (DUF4283) (1 of 81)	0.94	1.79E-02	1.06	1.62E-03	1.07	7.11E-03	1.31	1.12E-04
Phvul.001G089200	PF03004 - Plant transposase (Ptta/En/Spm family) (Transposase 24) (1 of 17)	0.94	7.03E-02	0.91	3.21E-02	0.75	2.24E-01	0.79	7.74E-02
Phvul.006G022800	PTHR13683:SF250 - ASPARTYL PROTEASE-LIKE PROTEIN (1 of 3)	0.94	1.76E-01	1.59	1.75E-03	0.55	5.54E-01	1.59	2.68E-03
Phvul.008G249500	1.11.1.7 - Peroxidase / Lactoperoxidase (1 of 96)	0.94	6.18E-02	1.07	7.54E-03	0.65	2.96E-01	0.64	1.51E-01
Phvul.005G026800	no data	0.94	1.35E-02	1.30	6.15E-05	0.87	3.63E-02	1.07	1.76E-03
Phvul.011G183766	PF00407 - Pathogenesis-related protein Bet v I family (Bet v 1)	0.94	1.02E-01	0.86	6.71E-02	0.78	2.45E-01	1.03	2.97E-02
Phvul.009G003400	PTHR10209:SF107 - 2-OXOGLUTARATE (2OG) AND FE(II)-DEPENDENT OXYGENASE-LIKE PROTEIN-RELATED (1 of 2)	0.93	2.96E-01	1.50	1.11E-02	1.02	2.85E-01	1.34	3.11E-02
Phvul.001G047500	no data	0.93	2.16E-01	-1.32	3.80E-02	-0.50	6.48E-01	-0.37	6.17E-01
Phvul.001G066550	PF00069//PF07645//PF13947 - Protein kinase domain (Pkinase) // Calcium-binding EGF domain (EGF CA) // Wall-associ	0.93	2.32E-01	1.15	4.10E-02	0.86	3.25E-01	0.91	1.31E-01
Phvul.001G021700	no data	0.92	1.42E-01	0.96	4.81E-02	1.07	9.19E-02	1.22	1.26E-02
Phvul.L000770	no data	0.92	5.27E-01	1.92	5.05E-02	1.34	3.45E-01	2.26	2.27E-02
Phvul.001G116900	K12857 - Prp8 binding protein (SNRNP40, PRP8BP) (1 of 1)	0.92	5.62E-02	2.01	4.61E-08	0.68	2.63E-01	1.90	4.18E-07
Phvul.002G022100	PTHR10992:SF784 - ALPHA-HYDROXYNITRILE LYASE-RELATED (1 of 2)	0.92	1.42E-02	1.52	1.02E-06	1.03	6.12E-03	1.20	2.01E-04
Phvul.011G014500	PTHR23155:SF402 - DISEASE RESISTANCE PROTEIN RPP13-RELATED (1 of 8)	0.92	1.00E-01	1.24	3.83E-03	0.94	1.12E-01	1.43	1.11E-03
Phvul.007G030300	PTHR27009:SF21 - PR5-LIKE RECEPTOR KINASE-RELATED (1 of 19)	0.92	1.36E-01	1.54	9.44E-04	0.62	4.38E-01	1.78	6.81E-05
Phvul.007G212600	PTHR15486:SF18 - GLYCEROL-3-PHOSPHATE ACYLTRANSFERASE 2-RELATED (1 of 3)	0.92	3.69E-01	1.60	2.56E-02	1.15	2.60E-01	1.44	5.76E-02
Phvul.002G265100	no data	0.92	3.98E-01	1.16	7.99E-02	0.67	6.28E-01	1.42	2.89E-02
Phvul.008G277268	PF08263 - Leucine rich repeat N-terminal domain (LRRNT 2) (1 of 284)	0.91	5.92E-02	1.60	1.82E-05	0.81	1.43E-01	1.58	3.75E-05
Phvul.009G207300	KOG1947//KOG4341 - Leucine rich repeat proteins, some proteins contain F-box // F-box protein containing LRR (1 of 1)	0.91	4.68E-02	0.84	3.04E-02	0.87	8.91E-02	0.96	1.69E-02
Phvul.002G290000	6.3.5.2 - GMP synthase (glutamine-hydrolyzing) / GMP synthetase (glutamine-hydrolyzing) (1 of 1)	0.91	9.08E-02	1.24	2.81E-03	0.38	6.29E-01	0.80	7.64E-02
Phvul.008G277000	PF00560//PF13855 - Leucine Rich Repeat (LRR 1) // Leucine rich repeat (LRR 8) (1 of 35)	0.91	1.65E-01	1.41	3.15E-03	0.63	4.35E-01	1.27	1.18E-02
Phvul.008G032551	2.3.1.115 - Isoflavone-7-O-beta-glucoside 6''-O-malonyltransferase / Flavone/flavonol 7-O-beta-D-glucoside malonyltran	0.91	5.72E-01	-2.65	2.95E-02	0.04	9.90E-01	-2.10	7.84E-02
Phvul.004G020300	PTHR14677:SF2 - AN1-TYPE ZINC FINGER PROTEIN 1 (1 of 2)	0.91	4.74E-02	0.94	1.24E-02	1.00	3.19E-02	0.62	1.31E-01
Phvul.001G243500	PTHR27003:SF102 - SERINE/THREONINE-PROTEIN KINASE-LIKE PROTEIN CCR3-RELATED (1 of 6)	0.91	3.23E-01	1.86	5.06E-03	0.78	4.66E-01	1.79	1.03E-02
Phvul.008G210400	PF04782 - Protein of unknown function (DUF632) (DUF632) (1 of 15)	0.91	8.96E-02	1.26	2.12E-03	0.67	3.03E-01	1.08	1.35E-02
Phvul.008G209900	K00423 - L-ascorbate oxidase (E1.10.3.3) (1 of 3)	0.91	9.81E-02	1.05	1.53E-02	0.81	1.94E-01	1.06	1.90E-02
Phvul.005G173400	K07767 - katanin p60 ATPase-containing subunit A1 [EC:3.6.4.3] (KATNA1) (1 of 3)	0.91	2.98E-01	1.17	3.75E-02	0.62	5.82E-01	0.96	1.06E-01
Phvul.003G019700	PF07676 - WD40-like Beta Propeller Repeat (PD40) (1 of 5)	0.91	7.98E-02	0.60	1.87E-01	1.26	8.63E-03	0.96	2.83E-02
Phvul.007G041000	PF04450 - Peptidase of plants and bacteria (BSP) (1 of 3)	0.91	2.07E-03	1.05	6.96E-05	0.84	8.24E-03	0.87	1.86E-03
Phvul.006G137100	PTHR23324//PTHR23324:SF79 - SEC14 RELATED PROTEIN // SUBFAMILY NOT NAMED (1 of 4)	0.91	2.49E-02	1.02	2.60E-03	0.59	2.57E-01	0.81	2.68E-02
Phvul.002G258600	PTHR34937:SF1 - MYOSIN HEAVY CHAIN-RELATED (1 of 1)	0.90	2.05E-01	1.26	1.46E-02	0.82	3.05E-01	1.63	2.06E-03
Phvul.007G082700	1.11.1.7 - Peroxidase / Lactoperoxidase (1 of 96)	0.90	1.79E-02	1.22	1.33E-04	0.65	1.64E-01	0.67	5.67E-02
Phvul.003G268500	PTHR22595:SF34 - BASIC ENDOCHITINASE B (1 of 8)	0.89	2.28E-01	1.26	1.88E-02	0.92	2.54E-01	1.00	8.32E-02
Phvul.006G111700	no data	0.89	2.26E-02	1.04	1.50E-03	1.09	3.74E-03	1.27	1.07E-04
Phvul.009G072900	K16296 - serine carboxypeptidase-like clade I [EC:3.4.16.-] (SCPL-I) (1 of 6)	0.89	5.50E-02	0.79	3.95E-02	0.81	1.11E-01	0.81	3.76E-02
Phvul.007G074800	PTHR22814:SF129 - COPPER TRANSPORT PROTEIN FAMILY PROTEIN-RELATED (1 of 9)	0.89	1.02E-01	1.11	8.91E-03	0.95	9.16E-02	1.35	2.00E-03
Phvul.011G189500	KOG2615 - Permease of the major facilitator superfamily (1 of 8)	0.88	1.07E-01	1.11	9.92E-03	0.30	7.23E-01	0.78	9.52E-02
Phvul.005G040750	K18999 - RNA polymerase II C-terminal domain phosphatase-like 3/4 [EC:3.1.3.16] (CPL3 4) (1 of 3)	0.88	2.14E-01	1.20	1.96E-02	0.80	3.19E-01	1.42	7.01E-03
Phvul.001G068700	3.1.4.46 - Glycerophosphodiester phosphodiesterase / Glycerophosphoryl diester phosphodiesterase (1 of 65)	0.88	1.38E-01	1.28	1.15E-03	0.78	2.64E-01	0.88	4.13E-02
Phvul.011G143600	no data	0.88	7.67E-02	0.80	4.74E-02	0.64	2.96E-01	0.56	2.00E-01
Phvul.001G199800	PF00917 - MATH domain (MATH) (1 of 11)	0.88	9.45E-02	1.32	8.58E-04	0.85	1.42E-01	1.07	1.10E-02
Phvul.011G183700	PF00407 - Pathogenesis-related protein Bet v I family (Bet v 1)	0.88	4.05E-02	0.86	1.79E-02	0.70	1.66E-01	0.83	2.80E-02
Phvul.009G029200	PTHR10174//PTHR10174:SF148 - RETINALDEHYDE BINDING PROTEIN-RELATED // SUBFAMILY NOT NAMED (1 of 3)	0.88	6.42E-02	1.49	6.57E-05	0.75	1.68E-01	1.10	6.06E-03
Phvul.005G156800	K15718 - linoleate 9S-lipoxygenase (LOX1 5) (1 of 19)	0.88	6.18E-01	-2.52	2.72E-02	0.19	9.46E-01	-1.01	4.37E-01
Phvul.003G030000	PTHR31793:SF5 - THIOESTERASE-LIKE PROTEIN (1 of 1)	0.88	3.04E-01	1.61	3.93E-03	1.11	1.83E-01	1.72	3.18E-03
Phvul.010G075400	PTHR31100:SF2 - AT-HOOK MOTIF NUCLEAR LOCALIZED PROTEIN 18-RELATED (1 of 1)	0.87	2.10E-01	1.23	1.67E-02	0.66	4.27E-01	1.25	2.14E-02
Phvul.002G158600	no data	0.87	2.05E-02	1.12	3.53E-04	0.73	9.62E-02	0.80	1.97E-02
Phvul.005G054000	PTHR11260:SF204 - GLUTATHIONE S-TRANSFERASE U21-RELATED (1 of 4)	0.87	6.11E-01	2.95	2.82E-02	1.52	3.30E-01	3.86	3.19E-03
Phvul.008G052500	K08057 - calreticulin (CALR) (1 of 3)	0.87	7.05E-03	1.19	2.12E-05	0.81	2.17E-02	1.29	3.71E-06
Phvul.011G002500	1.6.5.5 - NADPH:quinone reductase / Zeta-crystallin (1 of 8)	0.87	1.91E-02	1.27	3.71E-05	0.69	1.16E-01	1.10	6.48E-04
Phvul.003G009200	PF00067 - Cytochrome P450 (p450) (1 of 273)	0.87	4.99E-01	2.79	2.69E-04	0.61	7.00E-01	2.63	9.41E-04
Phvul.002G002600	PTHR27001:SF75 - PROTEIN KINASE FAMILY PROTEIN (1 of 1)	0.87	2.10E-01	1.94	1.05E-04	0.75	3.43E-01	1.75	7.67E-04
Phvul.003G285600	PF05678 - VQ motif (VQ) (1 of 39)	0.86	2.33E-01	1.08	4.13E-02	0.81	3.13E-01	1.30	1.55E-02
Phvul.006G183100	PF00847 - AP2 domain (AP2) (1 of 180)	0.86	1.59E-02	1.05	5.42E-04	0.75	6.43E-02	0.57	9.01E-02
Phvul.003G113800	K10578 - ubiquitin-conjugating enzyme E2 J1 (UBE2J1, NCUBE1, UBC6) (1 of 2)	0.86	2.88E-02	0.94	4.02E-03	0.60	2.34E-01	1.04	2.09E-03
Phvul.009G262900	PTHR11771:SF38 - LIPOXYGENASE 3, CHLOROPLASTIC-RELATED (1 of 2)	0.86	1.27E-01	1.20	4.91E-03	0.70	2.92E-01	1.04	2.11E-02
Phvul.007G217800	PTHR31190:SF27 - ETHYLENE-RESPONSIVE TRANSCRIPTION FACTOR CRF1-RELATED (1 of 1)	0.86	3.50E-01	1.26	5.03E-02	0.78	4.55E-01	1.81	4.09E-03
Phvul.007G159000	PTHR26374:SF198 - ZINC FINGER PROTEIN ZAT11 (1 of 4)	0.86	3.89E-01	1.28	5.98E-02	1.25	1.90E-01	1.61	1.89E-02
Phvul.008G262200	PTHR30546:SF11 - 1,4-BENZOQUINONE REDUCTASE-LIKE (1 of 1)	0.86	8.70E-02	0.81	4.45E-02	0.77	1.67E-01	0.83	4.75E-02
Phvul.005G063300	PF00076 - RNA recognition motif. (a.k.a. RRM, RBD, or RNP domain) (RRM 1) (1 of 252)	0.86	7.00E-02	1.11	2.81E-03	0.76	1.56E-01	0.98	1.33E-02
Phvul.008G182300	PTHR31642:SF12 - PROTEIN ECERIFERUM 1 (1 of 1)	0.86	1.10E-01	1.22	2.75E-03	0.99	6.52E-02	1.04	1.55E-02
Phvul.011G081800	2.7.11.1 - Non-specific serine/threonine protein kinase / Threonine-specific protein kinase (1 of 1198)	0.86	6.07E-01	1.73	1.62E-02	-1.10	6.59E-01	1.17	1.52E-01
Phvul.002G201800	PTHR27000:SF136 - POLYGALACTURONASE INHIBITOR 1-RELATED (1 of 6)	0.85	4.70E-03	1.01	2.20E-04	0.69	4.96E-02	0.87	2.75E-03
Phvul.001G069232	K04733 - interleukin-1 receptor-associated kinase 4 (IRAK4) (1 of 48)	0.85	4.70E-01	1.20	1.31E-01	0.26	8.93E-01	1.88	8.78E-03
Phvul.001G064700	PTHR31636:SF10 - SCARECROW-LIKE PROTEIN 1 (1 of 2)	0.85	1.13E-03	1.09	4.46E-06	0.78	5.77E-03	1.18	6.88E-07
Phvul.010G068300	PTHR11730:SF38 - AMMONIUM TRANSPORTER 2 (1 of 3)	0.85	4.01E-02	0.61	9.56E-02	0.72	1.34E-01	0.72	5.04E-02
Phvul.004G143600	K01177 - beta-amylase (E3.2.1.2) (1 of 5)	0.85	6.16E-02	0.70	6.95E-02	0.84	8.23E-02	0.92	1.86E-02
Phvul.006G142300	PTHR23130:SF80 - AUXIN-INDUCED IN ROOT CULTURES PROTEIN 12 (1 of 3)	0.85	4.61E-02	1.38	5.42E-05	0.91	3.76E-02	1.44	3.37E-05
Phvul.006G204200	4.1.99.12 - 3,4-dihydroxy-2-butanone-4-phosphate synthase / L-3,4-dihydroxybutan-2-one-4-phosphate synthase (1 of 3	0.84	1.38E-01	1.41	9.53E-04	0.88	1.49E-01	1.60	2.17E-04
Phvul.010G102700	PF13962 - Domain of unknown function (PGG) (1 of 69)	0.84	5.25E-01	1.46	1.36E-01	0.45	8.04E-01	2.04	3.05E-02
Phvul.009G233700	PTHR23155:SF402 - DISEASE RESISTANCE PROTEIN RPP13-RELATED (1 of 8)	0.84	3.76E-02	1.27	6.95E-05	0.82	5.69E-02	1.35	2.94E-05
Phvul.008G081800	PF03106 - WRKY DNA -binding domain (WRKY) (1 of 91)	0.84	2.42E-01	1.87	1.94E-04	1.03	1.53E-01	1.69	1.26E-03
Phvul.009G243800	PTHR22765:SF56 - VACUOLAR-SORTING RECEPTOR 5-RELATED (1 of 1)	0.83	5.31E-02	1.20	4.65E-04	0.89	4.50E-02	1.20	6.56E-04
Phvul.003G149600	PTHR18966:SF204 - GLUTAMATE RECEPTOR 2.5-RELATED (1 of 3)	0.83	5.59E-01	2.03	1.98E-02	1.55	1.87E-01	2.40	5.61E-03
Phvul.009G044600	PTHR10209:SF111 - DOWNY MILDEW RESISTANCE 6 PROTEIN / OXIDOREDUCTASE (1 of 2)	0.83	3.47E-01	2.08	2.12E-04	1.31	1.05E-01	1.53	1.19E-02
Phvul.001G222900	2.3.3.8 - ATP citrate synthase / Citric cleavage enzyme (1 of 5)	0.83	1.11E-01	0.92	2.57E-02	0.49	4.60E-01	0.56	2.14E-01
Phvul.001G068600	PF01535//PF12854//PF13041 - PPR repeat (PPR) // PPR repeat (PPR 1) // PPR repeat family (PPR 2) (1 of 29)	0.83	1.08E-01	0.92	2.02E-02	1.01	4.62E-02	0.80	5.66E-02
Phvul.011G183832	PF00407 - Pathogenesis-related protein Bet v I family (Bet v 1)	0.83	8.87E-02	0.89	2.25E-02	0.71	2.08E-01	0.92	2.39E-02
Phvul.002G056100	no data	0.83	1.78E-01	1.80	3.46E-05	0.76	2.69E-01	1.76	7.90E-05
Phvul.009G116000	PTHR10183//PTHR10183:SF323 - CALPAIN // SUBFAMILY NOT NAMED (1 of 1)	0.83	1.34E-03	0.56	2.12E-02	0.48	1.64E-01	0.02	9.60E-01
Phvul.002G156400	PTHR22950:SF269 - EXPRESSED PROTEIN (1 of 5)	0.83	2.26E-02	0.28	4.53E-01	0.33	5.39E-01	-0.11	7.94E-01
Phvul.008G193900	K12870 - pre-mRNA-splicing factor ISY1 (ISY1) (1 of 2)	0.82	2.69E-01	1.21	1.82E-02	0.61	4.96E-01	1.27	1.68E-02
Phvul.002G049500	PF01657//PF07714 - Salt stress response/antifungal (Stress-antifung) // Protein tyrosine kinase (Pkinase Tyr) (1 of 40)	0.82	3.98E-02	1.11	6.51E-04	0.72	1.10E-01	1.18	3.74E-04
Phvul.009G145200	no data	0.82	4.77E-02	0.33	4.08E-01	0.93	2.44E-02	0.51	1.63E-01
Phvul.002G064500	PF00646 - F-box domain (F-box) (1 of 135)	0.82	9.08E-03	0.74	7.52E-03	0.81	1.39E-02	0.63	3.30E-02
Phvul.009G240622	PF13637//PF13962 - Ankyrin repeats (many copies) (Ank 4) // Domain of unknown function (PGG) (1 of 3)	0.82	2.73E-01	1.58	2.36E-03	0.55	5.54E-01	1.09	5.22E-02
Phvul.007G108500	PTHR10641//PTHR10641:SF650 - MYB-LIKE DNA-BINDING PROTEIN MYB // SUBFAMILY NOT NAMED (1 of 2)	0.82	3.29E-01	1.15	5.22E-02	0.66	4.98E-01	1.45	1.65E-02
Phvul.010G040900	PTHR11566:SF80 - DYNAMIN-RELATED PROTEIN 1C (1 of 3)	0.81	2.00E-01	1.84	2.85E-05	0.98	1.29E-01	1.71	1.48E-04
Phvul.005G030900	PF00954//PF01453//PF07714//PF08276 - S-locus glycoprotein domain (S locus glycop) // D-mannose binding lectin (B le	0.81	5.86E-01	2.03	1.50E-02	1.13	4.28E-01	1.73	5.54E-02
Phvul.008G264500	PTHR32285:SF47 - PROTEIN TRICHOME BIREFRINGENCE-LIKE 43 (1 of 2)	0.81	3.46E-02	0.72	2.87E-02	0.76	7.33E-02	0.67	5.07E-02
Phvul.003G020300	PTHR10209//PTHR10209:SF148 - OXIDOREDUCTASE, 2OG-FE II OXYGENASE FAMILY PROTEIN // SUBFAMILY NOT NAME	0.81	1.34E-01	0.94	2.76E-02	0.75	2.20E-01	0.94	3.16E-02
Phvul.001G124700	PTHR10108//PTHR10108:SF692 - METHYLTRANSFERASE // SUBFAMILY NOT NAMED (1 of 3)	0.81	3.84E-01	1.49	2.47E-02	0.51	6.66E-01	2.42	1.41E-04
Phvul.007G169400	no data	0.81	1.11E-02	0.29	4.13E-01	0.50	2.42E-01	-0.01	9.88E-01
Phvul.001G228500	PTHR22849:SF43 - U-BOX DOMAIN-CONTAINING PROTEIN 54-RELATED (1 of 2)	0.81	3.48E-01	1.42	1.61E-02	1.39	5.71E-02	1.34	3.23E-02
Phvul.002G210200	PTHR31279:SF12 - PHI-1-LIKE PROTEIN (1 of 2)	0.80	1.46E-01	1.16	5.97E-03	0.88	1.35E-01	1.19	6.56E-03
Phvul.002G047500	PTHR23024//PTHR23024:SF196 - MEMBER OF 'GDXG' FAMILY OF LIPOLYTIC ENZYMES // SUBFAMILY NOT NAMED (1 of	0.80	9.90E-02	1.17	1.85E-03	0.66	2.47E-01	1.19	2.71E-03
Phvul.003G139200	K13105 - proline-rich protein PRCC (PRCC) (1 of 1)	0.80	1.85E-02	1.02	3.44E-04	0.76	4.19E-02	0.88	3.27E-03

Phvul.011G127300	PTHR12411//PTHR12411:SF339 - CYSTEINE PROTEASE FAMILY C1-RELATED // SUBFAMILY NOT NAMED (1 of 5)	0.80	4.84E-01	1.66	3.15E-02	0.33	8.43E-01	1.61	4.41E-02
Phvul.011G167300	PTHR11177//PTHR11177:SF161 - CHITINASE // SUBFAMILY NOT NAMED (1 of 1)	0.80	1.36E-01	1.23	2.64E-03	0.82	1.60E-01	1.08	1.32E-02
Phvul.008G281000	PF00560 - Leucine Rich Repeat (LRR 1) (1 of 227)	0.80	3.08E-02	1.00	8.86E-04	0.54	2.60E-01	0.93	3.34E-03
Phvul.001G202700	no data	0.80	2.71E-01	1.07	3.75E-02	0.80	3.14E-01	1.23	2.02E-02
Phvul.003G030601	PTHR11220:SF25 - F3F9.4 (1 of 2)	0.80	2.56E-01	1.11	2.94E-02	0.92	2.07E-01	1.40	6.56E-03
Phvul.001G157900	K13407 - fatty acid omega-hydroxylase (CYP94A1) (1 of 3)	0.80	4.53E-01	1.37	6.08E-02	1.09	3.02E-01	2.15	3.00E-03
Phvul.008G057900	PTHR31459:SF3 - DESICCATION-RELATED PROTEIN LEA14-RELATED (1 of 1)	0.80	3.91E-02	0.54	1.12E-01	0.96	1.04E-02	0.76	2.39E-02
Phvul.001G241300	PF07014 - Hs1pro-1 protein C-terminus (Hs1pro-1 C) (1 of 2)	0.80	4.85E-02	0.94	4.31E-03	0.81	5.72E-02	1.03	2.51E-03
Phvul.008G156900	K15627 - tether containing UBX domain for GLUT4 (ASPSCR1, ASPL) (1 of 1)	0.79	5.68E-01	2.07	3.01E-02	1.12	4.04E-01	2.31	1.98E-02
Phvul.008G146000	no data	0.79	2.35E-01	1.51	1.39E-03	0.65	4.03E-01	1.24	1.40E-02
Phvul.009G250400	K19041 - E3 ubiquitin-protein ligase RNF38/44 [EC:6.3.2.19] (RNF38 44) (1 of 9)	0.79	9.81E-02	1.01	7.03E-03	1.21	3.95E-03	1.20	1.90E-03
Phvul.006G147800	PF03106 - WRKY DNA -binding domain (WRKY) (1 of 91)	0.79	7.11E-02	1.42	2.77E-05	1.01	1.67E-02	1.46	2.04E-05
Phvul.002G286500	PF00314 - Thaumatin family (Thaumatin) (1 of 26) Pathogenesis-related	0.79	4.56E-01	2.01	3.32E-03	0.51	7.00E-01	2.15	2.57E-03
Phvul.007G250000	PTHR11260:SF254 - GLUTATHIONE S-TRANSFERASE U8 (1 of 1)	0.79	1.26E-02	0.87	1.36E-03	0.74	3.26E-02	0.94	6.94E-04
Phvul.001G232100	PF14009 - Domain of unknown function (DUF4228) (DUF4228) (1 of 38)	0.79	3.16E-01	1.38	2.64E-03	1.03	1.71E-01	1.05	3.37E-02
Phvul.005G088218	PTHR23213:SF195 - FORMIN-LIKE PROTEIN 12-RELATED (1 of 3)	0.79	4.37E-03	0.93	2.08E-04	0.72	1.84E-02	0.76	3.83E-03
Phvul.002G054200	PF03007//PF06974 - Wax ester synthase-like Acyl-CoA acyltransferase domain (WES acyltransf) // Protein of unknown f	0.79	1.00E-02	0.85	1.41E-03	0.75	2.26E-02	0.78	5.28E-03
Phvul.003G112800	no data	0.79	2.09E-01	0.66	1.87E-01	1.13	5.40E-02	0.97	4.78E-02
Phvul.008G149800	PF00069//PF00560//PF08263//PF13855 - Protein kinase domain (Pkinase) // Leucine Rich Repeat (LRR 1) // Leucine ric	0.78	7.95E-03	0.97	1.30E-04	0.69	3.45E-02	0.73	6.89E-03
Phvul.004G059000	PTHR10666//PTHR10666:SF115 - UBIQUITIN // SUBFAMILY NOT NAMED (1 of 1)	0.78	4.64E-02	0.15	7.39E-01	0.68	1.30E-01	0.07	8.93E-01
Phvul.001G026700	PTHR26374:SF208 - ZINC FINGER PROTEIN AZF3-RELATED (1 of 2)	0.78	5.50E-02	1.08	9.44E-04	0.73	1.06E-01	1.05	1.95E-03
Phvul.008G011100	PF03140 - Plant protein of unknown function (DUF247) (1 of 36)	0.78	3.50E-01	-1.01	6.42E-02	0.48	6.57E-01	-1.72	2.29E-03
Phvul.010G121100	PTHR31989:SF5 - F11O4.3-RELATED (1 of 8)	0.78	8.73E-02	0.81	2.67E-02	0.67	2.01E-01	0.96	9.79E-03
Phvul.001G047900	K04733 - interleukin-1 receptor-associated kinase 4 (IRAK4) (1 of 48)	0.78	4.84E-01	1.65	7.17E-03	1.02	3.45E-01	1.30	4.87E-02
Phvul.001G127601	no data	0.78	1.64E-01	1.28	2.02E-03	0.53	4.40E-01	0.79	7.91E-02
Phvul.003G174800	no data	0.77	1.86E-01	1.00	2.18E-02	1.08	4.90E-02	1.27	4.43E-03
Phvul.005G080300	no data	0.77	6.12E-01	2.14	1.14E-02	0.99	5.28E-01	1.61	7.47E-02
Phvul.001G183800	PTHR13683:SF350 - EUKARYOTIC ASPARTYL PROTEASE FAMILY PROTEIN (1 of 1)	0.77	3.34E-02	0.68	2.95E-02	0.67	1.10E-01	0.52	1.23E-01
Phvul.011G184200	PF00407 - Pathogenesis-related protein Bet v I family (Bet v 1)	0.77	1.00E-02	0.86	9.60E-04	0.62	7.75E-02	0.78	4.18E-03
Phvul.006G050700	PTHR22903 - PLEKHH PROTEIN (1 of 2)	0.77	5.08E-01	1.35	7.15E-02	0.95	4.07E-01	1.56	3.47E-02
Phvul.002G102400	PTHR32227:SF56 - CARBOHYDRATE-BINDING X8 DOMAIN-CONTAINING PROTEIN (1 of 2)	0.77	4.26E-02	0.58	6.69E-02	0.39	4.57E-01	0.14	7.31E-01
Phvul.003G200500	PTHR32263:SF6 - INACTIVE POLY [ADP-RIBOSE] POLYMERASE SRO4-RELATED (1 of 2)	0.77	2.68E-02	0.69	1.85E-02	0.50	2.67E-01	0.80	7.08E-03
Phvul.006G173000	no data	0.76	6.93E-01	3.42	1.97E-02	1.60	3.23E-01	4.75	4.76E-04
Phvul.007G104000	PTHR19241//PTHR19241:SF261 - ATP-BINDING CASSETTE TRANSPORTER // SUBFAMILY NOT NAMED (1 of 4)	0.76	6.73E-02	1.22	1.96E-04	0.48	3.66E-01	1.02	3.10E-03
Phvul.007G208900	PTHR22595//PTHR22595:SF35 - CHITINASE-RELATED // SUBFAMILY NOT NAMED (1 of 2)	0.76	3.61E-02	1.49	3.67E-06	0.77	4.63E-02	1.39	1.64E-05
Phvul.006G178800	K19038 - E3 ubiquitin-protein ligase ATL41 [EC:6.3.2.19] (ATL41) (1 of 2)	0.76	2.43E-01	1.18	9.11E-03	0.82	2.39E-01	0.96	4.90E-02
Phvul.001G255800	PTHR10159//PTHR10159:SF359 - DUAL SPECIFICITY PROTEIN PHOSPHATASE // SUBFAMILY NOT NAMED (1 of 1)	0.76	7.04E-02	1.05	1.46E-03	0.65	1.82E-01	0.96	5.54E-03
Phvul.002G310600	PTHR31846:SF8 - CRS2-ASSOCIATED FACTOR 1, CHLOROPLASTIC (1 of 3)	0.76	4.92E-02	0.94	2.53E-03	0.61	1.86E-01	0.93	4.24E-03
Phvul.001G226100	PF07887 - Calmodulin binding protein-like (Calmodulin bind) (1 of 10)	0.76	1.03E-01	1.09	2.30E-03	0.79	1.10E-01	1.07	4.37E-03
Phvul.001G036800	3.2.1.153 - Fructan beta-(2,1)-fructosidase / Inulinase (1 of 3)	0.76	6.42E-02	0.67	4.82E-02	0.52	3.07E-01	0.56	1.19E-01
Phvul.008G225000	PTHR31301:SF24 - LOB DOMAIN-CONTAINING PROTEIN 21 (1 of 2)	0.75	4.72E-01	1.45	2.99E-02	0.82	4.58E-01	1.48	3.22E-02
Phvul.010G022600	K17961 - cytochrome P450, family 82, subfamily G, polypeptide 1 (CYP82G1) (1 of 2)	0.75	2.61E-01	1.76	8.28E-05	-0.03	9.85E-01	1.04	3.60E-02
Phvul.005G040800	PTHR10994:SF84 - RETICULON-LIKE PROTEIN B21 (1 of 1)	0.75	5.20E-01	1.67	1.05E-02	-0.20	9.32E-01	1.21	8.77E-02
Phvul.011G101500	2.7.1.107 - Diacylglycerol kinase (ATP) / Diglyceride kinase (1 of 13)	0.75	2.33E-01	1.01	2.80E-02	0.53	4.88E-01	0.38	4.96E-01
Phvul.009G154000	PTHR21493//PTHR21493:SF130 - CGI-141-RELATED/LIPASE CONTAINING PROTEIN // SUBFAMILY NOT NAMED (1 of 2)	0.75	1.23E-01	1.46	3.41E-05	0.66	2.32E-01	1.20	1.25E-03
Phvul.008G210814	PTHR11695:SF492 - ALCOHOL DEHYDROGENASE-LIKE 1-RELATED (1 of 2)	0.75	1.58E-01	1.10	4.49E-03	0.62	3.11E-01	0.95	2.10E-02
Phvul.004G084688	1.4.1.2 - Glutamate dehydrogenase / Glutamic dehydrogenase (1 of 2)	0.74	2.54E-01	1.64	3.84E-04	0.66	3.72E-01	1.71	2.81E-04
Phvul.004G099900	2.7.10.1//2.7.11.1 - Receptor protein-tyrosine kinase / Receptor protein tyrosine kinase // Non-specific serine/threonine	0.74	2.16E-01	1.53	3.88E-04	0.53	4.67E-01	1.36	2.73E-03
Phvul.011G084300	K01904 - 4-coumarate--CoA ligase (4CL) (1 of 9)	0.74	3.94E-01	1.11	6.53E-02	0.67	4.95E-01	1.30	3.28E-02
Phvul.001G014400	PF00560//PF07714 - Leucine Rich Repeat (LRR 1) // Protein tyrosine kinase (Pkinase Tyr) (1 of 4)	0.74	9.11E-02	0.78	2.78E-02	0.59	2.52E-01	0.71	5.48E-02
Phvul.002G083600	PF01357//PF03330 - Pollen allergen (Pollen allerg 1) // Rare lipoprotein A (RlpA)-like double-psi beta-barrel (DPBB 1) (1	0.74	6.11E-01	-2.08	2.16E-02	0.35	8.52E-01	-1.60	9.90E-02
Phvul.003G269600	2.5.1.20 - Rubber cis-polyprenylcistransferase / Rubber transferase (1 of 1)	0.74	2.27E-01	0.91	4.11E-02	0.56	4.40E-01	0.70	1.39E-01
Phvul.002G222600	PF07887 - Calmodulin binding protein-like (Calmodulin bind) (1 of 10)	0.73	2.12E-01	1.14	7.43E-03	0.86	1.54E-01	1.17	9.13E-03
Phvul.004G096500	K18532 - adenylate kinase (AK6, FAP7) (1 of 1)	0.73	1.90E-01	0.96	2.25E-02	0.76	2.01E-01	1.08	1.28E-02
Phvul.009G150800	K15356 - GDP-mannose transporter (VRG4, GONST1) (1 of 3)	0.73	1.44E-01	1.04	5.43E-03	0.46	4.72E-01	0.74	6.90E-02
Phvul.002G051000	no data	0.73	2.28E-01	0.61	1.91E-01	1.10	4.05E-02	0.89	4.46E-02
Phvul.002G013700	PTHR13832:SF362 - PROTEIN PHOSPHATASE 2C 63-RELATED (1 of 2)	0.73	2.44E-01	1.60	2.29E-04	0.83	2.02E-01	1.53	5.85E-04
Phvul.004G154550	PTHR30566:SF13 - MECHANOSENSITIVE ION CHANNEL PROTEIN 2, CHLOROPLASTIC (1 of 2)	0.73	3.18E-02	0.43	1.82E-01	0.45	3.34E-01	0.04	9.37E-01
Phvul.002G235900	K06689 - ubiquitin-conjugating enzyme E2 D/E (UBE2D E, UBC4, UBC5) (1 of 8)	0.73	5.19E-02	0.93	1.79E-03	0.50	2.81E-01	0.92	3.16E-03
Phvul.004G088788	PF00169//PF01852 - PH domain (PH) // START domain (START) (1 of 1)	0.73	9.08E-02	0.94	4.93E-03	0.70	1.35E-01	0.84	1.84E-02
Phvul.002G005800	no data	0.73	8.28E-02	0.47	2.04E-01	0.62	1.96E-01	0.70	4.95E-02
Phvul.011G125200	no data	0.73	3.90E-02	0.65	2.54E-02	0.70	6.39E-02	0.63	3.61E-02
Phvul.002G091100	PTHR31221:SF22 - WRKY TRANSCRIPTION FACTOR 3-RELATED (1 of 2)	0.72	1.11E-01	1.01	3.51E-03	0.57	2.88E-01	0.83	2.39E-02
Phvul.005G063200	1.14.13.173 - 11-oxo-beta-amyrin 30-oxidase / CYP72A154 (1 of 17)	0.72	2.08E-01	0.92	3.02E-02	0.72	2.54E-01	0.76	9.07E-02
Phvul.008G251300	K13424 - WRKY transcription factor 33 (WRKY33) (1 of 4)	0.72	5.61E-02	0.78	1.07E-02	0.75	5.71E-02	0.87	6.16E-03
Phvul.001G243600	PTHR27003:SF102 - SERINE/THREONINE-PROTEIN KINASE-LIKE PROTEIN CCR3-RELATED (1 of 6)	0.72	3.80E-01	1.60	3.87E-03	0.74	4.04E-01	1.86	9.20E-04
Phvul.006G089000	PTHR10992:SF907 - ALPHA/BETA-HYDROLASES SUPERFAMILY PROTEIN (1 of 5)	0.72	2.27E-02	0.64	1.88E-02	0.71	3.47E-02	0.43	1.52E-01
Phvul.002G136200	PTHR31062:SF16 - XYLOGLUCAN ENDOTRANSGLUCOSYLASE/HYDROLASE PROTEIN 29-RELATED (1 of 1)	0.71	2.28E-01	1.13	7.72E-03	0.78	2.11E-01	1.20	6.23E-03
Phvul.001G013800	PTHR22849:SF40 - U-BOX DOMAIN-CONTAINING PROTEIN 5 (1 of 3)	0.71	2.52E-01	0.93	3.90E-02	0.71	3.04E-01	0.89	5.78E-02
Phvul.004G098301	PF04554 - Extensin-like region (Extensin 2) (1 of 3)	0.71	2.53E-01	1.41	1.27E-03	0.60	3.99E-01	0.89	5.92E-02
Phvul.008G124900	PTHR33385:SF4 - PROTEIN XRI1 (1 of 3)	0.71	2.43E-01	1.19	3.10E-03	0.66	3.38E-01	0.76	8.76E-02
Phvul.008G277310	KOG0617 - Ras suppressor protein (contains leucine-rich repeats) (1 of 30)	0.71	2.16E-02	1.08	2.23E-05	0.73	2.52E-02	0.99	1.46E-04
Phvul.005G115600	4.2.1.51//4.2.1.91 - Arogenate dehydratase / Cyclohexadienyl dehydratase (1 of 5)	0.71	2.42E-03	0.73	4.75E-04	0.53	5.44E-02	0.60	6.60E-03
Phvul.006G156700	K15813 - beta-amyrin synthase (LUP4) (1 of 8)	0.71	1.03E-02	0.80	8.06E-04	0.48	1.64E-01	0.52	4.46E-02
Phvul.005G077701	PTHR32285:SF30 - PROTEIN TRICHOME BIREFRINGENCE-LIKE 42 (1 of 2)	0.71	5.11E-01	1.31	5.02E-02	0.16	9.34E-01	1.52	2.42E-02
Phvul.007G039200	PTHR11926:SF167 - UDP-GLYCOSYLTRANSFERASE 84A1-RELATED (1 of 2)	0.70	5.00E-01	1.95	2.16E-03	0.30	8.41E-01	1.81	7.03E-03
Phvul.003G021000	PTHR33077:SF7 - PROTEIN TIFY 6A-RELATED (1 of 2)	0.70	6.11E-02	0.34	3.54E-01	0.56	2.06E-01	0.81	1.25E-02
Phvul.001G073700	PTHR11909:SF97 - CASEIN KINASE 1-LIKE PROTEIN 4 (1 of 4)	0.70	2.32E-01	1.26	1.59E-03	0.48	5.09E-01	1.12	7.90E-03
Phvul.006G017400	3.1.1.4 - Phospholipase A(2) / Phospholipase A2 (1 of 3)	0.70	4.71E-01	1.26	4.18E-02	0.84	3.98E-01	0.97	1.44E-01
Phvul.004G132200	K15777 - 4,5-DOPA dioxygenase extradiol (DOPA) (1 of 3)	0.70	1.71E-01	1.20	5.33E-04	1.04	2.40E-02	1.01	5.98E-03
Phvul.001G194600	PTHR31415:SF4 - HARPIN-INDUCED PROTEIN-LIKE-RELATED (1 of 9)	0.70	3.16E-01	1.85	9.49E-05	1.12	6.43E-02	1.48	3.36E-03
Phvul.010G121500	PTHR32440:SF0 - PHOSPHATASE DCR2-RELATED (1 of 2)	0.70	1.95E-02	0.93	2.38E-04	0.61	6.79E-02	0.97	1.38E-04
Phvul.008G277352	KOG0617 - Ras suppressor protein (contains leucine-rich repeats) (1 of 30)	0.70	8.26E-02	1.37	7.14E-06	0.48	3.34E-01	1.09	6.31E-04
Phvul.011G063900	PTHR22763//PTHR22763:SF42 - RING ZINC FINGER PROTEIN // SUBFAMILY NOT NAMED (1 of 1)	0.70	1.03E-01	0.72	3.40E-02	0.61	2.12E-01	0.62	8.11E-02
Phvul.009G011400	PTHR13711:SF222 - HIGH MOBILITY GROUP B PROTEIN 4-RELATED (1 of 1)	0.69	3.74E-02	1.11	3.56E-05	0.75	2.80E-02	1.18	1.17E-05
Phvul.011G015500	no data	0.69	3.17E-01	0.43	4.53E-01	0.70	3.44E-01	1.08	3.30E-02
Phvul.001G225300	PF00582 - Universal stress protein family (Usp) (1 of 40)	0.69	1.23E-01	0.75	2.57E-02	0.41	4.92E-01	0.55	1.32E-01
Phvul.007G076300	PTHR23074//PTHR23074:SF98 - AAA ATPASE // SUBFAMILY NOT NAMED (1 of 3)	0.69	3.45E-02	1.05	7.60E-05	0.59	1.19E-01	0.94	6.31E-04
Phvul.004G148400	no data	0.69	1.84E-01	0.60	1.51E-01	0.66	2.49E-01	0.88	3.07E-02
Phvul.002G094800	K09645 - vitellogenic carboxypeptidase-like protein [EC:3.4.16.-] (CPVL) (1 of 1)	0.69	2.16E-01	1.15	4.85E-03	0.70	2.51E-01	1.23	3.55E-03
Phvul.006G156600	5.4.99.39 - Beta-amyrin synthase / 2,3-oxidosqualene beta-amyrin cyclase (1 of 9)	0.69	1.77E-01	1.19	1.82E-03	0.61	2.93E-01	1.03	1.04E-02
Phvul.003G047200	PTHR24093:SF272 - CADMIUM/ZINC-TRANSPORTING ATPASE HMA1, CHLOROPLASTIC-RELATED (1 of 2)	0.69	3.23E-01	1.18	1.48E-02	0.45	6.11E-01	1.24	1.47E-02
Phvul.007G102800	K09287 - RAV-like factor (RAV) (1 of 3)	0.69	1.25E-01	0.77	2.78E-02	0.78	9.16E-02	0.68	6.57E-02
Phvul.004G026100	PTHR10264//PTHR10264:SF93 - BAND 7 PROTEIN-RELATED // SUBFAMILY NOT NAMED (1 of 3)	0.69	3.35E-01	1.03	4.10E-02	0.63	4.25E-01	1.08	3.76E-02
Phvul.006G137500	PTHR27005:SF7 - WALL-ASSOCIATED RECEPTOR KINASE-LIKE 1-RELATED (1 of 6)	0.69	6.06E-02	1.07	1.69E-04	0.61	1.40E-01	0.84	6.03E-03
Phvul.001G036700	3.2.1.153 - Fructan beta-(2,1)-fructosidase / Inulinase (1 of 3)	0.69	1.60E-01	0.90	1.52E-02	0.44	4.78E-01	0.84	3.23E-02
Phvul.002G099000	2.5.1.3 - Thiamine-phosphate diphosphorylase / TMP pyrophosphorylase (1 of 3)	0.69	2.21E-01	0.77	6.33E-02	0.67	2.81E-01	1.18	3.78E-03
Phvul.002G054300	K10875 - DNA repair and recombination protein RAD54 and RAD54-like protein (RAD54L, RAD54) (1 of 7)	0.68	1.03E-01	0.91	5.03E-03	0.43	4.19E-01	0.69	4.71E-02
Phvul.002G127200	PTHR11819 - SODIUM/SOLUTE SYMPORTER (1 of 1)	0.68	1.12E-01	0.74	2.87E-02	0.56	2.70E-01	0.46	2.18E-01
Phvul.007G039400	K13692 - UDP-glucose:(indol-3-yl)acetate beta-D-glucosyltransferase [EC:2.4.1.121] Glc b1- IAA (IAGLU) (1 of 4)	0.68	2.16E-01	1.04	7.74E-03	0.94	7.12E-02	1.19	3.04E-03
Phvul.008G033000	K01180 - endo-1,3(4)-beta-glucanase (E3.2.1.6) (1 of 6)	0.68	4.50E-01	1.28	3.42E-02	0.30	8.12E-01	1.42	2.72E-02
Phvul.007G260300	PTHR27007:SF56 - L-TYPE LECTIN-DOMAIN CONTAINING RECEPTOR KINASE S.5-RELATED (1 of 3)	0.68	4.59E-01	0.96	8.78E-02	1.03	2.26E-01	1.69	1.31E-03
Phvul.009G243100	PTHR11785:SF267 - CATIONIC AMINO ACID TRANSPORTER 5 (1 of 2)	0.68	4.74E-01	1.38	1.38E-02	0.70	5.07E-01	0.85	1.76E-01

Phvul.007G242900	PTHR32285:SF52 - PROTEIN TRICHOME BIREFRINGENCE-LIKE 36 (1 of 2)	0.68	4.84E-01	1.55	1.13E-02	0.42	7.45E-01	1.78	3.87E-03
Phvul.002G133600	PTHR23155//PTHR23155:SF633 - LEUCINE-RICH REPEAT-CONTAINING PROTEIN // SUBFAMILY NOT NAMED (1 of 2)	0.68	3.15E-01	1.24	6.35E-03	0.55	4.80E-01	1.36	4.14E-03
Phvul.006G205400	PTHR32332:SF20 - 2-NITROPROPANE DIOXYGENASE-LIKE PROTEIN (1 of 1)	0.67	1.21E-01	0.88	8.90E-03	0.42	4.57E-01	0.61	9.36E-02
Phvul.008G127100	K02872 - large subunit ribosomal protein L13Ae (RP-L13Ae, RPL13A) (1 of 2)	0.67	1.77E-01	1.14	1.25E-03	0.63	2.59E-01	0.90	1.74E-02
Phvul.011G018500	PF04640 - PLATZ transcription factor (PLATZ) (1 of 15)	0.67	2.27E-01	0.84	4.22E-02	0.62	3.18E-01	0.91	3.26E-02
Phvul.009G157000	PF11961 - Domain of unknown function (DUF3475) (DUF3475) (1 of 15)	0.67	1.39E-03	0.70	2.72E-04	0.37	2.16E-01	0.83	1.28E-05
Phvul.006G181500	PTHR31079:SF5 - NAC DOMAIN CONTAINING PROTEIN 10 (1 of 2)	0.67	3.28E-01	1.35	5.27E-03	0.62	4.25E-01	1.13	2.95E-02
Phvul.L001947	PTHR24064:SF297 - INORGANIC PHOSPHATE TRANSPORTER 1-1-RELATED (1 of 2)	0.67	6.12E-03	0.93	1.12E-05	0.63	1.73E-02	0.85	8.11E-05
Phvul.004G125300	PTHR31718:SF8 - EMBRYO-SPECIFIC PROTEIN 3 (1 of 3)	0.67	8.59E-02	0.74	1.51E-02	0.50	2.88E-01	0.56	8.65E-02
Phvul.006G129600	PTHR31388:SF22 - PEROXIDASE 22-RELATED (1 of 10)	0.67	1.03E-02	0.79	4.22E-04	0.53	7.72E-02	0.83	2.77E-04
Phvul.009G046700	no data	0.66	2.08E-02	0.79	1.09E-03	0.46	2.05E-01	0.46	8.65E-02
Phvul.008G033200	K01180 - endo-1,3(4)-beta-glucanase (E3.2.1.6) (1 of 6)	0.66	3.69E-01	1.51	2.57E-03	0.57	4.97E-01	1.38	9.93E-03
Phvul.005G078900	PF07714//PF11721//PF13855 - Protein tyrosine kinase (Pkinase Tyr) // Di-glucose binding within endoplasmic reticulum	0.66	2.12E-02	0.97	4.76E-05	0.51	1.49E-01	0.73	4.32E-03
Phvul.007G057600	PTHR10314//PTHR10314:SF101 - SER/THR DEHYDRATASE, TRP SYNTHASE // SUBFAMILY NOT NAMED (1 of 3)	0.66	8.12E-02	0.66	3.32E-02	0.50	2.70E-01	0.47	1.54E-01
Phvul.002G067400	PTHR22870//PTHR22870:SF190 - REGULATOR OF CHROMOSOME CONDENSATION // SUBFAMILY NOT NAMED (1 of 4)	0.66	3.27E-02	0.72	4.73E-03	0.77	1.29E-02	0.63	1.87E-02
Phvul.002G288700	PTHR13994//PTHR13994:SF15 - NUDIX HYDROLASE RELATED // SUBFAMILY NOT NAMED (1 of 2)	0.66	5.40E-01	1.05	1.64E-01	0.77	4.86E-01	1.66	1.97E-02
Phvul.004G104100	no data	0.66	7.26E-02	0.66	2.97E-02	0.50	2.60E-01	0.36	2.86E-01
Phvul.001G241200	PTHR19139:SF90 - AQUAPORIN PIP1-5-RELATED (1 of 1)	0.66	1.97E-01	0.84	2.99E-02	0.64	2.55E-01	0.52	2.23E-01
Phvul.003G284800	PTHR31065:SF2 - PLATZ TRANSCRIPTION FACTOR DOMAIN-CONTAINING PROTEIN (1 of 2)	0.66	1.84E-01	0.80	3.41E-02	0.60	2.86E-01	0.82	3.59E-02
Phvul.011G089800	PTHR22950:SF319 - GABA TRANSPORTER 1 (1 of 3)	0.66	1.20E-01	0.67	4.77E-02	0.65	1.63E-01	0.56	1.18E-01
Phvul.008G265300	PTHR24073:SF515 - GENOMIC DNA, CHROMOSOME 3, P1 CLONE: MSD21 (1 of 3)	0.66	3.64E-01	1.01	4.11E-02	0.47	5.89E-01	0.91	7.71E-02
Phvul.006G089300	K02883 - large subunit ribosomal protein L18e (RP-L18e, RPL18) (1 of 4)	0.66	3.61E-02	0.34	2.47E-01	0.44	2.71E-01	0.16	6.42E-01
Phvul.008G252101	PTHR31221:SF20 - WRKY TRANSCRIPTION FACTOR 12-RELATED (1 of 10)	0.66	5.79E-01	1.50	6.24E-02	0.56	6.88E-01	2.10	5.56E-03
Phvul.008G276600	2.7.11.1 - Non-specific serine/threonine protein kinase / Threonine-specific protein kinase (1 of 1198)	0.65	6.29E-01	1.63	1.29E-02	1.43	1.59E-01	1.54	2.62E-02
Phvul.006G001100	PTHR10857//PTHR10857:SF38 - COPINE // SUBFAMILY NOT NAMED (1 of 1)	0.65	3.22E-02	1.07	1.48E-05	0.70	2.68E-02	1.04	3.21E-05
Phvul.008G277184	PF13855 - Leucine rich repeat (LRR 8) (1 of 311)	0.65	1.69E-01	1.03	2.96E-03	0.47	4.13E-01	1.00	6.06E-03
Phvul.009G001500	K14488 - SAUR family protein (SAUR) (1 of 75)	0.65	5.15E-01	1.31	5.46E-02	-0.08	9.65E-01	1.59	2.06E-02
Phvul.008G276100	KOG0617 - Ras suppressor protein (contains leucine-rich repeats) (1 of 30)	0.65	7.65E-02	1.04	2.67E-04	0.59	1.55E-01	0.93	2.01E-03
Phvul.003G019500	PF12796//PF13962 - Ankyrin repeats (3 copies) (Ank 2) // Domain of unknown function (PGG) (1 of 27)	0.65	1.64E-02	0.11	7.37E-01	0.47	1.60E-01	0.11	7.14E-01
Phvul.008G008200	PF14368 - Probable lipid transfer (LTP 2) (1 of 66)	0.65	3.74E-02	0.93	2.35E-04	0.54	1.37E-01	0.43	1.33E-01
Phvul.009G228900	PTHR14155//PTHR14155:SF93 - RING FINGER DOMAIN-CONTAINING // SUBFAMILY NOT NAMED (1 of 2)	0.65	1.59E-01	0.71	4.37E-02	0.62	2.35E-01	0.34	4.01E-01
Phvul.011G149466	2.7.11.1 - Non-specific serine/threonine protein kinase / Threonine-specific protein kinase (1 of 1198)	0.65	3.58E-01	1.32	2.72E-03	0.66	3.86E-01	1.07	2.39E-02
Phvul.002G108700	PTHR24349:SF87 - CALCIUM-DEPENDENT PROTEIN KINASE 16-RELATED (1 of 2)	0.65	1.05E-01	0.95	2.00E-03	0.74	6.55E-02	0.95	2.80E-03
Phvul.004G118000	K11270 - chromosome transmission fidelity protein 8 (CTF8) (1 of 1)	0.64	1.29E-01	0.72	3.05E-02	0.45	3.91E-01	0.65	6.28E-02
Phvul.010G128900	1.14.13.21 - Flavonoid 3'-monooxygenase / Flavonoid 3'-hydroxylase (1 of 8)	0.64	2.90E-01	1.20	4.75E-03	0.63	3.46E-01	1.64	1.16E-04
Phvul.002G188800	PTHR33734:SF5 - LYSM DOMAIN-CONTAINING GPI-ANCHORED PROTEIN 2 (1 of 1)	0.64	1.27E-01	1.32	2.54E-05	0.59	2.19E-01	1.11	7.04E-04
Phvul.007G231700	PTHR23083//PTHR23083:SF380 - TETRATRICOPEPTIDE REPEAT PROTEIN, TPR // SUBFAMILY NOT NAMED (1 of 1)	0.64	1.05E-01	0.66	3.75E-02	0.62	1.60E-01	0.75	2.18E-02
Phvul.011G145600	no data	0.64	5.50E-02	0.45	1.25E-01	0.76	2.13E-02	0.24	4.62E-01
Phvul.011G149532	PF00954//PF01453//PF08276 - S-locus glycoprotein domain (S locus glycop) // D-mannose binding lectin (B lectin) // PA	0.64	5.21E-01	1.43	2.08E-02	0.68	5.27E-01	1.30	4.90E-02
Phvul.008G029500	K04730 - interleukin-1 receptor-associated kinase 1 (IRAK1) (1 of 24)	0.64	4.04E-02	0.61	1.80E-02	0.55	1.27E-01	0.60	2.44E-02
Phvul.002G235500	PTHR11785//PTHR11785:SF359 - AMINO ACID TRANSPORTER // SUBFAMILY NOT NAMED (1 of 1)	0.64	1.44E-01	0.68	4.60E-02	0.58	2.49E-01	0.66	6.11E-02
Phvul.002G080800	PTHR31205:SF2 - ACTIN CROSS-LINKING PROTEIN (1 of 2)	0.64	5.14E-02	0.62	2.32E-02	0.64	7.12E-02	0.67	1.61E-02
Phvul.003G150200	PTHR33307:SF2 - BNR/ASP-BOX REPEAT FAMILY PROTEIN (1 of 2)	0.64	1.38E-01	0.61	6.53E-02	0.74	9.19E-02	0.83	1.02E-02
Phvul.L005001	K01761 - methionine-gamma-lyase (E4.4.1.11) (1 of 2)	0.64	3.32E-02	-0.32	2.67E-01	0.53	1.34E-01	-0.22	4.86E-01
Phvul.002G241400	PTHR32077:SF7 - FASCICLIN-LIKE ARABINOGALACTAN PROTEIN 11 (1 of 25)	0.64	5.79E-02	0.65	1.91E-02	0.63	8.31E-02	0.66	2.15E-02
Phvul.011G213500	PF00010 - Helix-loop-helix DNA-binding domain (HLH) (1 of 158)	0.64	1.72E-01	0.73	4.08E-02	0.50	3.64E-01	0.58	1.31E-01
Phvul.001G266300	PTHR10015:SF157 - HEAT STRESS TRANSCRIPTION FACTOR A-5 (1 of 2)	0.64	2.75E-01	0.82	4.28E-02	0.39	6.03E-01	0.73	8.24E-02
Phvul.007G266700	PTHR11306//PTHR11306:SF12 - NIEMANN PICK TYPE C2 PROTEIN NPC2-RELATED // SUBFAMILY NOT NAMED (1 of 1)	0.64	3.43E-02	0.41	1.36E-01	0.36	3.64E-01	0.39	1.61E-01
Phvul.010G049200	PTHR23067//PTHR23067:SF43 - DOUBLE-STRANDED RNA-BINDING ZINC FINGER PROTEIN // SUBFAMILY NOT NAMED (1	0.63	3.33E-01	1.44	1.14E-03	0.39	6.40E-01	1.26	6.25E-03
Phvul.008G119500	PTHR11709:SF66 - LACCASE-17 (1 of 2)	0.63	2.34E-01	0.86	2.73E-02	0.46	4.72E-01	0.74	7.38E-02
Phvul.007G241600	K06228 - fused (FU) (1 of 1)	0.63	1.18E-01	0.86	5.28E-03	0.80	4.05E-02	0.79	1.58E-02
Phvul.008G041500	PTHR10641//PTHR10641:SF530 - MYB-LIKE DNA-BINDING PROTEIN MYB // SUBFAMILY NOT NAMED (1 of 2)	0.63	1.12E-01	0.85	4.81E-03	0.67	1.12E-01	0.60	6.84E-02
Phvul.002G290800	no data	0.63	1.79E-01	0.84	1.84E-02	0.87	4.74E-02	1.12	1.76E-03
Phvul.009G171200	PTHR27000:SF9 - LEUCINE-RICH REPEAT RECEPTOR-LIKE SERINE/THREONINE/TYROSINE-PROTEIN KINASE SOBIR1 (1 of 1)	0.63	4.15E-02	1.03	2.85E-05	0.71	2.21E-02	1.25	4.18E-07
Phvul.L002146	PTHR12298//PTHR12298:SF7 - PCDC2 PROGRAMMED CELL DEATH PROTEIN 2 -RELATED // SUBFAMILY NOT NAMED (1	0.63	1.27E-01	0.66	4.25E-02	0.65	1.38E-01	0.51	1.43E-01
Phvul.009G028500	PTHR11814//PTHR11814:SF109 - SULFATE TRANSPORTER // SUBFAMILY NOT NAMED (1 of 1)	0.63	1.52E-01	0.68	4.54E-02	0.51	3.20E-01	0.46	2.25E-01
Phvul.007G208500	K12126 - phytochrome-interacting factor 3 (PIF3) (1 of 3)	0.63	1.78E-01	0.63	8.56E-02	0.71	1.50E-01	0.82	2.61E-02
Phvul.002G096500	K12356 - coniferyl-alcohol glucosyltransferase (UGT72E) (1 of 3)	0.63	2.87E-01	1.15	4.52E-03	0.81	1.67E-01	1.17	5.75E-03
Phvul.001G075600	PTHR10992//PTHR10992:SF699 - ALPHA/BETA HYDROLASE FOLD-CONTAINING PROTEIN // SUBFAMILY NOT NAMED (1	0.63	4.20E-01	1.07	4.23E-02	0.53	5.56E-01	1.23	2.18E-02
Phvul.011G154800	no data	0.62	2.05E-03	0.25	2.26E-01	0.35	2.11E-01	0.12	6.15E-01
Phvul.008G273600	PF00560//PF08263//PF13855 - Leucine Rich Repeat (LRR 1) // Leucine rich repeat N-terminal domain (LRRNT 2) // Leuc	0.62	1.81E-01	0.89	1.31E-02	0.12	8.80E-01	0.82	3.14E-02
Phvul.008G164600	no data	0.62	1.92E-01	0.66	7.58E-02	0.55	3.24E-01	0.74	4.85E-02
Phvul.001G199600	3.4.17.11 - Glutamate carboxypeptidase / Carboxypeptidase G2 (1 of 2)	0.62	3.95E-02	0.45	9.34E-02	0.55	1.09E-01	0.22	4.69E-01
Phvul.005G057400	PTHR31568:SF17 - EXPRESSED PROTEIN (1 of 4)	0.62	6.11E-01	1.70	2.94E-02	0.79	5.14E-01	1.89	1.78E-02
Phvul.008G145900	PTHR26374:SF175 - C2H2-LIKE ZINC FINGER PROTEIN-RELATED (1 of 3)	0.62	1.22E-01	0.71	2.23E-02	0.42	4.04E-01	0.59	7.55E-02
Phvul.004G114600	6.2.1.8 - Oxalate--CoA ligase / Oxalyl-CoA synthetase (1 of 4)	0.62	2.87E-02	0.69	3.74E-03	0.67	2.26E-02	0.77	1.58E-03
Phvul.006G013100	K13083 - flavonoid 3',5'-hydroxylase (CYP75A) (1 of 2)	0.62	1.15E-01	0.64	4.25E-02	0.46	3.44E-01	0.40	2.44E-01
Phvul.006G192400	PF00036//PF00070 - EF hand (EF-hand 1) // Pyridine nucleotide-disulphide oxidoreductase (Pyr redox) (1 of 1)	0.62	5.99E-01	1.40	6.79E-02	0.88	4.39E-01	2.28	2.47E-03
Phvul.001G240100	3.1.3.2 - Acid phosphatase / Phosphomonoesterase (1 of 34)	0.61	2.38E-02	0.27	3.27E-01	0.33	3.77E-01	0.08	8.10E-01
Phvul.001G059700	PF01535//PF13041//PF13812 - PPR repeat (PPR) // PPR repeat family (PPR 2) // Pentatricopeptide repeat domain (PPR	0.61	3.45E-02	0.59	1.59E-02	0.52	1.20E-01	0.51	4.80E-02
Phvul.002G314800	PTHR22814//PTHR22814:SF104 - COPPER TRANSPORT PROTEIN ATOX1-RELATED // SUBFAMILY NOT NAMED (1 of 2)	0.61	1.97E-01	0.74	4.13E-02	0.56	3.01E-01	0.44	2.86E-01
Phvul.008G276400	2.7.11.1 - Non-specific serine/threonine protein kinase / Threonine-specific protein kinase (1 of 1198)	0.61	6.02E-01	1.71	1.25E-02	0.41	7.89E-01	1.32	7.84E-02
Phvul.011G139475	K03522 - electron transfer flavoprotein alpha subunit (fixB, etfA) (1 of 1)	0.61	2.39E-02	0.62	6.56E-03	0.51	1.06E-01	0.53	2.80E-02
Phvul.008G107300	PTHR13620 - 3-5 EXONUCLEASE (1 of 10)	0.61	8.63E-02	0.24	4.95E-01	0.51	2.24E-01	0.61	4.35E-02
Phvul.006G137300	PTHR12121:SF29 - PROTEIN ANGEL (1 of 2)	0.61	8.89E-02	0.66	2.00E-02	0.34	4.77E-01	0.53	8.12E-02
Phvul.007G009100	PTHR31561:SF24 - 3-KETOACYL-COA SYNTHASE 12-RELATED (1 of 4)	0.61	5.92E-01	0.85	2.46E-01	0.34	8.18E-01	1.39	4.60E-02
Phvul.010G033866	4.1.1.97 - 2-oxo-4-hydroxy-4-carboxy-5-ureidoimidazoline decarboxylase / OHCU decarboxylase (1 of 1)	0.61	1.55E-02	0.64	2.72E-03	0.46	1.34E-01	0.38	1.06E-01
Phvul.004G080200	no data	0.61	3.57E-01	0.38	4.86E-01	0.76	2.49E-01	0.97	3.92E-02
Phvul.001G264066	PF04408//PF07717 - Helicase associated domain (HA2) (HA2) // Oligonucleotide/oligosaccharide-binding (OB)-fold (OB	0.61	2.38E-01	0.77	3.92E-02	0.59	2.92E-01	0.82	3.38E-02
Phvul.010G121000	PTHR31989:SF5 - F11O4.3-RELATED (1 of 8)	0.61	3.04E-01	0.86	3.66E-02	0.55	4.00E-01	1.04	1.12E-02
Phvul.004G048000	PTHR23155//PTHR23155:SF554 - LEUCINE-RICH REPEAT-CONTAINING PROTEIN // SUBFAMILY NOT NAMED (1 of 22)	0.60	6.64E-01	1.94	1.71E-02	0.41	8.17E-01	2.54	2.22E-03
Phvul.007G198600	PTHR18896:SF64 - PHOSPHOLIPASE D P2 (1 of 2)	0.60	1.78E-01	0.69	2.77E-02	0.83	4.74E-02	0.51	1.26E-01
Phvul.001G002200	no data	0.60	2.88E-01	0.86	3.07E-02	0.93	6.55E-02	1.02	1.11E-02
Phvul.006G149640	PTHR10969:SF43 - AUTOPHAGY-RELATED PROTEIN 8A-RELATED (1 of 3)	0.60	2.34E-01	0.76	3.92E-02	0.52	3.67E-01	0.65	9.45E-02
Phvul.005G158500	PTHR31953:SF16 - BETA-FRUCTOFURANOSIDASE, INSOLUBLE ISOENZYME CWINV1-RELATED (1 of 3)	0.60	2.78E-01	1.35	3.55E-04	0.52	4.07E-01	1.46	1.30E-04
Phvul.004G039600	PF07714//PF11721 - Protein tyrosine kinase (Pkinase Tyr) // Di-glucose binding within endoplasmic reticulum (Malectin	0.60	2.38E-01	0.53	1.69E-01	0.90	4.92E-02	0.65	8.30E-02
Phvul.007G128800	PTHR31371:SF4 - SIMILARITY TO UNKNOWN PROTEIN (1 of 2)	0.60	1.05E-01	0.69	1.86E-02	0.23	6.89E-01	0.21	5.68E-01
Phvul.003G112700	no data	0.60	1.42E-01	0.40	2.46E-01	0.81	3.60E-02	0.70	3.35E-02
Phvul.001G008900	PTHR24298:SF86 - CYTOCHROME P450 71B21-RELATED (1 of 10)	0.60	1.33E-01	0.72	1.82E-02	0.78	4.24E-02	0.70	2.77E-02
Phvul.003G123400	K08770 - ubiquitin C (UBC) (1 of 14)	0.60	1.75E-01	0.74	2.65E-02	0.58	2.34E-01	0.83	1.50E-02
Phvul.001G182500	K13496 - UDP-glucosyl transferase 73C [EC:2.4.1.-] Glc - brassinosteroid (UGT73C) (1 of 11)	0.60	1.91E-01	0.88	9.11E-03	0.78	8.38E-02	0.67	6.31E-02
Phvul.008G212500	PTHR11527:SF156 - HEAT SHOCK FAMILY PROTEIN (1 of 3)	0.60	1.75E-01	1.32	2.12E-05	0.66	1.56E-01	1.53	9.39E-07
Phvul.004G013300	K00122 - formate dehydrogenase (FDH) (1 of 2)	0.59	2.29E-01	0.81	1.61E-02	0.18	8.26E-01	0.61	9.14E-02
Phvul.011G097400	PTHR10797:SF3 - CCR4-ASSOCIATED FACTOR 1 HOMOLOG 11-RELATED (1 of 14)	0.59	6.47E-02	0.47	8.14E-02	0.67	4.05E-02	0.60	2.71E-02
Phvul.002G158200	no data	0.59	2.52E-01	1.22	5.47E-04	0.44	4.85E-01	1.15	1.77E-03
Phvul.009G231000	PTHR31250:SF13 - CALMODULIN-BINDING PROTEIN (1 of 3)	0.59	2.64E-01	0.92	1.47E-02	0.60	2.94E-01	1.08	5.18E-03
Phvul.002G220500	PF00954//PF01453//PF07714//PF08276 - S-locus glycoprotein domain (S locus glycop) // D-mannose binding lectin (B le	0.59	2.89E-01	1.01	9.36E-03	0.42	5.33E-01	1.04	1.06E-02
Phvul.007G170400	PTHR13774 - PHENAZINE BIOSYNTHESIS PROTEIN (1 of 4)	0.59	1.34E-01	0.64	2.90E-02	0.41	3.95E-01	0.47	1.37E-01
Phvul.004G173300	PF00069//PF12819//PF13855 - Protein kinase domain (Pkinase) // Carbohydrate-binding protein of the ER (Malectin lik	0.59	3.15E-01	0.83	4.60E-02	0.33	6.69E-01	0.66	1.36E-01
Phvul.006G043900	PTHR10388//PTHR10388:SF16 - EUKARYOTIC TRANSLATION INITIATION FACTOR SUI1 // SUBFAMILY NOT NAMED (1 of 2	0.58	8.02E-02	0.59	2.95E-02	0.72	2.68E-02	0.43	1.44E-01
Phvul.003G236500	PTHR31517:SF3 - PEROXIDASE 35-RELATED (1 of 2)	0.58	1.25E-02	0.63	1.82E-03	0.36	2.36E-01	0.43	5.10E-02
Phvul.008G168800	PTHR22952//PTHR22952:SF174 - CAMP-RESPONSE ELEMENT BINDING PROTEIN-RELATED // SUBFAMILY NOT NAMED (1	0.58	6.33E-03	0.59	9.42E-04	0.55	1.67E-02	0.40	4.22E-02
Phvul.007G085100	PF07343 - Protein of unknown function (DUF1475) (DUF1475) (1 of 2)	0.58	2.12E-02	0.64	2.90E-03	0.17	6.98E-01	0.52	2.39E-02

Phvul.003G016900	PTHR10174//PTHR10174:SF121 - RETINALDEHYDE BINDING PROTEIN-RELATED // SUBFAMILY NOT NAMED (1 of 1)	0.58	4.20E-02	0.54	2.24E-02	0.48	1.47E-01	0.49	5.10E-02
Phvul.009G096100	no data	0.58	1.94E-01	0.72	3.16E-02	0.47	3.57E-01	0.68	5.00E-02
Phvul.007G047500	no data	0.58	1.17E-01	0.58	5.00E-02	0.46	2.88E-01	0.66	2.82E-02
Phvul.009G240400	PTHR24177:SF42 - ANKYRIN REPEAT FAMILY PROTEIN (1 of 4)	0.58	2.49E-01	0.90	1.26E-02	0.41	4.97E-01	0.86	2.38E-02
Phvul.002G229000	PF01535//PF13041//PF14432 - PPR repeat (PPR) // PPR repeat family (PPR 2) // DYW family of nucleic acid deaminases	0.58	2.85E-01	0.87	2.17E-02	0.61	2.89E-01	0.97	1.40E-02
Phvul.001G100500	PTHR24012:SF490 - UBP1-ASSOCIATED PROTEIN 2C (1 of 5)	0.57	2.77E-01	0.84	2.23E-02	0.54	3.55E-01	0.96	1.16E-02
Phvul.006G022600	PTHR11260//PTHR11260:SF287 - GLUTATHIONE S-TRANSFERASE, GST, SUPERFAMILY, GST DOMAIN CONTAINING // SUB	0.57	3.71E-01	1.02	1.87E-02	0.32	7.09E-01	0.65	1.83E-01
Phvul.004G154800	PF07714//PF14380 - Protein tyrosine kinase (Pkinase Tyr) // Wall-associated receptor kinase C-terminal (WAK assoc) (1	0.57	2.35E-01	1.01	3.44E-03	0.47	3.98E-01	1.34	1.24E-04
Phvul.006G023500	no data	0.57	4.38E-01	1.63	4.75E-04	0.45	6.08E-01	1.54	1.59E-03
Phvul.005G104200	PTHR12313//PTHR12313:SF5 - RNF5 // SUBFAMILY NOT NAMED (1 of 2)	0.57	8.52E-02	0.76	3.32E-03	0.41	3.14E-01	0.74	6.06E-03
Phvul.002G309600	PTHR11945:SF229 - F23H11.23 PROTEIN-RELATED (1 of 4)	0.57	3.97E-01	1.25	4.86E-03	0.40	6.34E-01	0.56	2.69E-01
Phvul.008G114100	K08081 - Tropinone reductase 1 (TR1) (1 of 15)	0.57	1.44E-01	0.74	1.31E-02	0.50	2.73E-01	0.70	2.61E-02
Phvul.003G247200	PTHR23155//PTHR23155:SF543 - LEUCINE-RICH REPEAT-CONTAINING PROTEIN // SUBFAMILY NOT NAMED (1 of 10)	0.57	1.32E-01	1.01	3.58E-04	0.50	2.52E-01	1.02	4.75E-04
Phvul.010G006500	KOG4779 - Predicted membrane protein (1 of 3)	0.57	1.45E-01	0.71	1.78E-02	0.76	4.05E-02	0.54	9.44E-02
Phvul.008G089000	PTHR31087:SF11 - PROTEIN LURP-ONE-RELATED 1-RELATED (1 of 9)	0.57	1.98E-01	0.80	1.48E-02	0.48	3.52E-01	0.75	3.04E-02
Phvul.001G182900	K13496 - UDP-glucosyl transferase 73C [EC:2.4.1.-] Glc - brassinosteroid (UGT73C) (1 of 11)	0.57	1.09E-01	0.57	4.53E-02	0.69	5.11E-02	0.45	1.39E-01
Phvul.008G267700	PF01582//PF13676 - TIR domain (TIR) // TIR domain (TIR 2) (1 of 2)	0.57	6.85E-01	1.59	5.08E-02	1.23	2.76E-01	1.88	2.04E-02
Phvul.006G052200	PTHR24223:SF181 - ABC TRANSPORTER C FAMILY MEMBER 3-RELATED (1 of 2)	0.57	1.11E-01	0.63	2.51E-02	0.19	7.33E-01	0.34	2.85E-01
Phvul.001G072200	K02735 - 20S proteasome subunit beta 3 (PSMB3) (1 of 1)	0.57	1.44E-01	0.42	2.04E-01	0.93	5.92E-03	0.50	1.18E-01
Phvul.001G067400	PTHR10108//PTHR10108:SF897 - METHYLTRANSFERASE // SUBFAMILY NOT NAMED (1 of 1)	0.57	7.09E-01	1.80	3.41E-02	0.10	9.71E-01	2.30	4.94E-03
Phvul.002G104600	PF01641 - SelR domain (SelR) (1 of 5)	0.56	4.03E-01	0.44	4.04E-01	0.39	6.42E-01	1.15	1.22E-02
Phvul.011G211200	PTHR10543:SF37 - CAROTENOID 9,10(9',10')-CLEAVAGE DIOXYGENASE 1 (1 of 1)	0.56	1.36E-01	0.47	1.26E-01	0.57	1.59E-01	0.62	4.17E-02
Phvul.006G192600	PTHR10809:SF46 - VESICLE-ASSOCIATED PROTEIN 4-2-RELATED (1 of 1)	0.56	8.53E-02	0.77	2.20E-03	0.36	3.79E-01	0.77	3.16E-03
Phvul.003G034400	no data	0.56	2.36E-01	1.10	1.18E-03	0.70	1.41E-01	1.41	2.81E-05
Phvul.001G153200	PF04570 - zinc-finger of the FCS-type, C2-C2 (zf-FLZ) (1 of 19)	0.56	4.41E-01	1.28	6.08E-03	0.30	7.60E-01	0.83	1.02E-01
Phvul.008G042000	PTHR10168//PTHR10168:SF110 - GLUTAREDOXIN // SUBFAMILY NOT NAMED (1 of 3)	0.56	1.45E-01	0.45	1.66E-01	0.62	1.20E-01	0.63	4.56E-02
Phvul.008G196100	PTHR31479:SF3 - GB (1 of 3)	0.56	3.05E-02	0.19	4.75E-01	0.29	4.16E-01	0.19	4.76E-01
Phvul.008G112500	PTHR33103:SF4 - EMB (1 of 16)	0.56	1.54E-01	0.63	3.80E-02	0.57	1.83E-01	0.63	4.90E-02
Phvul.005G107800	PF12609 - Wound-induced protein (DUF3774) (1 of 13)	0.56	7.57E-02	0.39	1.49E-01	0.68	2.80E-02	0.36	1.96E-01
Phvul.006G080500	PTHR32258:SF4 - KINASE INTERACTING (KIP1-LIKE) FAMILY PROTEIN (1 of 2)	0.55	1.52E-01	1.02	3.64E-04	0.38	4.27E-01	0.72	1.91E-02
Phvul.011G169332	PTHR14155:SF180 - RING/U-BOX DOMAIN-CONTAINING PROTEIN-RELATED (1 of 7)	0.55	2.57E-01	0.99	2.97E-03	0.26	7.05E-01	0.89	1.19E-02
Phvul.003G058800	PF12776 - Myb/SANT-like DNA-binding domain (Myb DNA-bind 3) (1 of 47)	0.55	4.69E-01	0.81	1.06E-01	0.56	5.00E-01	1.06	3.21E-02
Phvul.003G220400	2.4.1.186 - Glycogenin glucosyltransferase / Priming glucosyltransferase (1 of 2)	0.55	5.30E-01	0.88	1.36E-01	0.48	6.32E-01	1.22	3.31E-02
Phvul.001G160600	PTHR24064:SF274 - INORGANIC PHOSPHATE TRANSPORTER 1-4 (1 of 2)	0.55	4.80E-01	0.97	5.95E-02	0.45	6.19E-01	1.16	2.90E-02
Phvul.008G137600	2.4.1.218 - Hydroquinone glucosyltransferase / Hydroquinone:O-glucosyltransferase (1 of 14)	0.55	1.59E-01	0.60	4.96E-02	0.39	4.13E-01	0.43	1.95E-01
Phvul.003G111500	1.3.1.3 - Delta(4)-3-oxosteroid 5-beta-reductase / Testosterone 5-beta-reductase (1 of 5)	0.55	9.25E-02	0.64	1.21E-02	0.54	1.28E-01	0.42	1.30E-01
Phvul.002G331900	PF05542 - Protein of unknown function (DUF760) (DUF760) (1 of 8)	0.55	1.31E-01	0.76	6.18E-03	0.52	2.02E-01	0.69	1.88E-02
Phvul.002G273904	K03495 - tRNA uridine 5-carboxymethylaminomethyl modification enzyme (gidA, mnmG, MTO1) (1 of 1)	0.55	5.52E-01	1.33	1.80E-02	0.73	4.27E-01	1.43	1.45E-02
Phvul.003G214350	PTHR24298:SF114 - CYTOCHROME P450 - LIKE PROTEIN-RELATED (1 of 2)	0.55	7.14E-01	1.71	2.71E-03	0.55	7.54E-01	1.01	1.18E-01
Phvul.004G159200	PTHR13683:SF334 - ASPARTYL PROTEASE (1 of 1)	0.55	1.71E-01	0.78	8.97E-03	0.41	3.96E-01	0.73	2.12E-02
Phvul.005G176100	PTHR10374//PTHR10374:SF15 - LACTOYLGLUTATHIONE LYASE GLYOXALASE I // SUBFAMILY NOT NAMED (1 of 2)	0.55	5.04E-01	1.09	3.99E-02	0.48	6.08E-01	0.85	1.33E-01
Phvul.011G084200	PTHR22814:SF100 - HEAVY METAL TRANSPORT/DETOXIFICATION DOMAIN-CONTAINING PROTEIN-RELATED (1 of 2)	0.55	3.08E-01	0.60	1.34E-01	0.88	7.05E-02	0.92	2.04E-02
Phvul.011G172000	PTHR27006:SF44 - LEUCINE-RICH REPEAT TRANSMEMBRANE PROTEIN KINASE (1 of 3)	0.55	7.00E-01	1.69	1.63E-02	0.80	5.67E-01	0.96	2.33E-01
Phvul.010G100200	PTHR24067//PTHR24067:SF156 - UBIQUITIN-CONJUGATING ENZYME E2 // SUBFAMILY NOT NAMED (1 of 2)	0.54	1.00E-01	0.41	1.46E-01	0.41	3.07E-01	0.55	4.85E-02
Phvul.008G260300	K17871 - NADH:ubiquinone reductase (non-electrogenic) (ndh1) (1 of 7)	0.54	2.57E-01	0.81	1.80E-02	0.44	4.34E-01	0.45	2.42E-01
Phvul.001G087100	3.4.22.69 - SARS coronavirus main proteinase / Severe acute respiratory syndrome coronavirus main protease (1 of 6)	0.54	7.67E-02	0.54	3.23E-02	0.37	3.44E-01	0.75	2.35E-03
Phvul.002G096600	PTHR15351:SF3 - PROTEIN ERL-1 (1 of 1)	0.54	7.73E-01	2.29	3.92E-02	0.01	9.98E-01	2.35	4.18E-02
Phvul.001G185500	KOG0156 - Cytochrome P450 CYP2 subfamily (1 of 118)	0.54	5.23E-01	1.03	4.61E-02	0.57	5.21E-01	0.96	7.27E-02
Phvul.001G163700	PF00069//PF00560//PF08263//PF13855 - Protein kinase domain (Pkinase) // Leucine Rich Repeat (LRR 1) // Leucine ric	0.54	4.49E-01	1.04	2.94E-02	0.33	7.27E-01	0.92	6.56E-02
Phvul.009G174200	PTHR11926//PTHR11926:SF378 - GLUCOSYL/GLUCURONOSYL TRANSFERASES // SUBFAMILY NOT NAMED (1 of 1)	0.54	5.21E-01	0.76	1.81E-01	0.60	4.92E-01	1.07	4.87E-02
Phvul.004G068400	KOG2922 - Uncharacterized conserved protein (1 of 11)	0.54	3.91E-01	0.77	8.41E-02	0.90	1.19E-01	1.03	2.06E-02
Phvul.002G055800	K09286 - EREBP-like factor (EREBP) (1 of 35)	0.54	7.35E-02	0.84	2.93E-04	0.79	3.33E-03	0.85	3.09E-04
Phvul.007G022500	PTHR19370:SF124 - FERREDOXIN-4, CHLOROPLASTIC-RELATED (1 of 1)	0.54	8.39E-02	0.77	1.28E-03	0.29	4.86E-01	0.36	1.86E-01
Phvul.003G074700	PTHR36024:SF1 - ANKYRIN REPEAT PROTEIN SKIP35 (1 of 1)	0.53	4.18E-02	0.15	5.90E-01	0.33	3.43E-01	0.04	9.05E-01
Phvul.001G130800	PF00069//PF07645//PF13947 - Protein kinase domain (Pkinase) // Calcium-binding EGF domain (EGF CA) // Wall-associ	0.53	2.23E-01	0.88	5.05E-03	0.55	2.50E-01	0.71	3.32E-02
Phvul.001G255900	PTHR10857:SF34 - COPINE (CALCIUM-DEPENDENT PHOSPHOLIPID-BINDING PROTEIN) FAMILY PROTEIN (1 of 3)	0.53	1.38E-01	0.98	2.25E-04	0.53	1.86E-01	0.95	4.81E-04
Phvul.003G206400	KOG0156 - Cytochrome P450 CYP2 subfamily (1 of 118)	0.53	6.10E-01	1.26	4.86E-02	0.52	6.54E-01	0.86	2.15E-01
Phvul.002G298300	no data	0.53	3.98E-01	1.55	3.71E-05	1.11	2.80E-02	1.77	2.65E-06
Phvul.009G190830	K18211 - synaptosomal-associated protein 25 (SNAP25) (1 of 3)	0.53	3.08E-02	0.63	2.12E-03	0.47	8.79E-02	0.47	3.29E-02
Phvul.002G210400	PTHR19241//PTHR19241:SF301 - ATP-BINDING CASSETTE TRANSPORTER // SUBFAMILY NOT NAMED (1 of 5)	0.53	1.64E-01	0.93	9.18E-04	0.28	5.85E-01	0.69	2.27E-02
Phvul.010G099400	PTHR27003:SF52 - U-BOX DOMAIN-CONTAINING PROTEIN 33 (1 of 3)	0.53	2.56E-01	0.60	8.11E-02	0.49	3.51E-01	0.87	9.91E-03
Phvul.006G199100	3.5.1.60 - N-(long-chain-acyl)ethanolamine deacylase / N-acylethanolamine amidohydrolase (1 of 1)	0.53	9.47E-02	0.70	5.42E-03	0.25	5.69E-01	0.59	2.88E-02
Phvul.011G183400	PF00407 - Pathogenesis-related protein Bet v I family (Bet v 1)	0.53	2.24E-02	0.27	2.33E-01	0.40	1.49E-01	0.30	1.78E-01
Phvul.005G135300	PTHR19139:SF158 - AQUAPORIN PIP2-4-RELATED (1 of 2)	0.53	2.36E-01	0.76	1.87E-02	0.48	3.30E-01	0.78	2.19E-02
Phvul.007G111200	PF05514 - HR-like lesion-inducing (HR lesion) (1 of 4)	0.53	4.97E-01	1.33	9.27E-03	1.07	9.16E-02	1.39	8.64E-03
Phvul.008G169600	no data	0.53	7.85E-01	1.63	1.72E-02	-1.11	6.18E-01	2.27	8.06E-04
Phvul.011G134500	PTHR11926//PTHR11926:SF356 - GLUCOSYL/GLUCURONOSYL TRANSFERASES // SUBFAMILY NOT NAMED (1 of 11)	0.52	1.93E-01	0.67	2.51E-02	0.42	3.78E-01	0.54	9.29E-02
Phvul.001G094100	K11368 - enhancer of yellow 2 transcription factor (ENY2, DC6, SUS1) (1 of 1)	0.52	3.12E-01	0.79	3.21E-02	0.45	4.40E-01	0.65	9.09E-02
Phvul.008G114800	PTHR27007:SF27 - L-TYPE LECTIN-DOMAIN CONTAINING RECEPTOR KINASE S.4 (1 of 2)	0.52	8.89E-02	0.75	1.57E-03	0.31	4.45E-01	0.66	8.78E-03
Phvul.002G293000	K11135 - Pin2-interacting protein X1 (PINX1) (1 of 1)	0.52	5.51E-01	1.22	2.66E-02	0.59	5.20E-01	1.33	2.07E-02
Phvul.007G054400	PTHR23024//PTHR23024:SF193 - MEMBER OF 'GDXG' FAMILY OF LIPOLYTIC ENZYMES // SUBFAMILY NOT NAMED (1 of	0.52	2.36E-01	1.14	1.53E-04	0.48	3.34E-01	1.05	7.86E-04
Phvul.001G041200	PF08263//PF13855 - Leucine rich repeat N-terminal domain (LRRNT 2) // Leucine rich repeat (LRR 8) (1 of 20)	0.52	7.50E-02	0.59	1.04E-02	0.49	1.32E-01	0.47	5.66E-02
Phvul.003G047100	2.4.1.263 - Abscisate beta-glucosyltransferase / ABA-GTase (1 of 1)	0.52	8.08E-01	-2.66	2.18E-02	0.14	9.64E-01	-1.75	1.53E-01
Phvul.011G198500	3.1.4.46 - Glycerophosphodiester phosphodiesterase / Glycerophosphoryl diester phosphodiesterase (1 of 65)	0.52	3.90E-01	0.57	1.90E-01	0.21	8.17E-01	0.88	3.65E-02
Phvul.007G019100	PTHR21704 - NIPPED-B-LIKE PROTEIN DELANGIN SCC2-RELATED (1 of 3)	0.52	4.99E-01	1.36	4.32E-03	0.42	6.32E-01	1.60	1.16E-03
Phvul.011G152200	PTHR27002:SF129 - INACTIVE G-TYPE LECTIN S-RECEPTOR-LIKE SERINE/THREONINE-PROTEIN KINASE SRK-RELATED (1 of	0.52	1.11E-01	0.84	6.23E-04	0.26	5.52E-01	0.54	4.08E-02
Phvul.005G123400	PTHR10183//PTHR10183:SF299 - CALPAIN // SUBFAMILY NOT NAMED (1 of 1)	0.52	9.55E-02	0.82	5.15E-04	0.51	1.26E-01	0.80	1.12E-03
Phvul.007G050700	PF01657//PF07714 - Salt stress response/antifungal (Stress-antifung) // Protein tyrosine kinase (Pkinase Tyr) (1 of 40)	0.51	1.78E-01	0.72	1.09E-02	0.41	3.67E-01	0.65	3.11E-02
Phvul.002G158800	PTHR14155 - RING FINGER DOMAIN-CONTAINING (1 of 159)	0.51	1.13E-01	0.12	7.38E-01	0.70	2.17E-02	0.30	2.96E-01
Phvul.006G141900	K00857 - thymidine kinase (tdk, TK) (1 of 2)	0.51	2.56E-01	0.99	2.72E-03	0.55	2.60E-01	0.46	2.33E-01
Phvul.007G251200	K13495 - cis-zeatin O-glucosyltransferase (CISZOG) (1 of 5)	0.51	4.14E-01	1.01	1.39E-02	0.61	3.47E-01	0.84	5.48E-02
Phvul.001G069001	K04733 - interleukin-1 receptor-associated kinase 4 (IRAK4) (1 of 48)	0.51	4.99E-01	1.19	7.27E-03	0.97	1.25E-01	1.43	1.29E-03
Phvul.010G064100	no data	0.51	2.71E-01	0.71	3.62E-02	0.44	4.13E-01	0.58	1.06E-01
Phvul.005G179800	no data	0.51	2.38E-01	0.46	1.97E-01	0.62	1.60E-01	0.82	1.27E-02
Phvul.010G122600	PTHR14155:SF99 - RING-H2 FINGER PROTEIN ATL3 (1 of 5)	0.51	3.67E-01	1.22	1.59E-03	0.49	4.25E-01	1.54	5.45E-05
Phvul.006G206300	PTHR13832:SF314 - PROTEIN PHOSPHATASE 2C 10-RELATED (1 of 3)	0.51	1.09E-01	0.76	1.72E-03	0.41	2.68E-01	0.83	8.91E-04
Phvul.004G173700	2.3.2.15//2.3.2.2//3.4.19.13 - Glutathione gamma-glutamylcysteinyltransferase / Phytochelatin synthase // Gamma-glut	0.51	2.49E-01	0.65	4.85E-02	0.36	4.97E-01	0.51	1.42E-01
Phvul.007G167600	K14493 - gibberellin receptor GID1 (GID1) (1 of 3)	0.51	1.99E-01	0.70	1.65E-02	0.49	2.64E-01	0.53	9.55E-02
Phvul.009G066200	PF02458 - Transferase family (Transferase) (1 of 94)	0.51	6.87E-01	1.23	4.04E-02	0.69	5.89E-01	1.35	2.84E-02
Phvul.009G144300	PTHR31100:SF1 - AT-HOOK MOTIF NUCLEAR LOCALIZED PROTEIN 17-RELATED (1 of 2)	0.51	9.24E-02	0.76	9.07E-04	0.51	1.19E-01	0.87	1.66E-04
Phvul.011G108500	PTHR10641//PTHR10641:SF584 - MYB-LIKE DNA-BINDING PROTEIN MYB // SUBFAMILY NOT NAMED (1 of 4)	0.51	6.36E-01	1.28	4.44E-02	0.50	6.76E-01	1.12	9.11E-02
Phvul.009G229700	PTHR13047//PTHR13047:SF1 - PRE-MRNA CLEAVAGE FACTOR IM, 25KD SUBUNIT // SUBFAMILY NOT NAMED (1 of 1)	0.51	5.21E-01	0.98	4.46E-02	0.22	8.38E-01	1.12	2.38E-02
Phvul.002G097300	PTHR32227:SF62 - GLUCOSIDASE-LIKE PROTEIN (1 of 1)	0.51	2.19E-03	0.16	3.68E-01	0.48	6.04E-03	0.28	9.99E-02
Phvul.006G081900	no data	0.51	5.45E-01	1.83	3.13E-04	0.10	9.45E-01	0.93	1.14E-01
Phvul.007G253700	PTHR11732:SF239 - ALDO/KETO REDUCTASE-RELATED (1 of 5)	0.51	6.50E-02	0.54	1.61E-02	0.28	4.34E-01	0.31	2.15E-01
Phvul.004G121900	PTHR11206:SF103 - MATE EFFLUX FAMILY PROTEIN (1 of 2)	0.51	4.97E-01	-0.50	4.08E-01	1.38	3.95E-03	0.76	1.15E-01
Phvul.009G052700	PTHR23056:SF45 - CALCINEURIN B-LIKE PROTEIN 4 (1 of 3)	0.51	4.23E-01	1.13	4.50E-03	0.35	6.48E-01	0.80	6.41E-02
Phvul.011G046700	PTHR11538:SF40 - PHENYLALANINE--TRNA LIGASE ALPHA SUBUNIT (1 of 1)	0.50	2.54E-02	0.62	9.87E-04	0.51	3.57E-02	0.63	1.29E-03
Phvul.007G007000	no data	0.50	4.57E-01	0.68	1.36E-01	0.49	5.09E-01	1.01	2.09E-02
Phvul.006G058600	PTHR13301//PTHR13301:SF54 - X-BOX TRANSCRIPTION FACTOR-RELATED // SUBFAMILY NOT NAMED (1 of 6)	0.50	3.23E-01	0.55	1.45E-01	0.20	7.94E-01	0.78	3.61E-02
Phvul.006G137000	PTHR31964:SF10 - ADENINE NUCLEOTIDE ALPHA HYDROLASES-LIKE SUPERFAMILY PROTEIN (1 of 3)	0.50	5.50E-01	1.40	6.32E-03	0.39	7.06E-01	1.19	2.92E-02
Phvul.006G198200	PF00069//PF08263//PF13855 - Protein kinase domain (Pkinase) // Leucine rich repeat N-terminal domain (LRRNT 2) //	0.50	3.35E-01	1.26	3.11E-04	0.15	8.48E-01	0.91	1.66E-02
Phvul.009G234500	KOG0617 - Ras suppressor protein (contains leucine-rich repeats) (1 of 30)	0.50	7.04E-01	1.59	3.30E-02	0.56	6.91E-01	1.16	1.46E-01

Phvul.004G129600	PTHR33107:SF1 - DR4 PROTEIN-RELATED (1 of 9)	0.50	7.55E-01	-2.06	2.04E-02	0.00	9.99E-01	-1.38	1.44E-01
Phvul.007G034700	PTHR11206:SF87 - MATE EFFLUX FAMILY PROTEIN-RELATED (1 of 5)	0.50	7.93E-02	0.59	7.80E-03	0.46	1.44E-01	0.34	1.70E-01
Phvul.011G041100	no data	0.49	1.80E-01	0.51	7.63E-02	0.66	5.97E-02	0.84	2.80E-03
Phvul.011G119700	PTHR11746//PTHR11746:SF83 - O-METHYLTRANSFERASE // SUBFAMILY NOT NAMED (1 of 9)	0.49	2.75E-01	1.31	2.07E-05	0.43	4.07E-01	0.94	4.25E-03
Phvul.006G170200	PTHR19375:SF229 - HEAT SHOCK 70 KDA PROTEIN 5 (1 of 3)	0.49	3.10E-01	0.86	1.10E-02	0.54	2.96E-01	1.19	4.06E-04
Phvul.009G154400	KOG2712 - Transcriptional coactivator (1 of 1)	0.49	1.19E-02	0.13	5.56E-01	0.35	1.60E-01	0.10	6.43E-01
Phvul.002G291200	PF01388 - ARID/BRIGHT DNA binding domain (ARID) (1 of 13)	0.49	4.58E-02	0.10	6.97E-01	0.22	5.39E-01	-0.14	5.91E-01
Phvul.009G137600	K03093 - RNA polymerase sigma factor (SIGMA70) (1 of 3)	0.49	2.42E-01	0.55	8.67E-02	0.53	2.33E-01	0.88	4.96E-03
Phvul.003G061100	PF07816 - Protein of unknown function (DUF1645) (DUF1645) (1 of 14)	0.49	8.16E-02	0.60	7.17E-03	0.40	2.26E-01	0.45	6.23E-02
Phvul.010G043100	2.7.11.1 - Non-specific serine/threonine protein kinase / Threonine-specific protein kinase (1 of 1198)	0.49	1.85E-01	0.94	3.49E-04	0.39	3.79E-01	0.90	9.41E-04
Phvul.003G162600	PTHR12547:SF70 - ZINC FINGER CCCH DOMAIN-CONTAINING PROTEIN 39 (1 of 1)	0.49	5.65E-01	2.56	4.21E-05	0.00	9.98E-01	1.98	4.73E-03
Phvul.007G093600	PTHR31942:SF29 - MLO-LIKE PROTEIN 12-RELATED (1 of 3)	0.49	5.06E-01	1.27	6.57E-03	0.38	6.57E-01	1.07	3.04E-02
Phvul.001G210800	PTHR31875:SF5 - PROTEIN DEHYDRATION-INDUCED 19 HOMOLOG 2-RELATED (1 of 1)	0.49	5.00E-02	0.42	4.44E-02	0.46	9.57E-02	0.51	1.65E-02
Phvul.001G126700	PTHR10891:SF573 - CALCIUM-BINDING PROTEIN CML24-RELATED (1 of 4)	0.49	8.28E-02	0.75	5.98E-04	0.83	5.28E-04	0.65	4.47E-03
Phvul.003G240900	PTHR31282:SF33 - WRKY TRANSCRIPTION FACTOR 15-RELATED (1 of 2)	0.49	1.37E-01	0.79	1.23E-03	0.53	1.21E-01	0.75	3.23E-03
Phvul.001G126200	K13354 - solute carrier family 25 (peroxisomal adenine nucleotide transporter), member 17 (SLC25A17, PMP34) (1 of 2)	0.49	7.24E-02	0.49	2.34E-02	0.34	3.14E-01	0.40	8.33E-02
Phvul.011G116600	PTHR31301:SF19 - LOB DOMAIN-CONTAINING PROTEIN 2 (1 of 1)	0.49	4.85E-01	1.14	1.00E-02	0.02	9.91E-01	1.01	3.04E-02
Phvul.007G245700	no data	0.49	2.24E-02	0.31	1.20E-01	0.48	3.63E-02	0.37	6.24E-02
Phvul.003G186500	K18211 - synaptosomal-associated protein 25 (SNAP25) (1 of 3)	0.48	1.30E-01	0.76	1.71E-03	0.57	7.32E-02	0.95	6.60E-05
Phvul.007G211800	PTHR10641:SF494 - TRANSCRIPTION FACTOR MYB108 (1 of 3)	0.48	6.94E-01	1.09	1.44E-01	0.76	5.15E-01	1.83	7.52E-03
Phvul.003G134000	PTHR10052//PTHR10052:SF11 - 60S RIBOSOMAL PROTEIN L18A // SUBFAMILY NOT NAMED (1 of 2)	0.48	1.02E-01	0.59	1.05E-02	0.22	5.96E-01	0.38	1.35E-01
Phvul.010G124800	PTHR22835:SF262 - GDSL ESTERASE/LIPASE 5-RELATED (1 of 3)	0.48	4.10E-01	1.23	1.12E-03	0.16	8.52E-01	0.61	1.60E-01
Phvul.001G221200	PTHR31238:SF39 - GERMIN-LIKE PROTEIN SUBFAMILY 2 MEMBER 1-RELATED (1 of 3)	0.48	1.32E-01	1.01	1.54E-05	0.32	4.27E-01	0.69	6.23E-03
Phvul.002G140600	no data	0.48	2.02E-01	0.57	4.52E-02	0.45	2.80E-01	0.52	7.97E-02
Phvul.L002060	4.1.2.5 - L-threonine aldolase / L-threonine acetaldehyde-lyase (1 of 2)	0.48	2.89E-01	0.66	4.46E-02	0.46	3.52E-01	0.53	1.21E-01
Phvul.011G097200	KOG0725 - Reductases with broad range of substrate specificities (1 of 51)	0.48	1.39E-01	0.73	2.86E-03	0.62	4.72E-02	0.53	4.50E-02
Phvul.006G194100	PTHR27002:SF84 - ATP BINDING / PROTEIN KINASE-RELATED (1 of 5)	0.48	1.27E-01	0.79	8.73E-04	0.45	2.01E-01	0.52	4.44E-02
Phvul.001G181400	PTHR23086:SF42 - PHOSPHATIDYLINOSITOL 4-PHOSPHATE 5-KINASE 9 (1 of 2)	0.48	3.80E-01	0.97	6.20E-03	0.49	3.95E-01	0.75	4.70E-02
Phvul.006G028000	no data	0.48	7.18E-02	0.45	3.90E-02	0.26	4.50E-01	0.29	2.22E-01
Phvul.011G003100	PTHR27003:SF38 - SERINE/THREONINE-KINASE-LIKE PROTEIN CCR4 (1 of 1)	0.47	3.30E-01	0.70	4.96E-02	0.42	4.40E-01	0.62	9.42E-02
Phvul.008G281100	PF13855 - Leucine rich repeat (LRR 8) (1 of 311)	0.47	5.44E-01	1.26	6.32E-03	0.72	3.29E-01	1.06	3.23E-02
Phvul.002G231000	PF14111 - Domain of unknown function (DUF4283) (DUF4283) (1 of 81)	0.47	3.16E-01	0.66	4.46E-02	0.54	2.64E-01	0.96	3.14E-03
Phvul.002G195400	no data	0.47	2.78E-01	0.53	9.99E-02	0.55	2.19E-01	0.64	4.81E-02
Phvul.011G136130	K14488 - SAUR family protein (SAUR) (1 of 75)	0.47	3.47E-01	0.75	2.44E-02	0.14	8.59E-01	0.44	2.35E-01
Phvul.002G166900	PTHR31218:SF53 - PROTEIN WALLS ARE THIN 1 (1 of 3)	0.47	3.02E-01	0.65	4.19E-02	0.32	5.67E-01	0.46	1.79E-01
Phvul.010G011400	PTHR24298:SF86 - CYTOCHROME P450 71B21-RELATED (1 of 10)	0.47	1.36E-01	0.57	1.77E-02	0.36	3.31E-01	0.56	2.48E-02
Phvul.009G188333	PTHR20856:SF20 - DNA-DIRECTED RNA POLYMERASE SUBUNIT BETA (1 of 2)	0.47	6.83E-01	-0.66	3.62E-01	0.18	9.14E-01	-1.38	4.59E-02
Phvul.010G162700	K13065 - shikimate O-hydroxycinnamoyltransferase (E2.3.1.133, HCT) (1 of 15)	0.47	7.17E-01	1.60	3.28E-02	0.39	7.99E-01	1.47	5.87E-02
Phvul.009G089600	no data	0.47	1.29E-01	0.53	2.65E-02	0.45	1.83E-01	0.54	3.26E-02
Phvul.011G150400	PTHR27002:SF9 - CYSTEINE-RICH RECEPTOR-LIKE PROTEIN KINASE 27-RELATED (1 of 4)	0.47	6.57E-01	0.63	4.01E-01	0.20	8.95E-01	1.60	1.25E-02
Phvul.002G032700	PTHR10286:SF10 - F11O4.12 (1 of 3)	0.47	1.29E-02	0.48	3.57E-03	0.36	1.05E-01	0.25	1.78E-01
Phvul.007G143900	PTHR24223:SF246 - ABC TRANSPORTER C FAMILY MEMBER 14-RELATED (1 of 4)	0.47	3.59E-01	0.83	1.63E-02	0.41	4.77E-01	0.81	2.56E-02
Phvul.005G007100	PTHR27000:SF52 - LEUCINE-RICH REPEAT (LRR) FAMILY PROTEIN-RELATED (1 of 1)	0.46	2.54E-01	0.67	2.37E-02	0.40	3.94E-01	0.69	2.38E-02
Phvul.002G166000	PTHR23070//PTHR23070:SF12 - BCS1 AAA-TYPE ATPASE // SUBFAMILY NOT NAMED (1 of 3)	0.46	5.29E-01	0.95	4.50E-02	0.48	5.37E-01	1.32	6.06E-03
Phvul.001G021600	PF13499 - EF-hand domain pair (EF-hand 7) (1 of 103)	0.46	3.94E-01	0.55	1.64E-01	0.77	1.19E-01	0.99	8.52E-03
Phvul.003G077000	PTHR10353:SF27 - BETA-GLUCOSIDASE 45-RELATED (1 of 4)	0.46	2.74E-01	0.64	3.37E-02	0.10	8.81E-01	0.38	2.53E-01
Phvul.002G231200	no data	0.46	1.20E-02	0.13	5.29E-01	0.27	2.76E-01	0.00	9.98E-01
Phvul.001G242400	PTHR34953:SF1 - ALPHA/BETA HYDROLASE RELATED PROTEIN (1 of 2)	0.46	4.52E-01	1.21	2.37E-03	0.21	8.05E-01	1.39	5.07E-04
Phvul.003G125700	PTHR33077:SF5 - PROTEIN TIFY 3A-RELATED (1 of 2)	0.46	3.33E-02	0.33	8.61E-02	0.33	2.22E-01	0.37	5.69E-02
Phvul.006G137600	PTHR23324//PTHR23324:SF79 - SEC14 RELATED PROTEIN // SUBFAMILY NOT NAMED (1 of 4)	0.46	1.92E-01	0.91	2.91E-04	0.29	5.11E-01	0.67	1.52E-02
Phvul.011G152100	PTHR27002:SF129 - INACTIVE G-TYPE LECTIN S-RECEPTOR-LIKE SERINE/THREONINE-PROTEIN KINASE SRK-RELATED (1 of	0.46	2.57E-01	0.64	2.73E-02	0.21	7.14E-01	0.44	1.71E-01
Phvul.002G032200	2.7.7.3 - Pantetheine-phosphate adenylyltransferase / PPAT (1 of 1)	0.46	3.22E-02	0.28	1.57E-01	0.45	5.32E-02	0.28	1.66E-01
Phvul.011G136700	no data	0.46	5.15E-01	0.93	2.84E-02	0.44	5.67E-01	0.86	5.50E-02
Phvul.001G019500	PTHR11802//PTHR11802:SF86 - SERINE PROTEASE FAMILY S10 SERINE CARBOXYPEPTIDASE // SUBFAMILY NOT NAMED	0.46	2.88E-01	0.72	1.14E-02	0.31	5.73E-01	0.69	2.07E-02
Phvul.009G110900	PTHR11614:SF96 - ALPHA/BETA FOLD HYDROLASE FAMILY PROTEIN (1 of 2)	0.46	7.98E-02	0.51	1.50E-02	0.55	3.47E-02	0.67	1.52E-03
Phvul.009G135500	PF00892 - EamA-like transporter family (EamA) (1 of 74)	0.46	2.95E-01	0.64	3.84E-02	0.23	6.88E-01	0.47	1.58E-01
Phvul.008G243300	PTHR11911 - INOSINE-5-MONOPHOSPHATE DEHYDROGENASE RELATED (1 of 6)	0.45	2.45E-01	0.45	1.42E-01	0.31	5.13E-01	0.61	4.46E-02
Phvul.002G322400	K10845 - TFIIH basal transcription factor complex TTD-A subunit (TTDA, GTF2H5, TFB5) (1 of 2)	0.45	2.47E-02	0.50	3.47E-03	0.23	4.03E-01	0.39	3.17E-02
Phvul.002G263800	PF00067 - Cytochrome P450 (p450) (1 of 273)	0.45	2.09E-01	0.87	5.58E-04	0.52	1.59E-01	0.65	1.68E-02
Phvul.003G054000	no data	0.45	4.77E-02	0.16	4.87E-01	0.40	1.18E-01	0.05	8.57E-01
Phvul.009G068100	PF00069//PF00954//PF01453 - Protein kinase domain (Pkinase) // S-locus glycoprotein domain (S locus glycop) // D-ma	0.45	3.43E-01	1.15	2.19E-04	0.44	3.94E-01	0.98	2.73E-03
Phvul.008G027000	PTHR13887 - GLUTATHIONE S-TRANSFERASE KAPPA (1 of 3)	0.45	1.36E-01	0.63	4.91E-03	0.37	3.01E-01	0.57	1.74E-02
Phvul.001G030800	no data	0.45	7.00E-01	1.41	3.27E-02	0.28	8.50E-01	1.14	9.94E-02
Phvul.004G020000	PTHR21493:SF148 - SENESCENCE-ASSOCIATED CARBOXYLESTERASE 101 (1 of 1)	0.45	1.78E-01	0.72	3.56E-03	0.44	2.28E-01	0.86	5.35E-04
Phvul.008G266300	PTHR22953:SF5 - PURPLE ACID PHOSPHATASE 18 (1 of 2)	0.45	1.89E-01	0.77	1.67E-03	0.19	7.08E-01	0.54	4.01E-02
Phvul.008G030700	PF07714//PF12819 - Protein tyrosine kinase (Pkinase Tyr) // Carbohydrate-binding protein of the ER (Malectin like) (1 c	0.45	3.61E-01	0.41	2.59E-01	0.47	3.72E-01	0.66	4.98E-02
Phvul.003G278700	no data	0.45	1.18E-01	0.46	4.33E-02	0.53	6.52E-02	0.36	1.34E-01
Phvul.006G214800	KOG4176 - Uncharacterized conserved protein (1 of 6)	0.45	2.74E-01	0.60	4.25E-02	0.28	5.87E-01	0.52	9.08E-02
Phvul.003G229200	no data	0.45	1.33E-01	0.48	3.82E-02	0.25	5.17E-01	0.27	2.86E-01
Phvul.008G230400	PTHR22950:SF48 - PROLINE TRANSPORTER 1-RELATED (1 of 2)	0.45	1.62E-01	0.51	4.49E-02	0.23	6.11E-01	0.04	9.17E-01
Phvul.009G087000	K16860 - phospholipase D3/4 (PLD3 4) (1 of 1)	0.44	3.21E-01	0.69	3.37E-02	0.34	5.23E-01	0.64	6.17E-02
Phvul.008G267000	no data	0.44	4.64E-01	0.46	2.91E-01	0.81	1.20E-01	0.85	3.44E-02
Phvul.007G230100	K15200 - general transcription factor 3C polypeptide 2 (GTF3C2) (1 of 2)	0.44	1.66E-01	0.59	1.35E-02	0.48	1.49E-01	0.82	6.52E-04
Phvul.005G091900	K08914 - light-harvesting complex II chlorophyll a/b binding protein 3 (LHCB3) (1 of 2)	0.44	2.68E-01	0.63	3.29E-02	0.65	7.69E-02	1.04	2.07E-04
Phvul.011G152300	PTHR27002:SF129 - INACTIVE G-TYPE LECTIN S-RECEPTOR-LIKE SERINE/THREONINE-PROTEIN KINASE SRK-RELATED (1 of	0.44	3.04E-01	1.03	3.22E-04	0.42	3.74E-01	0.85	4.98E-03
Phvul.007G015100	PF10238 - E2F-associated phosphoprotein (Eapp C) (1 of 1)	0.44	4.85E-02	0.07	7.72E-01	0.41	9.20E-02	-0.17	4.38E-01
Phvul.002G064232	no data	0.44	3.53E-01	0.76	1.71E-02	0.01	9.91E-01	0.25	5.23E-01
Phvul.004G028500	PTHR10641//PTHR10641:SF530 - MYB-LIKE DNA-BINDING PROTEIN MYB // SUBFAMILY NOT NAMED (1 of 2)	0.44	2.45E-01	0.57	4.72E-02	0.31	4.94E-01	0.72	1.11E-02
Phvul.002G288800	PTHR31500:SF9 - AT HOOK MOTIF DNA-BINDING FAMILY PROTEIN (1 of 2)	0.43	4.04E-02	0.40	2.40E-02	0.32	2.19E-01	0.27	1.55E-01
Phvul.001G232300	2.7.10.2//2.7.11.1 - Non-specific protein-tyrosine kinase / Cytoplasmic protein tyrosine kinase // Non-specific serine/thr	0.43	1.68E-01	0.59	1.29E-02	0.27	5.02E-01	0.55	2.83E-02
Phvul.006G148300	K13347 - peroxisomal membrane protein 2 (PXMP2, PMP22) (1 of 3)	0.43	8.42E-02	0.42	3.84E-02	0.35	2.34E-01	0.22	3.30E-01
Phvul.006G215500	2.7.11.1 - Non-specific serine/threonine protein kinase / Threonine-specific protein kinase (1 of 1198)	0.43	2.10E-01	0.68	6.77E-03	0.40	2.96E-01	0.58	2.81E-02
Phvul.003G042000	no data	0.43	4.61E-01	0.84	3.12E-02	0.90	5.42E-02	0.84	3.95E-02
Phvul.004G024700	PTHR23070:SF5 - AAA-TYPE ATPASE FAMILY PROTEIN-RELATED (1 of 2)	0.43	4.69E-01	1.48	6.15E-05	0.61	3.00E-01	1.73	3.25E-06
Phvul.005G038300	no data	0.43	8.19E-01	1.95	3.92E-02	1.31	3.53E-01	1.89	5.62E-02
Phvul.005G182100	PTHR10366//PTHR10366:SF467 - NAD DEPENDENT EPIMERASE/DEHYDRATASE // SUBFAMILY NOT NAMED (1 of 1)	0.43	2.73E-01	0.92	7.34E-04	0.49	2.34E-01	0.80	4.79E-03
Phvul.011G025100	1.5.1.43 - Carboxynorspermidine synthase / Carboxyspermidine dehydrogenase (1 of 1)	0.43	6.11E-01	1.57	1.44E-03	0.66	4.13E-01	1.42	6.33E-03
Phvul.009G062400	K04421 - mitogen-activated protein kinase kinase kinase 3 (MAP3K3, MEKK3) (1 of 2)	0.43	3.65E-01	0.92	3.62E-03	0.39	4.60E-01	0.98	2.77E-03
Phvul.009G210200	no data	0.43	2.35E-01	0.41	1.53E-01	0.39	3.31E-01	0.58	3.88E-02
Phvul.009G184500	PF00069//PF00560//PF08263 - Protein kinase domain (Pkinase) // Leucine Rich Repeat (LRR 1) // Leucine rich repeat N	0.43	6.22E-01	1.05	4.16E-02	0.70	3.92E-01	1.17	2.65E-02
Phvul.006G061100	PTHR22950:SF228 - AMINO ACID PERMEASE 7-RELATED (1 of 4)	0.43	3.22E-01	0.63	4.00E-02	0.33	5.21E-01	0.49	1.31E-01
Phvul.007G162600	PTHR23359:SF94 - ADENYLATE KINASE 1, MITOCHONDRIAL-RELATED (1 of 2)	0.43	1.08E-01	0.44	3.28E-02	0.24	4.99E-01	0.40	6.80E-02
Phvul.009G221800	PTHR33077:SF5 - PROTEIN TIFY 3A-RELATED (1 of 2)	0.43	1.62E-02	0.37	2.18E-02	0.25	3.04E-01	0.42	1.10E-02
Phvul.001G208300	PTHR10992:SF811 - ALPHA/BETA-HYDROLASES SUPERFAMILY PROTEIN (1 of 1)	0.43	2.13E-01	0.62	1.03E-02	0.37	3.55E-01	0.50	5.00E-02
Phvul.009G000600	3.2.2.16 - Methylthioadenosine nucleosidase / MTA nucleosidase (1 of 1)	0.43	6.03E-03	0.37	9.67E-03	0.28	1.68E-01	0.13	4.65E-01
Phvul.002G052400	PTHR11785//PTHR11785:SF366 - AMINO ACID TRANSPORTER // SUBFAMILY NOT NAMED (1 of 1)	0.43	2.23E-01	0.68	6.94E-03	0.41	2.97E-01	0.52	5.46E-02
Phvul.010G065200	PTHR33345:SF3 - GENOMIC DNA, CHROMOSOME 3, BAC CLONE:F16J14 (1 of 2)	0.43	2.12E-02	0.38	1.85E-02	0.41	4.34E-02	0.28	1.08E-01
Phvul.007G268800	PTHR31415:SF4 - HARPIN-INDUCED PROTEIN-LIKE-RELATED (1 of 9)	0.43	3.04E-01	0.54	7.27E-02	0.65	9.26E-02	0.71	1.65E-02
Phvul.009G027000	PTHR23054:SF27 - ELECTRON CARRIER/ PROTEIN DISULFIDE OXIDOREDUCTASE (1 of 2)	0.43	7.81E-02	0.58	2.26E-03	0.29	3.50E-01	0.68	4.05E-04
Phvul.002G318300	PTHR31713:SF15 - PROTEIN SAR DEFICIENT 1 (1 of 2)	0.43	5.62E-01	0.82	7.52E-02	0.78	2.44E-01	1.14	1.19E-02
Phvul.002G290900	3.4.11.14 - Cytosol alanyl aminopeptidase / Soluble alanyl aminopeptidase (1 of 2)	0.43	3.74E-01	0.57	8.85E-02	0.45	3.73E-01	0.70	3.70E-02
Phvul.007G263200	K03016 - DNA-directed RNA polymerases I, II, and III subunit RPABC3 (RPB8, POLR2H) (1 of 3)	0.43	2.05E-02	0.34	3.83E-02	0.26	2.86E-01	0.21	2.51E-01
Phvul.002G092500	PTHR13018:SF45 - EARLY-RESPONSIVE TO DEHYDRATION STRESS PROTEIN (ERD4) (1 of 1)	0.43	2.38E-01	0.85	6.91E-04	0.18	7.33E-01	0.50	6.95E-02

Phvul.003G015900	PF00069//PF00954 - Protein kinase domain (Pkinase) // S-locus glycoprotein domain (S locus glycop) (1 of 1)	0.43	2.65E-01	0.89	8.05E-04	0.52	1.83E-01	0.80	4.05E-03
Phvul.008G032501	2.3.1.115 - Isoflavone-7-O-beta-glucoside 6''-O-malonyltransferase / Flavone/flavonol 7-O-beta-D-glucoside malonyltran	0.43	7.93E-01	-2.60	4.93E-03	-0.34	8.71E-01	-2.11	2.59E-02
Phvul.001G075500	PTHR31717:SF2 - CCT MOTIF FAMILY PROTEIN (1 of 2)	0.43	4.56E-01	0.61	1.22E-01	0.49	4.10E-01	0.97	1.16E-02
Phvul.005G048700	2.7.7.6 - DNA-directed RNA polymerase / RNA polymerase III (1 of 46)	0.43	2.93E-01	0.66	2.01E-02	0.38	4.06E-01	0.32	3.26E-01
Phvul.009G126200	PTHR13683:SF316 - ASPARTYL PROTEASE FAMILY PROTEIN-RELATED (1 of 2)	0.42	2.83E-01	0.60	3.21E-02	0.33	4.76E-01	0.59	3.99E-02
Phvul.005G110900	PF00954//PF01453//PF07714//PF13947 - S-locus glycoprotein domain (S locus glycop) // D-mannose binding lectin (B le	0.42	1.44E-01	0.52	2.00E-02	0.40	2.21E-01	0.57	1.23E-02
Phvul.008G153700	PF07714//PF08263//PF13855 - Protein tyrosine kinase (Pkinase Tyr) // Leucine rich repeat N-terminal domain (LRRNT 2	0.42	7.73E-02	0.45	1.71E-02	0.29	3.37E-01	0.19	4.05E-01
Phvul.003G210800	PTHR22953:SF24 - INACTIVE PURPLE ACID PHOSPHATASE 24-RELATED (1 of 1)	0.42	1.40E-01	0.46	4.02E-02	0.04	9.46E-01	0.27	2.74E-01
Phvul.003G050500	no data	0.42	2.89E-01	0.35	2.66E-01	0.55	1.60E-01	0.63	3.23E-02
Phvul.011G045500	PF03087 - Arabidopsis protein of unknown function (DUF241) (1 of 23)	0.42	3.70E-01	0.26	5.11E-01	0.61	1.75E-01	0.75	2.39E-02
Phvul.001G259700	K09775 - hypothetical protein (K09775) (1 of 3)	0.42	1.09E-01	0.57	3.92E-03	0.45	1.06E-01	0.61	2.91E-03
Phvul.011G209200	PTHR10502//PTHR10502:SF113 - ANNEXIN // SUBFAMILY NOT NAMED (1 of 5)	0.42	2.45E-01	0.79	2.05E-03	0.38	3.52E-01	0.37	2.08E-01
Phvul.008G140500	PTHR31251:SF15 - SQUAMOSA PROMOTER-BINDING-LIKE PROTEIN 3 (1 of 2)	0.42	7.51E-01	1.81	1.07E-02	0.83	4.74E-01	2.05	5.32E-03
Phvul.005G009300	PTHR23500:SF30 - SUGAR TRANSPORT PROTEIN 3 (1 of 2)	0.42	3.84E-01	1.09	5.29E-04	0.32	5.76E-01	1.11	5.89E-04
Phvul.004G120700	1.14.11.20 - Deacetoxyvindoline 4-hydroxylase / Desacetoxyvindoline-17-hydroxylase (1 of 14)	0.42	3.34E-01	0.65	2.94E-02	0.20	7.33E-01	0.50	1.15E-01
Phvul.004G140700	PF05729//PF13676 - NACHT domain (NACHT) // TIR domain (TIR 2) (1 of 5)	0.42	3.74E-01	0.81	8.70E-03	0.38	4.65E-01	0.85	7.76E-03
Phvul.008G042600	5.1.3.3 - Aldose 1-epimerase / Mutarotase (1 of 4)	0.41	7.23E-02	0.49	7.86E-03	0.33	2.35E-01	0.41	3.43E-02
Phvul.002G234500	PTHR11934:SF7 - RIBOSE-5-PHOSPHATE ISOMERASE 1-RELATED (1 of 2)	0.41	1.59E-01	0.60	6.21E-03	0.40	2.26E-01	0.45	5.38E-02
Phvul.002G231700	K01094 - phosphatidylglycerophosphatase GEP4 (GEP4) (1 of 2)	0.41	4.43E-01	0.76	4.15E-02	0.62	2.26E-01	0.89	1.71E-02
Phvul.002G256500	K11842 - ubiquitin carboxyl-terminal hydrolase 12/46 [EC:3.4.19.12] (USP12 46) (1 of 2)	0.41	2.62E-01	0.70	8.29E-03	0.46	2.42E-01	1.01	8.86E-05
Phvul.009G125900	PTHR31719:SF3 - GRAB1-LIKE PROTEIN (1 of 3)	0.41	1.32E-01	0.72	4.16E-04	0.74	1.31E-03	0.52	1.78E-02
Phvul.004G119600	PTHR10015:SF168 - HEAT STRESS TRANSCRIPTION FACTOR B-2A (1 of 2)	0.41	2.02E-01	0.46	6.01E-02	0.57	6.24E-02	0.66	6.32E-03
Phvul.001G180200	PTHR31096:SF23 - ACT DOMAIN-CONTAINING PROTEIN (1 of 2)	0.41	3.93E-01	0.89	4.68E-03	0.37	4.88E-01	0.77	2.16E-02
Phvul.007G024700	PTHR21576//PTHR21576:SF40 - UNCHARACTERIZED NODULIN-LIKE PROTEIN // SUBFAMILY NOT NAMED (1 of 4)	0.41	1.71E-01	0.50	2.72E-02	0.47	1.26E-01	0.58	1.15E-02
Phvul.010G117500	K14488 - SAUR family protein (SAUR) (1 of 75)	0.41	5.20E-01	1.10	3.96E-03	0.19	8.32E-01	0.99	1.52E-02
Phvul.002G055700	PTHR31190:SF30 - ETHYLENE-RESPONSIVE TRANSCRIPTION FACTOR 15-RELATED (1 of 3)	0.41	2.09E-01	0.69	3.15E-03	0.70	1.35E-02	0.68	5.09E-03
Phvul.010G038500	PF16029 - Domain of unknown function (DUF4787) (DUF4787) (1 of 1)	0.41	4.57E-02	0.23	2.21E-01	0.27	2.98E-01	-0.05	8.51E-01
Phvul.007G157500	PTHR24320:SF81 - NAD(P)-BINDING ROSSMANN-FOLD SUPERFAMILY PROTEIN (1 of 5)	0.41	9.01E-02	0.43	2.65E-02	0.25	4.19E-01	0.22	3.05E-01
Phvul.002G207900	PTHR10984//PTHR10984:SF35 - ENDOPLASMIC RETICULUM-GOLGI INTERMEDIATE COMPARTMENT PROTEIN // SUBFAN	0.41	7.70E-02	0.39	4.00E-02	0.21	5.05E-01	0.09	7.01E-01
Phvul.005G079733	PF13912 - C2H2-type zinc finger (zf-C2H2 6) (1 of 42)	0.41	4.36E-01	0.73	3.26E-02	0.30	6.43E-01	0.48	1.96E-01
Phvul.002G090511	K02963 - small subunit ribosomal protein S18 (RP-S18, MRPS18, rpsR) (1 of 1)	0.41	2.85E-01	-0.62	2.39E-02	-0.03	9.73E-01	-0.66	1.98E-02
Phvul.011G071400	PTHR11177//PTHR11177:SF186 - CHITINASE // SUBFAMILY NOT NAMED (1 of 1)	0.41	2.36E-01	0.60	1.53E-02	0.36	3.50E-01	0.63	1.48E-02
Phvul.009G239800	K04730 - interleukin-1 receptor-associated kinase 1 (IRAK1) (1 of 24)	0.41	1.57E-01	0.58	6.46E-03	0.31	3.59E-01	0.63	3.97E-03
Phvul.003G105100	PTHR10638:SF36 - AMINE OXIDASE-RELATED (1 of 3)	0.40	1.23E-01	0.45	2.78E-02	0.26	4.33E-01	0.39	6.56E-02
Phvul.008G004900	no data	0.40	4.40E-02	-0.02	9.45E-01	0.47	1.84E-02	-0.05	8.15E-01
Phvul.004G051700	PTHR10910:SF95 - DOUBLE-STRANDED RNA-BINDING PROTEIN 4 (1 of 1)	0.40	4.51E-02	0.13	5.42E-01	0.32	1.73E-01	0.23	2.19E-01
Phvul.006G086600	PTHR24073:SF570 - RAS-RELATED PROTEIN RABF2A-RELATED (1 of 1)	0.40	3.99E-02	0.02	9.40E-01	0.23	3.79E-01	0.07	7.38E-01
Phvul.008G178300	PTHR31376:SF1 - PURINE PERMEASE 1-RELATED (1 of 2)	0.40	3.48E-01	0.77	7.57E-03	0.80	2.16E-02	0.88	2.91E-03
Phvul.006G015900	no data	0.40	2.73E-01	0.48	7.58E-02	0.40	3.20E-01	0.56	4.01E-02
Phvul.011G119730	no data	0.40	1.31E-01	0.52	8.58E-03	0.27	4.16E-01	0.19	4.17E-01
Phvul.003G036200	PF07797 - Protein of unknown function (DUF1639) (DUF1639) (1 of 8)	0.40	2.36E-01	0.67	5.84E-03	0.31	4.39E-01	0.75	2.53E-03
Phvul.007G008900	KOG0502//KOG0508//KOG0512//KOG4412 - Integral membrane ankyrin-repeat protein Kidins220 (protein kinase D sub	0.40	4.43E-01	0.69	4.81E-02	0.45	4.07E-01	0.72	4.75E-02
Phvul.003G275000	PTHR10891:SF574 - CALCIUM-BINDING PROTEIN CML30-RELATED (1 of 5)	0.40	2.33E-01	0.51	3.84E-02	0.36	3.37E-01	0.42	1.12E-01
Phvul.007G039300	PTHR11926:SF167 - UDP-GLYCOSYLTRANSFERASE 84A1-RELATED (1 of 2)	0.40	4.46E-01	0.73	3.40E-02	0.52	3.19E-01	1.08	1.56E-03
Phvul.004G135800	K07407 - alpha-galactosidase (E3.2.1.22B, galA, rafA) (1 of 3)	0.40	1.36E-01	0.31	1.71E-01	0.33	2.83E-01	0.48	2.48E-02
Phvul.007G049300	PTHR32099:SF4 - CYSTEINE-RICH REPEAT SECRETORY PROTEIN 1-RELATED (1 of 5)	0.40	6.72E-01	1.49	2.99E-03	0.70	4.11E-01	1.56	2.73E-03
Phvul.009G033900	K10664 - E3 ubiquitin-protein ligase ATL6/9/15/31/42/55 [EC:6.3.2.19] (ATL6S) (1 of 10)	0.40	5.36E-01	1.02	5.76E-03	0.21	8.07E-01	0.86	2.92E-02
Phvul.001G226500	PTHR10071//PTHR10071:SF178 - TRANSCRIPTION FACTOR GATA GATA BINDING FACTOR // SUBFAMILY NOT NAMED (1	0.40	4.85E-01	0.44	2.96E-01	0.39	5.28E-01	0.79	4.08E-02
Phvul.009G187700	PTHR10641:SF641 - MYB FAMILY TRANSCRIPTION FACTOR (1 of 3)	0.39	3.78E-01	0.76	8.89E-03	0.25	6.61E-01	0.82	5.93E-03
Phvul.003G095700	PF08122 - NADH-ubiquinone oxidoreductase B12 subunit family (NDUF B12) (1 of 1)	0.39	5.45E-01	0.89	2.57E-02	0.79	1.35E-01	1.25	9.84E-04
Phvul.003G123900	PTHR10666:SF150 - POLYUBIQUITIN 3 (1 of 1)	0.39	2.38E-01	0.47	6.03E-02	0.50	1.29E-01	0.56	2.62E-02
Phvul.008G266100	PTHR12668:SF0 - UPF0136 MEMBRANE PROTEIN CG5532 (1 of 1)	0.39	3.98E-02	0.30	7.39E-02	0.27	2.52E-01	0.22	2.19E-01
Phvul.011G166500	PF00197 - Trypsin and protease inhibitor (Kunitz legume) (1 of 24)	0.39	6.61E-01	1.19	2.11E-02	0.34	7.40E-01	0.80	1.56E-01
Phvul.006G111400	K14506 - jasmonic acid-amino synthetase (JAR1) (1 of 2)	0.39	3.74E-01	0.44	1.75E-01	0.73	4.85E-02	0.63	4.25E-02
Phvul.L001650	PTHR32278:SF2 - F-BOX PROTEIN PP2-B13-RELATED (1 of 4)	0.39	4.13E-01	0.68	3.07E-02	0.30	6.00E-01	0.63	5.57E-02
Phvul.004G148200	K01772 - ferrochelatase (hemH, FECH) (1 of 4)	0.39	3.00E-01	0.70	6.27E-03	0.36	3.89E-01	0.61	2.39E-02
Phvul.002G221200	PTHR23500:SF55 - SUGAR TRANSPORT PROTEIN 1-RELATED (1 of 2)	0.39	4.35E-01	0.81	1.21E-02	0.45	3.88E-01	1.08	9.90E-04
Phvul.004G100200	3.2.1.15 - Polygalacturonase / Pectinase (1 of 57)	0.39	7.24E-01	2.15	4.51E-03	0.70	4.77E-01	2.54	6.39E-04
Phvul.005G049000	PTHR11040//PTHR11040:SF59 - ZINC/IRON TRANSPORTER // SUBFAMILY NOT NAMED (1 of 1)	0.39	3.37E-01	0.89	8.94E-04	0.43	3.13E-01	0.81	3.68E-03
Phvul.007G255600	PTHR32060:SF5 - PEPTIDASE S41 FAMILY PROTEIN (1 of 1)	0.38	3.57E-01	0.57	5.12E-02	0.43	3.31E-01	0.69	2.04E-02
Phvul.010G111400	K14506 - jasmonic acid-amino synthetase (JAR1) (1 of 2)	0.38	2.17E-01	0.56	1.44E-02	0.23	5.53E-01	0.45	6.39E-02
Phvul.003G149400	K11097 - small nuclear ribonucleoprotein E (SNRPE, SME) (1 of 2)	0.38	2.55E-02	0.21	1.95E-01	0.25	2.51E-01	0.05	8.03E-01
Phvul.009G122800	PTHR11751//PTHR11751:SF317 - SUBGROUP I AMINOTRANSFERASE RELATED // SUBFAMILY NOT NAMED (1 of 2)	0.38	3.80E-01	0.54	2.87E-02	-0.02	9.90E-01	0.25	4.01E-01
Phvul.006G043000	1.8.4.11 - Peptide-methionine (S)-S-oxide reductase / Peptide methionine sulfoxide reductase (1 of 4)	0.38	1.79E-01	0.51	1.66E-02	0.36	2.62E-01	0.59	6.47E-03
Phvul.009G176300	no data	0.38	2.49E-01	0.49	4.84E-02	0.14	7.87E-01	0.12	7.04E-01
Phvul.006G040800	K13065 - shikimate O-hydroxycinnamoyltransferase (E2.3.1.133, HCT) (1 of 15)	0.38	3.90E-01	0.42	1.96E-01	0.56	1.86E-01	0.68	3.03E-02
Phvul.008G223400	PTHR31213:SF9 - ABSCISIC ACID RECEPTOR PYL5 (1 of 2)	0.38	2.82E-01	0.71	4.06E-03	0.50	1.49E-01	0.79	2.01E-03
Phvul.002G160700	PTHR11972:SF69 - RESPIRATORY BURST OXIDASE HOMOLOG PROTEIN F-RELATED (1 of 1)	0.38	6.70E-01	1.29	1.09E-02	0.18	8.77E-01	1.19	2.66E-02
Phvul.007G215400	PTHR23241//PTHR23241:SF55 - LATE EMBRYOGENESIS ABUNDANT PLANTS LEA-RELATED // SUBFAMILY NOT NAMED (	0.38	5.74E-02	0.36	2.78E-02	0.24	3.46E-01	0.07	7.34E-01
Phvul.003G240200	PTHR10641:SF551 - MYB TRANSCRIPTION FACTOR (1 of 3)	0.38	2.97E-01	0.59	2.06E-02	0.53	1.25E-01	0.50	6.64E-02
Phvul.002G274500	no data	0.38	7.57E-02	0.42	1.49E-02	0.27	3.04E-01	0.39	2.91E-02
Phvul.008G068200	K15095 - (+)-neomenthol dehydrogenase (E1.1.1.208) (1 of 10)	0.38	2.03E-01	0.67	1.61E-03	0.35	2.86E-01	0.58	9.91E-03
Phvul.003G105000	PTHR11926:SF152 - UDP-GLUCOSYL TRANSFERASE 73B2-RELATED (1 of 5)	0.38	4.97E-01	0.76	3.42E-02	0.23	7.40E-01	0.50	2.02E-01
Phvul.011G169600	PTHR14155 - RING FINGER DOMAIN-CONTAINING (1 of 159)	0.38	5.42E-01	0.96	1.24E-02	0.17	8.42E-01	0.84	3.69E-02
Phvul.009G055700	PTHR12313//PTHR12313:SF10 - RNF5 // SUBFAMILY NOT NAMED (1 of 1)	0.37	2.45E-01	0.52	2.76E-02	0.26	5.03E-01	0.43	8.60E-02
Phvul.006G132000	2.4.1.221 - Peptide-O-fucosyltransferase / GDP-L-fucose:polypeptide fucosyltransferase (1 of 4)	0.37	3.13E-01	0.79	1.57E-03	0.38	3.46E-01	0.77	3.16E-03
Phvul.003G097300	PF00004 - ATPase family associated with various cellular activities (AAA) (AAA) (1 of 68)	0.37	3.68E-01	0.39	1.99E-01	0.85	8.46E-03	0.54	7.30E-02
Phvul.009G157800	PTHR24343:SF161 - SERINE/THREONINE-PROTEIN KINASE SRK2J (1 of 1)	0.37	1.74E-01	0.71	3.29E-04	0.35	2.54E-01	0.78	9.45E-05
Phvul.011G127600	PTHR31165:SF10 - PROTEIN LIGHT-DEPENDENT SHORT HYPOCOTYLS 10-RELATED (1 of 2)	0.37	2.85E-01	0.30	2.72E-01	0.51	1.27E-01	0.52	4.35E-02
Phvul.009G054050	PTHR12755:SF3 - POLYNUCLEOTIDE 5'-HYDROXYL-KINASE NOL9 (1 of 2)	0.37	6.16E-01	1.07	1.05E-02	-0.07	9.58E-01	0.31	5.77E-01
Phvul.L006443	PTHR21717:SF9 - PROTEIN TBF1 (1 of 4)	0.37	3.72E-01	0.70	1.26E-02	-0.11	8.61E-01	0.47	1.31E-01
Phvul.004G048300	4.2.1.122 - Tryptophan synthase (indole-salvaging) / Tryptophan synthase beta-2 (1 of 1)	0.37	1.72E-01	0.54	8.47E-03	0.41	1.49E-01	0.60	4.66E-03
Phvul.008G276300	PF08263 - Leucine rich repeat N-terminal domain (LRRNT 2) (1 of 284)	0.37	5.92E-01	1.29	1.29E-03	0.02	9.86E-01	1.34	1.32E-03
Phvul.002G092800	PF14009 - Domain of unknown function (DUF4228) (DUF4228) (1 of 38)	0.37	6.64E-01	1.09	3.81E-02	0.10	9.42E-01	0.30	6.74E-01
Phvul.003G063900	no data	0.37	7.50E-02	0.34	4.26E-02	0.15	6.23E-01	0.34	5.38E-02
Phvul.004G040200	2.3.1.115 - Isoflavone-7-O-beta-glucoside 6''-O-malonyltransferase / Flavone/flavonol 7-O-beta-D-glucoside malonyltran	0.37	6.04E-01	0.99	1.73E-02	-0.23	8.11E-01	0.96	2.72E-02
Phvul.009G220700	PF03106 - WRKY DNA -binding domain (WRKY) (1 of 91)	0.37	4.50E-01	0.70	2.85E-02	0.12	8.64E-01	0.88	6.06E-03
Phvul.003G177500	K03136 - transcription initiation factor TFIIE subunit alpha (TFIIE1, GTF2E1, TFA1, tfe) (1 of 2)	0.37	4.64E-02	0.31	4.67E-02	0.27	2.33E-01	0.20	2.43E-01
Phvul.008G275500	PF03514 - GRAS domain family (GRAS) (1 of 55)	0.37	3.86E-01	0.86	1.78E-03	0.49	2.48E-01	0.82	4.05E-03
Phvul.002G086200	PTHR12725:SF55 - F14L17.7 PROTEIN (1 of 2)	0.37	9.25E-02	0.15	4.89E-01	0.44	3.91E-02	0.17	4.09E-01
Phvul.011G151200	PTHR11926:SF253 - UDP-GLYCOSYLTRANSFERASE 89B1 (1 of 2)	0.37	4.02E-01	0.95	6.73E-04	0.58	1.58E-01	0.87	3.09E-03
Phvul.009G143100	PTHR28039:SF4 - CHALCONE--FLAVONONE ISOMERASE 3-RELATED (1 of 1)	0.36	3.91E-01	0.69	1.57E-02	0.26	6.17E-01	0.66	3.04E-02
Phvul.003G057301	no data	0.36	1.98E-01	0.56	5.96E-03	0.38	2.25E-01	0.58	7.00E-03
Phvul.006G154900	PTHR33739:SF5 - MEDIATOR OF RNA POLYMERASE II TRANSCRIPTION SUBUNIT 33A (1 of 3)	0.36	5.76E-01	0.88	2.80E-02	0.23	7.85E-01	0.75	7.84E-02
Phvul.003G134100	PTHR24361:SF385 - MITOGEN-ACTIVATED PROTEIN KINASE KINASE 1-RELATED (1 of 2)	0.36	3.43E-01	0.61	2.20E-02	0.21	6.65E-01	0.44	1.26E-01
Phvul.007G222200	no data	0.36	2.88E-01	0.36	1.76E-01	0.64	2.90E-02	0.51	4.75E-02
Phvul.001G129600	PF00122//PF00689//PF12710 - E1-E2 ATPase (E1-E2 ATPase) // Cation transporting ATPase, C-terminus (Cation ATPase	0.36	3.01E-01	0.65	6.99E-03	0.19	6.74E-01	0.52	4.29E-02
Phvul.008G032200	2.3.1.115 - Isoflavone-7-O-beta-glucoside 6''-O-malonyltransferase / Flavone/flavonol 7-O-beta-D-glucoside malonyltran	0.36	7.15E-01	1.05	6.18E-02	0.52	6.03E-01	1.34	1.60E-02
Phvul.002G119300	PTHR31651:SF5 - AUXIN EFFLUX CARRIER FAMILY PROTEIN-RELATED (1 of 2)	0.36	4.70E-01	0.69	3.64E-02	0.17	8.10E-01	0.38	3.00E-01
Phvul.011G006500	K08343 - ubiquitin-like-conjugating enzyme ATG3 (ATG3) (1 of 1)	0.36	2.57E-01	0.29	2.65E-01	0.36	3.04E-01	0.50	3.72E-02
Phvul.008G195400	PTHR11003//PTHR11003:SF140 - POTASSIUM CHANNEL, SUBFAMILY K // SUBFAMILY NOT NAMED (1 of 1)	0.36	1.03E-01	0.42	1.55E-02	0.39	8.58E-02	0.29	1.25E-01
Phvul.011G160500	PTHR20953:SF2 - PROTEIN SEEDLING PLASTID DEVELOPMENT 1 (1 of 2)	0.36	2.68E-01	0.36	1.52E-01	0.21	6.21E-01	0.50	4.23E-02
Phvul.006G208300	K18823 - soyasaponin III rhamnosyltransferase (UGT91H4) (1 of 1)	0.36	8.48E-01	-2.10	3.12E-02	-0.09	9.75E-01	-1.66	9.93E-02

Phvul.002G131900	PTHR16007 - EPIDIDYMAL MEMBRANE PROTEIN E9-RELATED (1 of 9)	0.36	4.71E-01	0.69	3.40E-02	0.20	7.70E-01	0.54	1.17E-01
Phvul.009G067000	PTHR12864//PTHR12864:SF23 - RAN BINDING PROTEIN 9-RELATED // SUBFAMILY NOT NAMED (1 of 2)	0.36	2.03E-01	0.56	5.91E-03	0.19	6.12E-01	0.44	4.17E-02
Phvul.001G192800	PF00076 - RNA recognition motif. (a.k.a. RRM, RBD, or RNP domain) (RRM 1) (1 of 252)	0.36	2.88E-01	0.49	3.57E-02	0.23	5.85E-01	0.18	5.39E-01
Phvul.011G064000	K16283 - E3 ubiquitin-protein ligase SDIR1 [EC:6.3.2.19] (SDIR1) (1 of 2)	0.36	1.13E-01	0.47	7.43E-03	0.32	2.12E-01	0.35	5.97E-02
Phvul.009G240677	PF13962 - Domain of unknown function (PGG) (1 of 69)	0.36	7.00E-01	1.10	3.92E-02	0.42	6.66E-01	1.15	4.07E-02
Phvul.007G144400	PTHR11062:SF112 - GLYCOSYLTRANSFERASE FAMILY PROTEIN 47 (1 of 1)	0.36	4.59E-01	0.84	6.79E-03	0.45	3.52E-01	0.73	2.57E-02
Phvul.004G012500	1.4.3.16 - L-aspartate oxidase / LASPO (1 of 1)	0.35	4.24E-01	0.99	4.15E-04	0.67	7.54E-02	1.01	4.71E-04
Phvul.006G204800	KOG0028 - Ca2+-binding protein (centrin/caltractin), EF-Hand superfamily protein (1 of 5)	0.35	3.56E-02	0.21	1.83E-01	0.28	1.75E-01	0.23	1.32E-01
Phvul.001G065100	PTHR24078//PTHR24078:SF278 - DNAJ HOMOLOG SUBFAMILY C MEMBER // SUBFAMILY NOT NAMED (1 of 1)	0.35	3.28E-01	0.68	5.05E-03	0.38	3.29E-01	0.66	8.33E-03
Phvul.004G125100	PTHR32099:SF1 - CYSTEINE-RICH RECEPTOR-LIKE PROTEIN KINASE 9-RELATED (1 of 5)	0.35	6.56E-01	0.95	3.76E-02	0.75	2.71E-01	0.70	1.62E-01
Phvul.003G072500	PTHR12565:SF107 - TRANSCRIPTION FACTOR BPE (1 of 1)	0.35	3.25E-01	0.50	4.40E-02	0.20	6.79E-01	0.61	1.35E-02
Phvul.007G234300	K11838 - ubiquitin carboxyl-terminal hydrolase 7 (USP7, UBP15) (1 of 4)	0.35	2.88E-01	0.57	1.43E-02	0.40	2.50E-01	0.41	1.00E-01
Phvul.003G112000	PTHR11711//PTHR11711:SF165 - ADP RIBOSYLATION FACTOR-RELATED // SUBFAMILY NOT NAMED (1 of 1)	0.35	1.41E-01	0.41	2.96E-02	0.28	3.30E-01	0.36	7.03E-02
Phvul.005G116000	PF03106 - WRKY DNA -binding domain (WRKY) (1 of 91)	0.35	4.71E-01	0.82	8.30E-03	0.35	5.11E-01	0.72	2.83E-02
Phvul.001G069280	3.1.4.46 - Glycerophosphodiester phosphodiesterase / Glycerophosphoryl diester phosphodiesterase (1 of 65)	0.35	5.04E-01	0.78	1.83E-02	0.20	7.75E-01	0.62	8.03E-02
Phvul.007G211600	PTHR33669:SF1 - PROTEIN NIM1-INTERACTING 1 (1 of 2)	0.35	8.59E-01	2.64	2.51E-03	0.29	9.01E-01	3.32	1.30E-04
Phvul.L002132	PTHR11730:SF39 - AMMONIUM TRANSPORTER 1 MEMBER 2 (1 of 2)	0.35	4.50E-01	0.43	2.01E-01	0.53	2.36E-01	0.69	3.09E-02
Phvul.003G276000	K14961 - COMPASS component SWD1 (RBBP5, SWD1, CPS50) (1 of 3)	0.35	4.37E-02	0.07	7.42E-01	0.24	2.84E-01	0.27	8.56E-02
Phvul.001G079400	PTHR24089:SF294 - CALCIUM-BINDING TRANSPORTER-LIKE PROTEIN (1 of 3)	0.35	5.38E-01	0.54	2.18E-01	0.45	4.29E-01	1.10	4.94E-03
Phvul.001G212800	PTHR22952//PTHR22952:SF167 - CAMP-RESPONSE ELEMENT BINDING PROTEIN-RELATED // SUBFAMILY NOT NAMED (1	0.35	1.93E-01	0.51	9.92E-03	0.43	1.19E-01	0.41	5.70E-02
Phvul.008G026800	PTHR13887 - GLUTATHIONE S-TRANSFERASE KAPPA (1 of 3)	0.35	2.23E-01	0.46	2.74E-02	0.26	4.57E-01	0.61	3.58E-03
Phvul.008G288000	PTHR22811:SF50 - TRANSMEMBRANE EMP24 DOMAIN-CONTAINING PROTEIN P24BETA2 (1 of 2)	0.35	3.86E-02	0.28	6.12E-02	0.26	2.05E-01	0.18	2.78E-01
Phvul.008G187600	PTHR31851:SF16 - MEMBRANE PROTEIN OF ER BODY 2 (1 of 4)	0.35	4.45E-01	0.78	9.95E-03	0.41	3.93E-01	0.54	1.01E-01
Phvul.007G029200	PF07797 - Protein of unknown function (DUF1639) (DUF1639) (1 of 8)	0.35	2.01E-01	0.25	2.57E-01	0.50	4.66E-02	0.33	1.35E-01
Phvul.001G057300	3.2.1.39 - Glucan endo-1,3-beta-D-glucosidase / Laminarinase (1 of 65)	0.35	2.12E-01	0.56	4.93E-03	0.39	1.78E-01	0.61	3.16E-03
Phvul.L001751	PTHR17204//PTHR17204:SF30 - PRE-MRNA PROCESSING PROTEIN PRP39-RELATED // SUBFAMILY NOT NAMED (1 of 2)	0.35	4.84E-01	0.76	1.49E-02	0.30	5.94E-01	0.87	7.01E-03
Phvul.006G185400	PTHR24067:SF97 - UBIQUITIN-CONJUGATING ENZYME E2 24-RELATED (1 of 3)	0.35	1.87E-01	0.51	9.68E-03	0.20	5.62E-01	0.43	3.61E-02
Phvul.008G127237	PTHR19359:SF26 - CYTOCHROME B5 ISOFORM B (1 of 2)	0.35	5.28E-01	0.78	2.04E-02	0.31	6.27E-01	0.57	1.15E-01
Phvul.008G243200	4.1.1.8 - Oxalyl-CoA decarboxylase / Oxalyl-CoA carboxy-lyase (1 of 1)	0.35	2.90E-01	0.60	8.76E-03	0.31	4.06E-01	0.63	8.16E-03
Phvul.010G132200	PTHR11017//PTHR11017:SF163 - LEUCINE-RICH REPEAT-CONTAINING PROTEIN // SUBFAMILY NOT NAMED (1 of 62)	0.35	5.03E-01	0.87	6.89E-03	0.05	9.54E-01	0.56	1.10E-01
Phvul.007G164800	PTHR33448:SF4 - ARABIDOPSIS THALIANA GENOMIC DNA, CHROMOSOME 5, P1 CLONE:MOK16-RELATED (1 of 2)	0.34	5.59E-01	0.16	7.55E-01	0.51	3.68E-01	0.78	4.08E-02
Phvul.008G195900	no data	0.34	4.30E-01	0.62	2.94E-02	0.26	6.16E-01	0.60	4.44E-02
Phvul.011G000200	1.14.13.173 - 11-oxo-beta-amyrin 30-oxidase / CYP72A154 (1 of 17)	0.34	7.91E-01	-1.68	1.60E-02	-0.14	9.42E-01	-0.99	1.91E-01
Phvul.011G071300	PTHR31319:SF1 - CCT MOTIF FAMILY PROTEIN (1 of 2)	0.34	1.32E-01	0.42	1.72E-02	0.51	1.56E-02	0.36	5.75E-02
Phvul.003G296800	KOG1032 - Uncharacterized conserved protein, contains GRAM domain (1 of 5)	0.34	3.44E-01	0.60	1.26E-02	0.10	8.59E-01	0.31	2.65E-01
Phvul.001G147300	K14487 - auxin responsive GH3 gene family (GH3) (1 of 11)	0.34	8.10E-01	1.57	3.47E-02	-0.34	8.43E-01	0.79	3.60E-01
Phvul.011G099100	PTHR22849:SF0 - WD REPEAT, SAM AND U-BOX DOMAIN-CONTAINING PROTEIN 1 (1 of 2)	0.34	3.59E-01	0.52	4.39E-02	0.37	3.49E-01	0.63	1.63E-02
Phvul.001G163300	PTHR13763 - BREAST CANCER TYPE 1 SUSCEPTIBILITY PROTEIN BRCA1 (1 of 2)	0.34	4.49E-01	0.66	2.60E-02	0.17	7.77E-01	0.42	1.96E-01
Phvul.007G257200	PTHR24073:SF578 - RAS-RELATED PROTEIN RABA4D (1 of 2)	0.34	6.11E-01	0.88	2.81E-02	0.27	7.31E-01	0.93	2.36E-02
Phvul.009G180300	2.4.1.15//3.1.3.12 - Alpha,alpha-trehalose-phosphate synthase (UDP-forming) / UDP-glucose--glucose-phosphate glucos	0.34	6.42E-01	0.75	9.86E-02	0.34	6.79E-01	0.90	4.63E-02
Phvul.008G098600	KOG2209 - Oxysterol-binding protein (1 of 3)	0.34	4.63E-01	0.68	2.38E-02	0.35	4.87E-01	0.69	2.87E-02
Phvul.006G216100	PTHR12832 - TESTIS-SPECIFIC PROTEIN PBS13 T-COMPLEX 11 (1 of 1)	0.34	4.28E-01	0.60	3.61E-02	0.36	4.30E-01	0.82	4.38E-03
Phvul.010G096100	PTHR12203:SF28 - DOWNSTREAM TARGET OF AGL15 2 (1 of 1)	0.34	7.00E-01	1.50	2.22E-03	0.11	9.32E-01	1.37	7.19E-03
Phvul.007G219500	PTHR12899//PTHR12899:SF4 - 39S RIBOSOMAL PROTEIN L18, MITOCHONDRIAL // SUBFAMILY NOT NAMED (1 of 1)	0.34	6.94E-01	0.99	4.84E-02	0.47	5.83E-01	1.41	4.67E-03
Phvul.002G322700	PF01535//PF13041//PF14432 - PPR repeat (PPR) // PPR repeat family (PPR 2) // DYW family of nucleic acid deaminases	0.34	5.94E-01	0.95	1.14E-02	0.60	2.92E-01	0.99	1.18E-02
Phvul.002G241800	no data	0.34	5.35E-01	0.68	4.48E-02	0.38	5.05E-01	0.86	1.20E-02
Phvul.009G255300	no data	0.33	5.08E-01	0.26	5.10E-01	0.50	3.02E-01	0.78	1.95E-02
Phvul.008G148700	PF00560//PF07714//PF08263 - Leucine Rich Repeat (LRR 1) // Protein tyrosine kinase (Pkinase Tyr) // Leucine rich repe	0.33	3.50E-01	0.64	8.27E-03	0.16	7.45E-01	0.56	2.80E-02
Phvul.008G062200	PTHR27003:SF52 - U-BOX DOMAIN-CONTAINING PROTEIN 33 (1 of 3)	0.33	3.84E-01	0.56	3.39E-02	0.30	4.88E-01	0.64	1.80E-02
Phvul.003G213200	2.3.3.10 - Hydroxymethylglutaryl-CoA synthase / Hydroxymethylglutaryl coenzyme alpha-condensing enzyme (1 of 3)	0.33	4.60E-01	0.71	1.49E-02	0.26	6.27E-01	0.52	9.99E-02
Phvul.010G054400	PF01582 - TIR domain (TIR) (1 of 38)	0.33	8.02E-01	2.10	1.81E-03	0.60	6.28E-01	1.89	7.60E-03
Phvul.006G131400	PTHR15140 - TUBULIN-SPECIFIC CHAPERONE E (1 of 1)	0.33	2.85E-01	0.66	2.00E-03	0.35	2.96E-01	0.56	1.41E-02
Phvul.002G169600	no data	0.33	4.11E-01	0.77	3.50E-03	0.20	6.95E-01	0.64	2.41E-02
Phvul.010G101800	PTHR11926:SF152 - UDP-GLUCOSYL TRANSFERASE 73B2-RELATED (1 of 5)	0.33	8.67E-01	-2.58	8.65E-03	0.03	9.93E-01	-1.83	7.47E-02
Phvul.003G143000	no data	0.33	3.19E-01	0.51	2.74E-02	0.31	4.04E-01	0.37	1.37E-01
Phvul.001G264132	PTHR18934//PTHR18934:SF120 - ATP-DEPENDENT RNA HELICASE // SUBFAMILY NOT NAMED (1 of 3)	0.33	6.41E-01	0.66	1.42E-01	0.62	3.46E-01	0.90	4.23E-02
Phvul.002G001400	PTHR23291:SF34 - BAX INHIBITOR 1 (1 of 2)	0.33	6.77E-02	0.33	2.39E-02	0.27	2.04E-01	0.30	5.63E-02
Phvul.002G164000	2.7.8.11//6.2.1.3 - CDP-diacylglycerol--inositol 3-phosphatidyltransferase / Phosphatidylinositol synthase // Long-chain-f	0.33	3.58E-01	0.54	2.94E-02	0.32	4.13E-01	0.62	1.41E-02
Phvul.005G103200	PTHR27007:SF9 - L-TYPE LECTIN-DOMAIN CONTAINING RECEPTOR KINASE VII.1-RELATED (1 of 4)	0.33	3.54E-01	0.75	1.34E-03	0.23	5.91E-01	0.67	6.91E-03
Phvul.010G127400	PTHR33077:SF13 - PROTEIN TIFY 10A-RELATED (1 of 4)	0.33	2.12E-01	0.25	2.59E-01	0.36	1.90E-01	0.56	5.37E-03
Phvul.004G079000	K12580 - CCR4-NOT transcription complex subunit 3 (CNOT3, NOT3) (1 of 2)	0.33	1.61E-01	0.42	1.98E-02	0.18	5.48E-01	0.40	3.27E-02
Phvul.002G283700	PTHR10791:SF22 - BIDIRECTIONAL SUGAR TRANSPORTER SWEET10 (1 of 5)	0.33	4.84E-01	0.64	3.59E-02	0.30	5.63E-01	0.61	5.59E-02
Phvul.003G061300	K02732 - 20S proteasome subunit beta 6 (PSMB1) (1 of 1)	0.33	3.32E-01	0.62	6.96E-03	0.25	5.30E-01	0.50	4.22E-02
Phvul.008G060600	PTHR22937//PTHR22937:SF24 - RING FINGER CONTAINING PROTEIN // SUBFAMILY NOT NAMED (1 of 2)	0.33	3.96E-01	0.52	4.70E-02	0.23	6.37E-01	0.45	1.01E-01
Phvul.003G223624	K09286 - EREBP-like factor (EREBP) (1 of 35)	0.33	3.16E-01	0.73	9.51E-04	0.59	3.47E-02	0.88	5.54E-05
Phvul.011G169900	2.4.2.14 - Amidophosphoribosyltransferase / Phosphoribosyldiphosphate 5-amidotransferase (1 of 3)	0.33	5.66E-01	1.02	2.75E-03	0.45	4.20E-01	0.75	4.46E-02
Phvul.002G209200	1.10.3.11 - Ubiquinol oxidase (non-electrogenic) / Ubiquinol oxidase (1 of 4)	0.33	6.16E-01	0.84	3.40E-02	0.55	3.59E-01	1.14	4.57E-03
Phvul.003G122400	PTHR24221:SF205 - ABC TRANSPORTER B FAMILY MEMBER 11-RELATED (1 of 8)	0.33	7.00E-01	0.64	2.40E-01	0.27	7.87E-01	1.09	3.28E-02
Phvul.008G223800	K02987 - small subunit ribosomal protein S4e (RP-S4e, RPS4) (1 of 4)	0.33	3.99E-01	0.37	1.94E-01	0.32	4.48E-01	0.54	4.87E-02
Phvul.009G117400	no data	0.33	6.56E-01	0.97	5.40E-03	0.21	8.21E-01	0.46	2.74E-01
Phvul.002G289300	PTHR15141:SF38 - BROMO-ADJACENT HOMOLOGY (BAH) DOMAIN-CONTAINING PROTEIN-RELATED (1 of 3)	0.32	3.21E-01	0.39	1.04E-01	0.41	2.21E-01	0.52	3.02E-02
Phvul.008G075300	PTHR33601:SF1 - LITTLE ZIPPER 1 PROTEIN-RELATED (1 of 2)	0.32	4.62E-01	0.39	2.18E-01	0.24	6.51E-01	0.76	9.60E-03
Phvul.001G135900	no data	0.32	3.90E-01	0.53	4.11E-02	0.10	8.65E-01	0.32	2.58E-01
Phvul.004G146900	PTHR10502//PTHR10502:SF120 - ANNEXIN // SUBFAMILY NOT NAMED (1 of 2)	0.32	2.89E-01	0.62	3.14E-03	0.29	4.06E-01	0.64	3.80E-03
Phvul.003G225800	PTHR24343//PTHR24343:SF168 - SERINE/THREONINE KINASE // SUBFAMILY NOT NAMED (1 of 3)	0.32	2.83E-01	0.48	2.67E-02	0.28	4.08E-01	0.51	2.04E-02
Phvul.002G182900	K04730 - interleukin-1 receptor-associated kinase 1 (IRAK1) (1 of 24)	0.32	2.94E-01	0.49	2.11E-02	0.42	1.62E-01	0.54	1.44E-02
Phvul.001G009900	PTHR12411//PTHR12411:SF365 - CYSTEINE PROTEASE FAMILY C1-RELATED // SUBFAMILY NOT NAMED (1 of 1)	0.32	4.76E-01	0.45	1.54E-01	0.44	3.30E-01	0.61	4.99E-02
Phvul.006G034000	K12450 - UDP-glucose 4,6-dehydratase (RHM) (1 of 3)	0.32	8.80E-01	-2.35	2.46E-02	-0.32	8.94E-01	-1.85	8.50E-02
Phvul.008G126000	PTHR27002:SF84 - ATP BINDING / PROTEIN KINASE-RELATED (1 of 5)	0.32	2.16E-01	-0.10	6.97E-01	0.10	8.07E-01	-0.44	3.56E-02
Phvul.003G223686	PTHR31190:SF30 - ETHYLENE-RESPONSIVE TRANSCRIPTION FACTOR 15-RELATED (1 of 3)	0.32	4.19E-01	0.81	1.53E-03	0.57	9.77E-02	0.69	1.12E-02
Phvul.001G134100	PTHR23155//PTHR23155:SF563 - LEUCINE-RICH REPEAT-CONTAINING PROTEIN // SUBFAMILY NOT NAMED (1 of 29)	0.32	7.09E-01	0.94	4.28E-02	0.26	8.04E-01	0.74	1.37E-01
Phvul.008G035500	PTHR13169:SF4 - MEMBRANE-ANCHORED UBIQUITIN-FOLD PROTEIN 1-RELATED (1 of 2)	0.32	3.72E-01	0.56	2.07E-02	0.17	7.29E-01	0.38	1.49E-01
Phvul.009G101200	PTHR22937//PTHR22937:SF53 - RING FINGER CONTAINING PROTEIN // SUBFAMILY NOT NAMED (1 of 2)	0.32	4.60E-01	0.65	1.96E-02	0.38	3.85E-01	0.76	8.26E-03
Phvul.003G075400	5.2.1.13 - Prolycopene isomerase / CRTISO (1 of 2)	0.32	3.21E-01	0.56	1.14E-02	-0.08	8.81E-01	0.20	4.55E-01
Phvul.007G031100	PF07714//PF13947//PF14380 - Protein tyrosine kinase (Pkinase Tyr) // Wall-associated receptor kinase galacturonan-bi	0.32	3.57E-01	0.61	7.72E-03	0.32	3.90E-01	0.42	8.95E-02
Phvul.009G255900	no data	0.32	5.34E-01	0.34	3.59E-01	0.63	1.51E-01	0.66	4.91E-02
Phvul.002G288700	PTHR11592:SF27 - GLUTATHIONE PEROXIDASE 8-RELATED (1 of 2)	0.31	3.54E-01	0.61	7.57E-03	0.37	3.01E-01	0.59	1.35E-02
Phvul.007G147500	no data	0.31	4.88E-01	0.24	5.10E-01	0.32	5.13E-01	0.61	4.98E-02
Phvul.005G156900	K15718 - linoleate 9S-lipoxygenase (LOX1 5) (1 of 19)	0.31	8.49E-01	-1.89	2.13E-02	-0.15	9.44E-01	-0.86	3.63E-01
Phvul.007G082500	K04730 - interleukin-1 receptor-associated kinase 1 (IRAK1) (1 of 24)	0.31	3.68E-01	0.54	2.05E-02	0.47	1.52E-01	0.70	2.58E-03
Phvul.002G133800	no data	0.31	5.38E-01	0.59	7.11E-02	0.31	5.80E-01	0.66	4.87E-02
Phvul.009G208400	PTHR12136//PTHR12136:SF49 - STEROIDOGENIC ACUTE REGULATORY PROTEIN STAR // SUBFAMILY NOT NAMED (1 of	0.31	4.07E-01	-0.57	2.94E-02	0.17	7.37E-01	-0.44	1.06E-01
Phvul.007G208800	PTHR31734:SF15 - AUXIN-RESPONSIVE PROTEIN IAA20-RELATED (1 of 2)	0.31	7.60E-01	1.59	2.53E-03	0.33	7.73E-01	1.32	1.87E-02
Phvul.010G070100	no data	0.31	2.78E-01	0.79	4.04E-05	0.33	2.80E-01	0.77	8.86E-05
Phvul.002G131800	PTHR22599//PTHR22599:SF26 - MPS ONE BINDER KINASE ACTIVATOR-LIKE MOB // SUBFAMILY NOT NAMED (1 of 1)	0.31	1.79E-01	0.47	6.14E-03	0.24	3.87E-01	0.38	3.45E-02
Phvul.002G011400	PTHR24093:SF262 - CALCIUM-TRANSPORTING ATPASE 2, PLASMA MEMBRANE-TYPE-RELATED (1 of 3)	0.31	3.87E-01	0.63	8.24E-03	0.32	4.16E-01	0.77	1.64E-03
Phvul.005G064900	PTHR32212:SF126 - EMB (1 of 24)	0.31	6.04E-01	0.74	4.39E-02	0.16	8.36E-01	0.65	8.98E-02
Phvul.007G002500	PTHR12276:SF45 - CLATHRIN INTERACTOR 1 (1 of 2)	0.31	4.34E-01	0.56	3.30E-02	0.25	5.81E-01	0.57	3.61E-02
Phvul.007G272600	PTHR23042:SF72 - TRANSCRIPTION FACTOR BHLH104-RELATED (1 of 1)	0.31	1.33E-01	0.32	4.97E-02	0.13	6.76E-01	0.25	1.48E-01
Phvul.010G123200	K00814 - alanine transaminase (GPT, ALT) (1 of 1)	0.31	2.83E-01	0.41	4.82E-02	0.22	5.33E-01	0.20	4.11E-01
Phvul.009G212100	K10088 - protein OS-9 (OS9) (1 of 1)	0.31	2.63E-01	0.41	3.82E-02	0.29	3.43E-01	0.52	1.05E-02
Phvul.006G011200	PTHR32166:SF33 - HAT FAMILY DIMERIZATION DOMAIN-CONTAINING PROTEIN (1 of 6)	0.31	3.84E-01	0.32	2.28E-01	0.33	3.90E-01	0.53	3.62E-02
Phvul.004G140800	PTHR11017//PTHR11017:SF171 - LEUCINE-RICH REPEAT-CONTAINING PROTEIN // SUBFAMILY NOT NAMED (1 of 15)	0.31	6.65E-01	1.14	4.47E-03	0.23	7.93E-01	1.01	1.78E-02

Phvul.004G143800	PTHR10217:SF475 - CYCLIC NUCLEOTIDE-GATED ION CHANNEL 19-RELATED (1 of 6)	0.31	3.49E-01	0.49	3.26E-02	0.12	8.05E-01	0.39	1.09E-01
Phvul.002G247800	K17255 - Rab GDP dissociation inhibitor (GDI1 2) (1 of 4)	0.31	2.74E-01	0.56	4.85E-03	0.24	4.60E-01	0.23	3.20E-01
Phvul.006G216400	PTHR24343:SF101 - SERINE/THREONINE-PROTEIN KINASE SRK2A-RELATED (1 of 3)	0.31	1.98E-01	0.41	2.07E-02	0.11	7.56E-01	0.20	3.30E-01
Phvul.002G131000	PTHR23155//PTHR23155:SF505 - LEUCINE-RICH REPEAT-CONTAINING PROTEIN // SUBFAMILY NOT NAMED (1 of 23)	0.31	3.72E-01	0.48	3.37E-02	0.14	7.72E-01	0.54	1.91E-02
Phvul.001G131150	K05643 - ATP-binding cassette, subfamily A (ABC1), member 3 (ABCA3) (1 of 1)	0.31	3.65E-01	0.60	7.90E-03	0.15	7.41E-01	0.55	2.02E-02
Phvul.001G018800	PTHR23155//PTHR23155:SF543 - LEUCINE-RICH REPEAT-CONTAINING PROTEIN // SUBFAMILY NOT NAMED (1 of 10)	0.30	3.21E-01	0.51	1.53E-02	0.36	2.55E-01	0.47	3.37E-02
Phvul.008G260900	PTHR14493:SF54 - ZINC FINGER CCCH DOMAIN-CONTAINING PROTEIN 66 (1 of 1)	0.30	3.92E-01	0.54	2.57E-02	0.25	5.45E-01	0.62	1.23E-02
Phvul.007G106000	PF11961 - Domain of unknown function (DUF3475) (DUF3475) (1 of 15)	0.30	3.23E-01	0.41	6.16E-02	0.24	5.12E-01	0.48	3.28E-02
Phvul.011G202900	PTHR23155//PTHR23155:SF414 - LEUCINE-RICH REPEAT-CONTAINING PROTEIN // SUBFAMILY NOT NAMED (1 of 124)	0.30	5.76E-01	0.79	2.01E-02	0.24	7.14E-01	0.27	5.26E-01
Phvul.008G233400	PF01535//PF13041 - PPR repeat (PPR) // PPR repeat family (PPR 2) (1 of 197)	0.30	3.54E-01	0.66	2.57E-03	0.34	3.15E-01	0.78	3.83E-04
Phvul.005G036000	no data	0.30	6.39E-01	0.80	3.90E-02	0.29	6.82E-01	0.94	1.53E-02
Phvul.001G255500	PTHR11452:SF14 - ALPHA-GALACTOSIDASE A (1 of 3)	0.30	2.16E-01	0.41	2.00E-02	0.31	2.31E-01	0.43	1.96E-02
Phvul.003G272500	K14457 - 2-acylglycerol O-acyltransferase 2 (MOGAT2, MGAT2) (1 of 3)	0.30	4.46E-01	0.55	3.49E-02	0.10	8.59E-01	0.46	9.40E-02
Phvul.006G045800	PTHR22835//PTHR22835:SF116 - ZINC FINGER FYVE DOMAIN CONTAINING PROTEIN // SUBFAMILY NOT NAMED (1 of 5)	0.30	2.37E-01	0.42	2.41E-02	0.16	6.38E-01	0.44	2.39E-02
Phvul.006G011300	PTHR24012:SF478 - GENOMIC DNA, CHROMOSOME 3, P1 CLONE: MJH23 (1 of 2)	0.30	5.52E-01	0.58	7.34E-02	0.25	6.64E-01	0.74	2.39E-02
Phvul.002G027000	PTHR10804:SF87 - XAA-PRO AMINOPEPTIDASE 1 (1 of 1)	0.30	2.26E-01	0.38	3.93E-02	0.17	6.06E-01	0.32	1.02E-01
Phvul.011G064400	K07025 - putative hydrolase of the HAD superfamily (K07025) (1 of 3)	0.30	4.39E-01	0.49	6.13E-02	0.19	7.11E-01	0.67	9.38E-03
Phvul.008G061700	PTHR34797:SF1 - EXPRESSED PROTEIN (1 of 1)	0.30	3.95E-01	0.41	1.00E-01	0.32	3.90E-01	0.57	2.15E-02
Phvul.003G288000	K14962 - COMPASS component SWD2 (WDR82, SWD2, CPS35) (1 of 3)	0.30	3.29E-01	0.42	4.77E-02	0.17	6.64E-01	0.29	2.14E-01
Phvul.005G006800	PTHR30560//PTHR30560:SF4 - TRIGGER FACTOR CHAPERONE AND PEPTIDYL-PROLYL CIS/TRANS ISOMERASE // SUBFAM	0.30	2.87E-01	-0.37	7.95E-02	0.06	8.94E-01	-0.60	3.58E-03
Phvul.011G155400	PF05875 - Ceramidase (Ceramidase) (1 of 5)	0.30	2.44E-01	0.39	3.73E-02	0.22	4.76E-01	0.30	1.31E-01
Phvul.003G056700	K11644 - paired amphipathic helix protein Sin3a (SIN3A) (1 of 3)	0.30	4.92E-01	0.75	7.80E-03	-0.05	9.52E-01	0.56	6.48E-02
Phvul.003G047900	PTHR11514//PTHR11514:SF57 - MYC // SUBFAMILY NOT NAMED (1 of 2)	0.30	6.89E-01	1.59	1.10E-04	0.35	6.61E-01	1.20	5.75E-03
Phvul.006G156900	K15813 - beta-amyrin synthase (LUP4) (1 of 8)	0.30	6.35E-01	1.18	9.60E-04	0.30	6.66E-01	0.95	1.30E-02
Phvul.007G050600	PF01657//PF07714 - Salt stress response/antifungal (Stress-antifung) // Protein tyrosine kinase (Pkinase Tyr) (1 of 40)	0.30	6.10E-01	0.82	1.59E-02	0.07	9.44E-01	0.49	1.95E-01
Phvul.003G205000	K17987 - next to BRCA1 gene 1 protein (NBR1) (1 of 1)	0.30	2.03E-01	0.43	1.29E-02	0.26	3.30E-01	0.40	2.80E-02
Phvul.005G113200	PTHR31717:SF11 - B-BOX TYPE ZINC FINGER-CONTAINING PROTEIN (1 of 3)	0.30	8.28E-01	1.30	4.11E-02	0.89	4.04E-01	1.13	9.04E-02
Phvul.001G095300	PTHR27001:SF120 - PROTEIN LYK2 (1 of 1)	0.30	5.20E-01	0.86	2.16E-03	0.43	3.31E-01	0.76	1.02E-02
Phvul.009G240644	K15135 - mediator of RNA polymerase II transcription subunit 18 (MED18) (1 of 1)	0.29	2.29E-01	0.40	2.82E-02	0.25	3.82E-01	0.21	2.96E-01
Phvul.001G077100	PTHR11909:SF73 - PROTEIN KINASE FAMILY PROTEIN (1 of 2)	0.29	3.61E-01	0.58	7.27E-03	0.25	4.92E-01	0.67	2.33E-03
Phvul.011G143800	no data	0.29	6.16E-01	-0.82	1.93E-02	-0.08	9.36E-01	-0.43	2.75E-01
Phvul.002G091600	PTHR14580:SF0 - MULTIPLE MYELOMA TUMOR-ASSOCIATED PROTEIN 2 (1 of 1)	0.29	1.30E-01	0.22	1.70E-01	0.26	2.45E-01	0.32	3.95E-02
Phvul.006G136600	PTHR10774//PTHR10774:SF90 - EXTENDED SYNAPTOTAGMIN-RELATED // SUBFAMILY NOT NAMED (1 of 4)	0.29	4.58E-01	0.70	4.73E-03	0.33	4.16E-01	0.48	7.51E-02
Phvul.007G092800	no data	0.29	1.32E-01	0.41	4.57E-03	0.24	2.71E-01	0.31	4.98E-02
Phvul.003G240300	PTHR13847:SF150 - OXIDOREDUCTASE TDA3-RELATED (1 of 1)	0.29	3.58E-01	0.44	4.31E-02	0.37	2.31E-01	0.43	5.43E-02
Phvul.010G076000	PTHR31500:SF15 - AT HOOK MOTIF DNA-BINDING FAMILY PROTEIN (1 of 2)	0.28	6.35E-01	0.78	2.60E-02	0.25	7.21E-01	0.60	1.07E-01
Phvul.005G173000	PTHR31734:SF34 - AUXIN-RESPONSIVE PROTEIN IAA15 (1 of 1)	0.28	7.64E-01	0.79	1.52E-01	0.40	6.80E-01	1.08	4.29E-02
Phvul.007G191400	PTHR24356:SF164 - SERINE/THREONINE PROTEIN KINASE-RELATED (1 of 2)	0.28	2.12E-01	0.41	1.24E-02	-0.02	9.76E-01	0.20	2.79E-01
Phvul.011G063000	PTHR26374:SF192 - C2H2 AND C2HC ZINC FINGERS SUPERFAMILY PROTEIN-RELATED (1 of 1)	0.28	3.79E-01	0.49	2.25E-02	0.18	6.61E-01	0.66	2.40E-03
Phvul.006G208100	K13416 - brassinosteroid insensitive 1-associated receptor kinase 1 (BAK1) (1 of 3)	0.28	5.13E-01	0.56	4.35E-02	0.23	6.55E-01	0.60	3.50E-02
Phvul.002G260500	PTHR21327:SF11 - MONOFUNCTIONAL RIBOFLAVIN BIOSYNTHESIS PROTEIN RIBA 2, CHLOROPLASTIC (1 of 1)	0.28	5.35E-01	0.84	2.50E-03	0.44	2.94E-01	0.91	1.36E-03
Phvul.003G106800	PTHR10110:SF117 - SODIUM/HYDROGEN EXCHANGER 1-RELATED (1 of 4)	0.28	1.24E-01	0.10	5.66E-01	0.35	4.74E-02	0.19	2.25E-01
Phvul.006G189200	PTHR10799:SF705 - SNF2 , HELICASE AND ZINC-FINGER DOMAIN-CONTAINING PROTEIN-RELATED (1 of 2)	0.28	3.88E-01	0.51	2.00E-02	0.20	6.06E-01	0.35	1.42E-01
Phvul.001G069600	PF13947 - Wall-associated receptor kinase galacturonan-binding (GUB WAK bind) (1 of 65)	0.28	7.72E-01	1.20	1.91E-02	0.29	7.89E-01	1.36	9.64E-03
Phvul.005G073200	no data	0.28	6.05E-01	0.76	1.95E-02	0.40	4.53E-01	0.92	5.93E-03
Phvul.007G263700	no data	0.28	3.60E-01	0.44	4.00E-02	0.08	8.59E-01	0.32	1.68E-01
Phvul.004G156110	K04733 - interleukin-1 receptor-associated kinase 4 (IRAK4) (1 of 48)	0.28	5.54E-01	0.62	3.34E-02	0.30	5.58E-01	0.66	2.84E-02
Phvul.002G086150	K02608 - origin recognition complex subunit 6 (ORC6) (1 of 1)	0.28	8.41E-01	-0.61	4.67E-01	-0.50	7.11E-01	-1.79	1.71E-02
Phvul.002G098800	K07542 - phosphatidylinositol glycan, class V (PIGV) (1 of 2)	0.28	5.42E-01	0.57	5.00E-02	0.21	6.98E-01	0.72	1.45E-02
Phvul.001G045400	PTHR27000:SF188 - LRR RECEPTOR-LIKE SERINE/THREONINE-PROTEIN KINASE ERECTA (1 of 5)	0.28	6.96E-01	1.24	1.43E-03	0.07	9.51E-01	1.38	5.20E-04
Phvul.011G172100	PTHR23155//PTHR23155:SF554 - LEUCINE-RICH REPEAT-CONTAINING PROTEIN // SUBFAMILY NOT NAMED (1 of 22)	0.27	7.89E-01	0.79	1.77E-01	-0.56	5.97E-01	1.21	2.48E-02
Phvul.006G171700	PTHR10015:SF161 - E2F/DP FAMILY WINGED-HELIX DNA-BINDING DOMAIN-CONTAINING PROTEIN-RELATED (1 of 2)	0.27	4.21E-01	0.73	7.29E-04	0.40	2.22E-01	0.68	2.44E-03
Phvul.008G003600	K00616 - transaldolase (E2.2.1.2, talA, talB) (1 of 3)	0.27	4.59E-01	0.51	3.67E-02	0.16	7.46E-01	0.23	4.17E-01
Phvul.009G062100	PTHR12383:SF15 - THYLAKOIDAL PROCESSING PEPTIDASE 1, CHLOROPLASTIC-RELATED (1 of 1)	0.27	2.49E-01	0.35	3.87E-02	0.25	3.27E-01	0.22	2.44E-01
Phvul.004G104600	PTHR31683:SF8 - MAJOR POLLEN ALLERGEN-LIKE PROTEIN (1 of 4)	0.27	1.94E-01	0.48	1.31E-03	0.21	3.95E-01	0.40	1.24E-02
Phvul.002G031400	PF04564//PF05804 - U-box domain (U-box) // Kinesin-associated protein (KAP) (KAP) (1 of 1)	0.27	5.90E-01	0.79	7.04E-03	0.16	8.07E-01	0.78	1.12E-02
Phvul.002G119900	PF00069//PF00139 - Protein kinase domain (Pkinase) // Legume lectin domain (Lectin legB) (1 of 32)	0.27	7.09E-01	0.75	6.31E-02	0.28	7.25E-01	0.89	2.91E-02
Phvul.008G146600	PF07816 - Protein of unknown function (DUF1645) (DUF1645) (1 of 14)	0.27	3.57E-01	0.60	1.91E-03	0.01	9.93E-01	0.42	4.40E-02
Phvul.008G173200	PTHR23160:SF3 - SYNAPTONEMAL COMPLEX PROTEIN 1-RELATED (1 of 1)	0.27	5.18E-01	0.99	9.68E-05	0.57	8.50E-02	1.22	9.42E-07
Phvul.007G085600	PTHR31989:SF27 - F23M19.14 PROTEIN-RELATED (1 of 1)	0.27	3.15E-01	0.55	2.34E-03	0.25	4.04E-01	0.55	3.09E-03
Phvul.009G087500	PTHR24347:SF199 - CBL-INTERACTING SERINE/THREONINE-PROTEIN KINASE 25-RELATED (1 of 3)	0.27	2.85E-01	0.23	2.25E-01	0.46	2.99E-02	0.35	5.74E-02
Phvul.006G048400	no data	0.26	5.38E-01	0.73	4.93E-03	-0.05	9.51E-01	0.75	5.71E-03
Phvul.005G007000	KOG3377 - Uncharacterized conserved protein (1 of 1)	0.26	6.65E-01	0.82	3.79E-02	0.17	8.30E-01	0.46	3.09E-01
Phvul.007G149800	PTHR12419//PTHR12419:SF19 - OTU DOMAIN CONTAINING PROTEIN // SUBFAMILY NOT NAMED (1 of 3)	0.26	4.35E-01	0.47	3.58E-02	0.15	7.30E-01	0.36	1.34E-01
Phvul.001G099600	no data	0.26	4.75E-01	0.49	4.40E-02	0.13	7.93E-01	0.41	1.15E-01
Phvul.003G215200	PTHR13105:SF7 - MYELOID LEUKEMIA FACTOR (1 of 1)	0.26	3.10E-01	0.34	6.67E-02	0.28	3.20E-01	0.42	2.49E-02
Phvul.007G239000	PTHR18937//PTHR18937:SF220 - STRUCTURAL MAINTENANCE OF CHROMOSOMES SMC FAMILY MEMBER // SUBFAMIL	0.26	6.29E-01	0.59	7.52E-02	0.24	7.00E-01	0.67	4.53E-02
Phvul.001G011100	PTHR11850:SF111 - BEL1-LIKE HOMEODOMAIN PROTEIN 1-RELATED (1 of 6)	0.26	5.40E-01	0.57	3.59E-02	0.25	6.06E-01	0.70	9.82E-03
Phvul.008G120600	PTHR23315:SF69 - U-BOX DOMAIN-CONTAINING PROTEIN 12-RELATED (1 of 2)	0.26	5.18E-01	0.57	2.41E-02	0.37	3.47E-01	0.63	1.65E-02
Phvul.009G255350	no data	0.26	6.32E-01	0.61	6.45E-02	0.22	7.33E-01	0.73	2.86E-02
Phvul.004G058900	no data	0.26	5.76E-01	0.64	2.27E-02	0.15	7.97E-01	0.54	7.26E-02
Phvul.003G267700	PTHR13710//PTHR13710:SF95 - DNA HELICASE RECQ FAMILY MEMBER // SUBFAMILY NOT NAMED (1 of 1)	0.26	4.79E-01	0.66	3.36E-03	0.18	6.98E-01	0.91	4.64E-05
Phvul.009G130600	PTHR10891//PTHR10891:SF530 - EF-HAND CALCIUM-BINDING DOMAIN CONTAINING PROTEIN // SUBFAMILY NOT NAM	0.26	8.64E-01	1.71	3.12E-02	0.39	7.95E-01	2.12	7.63E-03
Phvul.004G025700	PTHR24093:SF264 - ATPASE 4, PLASMA MEMBRANE-TYPE (1 of 3)	0.26	7.92E-01	1.14	2.36E-02	0.36	7.25E-01	1.01	5.80E-02
Phvul.002G160600	PTHR31087:SF14 - PROTEIN LURP-ONE-RELATED 17 (1 of 2)	0.26	8.02E-01	1.00	8.45E-02	0.67	4.34E-01	1.23	3.33E-02
Phvul.008G266000	no data	0.25	5.11E-01	0.62	9.42E-03	0.37	3.29E-01	0.70	4.76E-03
Phvul.003G120800	PTHR27001:SF177 - F11A17.22 PROTEIN-RELATED (1 of 1)	0.25	4.81E-01	0.44	6.88E-02	0.19	6.65E-01	0.59	1.37E-02
Phvul.001G179400	PTHR10641//PTHR10641:SF561 - MYB-LIKE DNA-BINDING PROTEIN MYB // SUBFAMILY NOT NAMED (1 of 1)	0.25	6.81E-01	0.92	7.14E-03	0.17	8.28E-01	0.58	1.21E-01
Phvul.006G086000	PF02201//PF03004//PF03126 - SWIB/MDM2 domain (SWIB) // Plant transposase (Ptta/En/Spm family) (Transposase 24	0.25	3.04E-01	0.39	2.48E-02	0.26	3.32E-01	0.31	8.90E-02
Phvul.009G199000	PTHR31561:SF3 - 3-KETOACYL-COA SYNTHASE 11 (1 of 2)	0.25	4.65E-01	0.52	2.02E-02	0.29	4.23E-01	0.43	7.08E-02
Phvul.006G034200	PTHR10015:SF157 - HEAT STRESS TRANSCRIPTION FACTOR A-5 (1 of 2)	0.25	4.01E-01	0.57	3.49E-03	0.15	6.98E-01	0.54	8.75E-03
Phvul.008G283200	PTHR10625:SF124 - HISTONE DEACETYLASE 9 (1 of 2)	0.25	3.64E-01	0.38	5.24E-02	0.33	2.27E-01	0.45	2.28E-02
Phvul.009G054101	PTHR12755 - CLEAVAGE/POLYADENYLATION FACTOR IA SUBUNIT CLP1P (1 of 4)	0.25	6.29E-01	0.78	7.86E-03	0.01	9.92E-01	0.73	1.71E-02
Phvul.006G200000	PTHR22601:SF11 - OLIGOPEPTIDE TRANSPORTER 4 (1 of 3)	0.25	3.24E-01	0.38	3.77E-02	0.21	4.83E-01	0.51	5.32E-03
Phvul.003G175700	PTHR14950:SF15 - DICER-LIKE PROTEIN 4 (1 of 1)	0.25	6.36E-01	0.75	1.66E-02	0.16	8.17E-01	0.73	2.65E-02
Phvul.002G021700	PTHR23155//PTHR23155:SF543 - LEUCINE-RICH REPEAT-CONTAINING PROTEIN // SUBFAMILY NOT NAMED (1 of 10)	0.25	6.57E-01	0.79	1.48E-02	0.24	7.03E-01	0.78	2.15E-02
Phvul.008G205900	K11886 - proteasome component ECM29 (ECM29) (1 of 1)	0.25	4.62E-01	0.46	3.71E-02	0.15	7.35E-01	0.31	1.93E-01
Phvul.004G156400	PTHR10774:SF1 - C2 DOMAIN-CONTAINING PROTEIN (1 of 2)	0.25	6.21E-01	0.58	5.32E-02	0.20	7.31E-01	0.65	3.38E-02
Phvul.007G183100	PTHR13268:SF2 - AUTOPHAGY-RELATED PROTEIN 18G (1 of 2)	0.25	4.08E-01	0.44	2.75E-02	0.16	6.64E-01	0.44	3.47E-02
Phvul.008G156500	KOG0320 - Predicted E3 ubiquitin ligase (1 of 7)	0.25	4.27E-01	0.48	1.91E-02	0.16	6.85E-01	0.37	9.04E-02
Phvul.006G076400	1.11.1.7 - Peroxidase / Lactoperoxidase (1 of 96)	0.24	4.27E-01	0.50	1.23E-02	0.38	1.87E-01	0.46	2.97E-02
Phvul.007G264500	PF00917 - MATH domain (MATH) (1 of 11)	0.24	6.88E-01	0.94	6.42E-03	0.33	5.94E-01	0.95	8.02E-03
Phvul.007G034600	PTHR11206:SF87 - MATE EFFLUX FAMILY PROTEIN-RELATED (1 of 5)	0.24	6.28E-01	0.63	4.32E-02	0.03	9.69E-01	0.39	2.63E-01
Phvul.011G159700	K04730 - interleukin-1 receptor-associated kinase 1 (IRAK1) (1 of 24)	0.24	3.77E-01	0.50	6.08E-03	0.11	7.81E-01	0.63	6.56E-04
Phvul.009G197200	K03426 - NAD+ diphosphatase [E3.6.1.22, NUDT12, nudC] (1 of 2)	0.24	4.01E-01	0.24	2.81E-01	0.38	1.60E-01	0.50	1.37E-02
Phvul.002G180300	1.1.3.15 - (S)-2-hydroxy-acid oxidase / Hydroxy-acid oxidase B (1 of 3)	0.24	5.89E-01	0.68	9.15E-03	0.29	5.30E-01	0.33	2.63E-01
Phvul.004G137600	PTHR33107:SF1 - DR4 PROTEIN-RELATED (1 of 9)	0.24	8.93E-01	-2.28	5.25E-03	-0.16	9.44E-01	-1.51	8.11E-02
Phvul.008G113600	K08081 - Tropinone reductase 1 (TR1) (1 of 15)	0.24	5.90E-01	0.85	1.21E-03	0.43	2.85E-01	0.87	1.29E-03
Phvul.008G207000	PF13962 - Domain of unknown function (PGG) (1 of 69)	0.24	5.93E-01	0.62	2.30E-02	0.22	6.65E-01	0.61	3.27E-02
Phvul.007G136200	K11838 - ubiquitin carboxyl-terminal hydrolase 7 (USP7, UBP15) (1 of 4)	0.24	6.51E-01	0.71	2.36E-02	-0.14	8.42E-01	0.51	1.35E-01
Phvul.011G035500	PTHR22950//PTHR22950:SF242 - AMINO ACID TRANSPORTER // SUBFAMILY NOT NAMED (1 of 2)	0.24	3.53E-01	0.28	1.37E-01	0.41	6.39E-02	0.37	4.73E-02
Phvul.003G036100	PTHR11654:SF178 - PROTEIN NRT1/ PTR FAMILY 3.1 (1 of 1)	0.23	4.82E-01	-0.51	2.63E-02	0.16	7.03E-01	-0.15	5.99E-01
Phvul.002G325900	PTHR31168:SF1 - DUF599 FAMILY PROTEIN (1 of 3)	0.23	5.35E-01	0.52	2.80E-02	0.15	7.64E-01	0.43	8.52E-02

Phvul.010G023900	K17686 - Cu+-exporting ATPase (copA, ATP7) (1 of 6)	0.23	6.54E-01	0.50	1.20E-01	0.29	5.85E-01	0.68	3.17E-02
Phvul.002G196800	PTHR13533:SF11 - O-ACETYLTRANSFERASE CAS1P-LIKE PROTEIN-RELATED (1 of 2)	0.23	7.41E-01	0.90	4.99E-02	-0.05	9.64E-01	0.84	7.28E-02
Phvul.010G101200	PTHR11017//PTHR11017:SF174 - LEUCINE-RICH REPEAT-CONTAINING PROTEIN // SUBFAMILY NOT NAMED (1 of 4)	0.23	7.79E-01	1.07	1.51E-02	0.11	9.26E-01	1.29	4.25E-03
Phvul.005G103700	PTHR31805:SF3 - PROLINE-RICH PROTEIN-RELATED (1 of 2)	0.23	4.04E-01	0.31	1.20E-01	0.21	4.97E-01	0.54	4.53E-03
Phvul.009G122400	PTHR28259:SF1 - UPF0695 MEMBRANE PROTEIN YOR390W-RELATED (1 of 2)	0.23	4.25E-01	0.37	5.97E-02	0.29	3.24E-01	0.40	4.87E-02
Phvul.008G023700	K00033 - 6-phosphogluconate dehydrogenase (PGD, gnd, gntZ) (1 of 3)	0.23	4.41E-01	0.49	1.17E-02	0.11	7.83E-01	0.32	1.38E-01
Phvul.001G017100	PF00412//PF02809//PF12315 - LIM domain (LIM) // Ubiquitin interaction motif (UIM) // Protein of unknown function (	0.23	5.04E-01	0.61	4.58E-03	0.30	3.90E-01	0.69	1.59E-03
Phvul.001G162400	K12192 - charged multivesicular body protein 2B (CHMP2B) (1 of 1)	0.23	3.83E-01	0.18	3.96E-01	0.26	3.38E-01	0.36	4.97E-02
Phvul.003G204400	PTHR11972:SF83 - FERRIC REDUCTION OXIDASE 8, MITOCHONDRIAL (1 of 1)	0.23	6.15E-01	0.51	6.30E-02	0.29	5.26E-01	0.64	2.22E-02
Phvul.001G161300	PTHR13856//PTHR13856:SF92 - VHS DOMAIN CONTAINING PROTEIN FAMILY // SUBFAMILY NOT NAMED (1 of 1)	0.23	3.92E-01	0.55	1.40E-03	0.28	2.92E-01	0.53	3.19E-03
Phvul.001G119400	PTHR31194:SF0 - ETHYLENE-RESPONSIVE TRANSCRIPTION FACTOR CRF3-RELATED (1 of 2)	0.22	4.69E-01	0.29	1.93E-01	0.28	3.75E-01	0.42	4.85E-02
Phvul.002G128900	no data	0.22	4.43E-01	0.28	1.79E-01	0.23	4.70E-01	0.44	3.03E-02
Phvul.002G166400	PTHR23155//PTHR23155:SF506 - LEUCINE-RICH REPEAT-CONTAINING PROTEIN // SUBFAMILY NOT NAMED (1 of 1)	0.22	5.45E-01	0.55	1.51E-02	0.29	4.27E-01	0.66	3.80E-03
Phvul.010G029900	K09561 - STIP1 homology and U-box containing protein 1 (STUB1, CHIP) (1 of 1)	0.22	3.73E-01	0.44	8.58E-03	0.26	3.16E-01	0.46	8.95E-03
Phvul.002G183500	PTHR24067//PTHR24067:SF137 - UBIQUITIN-CONJUGATING ENZYME E2 // SUBFAMILY NOT NAMED (1 of 1)	0.22	6.11E-01	0.56	3.40E-02	-0.01	9.92E-01	0.52	6.08E-02
Phvul.009G053200	PTHR12356:SF3 - NUCLEAR MIGRATION PROTEIN NUDC (1 of 2)	0.22	2.31E-01	0.28	4.32E-02	0.29	1.19E-01	0.15	3.19E-01
Phvul.005G043600	PTHR11489:SF9 - 40S RIBOSOMAL PROTEIN SA (1 of 1)	0.22	1.84E-01	0.10	4.97E-01	0.34	2.25E-02	0.10	5.14E-01
Phvul.002G274400	PTHR21493//PTHR21493:SF130 - CGI-141-RELATED/LIPASE CONTAINING PROTEIN // SUBFAMILY NOT NAMED (1 of 2)	0.22	5.45E-01	0.71	1.32E-03	0.06	9.26E-01	0.61	9.51E-03
Phvul.002G319100	PF04749 - PLAC8 family (PLAC8) (1 of 18)	0.22	4.77E-01	0.27	2.25E-01	0.36	2.26E-01	0.58	4.47E-03
Phvul.005G135911	PF01344 - Kelch motif (Kelch 1) (1 of 34)	0.22	5.19E-01	0.55	9.92E-03	0.21	5.76E-01	0.60	6.05E-03
Phvul.002G242000	PTHR19139:SF144 - AQUAPORIN NIP1-1-RELATED (1 of 5)	0.22	5.65E-01	0.48	4.50E-02	0.26	5.21E-01	0.54	2.50E-02
Phvul.006G101200	PTHR11934:SF0 - RIBOSE-5-PHOSPHATE ISOMERASE (1 of 1)	0.22	7.20E-01	1.00	3.12E-03	0.01	9.91E-01	0.50	2.10E-01
Phvul.003G055800	PTHR23201//PTHR23201:SF18 - EXTENSIN, PROLINE-RICH PROTEIN // SUBFAMILY NOT NAMED (1 of 4)	0.22	6.58E-01	0.74	7.48E-03	0.31	5.25E-01	0.92	9.02E-04
Phvul.006G131900	no data	0.22	3.88E-01	0.42	1.50E-02	0.22	4.33E-01	0.35	5.81E-02
Phvul.009G229000	K12191 - charged multivesicular body protein 2A (CHMP2A) (1 of 4)	0.22	3.37E-01	0.32	4.40E-02	0.27	2.47E-01	0.40	1.33E-02
Phvul.007G042900	PTHR14155//PTHR14155:SF182 - RING FINGER DOMAIN-CONTAINING // SUBFAMILY NOT NAMED (1 of 1)	0.22	5.60E-01	0.29	2.70E-01	0.42	2.07E-01	0.62	9.43E-03
Phvul.008G248800	K12761 - carbon catabolite-derepressing protein kinase (SNF1) (1 of 2)	0.22	5.62E-01	0.46	5.18E-02	0.43	1.96E-01	0.59	1.27E-02
Phvul.002G234800	PTHR24031//PTHR24031:SF264 - RNA HELICASE // SUBFAMILY NOT NAMED (1 of 2)	0.22	6.11E-01	0.44	1.22E-01	0.26	5.62E-01	0.61	2.72E-02
Phvul.005G040900	PTHR14499//PTHR14499:SF70 - POTASSIUM CHANNEL TETRAMERIZATION DOMAIN-CONTAINING // SUBFAMILY NOT N	0.22	7.59E-01	1.02	7.07E-03	0.08	9.37E-01	0.97	1.52E-02
Phvul.006G186300	PF05755 - Rubber elongation factor protein (REF) (REF) (1 of 4)	0.22	4.73E-01	0.44	2.45E-02	0.43	1.04E-01	0.52	9.44E-03
Phvul.006G019600	PF16709 - Fused Ig-PH domain of plant-specific actin-binding protein (SCAB-IgPH) (1 of 3)	0.22	7.18E-01	0.68	3.96E-02	0.19	7.94E-01	0.74	2.72E-02
Phvul.007G078800	PTHR23257:SF505 - SERINE/THREONINE-PROTEIN KINASE DRKC-RELATED (1 of 1)	0.22	2.75E-01	0.39	4.93E-03	0.12	6.63E-01	0.30	4.71E-02
Phvul.011G201800	no data	0.22	5.84E-01	0.50	3.70E-02	0.17	7.31E-01	0.26	3.53E-01
Phvul.008G180400	no data	0.22	4.62E-01	0.27	2.08E-01	0.26	4.03E-01	0.43	3.44E-02
Phvul.002G209700	PTHR13832:SF314 - PROTEIN PHOSPHATASE 2C 10-RELATED (1 of 3)	0.22	5.25E-01	0.51	1.58E-02	0.20	6.11E-01	0.56	1.02E-02
Phvul.010G108900	K00215 - 4-hydroxy-tetrahydrodipicolinate reductase (dapB) (1 of 2)	0.22	5.65E-01	-0.35	1.76E-01	0.08	8.80E-01	-0.58	1.79E-02
Phvul.010G048200	PTHR10746:SF10 - 50S RIBOSOMAL PROTEIN L4, CHLOROPLASTIC (1 of 1)	0.22	2.48E-01	0.32	1.82E-02	0.21	3.08E-01	0.24	1.03E-01
Phvul.008G138300	PTHR10641:SF469 - TRANSCRIPTION FACTOR MYB21-RELATED (1 of 3)	0.22	4.30E-01	0.38	3.96E-02	0.30	2.66E-01	0.37	5.25E-02
Phvul.007G040000	KOG4055 - Uncharacterized conserved protein (1 of 1)	0.21	3.84E-01	0.16	4.22E-01	0.42	4.05E-02	0.20	2.75E-01
Phvul.007G108300	PTHR24188:SF44 - E3 UBIQUITIN-PROTEIN LIGASE XBAT35-RELATED (1 of 1)	0.21	3.34E-01	0.19	2.54E-01	0.26	2.51E-01	0.34	3.05E-02
Phvul.007G020700	PTHR11926//PTHR11926:SF384 - GLUCOSYL/GLUCURONOSYL TRANSFERASES // SUBFAMILY NOT NAMED (1 of 11)	0.21	6.00E-01	0.28	3.33E-01	0.47	1.64E-01	0.52	4.53E-02
Phvul.003G022000	PTHR22951//PTHR22951:SF25 - CLATHRIN ASSEMBLY PROTEIN // SUBFAMILY NOT NAMED (1 of 2)	0.21	8.54E-01	1.06	4.32E-02	0.25	8.35E-01	0.95	8.62E-02
Phvul.006G035500	2.3.3.1 - Citrate (Si)-synthase / Citrate oxaloacetate-lyase ((pro-3S)-CH(2)COO->acetyl-CoA) (1 of 2)	0.21	4.08E-01	0.42	1.51E-02	0.10	7.73E-01	0.42	2.02E-02
Phvul.010G123500	2.7.1.150 - 1-phosphatidylinositol-3-phosphate 5-kinase / Type III PIP kinase (1 of 4)	0.21	5.99E-01	0.55	2.30E-02	0.10	8.56E-01	0.60	1.87E-02
Phvul.003G247651	PTHR23155//PTHR23155:SF543 - LEUCINE-RICH REPEAT-CONTAINING PROTEIN // SUBFAMILY NOT NAMED (1 of 10)	0.21	6.91E-01	0.78	1.01E-02	0.36	4.79E-01	0.98	1.48E-03
Phvul.010G102800	PTHR24055:SF227 - MITOGEN-ACTIVATED PROTEIN KINASE 11-RELATED (1 of 1)	0.21	5.93E-01	0.58	1.48E-02	0.21	6.27E-01	0.61	1.40E-02
Phvul.003G024200	PTHR24092:SF41 - PHOSPHOLIPID-TRANSPORTING ATPASE 10-RELATED (1 of 4)	0.21	8.17E-01	1.20	8.40E-03	0.23	8.25E-01	1.12	2.01E-02
Phvul.003G147200	PTHR11615//PTHR11615:SF161 - NITRATE, FROMATE, IRON DEHYDROGENASE // SUBFAMILY NOT NAMED (1 of 1)	0.21	5.18E-01	0.42	4.47E-02	0.35	2.57E-01	0.35	1.14E-01
Phvul.009G127000	PTHR26402//PTHR26402:SF498 - RESPONSE REGULATOR OF TWO-COMPONENT SYSTEM // SUBFAMILY NOT NAMED (1	0.21	8.88E-01	-1.59	2.60E-02	-0.08	9.68E-01	-1.31	8.57E-02
Phvul.003G292700	PTHR10891//PTHR10891:SF533 - EF-HAND CALCIUM-BINDING DOMAIN CONTAINING PROTEIN // SUBFAMILY NOT NAM	0.21	6.55E-01	0.38	1.97E-01	0.68	3.95E-02	0.52	6.46E-02
Phvul.011G164900	PTHR13832:SF333 - PROTEIN PHOSPHATASE 2C 39-RELATED (1 of 2)	0.21	4.69E-01	0.41	2.82E-02	0.28	3.42E-01	0.35	7.67E-02
Phvul.001G183500	K12185 - ESCRT-I complex subunit VPS37 (VPS37) (1 of 2)	0.21	4.89E-01	0.47	1.35E-02	0.21	5.14E-01	0.36	8.00E-02
Phvul.011G202966	PTHR23155//PTHR23155:SF414 - LEUCINE-RICH REPEAT-CONTAINING PROTEIN // SUBFAMILY NOT NAMED (1 of 124)	0.21	5.19E-01	0.49	1.49E-02	0.08	8.70E-01	0.42	4.95E-02
Phvul.004G095000	no data	0.21	6.00E-01	0.54	2.17E-02	0.24	5.59E-01	0.50	4.54E-02
Phvul.004G044100	PTHR13683:SF348 - ASPARTYL PROTEASE FAMILY PROTEIN (1 of 5)	0.21	8.32E-01	-1.26	1.04E-02	0.04	9.82E-01	-1.35	7.89E-03
Phvul.007G258800	PF12796//PF13962 - Ankyrin repeats (3 copies) (Ank 2) // Domain of unknown function (PGG) (1 of 27)	0.21	6.29E-01	0.54	3.67E-02	0.21	6.57E-01	0.44	1.12E-01
Phvul.001G070000	K04733 - interleukin-1 receptor-associated kinase 4 (IRAK4) (1 of 48)	0.21	6.60E-01	0.81	1.93E-03	0.24	6.37E-01	0.74	7.41E-03
Phvul.010G022700	K17961 - cytochrome P450, family 82, subfamily G, polypeptide 1 (CYP82G1) (1 of 2)	0.21	8.14E-01	0.88	3.84E-02	-0.04	9.78E-01	0.48	3.25E-01
Phvul.001G033101	PTHR12857:SF0 - UPF0587 PROTEIN C1ORF123 (1 of 1)	0.21	4.72E-01	0.30	1.32E-01	0.32	2.34E-01	0.39	4.45E-02
Phvul.003G174300	PTHR14155//PTHR14155:SF158 - RING FINGER DOMAIN-CONTAINING // SUBFAMILY NOT NAMED (1 of 1)	0.20	5.70E-01	0.44	4.87E-02	0.19	6.44E-01	0.43	6.17E-02
Phvul.005G051900	PTHR10509:SF27 - PROTEIN COMT-1 (1 of 1)	0.20	7.08E-01	-0.68	4.21E-02	0.29	5.86E-01	-0.66	5.52E-02
Phvul.002G318200	PTHR31042:SF16 - CORE-2/I-BRANCHING BETA-1,6-N-ACETYLGLUCOSAMINYLTRANSFERASE FAMILY PROTEIN (1 of 2)	0.20	8.07E-01	1.79	4.04E-05	0.30	7.20E-01	1.61	4.37E-04
Phvul.002G254700	KOG0548//KOG0624 - Molecular co-chaperone STI1 // dsRNA-activated protein kinase inhibitor P58, contains TPR and D	0.20	8.89E-01	1.20	4.97E-02	0.66	5.57E-01	1.17	6.31E-02
Phvul.011G022700	2.7.1.23//2.7.1.86 - NAD(+) kinase / DPN kinase (1 of 2)	0.20	3.90E-01	0.30	6.53E-02	0.16	5.66E-01	0.33	4.40E-02
Phvul.006G197200	PTHR10334//PTHR10334:SF231 - CYSTEINE-RICH SECRETORY PROTEIN-RELATED Pathogenesis-related	0.20	9.41E-01	2.34	4.93E-02	0.20	9.54E-01	2.62	3.09E-02
Phvul.008G098000	K08518 - syntaxin-binding protein 5 (STXBP5) (1 of 2)	0.20	5.76E-01	0.51	1.74E-02	-0.02	9.81E-01	0.45	4.78E-02
Phvul.004G008861	PTHR16212:SF4 - FOCADHESIN (1 of 11)	0.20	8.72E-01	-0.62	3.34E-01	-0.16	9.23E-01	-1.47	1.67E-02
Phvul.009G237900	PTHR12537//PTHR12537:SF76 - RNA BINDING PROTEIN PUMILIO-RELATED // SUBFAMILY NOT NAMED (1 of 2)	0.20	5.09E-01	0.42	2.56E-02	0.10	7.94E-01	0.28	1.74E-01
Phvul.009G030700	PTHR23500:SF44 - SUGAR TRANSPORT PROTEIN 5 (1 of 5)	0.20	6.87E-01	1.00	1.41E-04	0.16	7.91E-01	0.59	4.31E-02
Phvul.003G219666	no data	0.20	6.14E-01	0.53	2.00E-02	0.11	8.34E-01	0.42	8.86E-02
Phvul.001G117900	PTHR10926//PTHR10926:SF28 - CELL CYCLE CONTROL PROTEIN 50 // SUBFAMILY NOT NAMED (1 of 2)	0.20	7.81E-01	1.21	1.00E-03	0.20	8.07E-01	1.02	8.66E-03
Phvul.001G064500	PTHR10791:SF44 - BIDIRECTIONAL SUGAR TRANSPORTER SWEET1 (1 of 2)	0.20	5.03E-01	0.52	3.77E-03	0.23	4.30E-01	0.63	5.03E-04
Phvul.L005343	PTHR21726//PTHR21726:SF39 - PHOSPHATIDYLINOSITOL N-ACETYLGLUCOSAMINYLTRANSFERASE SUBUNIT P DOWN SY	0.20	7.80E-01	0.95	9.67E-03	0.37	5.63E-01	0.87	2.40E-02
Phvul.003G113300	no data	0.19	4.87E-01	0.01	9.84E-01	0.47	3.23E-02	0.34	7.41E-02
Phvul.002G143400	PTHR27003:SF71 - LEUCINE-RICH REPEAT (LRR) FAMILY PROTEIN (1 of 2)	0.19	9.20E-01	1.86	5.44E-03	0.28	8.92E-01	1.28	8.05E-02
Phvul.008G263900	PTHR27003:SF63 - CALMODULIN-BINDING RECEPTOR-LIKE CYTOPLASMIC KINASE 1 (1 of 4)	0.19	6.35E-01	0.55	2.29E-02	0.26	5.18E-01	0.54	3.30E-02
Phvul.003G227400	K01338 - ATP-dependent Lon protease (lon) (1 of 2)	0.19	7.96E-01	1.27	8.73E-04	0.18	8.29E-01	1.19	2.72E-03
Phvul.003G129200	PTHR33077:SF13 - PROTEIN TIFY 10A-RELATED (1 of 4)	0.19	8.87E-01	0.29	7.73E-01	0.39	7.54E-01	1.72	9.46E-03
Phvul.003G233000	no data	0.19	6.08E-01	0.45	4.61E-02	0.32	3.46E-01	0.54	1.84E-02
Phvul.003G241600	K10577 - ubiquitin-conjugating enzyme E2 I (UBE2I, UBC9) (1 of 2)	0.19	4.21E-01	0.13	4.95E-01	0.39	4.73E-02	0.18	3.06E-01
Phvul.011G092700	K12196 - vacuolar protein-sorting-associated protein 4 (VPS4) (1 of 2)	0.19	5.44E-01	0.35	7.78E-02	0.26	4.03E-01	0.49	1.47E-02
Phvul.007G137700	K12198 - charged multivesicular body protein 5 (CHMP5, VPS60) (1 of 1)	0.19	3.19E-01	0.39	2.78E-03	0.15	5.05E-01	0.38	5.17E-03
Phvul.003G160700	PTHR23012//PTHR23012:SF54 - MEMBRANE ASSOCIATED RING FINGER // SUBFAMILY NOT NAMED (1 of 1)	0.19	5.14E-01	0.41	2.43E-02	0.23	4.18E-01	0.39	3.87E-02
Phvul.003G151800	PTHR32191:SF2 - TETRASPANIN-11-RELATED (1 of 2)	0.19	7.60E-01	0.76	2.03E-02	0.21	7.53E-01	0.83	1.37E-02
Phvul.009G090200	no data	0.19	5.64E-01	0.50	1.00E-02	0.21	5.44E-01	0.33	1.26E-01
Phvul.007G016200	KOG2985 - Uncharacterized conserved protein (1 of 2)	0.18	5.26E-01	0.31	1.12E-01	0.34	2.02E-01	0.47	1.23E-02
Phvul.003G076800	PTHR22572:SF119 - GLUCOSE-1-PHOSPHATE ADENYLYLTRANSFERASE LARGE SUBUNIT 1, CHLOROPLASTIC (1 of 1)	0.18	8.92E-01	-1.56	1.35E-02	-0.11	9.51E-01	-1.08	1.09E-01
Phvul.003G191900	PTHR24067//PTHR24067:SF128 - UBIQUITIN-CONJUGATING ENZYME E2 // SUBFAMILY NOT NAMED (1 of 1)	0.18	5.53E-01	0.42	2.93E-02	0.07	8.81E-01	0.25	2.35E-01
Phvul.009G225300	PTHR33077:SF13 - PROTEIN TIFY 10A-RELATED (1 of 4)	0.18	6.26E-01	0.62	5.05E-03	0.24	5.20E-01	0.78	4.37E-04
Phvul.L002444	no data	0.18	7.14E-01	0.67	1.51E-02	0.33	4.74E-01	0.70	1.54E-02
Phvul.009G164400	PTHR13734:SF47 - POLYNUCLEOTIDE ADENYLYLTRANSFERASE DOMAIN AND RNA RECOGNITION MOTIF-CONTAINING PF	0.18	6.02E-01	-0.21	3.94E-01	-0.10	8.32E-01	-0.49	2.55E-02
Phvul.002G159500	PTHR24055:SF224 - MITOGEN-ACTIVATED PROTEIN KINASE 5 (1 of 2)	0.18	5.56E-01	0.39	4.20E-02	0.17	6.10E-01	0.31	1.24E-01
Phvul.007G142700	no data	0.18	8.69E-01	-0.62	2.83E-01	-0.07	9.65E-01	-1.31	1.92E-02
Phvul.008G150100	no data	0.18	5.56E-01	0.41	3.28E-02	0.22	4.74E-01	0.38	5.72E-02
Phvul.009G243500	PTHR13271:SF23 - N-LYSINE METHYLTRANSFERASE EFM1 (1 of 1)	0.18	6.21E-01	-0.43	5.49E-02	0.00	9.97E-01	-0.50	2.88E-02
Phvul.011G049100	PF05055 - Protein of unknown function (DUF677) (DUF677) (1 of 9)	0.18	5.76E-01	0.41	3.77E-02	0.14	7.27E-01	0.40	5.08E-02
Phvul.007G085000	PF07343 - Protein of unknown function (DUF1475) (DUF1475) (1 of 2)	0.18	6.42E-01	0.49	3.33E-02	0.10	8.38E-01	0.31	2.23E-01
Phvul.010G017100	PTHR11732:SF223 - ALDO-KETO REDUCTASE FAMILY 4 MEMBER C10 (1 of 9)	0.18	6.96E-01	0.62	1.61E-02	0.30	4.78E-01	0.45	1.13E-01
Phvul.003G026601	PTHR31846:SF8 - CRS2-ASSOCIATED FACTOR 1, CHLOROPLASTIC (1 of 3)	0.18	6.18E-01	0.46	2.91E-02	0.29	3.78E-01	0.34	1.39E-01
Phvul.003G067900	PTHR19432:SF27 - SUCROSE TRANSPORT PROTEIN SUC3 (1 of 1)	0.18	6.91E-01	0.75	2.81E-03	0.42	2.63E-01	0.89	4.81E-04
Phvul.001G134700	PTHR34267:SF1 - AT0ZI1 PROTEIN (1 of 1)	0.18	5.34E-01	-0.03	8.98E-01	-0.05	8.99E-01	-0.37	4.67E-02
Phvul.006G113300	3.1.27.1 - Ribonuclease T(2) / Ribonuclease T2 (1 of 13)	0.18	7.98E-01	0.75	3.27E-02	-0.12	8.86E-01	0.53	1.64E-01
Phvul.006G058800	PF05701 - Weak chloroplast movement under blue light (WEMBL) (1 of 16)	0.18	7.39E-01	0.59	4.41E-02	0.09	9.01E-01	0.67	2.52E-02

Phvul.009G054000	PF13639//PF14380 - Ring finger domain (zf-RING 2) // Wall-associated receptor kinase C-terminal (WAK assoc) (1 of 2)	0.17	8.56E-01	1.07	1.80E-02	0.09	9.43E-01	1.23	8.76E-03
Phvul.003G127100	PTHR15629:SF2 - SH3 DOMAIN-CONTAINING YSC84-LIKE PROTEIN 1 (1 of 1)	0.17	5.70E-01	0.32	1.13E-01	0.27	3.52E-01	0.45	2.21E-02
Phvul.010G035800	2.7.11.1 - Non-specific serine/threonine protein kinase / Threonine-specific protein kinase (1 of 1198)	0.17	5.89E-01	0.45	2.25E-02	0.15	6.95E-01	0.43	3.31E-02
Phvul.001G247700	PF07107 - Wound-induced protein WI12 (WI12) (1 of 4)	0.17	6.08E-01	0.62	1.51E-03	0.44	1.00E-01	0.57	5.66E-03
Phvul.003G002200	PTHR31072:SF7 - TRANSCRIPTION FACTOR TCP21-RELATED (1 of 3)	0.17	5.65E-01	0.46	1.15E-02	0.06	8.99E-01	0.37	6.19E-02
Phvul.003G112900	no data	0.17	7.05E-01	0.07	8.47E-01	0.41	2.86E-01	0.56	3.61E-02
Phvul.003G239300	PF07714//PF12819 - Protein tyrosine kinase (Pkinase Tyr) // Carbohydrate-binding protein of the ER (Malectin like) (1 c	0.17	7.62E-01	0.77	8.12E-03	0.15	8.18E-01	0.94	1.57E-03
Phvul.004G156900	no data	0.17	6.59E-01	0.46	4.48E-02	0.25	5.21E-01	0.48	4.22E-02
Phvul.005G165100	K00850 - 6-phosphofructokinase 1 (pfkA, PFK) (1 of 9)	0.17	6.65E-01	0.57	1.05E-02	0.14	7.70E-01	0.38	1.23E-01
Phvul.005G161200	PF02701 - Dof domain, zinc finger (zf-Dof) (1 of 41)	0.17	7.62E-01	0.65	3.36E-02	0.00	1.00E+00	0.52	1.10E-01
Phvul.007G090500	KOG2462 - C2H2-type Zn-finger protein (1 of 4)	0.17	5.71E-01	0.37	4.85E-02	0.04	9.37E-01	0.35	6.43E-02
Phvul.008G186500	PF11331 - Probable zinc-ribbon domain (zinc ribbon 12) (1 of 4)	0.17	5.56E-01	0.54	1.64E-03	-0.03	9.52E-01	0.50	5.75E-03
Phvul.009G251700	PTHR18896:SF64 - PHOSPHOLIPASE D P2 (1 of 2)	0.17	5.98E-01	0.41	3.33E-02	0.05	9.28E-01	0.42	3.50E-02
Phvul.008G108900	PF00560 - Leucine Rich Repeat (LRR 1) (1 of 227)	0.17	8.78E-01	1.25	9.84E-03	0.00	9.99E-01	0.73	1.83E-01
Phvul.001G068500	K15171 - transcription elongation factor SPT4 (SUPT4H1, SPT4) (1 of 2)	0.16	7.06E-01	0.66	3.23E-03	0.15	7.71E-01	0.65	4.84E-03
Phvul.002G221500	PTHR22912:SF180 - 3-PHENYLPROPIONATE/CINNAMIC ACID DIOXYGENASE FERREDOXIN--NAD(+) REDUCTASE COMPON	0.16	6.16E-01	0.62	1.24E-03	0.18	6.13E-01	0.54	7.70E-03
Phvul.003G254600	no data	0.16	7.50E-01	0.59	3.92E-02	0.03	9.76E-01	0.67	2.21E-02
Phvul.010G157400	K00864 - glycerol kinase (glpK, GK) (1 of 1)	0.16	4.58E-01	0.31	3.21E-02	0.10	7.18E-01	0.29	5.52E-02
Phvul.005G008900	PTHR27003:SF29 - U-BOX DOMAIN-CONTAINING PROTEIN 35-RELATED (1 of 1)	0.16	5.03E-01	0.36	1.95E-02	0.08	7.96E-01	0.28	9.52E-02
Phvul.009G056300	PTHR13286 - SAP30 (1 of 2)	0.16	6.89E-01	-0.56	1.59E-02	0.16	7.34E-01	-0.47	5.66E-02
Phvul.007G067300	PTHR22936:SF35 - MEMBRANE PROTEIN-RELATED (1 of 2)	0.16	6.55E-01	0.37	9.48E-02	0.12	7.77E-01	0.59	6.16E-03
Phvul.007G067800	PTHR10015:SF168 - HEAT STRESS TRANSCRIPTION FACTOR B-2A (1 of 2)	0.16	8.17E-01	0.65	7.54E-02	0.23	7.41E-01	0.93	1.04E-02
Phvul.008G285000	KOG0204 - Calcium transporting ATPase (1 of 1)	0.16	7.80E-01	0.54	9.33E-02	0.20	7.50E-01	0.78	1.28E-02
Phvul.011G014301	PTHR23155:SF402 - DISEASE RESISTANCE PROTEIN RPP13-RELATED (1 of 8)	0.16	5.14E-01	0.36	2.02E-02	0.16	5.40E-01	0.34	3.65E-02
Phvul.003G155500	K13431 - signal recognition particle receptor subunit alpha (SRPR) (1 of 2)	0.16	7.53E-01	0.58	3.89E-02	0.14	8.08E-01	0.51	8.65E-02
Phvul.009G079000	PTHR33193:SF8 - GENOMIC DNA, CHROMOSOME 3, P1 CLONE: MDC16 (1 of 2)	0.16	7.40E-01	0.58	2.90E-02	-0.03	9.64E-01	0.47	9.66E-02
Phvul.006G048000	PTHR13683:SF246 - ASPARTYL PROTEASE FAMILY PROTEIN (1 of 2)	0.16	7.52E-01	0.56	4.61E-02	0.20	7.12E-01	0.49	9.63E-02
Phvul.003G155800	PTHR10217:SF500 - CYCLIC NUCLEOTIDE-GATED ION CHANNEL 5-RELATED (1 of 1)	0.16	6.54E-01	0.36	9.59E-02	0.12	7.84E-01	0.45	3.59E-02
Phvul.008G028900	K13379 - reversibly glycosylated polypeptide / UDP-arabinopyranose mutase (RGP, UTM) (1 of 5)	0.16	9.24E-01	1.31	7.41E-02	0.08	9.69E-01	1.69	2.18E-02
Phvul.009G194700	K13431 - signal recognition particle receptor subunit alpha (SRPR) (1 of 2)	0.16	5.55E-01	0.34	4.18E-02	0.13	6.84E-01	0.34	5.27E-02
Phvul.009G148600	PTHR10615:SF112 - HISTONE-LYSINE N-METHYLTRANSFERASE ATXR5 (1 of 2)	0.16	7.99E-01	-0.82	2.78E-02	0.12	8.67E-01	-0.30	4.57E-01
Phvul.011G215600	KOG0255 - Synaptic vesicle transporter SVOP and related transporters (major facilitator superfamily) (1 of 6)	0.16	9.25E-01	1.33	3.92E-02	0.55	6.82E-01	1.49	2.15E-02
Phvul.009G208801	PTHR23500:SF12 - SUGAR TRANSPORT PROTEIN 7 (1 of 1)	0.15	7.62E-01	0.48	8.97E-02	0.20	7.00E-01	0.57	4.76E-02
Phvul.002G026600	KOG1330 - Sugar transporter/spinster transmembrane protein (1 of 6)	0.15	7.81E-01	0.65	2.12E-02	-0.02	9.89E-01	0.33	3.10E-01
Phvul.009G209500	PTHR11850//PTHR11850:SF127 - HOMEOBOX PROTEIN TRANSCRIPTION FACTORS // SUBFAMILY NOT NAMED (1 of 2)	0.15	5.08E-01	0.29	5.10E-02	0.23	2.90E-01	0.33	2.92E-02
Phvul.003G249100	PTHR16166//PTHR16166:SF98 - VACUOLAR PROTEIN SORTING-ASSOCIATED PROTEIN VPS13 // SUBFAMILY NOT NAMED	0.15	7.29E-01	0.51	4.32E-02	-0.10	8.59E-01	0.38	1.64E-01
Phvul.002G056700	PTHR10909:SF250 - PEROXISOMAL ACYL-COENZYME A OXIDASE 2 (1 of 2)	0.15	6.95E-01	0.48	3.46E-02	0.14	7.55E-01	0.33	1.78E-01
Phvul.002G151800	PF10693 - Protein of unknown function (DUF2499) (DUF2499) (1 of 1)	0.15	7.04E-01	-0.56	1.42E-02	0.09	8.64E-01	-0.52	3.23E-02
Phvul.011G183000	PF00407 - Pathogenesis-related protein Bet v I family (Bet v 1) (1 of 1)	0.15	9.29E-01	0.76	3.94E-01	-0.39	8.07E-01	1.82	2.40E-02
Phvul.008G292000	2.7.10.2//2.7.11.1 - Non-specific protein-tyrosine kinase / Cytoplasmic protein tyrosine kinase // Non-specific serine/thr	0.15	5.01E-01	0.43	1.89E-03	0.18	4.42E-01	0.39	7.47E-03
Phvul.009G028700	K11262 - acetyl-CoA carboxylase / biotin carboxylase (ACAC) (1 of 1)	0.15	6.33E-01	0.51	5.84E-03	0.09	8.32E-01	0.58	2.47E-03
Phvul.002G319700	PTHR32227:SF61 - GLUCAN ENDO-1,3-BETA-GLUCOSIDASE 3 (1 of 2)	0.15	5.21E-01	0.30	4.84E-02	0.23	3.10E-01	0.35	2.06E-02
Phvul.008G285600	PTHR21736:SF20 - PROTEIN OBERON 1-RELATED (1 of 2)	0.15	7.02E-01	0.42	6.21E-02	0.08	8.64E-01	0.51	2.41E-02
Phvul.001G049500	no data	0.15	7.15E-01	0.54	1.51E-02	0.20	6.30E-01	0.29	2.48E-01
Phvul.002G140100	PTHR31065:SF2 - PLATZ TRANSCRIPTION FACTOR DOMAIN-CONTAINING PROTEIN (1 of 2)	0.15	6.29E-01	0.39	3.05E-02	0.13	7.09E-01	0.45	1.56E-02
Phvul.009G129900	no data	0.15	8.47E-01	-0.50	2.25E-01	0.15	8.57E-01	-0.98	1.19E-02
Phvul.002G122200	PTHR24286:SF10 - ABSCISIC ACID 8'-HYDROXYLASE 1-RELATED (1 of 2)	0.15	8.58E-01	1.06	6.51E-03	0.33	6.45E-01	1.13	5.44E-03
Phvul.007G246100	no data	0.14	8.14E-01	0.01	9.90E-01	-0.04	9.64E-01	-0.67	4.40E-02
Phvul.004G173900	K00850 - 6-phosphofructokinase 1 (pfkA, PFK) (1 of 9)	0.14	6.34E-01	0.43	1.51E-02	0.23	4.28E-01	0.37	4.90E-02
Phvul.007G280200	PF00069//PF01476 - Protein kinase domain (Pkinase) // LysM domain (LysM) (1 of 4)	0.14	6.99E-01	0.78	4.04E-05	0.16	6.77E-01	0.72	2.46E-04
Phvul.002G330400	PTHR10108:SF793 - PHOSPHOETHANOLAMINE N-METHYLTRANSFERASE 1-RELATED (1 of 2)	0.14	9.10E-01	-1.13	4.87E-02	-0.10	9.51E-01	-0.94	1.23E-01
Phvul.004G049300	K01180 - endo-1,3(4)-beta-glucanase (E3.2.1.6) (1 of 6)	0.14	7.15E-01	0.40	7.75E-02	0.20	6.09E-01	0.47	3.81E-02
Phvul.006G180500	no data	0.14	5.41E-01	-0.28	5.35E-02	0.11	6.95E-01	-0.33	2.44E-02
Phvul.009G052800	3.4.14.5 - Dipeptidyl-peptidase IV / Xaa-Pro-dipeptidylaminopeptidase (1 of 1)	0.14	6.66E-01	0.38	4.43E-02	0.07	8.74E-01	0.35	7.04E-02
Phvul.011G157900	2.7.7.38 - 3-deoxy-manno-octulosonate cytidylyltransferase / CMP-KDO synthetase (1 of 1)	0.14	6.41E-01	-0.40	2.42E-02	0.04	9.36E-01	-0.31	1.03E-01
Phvul.010G086901	K05282 - gibberellin 20-oxidase (E1.14.11.12) (1 of 5)	0.14	8.62E-01	-0.90	1.48E-02	0.31	6.46E-01	-0.49	2.20E-01
Phvul.003G151600	PTHR11863:SF2 - PROTEIN ECERIFERUM 3 (1 of 1)	0.14	7.17E-01	0.49	2.14E-02	0.02	9.69E-01	0.42	6.32E-02
Phvul.005G085100	PTHR11668//PTHR11668:SF267 - SERINE/THREONINE PROTEIN PHOSPHATASE // SUBFAMILY NOT NAMED (1 of 2)	0.14	6.84E-01	0.38	5.26E-02	0.38	1.42E-01	0.42	3.14E-02
Phvul.002G328300	PF01535//PF13041//PF14432 - PPR repeat (PPR) // PPR repeat family (PPR 2) // DYW family of nucleic acid deaminases	0.14	6.69E-01	0.46	9.72E-03	0.08	8.35E-01	0.38	4.42E-02
Phvul.001G247000	PTHR18866//PTHR18866:SF99 - CARBOXYLASE:PYRUVATE/ACETYL-COA/PROPIONYL-COA CARBOXYLASE // SUBFAMILY N	0.14	6.35E-01	-0.34	5.41E-02	0.00	9.99E-01	-0.36	4.85E-02
Phvul.009G017500	PF15365 - Proline-rich nuclear receptor coactivator (PNRC) (1 of 5)	0.13	5.77E-01	0.21	2.14E-01	0.20	3.80E-01	0.33	3.36E-02
Phvul.007G041500	no data	0.13	8.17E-01	-0.44	1.80E-01	0.07	9.33E-01	-0.70	2.72E-02
Phvul.003G087100	no data	0.13	7.21E-01	0.43	4.16E-02	0.03	9.53E-01	0.36	1.12E-01
Phvul.007G012300	PTHR34669:SF1 - MESOPHYLL-CELL RNAI LIBRARY LINE 7-LIKE PROTEIN (1 of 1)	0.13	6.62E-01	-0.36	5.13E-02	-0.04	9.23E-01	-0.37	4.60E-02
Phvul.003G072200	PF00560//PF08263//PF13855 - Leucine Rich Repeat (LRR 1) // Leucine rich repeat N-terminal domain (LRRNT 2) // Leuc	0.13	6.92E-01	0.42	2.35E-02	0.02	9.64E-01	0.42	2.80E-02
Phvul.011G203200	K09567 - peptidyl-prolyl isomerase H (cyclophilin H) (PPIH, CYPH) (1 of 1)	0.13	6.10E-01	-0.15	4.21E-01	0.03	9.32E-01	-0.32	4.87E-02
Phvul.002G009400	no data	0.13	7.14E-01	0.15	5.17E-01	0.31	2.92E-01	0.46	2.24E-02
Phvul.008G249400	PTHR23315//PTHR23315:SF117 - BETA CATENIN-RELATED ARMADILLO REPEAT-CONTAINING // SUBFAMILY NOT NAMED	0.13	7.47E-01	0.63	2.78E-03	0.02	9.73E-01	0.58	9.59E-03
Phvul.003G136800	KOG4452 - Predicted membrane protein (1 of 1)	0.12	6.52E-01	-0.10	6.34E-01	0.01	9.86E-01	-0.41	1.60E-02
Phvul.002G196500	K12624 - U6 snRNA-associated Sm-like protein LSm5 (LSM5) (1 of 2)	0.12	7.22E-01	-0.34	9.92E-02	-0.09	8.32E-01	-0.41	4.87E-02
Phvul.011G195100	PTHR23155//PTHR23155:SF414 - LEUCINE-RICH REPEAT-CONTAINING PROTEIN // SUBFAMILY NOT NAMED (1 of 124)	0.12	9.27E-01	1.56	1.68E-02	0.75	3.86E-01	1.29	6.74E-02
Phvul.001G175600	K03000 - DNA-directed RNA polymerase I subunit RPA12 (RPA12, ZNRD1) (1 of 1)	0.12	7.35E-01	-0.17	4.99E-01	-0.10	8.24E-01	-0.50	1.79E-02
Phvul.003G099100	K15108 - solute carrier family 25 (mitochondrial thiamine pyrophosphate transporter), member 19 (SLC25A19, DNC, TPC	0.12	7.43E-01	-0.70	1.21E-03	-0.14	7.44E-01	-0.29	2.21E-01
Phvul.006G065400	no data	0.12	6.60E-01	0.37	2.18E-02	0.11	7.44E-01	0.31	6.55E-02
Phvul.006G082311	PTHR24282//PTHR24282:SF11 - CYTOCHROME P450 FAMILY MEMBER // SUBFAMILY NOT NAMED (1 of 3)	0.12	6.35E-01	-0.29	6.56E-02	0.07	8.33E-01	-0.44	4.14E-03
Phvul.006G125700	PTHR27005:SF7 - WALL-ASSOCIATED RECEPTOR KINASE-LIKE 1-RELATED (1 of 6)	0.12	7.40E-01	0.47	1.90E-02	0.33	2.60E-01	0.65	1.09E-03
Phvul.005G044300	PTHR31446:SF2 - ACID PHOSPHATASE/VANADIUM-DEPENDENT HALOPEROXIDASE-RELATED PROTEIN (1 of 1)	0.12	7.53E-01	-0.38	8.32E-02	-0.25	4.72E-01	-0.65	2.47E-03
Phvul.003G271800	PTHR10572:SF7 - 3-HYDROXY-3-METHYLGLUTARYL-COENZYME A REDUCTASE 2 (1 of 2)	0.12	9.41E-01	-1.79	1.15E-02	-0.58	6.54E-01	-1.19	1.15E-01
Phvul.008G286800	PTHR12899//PTHR12899:SF10 - 39S RIBOSOMAL PROTEIN L18, MITOCHONDRIAL // SUBFAMILY NOT NAMED (1 of 1)	0.12	8.56E-01	0.66	3.92E-02	0.13	8.48E-01	0.19	6.37E-01
Phvul.004G040901	PF11721 - Di-glucose binding within endoplasmic reticulum (Malectin) (1 of 50)	0.12	8.73E-01	0.38	3.41E-01	0.21	7.71E-01	0.74	4.29E-02
Phvul.002G059900	K15292 - syntaxin-binding protein 1 (STXBP1, MUNC18-1) (1 of 3)	0.12	7.83E-01	0.41	7.75E-02	0.13	7.77E-01	0.55	1.54E-02
Phvul.009G128066	K10755 - replication factor C subunit 2/4 (RFC2 4) (1 of 3)	0.12	7.18E-01	-0.09	7.25E-01	-0.03	9.46E-01	-0.39	3.92E-02
Phvul.011G162000	K08956 - AFG3 family protein [EC:3.4.24.-] (AFG3) (1 of 2)	0.12	6.95E-01	0.23	2.10E-01	0.02	9.69E-01	0.37	3.63E-02
Phvul.011G032000	K00208 - enoyl- (fabI) (1 of 2)	0.11	6.41E-01	0.37	8.72E-03	0.14	5.82E-01	0.24	1.30E-01
Phvul.001G058700	K10739 - replication factor A2 (RFA2, RPA2) (1 of 2)	0.11	8.40E-01	0.60	3.46E-02	0.08	9.14E-01	0.59	4.35E-02
Phvul.005G177900	PTHR27002:SF31 - G-TYPE LECTIN S-RECEPTOR-LIKE SERINE/THREONINE-PROTEIN KINASE SD2-5 (1 of 3)	0.11	7.73E-01	0.48	2.06E-02	0.12	7.74E-01	0.28	2.23E-01
Phvul.011G037300	PTHR23334 - CCAAT/ENHANCER BINDING PROTEIN (1 of 2)	0.11	9.10E-01	-1.39	1.16E-02	-0.19	8.46E-01	-0.40	4.98E-01
Phvul.011G109100	PTHR31717:SF7 - B-BOX DOMAIN PROTEIN 26-RELATED (1 of 4)	0.11	6.87E-01	0.44	5.02E-03	0.12	6.85E-01	0.49	2.25E-03
Phvul.003G281100	PTHR12560:SF13 - LAG1 LONGEVITY ASSURANCE HOMOLOG 1-RELATED (1 of 1)	0.11	6.95E-01	0.35	3.13E-02	0.05	9.03E-01	0.20	2.70E-01
Phvul.002G078300	PF13704 - Glycosyl transferase family 2 (Glyco tranf 2 4) (1 of 5)	0.11	8.00E-01	0.69	1.61E-03	0.28	4.60E-01	0.72	1.40E-03
Phvul.008G277800	PTHR23416:SF56 - SERINE ACETYLTRANSFERASE 1, CHLOROPLASTIC-RELATED (1 of 2)	0.11	9.06E-01	0.99	1.67E-02	0.23	7.94E-01	0.86	5.21E-02
Phvul.011G124500	PTHR14493:SF53 - ZINC FINGER CCCH DOMAIN-CONTAINING PROTEIN 2-RELATED (1 of 2)	0.11	7.96E-01	-0.07	8.13E-01	-0.01	9.95E-01	-0.49	4.16E-02
Phvul.002G305100	3.4.13.9 - Xaa-Pro dipeptidase / X-Pro dipeptidase (1 of 1)	0.11	7.77E-01	-0.17	4.91E-01	-0.01	9.90E-01	-0.50	2.04E-02
Phvul.005G160100	K11145 - ribonuclease III family protein [EC:3.1.26.-] (K11145) (1 of 1)	0.11	7.77E-01	-0.34	1.18E-01	0.03	9.64E-01	-0.48	2.46E-02
Phvul.002G012600	PTHR11207//PTHR11207:SF4 - RIBONUCLEASE III // SUBFAMILY NOT NAMED (1 of 2)	0.11	8.36E-01	-0.38	1.71E-01	0.13	8.16E-01	-0.57	3.61E-02
Phvul.011G089500	PTHR22950:SF319 - GABA TRANSPORTER 1 (1 of 3)	0.11	9.00E-01	0.96	1.24E-02	0.03	9.82E-01	0.91	2.33E-02
Phvul.008G158106	PTHR31236:SF2 - DEHYDRATION-RESPONSIVE PROTEIN RD22 (1 of 4)	0.11	9.33E-01	0.74	1.93E-01	-0.09	9.55E-01	1.20	2.39E-02
Phvul.007G042800	PTHR13832:SF362 - PROTEIN PHOSPHATASE 2C 63-RELATED (1 of 2)	0.11	8.22E-01	0.52	2.84E-02	0.20	6.59E-01	0.49	5.05E-02
Phvul.003G153800	PTHR13222 - CGTHBA PROTEIN -14 GENE PROTEIN (1 of 1)	0.10	8.14E-01	0.26	3.23E-01	0.12	8.03E-01	0.50	4.05E-02
Phvul.009G059200	PTHR13061//PTHR13061:SF9 - DYNACTIN SUBUNIT P25 // SUBFAMILY NOT NAMED (1 of 3)	0.10	7.36E-01	-0.13	5.29E-01	0.07	8.59E-01	-0.43	1.45E-02
Phvul.010G129100	PTHR21262//PTHR21262:SF14 - GUANOSINE-3',5'-BIS DIPHOSPHATE 3'-PYROPHOSPHOHYDROLASE // SUBFAMILY NOT	0.10	8.38E-01	0.29	3.06E-01	0.14	7.93E-01	0.55	3.63E-02
Phvul.011G119100	PTHR11746//PTHR11746:SF83 - O-METHYLTRANSFERASE // SUBFAMILY NOT NAMED (1 of 9)	0.10	7.72E-01	0.38	5.41E-02	0.07	8.80E-01	0.49	1.37E-02
Phvul.008G030100	2.7.11.1 - Non-specific serine/threonine protein kinase / Threonine-specific protein kinase (1 of 1198)	0.10	8.97E-01	1.10	2.74E-04	0.77	7.58E-02	0.95	3.09E-03

Phvul.003G138500	KOG2210 - Oxysterol-binding protein (1 of 4)	0.10	7.93E-01	-0.15	5.82E-01	0.04	9.47E-01	-0.55	1.28E-02
Phvul.005G004500	K12878 - THO complex subunit 1 (THOC1) (1 of 1)	0.10	7.24E-01	0.34	3.53E-02	0.08	8.26E-01	0.10	6.27E-01
Phvul.009G092900	PTHR27002:SF31 - G-TYPE LECTIN S-RECEPTOR-LIKE SERINE/THREONINE-PROTEIN KINASE SD2-5 (1 of 3)	0.10	8.01E-01	0.47	2.50E-02	0.27	4.32E-01	0.51	1.97E-02
Phvul.001G135300	PTHR24349:SF93 - CALCIUM-DEPENDENT PROTEIN KINASE 14 (1 of 1)	0.10	7.22E-01	0.36	2.72E-02	0.21	4.03E-01	0.46	4.68E-03
Phvul.009G244600	PF00069//PF00560//PF08263 - Protein kinase domain (Pkinase) // Leucine Rich Repeat (LRR 1) // Leucine rich repeat N	0.10	8.97E-01	-1.30	6.91E-04	-0.37	5.80E-01	-0.87	2.16E-02
Phvul.006G154400	PTHR10383//PTHR10383:SF24 - SERINE INCORPORATOR // SUBFAMILY NOT NAMED (1 of 1)	0.10	7.90E-01	0.42	3.92E-02	0.13	7.35E-01	0.46	2.50E-02
Phvul.008G167350	no data	0.10	8.25E-01	0.59	9.95E-03	0.15	7.33E-01	0.66	4.57E-03
Phvul.011G064200	PTHR33102:SF3 - DVL13-RELATED (1 of 2)	0.10	8.56E-01	0.65	1.20E-02	0.26	5.74E-01	0.60	2.78E-02
Phvul.001G152600	PF05910 - Plant protein of unknown function (DUF868) (DUF868) (1 of 16)	0.10	8.51E-01	0.53	3.73E-02	0.19	7.04E-01	0.43	1.13E-01
Phvul.002G306600	1.13.99.1 - Inositol oxygenase / Myo-inositol oxygenase (1 of 8)	0.10	7.57E-01	0.42	1.24E-02	0.01	9.82E-01	0.30	1.05E-01
Phvul.002G000600	PTHR31403:SF2 - PHOSPHOLIPASE A1-IBETA2, CHLOROPLASTIC (1 of 2)	0.10	8.89E-01	0.74	2.34E-02	0.44	3.86E-01	0.85	1.15E-02
Phvul.010G165100	K15813 - beta-amyrin synthase (LUP4) (1 of 8)	0.10	9.60E-01	-2.31	4.36E-03	-0.39	8.22E-01	-1.32	1.26E-01
Phvul.002G306800	PTHR31513:SF1 - GLYCINE-RICH PROTEIN (1 of 2)	0.10	8.59E-01	0.42	1.58E-01	0.23	6.23E-01	0.76	4.84E-03
Phvul.003G147800	PTHR31713:SF12 - CALMODULIN-BINDING PROTEIN (1 of 1)	0.09	8.07E-01	0.53	8.31E-03	0.17	6.55E-01	0.56	7.18E-03
Phvul.011G176800	PTHR23257:SF438 - F3O9.7 PROTEIN-RELATED (1 of 3)	0.09	7.88E-01	0.32	1.00E-01	0.03	9.46E-01	0.40	4.22E-02
Phvul.006G172500	PF13962 - Domain of unknown function (PGG) (1 of 69)	0.09	9.50E-01	-1.89	1.02E-02	-0.23	8.84E-01	-1.74	1.94E-02
Phvul.003G150900	K19044 - E3 ubiquitin-protein ligase XBAT32/33 [EC:6.3.2.19] (XBAT32 33) (1 of 3)	0.09	7.77E-01	0.36	4.76E-02	0.08	8.30E-01	0.36	5.50E-02
Phvul.003G274100	PTHR32054:SF2 - PROTEIN WEAK CHLOROPLAST MOVEMENT UNDER BLUE LIGHT 1-RELATED (1 of 3)	0.09	8.53E-01	0.17	5.91E-01	0.17	7.27E-01	0.53	4.15E-02
Phvul.011G115300	PTHR31407:SF14 - PSBP DOMAIN-CONTAINING PROTEIN 6, CHLOROPLASTIC (1 of 1)	0.09	8.91E-01	0.63	4.52E-02	0.20	7.54E-01	0.12	7.79E-01
Phvul.002G131600	PF11947 - Protein of unknown function (DUF3464) (DUF3464) (1 of 2)	0.09	8.65E-01	-0.52	5.46E-02	0.06	9.32E-01	-0.55	4.56E-02
Phvul.004G001700	3.5.1.94 - Gamma-glutamyl-gamma-aminobutyrate hydrolase / Gamma-glutamyl-GABA hydrolase (1 of 2)	0.09	7.92E-01	0.35	6.23E-02	0.14	6.77E-01	0.49	9.13E-03
Phvul.009G103700	PTHR10168//PTHR10168:SF87 - GLUTAREDOXIN // SUBFAMILY NOT NAMED (1 of 1)	0.09	8.70E-01	-0.58	3.15E-02	-0.14	8.02E-01	-0.47	1.03E-01
Phvul.011G101800	PF03595 - Voltage-dependent anion channel (SLAC1) (1 of 5)	0.09	7.57E-01	-0.37	1.91E-02	0.10	7.50E-01	-0.30	7.30E-02
Phvul.002G117400	PTHR13832:SF304 - PROTEIN PHOSPHATASE 2C 47-RELATED (1 of 1)	0.09	8.96E-01	-0.86	1.00E-02	-0.20	7.54E-01	-0.42	2.56E-01
Phvul.003G219500	PF04073 - Aminoacyl-tRNA editing domain (tRNA edit) (1 of 2)	0.09	8.48E-01	-0.48	4.72E-02	0.00	9.96E-01	-0.45	7.30E-02
Phvul.009G254700	PTHR11711//PTHR11711:SF174 - ADP RIBOSYLATION FACTOR-RELATED // SUBFAMILY NOT NAMED (1 of 2)	0.09	7.42E-01	0.34	2.20E-02	0.06	8.57E-01	0.22	1.83E-01
Phvul.005G032500	PTHR21495:SF71 - DIRIGENT PROTEIN 1-RELATED (1 of 10)	0.09	9.67E-01	-2.03	2.24E-02	-0.15	9.51E-01	-1.03	2.88E-01
Phvul.001G180700	PTHR36737:SF1 - EXPRESSED PROTEIN (1 of 1)	0.09	8.92E-01	-0.46	1.46E-01	0.05	9.54E-01	-0.75	1.49E-02
Phvul.003G067200	PTHR12300:SF51 - HVA22-LIKE PROTEIN C (1 of 2)	0.08	9.12E-01	0.62	7.67E-02	0.36	5.18E-01	0.70	4.66E-02
Phvul.008G035200	KOG3801 - Uncharacterized conserved protein BCN92 (1 of 1)	0.08	8.71E-01	-0.16	6.18E-01	0.16	7.40E-01	-0.59	2.36E-02
Phvul.008G104600	2.7.11.1 - Non-specific serine/threonine protein kinase / Threonine-specific protein kinase (1 of 1198)	0.08	8.84E-01	-0.47	8.71E-02	-0.01	9.93E-01	-0.72	7.53E-03
Phvul.007G267300	PTHR19957:SF69 - SYNTAXIN-121-RELATED (1 of 4)	0.08	8.80E-01	0.53	4.13E-02	0.22	6.27E-01	0.55	3.80E-02
Phvul.009G125600	PTHR35278:SF1 - F8K7.16 (1 of 2)	0.08	8.59E-01	-0.48	3.01E-02	-0.25	5.06E-01	-0.56	1.20E-02
Phvul.005G105200	PTHR31657:SF9 - ETHYLENE-RESPONSIVE TRANSCRIPTION FACTOR CRF5-RELATED (1 of 7)	0.08	9.37E-01	1.11	2.07E-02	0.45	5.29E-01	1.17	1.74E-02
Phvul.008G259800	PTHR13832:SF117 - PROTEIN PHOSPHATASE 2C-LIKE PROTEIN-RELATED (1 of 2)	0.08	8.95E-01	0.33	2.77E-01	0.43	3.07E-01	0.75	6.38E-03
Phvul.005G032600	PTHR21495:SF59 - DIRIGENT PROTEIN 20-RELATED (1 of 4)	0.08	9.62E-01	-1.85	8.91E-03	-0.11	9.57E-01	-0.90	2.49E-01
Phvul.004G079900	PTHR11709:SF94 - LACCASE-15 (1 of 3)	0.08	8.92E-01	-0.23	4.84E-01	0.22	6.50E-01	-0.61	3.62E-02
Phvul.004G172500	PTHR12161:SF13 - REGULATOR OF VPS4 ACTIVITY PROTEIN-RELATED (1 of 1)	0.08	8.22E-01	0.32	8.12E-02	-0.01	9.79E-01	0.44	1.52E-02
Phvul.003G054700	KOG4401 - Uncharacterized conserved protein (1 of 1)	0.08	9.32E-01	0.95	2.18E-02	-0.03	9.78E-01	1.05	1.38E-02
Phvul.002G016500	K18822 - soyasapogenol B glucuronide galactosyltransferase (UGT73P2) (1 of 1)	0.08	9.57E-01	-1.51	1.21E-02	-0.01	9.98E-01	-0.81	2.25E-01
Phvul.005G158200	PTHR37733:SF1 - SMAD/FHA DOMAIN-CONTAINING PROTEIN (1 of 1)	0.08	8.24E-01	-0.38	2.98E-02	0.11	7.56E-01	-0.08	7.23E-01
Phvul.006G199200	PTHR31066:SF2 - GB (1 of 3)	0.08	9.12E-01	0.55	7.60E-02	0.20	7.43E-01	0.74	1.53E-02
Phvul.010G134100	6.6.1.2 - Cobaltochelatase / Hydrogenobyrinic acid a,c-diamide cobaltochelatase (1 of 1)	0.07	8.72E-01	0.54	1.42E-02	-0.08	8.78E-01	0.32	1.99E-01
Phvul.009G217100	PTHR15323 - D123 PROTEIN (1 of 1)	0.07	8.00E-01	0.33	3.67E-02	0.01	9.92E-01	0.05	8.40E-01
Phvul.002G059000	3.1.1.3//3.1.1.32 - Triacylglycerol lipase / Triglyceride lipase // Phospholipase A(1) / Phospholipase A1 (1 of 1)	0.07	8.64E-01	0.18	4.49E-01	0.00	9.99E-01	0.44	3.47E-02
Phvul.009G229800	PTHR24361:SF385 - MITOGEN-ACTIVATED PROTEIN KINASE KINASE 1-RELATED (1 of 2)	0.07	9.41E-01	0.74	9.76E-02	0.11	9.18E-01	1.03	2.00E-02
Phvul.006G188100	PF00083 - Sugar (and other) transporter (Sugar tr) (1 of 45)	0.07	9.15E-01	0.52	9.12E-02	0.08	9.19E-01	0.88	3.24E-03
Phvul.007G214100	PTHR34372:SF2 - CYTOCHROME C OXIDASE SUBUNIT 5C-2-RELATED (1 of 2)	0.07	8.04E-01	-0.10	5.99E-01	0.09	7.77E-01	-0.32	4.35E-02
Phvul.010G100700	PTHR27003:SF117 - CALMODULIN-BINDING RECEPTOR-LIKE CYTOPLASMIC KINASE 2 (1 of 1)	0.07	7.82E-01	0.45	5.37E-04	0.12	6.08E-01	0.39	4.73E-03
Phvul.010G154200	3.5.1.88 - Peptide deformylase / Polypeptide deformylase (1 of 3)	0.07	8.63E-01	-0.42	4.02E-02	-0.02	9.78E-01	-0.37	8.27E-02
Phvul.006G215700	PTHR10217:SF493 - POTASSIUM CHANNEL AKT2/3 (1 of 2)	0.07	8.80E-01	0.34	1.47E-01	0.07	8.99E-01	0.51	2.68E-02
Phvul.004G099100	no data	0.07	9.71E-01	1.49	6.17E-02	-0.46	7.85E-01	1.67	3.68E-02
Phvul.010G078100	PTHR11817//PTHR11817:SF32 - PYRUVATE KINASE // SUBFAMILY NOT NAMED (1 of 1)	0.07	8.13E-01	-0.30	5.35E-02	0.03	9.39E-01	-0.31	4.86E-02
Phvul.011G021900	no data	0.07	9.58E-01	-1.45	3.08E-02	-1.01	2.73E-01	-0.81	2.24E-01
Phvul.002G086400	PTHR24089:SF282 - PEROXISOMAL ADENINE NUCLEOTIDE CARRIER 1-RELATED (1 of 2)	0.07	8.79E-01	0.47	2.20E-02	-0.03	9.63E-01	0.32	1.54E-01
Phvul.002G088200	PTHR24413:SF138 - F-BOX/KELCH-REPEAT PROTEIN SKIP11 (1 of 2)	0.07	8.98E-01	0.49	4.45E-02	0.15	7.58E-01	0.58	2.02E-02
Phvul.002G040300	PTHR13856//PTHR13856:SF93 - VHS DOMAIN CONTAINING PROTEIN FAMILY // SUBFAMILY NOT NAMED (1 of 2)	0.07	8.48E-01	0.39	2.55E-02	0.17	5.75E-01	0.43	1.66E-02
Phvul.006G027500	K17839 - polyamine oxidase (PAO4, PAO3, PAO2) (1 of 5)	0.07	8.13E-01	0.21	2.03E-01	0.14	5.82E-01	0.35	2.08E-02
Phvul.003G209832	6.3.4.5 - Argininosuccinate synthase / Citrulline--aspartate ligase (1 of 2)	0.07	9.10E-01	-0.68	1.23E-02	-0.04	9.54E-01	-0.59	3.63E-02
Phvul.007G194400	K10727 - chromatin licensing and DNA replication factor 1 (CDT1) (1 of 2)	0.07	9.62E-01	-0.43	5.65E-01	-0.28	8.30E-01	-1.41	3.05E-02
Phvul.002G045300	PF07227 - PHD - plant homeodomain finger protein (PHD Oberon) (1 of 11)	0.07	8.46E-01	0.33	5.15E-02	0.22	3.95E-01	0.37	3.21E-02
Phvul.002G262100	1.10.3.2//1.10.3.3 - Laccase / Urishiol oxidase // L-ascorbate oxidase / Ascorbase (1 of 2)	0.07	8.64E-01	0.40	3.13E-02	0.14	6.79E-01	0.26	2.08E-01
Phvul.008G254900	K14696 - solute carrier family 30 (zinc transporter), member 9 (SLC30A9, ZNT9) (1 of 1)	0.07	8.20E-01	-0.14	4.45E-01	0.02	9.66E-01	-0.32	4.04E-02
Phvul.010G149900	PTHR35713:SF1 - GENOMIC DNA, CHROMOSOME 3, P1 CLONE: MOB24 (1 of 1)	0.06	9.07E-01	-0.41	1.26E-01	0.03	9.66E-01	-0.62	1.77E-02
Phvul.009G104000	K03036 - 26S proteasome regulatory subunit N6 (PSMD11, RPN6) (1 of 2)	0.06	7.84E-01	0.27	3.66E-02	0.04	8.96E-01	0.17	2.18E-01
Phvul.003G162900	PTHR19370:SF90 - NADH-CYTOCHROME B5 REDUCTASE 2 (1 of 1)	0.06	8.76E-01	-0.12	6.47E-01	-0.16	6.57E-01	-0.42	4.32E-02
Phvul.003G104100	2.2.1.7 - 1-deoxy-D-xylulose-5-phosphate synthase / DXP-synthase (1 of 6)	0.06	8.85E-01	0.37	8.65E-02	0.09	8.35E-01	0.43	4.38E-02
Phvul.003G195000	PTHR11886//PTHR11886:SF37 - DYNEIN LIGHT CHAIN // SUBFAMILY NOT NAMED (1 of 1)	0.06	9.41E-01	0.85	2.08E-02	0.42	4.95E-01	0.80	3.67E-02
Phvul.008G088200	PTHR13856:SF75 - ENTH/VHS/GAT FAMILY PROTEIN (1 of 2)	0.06	8.93E-01	0.45	3.68E-02	0.03	9.55E-01	0.54	1.36E-02
Phvul.008G172800	PTHR10502:SF98 - ANNEXIN D1-RELATED (1 of 2)	0.06	8.96E-01	0.70	7.24E-04	0.17	6.87E-01	0.55	1.27E-02
Phvul.011G023100	PTHR19241//PTHR19241:SF247 - ATP-BINDING CASSETTE TRANSPORTER // SUBFAMILY NOT NAMED (1 of 5)	0.06	8.70E-01	0.34	6.85E-02	0.00	9.96E-01	0.38	4.40E-02
Phvul.010G053900	PTHR11017//PTHR11017:SF155 - LEUCINE-RICH REPEAT-CONTAINING PROTEIN // SUBFAMILY NOT NAMED (1 of 24)	0.06	8.57E-01	0.16	4.16E-01	0.10	7.76E-01	0.35	4.40E-02
Phvul.007G187000	PTHR12175//PTHR12175:SF2 - AD039 HT014 THIOREDOXIN FAMILY TRP26 // SUBFAMILY NOT NAMED (1 of 1)	0.06	8.55E-01	-0.20	2.82E-01	-0.14	6.39E-01	-0.41	1.66E-02
Phvul.001G058900	PF07800 - Protein of unknown function (DUF1644) (DUF1644) (1 of 7)	0.06	8.78E-01	0.32	8.21E-02	0.04	9.35E-01	0.42	2.24E-02
Phvul.008G292500	PTHR24349:SF83 - CALCIUM-DEPENDENT PROTEIN KINASE 6 (1 of 1)	0.06	8.64E-01	0.35	3.06E-02	-0.02	9.72E-01	0.36	3.05E-02
Phvul.007G079900	PTHR16517:SF31 - TUBBY-LIKE F-BOX PROTEIN 1-RELATED (1 of 2)	0.06	9.15E-01	0.40	1.13E-01	0.11	8.31E-01	0.59	1.58E-02
Phvul.008G058100	PTHR12358:SF43 - SPHINGOID LONG-CHAIN BASES KINASE 2, MITOCHONDRIAL (1 of 1)	0.06	9.19E-01	-0.52	4.01E-02	0.01	9.92E-01	-0.53	4.39E-02
Phvul.006G040700	PF05617 - Prolamin-like (Prolamin like) (1 of 8)	0.06	8.95E-01	0.20	4.00E-01	0.17	6.33E-01	0.43	3.43E-02
Phvul.005G100400	PTHR27000:SF215 - RECEPTOR-LIKE PROTEIN KINASE 5 (1 of 2)	0.06	9.40E-01	0.81	1.28E-02	-0.12	8.80E-01	0.51	1.54E-01
Phvul.001G199400	K02979 - small subunit ribosomal protein S28e (RP-S28e, RPS28) (1 of 2)	0.05	8.78E-01	-0.10	6.56E-01	0.08	8.24E-01	-0.36	4.35E-02
Phvul.011G165700	PF04859 - Plant protein of unknown function (DUF641) (DUF641) (1 of 8)	0.05	9.42E-01	0.77	1.66E-02	0.26	6.68E-01	0.79	1.84E-02
Phvul.010G114500	PTHR10666//PTHR10666:SF138 - UBIQUITIN // SUBFAMILY NOT NAMED (1 of 2)	0.05	8.79E-01	0.34	5.49E-02	0.22	4.10E-01	0.40	2.15E-02
Phvul.004G000700	PTHR22814//PTHR22814:SF47 - COPPER TRANSPORT PROTEIN ATOX1-RELATED // SUBFAMILY NOT NAMED (1 of 8)	0.05	9.19E-01	-0.45	6.03E-02	0.03	9.64E-01	-0.59	1.49E-02
Phvul.009G034000	PTHR22951//PTHR22951:SF24 - CLATHRIN ASSEMBLY PROTEIN // SUBFAMILY NOT NAMED (1 of 2)	0.05	8.94E-01	0.35	5.67E-02	0.44	4.62E-02	0.43	1.80E-02
Phvul.011G159500	PF07460 - NUMOD3 motif (2 copies) (NUMOD3) (1 of 2)	0.05	9.38E-01	-0.71	2.11E-02	0.05	9.55E-01	-0.41	2.20E-01
Phvul.003G175800	K02209 - DNA replication licensing factor MCM5 (MCM5, CDC46) (1 of 1)	0.05	9.64E-01	-0.17	7.93E-01	-0.42	6.17E-01	-1.07	3.61E-02
Phvul.L001676	no data	0.05	9.34E-01	-0.43	1.36E-01	-0.12	8.40E-01	-0.64	2.07E-02
Phvul.004G023700	PF00560//PF07714//PF08263 - Leucine Rich Repeat (LRR 1) // Protein tyrosine kinase (Pkinase Tyr) // Leucine rich repe	0.05	9.06E-01	0.28	1.83E-01	0.11	7.90E-01	0.41	4.46E-02
Phvul.004G104300	no data	0.05	9.08E-01	0.45	2.00E-02	0.02	9.69E-01	0.39	5.73E-02
Phvul.002G209300	PTHR23335:SF12 - CALMODULIN-BINDING TRANSCRIPTION ACTIVATOR 1-RELATED (1 of 2)	0.05	8.70E-01	0.26	9.24E-02	0.01	9.80E-01	0.44	2.91E-03
Phvul.009G067700	KOG0725 - Reductases with broad range of substrate specificities (1 of 51)	0.05	9.62E-01	1.04	8.31E-03	0.13	8.96E-01	0.48	2.92E-01
Phvul.004G029200	K00703 - starch synthase (E2.4.1.21, glgA) (1 of 3)	0.05	9.21E-01	0.47	2.56E-02	0.14	7.46E-01	0.32	1.66E-01
Phvul.007G172700	PF05910 - Plant protein of unknown function (DUF868) (DUF868) (1 of 16)	0.05	9.72E-01	-1.18	4.80E-02	0.06	9.71E-01	-0.64	3.27E-01
Phvul.007G010100	3.2.1.52 - Beta-N-acetylhexosaminidase / N-acetyl-beta-glucosaminidase (1 of 4)	0.05	9.52E-01	-1.01	2.11E-03	-0.13	8.55E-01	-0.80	2.14E-02
Phvul.002G161600	PTHR32468:SF34 - CATION/H(+) ANTIPORTER 18 (1 of 1)	0.05	9.52E-01	0.77	2.12E-02	-0.05	9.55E-01	0.56	1.18E-01
Phvul.007G280800	PTHR31642:SF9 - (Z)-3-HEXEN-1-OL ACETYLTRANSFERASE (1 of 8)	0.05	9.39E-01	0.53	4.99E-02	0.32	4.46E-01	0.73	5.94E-03
Phvul.003G147000	6.2.1.34 - Trans-feruloyl-CoA synthase / Trans-feruloyl-CoA synthetase (1 of 4)	0.05	9.63E-01	1.15	4.08E-03	0.21	8.13E-01	0.98	2.18E-02
Phvul.009G247100	PF00168 - C2 domain (C2) (1 of 105)	0.04	9.15E-01	0.33	8.48E-02	0.17	5.96E-01	0.43	2.59E-02
Phvul.009G063600	PTHR23024:SF131 - CARBOXYLESTERASE 2-RELATED (1 of 2)	0.04	9.29E-01	0.45	2.95E-02	0.20	5.82E-01	0.25	2.65E-01
Phvul.008G051500	PTHR12585:SF29 - FI11703P (1 of 1)	0.04	9.21E-01	0.30	1.24E-01	-0.02	9.77E-01	0.41	3.34E-02
Phvul.002G216900	1.14.13.132//1.14.99.7 - Squalene monooxygenase / Squalene epoxidase (1 of 4)	0.04	9.81E-01	-1.82	8.58E-03	-0.44	7.44E-01	-1.24	8.65E-02
Phvul.009G165700	no data	0.04	9.71E-01	1.04	3.33E-02	-0.36	6.94E-01	0.73	1.78E-01
Phvul.011G059600	PTHR10634//PTHR10634:SF32 - AN1-TYPE ZINC FINGER PROTEIN // SUBFAMILY NOT NAMED (1 of 2)	0.04	9.02E-01	-0.31	4.99E-02	0.01	9.78E-01	-0.36	2.15E-02

Phvul.009G172800	3.6.1.16 - CDP-glycerol diphosphatase / CDP-glycerol pyrophosphatase (1 of 4)	0.04	9.34E-01	0.50	1.31E-02	0.03	9.57E-01	0.41	6.00E-02
Phvul.005G164900	no data	0.04	9.39E-01	0.59	8.00E-03	0.15	7.54E-01	0.57	1.46E-02
Phvul.004G108000	no data	0.04	9.34E-01	-0.34	1.48E-01	-0.16	6.94E-01	-0.47	4.13E-02
Phvul.005G137000	PTHR10782:SF34 - E3 SUMO-PROTEIN LIGASE SIZ1 (1 of 2)	0.04	9.13E-01	0.33	4.52E-02	0.08	8.18E-01	0.20	2.59E-01
Phvul.010G102650	PTHR19241//PTHR19241:SF281 - ATP-BINDING CASSETTE TRANSPORTER // SUBFAMILY NOT NAMED (1 of 2)	0.04	9.36E-01	-0.30	1.93E-01	0.08	8.71E-01	-0.51	2.16E-02
Phvul.008G245600	PTHR27003:SF36 - LEUCINE-RICH REPEAT PROTEIN KINASE-LIKE PROTEIN (1 of 10)	0.04	9.86E-01	1.73	3.03E-02	-0.16	9.51E-01	1.72	3.63E-02
Phvul.003G022800	PF11805 - Protein of unknown function (DUF3326) (DUF3326) (1 of 1)	0.04	9.37E-01	-0.67	1.87E-03	0.21	5.47E-01	-0.49	3.26E-02
Phvul.006G085533	PTHR10887:SF358 - P-LOOP CONTAINING NUCLEOSIDE TRIPHOSPHATE HYDROLASES SUPERFAMILY PROTEIN (1 of 4)	0.04	9.68E-01	0.95	1.41E-02	-0.04	9.74E-01	0.38	4.34E-01
Phvul.003G164800	PF00069//PF00954//PF01453 - Protein kinase domain (Pkinase) // S-locus glycoprotein domain (S locus glycop) // D-ma	0.04	9.66E-01	0.52	1.77E-01	0.34	5.79E-01	0.79	3.45E-02
Phvul.005G059200	no data	0.04	9.24E-01	0.38	3.14E-02	-0.05	9.05E-01	0.26	1.78E-01
Phvul.009G009600	no data	0.04	9.58E-01	0.56	4.81E-02	0.16	7.91E-01	0.37	2.38E-01
Phvul.002G255700	PF15375 - Domain of unknown function (DUF4602) (DUF4602) (1 of 1)	0.04	9.76E-01	-0.93	6.65E-02	-0.05	9.73E-01	-1.30	1.15E-02
Phvul.002G240300	no data	0.03	9.47E-01	-0.41	7.56E-02	0.11	8.13E-01	-0.52	2.72E-02
Phvul.008G158118	PTHR11514:SF58 - TRANSCRIPTION FACTOR SPEECHLESS (1 of 2)	0.03	9.57E-01	-0.69	1.44E-02	0.07	9.22E-01	-0.61	3.93E-02
Phvul.002G267100	PTHR21677 - CRAMPED PROTEIN (1 of 2)	0.03	9.47E-01	-0.47	4.02E-02	0.05	9.29E-01	-0.47	4.90E-02
Phvul.002G096200	PF14111 - Domain of unknown function (DUF4283) (DUF4283) (1 of 81)	0.03	9.87E-01	1.80	3.77E-02	0.35	8.62E-01	1.59	8.43E-02
Phvul.005G180200	K14972 - PAX-interacting protein 1 (PAXIP1, PTIP) (1 of 2)	0.03	9.46E-01	0.53	1.16E-02	0.03	9.56E-01	0.58	6.91E-03
Phvul.004G065700	PTHR24078:SF207 - PROTEIN DNJ-19 (1 of 6)	0.03	9.35E-01	-0.10	6.57E-01	0.01	9.90E-01	-0.39	3.76E-02
Phvul.003G212000	PTHR23322:SF3 - PROTEIN SAY1 (1 of 2)	0.03	9.26E-01	0.25	1.39E-01	0.10	7.36E-01	0.38	1.77E-02
Phvul.002G235000	PF05553 - Cotton fibre expressed protein (DUF761) (1 of 36)	0.03	9.40E-01	0.39	4.40E-02	0.01	9.90E-01	0.32	1.12E-01
Phvul.001G076400	PF00069//PF13947 - Protein kinase domain (Pkinase) // Wall-associated receptor kinase galacturonan-binding (GUB WA	0.03	9.69E-01	0.85	1.55E-02	0.06	9.54E-01	0.78	3.45E-02
Phvul.003G134500	PF04564//PF07714 - U-box domain (U-box) // Protein tyrosine kinase (Pkinase Tyr) (1 of 6)	0.03	9.63E-01	0.72	9.72E-03	-0.06	9.35E-01	0.76	7.86E-03
Phvul.006G102900	PTHR22904//PTHR22904:SF361 - TPR REPEAT CONTAINING PROTEIN // SUBFAMILY NOT NAMED (1 of 1)	0.03	9.51E-01	0.61	4.40E-03	0.11	8.18E-01	0.45	5.01E-02
Phvul.001G246100	PTHR15071 - CATION-DEPENDENT MANNOSE-6-PHOSPHATE RECEPTOR (1 of 1)	0.03	9.54E-01	-0.36	1.43E-01	-0.29	4.42E-01	-0.52	3.30E-02
Phvul.004G106800	PTHR31269:SF2 - S-TYPE ANION CHANNEL SLAH2-RELATED (1 of 2)	0.03	9.67E-01	-0.48	1.39E-01	0.13	8.42E-01	-0.63	4.91E-02
Phvul.009G056200	PTHR13286 - SAP30 (1 of 2)	0.03	9.63E-01	-0.55	4.22E-02	0.00	9.97E-01	-0.58	3.44E-02
Phvul.002G313000	PTHR12383:SF25 - CHLOROPLAST PROCESSING PEPTIDASE (1 of 1)	0.03	9.87E-01	-1.35	6.29E-02	-0.27	8.61E-01	-1.50	4.35E-02
Phvul.008G247400	1.14.13.11 - Trans-cinnamate 4-monooxygenase / Cinnamic acid 4-monooxygenase (1 of 3)	0.03	9.49E-01	0.44	1.91E-02	0.15	6.84E-01	0.35	8.65E-02
Phvul.007G223000	no data	0.03	9.56E-01	-0.43	4.54E-02	-0.03	9.55E-01	-0.54	1.30E-02
Phvul.005G176200	PF01918 - Alba (Alba) (1 of 8)	0.03	9.38E-01	-0.24	1.45E-01	0.02	9.65E-01	-0.34	3.47E-02
Phvul.001G254900	no data	0.03	9.55E-01	-0.46	1.98E-02	0.27	3.98E-01	-0.05	8.75E-01
Phvul.005G001000	PTHR13301//PTHR13301:SF62 - X-BOX TRANSCRIPTION FACTOR-RELATED // SUBFAMILY NOT NAMED (1 of 2)	0.03	9.89E-01	-2.53	2.43E-03	-0.53	7.47E-01	-1.69	5.24E-02
Phvul.003G055100	no data	0.03	9.72E-01	-0.57	7.27E-02	-0.04	9.65E-01	-0.74	2.05E-02
Phvul.002G016400	PTHR11926//PTHR11926:SF384 - GLUCOSYL/GLUCURONOSYL TRANSFERASES // SUBFAMILY NOT NAMED (1 of 11)	0.03	9.87E-01	-1.59	1.53E-02	-0.20	9.00E-01	-1.18	8.85E-02
Phvul.002G323200	PTHR11017//PTHR11017:SF162 - LEUCINE-RICH REPEAT-CONTAINING PROTEIN // SUBFAMILY NOT NAMED (1 of 17)	0.03	9.71E-01	0.33	3.24E-01	-0.05	9.45E-01	0.62	4.08E-02
Phvul.008G252200	PTHR12542//PTHR12542:SF29 - EXOCYST COMPLEX PROTEIN EXO70 // SUBFAMILY NOT NAMED (1 of 5)	0.03	9.55E-01	0.25	2.33E-01	0.12	7.42E-01	0.41	3.53E-02
Phvul.007G012700	K11296 - high mobility group protein B3 (HMGB3) (1 of 1)	0.02	9.85E-01	-0.36	5.90E-01	-0.52	5.64E-01	-1.19	3.62E-02
Phvul.005G082300	K04498 - E1A/CREB-binding protein (EP300, CREBBP, KAT3) (1 of 3)	0.02	9.57E-01	0.43	2.31E-02	-0.03	9.52E-01	0.34	9.40E-02
Phvul.006G129900	PTHR31388:SF22 - PEROXIDASE 22-RELATED (1 of 10)	0.02	9.56E-01	-0.40	2.71E-02	-0.08	8.32E-01	-0.01	9.83E-01
Phvul.008G151818	PTHR31238:SF42 - GERMIN-LIKE PROTEIN SUBFAMILY 1 MEMBER 10-RELATED (1 of 12)	0.02	9.61E-01	-0.37	7.02E-02	-0.14	7.03E-01	-0.43	3.62E-02
Phvul.008G252500	PTHR12542//PTHR12542:SF29 - EXOCYST COMPLEX PROTEIN EXO70 // SUBFAMILY NOT NAMED (1 of 5)	0.02	9.76E-01	0.68	2.27E-02	0.09	8.95E-01	0.76	1.23E-02
Phvul.004G069800	PF12576 - Protein of unknown function (DUF3754) (DUF3754) (1 of 3)	0.02	9.57E-01	0.34	2.97E-02	0.09	7.79E-01	0.20	2.45E-01
Phvul.009G069100	PTHR10177:SF100 - CYCLIN-A3-2-RELATED (1 of 5)	0.02	9.88E-01	-0.46	5.35E-01	-0.54	6.03E-01	-1.31	4.26E-02
Phvul.001G077200	PTHR22950:SF278 - AMINO ACID PERMEASE 1-RELATED (1 of 3)	0.02	9.74E-01	0.48	6.17E-02	-0.06	9.29E-01	0.57	2.56E-02
Phvul.003G048500	PF01936//PF12872 - NYN domain (NYN) // OST-HTH/LOTUS domain (OST-HTH) (1 of 1)	0.02	9.87E-01	-1.10	4.39E-02	0.24	8.23E-01	-0.52	3.80E-01
Phvul.001G009400	PTHR10992//PTHR10992:SF866 - ALPHA/BETA HYDROLASE FOLD-CONTAINING PROTEIN // SUBFAMILY NOT NAMED (1 c	0.02	9.70E-01	0.30	1.79E-01	0.18	6.29E-01	0.44	4.10E-02
Phvul.008G282900	K12872 - pre-mRNA-splicing factor RBM22/SLT11 (RBM22, SLT11) (1 of 2)	0.02	9.73E-01	0.41	3.41E-02	0.07	8.69E-01	0.39	5.54E-02
Phvul.001G050800	PTHR22967//PTHR22967:SF67 - SERINE/THREONINE PROTEIN KINASE // SUBFAMILY NOT NAMED (1 of 2)	0.02	9.77E-01	-0.46	4.22E-02	-0.06	9.04E-01	-0.19	4.56E-01
Phvul.002G154500	K08912 - light-harvesting complex II chlorophyll a/b binding protein 1 (LHCB1) (1 of 13)	0.01	9.89E-01	-0.98	3.62E-02	0.14	8.98E-01	-0.35	5.30E-01
Phvul.001G078501	PTHR10000:SF8 - HAD SUPERFAMILY HYDROLASE-LIKE, TYPE 3 (1 of 1)	0.01	9.80E-01	-0.34	1.22E-01	-0.14	7.35E-01	-0.62	3.44E-03
Phvul.011G015300	K13427 - nitric-oxide synthase, plant (NOA1) (1 of 1)	0.01	9.82E-01	-0.34	1.56E-01	-0.01	9.83E-01	-0.56	1.66E-02
Phvul.004G135400	PTHR19241//PTHR19241:SF271 - ATP-BINDING CASSETTE TRANSPORTER // SUBFAMILY NOT NAMED (1 of 1)	0.01	9.83E-01	0.50	3.25E-02	-0.03	9.69E-01	0.56	2.07E-02
Phvul.004G073450	no data	0.01	9.85E-01	-0.45	6.70E-02	-0.03	9.63E-01	-0.50	4.87E-02
Phvul.002G080100	PTHR11260:SF193 - GLUTATHIONE S-TRANSFERASE U11-RELATED (1 of 2)	0.01	9.92E-01	0.96	9.82E-02	-0.49	6.60E-01	1.17	3.98E-02
Phvul.008G091200	PTHR13832:SF199 - PROTEIN PHOSPHATASE 2C 65-RELATED (1 of 2)	0.01	9.86E-01	0.23	4.30E-01	0.13	8.03E-01	0.52	4.04E-02
Phvul.001G054800	PTHR23023:SF37 - INDOLE-3-PYRUVATE MONOOXYGENASE YUCCA2-RELATED (1 of 4)	0.01	9.89E-01	0.44	8.54E-02	0.43	2.31E-01	0.61	1.74E-02
Phvul.010G000900	PTHR15245//PTHR15245:SF25 - SYMPLEKIN-RELATED // SUBFAMILY NOT NAMED (1 of 1)	0.01	9.92E-01	-0.83	1.77E-02	0.04	9.66E-01	-0.33	4.27E-01
Phvul.005G056300	PF00651 - BTB/POZ domain (BTB) (1 of 51)	0.01	9.90E-01	-0.68	1.09E-02	-0.13	8.20E-01	-0.74	7.47E-03
Phvul.L002643	PTHR19139//PTHR19139:SF55 - AQUAPORIN TRANSPORTER // SUBFAMILY NOT NAMED (1 of 3)	0.01	9.92E-01	0.81	2.28E-02	0.33	5.86E-01	0.33	4.30E-01
Phvul.L001232	PTHR14269:SF15 - HYDROLASE FAMILY PROTEIN / HAD-SUPERFAMILY PROTEIN (1 of 1)	0.01	9.94E-01	-0.76	6.53E-02	-0.30	6.92E-01	-0.91	3.11E-02
Phvul.010G157900	K14972 - PAX-interacting protein 1 (PAXIP1, PTIP) (1 of 2)	0.00	9.94E-01	0.41	6.53E-02	-0.03	9.59E-01	0.49	2.99E-02
Phvul.003G116400	PTHR24012:SF382 - FLOWERING TIME CONTROL PROTEIN FCA-RELATED (1 of 2)	0.00	9.91E-01	0.15	2.66E-01	0.08	7.30E-01	0.26	3.44E-02
Phvul.005G024100	PTHR31265:SF2 - F17A17.37 PROTEIN (1 of 2)	0.00	9.98E-01	-1.27	2.28E-02	-0.16	9.06E-01	-0.98	1.00E-01
Phvul.011G128300	no data	0.00	9.96E-01	0.04	9.07E-01	-0.02	9.82E-01	-0.55	2.92E-02
Phvul.010G069100	PTHR33144:SF3 - PLANT TRANSPOSASE (PTTA/EN/SPM FAMILY) (1 of 7)	0.00	9.98E-01	1.08	1.58E-03	0.05	9.63E-01	0.74	4.42E-02
Phvul.001G227000	PTHR22847:SF424 - TOPLESS-RELATED PROTEIN 2-RELATED (1 of 3)	0.00	9.97E-01	0.39	4.30E-02	-0.03	9.49E-01	0.42	3.60E-02
Phvul.003G215800	PTHR10381:SF15 - ATP-DEPENDENT CLP PROTEASE PROTEOLYTIC SUBUNIT-RELATED PROTEIN 2, CHLOROPLASTIC (1 of 1	0.00	9.96E-01	-0.30	3.99E-02	-0.01	9.77E-01	-0.16	3.35E-01
Phvul.001G000600	K15362 - fanconi anemia group J protein (BRIP1, BACH1, FANCJ) (1 of 1)	0.00	9.98E-01	-0.23	7.64E-01	-0.81	3.30E-01	-1.22	4.40E-02
Phvul.001G151300	PF00514//PF00651//PF13646 - Armadillo/beta-catenin-like repeat (Arm) // BTB/POZ domain (BTB) // HEAT repeats (HE	0.00	9.98E-01	-0.47	4.90E-01	-0.98	2.02E-01	-1.31	2.90E-02
Phvul.009G226400	5.3.1.23 - S-methyl-5-thioribose-1-phosphate isomerase / S-methyl-5-thio-D-ribose-1-phosphate aldose-ketose-isomera	0.00	9.98E-01	-0.27	1.05E-01	0.06	8.50E-01	-0.40	1.52E-02
Phvul.002G091200	K03681 - exosome complex component RRP40 (RRP40, EXOSC3) (1 of 2)	0.00	9.98E-01	-0.20	4.94E-01	-0.14	7.83E-01	-0.69	6.55E-03
Phvul.006G166400	K03861 - phosphatidylinositol glycan, class P (PIGP, GPI19, DSCR5) (1 of 1)	0.00	9.98E-01	-0.20	3.65E-01	-0.06	8.94E-01	-0.43	3.34E-02
Phvul.007G182300	PTHR37764:SF1 - MOG1/PSBP/DUF1795-LIKE PHOTOSYSTEM II REACTION CENTER PSBP FAMILY PROTEIN (1 of 1)	0.00	9.99E-01	-0.53	1.03E-02	0.01	9.93E-01	-0.41	6.20E-02
Phvul.L005143	PF04564//PF07714 - U-box domain (U-box) // Protein tyrosine kinase (Pkinase Tyr) (1 of 6)	0.00	9.98E-01	0.39	4.64E-02	0.08	8.52E-01	0.34	1.02E-01
Phvul.007G134000	PTHR11064:SF27 - NUCLEAR TRANSCRIPTION FACTOR Y SUBUNIT B-2-RELATED (1 of 2)	0.00	9.99E-01	-0.46	1.31E-01	-0.18	7.54E-01	-0.67	2.50E-02
Phvul.011G080900	K12587 - exosome complex component MTR3 (MTR3, EXOSC6) (1 of 1)	0.00	9.98E-01	-0.65	1.53E-02	0.06	9.37E-01	-0.62	2.74E-02
Phvul.005G132600	PTHR27001:SF194 - PROTEIN KINASE FAMILY PROTEIN (1 of 1)	0.00	9.98E-01	-0.91	1.11E-02	0.04	9.71E-01	-0.35	3.75E-01
Phvul.011G051500	PTHR36346:SF1 - EXPRESSED PROTEIN (1 of 2)	0.00	9.96E-01	0.64	1.12E-02	0.34	4.03E-01	0.45	1.05E-01
Phvul.011G193600	PF00931//PF13855 - NB-ARC domain (NB-ARC) // Leucine rich repeat (LRR 8) (1 of 27)	0.00	9.96E-01	-0.44	9.41E-02	-0.13	8.05E-01	-0.53	4.40E-02
Phvul.004G067300	no data	0.00	9.97E-01	-0.79	1.24E-02	-0.16	8.18E-01	-0.88	6.79E-03
Phvul.001G216900	PTHR11132//PTHR11132:SF80 - SOLUTE CARRIER FAMILY 35 // SUBFAMILY NOT NAMED (1 of 5)	0.00	9.94E-01	-0.40	8.09E-02	-0.19	6.38E-01	-0.50	2.80E-02
Phvul.011G094100	PTHR10766:SF60 - SYNTAXIN OF PLANT 111 (1 of 1)	0.00	9.92E-01	-0.45	2.44E-02	-0.35	2.11E-01	-0.52	1.11E-02
Phvul.007G098700	PTHR23315:SF111 - U-BOX DOMAIN-CONTAINING PROTEIN 14 (1 of 1)	0.00	9.92E-01	0.39	1.21E-01	-0.11	8.29E-01	0.59	1.49E-02
Phvul.010G002800	3.2.1.3 - Glucan 1,4-alpha-glucosidase / Lysosomal alpha-glucosidase (1 of 2)	-0.01	9.89E-01	0.18	3.64E-01	0.16	5.86E-01	0.36	4.39E-02
Phvul.005G058000	PTHR13148 - PER1-RELATED (1 of 3)	-0.01	9.98E-01	0.22	8.80E-01	0.56	7.95E-01	2.52	1.12E-02
Phvul.009G180501	PF08263//PF13855 - Leucine rich repeat N-terminal domain (LRRNT 2) // Leucine rich repeat (LRR 8) (1 of 20)	-0.01	9.96E-01	-1.21	4.51E-03	-0.21	8.13E-01	-1.06	1.76E-02
Phvul.006G049200	K02153 - V-type H+-transporting ATPase subunit e (ATPeV0E, ATP6H) (1 of 1)	-0.01	9.86E-01	-0.23	1.72E-01	-0.08	7.97E-01	-0.33	4.30E-02
Phvul.L002244	no data	-0.01	9.90E-01	-0.67	1.05E-02	0.06	9.26E-01	-0.35	2.20E-01
Phvul.003G118450	PTHR10641//PTHR10641:SF594 - MYB-LIKE DNA-BINDING PROTEIN MYB // SUBFAMILY NOT NAMED (1 of 1)	-0.01	9.89E-01	0.59	1.16E-02	0.23	5.88E-01	0.36	1.65E-01
Phvul.008G009300	PTHR12843:SF5 - METHYLTRANSFERASE-LIKE PROTEIN 10 (1 of 1)	-0.01	9.83E-01	0.32	2.41E-02	0.06	8.60E-01	0.20	1.96E-01
Phvul.011G086100	PF09133 - SANTA (SANT Associated) (SANTA) (1 of 2)	-0.01	9.90E-01	-0.43	1.58E-01	-0.24	6.25E-01	-0.74	1.07E-02
Phvul.008G043800	PTHR11743//PTHR11743:SF31 - VOLTAGE-DEPENDENT ANION-SELECTIVE CHANNEL // SUBFAMILY NOT NAMED (1 of 2)	-0.01	9.84E-01	-0.23	2.56E-01	-0.09	8.17E-01	-0.43	2.56E-02
Phvul.009G106200	PTHR27001:SF89 - PROLINE-RICH RECEPTOR-LIKE PROTEIN KINASE PERK1-RELATED (1 of 2)	-0.01	9.96E-01	-1.54	4.35E-02	0.05	9.81E-01	-0.98	2.28E-01
Phvul.006G063700	K14455 - aspartate aminotransferase, mitochondrial (GOT2) (1 of 3)	-0.01	9.92E-01	-1.04	2.00E-02	-0.02	9.90E-01	-0.61	2.14E-01
Phvul.L001937	PTHR11404:SF17 - SUPEROXIDE DISMUTASE [FE] 3, CHLOROPLASTIC (1 of 1)	-0.01	9.87E-01	-0.47	5.56E-02	-0.09	8.71E-01	-0.77	1.44E-03
Phvul.004G032400	3.1.3.2//3.1.3.3//3.1.3.62//3.6.1.1//3.6.1.7 - Acid phosphatase / Phosphomonoesterase // Multiple inositol-polyphospha	-0.01	9.86E-01	-0.48	4.70E-02	0.00	9.97E-01	-0.34	2.00E-01
Phvul.008G145713	PTHR31375:SF31 - F3F20.10 PROTEIN-RELATED (1 of 1)	-0.01	9.86E-01	-0.45	6.33E-02	-0.14	7.66E-01	-0.54	2.82E-02
Phvul.006G036144	PF13962 - Domain of unknown function (PGG) (1 of 69)	-0.01	9.92E-01	1.56	2.76E-02	-0.31	8.06E-01	1.36	7.11E-02
Phvul.005G032700	PTHR21495:SF59 - DIRIGENT PROTEIN 20-RELATED (1 of 4)	-0.01	9.93E-01	-1.39	1.79E-02	-0.04	9.80E-01	-1.07	8.12E-02
Phvul.002G236400	no data	-0.01	9.83E-01	-0.12	6.67E-01	0.03	9.57E-01	-0.45	4.50E-02
Phvul.008G194100	PTHR10774//PTHR10774:SF58 - EXTENDED SYNAPTOTAGMIN-RELATED // SUBFAMILY NOT NAMED (1 of 6)	-0.01	9.82E-01	0.03	9.23E-01	-0.13	7.41E-01	-0.45	4.40E-02
Phvul.006G080700	PTHR31234:SF7 - LATE EMBRYOGENESIS ABUNDANT HYDROXYPROLINE-RICH GLYCOPROTEIN (1 of 1)	-0.01	9.82E-01	-0.42	7.67E-02	-0.10	8.43E-01	-0.48	4.87E-02
Phvul.010G146900	PTHR14009//PTHR14009:SF14 - LEUCINE ZIPPER-EF-HAND CONTAINING TRANSMEMBRANE PROTEIN // SUBFAMILY NOT	-0.01	9.86E-01	-0.72	3.47E-02	-0.38	4.81E-01	-0.45	2.20E-01

Phvul.007G113200	K17255 - Rab GDP dissociation inhibitor (GDI1 2) (1 of 4)	-0.01	9.76E-01	-0.39	4.48E-02	0.08	8.51E-01	-0.14	5.64E-01
Phvul.009G204000	PTHR31992:SF8 - DOF ZINC FINGER PROTEIN DOF1.1-RELATED (1 of 10)	-0.01	9.80E-01	-0.55	1.79E-02	-0.01	9.95E-01	-0.37	1.47E-01
Phvul.009G168500	PTHR10836//PTHR10836:SF44 - GLYCERALDEHYDE 3-PHOSPHATE DEHYDROGENASE // SUBFAMILY NOT NAMED (1 of 2)	-0.02	9.80E-01	-0.20	5.13E-01	-0.09	8.80E-01	-0.59	2.10E-02
Phvul.008G264900	K11086 - small nuclear ribonucleoprotein B and B' (SNRPB, SMB) (1 of 2)	-0.02	9.82E-01	-0.19	5.97E-01	0.10	8.71E-01	-0.63	3.26E-02
Phvul.007G207400	K15030 - translation initiation factor 3 subunit M (EIF3M) (1 of 2)	-0.02	9.67E-01	-0.13	5.26E-01	-0.09	7.89E-01	-0.34	4.87E-02
Phvul.005G033500	PF01535//PF12854//PF13041//PF13812 - PPR repeat (PPR) // PPR repeat (PPR 1) // PPR repeat family (PPR 2) // Penta	-0.02	9.76E-01	-0.59	3.06E-02	-0.12	8.32E-01	-0.34	2.44E-01
Phvul.009G129100	KOG4615 - Uncharacterized conserved protein (1 of 1)	-0.02	9.65E-01	-0.31	9.77E-02	-0.06	8.91E-01	-0.40	3.23E-02
Phvul.008G133700	PTHR11214:SF5 - BETA-1,3-GALACTOSYLTRANSFERASE 2-RELATED (1 of 5)	-0.02	9.68E-01	-0.52	1.03E-02	0.04	9.42E-01	-0.21	3.69E-01
Phvul.009G061350	2.7.11.1 - Non-specific serine/threonine protein kinase / Threonine-specific protein kinase (1 of 1198)	-0.02	9.88E-01	1.27	3.00E-02	-0.05	9.79E-01	1.59	7.18E-03
Phvul.003G178500	PTHR11071:SF244 - CYCLOPHILIN-LIKE PEPTIDYL-PROLYL CIS-TRANS ISOMERASE FAMILY PROTEIN (1 of 1)	-0.02	9.71E-01	-0.62	8.00E-03	0.08	8.76E-01	-0.22	4.43E-01
Phvul.008G076300	K14423 - 4,4-dimethyl-9beta,19-cyclopropylsterol-4alpha-methyl oxidase (SMO1) (1 of 2)	-0.02	9.88E-01	-1.54	8.08E-03	-0.26	8.37E-01	-1.36	2.51E-02
Phvul.007G155500	K02693 - photosystem I subunit IV (psaE) (1 of 2)	-0.02	9.68E-01	-0.44	4.77E-02	0.05	9.33E-01	-0.12	6.61E-01
Phvul.004G072300	K10525 - allene oxide cyclase (AOC) (1 of 3)	-0.02	9.66E-01	-0.34	1.51E-01	-0.07	8.96E-01	-0.51	2.72E-02
Phvul.010G095500	K15095 - (+)-neomenthol dehydrogenase (E1.1.1.208) (1 of 10)	-0.02	9.74E-01	-0.72	1.10E-02	-0.04	9.63E-01	-0.61	4.22E-02
Phvul.006G059254	no data	-0.02	9.68E-01	-0.46	5.93E-02	0.02	9.78E-01	-0.55	2.65E-02
Phvul.008G068800	no data	-0.02	9.51E-01	-0.24	1.96E-01	0.03	9.54E-01	-0.35	4.87E-02
Phvul.008G128700	K00895 - pyrophosphate--fructose-6-phosphate 1-phosphotransferase (E2.7.1.90, pfk) (1 of 4)	-0.03	9.89E-01	-2.25	3.58E-03	-0.40	8.08E-01	-1.39	8.93E-02
Phvul.004G156132	K02993 - small subunit ribosomal protein S7e (RP-S7e, RPS7) (1 of 3)	-0.03	9.44E-01	-0.15	4.14E-01	-0.08	8.13E-01	-0.35	3.43E-02
Phvul.007G009300	4.2.2.2 - Pectate lyase / PPase-N (1 of 26)	-0.03	9.68E-01	0.54	3.87E-02	0.32	4.34E-01	0.43	1.14E-01
Phvul.011G088600	K15365 - RecQ-mediated genome instability protein 2 (RMI2) (1 of 1)	-0.03	9.70E-01	-0.73	1.15E-02	-0.20	7.27E-01	-0.66	2.78E-02
Phvul.007G148200	PTHR13068:SF5 - MITOCHONDRIAL TRANSCRIPTION TERMINATION FACTOR FAMILY PROTEIN (1 of 1)	-0.03	9.44E-01	-0.43	1.07E-02	0.03	9.50E-01	-0.38	3.47E-02
Phvul.006G036200	no data	-0.03	9.64E-01	-0.54	4.25E-02	-0.10	8.60E-01	-0.58	3.28E-02
Phvul.003G204500	PTHR27007:SF30 - L-TYPE LECTIN-DOMAIN CONTAINING RECEPTOR KINASE S.1 (1 of 1)	-0.03	9.64E-01	0.44	9.80E-02	0.32	4.40E-01	0.53	4.58E-02
Phvul.011G177000	3.2.1.39 - Glucan endo-1,3-beta-D-glucosidase / Laminarinase (1 of 65)	-0.03	9.85E-01	1.75	2.72E-03	-0.41	7.35E-01	1.39	2.57E-02
Phvul.006G168118	K02923 - large subunit ribosomal protein L38e (RP-L38e, RPL38) (1 of 3)	-0.03	9.50E-01	-0.26	2.25E-01	-0.04	9.46E-01	-0.41	4.85E-02
Phvul.004G127000	PTHR11638//PTHR11638:SF105 - ATP-DEPENDENT CLP PROTEASE // SUBFAMILY NOT NAMED (1 of 2)	-0.03	9.37E-01	-0.37	2.66E-02	-0.10	7.71E-01	-0.46	6.47E-03
Phvul.011G028501	PTHR23201:SF16 - PROLINE-RICH PROTEIN 4 (1 of 2)	-0.03	9.39E-01	-0.31	1.09E-01	-0.15	6.55E-01	-0.40	3.38E-02
Phvul.001G248100	no data	-0.03	9.34E-01	-0.17	3.99E-01	-0.02	9.68E-01	-0.39	3.16E-02
Phvul.005G167900	KOG0247 - Kinesin-like protein (1 of 1)	-0.03	9.61E-01	-0.70	1.45E-02	-0.13	8.33E-01	-0.53	8.07E-02
Phvul.002G231100	K10695 - E3 ubiquitin-protein ligase RNF1/2 [EC:6.3.2.19] (RNF1 2) (1 of 4)	-0.04	9.33E-01	-0.46	1.12E-02	-0.04	9.37E-01	-0.38	4.78E-02
Phvul.004G108100	no data	-0.04	9.79E-01	-0.01	9.93E-01	-0.51	6.00E-01	-1.26	3.37E-02
Phvul.011G126600	K02868 - large subunit ribosomal protein L11e (RP-L11e, RPL11) (1 of 3)	-0.04	9.34E-01	-0.17	4.63E-01	-0.07	8.83E-01	-0.41	4.87E-02
Phvul.003G011200	PF08041 - PetM family of cytochrome b6f complex subunit 7 (PetM) (1 of 2)	-0.04	9.67E-01	-0.76	4.25E-02	0.13	8.76E-01	-0.64	1.03E-01
Phvul.011G024400	PTHR13305 - RIBOSOME BIOGENESIS PROTEIN NOP10 (1 of 4)	-0.04	9.71E-01	-1.00	2.44E-02	-0.07	9.57E-01	-0.75	1.12E-01
Phvul.011G036300	PTHR10795//PTHR10795:SF442 - PROPROTEIN CONVERTASE SUBTILISIN/KEXIN // SUBFAMILY NOT NAMED (1 of 2)	-0.04	9.24E-01	-0.45	1.14E-02	-0.07	8.57E-01	-0.32	9.04E-02
Phvul.006G176700	PF01933 - Uncharacterised protein family UPF0052 (UPF0052) (1 of 1)	-0.04	9.13E-01	-0.34	4.33E-02	-0.11	7.31E-01	-0.28	1.24E-01
Phvul.002G127600	PTHR21596//PTHR21596:SF8 - RIBONUCLEASE P SUBUNIT P38 // SUBFAMILY NOT NAMED (1 of 1)	-0.04	9.53E-01	-0.69	2.36E-02	-0.25	6.35E-01	-0.59	6.33E-02
Phvul.004G175300	PTHR32295:SF22 - PROTEIN IQ-DOMAIN 25 (1 of 2)	-0.04	9.79E-01	-1.35	3.02E-02	0.18	8.99E-01	-1.38	3.38E-02
Phvul.008G053900	PTHR11079//PTHR11079:SF76 - CYTOSINE DEAMINASE // SUBFAMILY NOT NAMED (1 of 1)	-0.04	9.25E-01	-0.49	9.13E-03	0.00	9.97E-01	-0.33	1.05E-01
Phvul.009G084600	no data	-0.04	9.44E-01	-0.32	2.91E-01	-0.06	9.32E-01	-0.65	2.07E-02
Phvul.002G108300	PF07690 - Major Facilitator Superfamily (MFS 1) (1 of 103)	-0.04	9.38E-01	-0.39	1.37E-01	-0.12	8.18E-01	-0.53	3.63E-02
Phvul.004G172700	2.5.1.46 - Deoxyhypusine synthase / Spermidine dehydrogenase (1 of 1)	-0.04	9.37E-01	-0.04	8.99E-01	-0.10	8.47E-01	-0.52	4.26E-02
Phvul.006G211900	K11547 - kinetochore protein NDC80 (NDC80, HEC1, TID3) (1 of 1)	-0.04	9.39E-01	0.57	2.98E-02	0.03	9.71E-01	0.59	2.85E-02
Phvul.010G026400	PF00931//PF13676 - NB-ARC domain (NB-ARC) // TIR domain (TIR 2) (1 of 30)	-0.04	9.39E-01	0.58	2.51E-02	-0.15	7.78E-01	0.44	1.12E-01
Phvul.007G190800	K01900 - succinyl-CoA synthetase beta subunit (LSC2) (1 of 1)	-0.05	9.03E-01	-0.19	3.33E-01	-0.13	6.76E-01	-0.41	2.09E-02
Phvul.001G107900	no data	-0.05	9.53E-01	-0.80	1.96E-02	-0.13	8.57E-01	-0.42	2.68E-01
Phvul.010G058700	PTHR10025:SF43 - BIFUNCTIONAL PROTEIN FOLD 1, MITOCHONDRIAL (1 of 1)	-0.05	9.84E-01	-2.35	8.34E-03	-0.27	8.94E-01	-1.81	5.00E-02
Phvul.007G052600	PTHR10666:SF155 - POLYUBIQUITIN 4 (1 of 3)	-0.05	8.88E-01	0.23	1.51E-01	0.23	3.29E-01	0.32	4.74E-02
Phvul.007G102300	PTHR12919//PTHR12919:SF23 - 30S RIBOSOMAL PROTEIN S16 // SUBFAMILY NOT NAMED (1 of 1)	-0.05	9.38E-01	-0.65	1.48E-02	-0.01	9.95E-01	-0.52	6.84E-02
Phvul.008G072800	PTHR24057:SF19 - SHAGGY-RELATED PROTEIN KINASE BETA-RELATED (1 of 2)	-0.05	9.00E-01	0.23	2.26E-01	0.08	8.34E-01	0.43	1.80E-02
Phvul.005G175301	PTHR21198 - GLUTAMATE RACEMASE (1 of 2)	-0.05	9.21E-01	-0.46	4.70E-02	0.08	8.58E-01	-0.23	3.63E-01
Phvul.011G038100	K14488 - SAUR family protein (SAUR) (1 of 75)	-0.05	9.15E-01	-0.30	1.73E-01	-0.16	6.82E-01	-0.55	8.91E-03
Phvul.007G192400	PTHR34449:SF2 - ATP BINDING / ATPASE-RELATED (1 of 1)	-0.05	8.88E-01	-0.33	4.87E-02	0.02	9.67E-01	-0.27	1.29E-01
Phvul.009G155300	PTHR10857//PTHR10857:SF18 - COPINE // SUBFAMILY NOT NAMED (1 of 2)	-0.05	9.35E-01	0.49	7.37E-02	0.00	9.99E-01	0.66	1.40E-02
Phvul.010G105400	PTHR10994:SF70 - RETICULON-LIKE PROTEIN B4 (1 of 3)	-0.05	9.21E-01	-0.25	3.15E-01	-0.18	6.65E-01	-0.55	1.79E-02
Phvul.003G279200	PF00373 - FERM central domain (FERM M) (1 of 2)	-0.05	9.23E-01	-0.54	2.11E-02	-0.18	6.66E-01	-0.24	3.65E-01
Phvul.004G046700	K02940 - large subunit ribosomal protein L9e (RP-L9e, RPL9) (1 of 4)	-0.05	9.02E-01	-0.18	4.11E-01	-0.03	9.48E-01	-0.43	3.23E-02
Phvul.002G170300	no data	-0.05	9.75E-01	-1.77	7.57E-03	-0.15	9.32E-01	-1.55	2.72E-02
Phvul.004G054500	PTHR21109 - MITOCHONDRIAL 28S RIBOSOMAL PROTEIN S21 (1 of 2)	-0.05	9.06E-01	-0.44	2.54E-02	0.05	9.11E-01	-0.46	2.55E-02
Phvul.003G066900	PTHR11413:SF38 - CYSTEINE PROTEINASE INHIBITOR 2 (1 of 6)	-0.05	9.36E-01	-0.71	1.95E-02	-0.17	7.76E-01	-0.50	1.23E-01
Phvul.011G045100	PF03087 - Arabidopsis protein of unknown function (DUF241) (1 of 23)	-0.05	9.37E-01	-0.77	1.35E-02	-0.21	7.12E-01	-0.48	1.47E-01
Phvul.003G258900	PTHR10071:SF163 - GATA TRANSCRIPTION FACTOR 14-RELATED (1 of 2)	-0.05	9.20E-01	-0.42	9.29E-02	-0.10	8.40E-01	-0.52	3.42E-02
Phvul.011G141900	PTHR23213:SF195 - FORMIN-LIKE PROTEIN 12-RELATED (1 of 3)	-0.05	8.66E-01	0.24	1.45E-01	-0.05	8.80E-01	0.35	2.72E-02
Phvul.010G148800	K02695 - photosystem I subunit VI (psaH) (1 of 1)	-0.05	9.02E-01	-0.40	4.77E-02	0.02	9.73E-01	-0.12	6.28E-01
Phvul.001G067200	no data	-0.05	8.65E-01	-0.41	8.61E-03	-0.08	8.18E-01	-0.28	9.14E-02
Phvul.007G279600	PTHR24343:SF102 - CBL-INTERACTING SERINE/THREONINE-PROTEIN KINASE 7 (1 of 2)	-0.05	9.04E-01	0.20	4.00E-01	0.48	6.18E-02	0.46	2.87E-02
Phvul.007G215500	K02729 - 20S proteasome subunit alpha 5 (PSMA5) (1 of 2)	-0.05	8.73E-01	-0.13	5.28E-01	-0.06	8.68E-01	-0.36	3.76E-02
Phvul.003G183800	no data	-0.06	9.01E-01	-0.12	6.43E-01	0.08	8.63E-01	-0.44	4.01E-02
Phvul.003G262000	K17435 - large subunit ribosomal protein L54 (MRPL54) (1 of 3)	-0.06	8.93E-01	-0.22	3.13E-01	0.00	1.00E+00	-0.44	2.91E-02
Phvul.009G089400	K02924 - large subunit ribosomal protein L39e (RP-L39e, RPL39) (1 of 3)	-0.06	9.21E-01	-0.31	2.48E-01	-0.09	8.78E-01	-0.56	2.87E-02
Phvul.011G139700	PTHR13048 - TRAFFICKING PROTEIN PARTICLE COMPLEX SUBUNIT 3 (1 of 1)	-0.06	8.68E-01	-0.33	4.87E-02	-0.06	8.81E-01	-0.21	2.41E-01
Phvul.001G140700	PTHR12231:SF111 - MATRIX-REMODELING-ASSOCIATED PROTEIN 8 (1 of 2)	-0.06	8.70E-01	-0.51	1.40E-03	-0.12	6.93E-01	-0.28	1.12E-01
Phvul.006G208900	K02929 - large subunit ribosomal protein L44e (RP-L44e, RPL44) (1 of 2)	-0.06	8.97E-01	-0.36	9.33E-02	0.00	1.00E+00	-0.48	2.23E-02
Phvul.006G151100	3.5.4.4 - Adenosine deaminase / Adenosine aminohydrolase (1 of 2)	-0.06	9.24E-01	-0.61	1.97E-02	-0.16	7.72E-01	-0.37	2.06E-01
Phvul.003G220800	K02983 - small subunit ribosomal protein S30e (RP-S30e, RPS30) (1 of 2)	-0.06	8.93E-01	-0.37	8.35E-02	-0.02	9.75E-01	-0.47	2.42E-02
Phvul.003G177200	KOG0544 - FKBP-type peptidyl-prolyl cis-trans isomerase (1 of 10)	-0.06	8.89E-01	-0.35	1.01E-01	-0.14	7.18E-01	-0.53	1.24E-02
Phvul.011G155700	K08914 - light-harvesting complex II chlorophyll a/b binding protein 3 (LHCB3) (1 of 2)	-0.06	9.15E-01	-0.58	2.00E-02	0.00	9.99E-01	-0.45	8.82E-02
Phvul.001G235600	PF00805 - Pentapeptide repeats (8 copies) (Pentapeptide) (1 of 3)	-0.06	8.80E-01	-0.41	3.67E-02	-0.08	8.38E-01	-0.30	1.57E-01
Phvul.009G042300	PF00069//PF00560//PF08263 - Protein kinase domain (Pkinase) // Leucine Rich Repeat (LRR 1) // Leucine rich repeat N	-0.06	8.86E-01	0.47	1.67E-02	-0.12	7.73E-01	0.25	2.56E-01
Phvul.006G139600	K07199 - 5'-AMP-activated protein kinase, regulatory beta subunit (PRKAB) (1 of 4)	-0.06	8.94E-01	-0.49	2.39E-02	-0.14	7.46E-01	-0.40	8.42E-02
Phvul.009G150600	PF11910 - Cyanobacterial and plant NDH-1 subunit O (NdhO) (1 of 1)	-0.06	9.23E-01	-0.66	1.86E-02	0.02	9.85E-01	-0.45	1.43E-01
Phvul.010G060500	3.4.24.12 - Envelysin / Sea-urchin-hatching proteinase (1 of 4)	-0.06	8.70E-01	-0.41	2.70E-02	-0.13	7.11E-01	-0.42	2.79E-02
Phvul.009G132300	PTHR13856:SF95 - F24J8.3 PROTEIN (1 of 2)	-0.06	8.14E-01	0.29	4.47E-02	-0.07	8.16E-01	0.35	1.45E-02
Phvul.009G227500	K15082 - DNA repair protein RAD7 (RAD7) (1 of 2)	-0.06	9.58E-01	-1.02	8.21E-02	-0.25	8.26E-01	-1.33	2.26E-02
Phvul.007G208000	PTHR12725//PTHR12725:SF73 - HALOACID DEHALOGENASE-LIKE HYDROLASE // SUBFAMILY NOT NAMED (1 of 1)	-0.07	9.70E-01	-2.12	4.17E-03	-0.58	6.69E-01	-1.68	3.28E-02
Phvul.007G273500	K00025 - malate dehydrogenase (MDH1) (1 of 2)	-0.07	9.07E-01	-0.32	2.58E-01	-0.13	8.08E-01	-0.59	2.78E-02
Phvul.010G039200	1.1.1.54//1.3.1.102 - 2-alkenal reductase (NADP(+)) / NADPH-dependent alkenal/one oxidoreductase (1 of 3)	-0.07	8.83E-01	-0.48	3.25E-02	-0.01	9.85E-01	-0.33	1.80E-01
Phvul.010G151900	PTHR10177:SF239 - CYCLIN-A2-3-RELATED (1 of 1)	-0.07	9.51E-01	-1.35	1.90E-02	-0.77	3.08E-01	-0.75	2.03E-01
Phvul.007G251800	PTHR22912:SF48 - NADPH-DEPENDENT THIOREDOXIN REDUCTASE 3 (1 of 1)	-0.07	8.12E-01	-0.31	4.43E-02	0.04	9.29E-01	-0.12	5.28E-01
Phvul.002G031900	PTHR11122:SF12 - ALDOSE 1-EPIMERASE FAMILY PROTEIN (1 of 1)	-0.07	8.24E-01	-0.37	1.85E-02	0.03	9.51E-01	-0.21	2.47E-01
Phvul.009G032700	6.1.1.11 - Serine--tRNA ligase / Seryl-tRNA synthetase (1 of 2)	-0.07	9.09E-01	-0.60	2.97E-02	-0.05	9.47E-01	-0.56	5.10E-02
Phvul.005G105300	PTHR12161:SF18 - REGULATOR OF VPS4 ACTIVITY IN THE MVB PATHWAY PROTEIN (1 of 1)	-0.07	9.43E-01	0.50	2.44E-01	0.32	6.69E-01	0.83	3.43E-02
Phvul.008G173100	PF14111 - Domain of unknown function (DUF4283) (DUF4283) (1 of 81)	-0.07	9.03E-01	-0.67	1.20E-02	-0.05	9.49E-01	-0.93	5.35E-04
Phvul.009G093900	PTHR11132//PTHR11132:SF80 - SOLUTE CARRIER FAMILY 35 // SUBFAMILY NOT NAMED (1 of 5)	-0.07	8.64E-01	0.51	9.13E-03	0.03	9.57E-01	0.38	6.75E-02
Phvul.001G060200	PF01152 - Bacterial-like globin (Bac globin) (1 of 2)	-0.07	8.45E-01	0.43	1.47E-02	0.27	3.13E-01	0.47	1.06E-02
Phvul.008G289900	1.3.3.3 - Coproporphyrinogen oxidase / Coproporphyrinogenase (1 of 1)	-0.07	8.85E-01	-0.60	9.15E-03	-0.07	8.94E-01	-0.51	3.73E-02
Phvul.007G242200	KOG4372 - Predicted alpha/beta hydrolase (1 of 4)	-0.07	8.72E-01	-0.43	4.72E-02	-0.15	7.12E-01	-0.64	3.30E-03
Phvul.002G017100	no data	-0.07	8.68E-01	-0.42	4.54E-02	0.06	8.98E-01	-0.20	4.11E-01
Phvul.006G179800	PF00847 - AP2 domain (AP2) (1 of 180)	-0.07	9.15E-01	0.64	3.41E-02	0.21	7.26E-01	0.84	5.53E-03
Phvul.007G180000	PF09353 - Domain of unknown function (DUF1995) (DUF1995) (1 of 5)	-0.07	8.20E-01	-0.47	3.75E-03	0.00	9.94E-01	-0.24	1.84E-01
Phvul.011G179800	PTHR10794:SF53 - CAAX AMINO TERMINAL PROTEASE FAMILY PROTEIN (1 of 2)	-0.07	8.12E-01	-0.53	1.12E-03	-0.24	3.42E-01	-0.37	3.28E-02
Phvul.008G283400	PTHR33791:SF4 - CHAPERONIN-LIKE RBCX PROTEIN (1 of 2)	-0.07	8.73E-01	-0.46	4.58E-02	-0.01	9.86E-01	-0.34	1.63E-01
Phvul.009G020500	4.1.1.17 - Ornithine decarboxylase / L-ornithine carboxy-lyase (1 of 2)	-0.08	9.61E-01	-1.73	4.32E-02	0.35	8.08E-01	-0.97	2.55E-01

Phvul.003G232500	no data	-0.08	8.78E-01	-0.32	2.32E-01	-0.29	4.52E-01	-0.58	2.42E-02
Phvul.008G092900	PTHR11206:SF101 - MATE EFFLUX FAMILY PROTEIN 2, CHLOROPLASTIC (1 of 1)	-0.08	8.76E-01	-0.60	8.34E-03	-0.16	7.23E-01	-0.75	1.31E-03
Phvul.008G002250	PTHR12741:SF21 - CALLOSE SYNTHASE 3 (1 of 8)	-0.08	8.77E-01	-0.70	2.33E-03	-0.11	8.35E-01	-0.65	7.20E-03
Phvul.007G141100	K14944 - RNA-binding protein Nova (NOVA) (1 of 1)	-0.08	8.06E-01	-0.25	1.58E-01	-0.13	6.80E-01	-0.37	3.27E-02
Phvul.005G062700	no data	-0.08	7.96E-01	-0.34	3.88E-02	-0.02	9.61E-01	-0.02	9.36E-01
Phvul.008G050400	PTHR11177:SF167 - RHODANESE-LIKE DOMAIN-CONTAINING PROTEIN 4, CHLOROPLASTIC (1 of 2)	-0.08	8.49E-01	-0.43	4.11E-02	-0.03	9.63E-01	-0.38	7.83E-02
Phvul.001G161600	PTHR10694//PTHR10694:SF53 - JUMONJI DOMAIN CONTAINING PROTEIN // SUBFAMILY NOT NAMED (1 of 3)	-0.08	7.56E-01	-0.22	1.61E-01	-0.23	2.70E-01	-0.30	4.60E-02
Phvul.009G260200	K08908 - light-harvesting complex I chlorophyll a/b binding protein 2 (LHCA2) (1 of 3)	-0.08	8.97E-01	-0.65	2.09E-02	0.07	9.26E-01	-0.44	1.50E-01
Phvul.004G067925	PTHR24031:SF42 - ATP-DEPENDENT RNA HELICASE DDX59-RELATED (1 of 1)	-0.08	8.87E-01	-0.31	2.76E-01	0.00	9.98E-01	-0.55	3.89E-02
Phvul.010G112200	PF11282 - Protein of unknown function (DUF3082) (DUF3082) (1 of 2)	-0.08	8.67E-01	-0.80	2.20E-04	-0.14	7.62E-01	-0.76	6.31E-04
Phvul.003G201200	PTHR14209//PTHR14209:SF2 - ISOAMYL ACETATE-HYDROLYZING ESTERASE 1 // SUBFAMILY NOT NAMED (1 of 1)	-0.08	7.94E-01	-0.37	2.59E-02	-0.19	4.80E-01	-0.44	9.79E-03
Phvul.006G024100	PTHR13068:SF28 - MITOCHONDRIAL TRANSCRIPTION TERMINATION FACTOR FAMILY PROTEIN-RELATED (1 of 14)	-0.08	8.94E-01	-0.58	3.85E-02	0.02	9.84E-01	-0.34	2.81E-01
Phvul.003G151100	PTHR23091//PTHR23091:SF234 - N-TERMINAL ACETYLTRANSFERASE // SUBFAMILY NOT NAMED (1 of 1)	-0.08	8.14E-01	-0.36	4.46E-02	-0.14	6.65E-01	-0.39	3.77E-02
Phvul.007G247900	1.3.2.3 - L-galactonolactone dehydrogenase / L-galactono-gamma-lactone dehydrogenase (1 of 1)	-0.08	7.52E-01	-0.23	1.33E-01	-0.11	6.60E-01	-0.34	1.90E-02
Phvul.005G173700	PTHR19241:SF219 - ABC TRANSPORTER G FAMILY MEMBER 38 (1 of 18)	-0.08	9.76E-01	1.80	1.85E-02	0.18	9.43E-01	1.54	5.41E-02
Phvul.003G018100	PF07058 - Microtubule-associated protein 70 (MAP70) (1 of 7)	-0.08	7.76E-01	-0.35	2.56E-02	-0.08	8.07E-01	-0.25	1.39E-01
Phvul.004G120400	PTHR22936:SF35 - MEMBRANE PROTEIN-RELATED (1 of 2)	-0.08	8.57E-01	0.22	4.13E-01	-0.03	9.67E-01	0.53	2.34E-02
Phvul.006G166100	PTHR22939:SF76 - PROTEASE DO-LIKE 5, CHLOROPLASTIC (1 of 1)	-0.08	7.69E-01	-0.35	2.18E-02	-0.01	9.92E-01	-0.31	5.43E-02
Phvul.006G109400	PTHR10460 - ABL INTERACTOR (1 of 5)	-0.08	9.15E-01	-0.73	3.56E-02	-0.62	2.14E-01	-0.69	5.75E-02
Phvul.L001681	PTHR22981:SF53 - GLYOXYLATE/SUCCINIC SEMIALDEHYDE REDUCTASE 2, CHLOROPLASTIC (1 of 4)	-0.08	9.02E-01	-0.83	8.32E-03	0.29	5.76E-01	-0.34	3.12E-01
Phvul.003G071700	PTHR36308:SF1 - DENTIN SIALOPHOSPHOPROTEIN-LIKE PROTEIN-RELATED (1 of 1)	-0.08	9.15E-01	-0.44	2.61E-01	-0.18	8.04E-01	-0.74	5.00E-02
Phvul.007G015000	PTHR31072:SF7 - TRANSCRIPTION FACTOR TCP21-RELATED (1 of 3)	-0.08	8.63E-01	-0.43	7.56E-02	-0.13	7.92E-01	-0.50	4.52E-02
Phvul.003G023800	PTHR35483:SF1 - GLYCINE-RICH PROTEIN (1 of 2)	-0.08	7.62E-01	-0.37	1.66E-02	-0.10	7.31E-01	-0.24	1.46E-01
Phvul.011G074800	1.3.99.12 - 2-methylacyl-CoA dehydrogenase / Branched-chain acyl-CoA dehydrogenase (1 of 1)	-0.09	9.24E-01	0.90	2.50E-02	-0.08	9.37E-01	0.56	2.06E-01
Phvul.008G049200	KOG0884 - Similar to cyclophilin-type peptidyl-prolyl cis-trans isomerase (1 of 4)	-0.09	8.79E-01	-0.28	3.94E-01	-0.18	7.40E-01	-0.62	3.36E-02
Phvul.011G090400	PTHR21022//PTHR21022:SF5 - PREPHENATE DEHYDRATASE P PROTEIN // SUBFAMILY NOT NAMED (1 of 2)	-0.09	9.09E-01	0.80	1.73E-02	0.14	8.43E-01	0.91	6.75E-03
Phvul.008G083000	2.7.11.1 - Non-specific serine/threonine protein kinase / Threonine-specific protein kinase (1 of 1198)	-0.09	8.43E-01	0.58	5.03E-03	-0.01	9.94E-01	0.60	5.06E-03
Phvul.001G079100	PTHR23023:SF37 - INDOLE-3-PYRUVATE MONOOXYGENASE YUCCA2-RELATED (1 of 4)	-0.09	8.30E-01	-0.30	1.75E-01	-0.18	6.28E-01	-0.56	7.00E-03
Phvul.005G067500	K03152 - 4-methyl-5(b-hydroxyethyl)-thiazole monophosphate biosynthesis (thiJ) (1 of 3)	-0.09	7.77E-01	-0.29	9.75E-02	-0.08	8.10E-01	-0.35	4.46E-02
Phvul.003G162000	no data	-0.09	8.66E-01	-0.54	3.73E-02	-0.17	7.36E-01	-0.42	1.19E-01
Phvul.006G066400	PTHR11709:SF103 - LACCASE-16 (1 of 7)	-0.09	9.49E-01	-0.78	2.58E-01	-0.79	4.11E-01	-1.32	4.76E-02
Phvul.003G249800	K02716 - photosystem II oxygen-evolving enhancer protein 1 (psbO) (1 of 2)	-0.09	8.28E-01	-0.47	2.25E-02	0.09	8.44E-01	-0.16	5.30E-01
Phvul.002G106200	PTHR31704:SF3 - PROTEIN MID1-COMPLEMENTING ACTIVITY 1-RELATED (1 of 2)	-0.09	8.56E-01	-0.58	1.50E-02	-0.19	6.66E-01	-0.52	4.08E-02
Phvul.009G151900	PF03364 - Polyketide cyclase / dehydrase and lipid transport (Polyketide cyc) (1 of 5)	-0.09	8.14E-01	-0.40	4.61E-02	-0.17	6.42E-01	-0.30	1.67E-01
Phvul.001G032000	no data	-0.09	7.89E-01	-0.28	1.54E-01	-0.22	4.54E-01	-0.38	4.28E-02
Phvul.009G178000	PTHR11453//PTHR11453:SF51 - ANION EXCHANGE PROTEIN // SUBFAMILY NOT NAMED (1 of 2)	-0.09	7.96E-01	-0.43	2.11E-02	-0.24	4.16E-01	-0.40	3.79E-02
Phvul.010G120700	PTHR31989:SF5 - F11O4.3-RELATED (1 of 8)	-0.09	9.02E-01	0.64	1.41E-01	-0.41	4.72E-01	0.91	2.10E-02
Phvul.009G260300	PTHR32440:SF0 - PHOSPHATASE DCR2-RELATED (1 of 2)	-0.09	7.98E-01	-0.38	5.20E-02	-0.21	4.95E-01	-0.39	4.87E-02
Phvul.011G096400	K14332 - photosystem I subunit PsaO (psaO) (1 of 1)	-0.09	8.04E-01	-0.51	7.57E-03	0.03	9.63E-01	-0.28	1.88E-01
Phvul.007G021200	4.1.1.33 - Diphosphomevalonate decarboxylase / Mevalonate pyrophosphate decarboxylase (1 of 1)	-0.09	8.58E-01	-0.55	3.33E-02	-0.33	4.04E-01	-0.48	7.60E-02
Phvul.009G234300	PTHR11132:SF75 - GB (1 of 2)	-0.09	8.64E-01	-0.77	4.49E-03	-0.19	7.01E-01	-0.88	1.40E-03
Phvul.009G144100	PTHR13683:SF259 - ASPARTYL PROTEASE-LIKE PROTEIN (1 of 2)	-0.09	9.70E-01	-2.20	4.27E-02	-0.50	8.20E-01	-1.86	1.07E-01
Phvul.011G111800	no data	-0.09	8.20E-01	-0.37	1.01E-01	-0.10	8.17E-01	-0.54	1.33E-02
Phvul.001G257600	PF05627 - Cleavage site for pathogenic type III effector avirulence factor Avr (AvrRpt-cleavage) (1 of 10)	-0.09	9.38E-01	-1.40	4.27E-02	-0.59	5.28E-01	-0.60	4.01E-01
Phvul.011G047200	K17261 - adenylyl cyclase-associated protein (CAP1 2, SRV2) (1 of 1)	-0.09	8.91E-01	0.97	1.73E-03	0.06	9.44E-01	0.75	2.57E-02
Phvul.008G116300	PTHR11709:SF66 - LACCASE-17 (1 of 2)	-0.09	8.97E-01	-0.87	8.71E-03	-0.19	7.81E-01	-0.61	9.09E-02
Phvul.003G263500	no data	-0.09	9.29E-01	-0.29	6.02E-01	-0.14	9.00E-01	-0.99	3.64E-02
Phvul.008G285100	K08913 - light-harvesting complex II chlorophyll a/b binding protein 2 (LHCB2) (1 of 1)	-0.10	8.77E-01	-0.60	4.87E-02	0.12	8.49E-01	-0.24	5.10E-01
Phvul.008G135600	PTHR24351:SF69 - AGC (CAMP-DEPENDENT, CGMP-DEPENDENT AND PROTEIN KINASE C) KINASE FAMILY PROTEIN-RELA	-0.10	8.68E-01	-0.44	1.67E-01	-0.02	9.88E-01	-0.77	1.25E-02
Phvul.009G219500	PTHR11132//PTHR11132:SF80 - SOLUTE CARRIER FAMILY 35 // SUBFAMILY NOT NAMED (1 of 5)	-0.10	7.08E-01	-0.36	1.95E-02	-0.23	3.02E-01	-0.36	2.26E-02
Phvul.008G144000	no data	-0.10	9.16E-01	-1.06	3.57E-02	-0.18	8.42E-01	-0.45	4.11E-01
Phvul.005G055100	K03541 - photosystem II 10kDa protein (psbR) (1 of 1)	-0.10	7.91E-01	-0.49	1.86E-02	0.00	9.97E-01	-0.18	4.53E-01
Phvul.007G182400	PTHR30373:SF2 - UPF0603 PROTEIN YGCG (1 of 1)	-0.10	7.72E-01	-0.38	4.74E-02	0.03	9.58E-01	-0.10	6.75E-01
Phvul.009G057900	no data	-0.10	7.76E-01	-0.43	2.37E-02	-0.14	7.11E-01	-0.22	3.03E-01
Phvul.005G006300	PTHR10903:SF65 - AIG1 DOMAIN-CONTAINING PROTEIN-RELATED (1 of 6)	-0.10	9.21E-01	-0.83	8.60E-02	-0.24	8.03E-01	-1.10	2.39E-02
Phvul.010G006700	no data	-0.10	8.49E-01	-0.50	7.03E-02	-0.28	5.38E-01	-0.65	1.87E-02
Phvul.004G153400	PTHR11005//PTHR11005:SF30 - LYSOSOMAL ACID LIPASE-RELATED // SUBFAMILY NOT NAMED (1 of 1)	-0.10	8.84E-01	-0.85	1.20E-02	-0.17	8.15E-01	-0.39	3.12E-01
Phvul.011G117000	PTHR10909:SF11 - ACYL-COENZYME A OXIDASE-LIKE PROTEIN (1 of 1)	-0.10	9.33E-01	-1.38	1.88E-02	-0.17	8.91E-01	-1.00	9.08E-02
Phvul.001G088800	no data	-0.10	7.39E-01	-0.37	3.40E-02	-0.09	7.96E-01	-0.30	1.00E-01
Phvul.003G044500	2.1.2.5 - Glutamate formimidoyltransferase / Glutamate formyltransferase (1 of 2)	-0.10	8.22E-01	-0.39	1.33E-01	-0.24	5.62E-01	-0.60	1.74E-02
Phvul.L005643	5.3.1.1 - Triose-phosphate isomerase / Triosephosphate mutase (1 of 2)	-0.10	8.72E-01	-0.54	1.00E-01	-0.09	9.06E-01	-0.66	4.21E-02
Phvul.006G131500	K11717 - cysteine desulfurase / selenocysteine lyase (sufS) (1 of 1)	-0.11	8.66E-01	-0.80	7.47E-03	-0.31	5.47E-01	-0.51	1.12E-01
Phvul.010G024600	K02995 - small subunit ribosomal protein S8e (RP-S8e, RPS8) (1 of 3)	-0.11	7.58E-01	-0.28	1.68E-01	0.03	9.61E-01	-0.40	4.25E-02
Phvul.007G225600	PTHR11062//PTHR11062:SF66 - EXOSTOSIN HEPARAN SULFATE GLYCOSYLTRANSFERASE -RELATED // SUBFAMILY NOT N	-0.11	7.40E-01	-0.46	8.77E-03	-0.11	7.69E-01	-0.31	9.99E-02
Phvul.002G257400	no data	-0.11	7.65E-01	-0.39	4.59E-02	0.05	9.25E-01	-0.14	5.65E-01
Phvul.003G075100	no data	-0.11	7.63E-01	-0.41	3.59E-02	0.06	9.04E-01	-0.23	2.93E-01
Phvul.009G050400	PTHR14136:SF11 - THYLAKOID LUMENAL 17.4 KDA PROTEIN, CHLOROPLASTIC (1 of 1)	-0.11	7.09E-01	-0.40	1.25E-02	-0.02	9.73E-01	-0.20	2.69E-01
Phvul.007G136700	no data	-0.11	7.61E-01	-0.46	1.64E-02	0.09	8.28E-01	-0.32	1.15E-01
Phvul.003G164400	PF11317 - Protein of unknown function (DUF3119) (DUF3119) (1 of 1)	-0.11	6.88E-01	-0.39	9.72E-03	-0.02	9.63E-01	-0.25	1.31E-01
Phvul.002G011300	PTHR11743:SF29 - MITOCHONDRIAL OUTER MEMBRANE PROTEIN PORIN 2-RELATED (1 of 1)	-0.11	8.24E-01	-0.48	6.39E-02	-0.07	9.09E-01	-0.57	2.93E-02
Phvul.005G130000	K09646 - serine carboxypeptidase 1 [EC:3.4.16.-] (SCPEP1) (1 of 2)	-0.11	8.29E-01	-0.71	5.27E-03	-0.24	6.00E-01	-0.45	1.07E-01
Phvul.005G184700	PTHR27000:SF185 - LEUCINE-RICH REPEAT RECEPTOR-LIKE PROTEIN KINASE PEPR1-RELATED (1 of 3)	-0.11	7.84E-01	-0.48	3.82E-02	-0.20	6.04E-01	-0.11	6.87E-01
Phvul.003G253800	K06287 - septum formation protein (maf) (1 of 1)	-0.11	8.56E-01	-0.65	3.12E-02	-0.09	8.93E-01	-0.54	8.95E-02
Phvul.006G084500	PF07466 - Protein of unknown function (DUF1517) (DUF1517) (1 of 2)	-0.11	9.20E-01	1.35	4.21E-03	-0.48	5.64E-01	0.83	1.12E-01
Phvul.002G162600	PTHR33374:SF2 - ARABINOGALACTAN PEPTIDE 22-RELATED (1 of 1)	-0.11	9.42E-01	-1.73	9.03E-03	-0.08	9.64E-01	-1.23	8.12E-02
Phvul.002G052700	PTHR13844:SF8 - EXPRESSED PROTEIN (1 of 1)	-0.11	8.79E-01	-0.74	3.33E-02	-0.13	8.61E-01	-0.73	4.35E-02
Phvul.009G060400	PTHR11850:SF111 - BEL1-LIKE HOMEODOMAIN PROTEIN 1-RELATED (1 of 6)	-0.11	8.67E-01	-0.78	1.29E-02	0.07	9.33E-01	-0.79	1.49E-02
Phvul.002G223300	PTHR22835//PTHR22835:SF219 - ZINC FINGER FYVE DOMAIN CONTAINING PROTEIN // SUBFAMILY NOT NAMED (1 of 1)	-0.11	8.28E-01	-0.78	2.51E-03	-0.16	7.64E-01	-0.59	3.42E-02
Phvul.002G108000	1.6.99.1 - NADPH dehydrogenase / NADPH diaphorase (1 of 2)	-0.11	7.14E-01	-0.42	1.31E-02	-0.09	8.15E-01	-0.26	1.73E-01
Phvul.004G144500	PTHR11999:SF113 - GLUTAMATE DECARBOXYLASE 2 (1 of 2)	-0.11	8.47E-01	-0.68	1.62E-02	-0.08	9.03E-01	-0.52	8.96E-02
Phvul.001G152000	PTHR28039:SF7 - FATTY-ACID-BINDING PROTEIN 1 (1 of 1)	-0.11	7.40E-01	-0.59	1.56E-03	-0.06	9.02E-01	-0.38	5.93E-02
Phvul.005G018600	K02966 - small subunit ribosomal protein S19e (RP-S19e, RPS19) (1 of 3)	-0.11	8.22E-01	-0.33	2.47E-01	-0.04	9.50E-01	-0.57	3.42E-02
Phvul.001G194020	PTHR33057:SF17 - GB (1 of 4)	-0.11	9.50E-01	-1.92	2.00E-02	-0.16	9.40E-01	-0.84	3.65E-01
Phvul.009G111700	6.1.1.2 - Tryptophan--tRNA ligase / Tryptophanyl-tRNA synthetase (1 of 2)	-0.11	7.73E-01	-0.46	3.21E-02	-0.13	7.67E-01	-0.40	7.97E-02
Phvul.006G196600	PTHR23042:SF67 - TRANSCRIPTION FACTOR ILR3-RELATED (1 of 3)	-0.11	8.98E-01	-0.88	3.39E-02	0.01	9.97E-01	-0.86	4.53E-02
Phvul.005G182200	K02951 - small subunit ribosomal protein S12e (RP-S12e, RPS12) (1 of 3)	-0.11	8.21E-01	-0.29	3.19E-01	-0.05	9.50E-01	-0.58	3.03E-02
Phvul.009G126600	PTHR21450:SF8 - F8K7.18 PROTEIN (1 of 1)	-0.11	8.70E-01	-0.85	2.07E-02	-0.46	3.95E-01	-0.37	3.53E-01
Phvul.004G151900	PTHR34056:SF1 - GENOMIC DNA, CHROMOSOME 3, P1 CLONE: MRC8 (1 of 2)	-0.11	7.91E-01	-0.38	1.21E-01	-0.19	6.52E-01	-0.55	2.26E-02
Phvul.010G142300	PTHR10544//PTHR10544:SF1 - 60S RIBOSOMAL PROTEIN L28 // SUBFAMILY NOT NAMED (1 of 1)	-0.11	7.96E-01	-0.33	1.99E-01	-0.07	9.01E-01	-0.53	2.93E-02
Phvul.003G132300	K02957 - small subunit ribosomal protein S15Ae (RP-S15Ae, RPS15A) (1 of 5)	-0.11	7.85E-01	-0.38	1.08E-01	-0.16	7.27E-01	-0.65	4.84E-03
Phvul.003G189700	PTHR11540:SF14 - MALATE DEHYDROGENASE, CHLOROPLASTIC (1 of 1)	-0.12	7.81E-01	-0.40	8.71E-02	-0.08	8.71E-01	-0.59	1.01E-02
Phvul.006G055300	K02946 - small subunit ribosomal protein S10 (RP-S10, MRPS10, rpsJ) (1 of 2)	-0.12	7.17E-01	-0.47	8.97E-03	-0.08	8.35E-01	-0.37	5.63E-02
Phvul.008G159500	K02991 - small subunit ribosomal protein S6e (RP-S6e, RPS6) (1 of 5)	-0.12	8.13E-01	-0.56	3.12E-02	-0.27	5.21E-01	-0.64	1.61E-02
Phvul.011G070200	PTHR22814//PTHR22814:SF82 - COPPER TRANSPORT PROTEIN ATOX1-RELATED // SUBFAMILY NOT NAMED (1 of 2)	-0.12	6.88E-01	-0.48	3.32E-03	-0.09	7.96E-01	-0.47	5.98E-03
Phvul.009G258000	K13126 - polyadenylate-binding protein (PABPC) (1 of 8)	-0.12	7.70E-01	-0.52	1.66E-02	-0.08	8.78E-01	-0.48	3.44E-02
Phvul.007G258400	PF03110 - SBP domain (SBP) (1 of 23)	-0.12	7.37E-01	-0.30	1.38E-01	-0.12	7.72E-01	-0.42	3.37E-02
Phvul.010G040300	PTHR10210:SF58 - RIBOSE-PHOSPHATE PYROPHOSPHOKINASE 5, CHLOROPLASTIC (1 of 1)	-0.12	6.46E-01	-0.32	3.51E-02	-0.03	9.41E-01	-0.26	1.15E-01
Phvul.002G189800	2.5.1.78 - 6,7-dimethyl-8-ribityllumazine synthase / Lumazine synthase (1 of 1)	-0.12	7.33E-01	-0.40	4.86E-02	-0.05	9.30E-01	-0.43	3.46E-02
Phvul.006G033600	PTHR24055:SF158 - MITOGEN-ACTIVATED PROTEIN KINASE 20 (1 of 2)	-0.12	7.53E-01	-0.48	2.00E-02	-0.20	5.86E-01	-0.39	7.72E-02
Phvul.007G274200	K02936 - large subunit ribosomal protein L7Ae (RP-L7Ae, RPL7A) (1 of 2)	-0.12	7.06E-01	-0.28	1.50E-01	-0.01	9.80E-01	-0.44	1.91E-02
Phvul.002G236300	K02912 - large subunit ribosomal protein L32e (RP-L32e, RPL32) (1 of 3)	-0.12	8.58E-01	-0.57	1.15E-01	-0.35	5.28E-01	-0.73	3.98E-02
Phvul.007G211700	K07937//K07977 - ADP-ribosylation factor 1 (ARF1) // Arf/Sar family, other (ARF) (1 of 6)	-0.12	7.37E-01	-0.38	6.88E-02	-0.04	9.43E-01	-0.44	3.59E-02
Phvul.001G229400	no data	-0.12	9.29E-01	-1.33	4.28E-02	0.13	9.33E-01	-0.77	2.65E-01

Phvul.008G288300	K17778 - mitochondrial import inner membrane translocase subunit TIM10 (TIM10) (1 of 2)	-0.12	6.82E-01	-0.18	3.59E-01	-0.08	8.18E-01	-0.37	4.42E-02
Phvul.003G164500	no data	-0.12	6.67E-01	-0.47	3.70E-03	-0.02	9.59E-01	-0.28	1.18E-01
Phvul.009G168400	PTHR10836//PTHR10836:SF44 - GLYCERALDEHYDE 3-PHOSPHATE DEHYDROGENASE // SUBFAMILY NOT NAMED (1 of 2)	-0.12	8.20E-01	-0.41	1.65E-01	-0.18	7.40E-01	-0.75	8.41E-03
Phvul.010G152600	PF11145 - Protein of unknown function (DUF2921) (DUF2921) (1 of 6)	-0.12	7.37E-01	-0.37	8.04E-02	-0.33	2.77E-01	-0.45	3.50E-02
Phvul.004G054300	no data	-0.12	7.64E-01	-0.26	2.97E-01	-0.23	5.48E-01	-0.62	7.53E-03
Phvul.004G175800	PF00069//PF00560//PF08263//PF13855 - Protein kinase domain (Pkinase) // Leucine Rich Repeat (LRR 1) // Leucine ric	-0.12	8.10E-01	0.55	4.60E-02	-0.12	8.39E-01	0.36	2.28E-01
Phvul.006G122600	K07937 - ADP-ribosylation factor 1 (ARF1) (1 of 8)	-0.12	8.11E-01	0.70	8.15E-03	0.00	9.99E-01	0.55	5.61E-02
Phvul.007G230200	no data	-0.12	7.76E-01	-0.42	8.37E-02	-0.12	8.11E-01	-0.58	1.72E-02
Phvul.008G196500	PTHR13683:SF250 - ASPARTYL PROTEASE-LIKE PROTEIN (1 of 3)	-0.12	7.27E-01	-0.46	2.01E-02	-0.02	9.65E-01	-0.04	8.91E-01
Phvul.011G070000	PTHR22814//PTHR22814:SF82 - COPPER TRANSPORT PROTEIN ATOX1-RELATED // SUBFAMILY NOT NAMED (1 of 2)	-0.12	8.56E-01	-0.82	1.42E-02	-0.20	7.75E-01	-0.56	1.22E-01
Phvul.009G254800	K02905 - large subunit ribosomal protein L29e (RP-L29e, RPL29) (1 of 2)	-0.12	7.67E-01	-0.28	2.71E-01	-0.04	9.50E-01	-0.53	2.43E-02
Phvul.007G231600	PTHR32080:SF4 - CYSTEINE-RICH REPEAT SECRETORY PROTEIN 11-RELATED (1 of 2)	-0.12	7.18E-01	-0.49	1.03E-02	-0.01	9.86E-01	-0.32	1.31E-01
Phvul.011G042400	PF00643 - B-box zinc finger (zf-B box) (1 of 25)	-0.12	6.86E-01	-0.31	1.12E-01	-0.21	4.57E-01	-0.45	1.83E-02
Phvul.009G106900	K15029 - translation initiation factor 3 subunit L (EIF3L) (1 of 1)	-0.12	6.21E-01	-0.26	9.67E-02	-0.17	4.88E-01	-0.36	2.22E-02
Phvul.011G182132	K02868 - large subunit ribosomal protein L11e (RP-L11e, RPL11) (1 of 3)	-0.12	7.64E-01	-0.31	2.12E-01	-0.09	8.47E-01	-0.55	1.87E-02
Phvul.011G024500	no data	-0.12	7.41E-01	0.43	4.25E-02	-0.24	4.84E-01	0.32	1.68E-01
Phvul.001G267200	PTHR34209:SF1 - RHODANESE/CELL CYCLE CONTROL PHOSPHATASE SUPERFAMILY PROTEIN (1 of 1)	-0.13	6.83E-01	-0.39	3.02E-02	-0.11	7.65E-01	-0.18	3.83E-01
Phvul.007G247700	K14347 - solute carrier family 10 (sodium/bile acid cotransporter), member 7 (SLC10A7, P7) (1 of 1)	-0.13	7.29E-01	-0.43	4.10E-02	-0.03	9.55E-01	-0.36	1.06E-01
Phvul.011G043300	PTHR33923:SF2 - CALMODULIN-BINDING PROTEIN-LIKE PROTEIN (1 of 3)	-0.13	7.19E-01	-0.19	4.13E-01	-0.10	8.20E-01	-0.44	3.23E-02
Phvul.003G089300	no data	-0.13	6.73E-01	-0.36	4.38E-02	-0.20	4.92E-01	-0.28	1.41E-01
Phvul.005G128300	PF11891 - Domain of unknown function (DUF3411) (DUF3411) (1 of 9)	-0.13	6.75E-01	-0.37	4.50E-02	-0.02	9.73E-01	-0.31	1.09E-01
Phvul.008G054700	PTHR12608:SF4 - GDT1-LIKE PROTEIN 3 (1 of 1)	-0.13	6.19E-01	-0.30	6.31E-02	-0.18	4.81E-01	-0.34	3.59E-02
Phvul.007G084700	2.4.2.8 - Hypoxanthine phosphoribosyltransferase / Transphosphoribosidase (1 of 1)	-0.13	7.86E-01	-0.36	1.88E-01	-0.11	8.43E-01	-0.77	3.10E-03
Phvul.004G165200	K16584 - HAUS augmin-like complex subunit 1 (HAUS1) (1 of 1)	-0.13	7.24E-01	-0.61	2.79E-03	-0.17	6.42E-01	-0.38	8.89E-02
Phvul.005G154400	PTHR10965//PTHR10965:SF3 - 60S RIBOSOMAL PROTEIN L38 // SUBFAMILY NOT NAMED (1 of 1)	-0.13	7.59E-01	-0.32	1.95E-01	-0.05	9.29E-01	-0.61	1.01E-02
Phvul.002G300600	PF10382 - Protein of unknown function (DUF2439) (DUF2439) (1 of 1)	-0.13	7.63E-01	-0.63	5.98E-03	-0.06	9.23E-01	-0.45	6.43E-02
Phvul.005G011900	PTHR31707:SF21 - PECTINESTERASE/PECTINESTERASE INHIBITOR 25-RELATED (1 of 2)	-0.13	8.81E-01	-0.98	2.27E-02	0.02	9.91E-01	-0.59	2.07E-01
Phvul.004G001300	PTHR19845:SF0 - KATANIN P80 WD40 REPEAT-CONTAINING SUBUNIT B1 (1 of 1)	-0.13	7.72E-01	-0.50	4.78E-02	-0.05	9.43E-01	-0.32	2.34E-01
Phvul.006G128600	PTHR23180:SF245 - ADP-RIBOSYLATION FACTOR GTPASE-ACTIVATING PROTEIN AGD12-RELATED (1 of 2)	-0.13	6.84E-01	-0.46	1.18E-02	-0.12	7.33E-01	-0.39	4.38E-02
Phvul.011G002200	PTHR23324:SF48 - PATELLIN-3-RELATED (1 of 3)	-0.13	7.82E-01	-0.36	1.88E-01	-0.14	7.95E-01	-0.60	2.04E-02
Phvul.002G259200	K02942 - large subunit ribosomal protein LP1 (RP-LP1, RPLP1) (1 of 4)	-0.13	7.96E-01	-0.75	3.62E-03	-0.08	9.04E-01	-0.54	5.52E-02
Phvul.007G043800	K02980 - small subunit ribosomal protein S29e (RP-S29e, RPS29) (1 of 2)	-0.13	6.41E-01	-0.17	3.84E-01	-0.07	8.58E-01	-0.36	3.51E-02
Phvul.005G043500	K05284 - phosphatidylinositol glycan, class M (PIGM) (1 of 1)	-0.13	8.81E-01	-1.28	1.63E-03	-0.29	7.23E-01	-0.93	3.24E-02
Phvul.005G104700	K02942 - large subunit ribosomal protein LP1 (RP-LP1, RPLP1) (1 of 4)	-0.13	7.09E-01	-0.33	1.09E-01	-0.13	7.49E-01	-0.45	2.91E-02
Phvul.002G075400	KOG4308 - LRR-containing protein (1 of 1)	-0.13	7.13E-01	-0.31	1.48E-01	-0.17	6.38E-01	-0.52	1.26E-02
Phvul.007G113000	PTHR19308//PTHR19308:SF12 - PHOSPHATIDYLCHOLINE TRANSFER PROTEIN // SUBFAMILY NOT NAMED (1 of 2)	-0.13	8.20E-01	-0.62	4.03E-02	0.03	9.70E-01	-0.16	6.71E-01
Phvul.008G196600	PF03754 - Domain of unknown function (DUF313) (DUF313) (1 of 6)	-0.13	7.96E-01	-0.83	1.99E-03	-0.26	5.84E-01	-0.91	9.01E-04
Phvul.003G214600	K02953 - small subunit ribosomal protein S13e (RP-S13e, RPS13) (1 of 2)	-0.13	7.29E-01	-0.33	1.48E-01	-0.05	9.23E-01	-0.57	9.45E-03
Phvul.007G163700	K02973 - small subunit ribosomal protein S23e (RP-S23e, RPS23) (1 of 4)	-0.13	7.14E-01	-0.26	2.49E-01	-0.11	7.95E-01	-0.48	2.53E-02
Phvul.006G072300	PTHR22950:SF280 - TRANSMEMBRANE AMINO ACID TRANSPORTER FAMILY PROTEIN (1 of 4)	-0.13	8.70E-01	-0.98	1.70E-02	-0.10	9.23E-01	-0.97	2.10E-02
Phvul.011G174800	PTHR23305:SF12 - GTP-BINDING PROTEIN-RELATED (1 of 1)	-0.13	7.84E-01	-0.56	3.38E-02	-0.21	6.57E-01	-0.58	3.55E-02
Phvul.003G060200	PTHR11200:SF119 - PHOSPHOINOSITIDE PHOSPHATASE SAC9-RELATED (1 of 1)	-0.13	6.58E-01	-0.36	4.29E-02	-0.10	7.84E-01	-0.23	2.42E-01
Phvul.001G026100	PTHR10795//PTHR10795:SF388 - PROPROTEIN CONVERTASE SUBTILISIN/KEXIN // SUBFAMILY NOT NAMED (1 of 5)	-0.13	8.06E-01	0.21	5.52E-01	0.13	8.35E-01	0.70	1.91E-02
Phvul.001G191200	PTHR11564//PTHR11564:SF19 - GTPASE CONTAINING FAMILY OF SIGNAL RECOGNITION PARTICLE PROTEINS // SUBFAM	-0.14	7.02E-01	-0.47	1.90E-02	-0.04	9.47E-01	-0.27	2.27E-01
Phvul.002G190400	K02917 - large subunit ribosomal protein L35Ae (RP-L35Ae, RPL35A) (1 of 2)	-0.14	7.22E-01	-0.34	1.37E-01	-0.09	8.47E-01	-0.53	1.72E-02
Phvul.011G170300	PF00197 - Trypsin and protease inhibitor (Kunitz legume) (1 of 24)	-0.14	6.75E-01	-0.39	4.33E-02	-0.06	9.02E-01	-0.23	2.71E-01
Phvul.009G071300	PTHR24093:SF262 - CALCIUM-TRANSPORTING ATPASE 2, PLASMA MEMBRANE-TYPE-RELATED (1 of 3)	-0.14	6.51E-01	0.36	4.90E-02	-0.12	7.25E-01	0.30	1.16E-01
Phvul.001G248800	4.1.1.37 - Uroporphyrinogen decarboxylase / Uroporphyrinogen-III carboxy-lyase (1 of 2)	-0.14	5.99E-01	-0.39	1.28E-02	-0.20	4.31E-01	-0.22	2.02E-01
Phvul.006G178400	K03405 - magnesium chelatase subunit I (chlI, bchI) (1 of 2)	-0.14	7.78E-01	-0.61	1.84E-02	-0.08	9.04E-01	-0.39	1.67E-01
Phvul.002G262400	PTHR22814//PTHR22814:SF126 - COPPER TRANSPORT PROTEIN ATOX1-RELATED // SUBFAMILY NOT NAMED (1 of 2)	-0.14	7.53E-01	-0.55	2.05E-02	-0.01	9.88E-01	-0.33	2.12E-01
Phvul.007G122524	PF03087 - Arabidopsis protein of unknown function (DUF241) (1 of 23)	-0.14	6.83E-01	-0.30	1.46E-01	-0.26	3.94E-01	-0.46	2.34E-02
Phvul.007G080000	no data	-0.14	8.98E-01	0.15	8.75E-01	-1.69	2.25E-02	0.35	6.53E-01
Phvul.005G057300	PTHR31568:SF17 - EXPRESSED PROTEIN (1 of 4)	-0.14	5.86E-01	-0.21	2.12E-01	-0.06	8.66E-01	-0.36	2.73E-02
Phvul.001G181000	5.3.1.1 - Triose-phosphate isomerase / Triosephosphate mutase (1 of 2)	-0.14	7.09E-01	-0.49	1.90E-02	-0.06	9.13E-01	-0.35	1.21E-01
Phvul.005G132000	PTHR19241:SF200 - ABC TRANSPORTER G FAMILY MEMBER 3 (1 of 1)	-0.14	7.31E-01	-0.42	9.90E-02	-0.36	2.94E-01	-0.50	4.87E-02
Phvul.009G049400	K02927 - large subunit ribosomal protein L40e (RP-L40e, RPL40) (1 of 2)	-0.14	7.71E-01	-0.28	3.55E-01	-0.11	8.42E-01	-0.61	2.50E-02
Phvul.003G156600	K10255 - omega-6 fatty acid desaturase (delta-12 desaturase) (FAD6, desA) (1 of 1)	-0.14	7.89E-01	-0.56	4.86E-02	-0.05	9.52E-01	-0.43	1.54E-01
Phvul.010G068219	PTHR11709:SF103 - LACCASE-16 (1 of 7)	-0.14	6.82E-01	-0.29	1.79E-01	-0.09	8.35E-01	-0.44	3.37E-02
Phvul.005G158300	K18121 - glyoxylate/succinic semialdehyde reductase (GLYR) (1 of 3)	-0.14	7.05E-01	-0.47	2.75E-02	-0.10	8.20E-01	-0.23	3.46E-01
Phvul.008G250200	4.2.1.70 - Pseudouridylate synthase / Uracil hydrolyase (1 of 3)	-0.14	7.40E-01	-0.75	3.26E-03	-0.26	5.21E-01	-0.59	2.78E-02
Phvul.008G191400	PTHR33124:SF3 - EXPRESSED PROTEIN (1 of 1)	-0.14	9.30E-01	-1.64	2.41E-02	-0.14	9.42E-01	-1.20	1.25E-01
Phvul.008G225100	PTHR31642:SF19 - COA-DEPENDENT ACYLTRANSFERASE-RELATED (1 of 2)	-0.14	7.02E-01	-0.56	6.77E-03	-0.17	6.64E-01	-0.39	8.68E-02
Phvul.006G220100	K03233 - elongation factor 1-gamma (EEF1G) (1 of 2)	-0.14	6.36E-01	-0.28	1.53E-01	-0.10	7.84E-01	-0.44	1.77E-02
Phvul.008G259600	PTHR30540:SF12 - POTASSIUM TRANSPORTER 1 (1 of 1)	-0.14	7.20E-01	-0.52	2.27E-02	-0.34	3.18E-01	-0.19	4.98E-01
Phvul.010G045500	PF01535 - PPR repeat (PPR) (1 of 425)	-0.14	4.93E-01	-0.31	2.33E-02	-0.23	2.46E-01	-0.27	5.81E-02
Phvul.007G002100	PF14617 - U3-containing 90S pre-ribosomal complex subunit (CMS1) (1 of 1)	-0.14	7.01E-01	-0.38	9.20E-02	-0.12	7.94E-01	-0.61	5.56E-03
Phvul.009G148500	PF05678 - VQ motif (VQ) (1 of 39)	-0.14	8.29E-01	1.03	7.30E-04	0.50	3.08E-01	0.96	2.73E-03
Phvul.003G130300	PTHR10795:SF434 - SUBTILISIN SERINE PROTEASE-RELATED (1 of 2)	-0.14	8.89E-01	-1.06	3.39E-02	0.03	9.85E-01	-0.54	3.31E-01
Phvul.002G153600	2.3.3.10 - Hydroxymethylglutaryl-CoA synthase / Hydroxymethylglutaryl coenzyme alpha-condensing enzyme (1 of 3)	-0.15	9.00E-01	-1.53	3.42E-03	-0.80	3.17E-01	-1.19	3.03E-02
Phvul.009G103300	PTHR12192//PTHR12192:SF10 - CATION TRANSPORT PROTEIN CHAC-RELATED // SUBFAMILY NOT NAMED (1 of 1)	-0.15	9.03E-01	-1.12	4.81E-02	-0.50	6.09E-01	-1.25	3.10E-02
Phvul.005G020200	PTHR31407:SF20 - THYLAKOID LUMENAL 19 KDA PROTEIN, CHLOROPLASTIC (1 of 1)	-0.15	7.18E-01	-0.46	4.75E-02	0.07	8.93E-01	-0.34	1.72E-01
Phvul.007G033900	PTHR18934:SF146 - DEA(D/H)-BOX RNA HELICASE FAMILY PROTEIN (1 of 2)	-0.15	6.52E-01	-0.40	4.58E-02	-0.32	2.68E-01	-0.41	4.55E-02
Phvul.011G110700	PTHR34793:SF1 - PROTEIN THYLAKOID FORMATION 1, CHLOROPLASTIC (1 of 2)	-0.15	6.11E-01	-0.48	4.71E-03	-0.14	6.69E-01	-0.36	4.93E-02
Phvul.011G067600	K11252 - histone H2B (H2B) (1 of 10)	-0.15	6.59E-01	-0.25	2.38E-01	-0.07	8.66E-01	-0.47	2.12E-02
Phvul.009G232800	no data	-0.15	8.20E-01	-0.75	2.65E-02	-0.29	6.37E-01	-0.66	6.20E-02
Phvul.009G104800	PTHR24058:SF44 - SERINE/THREONINE-PROTEIN KINASE AFC1 (1 of 1)	-0.15	5.45E-01	-0.24	1.30E-01	-0.08	7.95E-01	-0.32	4.22E-02
Phvul.003G089200	PTHR31246:SF5 - MICROTUBULE-ASSOCIATED PROTEIN 70-5 (1 of 2)	-0.15	6.21E-01	-0.25	1.93E-01	-0.12	7.31E-01	-0.41	2.80E-02
Phvul.007G103100	PTHR28570:SF3 - ASPARTYL AMINOPEPTIDASE (1 of 1)	-0.15	6.77E-01	-0.53	1.26E-02	0.02	9.74E-01	-0.37	1.03E-01
Phvul.001G189700	K03531 - cell division protein FtsZ (ftsZ) (1 of 3)	-0.15	5.87E-01	-0.37	2.62E-02	-0.12	6.98E-01	-0.41	1.89E-02
Phvul.009G038400	K03626 - nascent polypeptide-associated complex subunit alpha (EGD2, NACA) (1 of 4)	-0.15	6.03E-01	-0.31	7.98E-02	-0.07	8.55E-01	-0.38	3.44E-02
Phvul.008G227300	K02941 - large subunit ribosomal protein LP0 (RP-LP0, RPLP0) (1 of 4)	-0.15	6.80E-01	-0.29	2.03E-01	-0.10	8.23E-01	-0.47	3.10E-02
Phvul.001G078100	no data	-0.15	7.24E-01	-0.51	3.87E-02	-0.21	6.16E-01	-0.40	1.26E-01
Phvul.010G112100	K17525 - chitinase domain-containing protein 1 (CHID1) (1 of 2)	-0.15	7.43E-01	-0.76	1.81E-03	-0.06	9.25E-01	-0.48	6.96E-02
Phvul.003G173800	K02949 - small subunit ribosomal protein S11e (RP-S11e, RPS11) (1 of 4)	-0.15	6.56E-01	-0.42	3.47E-02	-0.06	9.01E-01	-0.50	1.43E-02
Phvul.006G043400	3.5.99.10 - 2-iminobutanoate/2-iminopropanoate deaminase / Enamine/imine deaminase (1 of 2)	-0.15	5.60E-01	-0.41	8.77E-03	0.00	9.94E-01	-0.39	1.87E-02
Phvul.007G189900	K02894 - large subunit ribosomal protein L23e (RP-L23e, RPL23) (1 of 2)	-0.15	7.30E-01	-0.19	5.18E-01	-0.07	8.98E-01	-0.56	2.84E-02
Phvul.009G025500	PF01190 - Pollen proteins Ole e I like (Pollen Ole e I) (1 of 29)	-0.15	8.20E-01	-0.79	2.94E-02	-0.15	8.33E-01	-0.52	1.83E-01
Phvul.009G020300	K02638 - plastocyanin (petE) (1 of 1)	-0.15	7.92E-01	-0.66	3.00E-02	0.03	9.71E-01	-0.49	1.32E-01
Phvul.011G029800	K10904 - TIMELESS-interacting protein (TIPIN) (1 of 1)	-0.15	7.69E-01	-0.70	1.07E-02	-0.02	9.78E-01	-0.54	6.58E-02
Phvul.009G112000	PTHR22950//PTHR22950:SF208 - AMINO ACID TRANSPORTER // SUBFAMILY NOT NAMED (1 of 4)	-0.15	6.56E-01	-0.54	5.60E-03	-0.18	6.23E-01	-0.50	1.41E-02
Phvul.003G211100	K02113 - F-type H+-transporting ATPase subunit delta (ATPF1D, atpH) (1 of 1)	-0.15	8.00E-01	-0.69	3.08E-02	0.09	9.08E-01	-0.41	2.43E-01
Phvul.010G021100	PTHR12701:SF13 - B-CELL RECEPTOR-ASSOCIATED PROTEIN 31-LIKE PROTEIN (1 of 2)	-0.15	7.76E-01	-0.90	4.75E-03	-0.40	3.77E-01	-0.93	4.66E-03
Phvul.007G274000	PTHR11732:SF255 - ARYL-ALCOHOL DEHYDROGENASE YPL088W-RELATED (1 of 1)	-0.15	5.93E-01	-0.45	9.07E-03	-0.23	4.04E-01	-0.23	2.24E-01
Phvul.008G133500	PTHR33101:SF9 - ROP GUANINE NUCLEOTIDE EXCHANGE FACTOR 2-RELATED (1 of 3)	-0.15	7.98E-01	-0.69	4.48E-02	-0.18	7.84E-01	-0.40	2.73E-01
Phvul.002G283400	no data	-0.15	6.49E-01	-0.46	2.32E-02	-0.32	2.86E-01	-0.39	6.34E-02
Phvul.008G255000	K02937 - large subunit ribosomal protein L7e (RP-L7e, RPL7) (1 of 3)	-0.15	6.60E-01	-0.30	1.73E-01	-0.10	8.09E-01	-0.46	2.92E-02
Phvul.L001685	no data	-0.15	8.66E-01	-0.83	7.41E-02	-0.18	8.55E-01	-1.08	2.21E-02
Phvul.011G096200	no data	-0.15	5.21E-01	-0.44	3.00E-03	-0.19	4.20E-01	-0.41	8.89E-03
Phvul.001G125400	4.4.1.8 - Cystathionine beta-lyase / Cystine lyase (1 of 1)	-0.15	7.37E-01	-0.53	4.26E-02	-0.38	3.28E-01	-0.65	1.36E-02
Phvul.008G170700	no data	-0.15	7.60E-01	-0.57	4.22E-02	-0.18	7.45E-01	-0.15	6.70E-01
Phvul.007G172400	PTHR20982:SF8 - RIBOSOME-RECYCLING FACTOR, CHLOROPLASTIC (1 of 1)	-0.15	7.04E-01	-0.64	4.50E-03	-0.11	8.28E-01	-0.53	2.71E-02
Phvul.002G246700	PTHR12357//PTHR12357:SF21 - YTH YT521-B HOMOLOGY DOMAIN-CONTAINING // SUBFAMILY NOT NAMED (1 of 1)	-0.15	7.32E-01	-0.43	1.03E-01	-0.12	8.20E-01	-0.59	2.62E-02
Phvul.004G093194	PTHR11200:SF128 - TYPE I INOSITOL 1,4,5-TRISPHOSPHATE 5-PHOSPHATASE 1 (1 of 3)	-0.15	8.97E-01	-1.29	2.11E-02	0.00	9.99E-01	-0.82	1.80E-01

Phvul.001G156200	4.1.2.8 - Indole-3-glycerol-phosphate lyase / TSA (1 of 1)	-0.16	6.79E-01	-0.61	4.27E-03	-0.09	8.42E-01	-0.31	1.98E-01
Phvul.002G217200	PTHR12064//PTHR12064:SF30 - ANCIENT CONSERVED DOMAIN PROTEIN-RELATED // SUBFAMILY NOT NAMED (1 of 2)	-0.16	7.14E-01	-0.54	2.63E-02	-0.12	8.15E-01	-0.35	1.84E-01
Phvul.007G009400	K02918 - large subunit ribosomal protein L35e (RP-L35e, RPL35) (1 of 3)	-0.16	5.41E-01	-0.26	1.23E-01	-0.13	6.50E-01	-0.40	1.60E-02
Phvul.008G284200	1.3.7.4 - Phytochromobilin:ferredoxin oxidoreductase / Phytochromobilin synthase (1 of 1)	-0.16	7.10E-01	-0.49	4.74E-02	-0.34	3.59E-01	-0.47	7.02E-02
Phvul.002G238100	PTHR12482:SF11 - HYDROLASE-LIKE PROTEIN (1 of 1)	-0.16	7.90E-01	-0.61	5.93E-02	-0.16	8.18E-01	-0.76	1.89E-02
Phvul.001G261900	PTHR12298//PTHR12298:SF7 - PCDC2 PROGRAMMED CELL DEATH PROTEIN 2 -RELATED // SUBFAMILY NOT NAMED (1	-0.16	7.00E-01	-0.59	1.04E-02	-0.03	9.65E-01	-0.49	4.46E-02
Phvul.007G111600	PTHR10774//PTHR10774:SF90 - EXTENDED SYNAPTOTAGMIN-RELATED // SUBFAMILY NOT NAMED (1 of 4)	-0.16	7.30E-01	0.51	4.39E-02	0.07	9.17E-01	0.31	2.72E-01
Phvul.002G048000	K02957 - small subunit ribosomal protein S15Ae (RP-S15Ae, RPS15A) (1 of 5)	-0.16	6.51E-01	-0.37	8.50E-02	-0.23	5.08E-01	-0.53	1.14E-02
Phvul.009G055500	PTHR16092 - SEC3/SYNTAXIN-RELATED (1 of 3)	-0.16	8.65E-01	-1.01	5.39E-02	-0.56	4.53E-01	-1.19	2.72E-02
Phvul.004G074000	PTHR11062:SF51 - EXOSTOSIN FAMILY PROTEIN (1 of 1)	-0.16	5.73E-01	-0.36	4.04E-02	-0.26	3.24E-01	-0.27	1.51E-01
Phvul.003G230200	PTHR19370:SF109 - FERREDOXIN C2 (1 of 1)	-0.16	5.14E-01	-0.37	1.48E-02	-0.03	9.37E-01	-0.20	2.47E-01
Phvul.008G057300	PTHR10994:SF70 - RETICULON-LIKE PROTEIN B4 (1 of 3)	-0.16	5.94E-01	-0.30	1.16E-01	-0.12	7.23E-01	-0.44	1.88E-02
Phvul.007G033100	no data	-0.16	8.06E-01	-0.99	2.94E-03	-0.30	6.20E-01	-0.69	5.47E-02
Phvul.010G133500	PTHR14194//PTHR14194:SF44 - NITROGEN METABOLIC REGULATION PROTEIN NMR-RELATED // SUBFAMILY NOT NAME	-0.16	6.60E-01	-0.43	4.37E-02	-0.13	7.58E-01	-0.28	2.32E-01
Phvul.002G234700	1.3.3.8 - Tetrahydroberberine oxidase / THB oxidase (1 of 37)	-0.16	8.06E-01	0.47	1.96E-01	0.15	8.33E-01	0.75	2.91E-02
Phvul.003G208300	PTHR10566:SF72 - PROTEIN KINASE FAMILY PROTEIN (1 of 1)	-0.16	7.68E-01	-0.85	3.08E-03	-0.20	7.19E-01	-0.74	1.46E-02
Phvul.L001643	K02918 - large subunit ribosomal protein L35e (RP-L35e, RPL35) (1 of 3)	-0.16	6.34E-01	-0.32	1.34E-01	-0.16	6.68E-01	-0.55	7.18E-03
Phvul.002G254000	PF04782 - Protein of unknown function (DUF632) (DUF632) (1 of 15)	-0.16	6.11E-01	-0.21	3.29E-01	-0.13	7.20E-01	-0.40	4.25E-02
Phvul.001G019400	PTHR23054:SF29 - EXPRESSED PROTEIN (1 of 2)	-0.16	7.94E-01	-0.68	4.00E-02	-0.18	7.84E-01	-0.75	2.75E-02
Phvul.003G277700	PTHR14155:SF153 - RING/U-BOX DOMAIN-CONTAINING PROTEIN (1 of 2)	-0.16	7.66E-01	-0.62	4.37E-02	-0.25	6.34E-01	-0.50	1.21E-01
Phvul.002G249700	PF00190 - Cupin (Cupin 1) (1 of 22)	-0.16	8.43E-01	-0.17	7.66E-01	-0.66	2.72E-01	-0.92	4.06E-02
Phvul.009G171900	PTHR12231:SF150 - RHODANESE-LIKE DOMAIN-CONTAINING PROTEIN 11, CHLOROPLASTIC (1 of 1)	-0.16	6.24E-01	-0.57	2.77E-03	-0.21	5.28E-01	-0.45	2.72E-02
Phvul.001G136400	6.3.4.4 - Adenylosuccinate synthase / Succinoadenylic kinosynthetase (1 of 1)	-0.16	6.83E-01	-0.27	2.82E-01	-0.15	7.31E-01	-0.50	3.59E-02
Phvul.010G049401	3.1.1.74 - Cutinase / Cutin hydrolase (1 of 1)	-0.16	7.52E-01	-0.42	1.61E-01	-0.28	5.63E-01	-0.60	4.26E-02
Phvul.001G250200	PTHR13683:SF303 - ASPARTIC PROTEINASE-LIKE PROTEIN 1 (1 of 1)	-0.16	6.63E-01	-0.46	3.97E-02	-0.01	9.86E-01	-0.45	4.90E-02
Phvul.004G034800	K02966 - small subunit ribosomal protein S19e (RP-S19e, RPS19) (1 of 3)	-0.16	6.51E-01	-0.36	9.59E-02	-0.15	7.10E-01	-0.55	1.10E-02
Phvul.008G019800	K02908 - large subunit ribosomal protein L30e (RP-L30e, RPL30) (1 of 2)	-0.16	6.24E-01	-0.25	2.39E-01	-0.16	6.52E-01	-0.42	3.79E-02
Phvul.001G003500	PTHR13516//PTHR13516:SF9 - RIBONUCLEASE P SUBUNIT P25 // SUBFAMILY NOT NAMED (1 of 2)	-0.16	4.57E-01	-0.28	5.65E-02	-0.11	6.76E-01	-0.31	3.50E-02
Phvul.003G156500	PTHR14499//PTHR14499:SF70 - POTASSIUM CHANNEL TETRAMERIZATION DOMAIN-CONTAINING // SUBFAMILY NOT N	-0.16	7.61E-01	0.58	5.92E-02	-0.13	8.28E-01	0.66	2.95E-02
Phvul.007G250100	K03250 - translation initiation factor 3 subunit E (EIF3E, INT6) (1 of 1)	-0.16	4.63E-01	-0.24	1.12E-01	-0.10	7.09E-01	-0.30	4.46E-02
Phvul.004G159000	K03456 - serine/threonine-protein phosphatase 2A regulatory subunit A (PPP2R1) (1 of 3)	-0.16	4.22E-01	-0.29	3.81E-02	-0.15	4.81E-01	-0.20	1.70E-01
Phvul.010G113500	PTHR14068 - EUKARYOTIC TRANSLATION INITIATION FACTOR 3 EIF3 -RELATED (1 of 3)	-0.16	9.24E-01	-1.62	2.90E-02	-0.48	7.32E-01	-1.16	1.41E-01
Phvul.002G120200	PF00373 - FERM central domain (FERM M) (1 of 2)	-0.16	5.82E-01	-0.38	3.87E-02	-0.26	3.54E-01	-0.39	3.74E-02
Phvul.004G143300	PF00076 - RNA recognition motif. (a.k.a. RRM, RBD, or RNP domain) (RRM 1) (1 of 252)	-0.16	5.67E-01	-0.45	9.25E-03	-0.15	6.48E-01	-0.36	5.47E-02
Phvul.004G149800	PTHR10458:SF2 - PEPTIDE DEFORMYLASE 1B, CHLOROPLASTIC/MITOCHONDRIAL (1 of 1)	-0.16	4.08E-01	-0.29	3.14E-02	-0.11	6.32E-01	-0.30	3.21E-02
Phvul.007G174300	PTHR10209:SF170 - 2-OXOGLUTARATE (2OG) AND FE(II)-DEPENDENT OXYGENASE SUPERFAMILY PROTEIN (1 of 1)	-0.16	6.36E-01	-0.23	3.61E-01	-0.23	5.09E-01	-0.48	3.23E-02
Phvul.002G297900	no data	-0.16	7.41E-01	-0.72	8.24E-03	-0.14	8.07E-01	-0.82	3.34E-03
Phvul.007G166500	PTHR22939:SF83 - PROTEASE DO-LIKE 2, CHLOROPLASTIC (1 of 1)	-0.16	5.68E-01	-0.45	1.19E-02	-0.10	7.95E-01	-0.18	3.75E-01
Phvul.007G026300	K00627 - pyruvate dehydrogenase E2 component (dihydrolipoamide acetyltransferase) (DLAT, aceF, pdhC) (1 of 4)	-0.16	7.10E-01	-0.62	1.24E-02	-0.18	7.12E-01	-0.73	4.42E-03
Phvul.007G177400	PTHR32116:SF0 - GALACTURONOSYLTRANSFERASE 5-RELATED (1 of 2)	-0.16	6.53E-01	-0.54	1.34E-02	-0.26	4.65E-01	-0.49	3.28E-02
Phvul.001G124500	PTHR11106//PTHR11106:SF64 - GANGLIOSIDE INDUCED DIFFERENTIATION ASSOCIATED PROTEIN 2-RELATED // SUBFAM	-0.17	7.30E-01	0.47	9.39E-02	0.15	7.85E-01	0.55	4.92E-02
Phvul.002G280800	PTHR17130//PTHR17130:SF25 - MITOCHONDRIAL OUTER MEMBRANE PROTEIN 25 // SUBFAMILY NOT NAMED (1 of 2)	-0.17	8.48E-01	-1.06	1.42E-02	0.12	9.09E-01	-0.85	6.07E-02
Phvul.004G176100	PTHR10629:SF28 - DNA (CYTOSINE-5)-METHYLTRANSFERASE CMT3 (1 of 1)	-0.17	7.94E-01	-0.24	5.97E-01	-0.62	2.11E-01	-0.80	3.37E-02
Phvul.009G064600	PTHR33926:SF2 - PROTEIN TIC 22, CHLOROPLASTIC (1 of 1)	-0.17	6.58E-01	-0.56	1.08E-02	-0.17	6.82E-01	-0.56	1.35E-02
Phvul.007G263400	K12501 - homogentisate solanesyltransferase (HST) (1 of 1)	-0.17	5.62E-01	-0.47	7.51E-03	-0.28	3.03E-01	-0.47	1.13E-02
Phvul.005G081500	PTHR24067:SF100 - UBIQUITIN-CONJUGATING ENZYME FAMILY PROTEIN-RELATED (1 of 2)	-0.17	7.08E-01	-0.65	9.38E-03	-0.22	6.28E-01	-0.31	2.79E-01
Phvul.005G154000	PTHR23189:SF45 - FLOWERING TIME CONTROL PROTEIN FPA (1 of 2)	-0.17	5.99E-01	-0.40	4.65E-02	-0.25	4.10E-01	-0.28	1.95E-01
Phvul.001G114100	2.4.1.25 - 4-alpha-glucanotransferase / Oligo-1,4-1,4-glucantransferase (1 of 3)	-0.17	6.08E-01	-0.41	4.13E-02	0.02	9.73E-01	-0.16	5.08E-01
Phvul.007G147100	PTHR11455:SF2 - BLUE-LIGHT PHOTORECEPTOR PHR2 (1 of 1)	-0.17	6.04E-01	-0.40	4.40E-02	0.01	9.85E-01	-0.20	3.65E-01
Phvul.009G147600	K01956 - carbamoyl-phosphate synthase small subunit (carA, CPA1) (1 of 1)	-0.17	5.20E-01	-0.41	1.12E-02	-0.18	5.18E-01	-0.36	3.43E-02
Phvul.007G090400	PF13424 - Tetratricopeptide repeat (TPR 12) (1 of 22)	-0.17	4.18E-01	-0.32	2.20E-02	-0.17	4.33E-01	-0.27	7.15E-02
Phvul.004G065100	no data	-0.17	6.48E-01	-0.43	5.48E-02	-0.08	8.61E-01	-0.54	1.60E-02
Phvul.001G202600	K14485 - transport inhibitor response 1 (TIR1) (1 of 3)	-0.17	7.29E-01	-0.58	3.39E-02	-0.05	9.43E-01	-0.41	1.67E-01
Phvul.L001631	K02694 - photosystem I subunit III (psaF) (1 of 1)	-0.17	7.01E-01	-0.50	4.83E-02	0.10	8.52E-01	-0.17	5.90E-01
Phvul.003G186700	PTHR31434:SF2 - S PHASE CYCLIN A-ASSOCIATED PROTEIN IN THE ENDOPLASMIC RETICULUM (1 of 1)	-0.17	3.60E-01	-0.03	8.59E-01	-0.32	3.78E-02	-0.05	7.90E-01
Phvul.005G050700	PTHR22904//PTHR22904:SF10 - TPR REPEAT CONTAINING PROTEIN // SUBFAMILY NOT NAMED (1 of 1)	-0.17	6.35E-01	-0.48	2.33E-02	0.01	9.91E-01	-0.29	2.15E-01
Phvul.008G012900	no data	-0.17	8.23E-01	-0.92	1.96E-02	-0.38	5.82E-01	-0.92	2.65E-02
Phvul.002G122600	PTHR10668:SF80 - LONG-CHAIN-ALCOHOL OXIDASE FAO4A (1 of 2)	-0.17	8.51E-01	1.04	1.86E-02	-0.40	6.11E-01	0.63	1.98E-01
Phvul.010G110600	PTHR23416:SF2 - SERINE ACETYLTRANSFERASE 5 (1 of 2)	-0.17	7.00E-01	-0.34	2.22E-01	0.00	9.98E-01	-0.53	4.23E-02
Phvul.001G191700	K02927 - large subunit ribosomal protein L40e (RP-L40e, RPL40) (1 of 2)	-0.17	6.46E-01	-0.30	2.06E-01	-0.06	9.07E-01	-0.48	3.68E-02
Phvul.003G018300	K02981 - small subunit ribosomal protein S2e (RP-S2e, RPS2) (1 of 2)	-0.17	6.91E-01	-0.33	2.28E-01	-0.03	9.64E-01	-0.55	3.05E-02
Phvul.005G107100	K14829 - pre-rRNA-processing protein IPI3 (IPI3) (1 of 4)	-0.17	7.21E-01	-0.57	3.44E-02	-0.33	4.57E-01	-0.73	7.64E-03
Phvul.009G097300	PTHR33077:SF8 - PROTEIN TIFY 8 (1 of 2)	-0.17	5.15E-01	-0.35	4.05E-02	-0.20	4.47E-01	-0.39	2.76E-02
Phvul.010G044100	K02921 - large subunit ribosomal protein L37Ae (RP-L37Ae, RPL37A) (1 of 2)	-0.17	6.53E-01	-0.35	1.45E-01	-0.15	7.48E-01	-0.60	8.95E-03
Phvul.009G045800	K02942 - large subunit ribosomal protein LP1 (RP-LP1, RPLP1) (1 of 4)	-0.17	5.70E-01	-0.25	2.19E-01	-0.09	8.30E-01	-0.47	1.55E-02
Phvul.007G094500	no data	-0.17	8.26E-01	-0.99	2.37E-02	-0.09	9.30E-01	-0.74	1.05E-01
Phvul.003G148000	2.7.1.16 - Ribulokinase / L-ribulokinase (1 of 1)	-0.17	4.26E-01	-0.34	1.96E-02	-0.18	4.48E-01	-0.21	1.89E-01
Phvul.007G138600	3.6.1.17 - Bis(5'-nucleosyl)-tetraphosphatase (asymmetrical) / Dinucleosidetetraphosphatase (asymmetrical) (1 of 2)	-0.17	5.41E-01	-0.53	2.66E-03	-0.22	4.55E-01	-0.45	1.53E-02
Phvul.009G057000	no data	-0.17	8.36E-01	-0.46	3.85E-01	-0.56	3.98E-01	-1.23	1.11E-02
Phvul.006G150200	no data	-0.17	6.62E-01	-0.47	5.39E-02	-0.28	4.74E-01	-0.61	1.25E-02
Phvul.011G149100	PTHR14221:SF0 - WD REPEAT-CONTAINING PROTEIN 44 (1 of 2)	-0.17	7.17E-01	-0.74	7.78E-03	-0.25	5.98E-01	-0.64	2.83E-02
Phvul.008G229700	4.3.2.2 - Adenylosuccinate lyase / Succino AMP-lyase (1 of 1)	-0.18	5.50E-01	-0.38	4.25E-02	-0.14	6.80E-01	-0.31	1.13E-01
Phvul.004G035000	K02160 - acetyl-CoA carboxylase biotin carboxyl carrier protein (accB, bccP) (1 of 3)	-0.18	5.43E-01	-0.47	1.13E-02	-0.19	5.46E-01	-0.33	9.08E-02
Phvul.011G159000	K00026 - malate dehydrogenase (MDH2) (1 of 3)	-0.18	6.01E-01	-0.42	4.42E-02	-0.10	8.10E-01	-0.57	6.51E-03
Phvul.005G083700	1.3.1.33 - Protochlorophyllide reductase / Protochlorophyllide oxidoreductase (1 of 2)	-0.18	7.94E-01	-0.89	1.37E-02	-0.13	8.79E-01	-0.79	3.58E-02
Phvul.004G069100	PF14368 - Probable lipid transfer (LTP 2) (1 of 66)	-0.18	5.08E-01	-0.23	2.17E-01	-0.17	5.59E-01	-0.43	1.27E-02
Phvul.002G259300	PTHR32091:SF0 - EUKARYOTIC INITIATION FACTOR 4B-RELATED (1 of 1)	-0.18	5.11E-01	-0.29	1.68E-01	-0.02	9.66E-01	-0.51	1.12E-02
Phvul.011G074900	PTHR31384:SF20 - AUXIN RESPONSE FACTOR 4 (1 of 2)	-0.18	8.52E-01	-1.50	5.56E-03	-0.40	6.42E-01	-1.00	7.21E-02
Phvul.004G001500	PTHR10949//PTHR10949:SF11 - LIPOYL SYNTHASE // SUBFAMILY NOT NAMED (1 of 1)	-0.18	4.65E-01	-0.23	1.82E-01	-0.10	7.51E-01	-0.47	4.30E-03
Phvul.002G217300	PTHR32285:SF12 - PROTEIN TRICHOME BIREFRINGENCE-LIKE 13 (1 of 1)	-0.18	5.86E-01	-0.47	1.79E-02	-0.03	9.55E-01	-0.19	4.10E-01
Phvul.002G292500	PTHR10641//PTHR10641:SF667 - MYB-LIKE DNA-BINDING PROTEIN MYB // SUBFAMILY NOT NAMED (1 of 2)	-0.18	5.97E-01	-0.34	1.10E-01	0.00	9.98E-01	-0.56	6.91E-03
Phvul.008G240600	K00963 - UTP--glucose-1-phosphate uridylyltransferase (UGP2, galU, galF) (1 of 2)	-0.18	4.63E-01	-0.39	1.39E-02	-0.21	4.16E-01	-0.44	7.18E-03
Phvul.002G110000	K02938 - large subunit ribosomal protein L8e (RP-L8e, RPL8) (1 of 4)	-0.18	5.86E-01	-0.21	3.70E-01	0.03	9.55E-01	-0.43	4.40E-02
Phvul.009G181000	PTHR11119:SF48 - NUCLEOBASE-ASCORBATE TRANSPORTER 5-RELATED (1 of 2)	-0.18	5.60E-01	-0.43	2.44E-02	-0.17	6.15E-01	-0.18	4.37E-01
Phvul.002G320200	PTHR24067//PTHR24067:SF147 - UBIQUITIN-CONJUGATING ENZYME E2 // SUBFAMILY NOT NAMED (1 of 1)	-0.18	5.57E-01	-0.18	4.26E-01	-0.11	7.93E-01	-0.41	3.92E-02
Phvul.001G003300	PTHR24349:SF89 - CALCIUM-DEPENDENT PROTEIN KINASE 29 (1 of 5)	-0.18	8.84E-01	-0.79	2.65E-01	-0.24	8.58E-01	-1.55	2.92E-02
Phvul.002G321500	PTHR14155//PTHR14155:SF73 - RING FINGER DOMAIN-CONTAINING // SUBFAMILY NOT NAMED (1 of 4)	-0.18	6.39E-01	-0.49	3.28E-02	-0.21	6.17E-01	-0.44	7.31E-02
Phvul.008G292300	K00872 - homoserine kinase (thrB1) (1 of 1)	-0.18	5.30E-01	-0.26	1.93E-01	-0.12	7.33E-01	-0.41	3.76E-02
Phvul.007G073200	PTHR33162:SF3 - SEC-INDEPENDENT PROTEIN TRANSLOCASE PROTEIN TATB, CHLOROPLASTIC (1 of 1)	-0.18	6.51E-01	-0.58	1.46E-02	-0.13	7.95E-01	-0.60	1.48E-02
Phvul.002G127900	K02873 - large subunit ribosomal protein L13e (RP-L13e, RPL13) (1 of 3)	-0.18	5.94E-01	-0.35	1.04E-01	-0.13	7.54E-01	-0.47	2.93E-02
Phvul.004G063700	K02908 - large subunit ribosomal protein L30e (RP-L30e, RPL30) (1 of 2)	-0.18	6.07E-01	-0.37	1.04E-01	-0.20	5.95E-01	-0.45	4.43E-02
Phvul.008G255600	PTHR13343:SF14 - GENOMIC DNA, CHROMOSOME 3, P1 CLONE: MXL8 (1 of 1)	-0.18	5.78E-01	-0.41	4.67E-02	-0.17	6.41E-01	-0.36	9.51E-02
Phvul.005G178800	PTHR24078:SF307 - MOLECULAR CHAPERONE HSP40/DNAJ-LIKE PROTEIN (1 of 1)	-0.18	5.39E-01	-0.44	1.74E-02	-0.04	9.34E-01	-0.31	1.29E-01
Phvul.008G221300	PTHR31388:SF24 - PEROXIDASE 52 (1 of 2)	-0.18	6.55E-01	-0.22	4.46E-01	-0.20	6.45E-01	-0.59	1.84E-02
Phvul.008G044300	PTHR19297//PTHR19297:SF114 - GLYCOSYLTRANSFERASE 14 FAMILY MEMBER // SUBFAMILY NOT NAMED (1 of 2)	-0.18	6.96E-01	-0.64	1.68E-02	-0.09	8.83E-01	-0.65	1.99E-02
Phvul.002G317200	PTHR10641//PTHR10641:SF510 - MYB-LIKE DNA-BINDING PROTEIN MYB // SUBFAMILY NOT NAMED (1 of 1)	-0.18	6.28E-01	-0.41	7.80E-02	-0.10	8.35E-01	-0.56	1.62E-02
Phvul.008G096500	K04040 - chlorophyll synthase (chlG, bchG) (1 of 1)	-0.18	4.38E-01	-0.38	1.55E-02	-0.24	3.08E-01	-0.38	2.00E-02
Phvul.011G093000	2.4.99.4 - Beta-galactoside alpha-2,3-sialyltransferase / CMP-N-acetylneuraminate-beta-galactosamide-alpha-2,3-sialyltr	-0.18	6.29E-01	-0.47	4.72E-02	-0.14	7.55E-01	-0.55	2.14E-02
Phvul.001G005500	PTHR10774:SF50 - SYNAPTOTAGMIN-1-RELATED (1 of 3)	-0.18	5.35E-01	-0.41	3.16E-02	-0.26	3.68E-01	-0.32	1.12E-01
Phvul.002G275900	PF01535//PF13041 - PPR repeat (PPR) // PPR repeat family (PPR 2) (1 of 197)	-0.18	5.11E-01	-0.43	1.80E-02	-0.08	8.33E-01	-0.24	2.20E-01
Phvul.003G085000	PTHR23315:SF129 - ARMADILLO/BETA-CATENIN-LIKE REPEAT-CONTAINING PROTEIN (1 of 2)	-0.19	5.65E-01	0.44	2.72E-02	-0.03	9.55E-01	0.47	2.16E-02
Phvul.004G064800	K01602 - ribulose-bisphosphate carboxylase small chain (rbcS) (1 of 3)	-0.19	7.15E-01	-0.50	1.01E-01	-0.19	7.33E-01	-0.72	1.52E-02
Phvul.006G073300	K18693 - diacylglycerol diphosphate phosphatase / phosphatidate phosphatase (DPP1) (1 of 1)	-0.19	5.78E-01	-0.51	1.37E-02	-0.13	7.54E-01	-0.45	4.02E-02

Phvul.002G210750	K02548 - 1,4-dihydroxy-2-naphthoate octaprenyltransferase (menA) (1 of 1)	-0.19	7.47E-01	-0.82	9.36E-03	-0.30	5.95E-01	-0.73	2.79E-02
Phvul.002G044200	K02984 - small subunit ribosomal protein S3Ae (RP-S3Ae, RPS3A) (1 of 3)	-0.19	6.29E-01	-0.32	1.97E-01	-0.12	8.13E-01	-0.53	2.70E-02
Phvul.009G165800	no data	-0.19	7.74E-01	-0.87	1.40E-02	-0.19	7.92E-01	-0.74	4.76E-02
Phvul.006G130700	PTHR31707:SF41 - GENOMIC DNA, CHROMOSOME 3, TAC CLONE:K14A17 (1 of 2)	-0.19	5.62E-01	-0.56	4.40E-03	-0.19	5.80E-01	-0.56	6.82E-03
Phvul.002G064000	PF01657//PF07714 - Salt stress response/antifungal (Stress-antifung) // Protein tyrosine kinase (Pkinase Tyr) (1 of 40)	-0.19	6.48E-01	-0.66	7.12E-03	-0.25	5.50E-01	-0.54	3.65E-02
Phvul.002G006000	PTHR11715:SF1 - GLYCINE CLEAVAGE SYSTEM H PROTEIN 3, MITOCHONDRIAL (1 of 1)	-0.19	7.17E-01	-0.67	2.23E-02	-0.04	9.64E-01	-0.37	2.62E-01
Phvul.006G143500	3.7.1.2 - Fumarylacetoacetase / Fumarylacetoacetate hydrolase (1 of 1)	-0.19	4.39E-01	-0.33	4.87E-02	-0.07	8.33E-01	-0.31	7.47E-02
Phvul.002G207800	3.1.4.57 - Phosphoribosyl 1,2-cyclic phosphate 1,2-diphosphodiesterase / Cyclic phosphate dihydrolase (1 of 1)	-0.19	5.77E-01	-0.51	1.60E-02	-0.29	3.83E-01	-0.27	2.54E-01
Phvul.008G125900	PTHR27002:SF84 - ATP BINDING / PROTEIN KINASE-RELATED (1 of 5)	-0.19	5.99E-01	-0.27	2.78E-01	-0.10	8.28E-01	-0.55	1.46E-02
Phvul.003G182000	PF03909 - BSD domain (BSD) (1 of 10)	-0.19	7.17E-01	-0.67	2.33E-02	-0.26	6.32E-01	-0.66	3.37E-02
Phvul.007G027600	PTHR12934:SF11 - 50S RIBOSOMAL PROTEIN L15, CHLOROPLASTIC (1 of 1)	-0.19	6.18E-01	-0.63	4.99E-03	-0.05	9.29E-01	-0.45	6.21E-02
Phvul.002G318400	PF07714//PF14380 - Protein tyrosine kinase (Pkinase Tyr) // Wall-associated receptor kinase C-terminal (WAK assoc) (1	-0.19	5.99E-01	-0.52	1.57E-02	-0.21	5.73E-01	-0.47	4.08E-02
Phvul.005G074200	PTHR11886//PTHR11886:SF49 - DYNEIN LIGHT CHAIN // SUBFAMILY NOT NAMED (1 of 1)	-0.19	9.10E-01	-2.23	4.19E-03	-0.06	9.79E-01	-1.78	3.04E-02
Phvul.008G231400	PF03080 - Domain of unknown function (DUF239) (DUF239) (1 of 20)	-0.19	3.25E-01	-0.04	8.47E-01	-0.36	3.02E-02	-0.12	4.50E-01
Phvul.010G007600	PTHR11132//PTHR11132:SF104 - SOLUTE CARRIER FAMILY 35 // SUBFAMILY NOT NAMED (1 of 4)	-0.19	6.39E-01	-0.62	1.42E-02	-0.18	6.93E-01	-0.50	5.89E-02
Phvul.010G007300	PTHR31741:SF4 - O-FUCOSYLTRANSFERASE FAMILY PROTEIN (1 of 1)	-0.19	6.55E-01	-0.68	7.37E-03	-0.14	7.97E-01	-0.59	2.72E-02
Phvul.011G128100	PTHR24006:SF466 - F-BOX/LRR-REPEAT PROTEIN 18 (1 of 1)	-0.19	6.84E-01	-0.70	8.77E-03	-0.25	6.10E-01	-0.71	1.13E-02
Phvul.006G125200	PTHR32448:SF25 - FAD-BINDING BERBERINE FAMILY PROTEIN-RELATED (1 of 5)	-0.19	6.70E-01	-0.52	6.21E-02	-0.20	6.96E-01	-0.76	5.98E-03
Phvul.003G166300	PTHR35742:SF1 - THYLAKOID LUMENAL 16.5 KDA PROTEIN, CHLOROPLASTIC (1 of 1)	-0.19	5.23E-01	-0.55	3.23E-03	-0.16	6.43E-01	-0.51	8.78E-03
Phvul.004G038900	PTHR13844:SF12 - EXPRESSED PROTEIN-RELATED (1 of 1)	-0.19	4.48E-01	-0.47	4.74E-03	-0.10	7.69E-01	-0.40	2.45E-02
Phvul.002G194300	2.3.1.41 - Beta-ketoacyl-[acyl-carrier-protein] synthase I / KAS I (1 of 2)	-0.19	7.16E-01	-0.80	8.16E-03	-0.18	7.79E-01	-0.94	2.25E-03
Phvul.007G197900	K02883 - large subunit ribosomal protein L18e (RP-L18e, RPL18) (1 of 4)	-0.19	5.25E-01	-0.34	9.93E-02	-0.15	6.84E-01	-0.45	2.44E-02
Phvul.010G148600	PTHR33222:SF3 - PROTEIN CURVATURE THYLAKOID 1C, CHLOROPLASTIC (1 of 1)	-0.20	5.46E-01	-0.76	8.42E-05	-0.21	5.31E-01	-0.75	1.30E-04
Phvul.004G171400	K19033 - RPS31; 30S ribosomal protein S31 (PSRP4) (1 of 3)	-0.20	6.76E-01	-0.56	4.11E-02	-0.08	8.95E-01	-0.33	2.72E-01
Phvul.008G005100	K19032 - 30S ribosomal protein 3 (PSRP3) (1 of 1)	-0.20	5.50E-01	-0.47	2.23E-02	-0.10	8.10E-01	-0.35	1.12E-01
Phvul.002G057000	K02877 - large subunit ribosomal protein L15e (RP-L15e, RPL15) (1 of 2)	-0.20	6.51E-01	-0.36	2.00E-01	-0.08	8.87E-01	-0.54	4.20E-02
Phvul.009G241700	K02901 - large subunit ribosomal protein L27e (RP-L27e, RPL27) (1 of 2)	-0.20	5.99E-01	-0.42	7.02E-02	-0.07	8.94E-01	-0.60	9.18E-03
Phvul.003G215501	K14423 - 4,4-dimethyl-9beta,19-cyclopropylsterol-4alpha-methyl oxidase (SMO1) (1 of 2)	-0.20	6.77E-01	-0.59	3.48E-02	-0.11	8.55E-01	-0.63	2.65E-02
Phvul.011G091000	3.1.3.2//3.1.3.48 - Acid phosphatase / Phosphomonoesterase // Protein-tyrosine-phosphatase / PTPase (1 of 1)	-0.20	5.02E-01	-0.55	2.68E-03	-0.18	5.76E-01	-0.48	1.33E-02
Phvul.011G051700	no data	-0.20	4.28E-01	-0.48	3.15E-03	-0.07	8.38E-01	-0.26	1.52E-01
Phvul.003G147900	2.6.1.62 - Adenosylmethionine--8-amino-7-oxononanoate transaminase / DAPA aminotransferase (1 of 1)	-0.20	5.80E-01	-0.33	1.96E-01	-0.11	8.16E-01	-0.50	4.50E-02
Phvul.001G244200	K02865 - large subunit ribosomal protein L10Ae (RP-L10Ae, RPL10A) (1 of 2)	-0.20	4.16E-01	-0.27	1.10E-01	-0.19	4.90E-01	-0.44	7.53E-03
Phvul.002G163100	K14684 - solute carrier family 25 (mitochondrial phosphate transporter), member 23/24/25/41 (SLC25A23S) (1 of 14)	-0.20	7.70E-01	-1.11	1.09E-02	-0.19	8.08E-01	-0.21	6.70E-01
Phvul.008G236900	PF00069//PF08263 - Protein kinase domain (Pkinase) // Leucine rich repeat N-terminal domain (LRRNT 2) (1 of 19)	-0.20	4.85E-01	-0.46	1.17E-02	-0.08	8.47E-01	-0.30	1.41E-01
Phvul.003G102600	PTHR13832:SF267 - PROTEIN PHOSPHATASE 1K, MITOCHONDRIAL (1 of 2)	-0.20	6.33E-01	-0.61	1.38E-02	-0.26	5.31E-01	-0.66	1.04E-02
Phvul.004G093200	no data	-0.20	8.95E-01	-1.71	1.55E-02	-0.17	9.25E-01	-1.32	8.11E-02
Phvul.004G158300	PF00139 - Legume lectin domain (Lectin legB) (1 of 53)	-0.20	8.43E-01	-1.00	5.36E-02	0.09	9.47E-01	-1.09	3.74E-02
Phvul.006G198700	1.8.4.12 - Peptide-methionine (R)-S-oxide reductase / Selenoprotein R (1 of 4)	-0.20	5.04E-01	-0.56	2.68E-03	-0.03	9.48E-01	-0.37	6.76E-02
Phvul.011G048000	PF10950 - Protein of unknown function (DUF2775) (DUF2775) (1 of 4)	-0.20	6.09E-01	-0.47	4.94E-02	-0.14	7.80E-01	-0.27	3.01E-01
Phvul.005G085300	PTHR10108:SF754 - EXPRESSED PROTEIN (1 of 2)	-0.20	7.61E-01	-0.76	3.99E-02	-0.14	8.58E-01	-0.49	2.19E-01
Phvul.002G157300	PTHR31500:SF9 - AT HOOK MOTIF DNA-BINDING FAMILY PROTEIN (1 of 2)	-0.20	3.81E-01	-0.22	2.07E-01	-0.13	6.58E-01	-0.45	4.84E-03
Phvul.008G223300	PTHR10799:SF660 - SNF2 DOMAIN-CONTAINING PROTEIN CLASSY 1-RELATED (1 of 2)	-0.20	5.02E-01	-0.27	1.99E-01	-0.09	8.35E-01	-0.43	3.23E-02
Phvul.002G104200	PTHR18896:SF60 - PHOSPHOLIPASE D DELTA (1 of 1)	-0.20	4.52E-01	-0.37	4.11E-02	-0.13	7.09E-01	-0.21	2.92E-01
Phvul.011G193100	PTHR23155//PTHR23155:SF414 - LEUCINE-RICH REPEAT-CONTAINING PROTEIN // SUBFAMILY NOT NAMED (1 of 124)	-0.20	5.50E-01	-0.39	7.92E-02	-0.27	4.25E-01	-0.44	4.87E-02
Phvul.009G070500	PTHR10795//PTHR10795:SF388 - PROPROTEIN CONVERTASE SUBTILISIN/KEXIN // SUBFAMILY NOT NAMED (1 of 5)	-0.20	7.75E-01	-0.66	9.52E-02	-0.14	8.69E-01	-0.82	3.90E-02
Phvul.001G179200	6.1.1.21 - Histidine--tRNA ligase / Histidyl-tRNA synthetase (1 of 2)	-0.20	5.74E-01	-0.48	3.39E-02	0.05	9.29E-01	-0.14	6.03E-01
Phvul.010G160500	K02951 - small subunit ribosomal protein S12e (RP-S12e, RPS12) (1 of 3)	-0.20	5.60E-01	-0.29	2.27E-01	-0.20	5.88E-01	-0.55	1.24E-02
Phvul.009G194900	PTHR31807:SF2 - HAUS AUGMIN-LIKE COMPLEX SUBUNIT 8 (1 of 2)	-0.20	7.42E-01	-1.25	2.44E-04	-0.29	6.48E-01	-0.75	4.00E-02
Phvul.008G286500	K14411 - RNA-binding protein Musashi (MSI) (1 of 6)	-0.20	4.11E-01	-0.25	1.64E-01	-0.24	3.47E-01	-0.44	1.08E-02
Phvul.007G203300	KOG3195 - Uncharacterized membrane protein NPD008/CGI-148 (1 of 2)	-0.20	3.92E-01	-0.18	3.31E-01	-0.22	3.86E-01	-0.34	4.90E-02
Phvul.009G082300	K02221 - YggT family protein (yggT) (1 of 2)	-0.20	7.46E-01	-0.76	2.89E-02	-0.08	9.31E-01	-0.70	5.46E-02
Phvul.009G127100	4.2.1.24 - Porphobilinogen synthase / Delta-aminolevulinic acid dehydratase (1 of 1)	-0.20	6.58E-01	-0.66	1.35E-02	-0.16	7.65E-01	-0.56	4.71E-02
Phvul.002G295400	PTHR11122//PTHR11122:SF25 - APOSPORY-ASSOCIATED PROTEIN C-RELATED // SUBFAMILY NOT NAMED (1 of 2)	-0.20	5.38E-01	-0.40	6.02E-02	-0.20	5.85E-01	-0.43	4.85E-02
Phvul.005G072000	PTHR13902:SF3 - GB (1 of 2)	-0.20	6.33E-01	-0.74	4.48E-03	-0.27	5.22E-01	-0.69	1.17E-02
Phvul.006G111411	no data	-0.20	5.61E-01	-0.44	4.88E-02	-0.29	4.08E-01	-0.42	6.89E-02
Phvul.001G200900	K02889 - large subunit ribosomal protein L21e (RP-L21e, RPL21) (1 of 2)	-0.20	5.36E-01	-0.39	6.51E-02	-0.08	8.55E-01	-0.52	1.40E-02
Phvul.001G245600	K03237 - translation initiation factor 2 subunit 1 (EIF2S1) (1 of 2)	-0.20	4.74E-01	-0.39	4.48E-02	-0.17	6.23E-01	-0.44	2.57E-02
Phvul.009G176100	no data	-0.20	5.13E-01	-0.21	3.62E-01	-0.21	5.30E-01	-0.43	4.16E-02
Phvul.003G102100	PF00010 - Helix-loop-helix DNA-binding domain (HLH) (1 of 158)	-0.20	7.66E-01	-0.83	2.94E-02	-0.38	5.47E-01	-0.71	7.64E-02
Phvul.008G204800	PTHR33882:SF3 - RPM1-INTERACTING PROTEIN 4 (RIN4) FAMILY PROTEIN (1 of 3)	-0.20	6.12E-01	-0.32	2.31E-01	-0.21	6.27E-01	-0.55	2.91E-02
Phvul.009G032200	PF00646 - F-box domain (F-box) (1 of 135)	-0.20	4.37E-01	-0.55	1.43E-03	-0.29	2.53E-01	-0.35	5.78E-02
Phvul.002G188400	PTHR10366//PTHR10366:SF389 - NAD DEPENDENT EPIMERASE/DEHYDRATASE // SUBFAMILY NOT NAMED (1 of 2)	-0.20	4.42E-01	-0.33	7.09E-02	-0.22	4.43E-01	-0.37	4.50E-02
Phvul.003G143700	no data	-0.20	9.08E-01	-1.88	2.64E-02	-0.35	8.34E-01	-0.67	5.02E-01
Phvul.011G071600	K03018 - DNA-directed RNA polymerase III subunit RPC1 (RPC1, POLR3A) (1 of 1)	-0.20	6.00E-01	-0.75	3.48E-03	-0.49	1.37E-01	-0.38	1.64E-01
Phvul.001G012400	K02909 - large subunit ribosomal protein L31 (RP-L31, rpmE) (1 of 1)	-0.21	5.75E-01	-0.55	1.38E-02	0.03	9.65E-01	-0.43	7.13E-02
Phvul.005G050800	PTHR11406:SF8 - PHOSPHOGLYCERATE KINASE 1, CHLOROPLASTIC-RELATED (1 of 1)	-0.21	5.64E-01	-0.36	1.21E-01	-0.17	6.74E-01	-0.50	3.10E-02
Phvul.001G208200	K02883 - large subunit ribosomal protein L18e (RP-L18e, RPL18) (1 of 4)	-0.21	6.24E-01	-0.31	2.78E-01	-0.15	7.81E-01	-0.60	2.16E-02
Phvul.003G220700	PTHR22594:SF6 - ASPARAGINE--TRNA LIGASE, MITOCHONDRIAL (1 of 1)	-0.21	2.85E-01	-0.36	9.15E-03	-0.13	5.71E-01	-0.26	7.16E-02
Phvul.009G252700	PTHR31875:SF6 - PROTEIN DEHYDRATION-INDUCED 19 HOMOLOG 4-RELATED (1 of 1)	-0.21	4.72E-01	-0.41	2.94E-02	-0.14	7.03E-01	-0.29	1.52E-01
Phvul.003G076700	PTHR24031:SF228 - PROTEIN Y54G11A.3 (1 of 1)	-0.21	4.37E-01	-0.43	1.47E-02	-0.23	4.19E-01	-0.35	6.17E-02
Phvul.004G092376	K08912 - light-harvesting complex II chlorophyll a/b binding protein 1 (LHCB1) (1 of 13)	-0.21	7.97E-01	-1.06	1.25E-02	-0.18	8.47E-01	-0.74	1.05E-01
Phvul.004G091600	PTHR21649:SF26 - CHLOROPHYLL A-B BINDING PROTEIN 1, CHLOROPLASTIC-RELATED (1 of 14)	-0.21	8.20E-01	-1.20	1.06E-02	-0.12	9.19E-01	-0.79	1.22E-01
Phvul.001G132200	K03403 - magnesium chelatase subunit H (chlH, bchH) (1 of 2)	-0.21	6.11E-01	-0.49	4.97E-02	-0.08	8.94E-01	-0.21	4.65E-01
Phvul.003G121200	PTHR11132//PTHR11132:SF80 - SOLUTE CARRIER FAMILY 35 // SUBFAMILY NOT NAMED (1 of 5)	-0.21	6.57E-01	-0.46	1.22E-01	-0.27	5.76E-01	-0.63	3.21E-02
Phvul.009G056400	K14503 - brassinosteroid resistant 1/2 (BZR1 2) (1 of 5)	-0.21	6.34E-01	-0.52	5.11E-02	-0.05	9.43E-01	-0.59	3.16E-02
Phvul.003G225000	PTHR14154//PTHR14154:SF14 - UPF0041 BRAIN PROTEIN 44-RELATED // SUBFAMILY NOT NAMED (1 of 1)	-0.21	5.14E-01	-0.56	4.62E-03	-0.08	8.67E-01	-0.27	2.35E-01
Phvul.001G047100	K00811 - aspartate aminotransferase, chloroplastic (ASP5) (1 of 1)	-0.21	6.71E-01	-0.72	1.48E-02	-0.42	3.47E-01	-0.65	3.57E-02
Phvul.001G148700	3.2.1.68 - Isoamylase / Debranching enzyme (1 of 4)	-0.21	7.14E-01	-0.67	4.85E-02	-0.25	6.85E-01	-0.53	1.40E-01
Phvul.002G171200	PTHR31089:SF4 - CYCLIC DOF FACTOR 2-RELATED (1 of 7)	-0.21	4.61E-01	-0.35	6.59E-02	-0.17	6.15E-01	-0.44	2.24E-02
Phvul.002G011600	K18811 - cyclin D5, plant (CYCD5) (1 of 2)	-0.21	8.39E-01	-1.70	4.51E-03	-0.42	6.68E-01	-0.94	1.15E-01
Phvul.003G012100	no data	-0.21	4.50E-01	-0.10	6.94E-01	-0.17	6.00E-01	-0.40	4.58E-02
Phvul.007G114400	PF05212 - Protein of unknown function (DUF707) (DUF707) (1 of 10)	-0.21	6.46E-01	-0.05	9.10E-01	-0.29	5.30E-01	-0.61	3.77E-02
Phvul.009G142900	PF00170 - bZIP transcription factor (bZIP 1) (1 of 66)	-0.21	6.92E-01	-0.78	1.47E-02	-0.37	4.52E-01	-0.65	5.37E-02
Phvul.003G152300	PTHR10366//PTHR10366:SF459 - NAD DEPENDENT EPIMERASE/DEHYDRATASE // SUBFAMILY NOT NAMED (1 of 1)	-0.21	5.48E-01	-0.53	1.47E-02	0.00	1.00E+00	-0.33	1.64E-01
Phvul.006G180200	PF07714//PF13855 - Protein tyrosine kinase (Pkinase Tyr) // Leucine rich repeat (LRR 8) (1 of 6)	-0.21	8.29E-01	1.26	8.14E-03	0.23	8.32E-01	0.96	6.21E-02
Phvul.002G161500	PTHR12608:SF6 - GDT1-LIKE PROTEIN 1, CHLOROPLASTIC (1 of 1)	-0.21	3.70E-01	-0.56	3.53E-04	-0.23	3.59E-01	-0.46	5.17E-03
Phvul.007G084300	K15306 - Ran-binding protein 1 (RANBP1) (1 of 1)	-0.21	5.06E-01	-0.36	8.64E-02	-0.18	6.28E-01	-0.48	2.10E-02
Phvul.001G108000	no data	-0.21	6.46E-01	-0.68	1.21E-02	-0.18	7.39E-01	-0.71	1.12E-02
Phvul.001G086900	K02148 - V-type H+-transporting ATPase subunit C (ATPeV1C, ATP6C) (1 of 2)	-0.21	6.29E-01	-0.34	2.48E-01	-0.04	9.56E-01	-0.55	4.66E-02
Phvul.007G278700	PTHR13528//PTHR13528:SF3 - 39S RIBOSOMAL PROTEIN L28, MITOCHONDRIAL // SUBFAMILY NOT NAMED (1 of 1)	-0.21	6.63E-01	-0.71	1.15E-02	-0.03	9.68E-01	-0.67	2.36E-02
Phvul.002G232300	PTHR11527:SF158 - 17.6 KDA CLASS I HEAT SHOCK PROTEIN 1-RELATED (1 of 11)	-0.21	4.65E-01	-0.47	1.31E-02	-0.11	7.87E-01	-0.29	1.62E-01
Phvul.006G028800	PF14368 - Probable lipid transfer (LTP 2) (1 of 66)	-0.21	7.63E-01	-0.92	1.71E-02	-0.19	8.18E-01	-0.69	9.40E-02
Phvul.002G102100	PTHR10693:SF27 - NUCLEAR TRANSPORT FACTOR 2 AND RNA RECOGNITION MOTIF DOMAIN-CONTAINING PROTEIN (1	-0.21	7.75E-01	-0.90	3.29E-02	-0.20	8.21E-01	-1.13	8.55E-03
Phvul.009G113300	PTHR15451:SF19 - ERGOSTEROL BIOSYNTHETIC PROTEIN 28-RELATED (1 of 1)	-0.21	6.83E-01	-0.79	1.35E-02	-0.20	7.35E-01	-0.66	5.13E-02
Phvul.L002243	K02893 - large subunit ribosomal protein L23Ae (RP-L23Ae, RPL23A) (1 of 4)	-0.21	5.50E-01	-0.39	9.95E-02	-0.21	5.94E-01	-0.54	2.06E-02
Phvul.009G214400	PF05512 - AWPM-19-like family (AWPM-19) (1 of 4)	-0.21	5.50E-01	-0.36	1.25E-01	-0.19	6.31E-01	-0.55	1.78E-02
Phvul.001G025000	3.5.1.1 - Asparaginase / L-asparagine amidohydrolase (1 of 3)	-0.21	7.43E-01	-0.73	4.82E-02	0.07	9.45E-01	-0.27	5.34E-01
Phvul.001G253300	K07904 - Ras-related protein Rab-11A (RAB11A) (1 of 22)	-0.21	4.04E-01	-0.38	3.67E-02	-0.02	9.60E-01	-0.29	1.35E-01
Phvul.010G093600	K03110 - fused signal recognition particle receptor (ftsY) (1 of 1)	-0.21	2.34E-01	-0.28	3.71E-02	-0.08	7.49E-01	-0.21	1.42E-01
Phvul.011G078900	no data	-0.22	6.35E-01	-0.75	5.19E-03	-0.03	9.74E-01	-0.58	4.40E-02
Phvul.004G091988	K08912 - light-harvesting complex II chlorophyll a/b binding protein 1 (LHCB1) (1 of 13)	-0.22	7.96E-01	-1.05	1.74E-02	-0.13	9.06E-01	-0.68	1.58E-01
Phvul.006G038600	PTHR23155//PTHR23155:SF443 - LEUCINE-RICH REPEAT-CONTAINING PROTEIN // SUBFAMILY NOT NAMED (1 of 5)	-0.22	4.50E-01	-0.45	1.72E-02	-0.06	8.89E-01	-0.30	1.37E-01
Phvul.003G237900	PTHR10992:SF776 - ESTERASE KAI2-RELATED (1 of 3)	-0.22	7.71E-01	0.92	1.53E-02	0.30	6.69E-01	0.77	5.52E-02

Phvul.003G153400	K14305 - nuclear pore complex protein Nup43 (NUP43) (1 of 1)	-0.22	6.65E-01	-0.56	7.50E-02	-0.19	7.54E-01	-0.84	7.25E-03
Phvul.005G116800	K10528 - hydroperoxide lyase (HPL) (1 of 1)	-0.22	5.84E-01	-0.55	2.55E-02	-0.09	8.62E-01	-0.34	2.04E-01
Phvul.008G106900	no data	-0.22	3.39E-01	-0.40	1.04E-02	-0.12	6.76E-01	-0.34	4.42E-02
Phvul.006G099500	K02736 - 20S proteasome subunit beta 7 (PSMB4) (1 of 1)	-0.22	5.59E-01	-0.35	1.58E-01	-0.21	6.06E-01	-0.54	2.67E-02
Phvul.011G037900	PTHR24015:SF364 - ATPASE EXPRESSION PROTEIN 3 (1 of 1)	-0.22	8.14E-01	-1.18	1.58E-02	-0.10	9.41E-01	-0.93	7.20E-02
Phvul.004G027100	1.4.1.24 - 3-dehydroquinate synthase II / DHQ synthase II (1 of 1)	-0.22	6.92E-01	-0.93	4.62E-03	-0.16	8.09E-01	-0.98	3.91E-03
Phvul.003G036000	PTHR21091:SF123 - HOMOCYSTEINE S-METHYLTRANSFERASE (1 of 1)	-0.22	7.52E-01	-1.21	1.59E-03	-0.11	9.11E-01	-0.92	2.39E-02
Phvul.005G081400	2.7.4.9 - dTMP kinase / TMPK (1 of 1)	-0.22	5.39E-01	-0.57	1.11E-02	-0.25	5.05E-01	-0.61	8.50E-03
Phvul.007G184950	PTHR10663:SF114 - PROTEIN MON2 HOMOLOG (1 of 1)	-0.22	3.68E-01	-0.04	8.62E-01	-0.43	3.67E-02	-0.09	6.84E-01
Phvul.008G133450	PTHR32227:SF65 - O-GLYCOSYL HYDROLASES FAMILY 17 PROTEIN (1 of 2)	-0.22	5.42E-01	-0.31	2.12E-01	-0.11	8.32E-01	-0.49	3.81E-02
Phvul.001G261100	PTHR12771:SF19 - ELMO/CED-12 DOMAIN-CONTAINING PROTEIN-RELATED (1 of 1)	-0.22	7.99E-01	-1.22	1.03E-02	-0.33	7.12E-01	-1.04	3.57E-02
Phvul.002G199300	PTHR32448:SF34 - F22M8.11 PROTEIN-RELATED (1 of 7)	-0.22	7.53E-01	-0.86	3.49E-02	0.00	9.98E-01	-0.67	1.20E-01
Phvul.006G165600	PTHR11624:SF76 - PYRUVATE DEHYDROGENASE E1 COMPONENT SUBUNIT BETA-2, CHLOROPLASTIC-RELATED (1 of 1)	-0.22	5.86E-01	-0.64	9.39E-03	-0.21	6.48E-01	-0.59	2.29E-02
Phvul.010G125100	K02998 - small subunit ribosomal protein SAe (RP-SAe, RPSA) (1 of 2)	-0.22	4.54E-01	-0.26	2.27E-01	-0.13	7.39E-01	-0.42	4.03E-02
Phvul.005G173200	K15918 - D-glycerate 3-kinase (GLYK) (1 of 1)	-0.22	5.68E-01	-0.63	7.50E-03	-0.16	7.33E-01	-0.50	4.75E-02
Phvul.009G251900	PTHR21493:SF121 - LIPASE CLASS 3 FAMILY PROTEIN (1 of 3)	-0.22	3.66E-01	-0.41	1.68E-02	-0.11	7.49E-01	-0.16	4.05E-01
Phvul.007G063200	PTHR27000:SF84 - LRR RECEPTOR-LIKE SERINE/THREONINE-PROTEIN KINASE ERL1-RELATED (1 of 2)	-0.22	8.74E-01	-1.81	2.23E-02	-0.11	9.55E-01	-1.22	1.31E-01
Phvul.001G194300	no data	-0.22	4.43E-01	-0.45	1.98E-02	-0.05	9.15E-01	-0.32	1.29E-01
Phvul.010G105900	PTHR12526:SF412 - GLYCOSYL TRANSFERASE FAMILY 1 PROTEIN (1 of 1)	-0.22	2.16E-01	-0.30	2.68E-02	-0.20	3.36E-01	-0.19	2.03E-01
Phvul.005G076600	PTHR23111//PTHR23111:SF24 - ZINC FINGER PROTEIN // SUBFAMILY NOT NAMED (1 of 1)	-0.22	7.89E-01	-0.61	2.12E-01	0.05	9.71E-01	-0.95	4.66E-02
Phvul.007G264300	PTHR13200:SF0 - N(6)-ADENINE-SPECIFIC DNA METHYLTRANSFERASE 2 (1 of 1)	-0.22	5.85E-01	-0.60	1.99E-02	-0.17	7.28E-01	-0.78	2.92E-03
Phvul.006G222000	PF01535//PF13041//PF13812 - PPR repeat (PPR) // PPR repeat family (PPR 2) // Pentatricopeptide repeat domain (PPR	-0.22	6.64E-01	-0.87	1.04E-02	-0.44	3.59E-01	-0.58	9.70E-02
Phvul.010G019800	no data	-0.22	5.62E-01	-0.56	2.10E-02	-0.21	6.28E-01	-0.45	7.71E-02
Phvul.006G128500	6.2.1.26 - o-succinylbenzoate--CoA ligase / OSB-CoA synthetase (1 of 1)	-0.22	5.44E-01	-0.36	1.40E-01	-0.15	7.41E-01	-0.65	5.75E-03
Phvul.002G288900	PTHR27001:SF147 - PROTEIN KINASE PROTEIN WITH TETRATRICOPEPTIDE REPEAT DOMAIN (1 of 1)	-0.22	5.93E-01	-0.63	1.88E-02	-0.36	3.68E-01	-0.78	4.43E-03
Phvul.010G084400	PTHR23067//PTHR23067:SF44 - DOUBLE-STRANDED RNA-BINDING ZINC FINGER PROTEIN // SUBFAMILY NOT NAMED (1	-0.22	5.23E-01	-0.62	5.19E-03	-0.15	7.31E-01	-0.41	8.61E-02
Phvul.003G251800	PTHR24326:SF281 - HOMEOBOX-LEUCINE ZIPPER PROTEIN ATHB-21-RELATED (1 of 2)	-0.22	8.74E-01	-1.44	4.27E-02	-0.70	5.60E-01	-0.98	1.88E-01
Phvul.006G163400	K02882 - large subunit ribosomal protein L18Ae (RP-L18Ae, RPL18A) (1 of 3)	-0.23	4.71E-01	-0.28	2.17E-01	-0.16	6.63E-01	-0.48	2.31E-02
Phvul.007G035000	1.12.98.4 - Sulfhydrogenase / Sulfur reductase (1 of 1)	-0.23	4.85E-01	-0.52	1.24E-02	-0.07	8.77E-01	-0.45	3.81E-02
Phvul.004G099800	2.7.11.1 - Non-specific serine/threonine protein kinase / Threonine-specific protein kinase (1 of 1198)	-0.23	7.00E-01	-0.85	1.09E-02	-0.12	8.77E-01	-1.03	2.40E-03
Phvul.L001972	3.4.21.102 - C-terminal processing peptidase / Tsp protease (1 of 5)	-0.23	4.65E-01	-0.56	4.93E-03	-0.12	7.70E-01	-0.43	4.64E-02
Phvul.003G297200	PTHR31155:SF9 - ACYL-[ACYL-CARRIER-PROTEIN] DESATURASE 7, CHLOROPLASTIC (1 of 1)	-0.23	4.92E-01	-0.47	2.73E-02	-0.12	7.84E-01	-0.51	2.10E-02
Phvul.005G181600	1.1.1.85 - 3-isopropylmalate dehydrogenase / IMDH (1 of 1)	-0.23	4.02E-01	-0.49	6.42E-03	-0.12	7.42E-01	-0.36	6.67E-02
Phvul.004G176700	PTHR10438:SF227 - THIOREDOXIN-LIKE 2-1, CHLOROPLASTIC-RELATED (1 of 1)	-0.23	2.20E-01	-0.48	2.47E-04	-0.16	4.70E-01	-0.35	1.22E-02
Phvul.011G013700	PTHR24009:SF3 - RNA RECOGNITION MOTIF-CONTAINING PROTEIN-RELATED (1 of 2)	-0.23	4.26E-01	-0.39	4.22E-02	-0.28	3.25E-01	-0.38	5.54E-02
Phvul.001G087700	PTHR23032:SF2 - ENDOSOMAL TARGETING BRO1-LIKE DOMAIN-CONTAINING PROTEIN (1 of 1)	-0.23	3.67E-01	-0.44	1.12E-02	-0.22	4.25E-01	-0.19	3.27E-01
Phvul.003G091600	PTHR11260//PTHR11260:SF293 - GLUTATHIONE S-TRANSFERASE, GST, SUPERFAMILY, GST DOMAIN CONTAINING // SUE	-0.23	6.96E-01	-0.70	3.80E-02	-0.15	8.33E-01	-0.60	9.44E-02
Phvul.009G064300	K14499 - BRI1 kinase inhibitor 1 (BKI1) (1 of 2)	-0.23	7.61E-01	-1.37	6.73E-04	-0.26	7.54E-01	-0.83	5.62E-02
Phvul.007G244900	PTHR22847:SF502 - WD40 REPEAT PROTEIN MUCILAGE-MODIFIED 1 (1 of 1)	-0.23	3.08E-01	-0.45	4.04E-03	-0.13	6.63E-01	-0.32	5.49E-02
Phvul.006G122300	no data	-0.23	5.83E-01	-0.74	3.45E-03	-0.27	5.26E-01	-0.45	1.05E-01
Phvul.004G168100	K02357 - elongation factor Ts (tsf, TSFM) (1 of 1)	-0.23	5.99E-01	-0.62	1.71E-02	-0.19	7.02E-01	-0.54	4.87E-02
Phvul.010G154500	PF01918 - Alba (Alba) (1 of 8)	-0.23	4.48E-01	-0.43	3.35E-02	-0.14	7.15E-01	-0.41	4.99E-02
Phvul.010G044800	PTHR15615:SF28 - CYCLIN-U4-1 (1 of 2)	-0.23	7.81E-01	-1.24	1.14E-02	-0.23	8.08E-01	-1.19	1.96E-02
Phvul.003G141800	no data	-0.23	6.41E-01	-0.59	4.71E-02	-0.31	5.29E-01	-0.49	1.23E-01
Phvul.008G016700	PTHR21240 - 2-AMINO-3-CARBOXYLMUCONATE-6-SEMIALDEHYDE DECARBOXYLASE (1 of 1)	-0.23	7.03E-01	-0.83	1.49E-02	-0.21	7.62E-01	-0.78	2.98E-02
Phvul.002G216600	1.1.1.316 - L-galactose 1-dehydrogenase / L-galDH (1 of 1)	-0.23	5.04E-01	-0.55	1.16E-02	-0.08	8.79E-01	-0.33	1.75E-01
Phvul.009G040800	PTHR31851:SF16 - MEMBRANE PROTEIN OF ER BODY 2 (1 of 4)	-0.23	7.07E-01	-0.79	2.56E-02	-0.26	6.86E-01	-0.73	4.85E-02
Phvul.001G003100	K00215 - 4-hydroxy-tetrahydrodipicolinate reductase (dapB) (1 of 2)	-0.23	5.35E-01	-0.45	6.31E-02	-0.28	4.57E-01	-0.55	2.63E-02
Phvul.001G125800	PTHR26402:SF507 - TWO-COMPONENT RESPONSE REGULATOR ARR8-RELATED (1 of 1)	-0.23	7.01E-01	-0.80	2.00E-02	-0.22	7.49E-01	-0.58	1.15E-01
Phvul.001G119100	no data	-0.23	3.43E-01	-0.44	8.00E-03	-0.18	5.31E-01	-0.26	1.69E-01
Phvul.002G206700	PTHR31827:SF1 - EMB (1 of 1)	-0.23	4.81E-01	-0.63	2.51E-03	-0.04	9.42E-01	-0.47	3.43E-02
Phvul.002G150100	K02900 - large subunit ribosomal protein L27Ae (RP-L27Ae, RPL27A) (1 of 3)	-0.23	4.14E-01	-0.26	2.19E-01	-0.05	9.16E-01	-0.44	2.80E-02
Phvul.002G324300	PTHR31065:SF15 - PLATZ TRANSCRIPTION FACTOR FAMILY PROTEIN (1 of 1)	-0.23	5.19E-01	-0.34	1.61E-01	-0.03	9.61E-01	-0.57	1.58E-02
Phvul.006G006300	PTHR35491:SF2 - DENTIN SIALOPHOSPHOPROTEIN-RELATED PROTEIN (1 of 1)	-0.23	8.52E-01	-0.54	4.67E-01	0.29	8.18E-01	-1.72	1.57E-02
Phvul.009G166900	K11650 - SWI/SNF-related matrix-associated actin-dependent regulator of chromatin subfamily D (SMARCD) (1 of 4)	-0.23	7.54E-01	-1.17	8.77E-03	-0.22	8.03E-01	-0.86	6.12E-02
Phvul.004G157900	PTHR35735:SF1 - PROTEIN NIM1-INTERACTING 2 (1 of 3)	-0.23	8.28E-01	1.30	1.43E-02	0.27	8.14E-01	1.27	2.15E-02
Phvul.007G150300	K00856 - adenosine kinase (E2.7.1.20, ADK) (1 of 3)	-0.23	6.75E-01	-0.72	2.61E-02	-0.31	5.90E-01	-0.87	8.67E-03
Phvul.005G076100	PF03106 - WRKY DNA -binding domain (WRKY) (1 of 91)	-0.23	6.37E-01	-0.70	2.26E-02	-0.01	9.93E-01	-0.73	2.18E-02
Phvul.009G141800	K02976 - small subunit ribosomal protein S26e (RP-S26e, RPS26) (1 of 3)	-0.23	4.45E-01	-0.41	4.42E-02	-0.11	7.93E-01	-0.51	1.52E-02
Phvul.002G001200	PTHR15680:SF10 - 50S RIBOSOMAL PROTEIN L19-1, CHLOROPLASTIC-RELATED (1 of 1)	-0.23	3.80E-01	-0.45	1.19E-02	-0.13	7.26E-01	-0.27	1.80E-01
Phvul.011G011700	PTHR11586:SF18 - NUCLEIC ACID-BINDING, OB-FOLD-LIKE PROTEIN (1 of 1)	-0.23	3.95E-01	-0.48	1.07E-02	-0.08	8.47E-01	-0.24	2.60E-01
Phvul.005G012100	K02906 - large subunit ribosomal protein L3 (RP-L3, MRPL3, rplC) (1 of 2)	-0.23	6.03E-01	-0.22	5.17E-01	-0.11	8.58E-01	-0.63	2.89E-02
Phvul.004G099700	PF00560//PF08263//PF13855 - Leucine Rich Repeat (LRR 1) // Leucine rich repeat N-terminal domain (LRRNT 2) // Leuc	-0.23	7.99E-01	0.99	4.21E-02	-0.51	5.46E-01	0.19	7.73E-01
Phvul.009G177600	PTHR11132//PTHR11132:SF120 - SOLUTE CARRIER FAMILY 35 // SUBFAMILY NOT NAMED (1 of 2)	-0.24	6.11E-01	-0.35	2.58E-01	-0.31	5.05E-01	-0.69	1.84E-02
Phvul.011G157800	PTHR12313:SF2 - E3 UBIQUITIN-PROTEIN LIGASE RMA3 (1 of 1)	-0.24	4.65E-01	-0.55	9.34E-03	0.00	9.95E-01	-0.43	5.63E-02
Phvul.006G082200	no data	-0.24	5.87E-01	-0.88	6.73E-04	-0.32	4.53E-01	-0.71	8.91E-03
Phvul.007G090300	K00627 - pyruvate dehydrogenase E2 component (dihydrolipoamide acetyltransferase) (DLAT, aceF, pdhC) (1 of 4)	-0.24	5.34E-01	-0.65	6.31E-03	-0.24	5.62E-01	-0.76	1.76E-03
Phvul.008G081300	PTHR11584 - SERINE/THREONINE PROTEIN KINASE (1 of 3)	-0.24	7.16E-01	-1.03	5.35E-03	-0.12	8.92E-01	-0.48	2.42E-01
Phvul.008G098400	PTHR24349//PTHR24349:SF145 - SERINE/THREONINE-PROTEIN KINASE // SUBFAMILY NOT NAMED (1 of 1)	-0.24	5.18E-01	-0.55	1.89E-02	-0.35	3.15E-01	-0.57	1.84E-02
Phvul.003G140500	2.7.10.2//2.7.11.1 - Non-specific protein-tyrosine kinase / Cytoplasmic protein tyrosine kinase // Non-specific serine/thr	-0.24	5.84E-01	0.45	1.02E-01	0.08	9.03E-01	0.56	4.06E-02
Phvul.003G226400	K02921 - large subunit ribosomal protein L37Ae (RP-L37Ae, RPL37A) (1 of 2)	-0.24	4.58E-01	-0.49	1.79E-02	-0.07	8.80E-01	-0.58	6.47E-03
Phvul.004G029800	PF09366 - Protein of unknown function (DUF1997) (DUF1997) (1 of 4)	-0.24	6.89E-01	-0.73	3.58E-02	-0.13	8.59E-01	-0.58	1.18E-01
Phvul.008G180300	no data	-0.24	7.87E-01	-1.20	1.10E-02	-0.19	8.54E-01	-0.87	8.43E-02
Phvul.006G088800	PTHR35760:SF1 - SI:CH211-22I13.2 PROTEIN (1 of 1)	-0.24	4.58E-01	-0.28	2.25E-01	-0.13	7.69E-01	-0.46	3.62E-02
Phvul.007G244000	K12865 - polyglutamine-binding protein 1 (PQBP1, NPW38) (1 of 1)	-0.24	2.54E-01	-0.48	1.07E-03	-0.20	4.05E-01	-0.40	1.03E-02
Phvul.003G263800	PTHR22601//PTHR22601:SF12 - ISP4 LIKE PROTEIN // SUBFAMILY NOT NAMED (1 of 5)	-0.24	5.77E-01	-0.53	4.81E-02	-0.49	1.98E-01	-0.79	3.04E-03
Phvul.001G201900	no data	-0.24	7.98E-01	-1.14	2.23E-02	-0.21	8.42E-01	-1.03	4.76E-02
Phvul.001G057800	PF00631 - GGL domain (G-gamma) (1 of 4)	-0.24	5.99E-01	-0.60	3.17E-02	-0.15	7.97E-01	-0.68	1.92E-02
Phvul.009G238800	no data	-0.24	9.05E-01	-2.47	8.28E-03	-0.41	8.34E-01	-1.44	1.54E-01
Phvul.011G027700	PTHR12042//PTHR12042:SF13 - LACTOSYLCERAMIDE 4-ALPHA-GALACTOSYLTRANSFERASE ALPHA- 1,4-GALACTOSYLTRA	-0.24	5.72E-01	-0.64	1.19E-02	-0.02	9.84E-01	-0.52	5.87E-02
Phvul.001G003700	PTHR31867:SF4 - EXPANSIN-A11 (1 of 2)	-0.24	8.52E-01	-1.62	1.53E-02	-0.42	7.35E-01	-0.94	1.94E-01
Phvul.L001795	no data	-0.24	3.92E-01	-0.51	7.86E-03	-0.25	4.16E-01	-0.40	5.03E-02
Phvul.002G248000	PTHR24098:SF4 - ACYL-COA N-ACYLTRANSFERASE WITH RING/FYVE/PHD-TYPE ZINC FINGER DOMAIN-RELATED (1 of 3)	-0.24	2.12E-01	-0.36	1.15E-02	-0.14	5.86E-01	-0.29	5.96E-02
Phvul.009G060300	K07056 - 16S rRNA (cytidine1402-2'-O)-methyltransferase [EC:2.1.1.198] (rsmI) (1 of 1)	-0.24	4.38E-01	-0.52	1.21E-02	-0.12	7.76E-01	-0.37	9.80E-02
Phvul.009G249000	PTHR22844//PTHR22844:SF235 - F-BOX AND WD40 DOMAIN PROTEIN // SUBFAMILY NOT NAMED (1 of 1)	-0.24	7.44E-01	-0.97	1.66E-02	-0.44	5.16E-01	-0.90	3.60E-02
Phvul.002G125100	PTHR27000:SF202 - INACTIVE LEUCINE-RICH REPEAT RECEPTOR-LIKE PROTEIN KINASE CORYNE-RELATED (1 of 1)	-0.24	3.15E-01	-0.34	4.97E-02	-0.24	3.58E-01	-0.23	2.20E-01
Phvul.003G113400	PTHR20858:SF18 - PROTEIN PET18 (1 of 1)	-0.24	6.92E-01	-0.96	8.00E-03	-0.13	8.71E-01	-0.47	2.36E-01
Phvul.011G025500	PTHR22880:SF5 - NUCLEOSOME-REMODELING FACTOR SUBUNIT BPTF (1 of 1)	-0.24	8.00E-01	-1.17	2.25E-02	-0.19	8.68E-01	-0.90	9.90E-02
Phvul.002G081300	PTHR27000:SF120 - LEUCINE-RICH REPEAT (LRR) FAMILY PROTEIN-RELATED (1 of 1)	-0.24	3.78E-01	-0.40	3.84E-02	-0.26	3.60E-01	-0.34	8.88E-02
Phvul.003G159100	K02924 - large subunit ribosomal protein L39e (RP-L39e, RPL39) (1 of 3)	-0.24	4.71E-01	-0.44	4.92E-02	-0.16	6.93E-01	-0.56	1.24E-02
Phvul.001G261701	no data	-0.24	8.24E-01	-2.25	3.52E-03	0.38	7.12E-01	-0.53	4.86E-01
Phvul.008G130500	K05291 - phosphatidylinositol glycan, class S (PIGS) (1 of 1)	-0.24	7.00E-01	-0.82	3.41E-02	-0.17	8.31E-01	-0.88	2.66E-02
Phvul.009G027200	KOG0978//KOG4593//KOG4674 - E3 ubiquitin ligase involved in syntaxin degradation // Mitotic checkpoint protein MAD	-0.24	2.81E-01	-0.23	1.84E-01	-0.40	4.69E-02	-0.25	1.50E-01
Phvul.004G120200	PTHR32295:SF11 - IQ-DOMAIN 24 PROTEIN (1 of 2)	-0.24	5.00E-01	-0.46	4.96E-02	-0.26	4.83E-01	-0.34	1.75E-01
Phvul.006G218100	2.4.2.17 - ATP phosphoribosyltransferase / Phosphoribosyl-ATP pyrophosphorylase (1 of 1)	-0.24	5.56E-01	-0.66	9.27E-03	-0.18	7.09E-01	-0.72	6.10E-03
Phvul.005G031700	K02899 - large subunit ribosomal protein L27 (RP-L27, MRPL27, rpmA) (1 of 2)	-0.24	4.90E-01	-0.56	1.31E-02	-0.11	8.27E-01	-0.38	1.14E-01
Phvul.008G142500	PTHR27007:SF61 - RECEPTOR LIKE PROTEIN KINASE S.2 (1 of 2)	-0.24	8.20E-01	-1.40	1.23E-02	-0.16	9.04E-01	-0.91	1.33E-01
Phvul.006G100400	PF05910 - Plant protein of unknown function (DUF868) (DUF868) (1 of 16)	-0.24	4.90E-01	-0.49	3.47E-02	-0.25	5.17E-01	-0.34	1.80E-01
Phvul.006G163500	K02955 - small subunit ribosomal protein S14e (RP-S14e, RPS14) (1 of 3)	-0.24	4.27E-01	-0.31	1.62E-01	-0.15	7.07E-01	-0.50	1.87E-02
Phvul.002G249200	2.7.11.1 - Non-specific serine/threonine protein kinase / Threonine-specific protein kinase (1 of 1198)	-0.24	3.52E-01	-0.48	7.58E-03	-0.42	6.62E-02	-0.55	3.10E-03
Phvul.002G281900	K03107 - signal recognition particle subunit SRP68 (SRP68) (1 of 1)	-0.24	6.47E-01	-0.94	2.10E-03	-0.11	8.79E-01	-0.71	3.16E-02
Phvul.003G191700	PTHR32295:SF14 - PROTEIN IQ-DOMAIN 31-RELATED (1 of 4)	-0.24	5.23E-01	-0.65	5.97E-03	-0.24	5.58E-01	-0.50	5.12E-02
Phvul.008G270400	K09191 - general transcription factor IIIA (GTF3A) (1 of 2)	-0.24	3.26E-01	-0.31	8.86E-02	-0.23	3.91E-01	-0.41	2.39E-02

Phvul.001G060900	K17260 - actin-related protein 2 (ACTR2, ARP2) (1 of 4)	-0.24	4.45E-01	-0.43	5.08E-02	-0.36	2.46E-01	-0.48	3.27E-02
Phvul.008G123700	no data	-0.24	7.25E-01	-0.81	4.47E-02	-0.56	3.55E-01	-0.96	1.90E-02
Phvul.007G264200	K02989 - small subunit ribosomal protein S5e (RP-S5e, RPS5) (1 of 2)	-0.24	4.91E-01	-0.34	1.68E-01	-0.14	7.53E-01	-0.55	2.11E-02
Phvul.002G217100	2.6.1.83 - LL-diaminopimelate aminotransferase / LL-diaminopimelate transaminase (1 of 2)	-0.24	4.33E-01	-0.49	1.82E-02	-0.27	4.22E-01	-0.43	4.64E-02
Phvul.004G001400	PTHR12358//PTHR12358:SF39 - SPHINGOSINE KINASE // SUBFAMILY NOT NAMED (1 of 1)	-0.24	4.97E-01	-0.61	7.57E-03	-0.07	8.98E-01	-0.38	1.22E-01
Phvul.L004437	PTHR23308//PTHR23308:SF29 - NUCLEAR INHIBITOR OF PROTEIN PHOSPHATASE-1 // SUBFAMILY NOT NAMED (1 of 1)	-0.25	2.78E-01	-0.39	1.59E-02	-0.19	4.77E-01	-0.20	2.65E-01
Phvul.001G219900	PTHR22572//PTHR22572:SF112 - SUGAR-1-PHOSPHATE GUANYL TRANSFERASE // SUBFAMILY NOT NAMED (1 of 1)	-0.25	7.78E-01	-0.91	5.77E-02	-0.63	4.34E-01	-0.97	4.48E-02
Phvul.001G111400	PTHR10110:SF117 - SODIUM/HYDROGEN EXCHANGER 1-RELATED (1 of 4)	-0.25	6.60E-01	-0.77	1.85E-02	-0.17	8.04E-01	-0.64	6.67E-02
Phvul.003G206700	K11294 - nucleolin (NCL, NSR1) (1 of 6)	-0.25	5.83E-01	-0.62	2.54E-02	-0.14	8.13E-01	-0.52	7.39E-02
Phvul.010G101600	PTHR11926:SF152 - UDP-GLUCOSYL TRANSFERASE 73B2-RELATED (1 of 5)	-0.25	8.22E-01	-1.47	1.28E-02	-0.23	8.52E-01	-1.40	2.21E-02
Phvul.006G082300	PTHR35515:SF1 - NADH DEHYDROGENASE-LIKE COMPLEX N (1 of 1)	-0.25	3.40E-01	-0.57	1.04E-03	-0.24	3.89E-01	-0.47	1.03E-02
Phvul.006G116300	PTHR19359:SF22 - CYTOCHROME B5 ISOFORM A (1 of 3)	-0.25	6.86E-01	-1.04	2.41E-03	-0.18	8.10E-01	-0.75	4.28E-02
Phvul.001G179300	PTHR24221:SF119 - ABC TRANSPORTER B FAMILY MEMBER 1 (1 of 2)	-0.25	6.68E-01	-0.77	2.42E-02	-0.44	4.07E-01	-0.71	4.69E-02
Phvul.002G327800	PTHR19856 - WD-REPEATCONTAINING PROTEIN WDR1 (1 of 2)	-0.25	7.72E-01	-1.19	1.09E-02	-0.39	6.48E-01	-0.94	5.71E-02
Phvul.006G155200	K02957 - small subunit ribosomal protein S15Ae (RP-S15Ae, RPS15A) (1 of 5)	-0.25	3.70E-01	-0.23	2.71E-01	-0.26	3.71E-01	-0.44	2.58E-02
Phvul.005G113700	PTHR12197:SF75 - HISTONE-LYSINE N-METHYLTRANSFERASE ATXR2 (1 of 1)	-0.25	4.82E-01	-0.31	2.22E-01	-0.44	1.75E-01	-0.52	3.43E-02
Phvul.004G045400	2.7.7.4 - Sulfate adenylyltransferase / Sulfurylase (1 of 2)	-0.25	7.02E-01	-0.73	5.62E-02	-0.20	7.97E-01	-0.79	4.39E-02
Phvul.006G121100	PTHR10209:SF160 - GIBBERELLIN 2-BETA-DIOXYGENASE 4 (1 of 2)	-0.25	3.74E-01	-0.36	7.23E-02	-0.25	4.10E-01	-0.41	4.29E-02
Phvul.002G319500	PTHR11886:SF39 - DYNEIN LIGHT CHAIN TYPE 1-LIKE PROTEIN (1 of 2)	-0.25	5.97E-01	-0.73	1.60E-02	-0.48	2.61E-01	-0.60	5.81E-02
Phvul.003G153700	K02916 - large subunit ribosomal protein L35 (RP-L35, MRPL35, rpmI) (1 of 1)	-0.25	3.86E-01	-0.55	4.13E-03	-0.17	6.27E-01	-0.47	2.00E-02
Phvul.010G065700	3.1.1.11 - Pectinesterase / Pectin methylesterase (1 of 92)	-0.25	3.17E-01	-0.36	4.43E-02	-0.13	6.91E-01	-0.29	1.30E-01
Phvul.007G040300	PTHR34548:SF2 - PROTEIN TIC 21, CHLOROPLASTIC (1 of 1)	-0.25	4.33E-01	-0.52	1.41E-02	-0.20	6.03E-01	-0.54	1.30E-02
Phvul.008G176700	PTHR31356:SF6 - THYLAKOID LUMENAL 29 KDA PROTEIN, CHLOROPLASTIC (1 of 1)	-0.25	6.93E-01	-0.88	1.49E-02	-0.22	7.74E-01	-0.63	1.07E-01
Phvul.007G067100	PTHR32295:SF11 - IQ-DOMAIN 24 PROTEIN (1 of 2)	-0.25	3.08E-01	-0.36	4.07E-02	-0.17	5.63E-01	-0.15	4.86E-01
Phvul.001G153900	PTHR21091:SF6 - HOMOCYSTEINE S-METHYLTRANSFERASE 2 (1 of 1)	-0.25	5.57E-01	-0.73	6.42E-03	-0.21	6.63E-01	-0.74	7.64E-03
Phvul.007G147800	PTHR10343:SF60 - SNF1 PROTEIN KINASE SUBUNIT BETA-2-RELATED (1 of 1)	-0.25	6.11E-01	-0.67	2.84E-02	-0.23	6.76E-01	-0.36	2.83E-01
Phvul.010G108500	PTHR33222:SF4 - PROTEIN CURVATURE THYLAKOID 1A, CHLOROPLASTIC (1 of 1)	-0.25	5.02E-01	-0.74	1.51E-03	-0.22	6.11E-01	-0.57	2.24E-02
Phvul.005G126000	PTHR21141:SF48 - 60S ACIDIC RIBOSOMAL PROTEIN P2-4 (1 of 1)	-0.25	4.35E-01	-0.46	3.16E-02	-0.15	7.32E-01	-0.60	5.59E-03
Phvul.011G190300	PTHR10795:SF444 - SUBTILISIN-LIKE SERINE ENDOPEPTIDASE FAMILY PROTEIN (1 of 1)	-0.25	5.25E-01	-0.72	7.48E-03	-0.20	6.65E-01	-0.49	8.32E-02
Phvul.003G060300	no data	-0.25	5.08E-01	-0.58	1.70E-02	-0.19	6.79E-01	-0.44	8.71E-02
Phvul.011G086500	PTHR21368:SF18 - 39S RIBOSOMAL PROTEIN L9, MITOCHONDRIAL (1 of 1)	-0.25	3.92E-01	-0.57	3.78E-03	-0.24	4.74E-01	-0.60	3.19E-03
Phvul.005G105900	K08902 - photosystem II Psb27 protein (psb27) (1 of 1)	-0.25	7.96E-01	-1.27	1.58E-02	-0.04	9.82E-01	-1.12	3.98E-02
Phvul.002G058900	PF13912 - C2H2-type zinc finger (zf-C2H2 6) (1 of 42)	-0.25	6.78E-01	-0.81	2.72E-02	-0.32	6.19E-01	-0.46	2.53E-01
Phvul.001G004800	PTHR11588//PTHR11588:SF118 - TUBULIN // SUBFAMILY NOT NAMED (1 of 2)	-0.25	5.94E-01	-0.69	1.79E-02	-0.13	8.33E-01	-0.51	1.00E-01
Phvul.006G167200	K10683 - BRCA1-associated RING domain protein 1 [EC:6.3.2.19] (BARD1) (1 of 1)	-0.25	7.99E-01	-0.92	1.34E-01	-0.97	2.26E-01	-1.27	3.97E-02
Phvul.009G171500	K11294 - nucleolin (NCL, NSR1) (1 of 6)	-0.25	4.59E-01	-0.46	4.43E-02	-0.18	6.75E-01	-0.44	6.46E-02
Phvul.003G208400	no data	-0.25	7.68E-01	-0.63	2.60E-01	-0.83	2.31E-01	-1.21	2.48E-02
Phvul.L001941	K02918 - large subunit ribosomal protein L35e (RP-L35e, RPL35) (1 of 3)	-0.25	5.15E-01	-0.35	2.03E-01	-0.08	8.92E-01	-0.62	1.69E-02
Phvul.003G180100	no data	-0.26	4.00E-01	-0.49	1.71E-02	-0.08	8.59E-01	-0.18	4.48E-01
Phvul.001G186000	PTHR10430:SF16 - PEROXIREDOXIN-5, MITOCHONDRIAL (1 of 1)	-0.26	6.70E-01	-0.86	1.42E-02	0.03	9.79E-01	-0.90	1.33E-02
Phvul.001G086600	PTHR11937:SF37 - ACTIN-RELATED PROTEIN 2 (1 of 6)	-0.26	5.06E-01	-0.29	2.93E-01	-0.20	6.57E-01	-0.55	3.28E-02
Phvul.009G135200	PTHR23423//PTHR23423:SF27 - ORGANIC SOLUTE TRANSPORTER-RELATED // SUBFAMILY NOT NAMED (1 of 2)	-0.26	5.26E-01	-0.69	6.81E-03	-0.33	4.23E-01	-0.95	1.95E-04
Phvul.007G243300	PTHR12565:SF158 - TRANSCRIPTION FACTOR BHLH106-RELATED (1 of 2)	-0.26	6.75E-01	-0.75	4.71E-02	-0.06	9.48E-01	-0.67	9.02E-02
Phvul.007G089400	PTHR21349:SF2 - 50S RIBOSOMAL PROTEIN L21, CHLOROPLASTIC (1 of 1)	-0.26	3.68E-01	-0.63	9.42E-04	-0.16	6.48E-01	-0.33	1.14E-01
Phvul.007G162900	K02930 - large subunit ribosomal protein L4e (RP-L4e, RPL4) (1 of 2)	-0.26	4.87E-01	-0.39	1.30E-01	-0.15	7.49E-01	-0.51	4.06E-02
Phvul.006G135500	PTHR32116:SF7 - GALACTURONOSYLTRANSFERASE 4-RELATED (1 of 2)	-0.26	6.06E-01	-0.85	5.07E-03	-0.32	5.27E-01	-0.76	1.66E-02
Phvul.007G201500	KOG1877 - Putative transmembrane protein cmp44E (1 of 4)	-0.26	1.53E-01	-0.34	1.53E-02	-0.30	1.12E-01	-0.24	1.19E-01
Phvul.009G121200	PTHR23086:SF33 - PHOSPHATIDYLINOSITOL 4-PHOSPHATE 5-KINASE 1-RELATED (1 of 3)	-0.26	2.67E-01	-0.38	2.41E-02	-0.22	4.03E-01	-0.29	1.05E-01
Phvul.002G308400	no data	-0.26	3.54E-01	-0.63	8.11E-04	-0.29	3.18E-01	-0.67	5.07E-04
Phvul.003G208500	PTHR11349:SF44 - NUCLEOSIDE DIPHOSPHATE KINASE II, CHLOROPLASTIC (1 of 1)	-0.26	3.68E-01	-0.49	1.29E-02	-0.16	6.74E-01	-0.31	1.57E-01
Phvul.005G107000	K02989 - small subunit ribosomal protein S5e (RP-S5e, RPS5) (1 of 2)	-0.26	5.08E-01	-0.36	1.90E-01	-0.28	5.05E-01	-0.62	1.75E-02
Phvul.010G108400	PTHR13359 - 39S RIBOSOMAL PROTEIN L40, MITOCHONDRIAL (1 of 1)	-0.26	3.16E-01	-0.30	1.21E-01	-0.12	7.29E-01	-0.39	4.35E-02
Phvul.002G217400	PTHR11825:SF53 - BRANCHED-CHAIN-AMINO-ACID AMINOTRANSFERASE 6-RELATED (1 of 1)	-0.26	8.37E-01	-2.00	7.46E-03	0.01	9.97E-01	-1.39	6.18E-02
Phvul.001G261000	K02880 - large subunit ribosomal protein L17e (RP-L17e, RPL17) (1 of 3)	-0.26	4.49E-01	-0.33	1.91E-01	-0.14	7.61E-01	-0.56	1.77E-02
Phvul.008G081100	K07407 - alpha-galactosidase (E3.2.1.22B, galA, rafA) (1 of 3)	-0.26	5.69E-01	-0.82	4.32E-03	-0.43	3.30E-01	-0.53	8.65E-02
Phvul.003G095800	2.7.7.60 - 2-C-methyl-D-erythritol 4-phosphate cytidylyltransferase / MEP cytidylyltransferase (1 of 1)	-0.26	4.94E-01	-0.34	2.13E-01	-0.27	5.06E-01	-0.53	3.97E-02
Phvul.002G147300	PTHR31933:SF3 - O-FUCOSYLTRANSFERASE FAMILY PROTEIN (1 of 1)	-0.26	3.65E-01	-0.44	2.80E-02	-0.29	3.46E-01	-0.39	6.39E-02
Phvul.L002181	K02932 - large subunit ribosomal protein L5e (RP-L5e, RPL5) (1 of 3)	-0.26	3.16E-01	-0.30	1.20E-01	-0.18	5.86E-01	-0.41	3.24E-02
Phvul.L002432	PTHR31407:SF5 - PLASTID TRANSCRIPTIONALLY ACTIVE 3 (1 of 1)	-0.26	3.69E-01	-0.51	1.26E-02	-0.21	5.43E-01	-0.39	7.18E-02
Phvul.006G027801	PTHR31339:SF3 - GLYCOSIDE HYDROLASE FAMILY 28 PROTEIN / POLYGALACTURONASE (PECTINASE) FAMILY PROTEIN (1	-0.26	4.32E-01	-0.64	2.94E-03	-0.12	7.93E-01	-0.32	1.99E-01
Phvul.007G219800	K14488 - SAUR family protein (SAUR) (1 of 75)	-0.26	3.26E-01	-0.61	8.64E-04	-0.27	3.59E-01	-0.53	6.23E-03
Phvul.006G032100	PTHR12741:SF16 - CALLOSE SYNTHASE 7-RELATED (1 of 2)	-0.26	3.43E-01	-0.08	7.66E-01	-0.57	1.21E-02	0.03	9.09E-01
Phvul.010G158200	PTHR23042//PTHR23042:SF57 - CIRCADIAN PROTEIN CLOCK/ARNT/BMAL/PAS // SUBFAMILY NOT NAMED (1 of 13)	-0.26	7.61E-01	-1.23	9.27E-03	-0.24	8.09E-01	-1.06	3.35E-02
Phvul.007G156100	K16578 - CLIP-associating protein 1/2 (CLASP1 2) (1 of 2)	-0.27	2.38E-01	-0.36	3.40E-02	-0.39	6.18E-02	-0.24	2.01E-01
Phvul.006G161600	K19073 - divinyl chlorophyllide a 8-vinyl-reductase (DVR) (1 of 1)	-0.27	7.07E-01	-0.90	2.76E-02	-0.15	8.71E-01	-0.93	2.63E-02
Phvul.006G212300	PF02298 - Plastocyanin-like domain (Cu bind like) (1 of 53)	-0.27	2.61E-01	-0.35	4.96E-02	-0.36	1.25E-01	-0.37	4.46E-02
Phvul.011G011000	PF11891 - Domain of unknown function (DUF3411) (DUF3411) (1 of 9)	-0.27	2.29E-01	-0.43	7.49E-03	-0.13	6.61E-01	-0.33	5.69E-02
Phvul.003G049900	KOG2973 - Uncharacterized conserved protein (1 of 1)	-0.27	3.38E-01	-0.49	1.15E-02	-0.27	3.62E-01	-0.40	5.15E-02
Phvul.002G165600	K03404 - magnesium chelatase subunit D (chlD, bchD) (1 of 1)	-0.27	2.33E-01	-0.57	3.58E-04	-0.14	6.32E-01	-0.29	9.53E-02
Phvul.011G174000	PTHR11785:SF273 - CATIONIC AMINO ACID TRANSPORTER 8, VACUOLAR (1 of 1)	-0.27	5.26E-01	-0.52	5.98E-02	-0.39	3.48E-01	-0.69	1.35E-02
Phvul.001G052000	PTHR12169:SF5 - AFG1-LIKE ATPASE FAMILY PROTEIN (1 of 1)	-0.27	4.26E-01	-0.41	8.76E-02	-0.21	5.86E-01	-0.63	7.20E-03
Phvul.001G235900	K02922 - large subunit ribosomal protein L37e (RP-L37e, RPL37) (1 of 4)	-0.27	3.91E-01	-0.29	2.11E-01	-0.05	9.24E-01	-0.47	3.53E-02
Phvul.006G204300	K03319 - divalent anion:Na+ symporter, DASS family (TC.DASS) (1 of 1)	-0.27	6.33E-01	-0.75	2.58E-02	-0.12	8.76E-01	-0.66	6.31E-02
Phvul.009G049100	K01057 - 6-phosphogluconolactonase (PGLS, pgl, devB) (1 of 4)	-0.27	5.10E-01	-0.60	2.30E-02	-0.13	8.17E-01	-0.45	1.06E-01
Phvul.003G285700	K13422 - transcription factor MYC2 (MYC2) (1 of 3)	-0.27	6.34E-01	-0.76	2.59E-02	-0.06	9.51E-01	-0.31	4.44E-01
Phvul.009G183200	PF02985 - HEAT repeat (HEAT) (1 of 13)	-0.27	4.07E-01	-0.60	6.18E-03	-0.18	6.64E-01	-0.46	4.87E-02
Phvul.007G203600	K07937//K07977 - ADP-ribosylation factor 1 (ARF1) // Arf/Sar family, other (ARF) (1 of 6)	-0.27	3.74E-01	-0.51	1.42E-02	-0.18	6.23E-01	-0.48	2.73E-02
Phvul.006G068600	PTHR12356:SF18 - NUDC DOMAIN-CONTAINING PROTEIN 2 (1 of 1)	-0.27	8.94E-01	-2.44	1.12E-02	-0.29	8.96E-01	-1.84	6.95E-02
Phvul.003G276500	K02873 - large subunit ribosomal protein L13e (RP-L13e, RPL13) (1 of 3)	-0.27	4.55E-01	-0.31	2.51E-01	-0.28	4.77E-01	-0.55	2.95E-02
Phvul.009G259500	K13066 - caffeic acid 3-O-methyltransferase (E2.1.1.68, COMT) (1 of 18)	-0.27	6.67E-01	-0.79	4.76E-02	-0.19	8.07E-01	-0.57	1.81E-01
Phvul.011G042600	K05236 - coatomer protein complex, subunit alpha (xenin) (COPA) (1 of 2)	-0.27	6.34E-01	-0.78	2.32E-02	-0.20	7.78E-01	-0.71	4.81E-02
Phvul.002G177700	K02923 - large subunit ribosomal protein L38e (RP-L38e, RPL38) (1 of 3)	-0.27	4.09E-01	-0.39	9.66E-02	-0.22	5.68E-01	-0.58	1.08E-02
Phvul.011G046000	PTHR10366:SF254 - PINORESINOL-LARICIRESINOL REDUCTASE 3-RELATED (1 of 1)	-0.27	2.57E-01	-0.37	3.74E-02	-0.30	2.42E-01	-0.41	2.39E-02
Phvul.004G019800	no data	-0.27	3.48E-01	-0.54	6.21E-03	-0.30	3.20E-01	-0.48	2.07E-02
Phvul.007G231100	no data	-0.27	3.10E-01	-0.31	1.30E-01	-0.13	7.39E-01	-0.41	3.73E-02
Phvul.011G038200	K14488 - SAUR family protein (SAUR) (1 of 75)	-0.27	3.54E-01	-0.45	2.89E-02	-0.06	9.02E-01	-0.20	4.10E-01
Phvul.008G053000	PTHR23151:SF66 - BIOTIN CARBOXYL CARRIER PROTEIN OF ACETYL-COA CARBOXYLASE 1, CHLOROPLASTIC (1 of 1)	-0.27	4.47E-01	-0.61	1.00E-02	-0.17	7.14E-01	-0.65	7.79E-03
Phvul.011G104100	no data	-0.27	6.11E-01	-0.91	6.21E-03	-0.37	4.92E-01	-0.94	6.16E-03
Phvul.007G083400	K02964 - small subunit ribosomal protein S18e (RP-S18e, RPS18) (1 of 2)	-0.27	4.51E-01	-0.51	3.62E-02	-0.19	6.70E-01	-0.70	3.87E-03
Phvul.002G034500	2.5.1.61 - Hydroxymethylbilane synthase / Uroporphyrinogen synthetase (1 of 2)	-0.27	5.13E-01	-0.73	5.03E-03	-0.26	5.76E-01	-0.72	8.16E-03
Phvul.003G207300	PTHR24413:SF75 - BTB/POZ AND TAZ DOMAIN-CONTAINING PROTEIN 2 (1 of 2)	-0.27	6.39E-01	-0.71	4.84E-02	-0.08	9.29E-01	0.00	1.00E+00
Phvul.001G030600	K02946 - small subunit ribosomal protein S10 (RP-S10, RPS10, rpsJ) (1 of 2)	-0.27	2.49E-01	-0.53	2.01E-03	-0.21	4.47E-01	-0.33	7.71E-02
Phvul.011G079400	K02870 - large subunit ribosomal protein L12e (RP-L12e, RPL12) (1 of 6)	-0.28	3.84E-01	-0.35	1.21E-01	-0.17	6.68E-01	-0.52	2.04E-02
Phvul.008G056400	K15747 - beta-ring hydroxylase (LUT5, CYP97A3) (1 of 1)	-0.28	3.92E-01	-0.48	3.12E-02	-0.16	7.03E-01	-0.21	4.17E-01
Phvul.002G051500	no data	-0.28	2.34E-01	-0.27	1.40E-01	-0.31	2.15E-01	-0.36	4.50E-02
Phvul.004G050700	PF00076 - RNA recognition motif. (a.k.a. RRM, RBD, or RNP domain) (RRM 1) (1 of 252)	-0.28	5.13E-01	-0.64	2.68E-02	-0.52	1.75E-01	-0.57	5.75E-02
Phvul.011G076700	PTHR23155//PTHR23155:SF497 - LEUCINE-RICH REPEAT-CONTAINING PROTEIN // SUBFAMILY NOT NAMED (1 of 6)	-0.28	9.13E-01	2.45	2.65E-02	0.29	9.23E-01	1.97	9.15E-02
Phvul.003G118600	PTHR10724 - S1 RNA-BINDING DOMAIN-CONTAINING PROTEIN 1 (1 of 2)	-0.28	3.84E-01	-0.55	1.09E-02	-0.09	8.51E-01	-0.36	1.22E-01
Phvul.011G168200	PTHR11545:SF7 - 50S RIBOSOMAL PROTEIN L13, CHLOROPLASTIC (1 of 1)	-0.28	4.25E-01	-0.73	1.22E-03	-0.24	5.58E-01	-0.60	1.19E-02
Phvul.002G047800	no data	-0.28	5.87E-01	-0.73	2.36E-02	-0.52	2.60E-01	-0.94	3.83E-03
Phvul.002G012100	PTHR21314:SF0 - UPF0553 PROTEIN C9ORF64 (1 of 1)	-0.28	7.13E-01	-0.78	8.86E-02	-0.15	8.77E-01	-1.00	2.78E-02
Phvul.011G040700	PTHR11627:SF16 - FRUCTOSE-BISPHOSPHATE ALDOLASE 1, CHLOROPLASTIC-RELATED (1 of 2)	-0.28	6.09E-01	-1.10	7.24E-04	-0.42	4.29E-01	-0.67	5.54E-02
Phvul.003G088900	PTHR22814//PTHR22814:SF79 - COPPER TRANSPORT PROTEIN ATOX1-RELATED // SUBFAMILY NOT NAMED (1 of 1)	-0.28	2.26E-01	-0.33	5.76E-02	-0.32	1.91E-01	-0.39	2.80E-02

Phvul.008G203314	PF14009 - Domain of unknown function (DUF4228) (DUF4228) (1 of 38)	-0.28	1.71E-01	-0.36	2.46E-02	-0.26	2.67E-01	-0.14	4.41E-01
Phvul.004G075300	PTHR32295:SF32 - PROTEIN IQ-DOMAIN 3-RELATED (1 of 4)	-0.28	4.59E-01	-0.57	2.45E-02	-0.25	5.50E-01	-0.31	2.72E-01
Phvul.002G206400	PTHR11566:SF78 - DYNAMIN-LIKE PROTEIN ARC5 (1 of 1)	-0.28	4.34E-01	-0.78	8.46E-04	-0.29	4.49E-01	-0.57	2.21E-02
Phvul.003G143300	no data	-0.28	4.26E-01	-0.56	1.76E-02	-0.24	5.41E-01	-0.65	7.60E-03
Phvul.006G208400	PTHR11926//PTHR11926:SF227 - GLUCOSYL/GLUCURONOSYL TRANSFERASES // SUBFAMILY NOT NAMED (1 of 4)	-0.28	8.51E-01	-1.76	3.87E-02	-0.41	7.96E-01	-1.44	9.32E-02
Phvul.009G207900	K02924 - large subunit ribosomal protein L39e (RP-L39e, RPL39) (1 of 3)	-0.28	4.49E-01	-0.48	5.69E-02	-0.12	8.28E-01	-0.72	4.18E-03
Phvul.008G091600	PTHR21495:SF77 - DIRIGENT PROTEIN 19 (1 of 6)	-0.28	1.82E-01	-0.34	4.01E-02	-0.12	6.80E-01	-0.03	8.93E-01
Phvul.009G132000	PTHR10108:SF784 - METHYLTRANSFERASE PMT24-RELATED (1 of 2)	-0.28	5.50E-01	-0.68	2.25E-02	-0.26	6.31E-01	-0.34	3.04E-01
Phvul.001G175000	K02973 - small subunit ribosomal protein S23e (RP-S23e, RPS23) (1 of 4)	-0.28	5.38E-01	-0.47	1.23E-01	-0.24	6.56E-01	-0.77	9.79E-03
Phvul.003G163100	PTHR15725//PTHR15725:SF0 - ZN-FINGER, C-X8-C-X5-C-X3-H TYPE-CONTAINING // SUBFAMILY NOT NAMED (1 of 1)	-0.28	6.34E-01	-0.98	7.21E-03	-0.20	7.87E-01	-0.79	3.88E-02
Phvul.011G198400	PF00931//PF13855 - NB-ARC domain (NB-ARC) // Leucine rich repeat (LRR 8) (1 of 27)	-0.28	4.49E-01	-0.19	5.43E-01	-0.62	4.66E-02	-0.18	5.54E-01
Phvul.008G280600	PTHR12758:SF13 - GBF-INTERACTING PROTEIN 1 (1 of 2)	-0.28	2.64E-01	-0.36	6.13E-02	-0.20	5.05E-01	-0.39	4.39E-02
Phvul.001G147200	PTHR23500:SF112 - SUGAR TRANSPORTER ERD6-LIKE 7 (1 of 2)	-0.28	7.71E-01	-1.17	3.05E-02	-0.11	9.37E-01	-0.80	1.74E-01
Phvul.011G086300	PTHR10003:SF34 - SUPEROXIDE DISMUTASE [CU-ZN] 2, CHLOROPLASTIC (1 of 1)	-0.28	5.58E-01	-0.67	2.84E-02	-0.21	7.26E-01	-0.60	5.92E-02
Phvul.008G199400	no data	-0.28	7.07E-01	-0.98	2.36E-02	-0.20	8.32E-01	-0.77	9.72E-02
Phvul.003G158500	PTHR11177:SF219 - CHITINASE-LIKE PROTEIN-RELATED (1 of 1)	-0.28	5.65E-01	0.49	1.33E-01	0.16	8.09E-01	0.65	4.50E-02
Phvul.003G186600	PTHR14363:SF17 - HEPARANASE-LIKE PROTEIN 1-RELATED (1 of 2)	-0.28	6.15E-01	-0.69	6.84E-02	-0.70	1.51E-01	-1.03	6.56E-03
Phvul.009G258500	PTHR33222:SF9 - PROTEIN CURVATURE THYLAKOID 1B, CHLOROPLASTIC (1 of 2)	-0.28	6.80E-01	-1.14	3.70E-03	-0.25	7.56E-01	-0.85	4.32E-02
Phvul.005G154500	PF03514 - GRAS domain family (GRAS) (1 of 55)	-0.29	7.62E-01	0.76	2.41E-01	-0.59	5.11E-01	1.23	3.43E-02
Phvul.006G060600	K02949 - small subunit ribosomal protein S11e (RP-S11e, RPS11) (1 of 4)	-0.29	2.14E-01	-0.29	1.05E-01	-0.22	4.17E-01	-0.50	3.55E-03
Phvul.010G014000	5.4.99.8 - Cycloartenol synthase / 2,3-epoxysqualene--cycloartenol cyclase (1 of 3)	-0.29	7.13E-01	-1.04	1.97E-02	-0.18	8.56E-01	-0.78	1.03E-01
Phvul.007G077500	PF00560//PF08263//PF13855 - Leucine Rich Repeat (LRR 1) // Leucine rich repeat N-terminal domain (LRRNT 2) // Leuc	-0.29	6.11E-01	-0.78	2.93E-02	-0.38	4.99E-01	-0.40	3.08E-01
Phvul.010G118600	KOG0149 - Predicted RNA-binding protein SEB4 (RRM superfamily) (1 of 8)	-0.29	6.86E-01	-0.90	4.85E-02	-0.48	4.85E-01	-0.72	1.24E-01
Phvul.007G076200	K02940 - large subunit ribosomal protein L9e (RP-L9e, RPL9) (1 of 4)	-0.29	5.89E-01	-0.51	1.44E-01	-0.18	7.84E-01	-0.74	2.80E-02
Phvul.011G177600	PTHR23317 - DEDICATOR OF CYTOKINESIS DOCK (1 of 2)	-0.29	1.72E-01	-0.37	2.23E-02	-0.40	4.74E-02	-0.29	8.90E-02
Phvul.005G112200	no data	-0.29	6.46E-01	-1.13	1.01E-02	-0.27	7.09E-01	-1.24	6.06E-03
Phvul.010G146800	K18183 - cytochrome c oxidase assembly protein subunit 19 (COX19) (1 of 1)	-0.29	3.04E-01	-0.41	4.47E-02	-0.26	4.07E-01	-0.36	9.29E-02
Phvul.009G141700	K02976 - small subunit ribosomal protein S26e (RP-S26e, RPS26) (1 of 3)	-0.29	2.32E-01	-0.38	3.24E-02	-0.20	4.96E-01	-0.49	7.03E-03
Phvul.010G161000	K02149 - V-type H+-transporting ATPase subunit D (ATPeV1D, ATP6M) (1 of 1)	-0.29	3.01E-01	-0.48	1.55E-02	-0.20	5.63E-01	-0.40	5.75E-02
Phvul.009G061300	PTHR23324:SF43 - PHOSPHATIDYLINOSITOL/PHOSPHATIDYLCHOLINE TRANSFER PROTEIN SFH6-RELATED (1 of 6)	-0.29	3.33E-01	-0.50	1.95E-02	-0.32	3.19E-01	-0.43	5.39E-02
Phvul.008G227200	PTHR34953:SF1 - ALPHA/BETA HYDROLASE RELATED PROTEIN (1 of 2)	-0.29	5.19E-01	-0.71	1.29E-02	-0.35	4.44E-01	-0.64	3.44E-02
Phvul.011G212700	no data	-0.29	3.98E-01	-0.69	2.72E-03	-0.33	3.72E-01	-0.55	2.30E-02
Phvul.008G081200	PTHR31451:SF17 - MANNAN ENDO-1,4-BETA-MANNOSIDASE 6 (1 of 2)	-0.29	7.40E-01	-1.17	3.89E-02	-0.30	7.62E-01	-0.44	4.98E-01
Phvul.004G174100	PTHR27000:SF4 - EXTENSIN-LIKE PROTEIN-RELATED (1 of 2)	-0.29	7.52E-01	-1.14	3.04E-02	-0.21	8.51E-01	-0.58	3.15E-01
Phvul.011G160000	PTHR22904:SF374 - TPR REPEAT-CONTAINING THIOREDOXIN TTL1-RELATED (1 of 3)	-0.29	4.28E-01	-0.61	1.40E-02	-0.29	4.74E-01	-0.44	1.01E-01
Phvul.003G266600	PTHR13683:SF232 - ASPARTYL PROTEASE FAMILY PROTEIN (1 of 3)	-0.29	5.34E-01	-0.78	8.34E-03	-0.24	6.55E-01	-0.79	1.06E-02
Phvul.009G136100	PTHR10681//PTHR10681:SF91 - THIOREDOXIN PEROXIDASE // SUBFAMILY NOT NAMED (1 of 1)	-0.29	3.53E-01	-0.76	2.70E-04	-0.23	5.28E-01	-0.53	1.91E-02
Phvul.003G078400	no data	-0.29	3.74E-01	-0.79	4.81E-04	-0.23	5.60E-01	-0.71	2.40E-03
Phvul.001G259400	K00059 - 3-oxoacyl- (fabG) (1 of 2)	-0.29	3.44E-01	-0.57	7.88E-03	-0.25	4.71E-01	-0.66	2.54E-03
Phvul.002G283000	PTHR12565:SF90 - TRANSCRIPTION FACTOR BHLH137 (1 of 2)	-0.29	6.78E-01	-0.61	1.76E-01	-0.37	6.18E-01	-0.91	3.83E-02
Phvul.008G286600	PF03188//PF04526 - Eukaryotic cytochrome b561 (Cytochrom B561) // Protein of unknown function (DUF568) (DUF568	-0.30	6.39E-01	-0.94	5.06E-02	-0.23	7.54E-01	-1.05	3.26E-02
Phvul.004G172800	K02910 - large subunit ribosomal protein L31e (RP-L31e, RPL31) (1 of 3)	-0.30	3.62E-01	-0.40	8.25E-02	-0.19	6.37E-01	-0.54	1.95E-02
Phvul.008G046800	PTHR21450:SF9 - BZIP-LIKE PROTEIN-RELATED (1 of 2)	-0.30	3.25E-01	-0.53	1.15E-02	-0.28	4.04E-01	-0.45	4.23E-02
Phvul.006G162800	PTHR23244:SF308 - KELCH REPEAT-CONTAINING PROTEIN (1 of 2)	-0.30	6.29E-01	-1.10	2.12E-03	-0.47	4.21E-01	-1.18	1.42E-03
Phvul.009G236800	PTHR10795:SF431 - SUBTILISIN SERINE PROTEASE-RELATED (1 of 2)	-0.30	4.93E-01	-0.75	6.03E-03	-0.39	3.71E-01	-0.44	1.43E-01
Phvul.001G037500	KOG2922 - Uncharacterized conserved protein (1 of 11)	-0.30	4.52E-01	-0.68	9.59E-03	-0.30	4.83E-01	-0.51	6.50E-02
Phvul.002G074800	PTHR10177:SF206 - CYCLIN-D1-1 (1 of 2)	-0.30	5.99E-01	-0.85	1.18E-02	-0.37	5.22E-01	-0.73	4.31E-02
Phvul.004G002100	PTHR24078:SF279 - DUPLICATED SANT DNA-BINDING DOMAIN-CONTAINING PROTEIN (1 of 5)	-0.30	4.69E-01	-0.62	2.22E-02	-0.05	9.42E-01	-0.41	1.68E-01
Phvul.008G260700	K10257 - omega-3 fatty acid desaturase (delta-15 desaturase) (FAD8, desB) (1 of 4)	-0.30	7.08E-01	-1.33	2.88E-03	-0.34	6.93E-01	-1.40	2.60E-03
Phvul.004G000600	2.1.2.9 - Methionyl-tRNA formyltransferase / transformylase (1 of 1)	-0.30	1.23E-01	-0.31	5.07E-02	-0.38	4.66E-02	-0.31	6.13E-02
Phvul.010G102100	PTHR10811//PTHR10811:SF17 - FRINGE-RELATED // SUBFAMILY NOT NAMED (1 of 2)	-0.30	4.45E-01	-0.67	8.97E-03	-0.21	6.66E-01	-0.48	8.12E-02
Phvul.009G041000	PTHR31008//PTHR31008:SF5 - COP1-INTERACTING PROTEIN-RELATED // SUBFAMILY NOT NAMED (1 of 1)	-0.30	3.20E-01	-0.47	2.64E-02	-0.33	3.02E-01	-0.31	1.81E-01
Phvul.003G191100	PTHR12300:SF52 - HVA22-LIKE PROTEIN A-RELATED (1 of 2)	-0.30	4.45E-01	-0.74	5.03E-03	-0.42	2.80E-01	-0.55	5.38E-02
Phvul.003G126000	K13462 - guanine nucleotide-exchange factor (MIN7) (1 of 1)	-0.30	1.10E-01	-0.20	2.20E-01	-0.42	1.46E-02	-0.26	1.05E-01
Phvul.003G270900	PTHR18966//PTHR18966:SF245 - IONOTROPIC GLUTAMATE RECEPTOR // SUBFAMILY NOT NAMED (1 of 2)	-0.30	1.28E-01	-0.29	7.15E-02	-0.41	2.99E-02	-0.17	3.37E-01
Phvul.002G073100	KOG4510 - Permease of the drug/metabolite transporter (DMT) superfamily (1 of 4)	-0.30	5.04E-01	-1.18	2.23E-05	-0.17	7.78E-01	-1.04	2.62E-04
Phvul.005G111800	PF03514 - GRAS domain family (GRAS) (1 of 55)	-0.30	8.48E-01	-4.65	1.12E-05	-0.81	5.29E-01	-2.48	4.91E-03
Phvul.004G121000	1.14.11.20 - Deacetoxyvindoline 4-hydroxylase / Desacetyoxyvindoline-17-hydroxylase (1 of 14)	-0.30	5.72E-01	-0.77	2.09E-02	-0.24	7.02E-01	-0.40	2.64E-01
Phvul.011G021800	1.8.7.1 - Assimilatory sulfite reductase (ferredoxin) / Sulfite reductase (ferredoxin) (1 of 1)	-0.30	3.35E-01	-0.40	7.50E-02	-0.37	2.45E-01	-0.52	2.18E-02
Phvul.002G107000	PTHR31356:SF8 - L-ASCORBATE PEROXIDASE 5, PEROXISOMAL (1 of 2)	-0.30	6.16E-01	-0.91	1.82E-02	-0.31	6.37E-01	-0.68	9.39E-02
Phvul.008G230600	PTHR31300:SF2 - EMB (1 of 2)	-0.30	4.43E-01	-0.57	3.02E-02	-0.25	5.80E-01	-0.45	1.06E-01
Phvul.007G236800	PTHR14155//PTHR14155:SF115 - RING FINGER DOMAIN-CONTAINING // SUBFAMILY NOT NAMED (1 of 1)	-0.30	7.74E-01	-1.31	2.44E-02	-0.26	8.33E-01	-0.81	1.99E-01
Phvul.001G116500	no data	-0.30	4.66E-01	-0.75	5.03E-03	-0.10	8.71E-01	-0.51	7.66E-02
Phvul.001G024200	K10395 - kinesin family member 4/21/27 (KIF4 21 27) (1 of 5)	-0.30	4.71E-01	-0.84	2.37E-03	-0.34	4.47E-01	-0.61	3.67E-02
Phvul.001G021200	PTHR13832:SF25 - PROTEIN PHOSPHATASE 2C 12-RELATED (1 of 2)	-0.30	6.20E-01	-0.82	3.14E-02	-0.28	6.94E-01	-0.42	3.13E-01
Phvul.002G182700	PTHR13794 - ENOLASE SUPERFAMILY, MANDELATE RACEMASE (1 of 1)	-0.30	3.27E-01	-0.37	1.01E-01	-0.28	4.16E-01	-0.47	3.91E-02
Phvul.002G280600	1.1.1.208 - (+)-neomenthol dehydrogenase / Monoterpenoid dehydrogenase (1 of 1)	-0.30	6.67E-01	-1.16	4.49E-03	-0.42	5.46E-01	-1.08	1.15E-02
Phvul.002G174200	K02880 - large subunit ribosomal protein L17e (RP-L17e, RPL17) (1 of 3)	-0.30	1.97E-01	-0.29	1.18E-01	-0.17	5.79E-01	-0.40	3.16E-02
Phvul.L003800	K02922 - large subunit ribosomal protein L37e (RP-L37e, RPL37) (1 of 4)	-0.30	4.29E-01	-0.40	1.49E-01	-0.21	6.53E-01	-0.77	3.19E-03
Phvul.005G067000	PTHR22904:SF374 - TPR REPEAT-CONTAINING THIOREDOXIN TTL1-RELATED (1 of 3)	-0.31	5.60E-01	-0.85	8.00E-03	-0.26	6.64E-01	-0.56	1.09E-01
Phvul.009G117900	PTHR31942:SF13 - MLO-LIKE PROTEIN 15 (1 of 1)	-0.31	2.90E-01	-0.44	3.40E-02	-0.30	3.46E-01	-0.41	6.24E-02
Phvul.003G277500	PTHR21562:SF4 - PECTINACETYLESTERASE FAMILY PROTEIN (1 of 3)	-0.31	6.32E-01	-0.98	9.10E-03	-0.26	7.30E-01	-0.65	1.10E-01
Phvul.005G034800	1.1.1.195 - Cinnamyl-alcohol dehydrogenase / CAD (1 of 20)	-0.31	2.28E-01	-0.44	1.90E-02	-0.24	4.12E-01	-0.33	9.56E-02
Phvul.007G264100	K02979 - small subunit ribosomal protein S28e (RP-S28e, RPS28) (1 of 2)	-0.31	4.38E-01	-0.53	4.84E-02	-0.13	8.15E-01	-0.70	9.45E-03
Phvul.002G065200	K02977 - small subunit ribosomal protein S27Ae (RP-S27Ae, RPS27A) (1 of 2)	-0.31	3.43E-01	-0.39	9.33E-02	-0.12	7.95E-01	-0.56	1.60E-02
Phvul.008G047700	PTHR10795//PTHR10795:SF365 - PROPROTEIN CONVERTASE SUBTILISIN/KEXIN // SUBFAMILY NOT NAMED (1 of 4)	-0.31	7.14E-01	-1.44	2.53E-03	-0.40	6.47E-01	-0.96	5.93E-02
Phvul.008G238600	PTHR27001:SF164 - RECEPTOR PROTEIN KINASE TMK1-RELATED (1 of 3)	-0.31	6.86E-01	-0.91	4.32E-02	-0.03	9.85E-01	-0.51	3.12E-01
Phvul.011G212400	K09272 - structure-specific recognition protein 1 (SSRP1) (1 of 2)	-0.31	6.20E-01	-0.75	5.73E-02	-0.18	8.21E-01	-0.89	2.80E-02
Phvul.002G307800	K05759 - profilin (PFN) (1 of 5)	-0.31	7.64E-01	-2.03	6.29E-04	-0.37	7.41E-01	-1.32	3.14E-02
Phvul.001G151100	PTHR15615:SF20 - CYCLIN FAMILY PROTEIN-RELATED (1 of 3)	-0.31	2.48E-01	-0.42	3.41E-02	-0.35	2.06E-01	-0.32	1.28E-01
Phvul.004G092600	K08912 - light-harvesting complex II chlorophyll a/b binding protein 1 (LHCB1) (1 of 13)	-0.31	7.72E-01	-1.51	9.27E-03	-0.19	8.90E-01	-1.01	1.05E-01
Phvul.004G017000	PTHR10641:SF469 - TRANSCRIPTION FACTOR MYB21-RELATED (1 of 3)	-0.31	4.61E-01	-0.62	2.51E-02	-0.27	5.65E-01	-0.49	9.39E-02
Phvul.007G064900	5.4.2.5 - Phosphoglucomutase (glucose-cofactor) / Glucose-1-phosphate phosphotransferase (1 of 1)	-0.31	3.94E-01	-0.70	3.62E-03	-0.27	5.22E-01	-0.49	6.07E-02
Phvul.010G111700	PTHR32285:SF9 - PROTEIN TRICHOME BIREFRINGENCE-LIKE 25-RELATED (1 of 2)	-0.31	5.77E-01	-0.81	2.06E-02	-0.32	5.94E-01	-0.57	1.29E-01
Phvul.007G208200	PTHR21562:SF5 - PECTINACETYLESTERASE FAMILY PROTEIN (1 of 6)	-0.31	5.37E-01	-1.05	8.48E-04	-0.37	4.76E-01	-0.79	1.72E-02
Phvul.011G148100	PTHR11071:SF174 - PEPTIDYL-PROLYL CIS-TRANS ISOMERASE CYP37, CHLOROPLASTIC (1 of 1)	-0.31	3.43E-01	-0.72	1.12E-03	-0.16	7.20E-01	-0.49	4.30E-02
Phvul.007G005400	PF04755 - PAP fibrillin (PAP fibrillin) (1 of 12)	-0.31	3.65E-01	-0.78	6.14E-04	-0.27	4.92E-01	-0.58	1.78E-02
Phvul.011G117400	2.1.1.14 - 5-methyltetrahydropteroyltriglutamate--homocysteine S-methyltransferase / Tetrahydropteroylglutamate-ho	-0.31	4.61E-01	-0.63	2.68E-02	-0.25	6.11E-01	-0.61	3.80E-02
Phvul.003G292600	PTHR36035:SF1 - PROTEIN DISULFIDE-ISOMERASE SCO2 (1 of 1)	-0.31	4.91E-01	-0.91	1.72E-03	-0.17	7.73E-01	-0.88	3.34E-03
Phvul.004G070900	no data	-0.31	2.46E-01	-0.44	2.66E-02	-0.23	4.76E-01	-0.45	2.91E-02
Phvul.009G018200	PTHR22835:SF184 - GDSL ESTERASE/LIPASE EXL1-RELATED (1 of 8)	-0.31	3.68E-01	-0.50	5.26E-02	-0.53	9.77E-02	-0.56	3.35E-02
Phvul.008G000300	2.7.8.5 - CDP-diacylglycerol--glycerol-3-phosphate 3-phosphatidyltransferase / Phosphatidylglycerophosphate synthase	-0.31	2.83E-01	-0.12	6.81E-01	-0.13	7.58E-01	-0.46	4.40E-02
Phvul.007G199100	PTHR13832:SF158 - PROTEIN PHOSPHATASE 2C 5-RELATED (1 of 3)	-0.31	2.57E-01	-0.43	3.80E-02	-0.12	7.58E-01	-0.24	2.83E-01
Phvul.011G094500	PTHR32227:SF70 - GLUCAN ENDO-1,3-BETA-GLUCOSIDASE 14 (1 of 2)	-0.31	4.68E-01	-0.57	7.18E-02	-0.24	6.44E-01	-0.68	3.11E-02
Phvul.002G264200	PTHR11064//PTHR11064:SF45 - CCAAT-BINDING TRANSCRIPTION FACTOR-RELATED // SUBFAMILY NOT NAMED (1 of 6)	-0.31	6.66E-01	-1.01	1.80E-02	-0.19	8.33E-01	-0.63	1.81E-01
Phvul.003G241400	2.7.11.1 - Non-specific serine/threonine protein kinase / Threonine-specific protein kinase (1 of 1198)	-0.32	5.38E-01	-0.68	3.82E-02	-0.24	6.96E-01	-0.76	2.47E-02
Phvul.003G191200	K02997 - small subunit ribosomal protein S9e (RP-S9e, RPS9) (1 of 4)	-0.32	4.09E-01	-0.39	1.58E-01	-0.15	7.85E-01	-0.63	1.76E-02
Phvul.002G057900	PF10250 - GDP-fucose protein O-fucosyltransferase (O-FucT) (1 of 39)	-0.32	2.10E-01	-0.37	6.64E-02	-0.20	5.13E-01	-0.49	1.47E-02
Phvul.005G000700	PF02362 - B3 DNA binding domain (B3) (1 of 72)	-0.32	7.62E-01	-1.11	3.28E-02	0.11	9.40E-01	-0.95	7.28E-02
Phvul.006G177300	PTHR31828:SF1 - PHOSPHOLIPASE A1-IIALPHA-RELATED (1 of 3)	-0.32	7.68E-01	-1.66	3.20E-02	-0.24	8.49E-01	-0.62	4.38E-01
Phvul.010G065800	PF04859 - Plant protein of unknown function (DUF641) (DUF641) (1 of 8)	-0.32	7.36E-01	-1.48	4.67E-03	-0.60	4.89E-01	-1.04	6.71E-02
Phvul.009G067100	K10395 - kinesin family member 4/21/27 (KIF4 21 27) (1 of 5)	-0.32	3.96E-01	-0.56	2.95E-02	-0.36	3.52E-01	-0.33	2.45E-01
Phvul.001G229200	PTHR13683:SF327 - ASPARTYL PROTEASE FAMILY PROTEIN (1 of 2)	-0.32	4.80E-01	-0.69	1.80E-02	-0.10	8.86E-01	-0.54	8.02E-02

Phvul.011G061600	PF07714//PF13414 - Protein tyrosine kinase (Pkinase Tyr) // TPR repeat (TPR 11) (1 of 1)	-0.32	5.23E-01	-0.83	7.57E-03	-0.12	8.68E-01	-0.61	7.01E-02
Phvul.006G073400	PTHR32227:SF28 - GLUCAN ENDO-1,3-BETA-GLUCOSIDASE 9 (1 of 1)	-0.32	2.90E-01	-0.30	1.94E-01	0.04	9.38E-01	-0.44	5.00E-02
Phvul.005G168800	PTHR10744:SF7 - 30S RIBOSOMAL PROTEIN S17, CHLOROPLASTIC (1 of 1)	-0.32	5.99E-01	-0.76	4.31E-02	-0.02	9.90E-01	-0.54	1.84E-01
Phvul.002G250300	PF00314 - Thaumatin family (Thaumatin) (1 of 26)	-0.32	8.36E-01	-1.34	1.67E-01	-1.01	4.36E-01	-2.91	6.06E-03
Phvul.009G170500	PTHR31683:SF28 - PECTATE LYASE 22-RELATED (1 of 2)	-0.32	6.10E-01	-0.48	2.59E-01	-0.24	7.48E-01	-0.85	3.54E-02
Phvul.007G099200	K16803 - cytoskeleton-associated protein 5 (CKAP5, XMAP215) (1 of 1)	-0.32	7.67E-02	-0.21	1.87E-01	-0.37	3.96E-02	-0.08	6.58E-01
Phvul.007G143200	5.3.99.9 - Neoxanthin synthase / NSY (1 of 4)	-0.32	3.94E-01	-0.84	1.21E-03	-0.47	1.96E-01	-0.63	2.07E-02
Phvul.010G048100	PF02298 - Plastocyanin-like domain (Cu bind like) (1 of 53)	-0.32	4.93E-01	-0.88	2.81E-03	-0.17	7.86E-01	-0.70	2.65E-02
Phvul.003G205600	PTHR23115//PTHR23115:SF146 - TRANSLATION FACTOR // SUBFAMILY NOT NAMED (1 of 2)	-0.32	5.59E-01	-0.84	1.37E-02	-0.17	8.23E-01	-0.66	6.85E-02
Phvul.002G273300	no data	-0.32	3.40E-01	-0.61	9.15E-03	-0.34	3.43E-01	-0.52	3.38E-02
Phvul.007G000500	no data	-0.32	5.29E-01	-0.99	2.01E-03	-0.37	4.84E-01	-0.88	8.79E-03
Phvul.002G251100	K07437 - cytochrome P450, family 26, subfamily A (CYP26A) (1 of 4)	-0.32	5.06E-01	-0.69	3.33E-02	-0.28	6.08E-01	-0.56	1.03E-01
Phvul.006G125900	PTHR32448:SF34 - F22M8.11 PROTEIN-RELATED (1 of 7)	-0.32	3.59E-01	-1.02	1.05E-05	-0.39	2.88E-01	-0.87	2.39E-04
Phvul.001G224100	PF01789 - PsbP (PsbP) (1 of 12)	-0.32	4.97E-01	-0.98	9.71E-04	-0.22	7.11E-01	-0.79	1.24E-02
Phvul.011G051300	KOG0313//KOG0315 - Microtubule binding protein YTM1 (contains WD40 repeats) // G-protein beta subunit-like protei	-0.32	5.35E-01	-0.79	1.65E-02	-0.18	7.91E-01	-0.58	1.03E-01
Phvul.010G030800	PTHR22594:SF36 - ASPARAGINE--TRNA LIGASE, CYTOPLASMIC 2 (1 of 1)	-0.32	4.98E-01	-0.98	1.45E-02	-0.58	1.98E-01	-0.59	1.49E-01
Phvul.003G009600	K00895 - pyrophosphate--fructose-6-phosphate 1-phosphotransferase (E2.7.1.90, pfk) (1 of 4)	-0.32	3.71E-01	-0.51	4.46E-02	-0.38	3.08E-01	-0.64	1.31E-02
Phvul.002G188500	K11714 - rhamnogalacturonan II specific xylosyltransferase [EC:2.4.2.-] Xyl a1-3 Fuc (RGXT) (1 of 1)	-0.32	3.97E-01	-0.56	3.92E-02	-0.41	3.01E-01	-0.83	2.16E-03
Phvul.003G117800	PTHR13533:SF11 - O-ACETYLTRANSFERASE CAS1P-LIKE PROTEIN-RELATED (1 of 2)	-0.32	4.28E-01	-0.60	3.24E-02	-0.38	3.62E-01	-0.56	5.50E-02
Phvul.011G162800	K02968 - small subunit ribosomal protein S20 (RP-S20, rpsT) (1 of 1)	-0.32	2.76E-01	-0.71	6.11E-04	-0.23	5.22E-01	-0.52	1.97E-02
Phvul.007G266000	PTHR31358:SF10 - TARGETING PROTEIN FOR XKLP2-LIKE PROTEIN (1 of 5)	-0.32	4.37E-01	-0.59	3.59E-02	-0.25	6.22E-01	-0.39	2.06E-01
Phvul.001G053100	KOG0865//KOG0879 - Cyclophilin type peptidyl-prolyl cis-trans isomerase // U-snRNP-associated cyclophilin type peptid	-0.32	7.12E-01	-1.05	3.86E-02	-0.25	8.10E-01	-0.62	2.69E-01
Phvul.003G097003	K05573 - NAD(P)H-quinone oxidoreductase subunit 2 (ndhB) (1 of 3)	-0.32	5.49E-01	-0.49	1.93E-01	0.11	8.90E-01	-0.85	1.95E-02
Phvul.005G031800	no data	-0.32	5.76E-01	0.80	1.32E-02	-0.07	9.40E-01	0.03	9.49E-01
Phvul.002G080000	PF13947//PF14380 - Wall-associated receptor kinase galacturonan-binding (GUB WAK bind) // Wall-associated recepto	-0.32	4.88E-01	-0.82	1.00E-02	-0.34	5.00E-01	-0.77	1.89E-02
Phvul.002G093100	no data	-0.33	5.25E-01	-0.39	2.96E-01	-0.37	4.88E-01	-0.71	3.92E-02
Phvul.007G207200	K16911 - ATP-dependent RNA helicase DDX21 [EC:3.6.4.13] (DDX21) (1 of 3)	-0.33	3.49E-01	-0.52	3.27E-02	-0.27	4.99E-01	-0.45	7.88E-02
Phvul.002G246500	PTHR24326:SF191 - HOMEOBOX-LEUCINE ZIPPER PROTEIN ATHB-14-RELATED (1 of 2)	-0.33	7.07E-01	-1.34	1.77E-02	-0.20	8.51E-01	-0.66	2.66E-01
Phvul.003G114300	PTHR23118//PTHR23118:SF3 - ATP-CITRATE SYNTHASE // SUBFAMILY NOT NAMED (1 of 2)	-0.33	3.89E-01	-0.66	1.04E-02	-0.26	5.58E-01	-0.46	9.88E-02
Phvul.001G043100	PTHR12373 - ENHANCER OF RUDIMENTARY ERH (1 of 1)	-0.33	6.08E-01	-0.87	3.12E-02	-0.59	3.20E-01	-0.46	3.08E-01
Phvul.011G211500	PTHR13301//PTHR13301:SF79 - X-BOX TRANSCRIPTION FACTOR-RELATED // SUBFAMILY NOT NAMED (1 of 1)	-0.33	3.32E-01	-0.49	4.19E-02	-0.24	5.47E-01	-0.10	7.42E-01
Phvul.002G312604	PTHR34282:SF2 - GENOMIC DNA, CHROMOSOME 3, P1 CLONE:MSD24 (1 of 2)	-0.33	4.46E-01	-0.43	1.58E-01	-0.35	4.44E-01	-0.69	2.04E-02
Phvul.002G035700	K14826 - FK506-binding nuclear protein [EC:5.2.1.8] (FPR3 4) (1 of 2)	-0.33	4.38E-01	-0.47	1.17E-01	-0.43	3.09E-01	-0.72	1.52E-02
Phvul.008G259500	PTHR31342:SF9 - F10K1.18 PROTEIN (1 of 1)	-0.33	5.99E-01	-0.91	3.67E-02	-0.20	7.99E-01	-0.19	7.22E-01
Phvul.009G020200	PTHR18896//PTHR18896:SF72 - PHOSPHOLIPASE D // SUBFAMILY NOT NAMED (1 of 1)	-0.33	5.21E-01	-0.89	1.18E-02	-0.28	6.28E-01	-0.36	3.71E-01
Phvul.006G087000	PTHR13980 - CDC68 RELATED (1 of 2)	-0.33	7.51E-01	-1.49	3.02E-02	-0.40	7.23E-01	-0.52	4.69E-01
Phvul.009G033200	PTHR10357:SF74 - ISOAMYLASE 3, CHLOROPLASTIC (1 of 1)	-0.33	2.39E-01	-0.54	7.17E-03	-0.39	1.80E-01	-0.63	2.35E-03
Phvul.004G108200	K16900 - two pore calcium channel protein, plant (TPC1, CCH1) (1 of 1)	-0.33	7.34E-01	-0.81	2.44E-01	-0.44	6.61E-01	-1.44	3.92E-02
Phvul.003G128266	K03564 - peroxiredoxin Q/BCP (BCP, PRXQ, DOT5) (1 of 1)	-0.33	5.59E-01	-0.87	1.28E-02	-0.22	7.54E-01	-0.71	5.54E-02
Phvul.001G123200	PF07058 - Microtubule-associated protein 70 (MAP70) (1 of 7)	-0.33	3.88E-01	-0.79	2.02E-03	-0.37	3.64E-01	-0.79	3.10E-03
Phvul.009G117700	PTHR11229:SF5 - 50S RIBOSOMAL PROTEIN L3-1, CHLOROPLASTIC (1 of 1)	-0.33	3.62E-01	-0.71	3.96E-03	-0.20	6.72E-01	-0.62	1.62E-02
Phvul.005G120700	PTHR24362:SF258 - SERINE/THREONINE-PROTEIN KINASE NEK5 (1 of 4)	-0.33	3.20E-01	-0.55	2.10E-02	-0.36	3.15E-01	-0.47	5.84E-02
Phvul.008G119600	PF06232 - Embryo-specific protein 3, (ATS3) (ATS3) (1 of 5)	-0.33	3.97E-01	-0.75	4.06E-03	-0.20	6.91E-01	-0.47	1.02E-01
Phvul.002G047700	PF11250 - Fantastic Four meristem regulator (FAF) (1 of 11)	-0.33	4.81E-01	-0.89	3.98E-03	0.08	9.11E-01	-0.57	7.98E-02
Phvul.008G241700	1.5.3.16//1.5.3.17 - Spermine oxidase / SMO // Non-specific polyamine oxidase / Polyamine oxidase (1 of 2)	-0.33	4.72E-01	-0.71	2.00E-02	-0.39	4.22E-01	-0.54	9.99E-02
Phvul.003G257500	PTHR24089:SF321 - GM03767P (1 of 1)	-0.33	3.97E-01	-0.45	1.25E-01	-0.30	5.03E-01	-0.62	3.28E-02
Phvul.004G127500	K01689 - enolase (ENO, eno) (1 of 3)	-0.34	4.84E-01	-0.91	3.74E-03	-0.35	5.00E-01	-0.66	4.93E-02
Phvul.007G014500	2.7.7.2 - FAD synthetase / FMN adenylyltransferase (1 of 2)	-0.34	1.56E-01	-0.26	1.95E-01	-0.41	9.12E-02	-0.44	2.05E-02
Phvul.001G159200	PTHR10459:SF60 - POLY [ADP-RIBOSE] POLYMERASE 1 (1 of 1)	-0.34	3.82E-01	-0.67	1.57E-02	-0.36	3.87E-01	-0.54	6.39E-02
Phvul.006G115700	PTHR10891:SF624 - CALCIUM-BINDING PROTEIN KIC (1 of 2)	-0.34	5.40E-01	-0.74	3.39E-02	-0.18	8.12E-01	-0.50	1.90E-01
Phvul.002G017700	2.7.11.1 - Non-specific serine/threonine protein kinase / Threonine-specific protein kinase (1 of 1198)	-0.34	6.94E-01	-1.21	1.40E-02	-0.16	8.85E-01	-0.88	9.34E-02
Phvul.009G206800	PTHR10994 - RETICULON (1 of 23)	-0.34	1.69E-01	-0.39	4.67E-02	-0.32	2.43E-01	-0.10	6.73E-01
Phvul.003G244700	PTHR10265:SF25 - CYCLIN-DEPENDENT KINASE INHIBITOR 6 (1 of 2)	-0.34	6.60E-01	-1.01	2.67E-02	-0.13	9.04E-01	-0.80	9.70E-02
Phvul.009G248600	K10393 - kinesin family member 2/24 (KIF2 24, MCAK) (1 of 5)	-0.34	2.75E-01	-0.64	3.44E-03	-0.27	4.58E-01	-0.48	3.91E-02
Phvul.009G121100	K02952 - small subunit ribosomal protein S13 (RP-S13, rpsM) (1 of 2)	-0.34	2.30E-01	-0.78	8.44E-05	-0.18	6.45E-01	-0.56	8.67E-03
Phvul.009G043600	PF00560//PF07714//PF08263//PF13855 - Leucine Rich Repeat (LRR 1) // Protein tyrosine kinase (Pkinase Tyr) // Leucin	-0.34	3.37E-01	-0.50	4.87E-02	-0.23	6.04E-01	-0.35	1.98E-01
Phvul.006G197600	no data	-0.34	2.85E-01	-0.60	7.49E-03	-0.24	5.23E-01	-0.44	6.49E-02
Phvul.003G241700	PTHR31729:SF0 - ETHYLENE-RESPONSIVE TRANSCRIPTION FACTOR ERF008-RELATED (1 of 4)	-0.34	4.94E-01	-0.65	4.84E-02	-0.20	7.54E-01	-0.63	6.26E-02
Phvul.003G130000	PF07911 - Protein of unknown function (DUF1677) (DUF1677) (1 of 10)	-0.34	4.87E-01	-0.87	3.74E-03	0.13	8.45E-01	-0.74	1.76E-02
Phvul.002G162400	PF00069//PF00560//PF08263 - Protein kinase domain (Pkinase) // Leucine Rich Repeat (LRR 1) // Leucine rich repeat N	-0.34	6.96E-01	-1.69	1.87E-03	-0.37	7.04E-01	-1.14	4.10E-02
Phvul.007G186700	PTHR21654:SF22 - TRIHELIX TRANSCRIPTION FACTOR GTL2 (1 of 2)	-0.34	5.68E-01	-1.03	4.85E-03	-0.30	6.64E-01	-0.71	6.95E-02
Phvul.006G007380	K16285 - RING/U-box domain-containing protein (XERICO) (1 of 3)	-0.34	1.68E-01	-0.59	1.15E-03	-0.25	3.95E-01	-0.49	1.16E-02
Phvul.008G032800	PTHR10693//PTHR10693:SF25 - RAS GTPASE-ACTIVATING PROTEIN-BINDING PROTEIN // SUBFAMILY NOT NAMED (1 of	-0.34	5.65E-01	-0.86	3.68E-02	-0.37	5.63E-01	-0.53	2.24E-01
Phvul.009G184900	no data	-0.34	7.72E-01	1.41	2.56E-02	-0.69	5.44E-01	1.11	1.09E-01
Phvul.004G158000	PF00139 - Legume lectin domain (Lectin legB) (1 of 53)	-0.34	6.12E-01	-0.65	1.36E-01	0.03	9.81E-01	-0.90	3.40E-02
Phvul.009G048400	PTHR12231:SF112 - RHODANESE-LIKE DOMAIN-CONTAINING PROTEIN 14, CHLOROPLASTIC (1 of 2)	-0.34	5.59E-01	-1.07	2.71E-03	-0.46	4.25E-01	-0.93	1.36E-02
Phvul.009G072200	PTHR24206:SF35 - TRANSCRIPTION FACTOR-RELATED (1 of 2)	-0.34	3.04E-01	-0.96	2.24E-05	-0.43	2.05E-01	-0.59	1.63E-02
Phvul.005G070200	K00852 - ribokinase (rbsK, RBKS) (1 of 2)	-0.34	4.50E-01	-0.66	3.67E-02	-0.24	6.71E-01	-0.61	6.09E-02
Phvul.002G328804	PTHR16166//PTHR16166:SF87 - VACUOLAR PROTEIN SORTING-ASSOCIATED PROTEIN VPS13 // SUBFAMILY NOT NAMED	-0.34	4.35E-01	-0.83	3.59E-03	-0.08	9.08E-01	-0.43	1.84E-01
Phvul.009G153200	K11097 - small nuclear ribonucleoprotein E (SNRPE, SME) (1 of 2)	-0.34	5.20E-01	-0.62	8.65E-02	-0.44	4.13E-01	-0.73	4.49E-02
Phvul.003G213400	1.1.1.133 - dTDP-4-dehydrorhamnose reductase / dTDP-6-deoxy-L-mannose dehydrogenase (1 of 2)	-0.34	2.90E-01	-0.49	3.73E-02	-0.16	7.31E-01	-0.40	1.13E-01
Phvul.009G203100	PTHR31673:SF3 - COBRA-LIKE EXTRACELLULAR GLYCOSYL-PHOSPHATIDYL INOSITOL-ANCHORED FAMILY PROTEIN-RELAT	-0.34	6.24E-01	-1.17	4.66E-03	-0.46	5.09E-01	-1.05	1.58E-02
Phvul.001G225800	PTHR21726:SF29 - F1O19.10 PROTEIN (1 of 4)	-0.35	3.78E-01	-0.62	2.32E-02	-0.40	3.28E-01	-0.57	4.42E-02
Phvul.006G216700	PTHR19370:SF120 - FERREDOXIN-1, CHLOROPLASTIC-RELATED (1 of 3)	-0.35	7.39E-01	-1.60	5.31E-03	-0.26	8.33E-01	-1.21	4.67E-02
Phvul.002G332700	PTHR24058:SF23 - SERINE/THREONINE KINASE-RELATED (1 of 2)	-0.35	3.06E-01	-0.55	2.96E-02	-0.33	3.82E-01	-0.41	1.32E-01
Phvul.009G256900	no data	-0.35	2.58E-01	-0.46	3.96E-02	-0.06	9.16E-01	-0.06	8.55E-01
Phvul.001G184400	PF01535//PF13041//PF14432 - PPR repeat (PPR) // PPR repeat family (PPR 2) // DYW family of nucleic acid deaminases	-0.35	2.94E-01	-0.54	2.50E-02	-0.22	5.85E-01	-0.63	1.13E-02
Phvul.009G004801	PTHR11850:SF65 - BEL1-LIKE HOMEODOMAIN PROTEIN 6-RELATED (1 of 1)	-0.35	6.41E-01	-0.92	4.55E-02	-0.09	9.41E-01	-0.66	1.81E-01
Phvul.006G146000	K02926 - large subunit ribosomal protein L4 (RP-L4, MRPL4, rplD) (1 of 3)	-0.35	4.69E-01	-0.99	2.53E-03	-0.66	1.29E-01	-1.13	7.51E-04
Phvul.001G221300	PTHR11654//PTHR11654:SF110 - OLIGOPEPTIDE TRANSPORTER-RELATED // SUBFAMILY NOT NAMED (1 of 2)	-0.35	5.48E-01	-0.77	3.63E-02	-0.36	5.60E-01	-0.41	3.12E-01
Phvul.001G144600	PTHR24115:SF70 - KINESIN-RELATED PROTEIN 11 (1 of 2)	-0.35	2.69E-01	-0.70	1.68E-03	-0.55	5.32E-02	-0.59	1.22E-02
Phvul.002G287900	K11883 - RNA-binding protein NOB1 (NOB1) (1 of 1)	-0.35	1.86E-01	-0.46	2.28E-02	-0.51	3.71E-02	-0.35	1.03E-01
Phvul.004G033800	PTHR31348:SF2 - EID1-LIKE F-BOX PROTEIN 1-RELATED (1 of 3)	-0.35	3.37E-01	-0.49	6.29E-02	-0.38	3.19E-01	-0.64	1.58E-02
Phvul.008G082900	K02356 - elongation factor P (efp) (1 of 1)	-0.35	4.90E-01	-0.67	4.61E-02	-0.20	7.55E-01	-0.48	1.84E-01
Phvul.001G173500	PTHR11260//PTHR11260:SF222 - GLUTATHIONE S-TRANSFERASE, GST, SUPERFAMILY, GST DOMAIN CONTAINING // SUE	-0.35	1.50E-01	-0.49	7.50E-03	-0.43	7.06E-02	-0.50	9.52E-03
Phvul.008G249300	PTHR11766 - TYROSYL-TRNA SYNTHETASE (1 of 1)	-0.35	4.22E-01	-0.57	5.98E-02	-0.25	6.29E-01	-0.72	1.91E-02
Phvul.002G027100	PTHR30546:SF3 - MINOR ALLERGEN (1 of 1)	-0.35	2.95E-01	-0.61	1.45E-02	-0.25	5.24E-01	-0.52	4.69E-02
Phvul.006G135100	PTHR31314:SF5 - MYB-LIKE HTH TRANSCRIPTIONAL REGULATOR FAMILY PROTEIN-RELATED (1 of 1)	-0.35	4.26E-01	-0.75	9.58E-03	-0.38	4.20E-01	-0.63	3.88E-02
Phvul.003G204100	K02900 - large subunit ribosomal protein L27Ae (RP-L27Ae, RPL27A) (1 of 3)	-0.35	3.90E-01	-0.45	1.28E-01	-0.21	6.91E-01	-0.70	1.38E-02
Phvul.007G243900	K18460 - exportin-7 (XPO7, EXP7) (1 of 2)	-0.35	4.73E-01	-0.76	1.73E-02	-0.27	6.38E-01	-0.76	2.15E-02
Phvul.004G116200	PTHR11655:SF14 - 54S RIBOSOMAL PROTEIN L6, MITOCHONDRIAL (1 of 1)	-0.35	3.71E-01	-0.78	2.71E-03	-0.18	7.37E-01	-0.54	5.63E-02
Phvul.001G094500	PTHR11689:SF67 - CHLORIDE CHANNEL PROTEIN CLC-A-RELATED (1 of 2)	-0.35	2.36E-01	-0.64	2.42E-03	-0.18	6.51E-01	-0.40	8.61E-02
Phvul.009G155600	KOG0866 - Ran-binding protein RANBP3 (1 of 1)	-0.35	1.95E-01	-0.47	2.08E-02	-0.28	3.81E-01	-0.35	1.06E-01
Phvul.008G213300	no data	-0.35	4.25E-01	-0.59	4.94E-02	-0.37	4.31E-01	-0.40	2.16E-01
Phvul.002G302900	PF00582//PF07714 - Universal stress protein family (Usp) // Protein tyrosine kinase (Pkinase Tyr) (1 of 3)	-0.35	1.64E-01	-0.52	6.74E-03	-0.27	3.58E-01	-0.52	9.43E-03
Phvul.001G002600	PTHR13675:SF0 - LYR MOTIF-CONTAINING PROTEIN 2 (1 of 2)	-0.35	4.63E-01	-0.41	2.29E-01	-0.35	5.12E-01	-0.80	1.52E-02
Phvul.002G054900	PTHR11132//PTHR11132:SF132 - SOLUTE CARRIER FAMILY 35 // SUBFAMILY NOT NAMED (1 of 1)	-0.35	1.07E-01	-0.41	2.07E-02	-0.30	2.33E-01	-0.32	8.65E-02
Phvul.004G014200	PTHR32212:SF126 - EMB (1 of 24)	-0.35	3.77E-01	-0.61	2.63E-02	-0.53	1.62E-01	-0.71	1.18E-02
Phvul.003G075700	3.2.1.45 - Glucosylceramidase / D-glucosyl-N-acylsphingosine glucohydrolase (1 of 2)	-0.35	1.49E-01	-0.52	7.43E-03	-0.01	9.85E-01	-0.08	7.51E-01
Phvul.003G116800	PTHR34188:SF1 - T6A9.7 PROTEIN (1 of 3)	-0.35	5.02E-01	-0.75	3.41E-02	-0.31	5.97E-01	-0.31	4.41E-01
Phvul.007G108200	PTHR31165:SF13 - PROTEIN LIGHT-DEPENDENT SHORT HYPOCOTYLS 1-RELATED (1 of 2)	-0.35	5.60E-01	0.81	5.54E-02	-0.44	4.85E-01	0.85	4.67E-02
Phvul.002G035500	PTHR10992:SF833 - HYDROLASE, ALPHA/BETA FOLD FAMILY PROTEIN (1 of 1)	-0.35	4.83E-01	-0.84	9.92E-03	-0.25	6.89E-01	-0.51	1.58E-01
Phvul.006G059284	PF00069//PF08263//PF13855 - Protein kinase domain (Pkinase) // Leucine rich repeat N-terminal domain (LRRNT 2) //	-0.35	6.01E-01	-0.92	2.50E-02	-0.40	5.77E-01	-0.42	3.85E-01

Phvul.002G290400	1.1.1.330 - Very-long-chain 3-oxoacyl-CoA reductase / Very-long-chain beta-ketoacyl-CoA reductase (1 of 18)	-0.35	6.61E-01	-1.13	1.78E-02	-0.19	8.58E-01	-0.58	2.76E-01
Phvul.003G008100	PTHR10593//PTHR10593:SF50 - SERINE/THREONINE-PROTEIN KINASE RIO // SUBFAMILY NOT NAMED (1 of 3)	-0.35	3.73E-01	-0.77	3.63E-03	-0.38	3.58E-01	-0.66	1.91E-02
Phvul.002G227200	PTHR10639:SF7 - CLATHRIN LIGHT CHAIN (1 of 2)	-0.35	2.80E-01	-0.53	2.63E-02	-0.34	3.51E-01	-0.36	1.63E-01
Phvul.003G273100	PTHR10629:SF34 - DNA (CYTOSINE-5)-METHYLTRANSFERASE CMT2 (1 of 1)	-0.35	2.34E-01	-0.61	7.54E-03	-0.56	3.98E-02	-0.24	3.68E-01
Phvul.001G002800	PTHR24349:SF89 - CALCIUM-DEPENDENT PROTEIN KINASE 29 (1 of 5)	-0.35	3.41E-01	-0.64	3.05E-02	-0.26	5.53E-01	-0.24	4.68E-01
Phvul.002G289700	PF10157 - Uncharacterized conserved protein (DUF2365) (DUF2365) (1 of 1)	-0.35	3.56E-01	-0.74	4.92E-03	-0.45	2.45E-01	-0.71	1.00E-02
Phvul.001G036200	1.14.19.4 - Delta(8)-fatty-acid desaturase / SLD (1 of 3)	-0.36	7.04E-01	-1.69	1.48E-03	-0.45	6.48E-01	-1.36	1.44E-02
Phvul.009G143200	PTHR10177:SF213 - CYCLIN-D4-1-RELATED (1 of 2)	-0.36	5.52E-01	-1.00	9.88E-03	-0.53	3.63E-01	-0.80	4.86E-02
Phvul.008G068700	K00028 - malate dehydrogenase (decarboxylating) (E1.1.1.39) (1 of 3)	-0.36	6.48E-01	-0.99	3.23E-02	-0.52	4.94E-01	-0.56	2.73E-01
Phvul.007G117800	K08916 - light-harvesting complex II chlorophyll a/b binding protein 5 (LHCB5) (1 of 2)	-0.36	1.60E-01	-0.42	3.40E-02	-0.36	2.01E-01	-0.42	4.07E-02
Phvul.002G324900	K03032 - 26S proteasome regulatory subunit N2 (PSMD1, RPN2) (1 of 1)	-0.36	6.37E-01	-1.08	1.61E-02	-0.31	7.21E-01	-0.81	9.20E-02
Phvul.008G068100	K17497 - phosphomannomutase (PMM) (1 of 1)	-0.36	3.21E-01	-0.82	1.12E-03	-0.40	3.01E-01	-0.76	3.30E-03
Phvul.007G158200	K11252 - histone H2B (H2B) (1 of 10)	-0.36	3.43E-01	-0.54	4.40E-02	-0.43	2.75E-01	-0.74	5.77E-03
Phvul.002G206800	K15920 - beta-D-xylosidase 4 (XYL4) (1 of 2)	-0.36	5.48E-01	-0.82	4.87E-02	-0.51	3.84E-01	-0.50	2.60E-01
Phvul.008G144200	no data	-0.36	6.56E-01	-1.36	6.17E-03	-0.51	5.13E-01	-0.89	8.65E-02
Phvul.009G020400	4.1.1.17 - Ornithine decarboxylase / L-ornithine carboxy-lyase (1 of 2)	-0.36	7.26E-01	-1.45	1.28E-02	-0.08	9.59E-01	-0.82	1.99E-01
Phvul.002G034300	PF00076//PF00642 - RNA recognition motif. (a.k.a. RRM, RBD, or RNP domain) (RRM 1) // Zinc finger C-x8-C-x5-C-x3-H t	-0.36	2.03E-01	-0.72	1.05E-03	-0.45	1.19E-01	-0.74	1.02E-03
Phvul.006G185000	PTHR27007:SF9 - L-TYPE LECTIN-DOMAIN CONTAINING RECEPTOR KINASE VII.1-RELATED (1 of 4)	-0.36	8.26E-01	1.39	5.13E-02	0.36	8.34E-01	1.43	4.99E-02
Phvul.011G019100	PTHR23160 - MYOSIN HEAVY CHAIN-RELATED (1 of 5)	-0.36	3.10E-01	-0.58	2.09E-02	-0.40	2.86E-01	-0.34	2.22E-01
Phvul.006G176900	K02914 - large subunit ribosomal protein L34 (RP-L34, MRPL34, rpmH) (1 of 3)	-0.36	5.70E-01	-0.85	3.88E-02	-0.42	5.22E-01	-1.19	4.73E-03
Phvul.002G247500	K17800 - LETM1 and EF-hand domain-containing protein 1, mitochondrial (LETM1, MDM38) (1 of 2)	-0.36	4.44E-01	-0.70	2.43E-02	-0.27	6.35E-01	-0.51	1.24E-01
Phvul.006G172900	PTHR11669:SF16 - PROTEIN STICHEL-LIKE 3-RELATED (1 of 1)	-0.36	4.87E-02	-0.19	2.90E-01	-0.30	1.51E-01	0.01	9.73E-01
Phvul.006G115500	no data	-0.36	7.19E-01	-1.49	9.01E-03	-0.38	7.36E-01	-1.24	3.87E-02
Phvul.001G219400	PTHR11071:SF247 - PEPTIDYL-PROLYL CIS-TRANS ISOMERASE D (1 of 1)	-0.36	3.14E-01	-0.72	3.25E-03	-0.30	4.60E-01	-0.56	3.43E-02
Phvul.001G012200	K10572 - inositol-pentakisphosphate 2-kinase (IPPK) (1 of 2)	-0.36	6.63E-02	-0.42	1.07E-02	-0.31	1.71E-01	-0.21	2.64E-01
Phvul.010G125400	PF02519 - Auxin responsive protein (Auxin inducible) (1 of 85)	-0.36	6.75E-01	-0.93	7.57E-02	-0.18	8.70E-01	-1.17	2.67E-02
Phvul.005G164800	PTHR22835//PTHR22835:SF170 - ZINC FINGER FYVE DOMAIN CONTAINING PROTEIN // SUBFAMILY NOT NAMED (1 of 2)	-0.36	2.83E-01	-0.52	3.28E-02	-0.25	5.35E-01	-0.14	6.43E-01
Phvul.005G090100	PF00954//PF07714//PF08276 - S-locus glycoprotein domain (S locus glycop) // Protein tyrosine kinase (Pkinase Tyr) // I	-0.36	1.16E-01	-0.69	7.27E-05	-0.21	4.89E-01	-0.44	1.79E-02
Phvul.002G085600	K02915 - large subunit ribosomal protein L34e (RP-L34e, RPL34) (1 of 3)	-0.36	3.51E-01	-0.59	2.86E-02	-0.27	5.52E-01	-0.77	4.52E-03
Phvul.001G239500	PTHR11132//PTHR11132:SF104 - SOLUTE CARRIER FAMILY 35 // SUBFAMILY NOT NAMED (1 of 4)	-0.36	2.77E-01	-0.49	4.48E-02	-0.50	1.26E-01	-0.64	9.44E-03
Phvul.007G080500	K02964 - small subunit ribosomal protein S18e (RP-S18e, RPS18) (1 of 2)	-0.36	2.96E-01	-0.48	5.63E-02	-0.18	7.04E-01	-0.62	1.41E-02
Phvul.010G127700	PTHR21229:SF15 - LUNG SEVEN TRANSMEMBRANE RECEPTOR-LIKE PROTEIN (1 of 1)	-0.36	4.56E-01	-0.73	2.47E-02	-0.37	4.76E-01	-0.63	6.56E-02
Phvul.006G057700	K11416 - mono-ADP-ribosyltransferase sirtuin 6 (SIRT6, SIR2L6) (1 of 1)	-0.36	2.58E-01	-0.49	4.39E-02	-0.22	5.81E-01	-0.45	6.96E-02
Phvul.007G032600	PTHR10165:SF80 - LIPID PHOSPHATE PHOSPHATASE 1-RELATED (1 of 4)	-0.36	2.55E-01	-0.68	3.05E-03	-0.35	3.25E-01	-0.60	1.24E-02
Phvul.001G252300	no data	-0.36	3.29E-01	-0.92	2.35E-04	-0.46	2.21E-01	-0.90	4.83E-04
Phvul.002G141200	PF05678 - VQ motif (VQ) (1 of 39)	-0.36	6.70E-01	-1.11	2.80E-02	-0.25	8.15E-01	-0.72	1.92E-01
Phvul.003G241500	K10395 - kinesin family member 4/21/27 (KIF4 21 27) (1 of 5)	-0.36	1.55E-01	-0.50	1.11E-02	-0.37	1.90E-01	-0.27	2.21E-01
Phvul.001G195500	PF07714//PF08263//PF13855 - Protein tyrosine kinase (Pkinase Tyr) // Leucine rich repeat N-terminal domain (LRRNT 2	-0.36	4.38E-01	-0.80	9.17E-03	-0.33	5.30E-01	-0.61	6.39E-02
Phvul.002G041800	PF08879//PF08880 - WRC (WRC) // QLQ (QLQ) (1 of 10)	-0.36	7.36E-01	-1.45	1.74E-02	-0.64	5.23E-01	-1.24	5.22E-02
Phvul.002G308600	PTHR19443//PTHR19443:SF26 - HEXOKINASE // SUBFAMILY NOT NAMED (1 of 3)	-0.36	2.95E-01	-0.71	4.05E-03	-0.19	6.89E-01	-0.45	8.99E-02
Phvul.001G161900	PTHR10108:SF906 - METHYLTRANSFERASE PMT1-RELATED (1 of 4)	-0.36	2.70E-01	-0.54	2.30E-02	-0.33	3.77E-01	-0.53	3.38E-02
Phvul.002G015700	3.1.3.16//3.1.3.48//3.1.3.67 - Protein-serine/threonine phosphatase / Serine/threonine specific protein phosphatase //	-0.36	1.78E-01	-0.61	3.44E-03	-0.27	4.11E-01	-0.31	1.84E-01
Phvul.006G037600	PF12796//PF13962 - Ankyrin repeats (3 copies) (Ank 2) // Domain of unknown function (PGG) (1 of 27)	-0.36	2.33E-01	-0.70	1.40E-03	-0.21	6.08E-01	-0.49	3.63E-02
Phvul.002G304100	3.6.1.9 - Nucleotide diphosphatase / Nucleotide pyrophosphatase (1 of 2)	-0.36	4.55E-01	-0.73	3.08E-02	-0.33	5.39E-01	-0.56	1.17E-01
Phvul.004G176300	PTHR19359//PTHR19359:SF10 - CYTOCHROME B5 // SUBFAMILY NOT NAMED (1 of 2)	-0.36	6.86E-01	-1.08	5.62E-02	-0.47	6.23E-01	-1.59	4.85E-03
Phvul.008G020100	no data	-0.36	8.05E-01	-2.06	9.36E-03	-0.68	6.27E-01	-1.77	3.20E-02
Phvul.009G006500	PTHR18966//PTHR18966:SF186 - IONOTROPIC GLUTAMATE RECEPTOR // SUBFAMILY NOT NAMED (1 of 1)	-0.37	4.35E-01	-0.74	2.16E-02	-0.43	3.81E-01	-0.23	5.56E-01
Phvul.008G087000	PF00234 - Protease inhibitor/seed storage/LTP family (Tryp alpha amyl) (1 of 24)	-0.37	8.14E-01	-1.98	1.47E-02	-0.49	7.59E-01	-1.75	4.02E-02
Phvul.003G189200	PF01844 - HNH endonuclease (HNH) (1 of 4)	-0.37	4.39E-01	-0.68	3.84E-02	-0.10	8.92E-01	-0.53	1.23E-01
Phvul.001G111700	2.1.2.2 - Phosphoribosylglycinamide formyltransferase / Glycinamide ribonucleotide transformylase (1 of 1)	-0.37	2.20E-01	-0.42	6.81E-02	-0.58	3.30E-02	-0.36	1.32E-01
Phvul.002G232600	no data	-0.37	5.54E-01	-1.06	6.68E-03	-0.24	7.57E-01	-0.56	1.95E-01
Phvul.002G256700	PTHR15139 - TUBULIN FOLDING COFACTOR C (1 of 1)	-0.37	3.04E-01	-0.80	1.21E-03	-0.14	7.99E-01	-0.61	1.95E-02
Phvul.003G027600	K09287 - RAV-like factor (RAV) (1 of 3)	-0.37	5.94E-01	-1.16	6.55E-03	-0.43	5.52E-01	-1.14	1.01E-02
Phvul.007G223700	PTHR10015:SF164 - HEAT STRESS TRANSCRIPTION FACTOR A-3 (1 of 1)	-0.37	2.47E-01	-0.54	2.36E-02	-0.26	5.06E-01	-0.47	6.13E-02
Phvul.009G031900	PTHR10108:SF899 - PECTIN METHYLTRANSFERASE QUA2-RELATED (1 of 2)	-0.37	2.38E-01	-0.48	4.48E-02	-0.37	2.90E-01	-0.41	1.01E-01
Phvul.005G058900	K06892 - feruloyl-CoA ortho-hydroxylase (F6H1) (1 of 3)	-0.37	4.43E-01	-0.69	4.76E-02	-0.14	8.33E-01	-0.63	7.55E-02
Phvul.006G192500	PTHR23315:SF56 - ARMADILLO/BETA-CATENIN-LIKE REPEAT-CONTAINING PROTEIN (1 of 2)	-0.37	4.25E-01	-0.83	7.02E-03	-0.20	7.49E-01	-0.45	1.93E-01
Phvul.003G190900	PF03634 - TCP family transcription factor (TCP) (1 of 27)	-0.37	2.34E-01	-0.61	7.03E-03	-0.31	3.93E-01	-0.42	8.54E-02
Phvul.002G297000	PF03106 - WRKY DNA -binding domain (WRKY) (1 of 91)	-0.37	2.13E-01	-0.49	2.83E-02	-0.18	6.47E-01	-0.46	5.34E-02
Phvul.003G193000	PF04982 - HPP family (HPP) (1 of 1)	-0.37	3.53E-01	-0.73	7.40E-03	-0.31	4.98E-01	-0.69	1.55E-02
Phvul.008G269200	no data	-0.37	7.95E-01	-1.64	4.83E-02	-0.53	7.23E-01	-1.25	1.46E-01
Phvul.008G233600	PTHR10108:SF803 - METHYLTRANSFERASE PMT10-RELATED (1 of 1)	-0.37	1.60E-01	-0.44	3.48E-02	-0.42	1.32E-01	-0.42	5.48E-02
Phvul.002G308000	PTHR32227:SF88 - GLUCAN ENDO-1,3-BETA-GLUCOSIDASE 12-RELATED (1 of 1)	-0.37	7.25E-01	-1.36	3.86E-02	-0.18	9.02E-01	-1.05	1.21E-01
Phvul.002G296400	K10395 - kinesin family member 4/21/27 (KIF4 21 27) (1 of 5)	-0.37	2.50E-01	-0.52	3.31E-02	-0.26	5.03E-01	-0.37	1.67E-01
Phvul.004G093000	no data	-0.37	7.27E-01	-1.35	2.91E-02	0.01	9.97E-01	-0.57	4.18E-01
Phvul.004G121800	PTHR12329:SF11 - BAG FAMILY MOLECULAR CHAPERONE REGULATOR 2-RELATED (1 of 2)	-0.37	2.64E-01	-0.51	4.02E-02	-0.21	6.41E-01	-0.24	4.08E-01
Phvul.008G157000	PTHR33144:SF3 - PLANT TRANSPOSASE (PTTA/EN/SPM FAMILY) (1 of 7)	-0.38	4.04E-01	-0.77	1.13E-02	-0.20	7.39E-01	-0.50	1.34E-01
Phvul.005G044700	6.3.4.2 - CTP synthase (glutamine hydrolyzing) / UTP--ammonia ligase (1 of 4)	-0.38	4.62E-01	-0.75	2.73E-02	-0.30	6.22E-01	-0.82	1.90E-02
Phvul.010G089700	K09338 - homeobox-leucine zipper protein (HD-ZIP) (1 of 50)	-0.38	6.06E-01	-0.97	3.05E-02	-0.31	7.21E-01	-0.70	1.45E-01
Phvul.001G201000	K02889 - large subunit ribosomal protein L21e (RP-L21e, RPL21) (1 of 2)	-0.38	2.95E-01	-0.48	6.99E-02	-0.17	7.46E-01	-0.57	3.30E-02
Phvul.010G012900	no data	-0.38	6.05E-01	-0.92	3.93E-02	-0.50	4.82E-01	-0.49	3.26E-01
Phvul.008G021001	PTHR27001:SF132 - PROLINE-RICH RECEPTOR-LIKE PROTEIN KINASE PERK8 (1 of 2)	-0.38	3.53E-01	-0.85	2.51E-03	-0.24	6.38E-01	-0.52	9.04E-02
Phvul.001G212000	PTHR22904:SF7 - T14P4.7 PROTEIN (1 of 2)	-0.38	6.60E-01	-1.14	2.66E-02	-0.30	7.71E-01	-0.67	2.37E-01
Phvul.001G107400	PTHR24411:SF35 - BTB/POZ AND MATH DOMAIN-CONTAINING PROTEIN 1-RELATED (1 of 4)	-0.38	6.57E-01	-1.55	1.07E-02	-0.22	8.35E-01	-0.45	4.89E-01
Phvul.009G013400	PTHR13233:SF0 - MICROSPHERULE PROTEIN 1 (1 of 1)	-0.38	4.33E-01	-0.65	4.67E-02	-0.48	3.26E-01	-0.51	1.48E-01
Phvul.001G231800	2.7.11.1 - Non-specific serine/threonine protein kinase / Threonine-specific protein kinase (1 of 1198)	-0.38	6.68E-01	-1.15	4.90E-02	-0.22	8.46E-01	-1.13	5.93E-02
Phvul.002G104700	PTHR31356:SF8 - L-ASCORBATE PEROXIDASE 5, PEROXISOMAL (1 of 2)	-0.38	4.52E-01	-0.95	6.89E-03	-0.39	4.76E-01	-0.65	7.66E-02
Phvul.007G063600	PTHR31718:SF8 - EMBRYO-SPECIFIC PROTEIN 3 (1 of 3)	-0.38	2.94E-01	-0.55	4.70E-02	-0.60	7.29E-02	-0.53	5.87E-02
Phvul.001G010900	PTHR23421:SF51 - BETA-GALACTOSIDASE 5 (1 of 2)	-0.38	5.70E-01	-1.35	9.07E-04	-0.44	5.29E-01	-0.90	3.82E-02
Phvul.011G098200	PTHR24362:SF258 - SERINE/THREONINE-PROTEIN KINASE NEK5 (1 of 4)	-0.38	3.62E-01	-0.63	3.33E-02	-0.46	2.89E-01	-0.24	4.92E-01
Phvul.006G150500	PTHR12298//PTHR12298:SF22 - PCDC2 PROGRAMMED CELL DEATH PROTEIN 2 -RELATED // SUBFAMILY NOT NAMED (1	-0.38	2.58E-01	-0.54	4.04E-02	-0.39	2.86E-01	-0.66	1.39E-02
Phvul.003G201600	no data	-0.38	4.03E-01	-0.92	4.62E-03	-0.19	7.62E-01	-0.77	2.37E-02
Phvul.005G038900	K02934 - large subunit ribosomal protein L6e (RP-L6e, RPL6) (1 of 3)	-0.38	2.62E-01	-0.36	1.79E-01	-0.24	5.90E-01	-0.58	2.46E-02
Phvul.003G131600	PTHR22893:SF67 - 12-OXOPHYTODIENOATE REDUCTASE 3 (1 of 2)	-0.39	2.89E-01	-0.58	2.82E-02	-0.19	7.10E-01	-0.25	4.15E-01
Phvul.010G092800	3.4.22.69 - SARS coronavirus main proteinase / Severe acute respiratory syndrome coronavirus main protease (1 of 6)	-0.39	1.67E-01	-0.35	1.25E-01	-0.46	1.03E-01	-0.47	3.50E-02
Phvul.002G058200	PF00892 - EamA-like transporter family (EamA) (1 of 74)	-0.39	3.89E-01	-0.98	1.15E-03	-0.40	4.08E-01	-0.87	6.16E-03
Phvul.006G155800	PTHR20852:SF53 - GLUTAMINE SYNTHETASE, CHLOROPLASTIC/MITOCHONDRIAL (1 of 1)	-0.39	1.69E-01	-0.67	1.17E-03	-0.21	5.85E-01	-0.43	5.83E-02
Phvul.009G254900	1.8.1.4 - Dihydrolipoyl dehydrogenase / Lipoyl dehydrogenase (1 of 3)	-0.39	1.86E-01	-0.60	6.41E-03	-0.31	3.74E-01	-0.63	5.75E-03
Phvul.006G125100	PTHR32448:SF25 - FAD-BINDING BERBERINE FAMILY PROTEIN-RELATED (1 of 5)	-0.39	3.39E-01	-0.71	1.50E-02	-0.48	2.50E-01	-0.57	6.27E-02
Phvul.L008743	PTHR31390:SF4 - GB (1 of 1)	-0.39	3.51E-01	-0.61	5.00E-02	-0.37	4.25E-01	-0.39	2.48E-01
Phvul.008G231000	PTHR32175:SF2 - SURFEIT LOCUS PROTEIN 2 (1 of 1)	-0.39	1.24E-01	-0.39	5.71E-02	-0.33	2.60E-01	-0.44	3.43E-02
Phvul.003G216800	PTHR11132//PTHR11132:SF65 - SOLUTE CARRIER FAMILY 35 // SUBFAMILY NOT NAMED (1 of 1)	-0.39	5.50E-01	-0.93	2.28E-02	-0.18	8.35E-01	-0.71	1.01E-01
Phvul.008G034800	PTHR19317:SF9 - PRA1 FAMILY PROTEIN B1-RELATED (1 of 4)	-0.39	4.88E-01	-0.80	2.93E-02	-0.21	7.83E-01	-0.99	7.79E-03
Phvul.009G118900	PTHR19139:SF152 - AQUAPORIN PIP2-7 (1 of 1)	-0.39	6.37E-01	-1.01	4.37E-02	-0.04	9.81E-01	-0.68	2.11E-01
Phvul.003G286200	PTHR31734:SF24 - AUXIN-RESPONSIVE PROTEIN IAA9 (1 of 2)	-0.39	3.49E-01	-0.99	3.95E-04	-0.30	5.44E-01	-0.58	5.84E-02
Phvul.005G159400	K15403 - fatty acid omega-hydroxy dehydrogenase (ACE, HTH) (1 of 2)	-0.39	5.80E-01	-1.30	2.51E-03	-0.28	7.45E-01	-1.00	2.91E-02
Phvul.007G041400	K15923 - alpha-L-fucosidase 2 (AXY8, FUC95A, afcA) (1 of 2)	-0.39	5.33E-01	-1.14	4.10E-03	-0.50	4.29E-01	-0.93	2.74E-02
Phvul.004G034500	K00012 - UDPglucose 6-dehydrogenase (UGDH, ugd) (1 of 4)	-0.39	3.10E-01	-0.64	1.92E-02	-0.31	4.86E-01	-0.54	6.19E-02
Phvul.005G124100	PTHR22952//PTHR22952:SF179 - CAMP-RESPONSE ELEMENT BINDING PROTEIN-RELATED // SUBFAMILY NOT NAMED (1	-0.39	3.53E-01	0.74	1.09E-02	-0.04	9.59E-01	0.34	3.22E-01
Phvul.009G221700	K02881 - large subunit ribosomal protein L18 (RP-L18, MRPL18, rplR) (1 of 3)	-0.39	2.97E-01	-0.83	1.33E-03	-0.20	7.02E-01	-0.60	3.04E-02
Phvul.009G056800	PTHR10593:SF31 - C2H2-LIKE ZINC FINGER PROTEIN-RELATED (1 of 2)	-0.39	4.56E-01	-0.75	4.08E-02	-0.23	7.34E-01	-0.52	1.80E-01
Phvul.002G086700	PTHR12300:SF51 - HVA22-LIKE PROTEIN C (1 of 2)	-0.39	5.77E-01	-1.03	1.99E-02	-0.16	8.70E-01	-0.84	7.18E-02
Phvul.011G057000	K01188 - beta-glucosidase (E3.2.1.21) (1 of 34)	-0.39	4.20E-01	-0.69	3.71E-02	-0.41	4.30E-01	-0.63	6.73E-02

Phvul.002G294304	PTHR24349:SF95 - CALCIUM-DEPENDENT PROTEIN KINASE 3 (1 of 2)	-0.39	5.56E-01	-0.87	4.36E-02	-0.43	5.48E-01	-0.82	6.42E-02
Phvul.009G140500	PF00069//PF00560//PF08263 - Protein kinase domain (Pkinase) // Leucine Rich Repeat (LRR 1) // Leucine rich repeat N	-0.39	3.90E-02	-0.17	3.87E-01	-0.32	1.54E-01	-0.05	8.21E-01
Phvul.006G163600	K02955 - small subunit ribosomal protein S14e (RP-S14e, RPS14) (1 of 3)	-0.39	1.93E-01	-0.33	1.89E-01	-0.30	4.04E-01	-0.58	1.34E-02
Phvul.011G148900	1.3.1.33 - Protochlorophyllide reductase / Protochlorophyllide oxidoreductase (1 of 2)	-0.39	8.39E-01	-2.55	1.11E-02	-0.59	7.62E-01	-2.12	3.82E-02
Phvul.002G308700	1.1.1.60 - 2-hydroxy-3-oxopropionate reductase / Tartronate semialdehyde reductase (1 of 2)	-0.40	4.38E-01	-0.86	1.05E-02	-0.44	4.07E-01	-0.65	7.13E-02
Phvul.011G060600	no data	-0.40	6.16E-01	-1.19	4.84E-02	-0.65	4.03E-01	-0.91	1.31E-01
Phvul.009G209200	PF00069//PF08263//PF13855 - Protein kinase domain (Pkinase) // Leucine rich repeat N-terminal domain (LRRNT 2) //	-0.40	1.41E-01	0.15	5.66E-01	-0.53	4.56E-02	0.01	9.68E-01
Phvul.003G230100	PTHR31747:SF1 - PROTEIN LOL1 (1 of 2)	-0.40	4.48E-01	-1.02	2.30E-03	-0.46	4.00E-01	-1.16	7.71E-04
Phvul.006G012700	PTHR10857:SF46 - DCD (DEVELOPMENT AND CELL DEATH) DOMAIN PROTEIN (1 of 4)	-0.40	4.48E-01	-0.86	1.51E-02	-0.44	4.25E-01	-1.11	2.54E-03
Phvul.005G122600	PTHR33083:SF5 - POLLEN OLE E 1 ALLERGEN AND EXTENSIN FAMILY PROTEIN (1 of 2)	-0.40	7.37E-01	-1.50	4.19E-02	-0.64	5.67E-01	-1.41	5.78E-02
Phvul.008G058600	PTHR27002:SF122 - CYSTEINE-RICH RECEPTOR-LIKE PROTEIN KINASE 28-RELATED (1 of 17)	-0.40	3.77E-01	-0.70	2.90E-02	-0.25	6.60E-01	-0.61	6.65E-02
Phvul.007G208700	PTHR10742:SF294 - PROTOPORPHYRINOGEN OXIDASE 1, CHLOROPLASTIC (1 of 1)	-0.40	8.60E-02	-0.61	7.90E-04	-0.33	2.28E-01	-0.44	2.39E-02
Phvul.009G053000	PTHR31707:SF8 - PECTINESTERASE/PECTINESTERASE INHIBITOR 34-RELATED (1 of 2)	-0.40	6.84E-01	-1.15	4.84E-02	-0.07	9.68E-01	-0.76	2.23E-01
Phvul.008G090200	1.1.1.271 - GDP-L-fucose synthase / GDP-fucose synthetase (1 of 1)	-0.40	4.33E-02	-0.31	7.81E-02	-0.19	4.85E-01	-0.22	2.37E-01
Phvul.008G059400	PTHR27001:SF156 - PROTEIN KINASE FAMILY PROTEIN (1 of 3)	-0.40	4.95E-01	-0.95	1.01E-02	-0.25	7.35E-01	-0.73	6.56E-02
Phvul.002G285700	PTHR31221:SF25 - WRKY TRANSCRIPTION FACTOR 45-RELATED (1 of 2)	-0.40	3.73E-01	-0.79	8.77E-03	-0.24	6.72E-01	-0.73	2.08E-02
Phvul.011G172300	PTHR31942:SF15 - MLO-LIKE PROTEIN 11-RELATED (1 of 2)	-0.40	3.90E-01	-0.67	4.89E-02	-0.57	2.11E-01	-0.44	2.27E-01
Phvul.008G005900	5.3.99.9 - Neoxanthin synthase / NSY (1 of 4)	-0.40	4.99E-01	-0.97	1.08E-02	-0.47	4.30E-01	-0.83	3.81E-02
Phvul.010G041700	PTHR10772:SF13 - CHLOROPLAST CHAPERONIN 10 (1 of 2)	-0.40	2.74E-01	-0.62	1.86E-02	-0.31	4.75E-01	-0.48	8.38E-02
Phvul.001G167900	PTHR13140:SF256 - MYOSIN-14-RELATED (1 of 4)	-0.40	2.90E-01	-0.56	4.46E-02	-0.39	3.46E-01	-0.41	1.68E-01
Phvul.001G226800	PTHR13832:SF158 - PROTEIN PHOSPHATASE 2C 5-RELATED (1 of 3)	-0.40	5.56E-01	-1.25	2.75E-03	-0.43	5.55E-01	-1.35	1.79E-03
Phvul.007G198500	PF13414 - TPR repeat (TPR 11) (1 of 76)	-0.40	4.07E-01	-0.92	4.26E-03	-0.36	5.14E-01	-0.77	2.30E-02
Phvul.009G098000	PTHR31407:SF7 - PSBP DOMAIN-CONTAINING PROTEIN 5, CHLOROPLASTIC (1 of 1)	-0.40	1.13E-01	-0.66	8.29E-04	-0.34	2.53E-01	-0.59	4.15E-03
Phvul.001G038800	PTHR27007:SF24 - L-TYPE LECTIN-DOMAIN CONTAINING RECEPTOR KINASE IX.1-RELATED (1 of 16)	-0.40	2.37E-01	-0.60	1.55E-02	-0.19	6.88E-01	-0.37	1.80E-01
Phvul.001G096900	K16223 - protein FLOWERING LOCUS T (FT) (1 of 3)	-0.40	3.35E-01	-0.77	7.74E-03	-0.30	5.52E-01	-0.67	2.76E-02
Phvul.007G195300	K08504 - blocked early in transport 1 (BET1) (1 of 3)	-0.40	4.59E-01	-0.87	1.87E-02	-0.51	3.52E-01	-0.63	1.10E-01
Phvul.010G162400	PF02298 - Plastocyanin-like domain (Cu bind like) (1 of 53)	-0.40	5.28E-01	-1.00	1.40E-02	-0.20	8.15E-01	-0.92	3.30E-02
Phvul.006G078500	PTHR11945:SF183 - AGAMOUS-LIKE MADS-BOX PROTEIN AGL80-RELATED (1 of 8)	-0.40	4.88E-01	-1.14	3.08E-03	-0.38	5.56E-01	-0.53	2.14E-01
Phvul.L009243	K02603 - origin recognition complex subunit 1 (ORC1) (1 of 1)	-0.40	5.05E-01	-0.24	6.51E-01	-0.58	3.23E-01	-0.89	4.10E-02
Phvul.003G219732	no data	-0.40	5.53E-01	0.73	1.02E-01	-0.37	6.30E-01	0.93	3.63E-02
Phvul.006G107800	PTHR22835//PTHR22835:SF171 - ZINC FINGER FYVE DOMAIN CONTAINING PROTEIN // SUBFAMILY NOT NAMED (1 of 2)	-0.40	5.11E-01	-0.85	3.11E-02	-0.42	5.20E-01	-0.75	7.26E-02
Phvul.007G100900	PTHR19241:SF303 - ABC TRANSPORTER G FAMILY MEMBER 21 (1 of 2)	-0.41	3.98E-02	-0.19	3.30E-01	-0.18	5.14E-01	0.08	7.20E-01
Phvul.005G034000	PTHR13878:SF53 - CYTOKININ DEHYDROGENASE 6 (1 of 3)	-0.41	7.59E-01	-1.68	2.51E-02	-0.21	9.05E-01	-1.40	7.37E-02
Phvul.011G095750	no data	-0.41	7.19E-01	-1.58	1.16E-02	-0.26	8.52E-01	-1.55	1.68E-02
Phvul.008G281900	2.4.2.9//2.7.1.48 - Uracil phosphoribosyltransferase / UMP pyrophosphorylase // Uridine kinase / Uridine monophospho	-0.41	1.68E-01	-0.66	5.12E-03	-0.43	1.71E-01	-0.63	9.79E-03
Phvul.003G227800	K07893 - Ras-related protein Rab-6A (RAB6A) (1 of 4)	-0.41	3.98E-01	-0.92	5.04E-03	-0.42	4.14E-01	-0.84	1.53E-02
Phvul.001G194100	K03386 - peroxiredoxin (alkyl hydroperoxide reductase subunit C) [EC:1.11.1.15] (E1.11.1.15, PRDX, ahpC) (1 of 2)	-0.41	2.45E-01	-0.70	5.03E-03	-0.32	4.29E-01	-0.70	7.39E-03
Phvul.004G028200	PTHR22939:SF99 - PROTEASE DO-LIKE 8, CHLOROPLASTIC (1 of 1)	-0.41	1.08E-01	-0.72	2.71E-04	-0.36	2.24E-01	-0.58	5.34E-03
Phvul.009G189500	PTHR32295:SF14 - PROTEIN IQ-DOMAIN 31-RELATED (1 of 4)	-0.41	4.23E-01	-0.72	3.64E-02	-0.28	6.51E-01	-0.55	1.35E-01
Phvul.002G024700	no data	-0.41	1.07E-01	-0.51	1.29E-02	-0.38	1.86E-01	-0.39	7.28E-02
Phvul.002G134500	no data	-0.41	6.15E-01	-1.16	1.97E-02	-0.36	7.03E-01	-1.04	4.58E-02
Phvul.002G146700	PTHR11119:SF54 - NUCLEOBASE-ASCORBATE TRANSPORTER 11 (1 of 2)	-0.41	2.88E-01	-1.05	3.45E-04	-0.66	5.86E-02	-0.76	1.29E-02
Phvul.002G079400	PTHR12565//PTHR12565:SF101 - STEROL REGULATORY ELEMENT-BINDING PROTEIN // SUBFAMILY NOT NAMED (1 of 1)	-0.41	2.21E-01	-0.85	5.28E-04	-0.28	4.88E-01	-0.70	6.20E-03
Phvul.002G237400	PTHR24055:SF196 - MITOGEN-ACTIVATED PROTEIN KINASE 14-RELATED (1 of 1)	-0.41	3.88E-01	-0.67	3.96E-02	-0.17	8.08E-01	-0.57	9.99E-02
Phvul.011G095700	PTHR36029:SF1 - PROTEIN TPLATE (1 of 1)	-0.41	2.00E-01	-0.71	2.59E-03	-0.28	4.67E-01	-0.53	3.46E-02
Phvul.009G201300	K10401 - kinesin family member 18/19 (KIF18 19) (1 of 3)	-0.41	4.13E-01	-1.61	5.00E-06	-0.37	5.08E-01	-0.67	6.17E-02
Phvul.007G252800	PTHR31362:SF1 - EXPRESSED PROTEIN (1 of 1)	-0.41	3.32E-02	-0.44	8.79E-03	-0.29	2.29E-01	-0.25	1.75E-01
Phvul.011G107700	PTHR10811//PTHR10811:SF25 - FRINGE-RELATED // SUBFAMILY NOT NAMED (1 of 1)	-0.41	2.74E-01	-1.17	5.51E-06	-0.30	5.12E-01	-0.89	9.75E-04
Phvul.008G261500	K07300 - Ca2+:H+ antiporter (chaA, CAX) (1 of 6)	-0.41	4.46E-01	-0.69	6.43E-02	-0.27	6.95E-01	-0.76	4.90E-02
Phvul.007G011800	PTHR11946:SF89 - METHIONYL-TRNA SYNTHETASE (1 of 1)	-0.41	9.87E-02	-0.72	2.29E-04	-0.28	3.74E-01	-0.52	1.21E-02
Phvul.003G245900	1.1.1.102 - 3-dehydrosphinganine reductase / KTS reductase (1 of 3)	-0.41	3.81E-01	-0.68	3.71E-02	-0.20	7.54E-01	-0.57	9.58E-02
Phvul.008G093000	2.7.10.2//2.7.11.1 - Non-specific protein-tyrosine kinase / Cytoplasmic protein tyrosine kinase // Non-specific serine/thr	-0.41	1.72E-01	-0.59	9.95E-03	-0.15	7.47E-01	-0.49	4.40E-02
Phvul.003G035400	no data	-0.41	5.69E-01	-1.41	1.30E-03	-0.49	5.14E-01	-1.12	1.65E-02
Phvul.006G065700	PF11493 - Thylakoid soluble phosphoprotein TSP9 (TSP9) (1 of 1)	-0.41	6.19E-02	-0.17	4.59E-01	-0.45	4.60E-02	0.07	7.70E-01
Phvul.003G231500	K07374 - tubulin alpha (TUBA) (1 of 7)	-0.41	3.43E-01	-0.74	1.31E-02	-0.28	6.10E-01	-0.67	3.38E-02
Phvul.001G230500	K14494 - DELLA protein (DELLA) (1 of 5)	-0.41	3.22E-01	-0.66	2.47E-02	-0.12	8.47E-01	-0.34	3.07E-01
Phvul.009G173000	PF05910 - Plant protein of unknown function (DUF868) (DUF868) (1 of 16)	-0.41	5.17E-01	-0.57	2.18E-01	-0.49	4.57E-01	-0.97	3.05E-02
Phvul.010G148700	PTHR24326:SF272 - HOMEOBOX-LEUCINE ZIPPER PROTEIN GLABRA 2 (1 of 2)	-0.42	2.15E-01	-0.59	1.99E-02	-0.39	2.92E-01	-0.37	1.82E-01
Phvul.011G026700	PTHR22950//PTHR22950:SF299 - AMINO ACID TRANSPORTER // SUBFAMILY NOT NAMED (1 of 1)	-0.42	7.77E-01	-1.90	2.00E-02	-0.38	8.26E-01	-1.40	1.02E-01
Phvul.008G255300	PTHR31288:SF5 - GENOMIC DNA, CHROMOSOME 3, P1 CLONE: MXL8-RELATED (1 of 2)	-0.42	4.28E-01	-1.12	1.13E-03	-0.53	3.20E-01	-0.93	1.00E-02
Phvul.006G207900	PTHR22814//PTHR22814:SF80 - COPPER TRANSPORT PROTEIN ATOX1-RELATED // SUBFAMILY NOT NAMED (1 of 2)	-0.42	8.32E-01	-2.10	4.27E-02	-0.22	9.35E-01	-1.66	1.25E-01
Phvul.001G268400	PTHR24031:SF235 - DEAD-BOX ATP-DEPENDENT RNA HELICASE 37-RELATED (1 of 2)	-0.42	3.05E-01	-0.78	7.00E-03	-0.34	4.67E-01	-0.62	4.27E-02
Phvul.007G155800	PTHR21726//PTHR21726:SF36 - PHOSPHATIDYLINOSITOL N-ACETYLGLUCOSAMINYLTRANSFERASE SUBUNIT P DOWN SY	-0.42	4.65E-01	-0.83	3.07E-02	-0.38	5.53E-01	-0.55	1.85E-01
Phvul.001G239400	PTHR15858:SF0 - IMMEDIATE EARLY RESPONSE 3-INTERACTING PROTEIN 1 (1 of 2)	-0.42	4.87E-01	-0.84	3.45E-02	-0.80	1.34E-01	-1.25	1.96E-03
Phvul.010G163500	no data	-0.42	2.43E-01	-0.75	4.14E-03	-0.42	2.76E-01	-0.59	3.59E-02
Phvul.011G118900	PTHR11439//PTHR11439:SF192 - GAG-POL-RELATED RETROTRANSPOSON // SUBFAMILY NOT NAMED (1 of 2)	-0.42	2.85E-01	-0.69	2.11E-02	-0.47	2.56E-01	-0.38	2.49E-01
Phvul.003G096700	PTHR27003:SF15 - LEUCINE-RICH REPEAT PROTEIN KINASE-LIKE PROTEIN (1 of 3)	-0.42	7.13E-01	1.59	1.12E-02	-0.15	9.29E-01	1.31	4.85E-02
Phvul.008G029100	PTHR31673:SF3 - COBRA-LIKE EXTRACELLULAR GLYCOSYL-PHOSPHATIDYL INOSITOL-ANCHORED FAMILY PROTEIN-RELAT	-0.42	4.45E-01	-0.85	1.95E-02	-0.42	4.83E-01	-0.85	2.53E-02
Phvul.006G115200	PTHR10641//PTHR10641:SF613 - MYB-LIKE DNA-BINDING PROTEIN MYB // SUBFAMILY NOT NAMED (1 of 2)	-0.42	7.42E-02	-0.41	3.88E-02	-0.31	2.68E-01	-0.35	9.08E-02
Phvul.008G171600	PF01915//PF14310 - Glycosyl hydrolase family 3 C-terminal domain (Glyco hydro 3 C) // Fibronectin type III-like domain	-0.42	7.80E-01	-1.59	6.56E-02	-0.83	5.46E-01	-1.83	3.59E-02
Phvul.004G085900	PTHR10797//PTHR10797:SF10 - CCR4-NOT TRANSCRIPTION COMPLEX SUBUNIT // SUBFAMILY NOT NAMED (1 of 15)	-0.42	1.84E-01	-0.51	4.43E-02	-0.39	2.76E-01	-0.43	1.04E-01
Phvul.009G016600	no data	-0.42	6.77E-01	-2.06	1.95E-03	0.00	9.99E-01	-1.15	8.13E-02
Phvul.002G262600	K17785 - mitofilin (IMMT, FCJ1, MNOS2) (1 of 1)	-0.42	1.78E-01	-0.73	1.68E-03	-0.50	1.19E-01	-0.57	2.08E-02
Phvul.002G294900	PF03417 - Acyl-coenzyme A:6-aminopenicillanic acid acyl-transferase (AAT) (1 of 1)	-0.42	7.01E-01	1.97	9.26E-06	-0.16	9.23E-01	1.10	3.38E-02
Phvul.003G160400	PTHR31561:SF3 - 3-KETOACYL-COA SYNTHASE 11 (1 of 2)	-0.42	3.43E-01	-0.70	3.33E-02	-0.39	4.25E-01	-0.42	2.40E-01
Phvul.003G084700	K08272 - calcium binding protein 39 (CAB39, MO25) (1 of 4)	-0.42	3.57E-01	-0.78	1.48E-02	-0.22	7.20E-01	-0.42	2.35E-01
Phvul.001G113800	K11292 - transcription elongation factor SPT6 (SUPT6H, SPT6) (1 of 2)	-0.42	4.53E-01	-0.80	3.56E-02	-0.29	6.78E-01	-0.79	4.40E-02
Phvul.004G044200	KOG0580 - Serine/threonine protein kinase (1 of 5)	-0.42	6.84E-01	-1.00	9.50E-02	0.14	9.28E-01	-1.61	9.27E-03
Phvul.007G197000	PTHR33136:SF5 - PROTEIN RALF-LIKE 1-RELATED (1 of 4)	-0.43	3.53E-01	-0.93	3.11E-03	-0.19	7.67E-01	-0.66	4.96E-02
Phvul.005G127000	PTHR11119:SF28 - NUCLEOBASE-ASCORBATE TRANSPORTER 12 (1 of 2)	-0.43	4.38E-01	-0.83	3.15E-02	-0.60	2.65E-01	-0.72	7.57E-02
Phvul.007G128900	PTHR23050 - CALCIUM BINDING PROTEIN (1 of 17)	-0.43	5.29E-01	-1.08	3.42E-02	-0.54	4.33E-01	-0.92	7.71E-02
Phvul.011G041000	K01179 - endoglucanase (E3.2.1.4) (1 of 3)	-0.43	4.09E-02	-0.37	4.29E-02	-0.44	4.50E-02	-0.28	1.57E-01
Phvul.009G155700	no data	-0.43	6.82E-01	-1.47	1.51E-02	-0.19	8.96E-01	-1.10	8.79E-02
Phvul.006G164100	PF04862 - Protein of unknown function (DUF642) (DUF642) (1 of 4)	-0.43	7.09E-01	-1.49	2.56E-02	-0.07	9.71E-01	-1.21	8.33E-02
Phvul.002G331400	PTHR27008:SF19 - INACTIVE RECEPTOR KINASE RLK902-RELATED (1 of 2)	-0.43	4.15E-01	-1.00	3.74E-03	-0.21	7.76E-01	-0.69	6.32E-02
Phvul.008G018100	PTHR22595:SF42 - CHITINASE-LIKE PROTEIN 1 (1 of 2)	-0.43	3.02E-01	-0.78	6.53E-03	-0.19	7.39E-01	-0.40	2.21E-01
Phvul.009G048300	PF05631 - Sugar-transporters, 12 TM (MFS 5) (1 of 2)	-0.43	2.88E-01	-0.79	5.06E-03	-0.46	2.83E-01	-0.84	3.96E-03
Phvul.010G123132	PTHR31707:SF42 - PECTINESTERASE 1-RELATED (1 of 2)	-0.43	5.03E-01	-1.15	7.74E-03	-0.46	4.98E-01	-0.76	9.66E-02
Phvul.002G266200	K19026 - spatacsin (SPG11) (1 of 1)	-0.43	2.34E-01	-0.66	1.42E-02	-0.51	1.72E-01	-0.74	7.42E-03
Phvul.002G034200	PTHR24078//PTHR24078:SF187 - DNAJ HOMOLOG SUBFAMILY C MEMBER // SUBFAMILY NOT NAMED (1 of 3)	-0.43	7.71E-02	-0.21	3.65E-01	-0.52	3.23E-02	-0.24	2.96E-01
Phvul.001G230400	PTHR11260:SF171 - GLUTATHIONE S-TRANSFERASE DHAR3, CHLOROPLASTIC (1 of 2)	-0.43	1.23E-01	-0.58	6.91E-03	-0.24	5.07E-01	-0.38	1.10E-01
Phvul.003G119000	PTHR11214:SF131 - BETA-1,3-GALACTOSYLTRANSFERASE 16-RELATED (1 of 1)	-0.43	3.55E-02	-0.31	1.15E-01	-0.48	2.14E-02	-0.35	7.72E-02
Phvul.006G038800	no data	-0.43	6.47E-01	-1.47	8.59E-03	-0.61	5.14E-01	-1.23	3.76E-02
Phvul.008G033300	PTHR11802:SF43 - SERINE CARBOXYPEPTIDASE-LIKE 25 (1 of 1)	-0.43	3.20E-01	-0.94	1.73E-03	-0.29	5.88E-01	-0.62	5.51E-02
Phvul.L007043	K07025 - putative hydrolase of the HAD superfamily (K07025) (1 of 3)	-0.43	5.10E-01	-0.73	8.64E-02	-0.44	5.33E-01	-1.09	1.10E-02
Phvul.009G058700	PTHR33472:SF3 - OXIDOREDUCTASE (1 of 2)	-0.43	6.46E-01	-1.47	2.30E-02	-0.06	9.69E-01	-0.35	6.38E-01
Phvul.008G041200	PTHR23201:SF21 - GIBBERELLIN-REGULATED PROTEIN 4 (1 of 3)	-0.43	5.99E-01	-1.13	2.31E-02	-0.59	4.64E-01	-1.15	2.53E-02
Phvul.009G039400	K02940 - large subunit ribosomal protein L9e (RP-L9e, RPL9) (1 of 4)	-0.43	3.17E-01	-0.39	2.47E-01	-0.23	6.85E-01	-0.72	2.46E-02
Phvul.004G048700	PF14416 - PMR5 N terminal Domain (PMR5N) (1 of 64)	-0.43	6.08E-01	-1.65	1.68E-03	-0.56	5.06E-01	-0.81	1.52E-01
Phvul.011G080100	PTHR31384:SF27 - AUXIN RESPONSE FACTOR 10-RELATED (1 of 4)	-0.43	2.16E-01	-0.60	2.14E-02	-0.40	3.04E-01	-0.56	4.02E-02
Phvul.008G061800	K02607 - origin recognition complex subunit 5 (ORC5) (1 of 1)	-0.43	2.44E-01	-0.49	1.10E-01	-0.46	2.52E-01	-0.75	1.34E-02
Phvul.007G071600	K01280 - tripeptidyl-peptidase II [EC:3.4.14.10] (TPP2) (1 of 2)	-0.43	2.82E-01	-0.90	1.32E-03	-0.41	3.57E-01	-0.55	7.40E-02
Phvul.004G034400	PTHR11468:SF8 - ALPHA-GLUCAN PHOSPHORYLASE 1 (1 of 1)	-0.43	1.53E-02	-0.55	3.44E-04	-0.40	3.77E-02	-0.39	2.03E-02

Phvul.005G173500	PTHR10209:SF95 - GIBBERELLIN 3-BETA-DIOXYGENASE 1-RELATED (1 of 2)	-0.43	5.27E-01	-0.97	3.50E-02	-0.53	4.53E-01	-0.84	7.55E-02
Phvul.003G011800	1.14.11.14 - 6-beta-hydroxyhyoscyamine epoxidase / Hydroxyhyoscyamine dioxygenase (1 of 1)	-0.43	3.73E-01	-1.01	2.00E-03	-0.40	4.70E-01	-0.90	8.37E-03
Phvul.011G148700	PTHR27003:SF37 - INTERLEUKIN-1 RECEPTOR-ASSOCIATED KINASE 4 PROTEIN-RELATED (1 of 2)	-0.43	5.23E-01	-0.95	3.02E-02	-0.18	8.52E-01	-0.66	1.63E-01
Phvul.003G139700	PTHR23216 - NUCLEOLAR AND COILED-BODY PHOSPHOPROTEIN 1 (1 of 1)	-0.43	3.51E-01	-0.74	4.92E-02	-0.59	2.10E-01	-0.40	3.19E-01
Phvul.009G067400	PTHR13902//PTHR13902:SF64 - SERINE/THREONINE-PROTEIN KINASE WNK WITH NO LYSINE -RELATED // SUBFAMILY N	-0.43	6.83E-01	-1.36	2.99E-02	-0.27	8.42E-01	-1.11	9.31E-02
Phvul.006G041600	K15077 - elongin-A (ELA1) (1 of 4)	-0.43	4.00E-01	-0.90	2.08E-02	-0.29	6.44E-01	-0.50	2.32E-01
Phvul.008G189900	no data	-0.43	1.27E-01	-0.71	1.15E-03	-0.41	2.06E-01	-0.50	3.31E-02
Phvul.003G049500	1.14.19.4 - Delta(8)-fatty-acid desaturase / SLD (1 of 3)	-0.44	2.27E-01	-0.59	3.08E-02	-0.42	2.86E-01	-0.47	1.03E-01
Phvul.001G017500	PTHR21569:SF2 - 30S RIBOSOMAL PROTEIN S9, CHLOROPLASTIC (1 of 1)	-0.44	2.36E-01	-0.74	5.36E-03	-0.27	5.60E-01	-0.58	3.92E-02
Phvul.001G008400	PTHR10108//PTHR10108:SF837 - METHYLTRANSFERASE // SUBFAMILY NOT NAMED (1 of 2)	-0.44	2.16E-01	-0.97	3.73E-04	-0.39	3.27E-01	-0.83	3.50E-03
Phvul.002G236500	1.14.12.20 - Pheophorbide a oxygenase / Pheide a oxygenase (1 of 4)	-0.44	7.67E-02	-0.41	4.92E-02	-0.28	3.77E-01	-0.26	2.50E-01
Phvul.003G273400	PTHR23324:SF84 - PHOSPHATIDYLINOSITOL/PHOSPHATIDYLCHOLINE TRANSFER PROTEIN SFH13-RELATED (1 of 2)	-0.44	2.68E-01	-0.82	4.13E-03	-0.28	5.68E-01	-0.53	8.69E-02
Phvul.003G092800	no data	-0.44	2.34E-01	-0.69	1.66E-02	-0.44	2.63E-01	-0.56	5.95E-02
Phvul.002G229900	PF05678 - VQ motif (VQ) (1 of 39)	-0.44	2.39E-01	-0.52	6.33E-02	-0.36	3.98E-01	-0.70	1.34E-02
Phvul.003G218000	PTHR10683:SF10 - TRANSALDOLASE-LIKE PROTEIN (1 of 1)	-0.44	2.31E-01	-0.84	1.31E-03	-0.39	3.48E-01	-0.81	3.18E-03
Phvul.001G250100	KOG2160//KOG4224 - Armadillo/beta-catenin-like repeat-containing protein // Armadillo repeat protein VAC8 required	-0.44	2.58E-01	-0.60	3.92E-02	-0.52	1.94E-01	-0.54	7.19E-02
Phvul.001G161500	5.3.1.13 - Arabinose-5-phosphate isomerase / Phosphoarabinoisomerase (1 of 1)	-0.44	4.38E-01	-0.94	1.30E-02	-0.21	7.95E-01	-0.50	2.37E-01
Phvul.008G010300	PF04720 - PDDEXK-like family of unknown function (PDDEXK 6) (1 of 14)	-0.44	2.83E-01	-0.56	6.73E-02	-0.30	5.42E-01	-0.66	3.22E-02
Phvul.006G208200	K15920 - beta-D-xylosidase 4 (XYL4) (1 of 2)	-0.44	3.91E-01	-0.83	1.66E-02	-0.30	6.44E-01	-0.50	1.89E-01
Phvul.001G222500	PTHR32227:SF118 - CARBOHYDRATE-BINDING X8 DOMAIN-CONTAINING PROTEIN (1 of 2)	-0.44	3.73E-01	-0.72	4.60E-02	-0.42	4.47E-01	-0.72	5.10E-02
Phvul.005G084000	PTHR31008:SF4 - COP1-INTERACTING PROTEIN 7 (CIP7) (1 of 1)	-0.44	2.31E-01	-0.62	2.44E-02	-0.40	3.29E-01	-0.31	3.08E-01
Phvul.007G185900	PTHR13902:SF21 - SERINE/THREONINE-PROTEIN KINASE WNK1-RELATED (1 of 2)	-0.44	1.45E-02	-0.35	3.74E-02	-0.39	5.59E-02	-0.13	5.01E-01
Phvul.011G073600	PTHR32191:SF7 - TETRASPANIN-16-RELATED (1 of 2)	-0.44	1.71E-01	-1.03	2.18E-05	-0.41	2.55E-01	-0.84	8.37E-04
Phvul.006G126100	PTHR32448:SF34 - F22M8.11 PROTEIN-RELATED (1 of 7)	-0.44	1.55E-01	-0.49	4.20E-02	-0.27	5.03E-01	-0.40	1.19E-01
Phvul.002G084100	PTHR31499:SF11 - HOMEODOMAIN-LIKE SUPERFAMILY PROTEIN (1 of 2)	-0.45	1.82E-01	-0.70	5.58E-03	-0.14	7.92E-01	-0.51	6.16E-02
Phvul.001G210300	PF12708 - Pectate lyase superfamily protein (Pectate lyase 3) (1 of 32)	-0.45	7.22E-01	-1.12	1.94E-01	-0.83	4.88E-01	-1.83	4.03E-02
Phvul.009G166100	K01652 - acetolactate synthase I/II/III large subunit (E2.2.1.6L, ilvB, ilvG, ilvI) (1 of 2)	-0.45	6.11E-01	-1.13	3.84E-02	-0.30	7.89E-01	-1.11	4.97E-02
Phvul.006G192800	PTHR20854:SF30 - PAP-SPECIFIC PHOSPHATASE, MITOCHONDRIAL-RELATED (1 of 1)	-0.45	1.31E-01	-0.76	7.44E-04	-0.21	6.15E-01	-0.37	1.45E-01
Phvul.007G060600	KOG0978//KOG1003//KOG4643 - E3 ubiquitin ligase involved in syntaxin degradation // Actin filament-coating protein t	-0.45	5.68E-01	-1.24	9.42E-03	-0.71	3.31E-01	-1.03	4.29E-02
Phvul.008G072300	PTHR23155//PTHR23155:SF590 - LEUCINE-RICH REPEAT-CONTAINING PROTEIN // SUBFAMILY NOT NAMED (1 of 15)	-0.45	4.26E-01	0.03	9.67E-01	-1.00	3.95E-02	-0.02	9.76E-01
Phvul.008G245900	2.7.10.1//2.7.11.1 - Receptor protein-tyrosine kinase / Receptor protein tyrosine kinase // Non-specific serine/threonine	-0.45	1.69E-01	-0.71	4.24E-03	-0.48	1.70E-01	-0.60	2.28E-02
Phvul.002G279500	PTHR12415//PTHR12415:SF2 - TYROSYL-DNA PHOSPHODIESTERASE 1 // SUBFAMILY NOT NAMED (1 of 1)	-0.45	2.14E-01	-0.57	4.31E-02	-0.49	2.06E-01	-0.35	2.51E-01
Phvul.005G067950	PTHR31072:SF3 - TRANSCRIPTION FACTOR TCP10-RELATED (1 of 3)	-0.45	2.23E-01	-0.61	2.59E-02	-0.32	4.71E-01	-0.49	9.06E-02
Phvul.008G161100	PTHR22835//PTHR22835:SF239 - ZINC FINGER FYVE DOMAIN CONTAINING PROTEIN // SUBFAMILY NOT NAMED (1 of 6)	-0.45	4.81E-01	-1.02	1.48E-02	-0.48	4.77E-01	-0.86	5.16E-02
Phvul.003G163950	K12845 - U4/U6 small nuclear ribonucleoprotein SNU13 (SNU13, NHP2L) (1 of 2)	-0.45	3.59E-01	-0.71	3.83E-02	-0.42	4.44E-01	-0.97	5.12E-03
Phvul.002G282800	PTHR24089:SF269 - MITOCHONDRIAL PHOSPHATE CARRIER PROTEIN 3, MITOCHONDRIAL (1 of 2)	-0.45	5.76E-01	-1.56	1.37E-03	-0.36	7.05E-01	-1.14	2.71E-02
Phvul.003G208000	PTHR22835//PTHR22835:SF231 - ZINC FINGER FYVE DOMAIN CONTAINING PROTEIN // SUBFAMILY NOT NAMED (1 of 1)	-0.45	4.46E-01	-0.96	1.46E-02	-0.35	6.23E-01	-0.77	6.63E-02
Phvul.008G267800	PTHR10388//PTHR10388:SF16 - EUKARYOTIC TRANSLATION INITIATION FACTOR SUI1 // SUBFAMILY NOT NAMED (1 of 2	-0.45	2.16E-01	-0.80	2.47E-03	-0.51	1.92E-01	-0.69	1.35E-02
Phvul.002G071500	PF01535//PF13041//PF13812 - PPR repeat (PPR) // PPR repeat family (PPR 2) // Pentatricopeptide repeat domain (PPR	-0.45	5.37E-01	-0.96	4.35E-02	-0.22	8.32E-01	-0.80	1.07E-01
Phvul.006G165000	PTHR10209:SF171 - GIBBERELLIN 2-BETA-DIOXYGENASE 2-RELATED (1 of 2)	-0.45	1.41E-01	-1.29	6.02E-07	-0.29	4.46E-01	-0.45	9.56E-02
Phvul.005G182600	2.4.1.69 - Galactoside 2-alpha-L-fucosyltransferase / Secretor-type beta-galactoside alpha-1->2 fucosyltransferase (1 of	-0.45	9.18E-02	-0.40	9.59E-02	-0.57	3.23E-02	-0.52	2.86E-02
Phvul.005G001200	PTHR22835//PTHR22835:SF207 - ZINC FINGER FYVE DOMAIN CONTAINING PROTEIN // SUBFAMILY NOT NAMED (1 of 1)	-0.46	1.41E-01	-0.60	1.30E-02	-0.38	2.87E-01	-0.45	8.52E-02
Phvul.007G227600	PTHR10071//PTHR10071:SF173 - TRANSCRIPTION FACTOR GATA GATA BINDING FACTOR // SUBFAMILY NOT NAMED (1	-0.46	2.16E-01	-0.92	6.24E-04	-0.31	5.00E-01	-0.73	9.40E-03
Phvul.L003700	K02922 - large subunit ribosomal protein L37e (RP-L37e, RPL37) (1 of 4)	-0.46	2.51E-01	-0.51	8.94E-02	-0.19	7.49E-01	-0.76	1.08E-02
Phvul.004G034300	K02160 - acetyl-CoA carboxylase biotin carboxyl carrier protein (accB, bccP) (1 of 3)	-0.46	3.33E-01	-1.14	3.91E-04	-0.46	3.68E-01	-0.86	1.22E-02
Phvul.006G121500	PTHR10724 - S1 RNA-BINDING DOMAIN-CONTAINING PROTEIN 1 (1 of 2)	-0.46	5.97E-01	-1.34	1.62E-02	-0.62	4.66E-01	-0.93	1.15E-01
Phvul.003G226600	PTHR21230:SF27 - NOVEL PLANT SNARE 11 (1 of 1)	-0.46	3.20E-01	-0.72	2.71E-02	-0.41	4.19E-01	-0.67	4.99E-02
Phvul.003G276200	PTHR11527//PTHR11527:SF144 - SMALL HEAT-SHOCK PROTEIN HSP20 FAMILY // SUBFAMILY NOT NAMED (1 of 2)	-0.46	1.46E-01	-0.61	1.99E-02	-0.31	4.18E-01	-0.43	1.23E-01
Phvul.002G090100	PF04398 - Protein of unknown function, DUF538 (DUF538) (1 of 22)	-0.46	1.97E-01	-0.77	2.99E-03	-0.19	7.16E-01	-0.52	6.16E-02
Phvul.010G069500	K08876 - SCY1-like protein 1 (SCYL1) (1 of 2)	-0.46	5.33E-01	-1.03	2.70E-02	-0.38	6.56E-01	-1.22	1.10E-02
Phvul.003G043000	PF01535//PF13812 - PPR repeat (PPR) // Pentatricopeptide repeat domain (PPR 3) (1 of 12)	-0.46	1.12E-01	-0.60	8.30E-03	-0.32	3.59E-01	-0.46	5.66E-02
Phvul.007G177800	PF01535//PF13041 - PPR repeat (PPR) // PPR repeat family (PPR 2) (1 of 197)	-0.46	1.68E-01	-0.16	6.22E-01	-0.64	4.62E-02	-0.22	4.76E-01
Phvul.007G188100	PTHR19317:SF3 - PRA1 FAMILY PROTEIN E (1 of 1)	-0.46	2.72E-01	-0.41	2.36E-01	-0.35	4.66E-01	-0.73	2.84E-02
Phvul.011G214700	K02925 - large subunit ribosomal protein L3e (RP-L3e, RPL3) (1 of 2)	-0.46	2.45E-01	-0.29	3.81E-01	-0.40	3.79E-01	-0.60	4.87E-02
Phvul.006G171500	PF12796//PF13962 - Ankyrin repeats (3 copies) (Ank 2) // Domain of unknown function (PGG) (1 of 27)	-0.46	3.58E-01	-1.05	5.31E-03	-0.54	3.00E-01	-0.41	3.27E-01
Phvul.006G013500	PTHR32468:SF17 - CATION/H(+) ANTIPORTER 10-RELATED (1 of 5)	-0.46	6.09E-01	-0.51	4.38E-01	-1.11	1.37E-01	-1.24	3.69E-02
Phvul.004G038000	K18635 - protein SPIRAL1 and related proteins (SPR1) (1 of 4)	-0.46	4.10E-01	-1.16	1.61E-03	-0.33	6.23E-01	-0.96	1.31E-02
Phvul.011G058300	PTHR22754:SF4 - DISCO-INTERACTING PROTEIN 2 (1 of 1)	-0.46	5.76E-01	-1.55	9.34E-03	-0.55	5.20E-01	-0.67	2.83E-01
Phvul.008G049000	2.5.1.55 - 3-deoxy-8-phosphooctulonate synthase / Phospho-2-keto-3-deoxyoctonate aldolase (1 of 1)	-0.46	1.59E-01	-0.85	6.24E-04	-0.39	3.05E-01	-0.68	9.49E-03
Phvul.003G260200	K11498 - centromeric protein E (CENPE) (1 of 14)	-0.46	9.49E-02	-0.48	3.30E-02	-0.34	3.03E-01	-0.38	1.12E-01
Phvul.003G264400	PTHR11607:SF18 - ALPHA-MANNOSIDASE (1 of 2)	-0.46	6.20E-01	-1.71	1.57E-03	-0.68	4.45E-01	-1.55	6.11E-03
Phvul.001G167700	PTHR27001:SF10 - RECEPTOR-LIKE SERINE/THREONINE-PROTEIN KINASE ALE2 (1 of 4)	-0.46	2.39E-01	-0.62	3.80E-02	-0.31	5.17E-01	-0.64	3.87E-02
Phvul.002G142800	PF00010 - Helix-loop-helix DNA-binding domain (HLH) (1 of 158)	-0.46	5.20E-01	-0.96	3.80E-02	-0.49	5.20E-01	-0.86	7.69E-02
Phvul.010G087600	PTHR23063:SF9 - LYSOPHOSPHOLIPID ACYLTRANSFERASE LPEAT2 (1 of 1)	-0.46	4.25E-02	-0.82	1.16E-05	-0.51	2.80E-02	-0.62	1.55E-03
Phvul.011G089900	PTHR32044:SF17 - GLUCOMANNAN 4-BETA-MANNOSYLTRANSFERASE 2 (1 of 1)	-0.46	5.98E-01	-1.56	3.14E-03	-0.53	5.64E-01	-1.07	5.69E-02
Phvul.007G110900	no data	-0.46	1.69E-01	-0.76	2.71E-03	-0.42	2.76E-01	-0.72	6.06E-03
Phvul.006G110400	K11600 - exosome complex component RRP41 (RRP41, EXOSC4, SKI6) (1 of 1)	-0.46	7.63E-01	-3.04	3.04E-04	-0.87	5.24E-01	-2.32	7.74E-03
Phvul.007G226500	PTHR31065:SF4 - PLATZ TRANSCRIPTION FACTOR FAMILY PROTEIN (1 of 3)	-0.46	7.71E-02	-0.83	5.19E-05	-0.34	2.82E-01	-0.49	2.77E-02
Phvul.005G039000	KOG0131 - Splicing factor 3b, subunit 4 (1 of 23)	-0.47	1.23E-01	-0.87	1.30E-04	-0.46	1.60E-01	-0.68	5.18E-03
Phvul.009G185950	PTHR10641//PTHR10641:SF648 - MYB-LIKE DNA-BINDING PROTEIN MYB // SUBFAMILY NOT NAMED (1 of 1)	-0.47	3.64E-01	-1.04	2.69E-03	-0.51	3.51E-01	-0.94	9.56E-03
Phvul.003G231700	no data	-0.47	2.35E-01	-0.93	1.02E-03	-0.44	3.13E-01	-0.68	2.30E-02
Phvul.007G159500	PTHR21726:SF49 - PHOSPHATIDYLINOSITOL N-ACETYGLUCOSAMINLYTRANSFERASE SUBUNIT P-LIKE PROTEIN (1 of 3)	-0.47	1.24E-01	-0.51	4.77E-02	-0.64	2.79E-02	-0.54	3.61E-02
Phvul.010G117100	PTHR24298:SF46 - CYTOCHROME P450 78A6-RELATED (1 of 1)	-0.47	3.43E-01	-0.68	4.90E-02	-0.30	6.28E-01	-0.60	9.90E-02
Phvul.002G175500	PTHR11771:SF60 - LIPOXYGENASE 6, CHLOROPLASTIC (1 of 1)	-0.47	3.22E-01	-0.71	4.38E-02	-0.39	4.76E-01	-0.33	4.10E-01
Phvul.008G247800	PF00170 - bZIP transcription factor (bZIP 1) (1 of 66)	-0.47	9.11E-02	-0.90	5.82E-05	-0.29	4.07E-01	-0.60	1.16E-02
Phvul.006G173850	no data	-0.47	4.60E-01	-1.02	2.78E-02	-0.13	8.92E-01	-0.42	4.09E-01
Phvul.002G238900	no data	-0.47	1.26E-01	-0.57	2.11E-02	-0.51	1.17E-01	-0.32	2.41E-01
Phvul.002G209900	KOG1546 - Metacaspase involved in regulation of apoptosis (1 of 9)	-0.47	6.69E-01	-1.92	4.74E-02	-0.56	6.23E-01	-0.02	9.86E-01
Phvul.007G164300	PTHR32295:SF32 - PROTEIN IQ-DOMAIN 3-RELATED (1 of 4)	-0.47	1.00E-01	-0.46	4.72E-02	-0.30	4.16E-01	-0.31	2.20E-01
Phvul.009G139500	PTHR32438:SF5 - 4-ALPHA-GLUCANOTRANSFERASE DPE1, CHLOROPLASTIC/AMYLOPLASTIC (1 of 1)	-0.47	3.74E-02	-0.64	5.79E-04	-0.42	9.62E-02	-0.64	9.75E-04
Phvul.002G075000	PTHR23155//PTHR23155:SF505 - LEUCINE-RICH REPEAT-CONTAINING PROTEIN // SUBFAMILY NOT NAMED (1 of 23)	-0.47	6.08E-01	-0.67	3.39E-01	-1.51	4.62E-02	-0.63	3.63E-01
Phvul.002G152100	PTHR27000:SF7 - F16P17.10 PROTEIN (1 of 1)	-0.47	2.29E-01	-0.61	3.97E-02	-0.49	2.47E-01	-0.78	9.16E-03
Phvul.008G027200	K04733 - interleukin-1 receptor-associated kinase 4 (IRAK4) (1 of 48)	-0.47	1.06E-01	-0.59	1.24E-02	-0.35	3.12E-01	-0.26	3.11E-01
Phvul.009G084200	PTHR10366//PTHR10366:SF471 - NAD DEPENDENT EPIMERASE/DEHYDRATASE // SUBFAMILY NOT NAMED (1 of 1)	-0.47	1.27E-01	-0.71	4.24E-03	-0.29	4.59E-01	-0.66	1.20E-02
Phvul.010G116700	PF13301 - Protein of unknown function (DUF4079) (DUF4079) (1 of 3)	-0.47	4.53E-01	-0.88	4.46E-02	-0.38	6.15E-01	-0.59	2.06E-01
Phvul.002G295600	PTHR31194//PTHR31194:SF16 - SHN SHINE , DNA BINDING / TRANSCRIPTION FACTOR // SUBFAMILY NOT NAMED (1 of	-0.47	5.25E-01	-1.11	1.96E-02	-0.47	5.63E-01	-1.05	3.41E-02
Phvul.009G112450	K00850 - 6-phosphofructokinase 1 (pfkA, PFK) (1 of 9)	-0.47	1.99E-01	-0.87	1.66E-03	-0.37	3.96E-01	-0.87	2.46E-03
Phvul.003G196100	PF02985 - HEAT repeat (HEAT) (1 of 13)	-0.48	5.82E-01	-1.11	4.99E-02	-0.51	5.82E-01	-0.97	1.04E-01
Phvul.001G217600	PTHR24078:SF255 - DNAJ HOMOLOG DNJ-5 (1 of 2)	-0.48	3.36E-01	-0.75	4.81E-02	-0.14	8.45E-01	-0.53	1.83E-01
Phvul.002G330700	3.6.3.27 - Phosphate-transporting ATPase / ABC phosphate transporter (1 of 7)	-0.48	2.53E-01	-0.87	3.63E-03	-0.37	4.46E-01	-0.59	6.49E-02
Phvul.011G073301	PTHR21022:SF1 - AROGENATE DEHYDRATASE/PREPHENATE DEHYDRATASE 2, CHLOROPLASTIC (1 of 1)	-0.48	1.79E-02	-0.50	4.38E-03	-0.50	1.74E-02	-0.36	5.83E-02
Phvul.008G274200	PTHR23344:SF8 - GLYCEROPHOSPHODIESTER PHOSPHODIESTERASE-LIKE PROTEIN-RELATED (1 of 6)	-0.48	9.98E-02	-0.58	1.21E-02	-0.57	4.85E-02	-0.44	7.66E-02
Phvul.008G001600	PTHR31614:SF6 - POLLEN OLE E 1 ALLERGEN AND EXTENSIN FAMILY PROTEIN (1 of 1)	-0.48	4.48E-01	-0.98	3.81E-02	-0.43	5.43E-01	-0.75	1.29E-01
Phvul.001G161700	PF02365 - No apical meristem (NAM) protein (NAM) (1 of 91)	-0.48	5.77E-01	1.08	3.28E-02	-0.57	5.24E-01	0.42	5.09E-01
Phvul.010G122400	K03691 - peptide-O-fucosyltransferase (POFUT) (1 of 2)	-0.48	4.52E-01	-1.05	1.24E-02	-0.33	6.69E-01	-0.84	5.83E-02
Phvul.005G184900	no data	-0.48	4.46E-02	-0.76	4.55E-04	-0.50	4.28E-02	-0.68	2.35E-03
Phvul.003G008900	PF16058 - Mucin-like (Mucin-like) (1 of 1)	-0.48	1.76E-01	-0.62	2.24E-02	-0.37	3.82E-01	-0.46	1.15E-01
Phvul.004G060000	K03231 - elongation factor 1-alpha (EEF1A) (1 of 3)	-0.48	2.62E-01	-0.47	1.55E-01	-0.35	5.00E-01	-0.68	3.43E-02
Phvul.010G099200	PTHR21726:SF30 - PHOSPHATIDYLINOSITOL N-ACETYGLUCOSAMINLYTRANSFERASE SUBUNIT P-LIKE PROTEIN (1 of 2)	-0.48	4.58E-02	-0.45	4.54E-02	-0.54	2.66E-02	-0.12	6.50E-01
Phvul.002G321400	K14312 - nuclear pore complex protein Nup155 (NUP155, NUP170, NUP157) (1 of 1)	-0.48	1.72E-01	-0.47	8.89E-02	-0.23	6.45E-01	-0.67	1.51E-02
Phvul.001G007300	PTHR12300:SF26 - HVA22-LIKE PROTEIN G-RELATED (1 of 2)	-0.48	1.91E-01	-1.07	9.85E-05	-0.51	1.90E-01	-0.75	9.87E-03
Phvul.010G058000	PTHR36310:SF1 - T6D22.11 (1 of 1)	-0.48	3.99E-01	-1.06	6.30E-03	-0.50	4.20E-01	-0.70	9.33E-02
Phvul.002G229700	PTHR13935:SF46 - BASIC HELIX-LOOP-HELIX DOMAIN-CONTAINING PROTEIN (1 of 2)	-0.48	4.25E-01	-1.05	8.58E-03	-0.17	8.43E-01	-0.88	3.65E-02

Phvul.003G027400	PTHR13778:SF4 - GALACTURONOSYLTRANSFERASE-LIKE 3-RELATED (1 of 1)	-0.48	3.94E-01	-0.81	3.92E-02	-0.12	8.94E-01	-0.42	3.37E-01
Phvul.008G076900	PTHR32116:SF12 - GALACTURONOSYLTRANSFERASE 7-RELATED (1 of 1)	-0.48	8.52E-02	-0.65	4.23E-03	-0.41	2.15E-01	-0.71	2.60E-03
Phvul.003G156200	3.2.2.20 - DNA-3-methyladenine glycosylase I / DNA-3-methyladenine glycosidase I (1 of 7)	-0.48	3.92E-01	-0.84	3.20E-02	-0.33	6.38E-01	-1.01	1.19E-02
Phvul.002G121200	PTHR19241:SF213 - ABC TRANSPORTER G FAMILY MEMBER 14 (1 of 2)	-0.48	1.58E-01	-0.59	2.76E-02	-0.42	2.83E-01	-0.33	2.64E-01
Phvul.009G107301	3.4.22.40 - Bleomycin hydrolase / Aminopeptidase C (Lactococcus lactis) (1 of 3)	-0.48	4.11E-01	-1.21	1.30E-02	-0.17	8.39E-01	-0.33	5.37E-01
Phvul.008G029000	PTHR31673:SF2 - COBRA-LIKE PROTEIN 4 (1 of 2)	-0.49	7.02E-01	-1.87	3.73E-02	-0.62	6.38E-01	-0.20	8.59E-01
Phvul.002G091400	PTHR22883//PTHR22883:SF96 - ZINC FINGER DHHC DOMAIN CONTAINING PROTEIN // SUBFAMILY NOT NAMED (1 of 2)	-0.49	1.88E-01	-0.79	3.65E-03	-0.41	3.30E-01	-0.75	8.53E-03
Phvul.003G235400	PF00651//PF03000 - BTB/POZ domain (BTB) // NPH3 family (NPH3) (1 of 21)	-0.49	2.74E-01	-0.86	9.23E-03	-0.27	6.40E-01	-0.60	8.58E-02
Phvul.007G274300	PTHR10108:SF906 - METHYLTRANSFERASE PMT1-RELATED (1 of 4)	-0.49	7.82E-02	-0.63	4.49E-03	-0.43	1.70E-01	-0.51	3.38E-02
Phvul.008G266800	3.5.99.10 - 2-iminobutanoate/2-iminopropanoate deaminase / Enamine/imine deaminase (1 of 2)	-0.49	1.43E-01	-0.83	1.01E-03	-0.37	3.57E-01	-0.84	1.12E-03
Phvul.004G095400	1.3.1.22 - 3-oxo-5-alpha-steroid 4-dehydrogenase (NADP(+)) / Cholestenone 5-alpha-reductase (1 of 3)	-0.49	8.89E-02	-0.51	2.67E-02	-0.30	4.14E-01	-0.43	8.31E-02
Phvul.001G142500	PTHR11937//PTHR11937:SF210 - ACTIN // SUBFAMILY NOT NAMED (1 of 1)	-0.49	3.21E-01	-0.74	3.33E-02	-0.35	5.52E-01	-0.74	4.08E-02
Phvul.001G236801	2.7.7.13 - Mannose-1-phosphate guanylyltransferase / GTP-mannose-1-phosphate guanylyltransferase (1 of 4)	-0.49	9.08E-02	-0.53	2.57E-02	-0.32	3.83E-01	-0.38	1.39E-01
Phvul.003G051100	no data	-0.49	3.59E-01	-0.70	6.57E-02	-0.28	6.84E-01	-0.77	4.60E-02
Phvul.003G196600	K09391 - transcription factor E2F7/8 (E2F7 8) (1 of 2)	-0.49	6.42E-01	-0.69	3.86E-01	-1.04	2.62E-01	-1.62	3.06E-02
Phvul.003G178800	PTHR27000:SF18 - LEUCINE-RICH REPEAT-CONTAINING PROTEIN (1 of 3)	-0.49	7.84E-01	1.79	1.58E-01	0.15	9.55E-01	2.58	2.88E-02
Phvul.009G051700	no data	-0.49	3.05E-01	-0.79	2.20E-02	-0.32	5.95E-01	-0.76	3.35E-02
Phvul.011G004400	PF04674 - Phosphate-induced protein 1 conserved region (Phi 1) (1 of 11)	-0.49	5.56E-01	-1.43	6.06E-03	-0.25	8.27E-01	-1.37	1.17E-02
Phvul.004G077550	K14641 - apyrase (APY1 2) (1 of 4)	-0.49	4.04E-01	-1.25	2.12E-03	-0.33	6.48E-01	-0.82	5.83E-02
Phvul.008G214100	PTHR36705:SF2 - CLAVATA3/ESR (CLE)-RELATED PROTEIN 20 (1 of 2)	-0.49	8.49E-02	-0.95	1.59E-05	-0.59	3.99E-02	-0.68	3.37E-03
Phvul.011G035400	PTHR11544 - COLD SHOCK DOMAIN CONTAINING PROTEINS (1 of 6)	-0.49	2.37E-01	-0.90	2.77E-03	-0.37	4.62E-01	-0.68	3.41E-02
Phvul.L004743	PTHR24128:SF14 - E3 UBIQUITIN-PROTEIN LIGASE XBAT31-RELATED (1 of 3)	-0.49	2.86E-01	-0.66	5.25E-02	-0.45	3.82E-01	-0.76	2.85E-02
Phvul.006G134900	PTHR23029:SF49 - PHOSPHOHISTIDINE PHOSPHATASE SIXA (1 of 1)	-0.49	3.46E-01	-0.89	2.14E-02	-0.42	4.77E-01	-0.86	3.11E-02
Phvul.004G046800	PTHR27001:SF217 - PROTEIN NSP-INTERACTING KINASE 1 (1 of 2)	-0.49	3.01E-01	-0.85	1.84E-02	-0.43	4.25E-01	-0.50	1.96E-01
Phvul.004G022100	no data	-0.49	3.57E-01	-0.90	1.51E-02	-0.36	5.80E-01	-0.67	9.39E-02
Phvul.011G058500	K10277 - lysine-specific demethylase 8 [EC:1.14.11.27] (KDM8, JMJD5) (1 of 1)	-0.50	3.49E-01	-1.59	7.96E-05	-0.46	4.29E-01	-1.01	1.16E-02
Phvul.001G185800	2.4.1.32 - Glucomannan 4-beta-mannosyltransferase / Glucomannan-synthase (1 of 4)	-0.50	5.06E-01	-1.22	9.86E-03	-0.42	6.25E-01	-1.03	4.00E-02
Phvul.008G106300	K09591 - steroid 5-alpha-reductase (DET2) (1 of 1)	-0.50	4.90E-02	-0.86	9.34E-05	-0.69	3.45E-03	-0.54	2.04E-02
Phvul.001G223500	PTHR22835:SF275 - GDSL ESTERASE/LIPASE LIP-4 (1 of 1)	-0.50	4.41E-01	-1.32	1.99E-03	-0.49	4.88E-01	-1.26	4.46E-03
Phvul.009G094200	PTHR13301:SF33 - CELLULOSE SYNTHASE A CATALYTIC SUBUNIT 1 [UDP-FORMING] (1 of 1)	-0.50	1.92E-01	-0.67	1.91E-02	-0.39	3.93E-01	-0.48	1.18E-01
Phvul.003G127801	PTHR31374:SF22 - AUXIN-RESPONSIVE PROTEIN-LIKE PROTEIN-RELATED (1 of 2)	-0.50	4.07E-01	-1.12	7.57E-03	-0.26	7.45E-01	-0.95	3.01E-02
Phvul.009G148900	PTHR31707:SF24 - PECTINESTERASE/PECTINESTERASE INHIBITOR 51-RELATED (1 of 2)	-0.50	5.02E-01	-0.89	8.32E-02	-0.25	8.03E-01	-1.08	3.57E-02
Phvul.L003646	PTHR10980:SF3 - LD16419P (1 of 1)	-0.50	6.54E-01	-2.21	9.90E-04	-0.23	8.80E-01	-1.09	1.28E-01
Phvul.005G036600	PTHR27000:SF151 - DNA-DAMAGE-REPAIR/TOLERATION PROTEIN DRT100-RELATED (1 of 4)	-0.50	5.94E-01	-1.50	8.16E-03	-0.37	7.46E-01	-1.22	4.28E-02
Phvul.002G041900	no data	-0.50	4.59E-01	-1.26	4.39E-03	-0.49	5.07E-01	-0.96	4.07E-02
Phvul.011G031700	PTHR31388:SF37 - PEROXIDASE 4-RELATED (1 of 2)	-0.50	3.99E-01	-1.06	7.79E-03	-0.45	4.93E-01	-1.05	1.09E-02
Phvul.001G041600	PTHR19315:SF9 - ER MEMBRANE PROTEIN COMPLEX SUBUNIT 4 (1 of 1)	-0.50	4.87E-01	-1.49	1.23E-03	-0.59	4.29E-01	-1.26	8.89E-03
Phvul.003G142300	PTHR24343:SF102 - CBL-INTERACTING SERINE/THREONINE-PROTEIN KINASE 7 (1 of 2)	-0.50	6.50E-01	1.45	2.94E-02	-0.43	7.41E-01	1.00	1.65E-01
Phvul.007G217500	PF12708 - Pectate lyase superfamily protein (Pectate lyase 3) (1 of 32)	-0.50	2.85E-01	-0.85	1.19E-02	-0.52	3.04E-01	-0.80	2.25E-02
Phvul.009G188100	PF03634 - TCP family transcription factor (TCP) (1 of 27)	-0.50	2.34E-01	-0.65	4.12E-02	-0.37	4.60E-01	-0.54	1.06E-01
Phvul.005G013300	no data	-0.50	2.16E-01	-1.18	2.08E-04	-0.04	9.61E-01	-0.69	3.61E-02
Phvul.008G174100	PF13947 - Wall-associated receptor kinase galacturonan-binding (GUB WAK bind) (1 of 65)	-0.50	6.49E-01	-1.73	1.09E-02	-0.56	6.45E-01	-1.21	9.13E-02
Phvul.009G017300	PTHR15141//PTHR15141:SF48 - TRANSCRIPTION ELONGATION FACTOR B POLYPEPTIDE 3 // SUBFAMILY NOT NAMED (1	-0.50	5.94E-01	-1.73	2.11E-03	-0.44	6.81E-01	-1.59	6.92E-03
Phvul.004G029000	PF05729//PF13676 - NACHT domain (NACHT) // TIR domain (TIR 2) (1 of 5)	-0.51	3.04E-01	0.12	8.19E-01	-0.96	2.36E-02	-0.22	6.40E-01
Phvul.011G213600	PTHR24073:SF458 - RAS-RELATED PROTEIN RABA4A (1 of 1)	-0.51	4.09E-01	-0.56	2.50E-01	-0.76	2.11E-01	-1.27	6.02E-03
Phvul.006G120500	PF13668 - Ferritin-like domain (Ferritin 2) (1 of 1)	-0.51	3.07E-01	-0.38	3.53E-01	-0.51	3.45E-01	-0.84	2.16E-02
Phvul.002G316200	PTHR33734:SF3 - LYSM DOMAIN-CONTAINING GPI-ANCHORED PROTEIN 2 (1 of 1)	-0.51	2.14E-01	-0.83	9.20E-03	-0.18	7.67E-01	-0.51	1.22E-01
Phvul.003G169100	PF14547 - Hydrophobic seed protein (Hydrophob seed) (1 of 17)	-0.51	7.80E-01	-2.43	1.59E-02	-0.45	8.31E-01	-2.21	3.61E-02
Phvul.002G008400	PTHR31062:SF48 - XYLOGLUCAN ENDOTRANSGLUCOSYLASE/HYDROLASE PROTEIN 6-RELATED (1 of 2)	-0.51	6.25E-01	-1.38	3.02E-02	-0.37	7.75E-01	-1.15	8.60E-02
Phvul.002G328908	K03252 - translation initiation factor 3 subunit C (EIF3C) (1 of 2)	-0.51	1.57E-02	-0.23	2.71E-01	-0.01	9.85E-01	-0.03	9.00E-01
Phvul.006G121300	K10589 - ubiquitin-protein ligase E3 C (UBE3C) (1 of 2)	-0.51	6.74E-01	-1.52	4.36E-02	-0.49	7.23E-01	-1.21	1.26E-01
Phvul.006G148700	K00605 - aminomethyltransferase (gcvT, AMT) (1 of 2)	-0.51	1.49E-01	-0.63	2.57E-02	-0.43	2.92E-01	-0.41	1.86E-01
Phvul.001G265500	PTHR27001:SF172 - GENOMIC DNA, CHROMOSOME 3, P1 CLONE: MYM9 (1 of 1)	-0.51	1.60E-01	-0.61	2.96E-02	-0.30	5.18E-01	-0.58	4.83E-02
Phvul.009G043400	PTHR26402:SF521 - TWO-COMPONENT RESPONSE REGULATOR ARR16-RELATED (1 of 1)	-0.51	7.28E-01	-2.08	2.11E-02	-0.44	7.99E-01	-1.16	2.15E-01
Phvul.002G289100	6.3.2.2 - Glutamate--cysteine ligase / Gamma-glutamylcysteine synthetase (1 of 2)	-0.51	3.72E-01	-0.84	3.39E-02	-0.38	5.89E-01	-0.53	2.25E-01
Phvul.007G186800	PTHR24362:SF255 - SERINE/THREONINE-PROTEIN KINASE NEK1 (1 of 2)	-0.51	5.69E-01	-1.66	2.48E-03	-0.77	3.70E-01	-1.46	1.12E-02
Phvul.009G047000	PF08879//PF08880 - WRC (WRC) // QLQ (QLQ) (1 of 10)	-0.51	4.00E-01	-1.01	1.47E-02	-0.52	4.35E-01	-0.73	1.01E-01
Phvul.002G234304	PTHR16166//PTHR16166:SF87 - VACUOLAR PROTEIN SORTING-ASSOCIATED PROTEIN VPS13 // SUBFAMILY NOT NAMED	-0.51	5.19E-01	1.27	1.43E-02	-1.50	2.40E-02	0.98	8.90E-02
Phvul.005G115650	no data	-0.51	1.77E-01	-0.67	2.15E-02	-0.54	1.84E-01	-0.70	1.97E-02
Phvul.011G030400	no data	-0.51	3.00E-01	-0.96	5.22E-03	-0.58	2.66E-01	-0.92	1.10E-02
Phvul.006G069700	PF00628//PF05641//PF16135 - PHD-finger (PHD) // Agenet domain (Agenet) // TPL-binding domain in jasmonate signa	-0.52	7.56E-02	-0.86	2.19E-04	-0.54	7.33E-02	-0.60	1.52E-02
Phvul.007G077700	PTHR11817:SF2 - PLASTIDIAL PYRUVATE KINASE 2 (1 of 2)	-0.52	1.24E-01	-0.77	3.21E-03	-0.56	1.10E-01	-0.51	7.39E-02
Phvul.010G003200	PF03140 - Plant protein of unknown function (DUF247) (1 of 36)	-0.52	2.30E-01	-1.09	4.30E-04	-0.35	4.99E-01	-0.79	1.61E-02
Phvul.002G019500	PTHR10265:SF25 - CYCLIN-DEPENDENT KINASE INHIBITOR 6 (1 of 2)	-0.52	5.88E-01	-1.75	3.10E-03	-0.55	5.95E-01	-1.22	5.15E-02
Phvul.006G177200	no data	-0.52	5.78E-01	-1.44	1.26E-02	-0.32	7.93E-01	-1.10	7.26E-02
Phvul.006G187600	PTHR35130:SF1 - MEDIATOR OF RNA POLYMERASE II TRANSCRIPTION SUBUNIT 16 (1 of 2)	-0.52	1.25E-02	-0.19	3.81E-01	-0.46	4.66E-02	-0.13	5.87E-01
Phvul.001G129500	PTHR11132//PTHR11132:SF116 - SOLUTE CARRIER FAMILY 35 // SUBFAMILY NOT NAMED (1 of 1)	-0.52	3.35E-01	-1.64	7.88E-06	-0.59	2.87E-01	-1.23	1.15E-03
Phvul.002G322900	PTHR10015:SF156 - HEAT STRESS TRANSCRIPTION FACTOR A-8 (1 of 1)	-0.52	2.85E-01	-0.79	2.83E-02	-0.22	7.55E-01	-0.41	2.95E-01
Phvul.009G020000	2.1.1.142 - Cycloartenol 24-C-methyltransferase / Sterol C-methyltransferase (1 of 1)	-0.52	2.28E-01	-0.66	4.08E-02	-0.09	9.05E-01	-0.45	1.97E-01
Phvul.009G182400	2.7.7.13 - Mannose-1-phosphate guanylyltransferase / GTP-mannose-1-phosphate guanylyltransferase (1 of 4)	-0.52	1.65E-01	-1.09	7.27E-05	-0.52	2.08E-01	-0.91	1.60E-03
Phvul.010G149800	PTHR24089:SF270 - SOLUTE CARRIER FAMILY 25 MEMBER 48 (1 of 2)	-0.52	5.02E-01	-1.48	2.47E-03	-0.25	8.17E-01	-1.05	4.41E-02
Phvul.002G021900	PTHR11709//PTHR11709:SF26 - MULTI-COPPER OXIDASE // SUBFAMILY NOT NAMED (1 of 2)	-0.52	5.26E-01	-1.48	4.14E-03	-0.65	4.30E-01	-1.13	3.71E-02
Phvul.002G254900	PTHR31852:SF47 - HYDROXYPROLINE-RICH GLYCOPROTEIN FAMILY PROTEIN (1 of 1)	-0.52	5.19E-01	-1.12	3.28E-02	-0.25	8.17E-01	-0.97	7.64E-02
Phvul.011G003200	PTHR27000:SF97 - LEUCINE-RICH RECEPTOR-LIKE PROTEIN KINASE (1 of 1)	-0.52	8.75E-02	-0.56	2.90E-02	-0.47	1.67E-01	-0.43	1.12E-01
Phvul.007G105332	PTHR10315//PTHR10315:SF28 - SEVEN IN ABSENTIA HOMOLOG // SUBFAMILY NOT NAMED (1 of 1)	-0.52	5.80E-01	-1.33	2.94E-02	-0.58	5.62E-01	-0.87	1.79E-01
Phvul.011G016500	no data	-0.52	1.81E-01	-1.07	1.87E-04	-0.50	2.55E-01	-0.96	1.28E-03
Phvul.002G041100	PTHR23160 - MYOSIN HEAVY CHAIN-RELATED (1 of 5)	-0.52	3.37E-01	-0.92	1.49E-02	-0.48	4.25E-01	-0.65	1.10E-01
Phvul.005G068500	PTHR13301:SF69 - BASIC HELIX-LOOP-HELIX DOMAIN-CONTAINING PROTEIN (1 of 3)	-0.52	7.73E-01	-2.41	1.21E-02	-0.29	9.04E-01	-1.83	7.16E-02
Phvul.007G163800	no data	-0.52	1.44E-01	-0.54	1.50E-01	-0.65	7.58E-02	-1.01	6.64E-03
Phvul.007G230700	PTHR22912:SF144 - MONODEHYDROASCORBATE REDUCTASE, CYTOPLASMIC ISOFORM 1-RELATED (1 of 1)	-0.52	2.83E-01	-0.78	3.57E-02	-0.12	8.77E-01	-0.27	5.45E-01
Phvul.010G148500	PTHR23503:SF19 - PLASTIDIC GLUCOSE TRANSPORTER 3-RELATED (1 of 1)	-0.52	8.71E-02	-0.72	3.91E-03	-0.36	3.49E-01	-0.85	9.47E-04
Phvul.001G197900	KOG1437 - Fasciclin and related adhesion glycoproteins (1 of 1)	-0.52	3.80E-01	-0.93	2.28E-02	-0.21	8.07E-01	-0.76	7.83E-02
Phvul.007G278600	PTHR22891:SF45 - PROTEIN ARGONAUTE 10 (1 of 2)	-0.52	8.04E-02	-0.54	3.51E-02	-0.65	2.90E-02	-0.28	3.14E-01
Phvul.011G156900	PTHR31072:SF3 - TRANSCRIPTION FACTOR TCP10-RELATED (1 of 3)	-0.53	3.08E-01	-1.09	2.02E-03	-0.34	5.97E-01	-0.83	2.92E-02
Phvul.002G270900	PTHR12565:SF74 - TRANSCRIPTION FACTOR HEC1 (1 of 2)	-0.53	3.96E-01	-0.98	2.39E-02	-0.40	5.85E-01	-0.45	3.65E-01
Phvul.003G034800	PTHR31917:SF9 - AGENET DOMAIN-CONTAINING PROTEIN / BROMO-ADJACENT HOMOLOGY (BAH) DOMAIN-CONTAINI	-0.53	2.49E-01	-0.75	3.51E-02	-0.59	2.27E-01	-0.71	5.42E-02
Phvul.003G025900	PTHR10106:SF20 - TRANSMEMBRANE ASCORBATE FERRIREDUCTASE 4-RELATED (1 of 1)	-0.53	4.90E-01	-1.43	3.49E-03	-0.28	7.81E-01	-1.17	2.39E-02
Phvul.006G134800	PTHR22847//PTHR22847:SF478 - WD40 REPEAT PROTEIN // SUBFAMILY NOT NAMED (1 of 4)	-0.53	7.51E-02	-0.43	9.09E-02	-0.60	4.39E-02	-0.40	1.29E-01
Phvul.010G040600	K15404 - aldehyde decarbonylase (K15404, CER1) (1 of 2)	-0.53	7.42E-01	-2.05	2.88E-02	-0.98	4.85E-01	-2.12	2.64E-02
Phvul.002G287700	PF04525 - LURP-one-related (LOR) (1 of 22)	-0.53	2.64E-01	-0.83	1.55E-02	-0.54	2.97E-01	-0.53	1.58E-01
Phvul.009G177400	no data	-0.53	5.09E-01	-1.39	2.28E-02	-0.70	3.89E-01	-0.37	5.88E-01
Phvul.003G175400	PTHR10766:SF54 - ENDOMEMBRANE FAMILY PROTEIN 70 (1 of 2)	-0.53	3.77E-01	-1.31	1.43E-03	-0.45	5.12E-01	-1.28	2.70E-03
Phvul.001G188100	PTHR24351:SF92 - PROTEIN KINASE-RELATED (1 of 2)	-0.53	2.95E-01	-0.87	1.68E-02	-0.47	4.05E-01	-0.77	4.35E-02
Phvul.010G113000	3.4.17.23 - Angiotensin-converting enzyme 2 / Angiotensin converting enzyme-2 (1 of 7)	-0.53	3.54E-01	-0.64	1.31E-01	-0.52	4.11E-01	-0.91	2.87E-02
Phvul.006G191900	PF07887 - Calmodulin binding protein-like (Calmodulin bind) (1 of 10)	-0.53	1.92E-01	-0.67	3.83E-02	-0.48	2.97E-01	-0.71	3.37E-02
Phvul.009G063100	4.2.1.75 - Uroporphyrinogen-III synthase / Uroporphyrinogen-III cosynthetase (1 of 1)	-0.53	1.72E-01	-1.06	2.62E-04	-0.47	2.96E-01	-0.72	2.03E-02
Phvul.002G268200	K13124 - mitogen-activated protein kinase organizer 1 (MORG1) (1 of 1)	-0.53	1.67E-01	-0.83	3.62E-03	-0.45	3.17E-01	-0.64	3.74E-02
Phvul.008G272600	PTHR19321//PTHR19321:SF11 - PROTEIN REGULATOR OF CYTOKINESIS 1 PRC1-RELATED // SUBFAMILY NOT NAMED (1 c	-0.53	2.53E-01	-1.06	1.51E-03	-0.45	4.06E-01	-0.89	1.23E-02
Phvul.003G253000	PTHR14255//PTHR14255:SF11 - ATP-DEPENDENT PROTEASE CEREBLON // SUBFAMILY NOT NAMED (1 of 2)	-0.53	2.28E-01	-0.84	1.71E-02	-0.45	3.73E-01	-1.13	1.57E-03
Phvul.002G175200	PTHR10209:SF206 - GIBBERELLIN OXIDASE-LIKE PROTEIN-RELATED (1 of 2)	-0.53	8.94E-02	-0.51	4.77E-02	-0.27	5.28E-01	-0.60	2.35E-02
Phvul.001G242100	PTHR21649:SF32 - CHLOROPHYLL A-B BINDING PROTEIN CP29.3, CHLOROPLASTIC (1 of 1)	-0.53	4.59E-01	-1.70	2.49E-04	-0.08	9.52E-01	-1.30	7.55E-03
Phvul.003G176100	PTHR10257//PTHR10257:SF13 - SERINE/THREONINE PROTEIN PHOSPHATASE 2A PP2A REGULATORY SUBUNIT B // SUB	-0.53	2.47E-02	-0.54	9.92E-03	-0.45	9.47E-02	-0.32	1.62E-01
Phvul.003G119900	KOG1987 - Speckle-type POZ protein SPOP and related proteins with TRAF, MATH and BTB/POZ domains (1 of 7)	-0.53	3.89E-01	-0.89	5.07E-02	-0.32	6.76E-01	-1.25	6.92E-03

Phvul.005G131000	K16298 - serine carboxypeptidase-like clade IV [EC:3.4.16.-] (SCPL-IV) (1 of 9)	-0.54	3.19E-01	-0.80	4.62E-02	-0.69	2.01E-01	-0.70	9.55E-02
Phvul.002G301600	no data	-0.54	1.84E-01	-0.88	3.73E-03	-0.41	3.88E-01	-0.38	2.66E-01
Phvul.003G035600	no data	-0.54	4.43E-01	-1.40	2.64E-03	-0.34	7.03E-01	-1.38	4.73E-03
Phvul.008G190500	PTHR12300:SF27 - HVA22-LIKE PROTEIN F (1 of 2)	-0.54	3.84E-01	-1.32	3.11E-03	-0.58	3.77E-01	-1.28	5.12E-03
Phvul.008G059200	K02931 - large subunit ribosomal protein L5 (RP-L5, MRPL5, rplE) (1 of 1)	-0.54	7.45E-02	-0.90	1.30E-04	-0.32	4.05E-01	-0.66	8.58E-03
Phvul.001G127700	PTHR23201//PTHR23201:SF8 - EXTENSIN, PROLINE-RICH PROTEIN // SUBFAMILY NOT NAMED (1 of 2)	-0.54	3.66E-01	-1.57	1.89E-04	-1.13	2.88E-02	-1.29	2.68E-03
Phvul.009G128500	PTHR22950:SF205 - AMINO ACID PERMEASE 3-RELATED (1 of 3)	-0.54	5.42E-01	-2.05	8.31E-04	-1.08	1.71E-01	-1.05	9.29E-02
Phvul.002G072500	PTHR13833:SF42 - NHL DOMAIN-CONTAINING PROTEIN (1 of 2)	-0.54	5.06E-01	-1.40	6.35E-03	-0.51	5.67E-01	-1.09	4.55E-02
Phvul.003G044800	no data	-0.54	5.36E-01	-1.85	3.44E-03	-0.50	6.07E-01	-0.91	1.44E-01
Phvul.007G204300	2.7.11.1 - Non-specific serine/threonine protein kinase / Threonine-specific protein kinase (1 of 1198)	-0.54	1.17E-01	-0.60	3.16E-02	-0.38	3.57E-01	-0.55	5.75E-02
Phvul.L005043	PTHR11937//PTHR11937:SF255 - ACTIN // SUBFAMILY NOT NAMED (1 of 4)	-0.54	3.94E-01	-1.26	3.55E-03	-0.22	8.13E-01	-1.00	2.74E-02
Phvul.006G093700	PTHR10252//PTHR10252:SF49 - HISTONE-LIKE TRANSCRIPTION FACTOR CCAAT-RELATED // SUBFAMILY NOT NAMED (1	-0.54	4.18E-01	-1.09	1.53E-02	-0.61	3.79E-01	-0.56	2.64E-01
Phvul.009G029900	no data	-0.54	2.83E-01	-1.30	9.44E-04	-0.29	6.61E-01	-0.76	6.19E-02
Phvul.011G161400	1.14.13.173 - 11-oxo-beta-amyrin 30-oxidase / CYP72A154 (1 of 17)	-0.54	5.12E-01	-0.04	9.66E-01	-1.72	1.85E-02	-0.11	9.01E-01
Phvul.005G085600	PTHR27003:SF37 - INTERLEUKIN-1 RECEPTOR-ASSOCIATED KINASE 4 PROTEIN-RELATED (1 of 2)	-0.54	2.56E-01	-0.78	2.73E-02	-0.70	1.45E-01	-0.52	1.74E-01
Phvul.009G114100	K07374 - tubulin alpha (TUBA) (1 of 7)	-0.54	1.86E-01	-0.64	4.35E-02	-0.21	7.39E-01	-0.50	1.42E-01
Phvul.001G155100	no data	-0.54	4.25E-01	-0.69	1.59E-01	-0.76	2.60E-01	-0.96	4.87E-02
Phvul.001G031000	PTHR31602:SF2 - GROWTH-REGULATING FACTOR 1-RELATED (1 of 1)	-0.54	1.03E-01	-0.80	2.44E-03	-0.46	2.32E-01	-0.65	2.00E-02
Phvul.011G146900	no data	-0.54	1.61E-01	-0.72	1.69E-02	-0.43	3.46E-01	-0.59	6.34E-02
Phvul.007G001900	PTHR12174:SF36 - SIGNAL PEPTIDE PEPTIDASE-LIKE 3-RELATED (1 of 2)	-0.54	1.43E-01	-0.78	3.34E-02	-0.33	4.78E-01	-0.37	3.45E-01
Phvul.L002157	PTHR24115:SF515 - KINESIN MOTOR PROTEIN-RELATED PROTEIN-RELATED (1 of 2)	-0.54	6.54E-02	-0.48	5.98E-02	-0.60	4.68E-02	-0.34	2.06E-01
Phvul.007G260900	K09338 - homeobox-leucine zipper protein (HD-ZIP) (1 of 50)	-0.54	3.50E-01	-0.93	3.25E-02	-0.47	4.76E-01	-0.61	1.83E-01
Phvul.006G187100	K04712 - sphingolipid delta-4 desaturase (DEGS) (1 of 1)	-0.54	4.04E-01	-1.49	9.99E-04	-0.46	5.39E-01	-1.02	3.04E-02
Phvul.009G102200	PTHR32044:SF13 - XYLOGLUCAN GLYCOSYLTRANSFERASE 5-RELATED (1 of 2)	-0.55	2.72E-01	-1.30	1.88E-04	-0.59	2.61E-01	-0.99	6.89E-03
Phvul.003G070800	PTHR11654:SF20 - PROTEIN NRT1/ PTR FAMILY 2.12-RELATED (1 of 4)	-0.55	3.50E-01	-1.31	9.05E-04	-0.67	2.60E-01	-1.07	1.03E-02
Phvul.003G010300	KOG0324 - Uncharacterized conserved protein (1 of 12)	-0.55	3.05E-01	-0.90	2.84E-02	-0.73	1.68E-01	-0.86	4.40E-02
Phvul.002G210000	KOG1546 - Metacaspase involved in regulation of apoptosis (1 of 9)	-0.55	2.68E-02	-0.20	4.42E-01	-0.20	5.98E-01	0.06	8.42E-01
Phvul.006G137811	PTHR27005:SF7 - WALL-ASSOCIATED RECEPTOR KINASE-LIKE 1-RELATED (1 of 6)	-0.55	2.83E-01	-1.22	8.00E-04	-0.61	2.55E-01	-1.14	2.47E-03
Phvul.010G013000	PTHR31623:SF15 - ACETYL-COA:BENZYLALCOHOL ACETYLTRANFERASE-LIKE PROTEIN-RELATED (1 of 4)	-0.55	4.38E-01	-1.34	5.06E-03	-0.66	3.59E-01	-0.42	4.68E-01
Phvul.009G008400	PTHR31407:SF17 - PSBP DOMAIN-CONTAINING PROTEIN 3, CHLOROPLASTIC (1 of 1)	-0.55	3.16E-01	-1.37	2.47E-04	-0.42	5.20E-01	-1.13	3.83E-03
Phvul.008G267200	PTHR31071:SF2 - F11F12.2 PROTEIN (1 of 2)	-0.55	1.22E-01	-0.67	2.00E-02	-0.51	2.04E-01	-0.46	1.40E-01
Phvul.005G159200	2.7.11.1 - Non-specific serine/threonine protein kinase / Threonine-specific protein kinase (1 of 1198)	-0.55	5.92E-01	-1.69	9.60E-03	-0.81	4.13E-01	-1.42	3.65E-02
Phvul.010G046400	6.1.1.6 - Lysine--tRNA ligase / Lysyl-tRNA synthetase (1 of 4)	-0.55	4.32E-02	-0.34	1.83E-01	-0.47	1.35E-01	-0.39	1.19E-01
Phvul.003G241200	PTHR10209:SF144 - IRON/ASCORBATE OXIDOREDUCTASE DDB G0283291-RELATED (1 of 1)	-0.55	6.79E-02	-0.69	9.40E-03	-0.38	2.97E-01	-0.50	7.37E-02
Phvul.008G007100	PTHR21726//PTHR21726:SF35 - PHOSPHATIDYLINOSITOL N-ACETYLGLUCOSAMINYLTRANSFERASE SUBUNIT P DOWN SY	-0.55	1.62E-01	-0.91	2.05E-03	-0.55	2.06E-01	-0.85	6.20E-03
Phvul.007G047300	K07374 - tubulin alpha (TUBA) (1 of 7)	-0.55	1.20E-01	-0.57	4.24E-02	-0.38	3.90E-01	-0.68	1.77E-02
Phvul.005G088900	PF00954//PF01453//PF07714//PF08276 - S-locus glycoprotein domain (S locus glycop) // D-mannose binding lectin (B le	-0.55	4.00E-02	-0.18	5.34E-01	-0.34	3.25E-01	-0.14	6.33E-01
Phvul.004G083100	K17805 - mitochondrial import inner membrane translocase subunit TIM16 (PAM16, TIM16) (1 of 1)	-0.55	1.41E-01	-0.90	1.61E-03	-0.49	2.59E-01	-0.82	6.23E-03
Phvul.001G188600	PTHR22835//PTHR22835:SF216 - ZINC FINGER FYVE DOMAIN CONTAINING PROTEIN // SUBFAMILY NOT NAMED (1 of 2)	-0.55	2.98E-02	-0.63	5.60E-03	-0.54	4.66E-02	-0.56	1.91E-02
Phvul.002G271400	K00888 - phosphatidylinositol 4-kinase (PI4K) (1 of 3)	-0.55	6.96E-01	-2.35	7.50E-03	-0.27	8.86E-01	-1.85	3.92E-02
Phvul.003G245700	PF04784//PF14389 - Protein of unknown function, DUF547 (DUF547) // Leucine-zipper of ternary complex factor MIP1	-0.55	3.84E-01	-1.57	2.91E-04	-0.62	3.48E-01	-1.23	6.31E-03
Phvul.006G133700	K08497 - protein transport protein SEC20 (SEC20) (1 of 2)	-0.55	1.30E-01	-0.80	4.39E-03	-0.43	3.25E-01	-0.58	5.54E-02
Phvul.001G214300	2.4.1.67 - Galactinol--raffinose galactosyltransferase / Stachyose synthetase (1 of 1)	-0.55	2.09E-01	-0.75	3.34E-02	-0.57	2.32E-01	-0.33	4.06E-01
Phvul.002G196050	no data	-0.55	4.34E-01	-1.13	1.72E-02	-0.85	2.12E-01	-0.52	3.29E-01
Phvul.001G086200	no data	-0.55	3.46E-01	-1.71	8.96E-05	-0.58	3.57E-01	-1.43	1.31E-03
Phvul.003G051200	no data	-0.56	2.67E-01	-0.76	3.87E-02	-0.37	5.52E-01	-0.88	1.82E-02
Phvul.008G010600	PTHR32227:SF54 - BETA-1, 3-GLUCANASE-LIKE PROTEIN-RELATED (1 of 1)	-0.56	4.23E-01	-1.19	1.04E-02	-0.20	8.45E-01	-1.04	3.43E-02
Phvul.006G159400	PTHR12304:SF6 - INOSINE-URIDINE PREFERRING NUCLEOSIDE HYDROLASE FAMILY PROTEIN (1 of 2)	-0.56	5.23E-01	-1.17	4.53E-02	-0.75	3.91E-01	-0.91	1.34E-01
Phvul.006G142900	no data	-0.56	5.30E-02	-0.55	2.07E-02	-0.41	2.42E-01	-0.50	4.67E-02
Phvul.007G188500	PTHR31358:SF4 - PROTEIN WAVE-DAMPENED 2-RELATED (1 of 2)	-0.56	2.61E-01	-1.00	6.04E-03	-0.51	3.59E-01	-0.80	3.73E-02
Phvul.007G145200	K11252 - histone H2B (H2B) (1 of 10)	-0.56	5.53E-01	-0.32	6.81E-01	-0.90	3.13E-01	-1.30	3.82E-02
Phvul.007G171333	PTHR11514:SF28 - TRANSCRIPTION FACTOR TT8 (1 of 2)	-0.56	1.53E-01	-0.65	3.68E-02	-0.28	5.89E-01	-0.35	3.07E-01
Phvul.002G198700	PTHR12863 - FATTY ACID HYDROXYLASE (1 of 2)	-0.56	3.19E-01	-1.18	2.64E-03	-0.38	5.85E-01	-0.70	1.01E-01
Phvul.004G032300	PTHR24056//PTHR24056:SF188 - CELL DIVISION PROTEIN KINASE // SUBFAMILY NOT NAMED (1 of 2)	-0.56	3.00E-01	-0.94	1.88E-02	-0.38	5.64E-01	-0.61	1.55E-01
Phvul.004G161500	no data	-0.56	4.68E-01	-1.22	1.51E-02	-0.38	6.99E-01	-1.04	5.13E-02
Phvul.010G116800	PTHR33472:SF1 - EXTENSIN-RELATED (1 of 2)	-0.56	7.02E-01	-2.75	5.91E-03	-0.87	5.42E-01	-1.34	1.77E-01
Phvul.002G297600	no data	-0.56	5.51E-01	-1.55	8.14E-03	-0.55	6.00E-01	-1.31	3.32E-02
Phvul.001G003800	PTHR31235:SF67 - PEROXIDASE 64 (1 of 2)	-0.56	6.76E-01	-1.76	6.02E-02	-1.00	4.39E-01	-2.67	7.80E-03
Phvul.004G038300	no data	-0.56	6.91E-02	-0.79	1.87E-03	-0.33	4.07E-01	-0.20	5.28E-01
Phvul.007G100966	PTHR11076:SF38 - DNA POLYMERASE ETA (1 of 2)	-0.56	1.82E-01	-0.79	2.18E-02	-0.47	3.27E-01	-0.35	3.53E-01
Phvul.007G252301	no data	-0.56	3.85E-01	-1.01	2.51E-02	-0.31	7.15E-01	-0.72	1.36E-01
Phvul.002G171000	PTHR11240:SF19 - F7A19.32 PROTEIN-RELATED (1 of 2)	-0.57	4.33E-01	-1.36	5.91E-03	-0.37	6.82E-01	-1.09	3.49E-02
Phvul.003G092500	PTHR31169:SF1 - ZINC-FINGER DOMAIN OF MONOAMINE-OXIDASE A REPRESSOR R1 PROTEIN (1 of 4)	-0.57	3.04E-01	-1.14	3.21E-03	-0.44	4.98E-01	-0.95	1.98E-02
Phvul.003G079100	PTHR12526//PTHR12526:SF341 - GLYCOSYLTRANSFERASE // SUBFAMILY NOT NAMED (1 of 2)	-0.57	3.05E-01	-0.89	2.63E-02	-0.52	4.04E-01	-0.90	2.93E-02
Phvul.L002081	PTHR19359//PTHR19359:SF10 - CYTOCHROME B5 // SUBFAMILY NOT NAMED (1 of 2)	-0.57	3.04E-01	-0.59	1.80E-01	-0.23	7.69E-01	-1.18	6.06E-03
Phvul.004G142800	PTHR12526:SF379 - SUCROSE SYNTHASE 5 (1 of 3)	-0.57	4.87E-02	-0.69	7.43E-03	-0.25	5.40E-01	-0.34	2.37E-01
Phvul.011G181800	PTHR10217//PTHR10217:SF520 - VOLTAGE AND LIGAND GATED POTASSIUM CHANNEL // SUBFAMILY NOT NAMED (1 of	-0.57	4.69E-02	-0.07	8.25E-01	-0.40	2.58E-01	-0.03	9.35E-01
Phvul.004G062900	no data	-0.57	5.52E-01	-1.45	1.55E-02	-0.49	6.58E-01	-1.09	8.57E-02
Phvul.007G168900	PTHR31447:SF2 - OXIDOREDUCTASE, 2OG-FE(II) OXYGENASE FAMILY PROTEIN (1 of 2)	-0.57	2.57E-02	-0.34	1.75E-01	-0.51	7.29E-02	-0.22	4.19E-01
Phvul.006G179500	PTHR22835//PTHR22835:SF218 - ZINC FINGER FYVE DOMAIN CONTAINING PROTEIN // SUBFAMILY NOT NAMED (1 of 1)	-0.57	7.06E-01	-2.18	1.13E-02	-0.94	5.02E-01	-1.34	1.44E-01
Phvul.008G216100	PTHR10795//PTHR10795:SF365 - PROPROTEIN CONVERTASE SUBTILISIN/KEXIN // SUBFAMILY NOT NAMED (1 of 4)	-0.57	4.10E-01	-1.20	2.65E-02	-0.83	2.29E-01	-0.63	2.73E-01
Phvul.003G010400	PF05055 - Protein of unknown function (DUF677) (DUF677) (1 of 9)	-0.57	2.57E-02	-0.17	5.72E-01	-0.55	4.83E-02	0.03	9.34E-01
Phvul.007G063900	PTHR10030:SF25 - ALPHA-L-FUCOSIDASE-RELATED (1 of 1)	-0.57	4.39E-01	-1.16	1.92E-02	-0.39	6.68E-01	-1.29	1.24E-02
Phvul.002G304500	PTHR16254:SF30 - K(+) EFFLUX ANTIPORTER 4 (1 of 2)	-0.57	2.11E-01	-0.50	1.97E-01	-0.32	5.87E-01	-0.75	4.76E-02
Phvul.011G187100	K11253 - histone H3 (H3) (1 of 11)	-0.58	5.55E-01	-0.42	5.92E-01	-1.07	2.38E-01	-1.61	1.49E-02
Phvul.010G126700	PTHR24078 - DNAJ HOMOLOG SUBFAMILY C MEMBER (1 of 123)	-0.58	1.90E-01	-0.65	5.66E-02	-0.56	2.54E-01	-0.78	2.38E-02
Phvul.006G207200	4.2.1.59 - 3-hydroxyacyl-[acyl-carrier-protein] dehydratase / D-3-hydroxyoctanoyl-[acyl carrier protein] dehydratase (1 c	-0.58	7.81E-02	-0.83	1.63E-03	-0.46	2.31E-01	-0.65	2.00E-02
Phvul.008G134300	PTHR12565//PTHR12565:SF99 - STEROL REGULATORY ELEMENT-BINDING PROTEIN // SUBFAMILY NOT NAMED (1 of 2)	-0.58	4.27E-01	-1.43	4.49E-03	-0.70	3.42E-01	-1.12	3.30E-02
Phvul.008G248000	PTHR31867:SF22 - EXPANSIN-A8 (1 of 3)	-0.58	7.37E-01	-2.56	1.10E-02	-0.76	6.68E-01	-1.69	1.12E-01
Phvul.009G040500	no data	-0.58	4.62E-01	-0.73	1.95E-01	-0.54	5.34E-01	-1.09	4.67E-02
Phvul.009G262100	PTHR10795:SF424 - SUBTILASE FAMILY PROTEIN-RELATED (1 of 2)	-0.58	4.45E-01	-1.24	1.47E-02	-0.36	7.14E-01	-0.95	7.87E-02
Phvul.011G057200	K01188 - beta-glucosidase (E3.2.1.21) (1 of 34)	-0.58	5.21E-01	-0.32	6.61E-01	-0.84	3.30E-01	-1.21	4.80E-02
Phvul.009G095000	3.1.1.5 - Lysophospholipase / Phospholipase B (1 of 5)	-0.58	5.49E-01	-2.24	2.45E-04	-0.36	7.71E-01	-1.42	2.29E-02
Phvul.001G004000	PTHR11695//PTHR11695:SF608 - ALCOHOL DEHYDROGENASE RELATED // SUBFAMILY NOT NAMED (1 of 1)	-0.58	5.11E-01	-1.68	2.82E-03	-0.51	6.13E-01	-1.43	1.58E-02
Phvul.003G231400	PF00069//PF00560//PF08263//PF13855 - Protein kinase domain (Pkinase) // Leucine Rich Repeat (LRR 1) // Leucine ric	-0.58	1.60E-01	-1.05	8.73E-04	-0.34	5.28E-01	-0.90	6.20E-03
Phvul.002G077400	PTHR13778//PTHR13778:SF9 - GLYCOSYLTRANSFERASE 8 DOMAIN-CONTAINING PROTEIN // SUBFAMILY NOT NAMED (1	-0.58	2.91E-01	-0.98	1.20E-02	-0.29	6.99E-01	-0.63	1.37E-01
Phvul.009G032300	2.5.1.54 - 3-deoxy-7-phosphoheptulonate synthase / Phospho-2-oxo-3-deoxyheptonate aldolase (1 of 3)	-0.58	4.18E-01	-1.85	3.13E-04	-0.33	7.20E-01	-1.58	2.72E-03
Phvul.011G099500	K14539 - large subunit GTPase 1 (LSG1) (1 of 1)	-0.58	3.00E-01	-0.83	4.43E-02	-0.44	5.10E-01	-0.57	2.02E-01
Phvul.003G159700	PF00069//PF08263//PF13855 - Protein kinase domain (Pkinase) // Leucine rich repeat N-terminal domain (LRRNT 2) //	-0.59	8.64E-02	-0.78	4.02E-03	-0.21	6.85E-01	-0.29	3.67E-01
Phvul.006G164300	PTHR10217//PTHR10217:SF520 - VOLTAGE AND LIGAND GATED POTASSIUM CHANNEL // SUBFAMILY NOT NAMED (1 of	-0.59	4.70E-01	-1.20	2.99E-02	-0.66	4.42E-01	-0.65	2.83E-01
Phvul.008G241300	PTHR12526:SF379 - SUCROSE SYNTHASE 5 (1 of 3)	-0.59	1.22E-01	-0.71	2.70E-02	-0.37	4.34E-01	-0.59	7.71E-02
Phvul.010G153100	no data	-0.59	4.71E-01	-1.06	5.42E-02	-0.41	6.82E-01	-1.41	1.23E-02
Phvul.002G029400	PTHR14255//PTHR14255:SF11 - ATP-DEPENDENT PROTEASE CEREBLON // SUBFAMILY NOT NAMED (1 of 2)	-0.59	2.26E-02	-0.64	6.51E-03	-0.46	1.30E-01	-0.48	5.19E-02
Phvul.010G075300	PTHR33601:SF1 - LITTLE ZIPPER 1 PROTEIN-RELATED (1 of 2)	-0.59	1.38E-01	-0.95	2.20E-03	-0.28	6.03E-01	-0.43	2.19E-01
Phvul.003G235600	PTHR23213//PTHR23213:SF215 - FORMIN-RELATED // SUBFAMILY NOT NAMED (1 of 2)	-0.59	3.25E-02	-0.71	5.07E-03	-0.25	5.22E-01	-0.50	5.69E-02
Phvul.002G140400	PTHR12510:SF7 - AIG2-LIKE (AVIRULENCE INDUCED GENE) FAMILY PROTEIN (1 of 1)	-0.59	3.68E-01	-1.22	6.77E-03	-0.58	4.23E-01	-0.99	3.80E-02
Phvul.008G109001	PF00560//PF13855 - Leucine Rich Repeat (LRR 1) // Leucine rich repeat (LRR 8) (1 of 35)	-0.59	3.61E-01	-0.65	1.80E-01	-0.46	5.44E-01	-1.06	2.51E-02
Phvul.003G043700	K08827 - serine/threonine-protein kinase PRP4 [EC:2.7.11.1] (PRPF4B) (1 of 2)	-0.59	3.52E-01	-0.93	3.82E-02	-0.45	5.47E-01	-0.89	5.45E-02
Phvul.003G217800	PTHR11709:SF82 - MONOCOPPER OXIDASE-LIKE PROTEIN SKU5 (1 of 2)	-0.59	5.50E-01	-1.78	3.14E-03	-0.42	7.35E-01	-1.40	2.83E-02
Phvul.002G123300	PTHR33101:SF2 - ROP GUANINE NUCLEOTIDE EXCHANGE FACTOR 14 (1 of 2)	-0.60	4.33E-01	-1.59	1.43E-03	-0.64	4.25E-01	-1.29	1.36E-02
Phvul.008G039800	PTHR24056//PTHR24056:SF188 - CELL DIVISION PROTEIN KINASE // SUBFAMILY NOT NAMED (1 of 2)	-0.60	5.71E-01	-0.84	2.52E-01	0.15	9.25E-01	-1.46	4.40E-02
Phvul.007G001100	PTHR11071:SF11 - PEPTIDYL-PROLYL CIS-TRANS ISOMERASE C (1 of 1)	-0.60	9.26E-02	-0.92	8.28E-04	-0.45	2.90E-01	-0.86	2.80E-03
Phvul.007G139400	3.2.1.142 - Limit dextrinase / R-enzyme (1 of 2)	-0.60	8.50E-02	-0.88	1.31E-03	-0.55	1.57E-01	-0.72	1.26E-02

Phvul.006G029000	PTHR13439//PTHR13439:SF17 - CT120 PROTEIN // SUBFAMILY NOT NAMED (1 of 2)	-0.60	5.62E-02	-0.54	4.94E-02	-0.47	2.02E-01	-0.37	2.12E-01
Phvul.011G191600	PF13191//PF13855 - AAA ATPase domain (AAA 16) // Leucine rich repeat (LRR 8) (1 of 1)	-0.60	3.96E-02	-0.03	9.38E-01	-0.65	3.26E-02	-0.18	5.65E-01
Phvul.008G087300	PTHR32444:SF6 - D-MANNOSE BINDING LECTIN PROTEIN WITH APPLE-LIKE CARBOHYDRATE-BINDING DOMAIN (1 of 1)	-0.60	1.65E-01	-0.88	2.72E-02	-0.11	8.84E-01	-0.50	2.33E-01
Phvul.009G209100	PTHR10766:SF54 - ENDOMEMBRANE FAMILY PROTEIN 70 (1 of 2)	-0.60	1.64E-01	-0.97	3.10E-03	-0.54	2.71E-01	-0.70	4.46E-02
Phvul.004G080600	PTHR10566//PTHR10566:SF83 - CHAPERONE-ACTIVITY OF BC1 COMPLEX CABC1 -RELATED // SUBFAMILY NOT NAMED (	-0.60	4.87E-01	-1.23	3.00E-02	-0.82	3.34E-01	-1.26	3.37E-02
Phvul.011G150950	PF00954//PF01453//PF07714//PF08276//PF11883 - S-locus glycoprotein domain (S locus glycop) // D-mannose binding	-0.60	3.54E-01	-1.58	4.71E-04	-0.74	2.64E-01	-1.44	2.11E-03
Phvul.009G254200	PTHR34804:SF1 - CAMP-REGULATED PHOSPHOPROTEIN 19-RELATED PROTEIN (1 of 1)	-0.60	1.80E-01	-0.83	1.92E-02	-0.35	5.32E-01	-0.70	5.94E-02
Phvul.003G070700	PTHR11654:SF20 - PROTEIN NRT1/ PTR FAMILY 2.12-RELATED (1 of 4)	-0.60	2.90E-01	-1.00	1.53E-02	-0.38	5.94E-01	-0.63	1.58E-01
Phvul.004G072000	PTHR31942:SF29 - MLO-LIKE PROTEIN 12-RELATED (1 of 3)	-0.60	9.00E-02	-0.62	3.84E-02	-0.36	4.23E-01	-0.59	5.63E-02
Phvul.002G128400	PTHR23083:SF39 - TETRATRICOPEPTIDE REPEAT PROTEIN 8 (1 of 1)	-0.60	1.30E-01	-0.76	1.99E-02	-0.70	9.04E-02	-0.63	6.66E-02
Phvul.009G024000	PTHR32227:SF16 - GLUCAN ENDO-1,3-BETA-GLUCOSIDASE 7-RELATED (1 of 2)	-0.60	7.67E-02	-0.91	7.22E-04	-0.41	3.27E-01	-0.79	5.40E-03
Phvul.002G111200	PTHR24115:SF496 - ATP BINDING MICROTUBULE MOTOR FAMILY PROTEIN (1 of 1)	-0.60	4.78E-01	-1.40	1.11E-02	-0.55	5.64E-01	-0.98	9.84E-02
Phvul.001G165332	PTHR11062:SF56 - XYLOGLUCAN GALACTOSYLTRANSFERASE KATAMARI1 (1 of 2)	-0.61	1.65E-01	-0.76	2.87E-02	-0.42	4.38E-01	-0.59	1.06E-01
Phvul.011G045600	PTHR22572:SF111 - GLUCOSE-1-PHOSPHATE ADENYLYLTRANSFERASE LARGE SUBUNIT 3, CHLOROPLASTIC-RELATED (1 o	-0.61	4.18E-02	-0.18	5.64E-01	-0.49	1.53E-01	-0.22	4.63E-01
Phvul.010G087100	PTHR32116:SF4 - POLYGALACTURONATE 4-ALPHA-GALACTURONOSYLTRANSFERASE-RELATED (1 of 2)	-0.61	3.22E-01	-0.98	4.23E-02	-0.61	3.63E-01	-0.46	3.85E-01
Phvul.007G060200	PTHR11200:SF174 - POLYPHOSPHATE 5'-PHOSPHATASE-RELATED (1 of 2)	-0.61	1.81E-01	-0.79	2.69E-02	-0.42	4.35E-01	-0.45	2.36E-01
Phvul.011G100600	PTHR23024//PTHR23024:SF189 - MEMBER OF 'GDXG' FAMILY OF LIPOLYTIC ENZYMES // SUBFAMILY NOT NAMED (1 of	-0.61	3.29E-01	-1.01	2.00E-02	-0.44	5.53E-01	-0.94	4.04E-02
Phvul.003G035500	PTHR31769:SF5 - EXPRESSED PROTEIN (1 of 1)	-0.61	3.45E-01	-0.68	1.65E-01	-0.55	4.48E-01	-1.11	1.88E-02
Phvul.002G155900	KOG0978//KOG0993 - E3 ubiquitin ligase involved in syntaxin degradation // Rab5 GTPase effector Rabaptin-5 (1 of 2)	-0.61	6.28E-02	-0.62	2.36E-02	-0.59	9.65E-02	-0.51	7.74E-02
Phvul.007G217400	K10406 - kinesin family member C2/C3 (KIFC2 3) (1 of 13)	-0.61	1.58E-01	-1.07	2.43E-03	-0.66	1.53E-01	-0.71	5.67E-02
Phvul.009G016100	no data	-0.61	3.33E-01	-1.46	7.22E-04	-0.48	5.18E-01	-1.15	1.12E-02
Phvul.005G046500	PF12796 - Ankyrin repeats (3 copies) (Ank 2) (1 of 93)	-0.61	2.52E-02	-0.25	3.94E-01	-0.34	3.43E-01	-0.24	4.09E-01
Phvul.011G099900	PTHR11306//PTHR11306:SF13 - NIEMANN PICK TYPE C2 PROTEIN NPC2-RELATED // SUBFAMILY NOT NAMED (1 of 4)	-0.61	4.13E-01	-1.79	5.07E-04	-0.43	6.34E-01	-1.06	5.19E-02
Phvul.001G216700	PTHR11964:SF18 - S-ADENOSYLMETHIONINE SYNTHASE 2 (1 of 3)	-0.61	6.51E-02	-0.77	3.83E-03	-0.45	2.61E-01	-0.56	5.30E-02
Phvul.003G223300	PF05553 - Cotton fibre expressed protein (DUF761) (1 of 36)	-0.61	2.90E-02	-0.39	1.47E-01	-0.58	5.59E-02	-0.09	7.82E-01
Phvul.002G019900	PF00685 - Sulfotransferase domain (Sulfotransfer 1) (1 of 13)	-0.61	9.39E-02	-0.59	6.13E-02	-0.64	1.03E-01	-0.63	4.85E-02
Phvul.008G221800	PTHR31087:SF17 - PROTEIN LURP-ONE-RELATED 14-RELATED (1 of 1)	-0.61	4.48E-01	-1.34	1.66E-02	-0.33	7.55E-01	-0.65	2.77E-01
Phvul.011G138400	PF06764 - Protein of unknown function (DUF1223) (DUF1223) (1 of 1)	-0.61	3.45E-02	-0.01	9.73E-01	-0.45	2.07E-01	0.16	6.17E-01
Phvul.002G162500	PTHR31194//PTHR31194:SF16 - SHN SHINE , DNA BINDING / TRANSCRIPTION FACTOR // SUBFAMILY NOT NAMED (1 of	-0.61	5.54E-01	-2.29	8.94E-04	-0.77	4.78E-01	-1.93	6.23E-03
Phvul.001G012100	PTHR10795:SF316 - SERINE PROTEASE-LIKE PROTEIN-RELATED (1 of 2)	-0.61	3.17E-01	-1.57	1.79E-03	-0.58	3.93E-01	-1.10	2.91E-02
Phvul.010G052000	no data	-0.62	4.38E-02	-0.74	4.37E-03	-0.54	1.16E-01	-0.47	9.04E-02
Phvul.005G022100	PTHR13301//PTHR13301:SF61 - X-BOX TRANSCRIPTION FACTOR-RELATED // SUBFAMILY NOT NAMED (1 of 1)	-0.62	8.44E-02	-0.65	2.51E-02	-0.53	2.05E-01	-0.37	2.56E-01
Phvul.011G034700	PTHR31490:SF2 - GLYCOSYL HYDROLASE FAMILY 10 PROTEIN (1 of 1)	-0.62	2.98E-01	-1.11	7.53E-03	-0.28	7.41E-01	-0.83	6.36E-02
Phvul.004G014000	K05579 - NAD(P)H-quinone oxidoreductase subunit H (ndhH) (1 of 1)	-0.62	3.08E-01	-1.40	9.39E-04	-0.64	3.25E-01	-1.03	2.18E-02
Phvul.004G001000	no data	-0.62	2.90E-01	-1.18	4.75E-03	-0.36	6.32E-01	-0.78	8.38E-02
Phvul.004G081300	K12622 - U6 snRNA-associated Sm-like protein LSm3 (LSM3) (1 of 2)	-0.62	4.19E-01	-1.45	1.24E-02	-0.45	6.23E-01	-0.81	1.84E-01
Phvul.008G147900	PTHR22939:SF67 - PROTEASE DO-LIKE 9 (1 of 1)	-0.62	1.84E-01	-0.91	1.22E-02	-0.49	3.74E-01	-0.44	2.76E-01
Phvul.004G146200	PTHR31948:SF16 - ZINC-FINGER HOMEODOMAIN PROTEIN 14 (1 of 2)	-0.62	2.49E-01	-1.22	2.17E-03	-0.45	4.85E-01	-1.10	8.21E-03
Phvul.006G127900	K00020 - 3-hydroxyisobutyrate dehydrogenase (E1.1.1.31, mmsB) (1 of 1)	-0.62	3.04E-01	-1.05	1.91E-02	-0.58	3.82E-01	-0.99	3.38E-02
Phvul.003G291500	K14494 - DELLA protein (DELLA) (1 of 5)	-0.62	7.62E-03	-0.25	3.01E-01	-0.30	3.52E-01	-0.11	6.96E-01
Phvul.003G275200	PTHR21461:SF12 - F21O3.9 PROTEIN-RELATED (1 of 2)	-0.62	3.92E-01	-1.51	2.11E-03	-0.43	6.31E-01	-0.95	7.26E-02
Phvul.005G064500	PTHR28039:SF6 - ALTERED INHERITANCE OF MITOCHONDRIA PROTEIN 18, MITOCHONDRIAL-RELATED (1 of 2)	-0.62	1.68E-01	-1.26	5.15E-04	-0.65	1.86E-01	-0.81	3.36E-02
Phvul.001G034100	PTHR11034:SF23 - N-MYC DOWNREGULATED-LIKE 3 PROTEIN (1 of 4)	-0.62	3.53E-01	-1.29	4.86E-03	-0.49	5.29E-01	-1.12	2.04E-02
Phvul.001G225700	PF07816 - Protein of unknown function (DUF1645) (DUF1645) (1 of 14)	-0.62	2.34E-01	-0.97	1.59E-02	-0.19	8.13E-01	-0.26	5.79E-01
Phvul.011G212100	PTHR27000:SF151 - DNA-DAMAGE-REPAIR/TOLERATION PROTEIN DRT100-RELATED (1 of 4)	-0.62	5.78E-01	-0.87	2.46E-01	-0.79	4.76E-01	-1.76	1.60E-02
Phvul.002G194800	PF04481 - Protein of unknown function (DUF561) (DUF561) (1 of 1)	-0.62	4.57E-02	-0.67	9.79E-03	-0.63	5.54E-02	-0.62	2.32E-02
Phvul.008G289600	PTHR10766:SF44 - ENDOMEMBRANE FAMILY PROTEIN 70 (1 of 2)	-0.62	2.12E-01	-1.22	8.68E-04	-0.54	3.48E-01	-0.94	1.55E-02
Phvul.009G160600	PTHR17130//PTHR17130:SF25 - MITOCHONDRIAL OUTER MEMBRANE PROTEIN 25 // SUBFAMILY NOT NAMED (1 of 2)	-0.63	5.62E-01	-1.59	2.14E-02	-0.04	9.88E-01	-1.45	4.39E-02
Phvul.010G129900	PTHR31238:SF21 - GERMIN-LIKE PROTEIN SUBFAMILY 3 MEMBER 3 (1 of 3)	-0.63	7.50E-01	-2.94	7.14E-03	-0.60	7.92E-01	-2.24	4.87E-02
Phvul.003G237500	no data	-0.63	4.22E-01	-1.07	8.38E-02	-0.32	7.63E-01	-1.31	3.70E-02
Phvul.007G260000	PTHR11937//PTHR11937:SF255 - ACTIN // SUBFAMILY NOT NAMED (1 of 4)	-0.63	2.49E-01	-1.11	7.50E-03	-0.41	5.39E-01	-0.92	3.37E-02
Phvul.007G093300	PTHR11802:SF71 - SERINE CARBOXYPEPTIDASE-LIKE 26-RELATED (1 of 1)	-0.63	3.16E-01	-1.23	5.06E-03	-0.65	3.36E-01	-0.97	3.72E-02
Phvul.002G150400	PTHR10638//PTHR10638:SF34 - COPPER AMINE OXIDASE // SUBFAMILY NOT NAMED (1 of 4)	-0.63	1.79E-02	-0.25	3.56E-01	-0.41	2.26E-01	-0.03	9.40E-01
Phvul.003G110900	PF06749 - Protein of unknown function (DUF1218) (DUF1218) (1 of 14)	-0.63	5.11E-01	-2.08	4.31E-03	-0.34	7.88E-01	-1.66	2.32E-02
Phvul.010G147100	PTHR19139:SF99 - AQUAPORIN TIP2-1 (1 of 2)	-0.63	1.07E-01	-0.75	1.57E-02	-0.45	3.46E-01	-0.55	9.69E-02
Phvul.007G186500	K18266 - protein NDRG1 (NDRG1) (1 of 1)	-0.63	5.23E-01	-1.38	2.90E-02	-0.29	8.33E-01	-1.15	8.16E-02
Phvul.005G083900	PF06749 - Protein of unknown function (DUF1218) (DUF1218) (1 of 14)	-0.63	1.76E-01	-1.22	4.40E-04	-0.48	3.86E-01	-0.88	1.77E-02
Phvul.004G020100	PTHR31517:SF11 - PEROXIDASE 31-RELATED (1 of 2)	-0.64	3.50E-01	-1.21	9.62E-03	-0.24	8.15E-01	-1.00	4.40E-02
Phvul.010G063300	PTHR19317:SF9 - PRA1 FAMILY PROTEIN B1-RELATED (1 of 4)	-0.64	2.77E-01	-1.39	1.17E-03	-0.33	6.79E-01	-1.17	8.67E-03
Phvul.006G048500	PTHR19321//PTHR19321:SF11 - PROTEIN REGULATOR OF CYTOKINESIS 1 PRC1-RELATED // SUBFAMILY NOT NAMED (1 (	-0.64	1.40E-01	-1.06	1.65E-03	-0.55	2.71E-01	-0.94	7.52E-03
Phvul.011G049400	K07399 - cytochrome c biogenesis protein (resB, ccs1) (1 of 1)	-0.64	3.27E-01	-1.16	1.00E-02	-0.93	1.32E-01	-1.00	3.42E-02
Phvul.006G144300	no data	-0.64	2.47E-01	-1.23	2.16E-03	-0.54	3.96E-01	-0.99	1.85E-02
Phvul.003G053100	PTHR30540:SF4 - POTASSIUM TRANSPORTER 12-RELATED (1 of 1)	-0.64	3.90E-01	-1.25	1.84E-02	-0.67	4.05E-01	-1.30	1.78E-02
Phvul.006G146100	PTHR27001:SF21 - PROTEIN STRUBBELIG-RECEPTOR FAMILY 6-RELATED (1 of 2)	-0.64	2.64E-01	-1.02	1.56E-02	-0.58	3.64E-01	-0.82	6.65E-02
Phvul.008G155300	no data	-0.64	1.24E-01	-0.96	2.97E-03	-0.58	2.13E-01	-0.69	4.51E-02
Phvul.009G252200	PTHR31339:SF15 - PECTIN LYASE-LIKE SUPERFAMILY PROTEIN-RELATED (1 of 2)	-0.64	1.22E-01	-0.89	5.48E-03	-0.70	1.07E-01	-0.84	1.29E-02
Phvul.008G279900	PF05701 - Weak chloroplast movement under blue light (WEMBL) (1 of 16)	-0.64	9.35E-02	-0.89	3.45E-03	-0.63	1.30E-01	-0.90	4.47E-03
Phvul.002G308900	PTHR12726 - CERAMIDE GLUCOSYLTRANSFERASE (1 of 2)	-0.64	3.47E-01	-1.54	2.26E-03	-0.62	4.04E-01	-1.19	2.16E-02
Phvul.008G255800	PTHR32044:SF13 - XYLOGLUCAN GLYCOSYLTRANSFERASE 5-RELATED (1 of 2)	-0.64	1.97E-01	-1.21	8.58E-04	-0.65	2.26E-01	-0.89	2.18E-02
Phvul.001G173700	PF03106 - WRKY DNA -binding domain (WRKY) (1 of 91)	-0.64	2.69E-02	-0.26	3.77E-01	-0.51	1.35E-01	-0.15	6.31E-01
Phvul.008G136800	no data	-0.64	4.22E-02	-0.58	3.94E-02	-0.71	2.80E-02	-0.42	1.57E-01
Phvul.003G078300	no data	-0.64	2.09E-01	-0.90	1.90E-02	-0.08	9.39E-01	-0.70	8.39E-02
Phvul.008G146900	PTHR31374:SF56 - AUXIN-REGULATED PROTEIN-RELATED (1 of 1)	-0.64	5.27E-01	-1.83	8.36E-03	-0.29	8.33E-01	-1.65	2.08E-02
Phvul.009G032000	KOG0149 - Predicted RNA-binding protein SEB4 (RRM superfamily) (1 of 8)	-0.64	4.20E-01	-1.27	2.78E-02	-0.99	1.96E-01	-0.99	1.02E-01
Phvul.010G005800	PTHR11709:SF9 - LACCASE-7-RELATED (1 of 3)	-0.64	7.83E-01	-4.23	2.66E-03	-0.85	7.36E-01	-2.45	7.71E-02
Phvul.003G051300	PTHR10795//PTHR10795:SF362 - PROPROTEIN CONVERTASE SUBTILISIN/KEXIN // SUBFAMILY NOT NAMED (1 of 1)	-0.64	2.00E-01	-1.09	3.62E-03	-0.59	2.90E-01	-1.22	1.60E-03
Phvul.002G167200	PTHR23421:SF79 - BETA-GALACTOSIDASE 3 (1 of 2)	-0.64	2.79E-01	-1.26	2.35E-03	-0.54	4.27E-01	-1.06	1.61E-02
Phvul.007G280600	PF14577 - Sieve element occlusion C-terminus (SEO C) (1 of 14)	-0.64	1.73E-01	-0.74	4.74E-02	-0.25	7.15E-01	-0.38	3.62E-01
Phvul.011G026400	PTHR31692:SF4 - EXPANSIN-LIKE A1-RELATED (1 of 1)	-0.64	4.36E-01	-1.27	3.05E-02	-0.50	6.14E-01	-1.41	2.04E-02
Phvul.008G021200	PF14416 - PMR5 N terminal Domain (PMR5N) (1 of 64)	-0.64	8.46E-02	-0.65	3.07E-02	-0.62	1.32E-01	-0.70	2.43E-02
Phvul.009G092200	2.4.2.7 - Adenine phosphoribosyltransferase / Transphosphoribosidase (1 of 4)	-0.64	3.88E-01	-1.03	4.66E-02	-0.29	7.78E-01	-1.03	5.38E-02
Phvul.011G063800	PTHR12537:SF78 - PUMILIO HOMOLOG 1-RELATED (1 of 3)	-0.64	6.46E-01	-3.65	1.54E-05	-1.00	4.44E-01	-2.75	1.11E-03
Phvul.001G209400	PTHR31707:SF25 - PECTINESTERASE/PECTINESTERASE INHIBITOR 39-RELATED (1 of 2)	-0.64	5.69E-01	-2.10	2.81E-03	-0.56	6.65E-01	-1.81	1.36E-02
Phvul.002G107900	PF00314 - Thaumatin family (Thaumatin) (1 of 26)	-0.65	4.69E-01	-1.47	1.15E-02	-0.35	7.66E-01	-1.40	2.04E-02
Phvul.005G184800	PTHR31288:SF5 - GENOMIC DNA, CHROMOSOME 3, P1 CLONE: MXL8-RELATED (1 of 2)	-0.65	2.45E-01	-1.20	2.77E-03	-0.60	3.36E-01	-0.96	2.35E-02
Phvul.001G038400	PTHR13140:SF304 - MYOSIN IA HEAVY CHAIN-RELATED (1 of 6)	-0.65	3.08E-01	-1.10	1.88E-02	-0.76	2.46E-01	-0.49	3.58E-01
Phvul.006G134200	K14724 - solute carrier family 9 (sodium/hydrogen exchanger), member 8 (SLC9A8, NHE8) (1 of 2)	-0.65	4.97E-01	-1.59	9.60E-03	-0.61	5.60E-01	-0.92	1.68E-01
Phvul.009G240100	2.7.11.1 - Non-specific serine/threonine protein kinase / Threonine-specific protein kinase (1 of 1198)	-0.65	3.84E-01	-1.13	3.41E-02	-0.49	5.79E-01	-1.10	4.39E-02
Phvul.001G134600	PF00560//PF07714//PF13855 - Leucine Rich Repeat (LRR 1) // Protein tyrosine kinase (Pkinase Tyr) // Leucine rich repe	-0.65	3.38E-01	-1.39	8.16E-03	-1.09	7.96E-02	-0.93	8.93E-02
Phvul.011G072000	K04733 - interleukin-1 receptor-associated kinase 4 (IRAK4) (1 of 48)	-0.65	5.21E-01	-1.93	2.86E-03	-0.57	6.19E-01	-1.31	5.79E-02
Phvul.007G209200	PF12708 - Pectate lyase superfamily protein (Pectate lyase 3) (1 of 32)	-0.65	6.42E-02	-0.73	1.50E-02	-0.50	2.29E-01	-0.50	1.17E-01
Phvul.009G109600	PTHR31985:SF23 - DEHYDRATION-RESPONSIVE ELEMENT-BINDING PROTEIN 3-RELATED (1 of 3)	-0.65	3.92E-01	-1.33	9.42E-03	-0.45	6.27E-01	-1.01	6.13E-02
Phvul.004G173101	PF06376 - Protein of unknown function (DUF1070) (DUF1070) (1 of 7)	-0.65	4.88E-01	-1.38	3.90E-02	-0.50	6.55E-01	-1.19	8.56E-02
Phvul.003G053300	2.4.1.15//3.1.3.12 - Alpha,alpha-trehalose-phosphate synthase (UDP-forming) / UDP-glucose--glucose-phosphate glucos	-0.65	1.58E-01	-1.00	6.51E-03	-0.75	1.16E-01	-1.12	3.14E-03
Phvul.006G189400	no data	-0.65	3.17E-01	-1.32	5.06E-03	-0.46	5.50E-01	-1.04	3.62E-02
Phvul.006G107300	PTHR31745:SF1 - SINGLE-STRANDED DNA-BINDING PROTEIN WHY2, MITOCHONDRIAL (1 of 1)	-0.65	7.16E-01	-3.03	3.10E-03	-1.02	5.45E-01	-2.56	1.63E-02
Phvul.007G095300	PTHR10071:SF194 - GATA TRANSCRIPTION FACTOR 8 (1 of 2)	-0.65	2.35E-01	-1.15	3.65E-03	-0.51	4.32E-01	-0.99	1.77E-02
Phvul.008G116600	PTHR12725//PTHR12725:SF68 - HALOACID DEHALOGENASE-LIKE HYDROLASE // SUBFAMILY NOT NAMED (1 of 1)	-0.65	8.62E-02	-0.53	1.61E-01	-0.80	3.59E-02	-0.24	5.67E-01
Phvul.003G203000	PF14365 - Domain of unknown function (DUF4409) (DUF4409) (1 of 17)	-0.66	3.06E-02	-0.84	1.46E-03	-0.60	7.14E-02	-0.55	5.19E-02
Phvul.008G220700	PTHR33232:SF1 - F12A21.8 (1 of 2)	-0.66	1.08E-01	-0.75	4.18E-02	-0.47	3.37E-01	-0.28	5.04E-01
Phvul.008G292700	no data	-0.66	2.28E-01	-0.95	1.80E-02	-0.29	7.11E-01	-0.62	1.54E-01
Phvul.011G048600	PTHR10994//PTHR10994:SF79 - RETICULON // SUBFAMILY NOT NAMED (1 of 1)	-0.66	1.23E-01	-1.05	1.29E-03	-0.51	3.15E-01	-1.05	2.30E-03
Phvul.010G110900	PTHR23213//PTHR23213:SF215 - FORMIN-RELATED // SUBFAMILY NOT NAMED (1 of 2)	-0.66	3.43E-01	-1.47	1.71E-03	-0.55	4.85E-01	-1.16	1.91E-02

Phvul.008G166600	PF00069//PF08263//PF13855 - Protein kinase domain (Pkinase) // Leucine rich repeat N-terminal domain (LRRNT 2) //	-0.66	6.08E-02	-1.39	1.12E-05	-0.51	2.16E-01	-1.27	4.54E-05
Phvul.008G210100	K12394 - AP-1 complex subunit sigma 1/2 (AP1S1 2) (1 of 3)	-0.66	4.97E-01	-1.69	5.82E-03	-0.45	7.11E-01	-1.38	3.28E-02
Phvul.009G015700	PTHR12081:SF51 - TRANSCRIPTION FACTOR E2FC (1 of 1)	-0.66	9.05E-02	-0.95	2.16E-03	-0.53	2.52E-01	-0.65	5.27E-02
Phvul.005G092700	PF02298 - Plastocyanin-like domain (Cu bind like) (1 of 53)	-0.66	3.61E-02	-0.82	2.86E-03	-0.20	6.88E-01	-0.40	1.93E-01
Phvul.003G238600	K12347 - natural resistance-associated macrophage protein (SLC11A, NRAMP) (1 of 4)	-0.66	4.89E-02	-0.75	6.43E-03	-0.57	1.42E-01	-0.76	8.16E-03
Phvul.001G216600	PTHR11964:SF18 - S-ADENOSYLMETHIONINE SYNTHASE 2 (1 of 3)	-0.66	3.00E-01	-1.12	1.17E-02	-0.46	5.53E-01	-0.98	3.74E-02
Phvul.008G157100	no data	-0.66	3.64E-01	-1.13	4.56E-02	-0.53	5.24E-01	-0.37	5.70E-01
Phvul.002G277600	no data	-0.66	4.36E-01	-1.29	3.02E-02	-0.54	5.88E-01	-1.34	2.88E-02
Phvul.007G074000	PF07714//PF11721 - Protein tyrosine kinase (Pkinase Tyr) // Di-glucose binding within endoplasmic reticulum (Malectin	-0.66	1.72E-02	-0.36	1.88E-01	-0.62	4.26E-02	-0.32	2.45E-01
Phvul.001G176600	PTHR33597:SF4 - CONSERVED PEPTIDE UPSTREAM OPEN READING FRAME 47 (1 of 2)	-0.66	3.52E-01	-1.57	1.24E-03	-0.27	7.95E-01	-0.91	8.12E-02
Phvul.002G017300	no data	-0.67	1.29E-01	-0.90	1.83E-02	-0.87	4.39E-02	-0.50	2.18E-01
Phvul.006G115900	PTHR24073:SF345 - RAS-RELATED PROTEIN RABA3 (1 of 2)	-0.67	3.36E-01	-1.81	1.77E-04	-0.59	4.45E-01	-1.13	2.52E-02
Phvul.011G053800	PF16021 - Programmed cell death protein 7 (PDCD7) (1 of 1)	-0.67	1.33E-02	-0.55	3.16E-02	-0.37	3.11E-01	-0.47	8.12E-02
Phvul.004G063900	PF03007//PF06974 - Wax ester synthase-like Acyl-CoA acyltransferase domain (WES acyltransf) // Protein of unknown f	-0.67	3.43E-01	-1.88	9.02E-05	-0.86	2.25E-01	-1.48	3.36E-03
Phvul.001G143600	PTHR32285:SF22 - PROTEIN TRICHOME BIREFRINGENCE-RELATED (1 of 2)	-0.67	9.44E-02	-0.84	9.13E-03	-0.58	2.04E-01	-0.66	5.50E-02
Phvul.008G179000	PTHR24006:SF430 - F-BOX PROTEIN FBX14-RELATED (1 of 1)	-0.67	4.32E-02	-0.41	1.80E-01	-0.45	2.81E-01	-0.46	1.25E-01
Phvul.009G058500	PF00249 - Myb-like DNA-binding domain (Myb DNA-binding) (1 of 287)	-0.67	5.47E-01	-2.29	1.12E-03	-0.59	6.48E-01	-1.39	6.08E-02
Phvul.007G094300	PTHR32241:SF3 - PATATIN-LIKE PROTEIN 6-RELATED (1 of 1)	-0.67	7.67E-02	-0.63	4.41E-02	-0.46	3.29E-01	-0.59	7.21E-02
Phvul.008G170900	no data	-0.67	2.89E-01	-1.72	2.91E-04	-0.51	5.00E-01	-1.22	1.29E-02
Phvul.009G074400	PTHR32044:SF7 - XYLOGLUCAN GLYCOSYLTRANSFERASE 12-RELATED (1 of 1)	-0.67	3.26E-01	-1.17	1.38E-02	-0.55	4.92E-01	-0.99	4.88E-02
Phvul.008G130900	K10782 - fatty acyl-ACP thioesterase A (FATA) (1 of 1)	-0.67	2.75E-01	-1.17	1.17E-02	-0.41	5.96E-01	-0.88	7.16E-02
Phvul.008G202400	2.7.1.59 - N-acetylglucosamine kinase / GlcNAc kinase (1 of 1)	-0.67	7.78E-01	-2.96	4.84E-02	-0.93	7.19E-01	-3.41	2.67E-02
Phvul.001G164400	PTHR32080:SF9 - CYSTEINE-RICH REPEAT SECRETORY PROTEIN 56 (1 of 2)	-0.67	4.45E-01	-1.06	1.46E-01	-0.66	4.96E-01	-1.52	4.18E-02
Phvul.009G028900	PTHR31614:SF5 - ALLERGEN-LIKE PROTEIN BRSN20-RELATED (1 of 2)	-0.67	3.16E-01	-1.66	2.91E-04	-0.46	5.79E-01	-1.15	1.84E-02
Phvul.002G072900	PTHR13902//PTHR13902:SF23 - SERINE/THREONINE-PROTEIN KINASE WNK WITH NO LYSINE -RELATED // SUBFAMILY N	-0.68	1.14E-01	-0.64	7.93E-02	-0.54	2.75E-01	-0.82	2.39E-02
Phvul.007G204900	PTHR11601//PTHR11601:SF47 - CYSTEINE DESULFURYLASE // SUBFAMILY NOT NAMED (1 of 3)	-0.68	1.64E-02	-0.09	7.96E-01	-0.30	4.54E-01	0.07	8.56E-01
Phvul.003G260300	PTHR27000:SF26 - F10A5.16 (1 of 3)	-0.68	1.47E-01	-1.04	4.31E-03	-0.54	3.24E-01	-0.86	2.48E-02
Phvul.005G008700	PTHR15907:SF35 - GENE1000 PROTEIN-RELATED (1 of 2)	-0.68	2.34E-01	-0.73	1.15E-01	-0.53	4.29E-01	-1.01	3.02E-02
Phvul.005G064600	PTHR28039:SF6 - ALTERED INHERITANCE OF MITOCHONDRIA PROTEIN 18, MITOCHONDRIAL-RELATED (1 of 2)	-0.68	1.78E-01	-1.42	2.33E-04	-0.73	1.70E-01	-1.31	9.57E-04
Phvul.L002259	PF14577 - Sieve element occlusion C-terminus (SEO C) (1 of 14)	-0.68	7.57E-02	-0.70	2.75E-02	-0.43	3.68E-01	-0.51	1.34E-01
Phvul.006G189600	no data	-0.68	1.36E-01	-1.08	3.76E-03	-0.85	5.98E-02	-0.70	7.55E-02
Phvul.008G235700	PTHR31448:SF3 - IFA-BINDING PROTEIN (1 of 3)	-0.68	1.11E-01	-0.76	3.25E-02	-0.65	1.71E-01	-0.70	5.80E-02
Phvul.001G120400	2.3.3.13 - 2-isopropylmalate synthase / Isopropylmalate synthetase (1 of 7)	-0.68	1.10E-01	-0.90	6.45E-03	-0.70	1.23E-01	-0.59	1.02E-01
Phvul.004G164600	6.3.4.19 - tRNA(Ile)-lysidine synthetase / Isoleucine-specific transfer ribonucleate lysidine synthetase (1 of 1)	-0.68	2.75E-01	-1.26	5.19E-03	-0.46	5.50E-01	-1.68	2.52E-04
Phvul.007G210100	no data	-0.68	7.50E-01	-2.32	5.42E-02	-1.61	4.25E-01	-2.85	2.14E-02
Phvul.008G017400	PF00069//PF00560//PF08263//PF13855 - Protein kinase domain (Pkinase) // Leucine Rich Repeat (LRR 1) // Leucine ric	-0.68	3.88E-01	-1.82	5.90E-04	-0.95	2.24E-01	-1.36	1.45E-02
Phvul.003G020900	no data	-0.68	3.84E-01	-1.36	2.80E-02	-0.63	4.65E-01	-0.23	7.47E-01
Phvul.003G288300	PTHR27001:SF105 - PROTEIN KINASE FAMILY PROTEIN-RELATED (1 of 2)	-0.68	3.49E-01	-1.14	1.20E-01	-1.99	3.64E-03	-1.24	9.08E-02
Phvul.001G212100	PTHR30620:SF4 - BETA-D-XYLOSIDASE 2-RELATED (1 of 2)	-0.68	2.03E-01	-1.29	1.07E-03	-0.47	4.78E-01	-1.09	8.23E-03
Phvul.011G039400	K11341 - YEATS domain-containing protein 4 (YEATS4, GAS41, YAF9) (1 of 2)	-0.69	4.81E-01	-1.88	7.78E-03	-0.71	4.91E-01	-1.29	7.37E-02
Phvul.L001744	PTHR32080:SF4 - CYSTEINE-RICH REPEAT SECRETORY PROTEIN 11-RELATED (1 of 2)	-0.69	2.36E-01	-0.99	4.11E-02	-0.44	5.43E-01	-0.58	2.61E-01
Phvul.005G136900	PTHR31384:SF20 - AUXIN RESPONSE FACTOR 4 (1 of 2)	-0.69	1.04E-01	-0.97	3.61E-03	-0.41	4.52E-01	-0.76	3.23E-02
Phvul.L002459	PF14577 - Sieve element occlusion C-terminus (SEO C) (1 of 14)	-0.69	1.30E-01	-1.00	5.58E-03	-0.56	2.92E-01	-0.51	1.99E-01
Phvul.002G009500	PTHR31096:SF7 - ACR1 (1 of 2)	-0.69	2.89E-01	-1.29	4.75E-03	-0.21	8.37E-01	-0.88	7.40E-02
Phvul.011G102500	PTHR11709//PTHR11709:SF106 - MULTI-COPPER OXIDASE // SUBFAMILY NOT NAMED (1 of 1)	-0.69	3.49E-01	-1.59	1.40E-03	-0.65	4.25E-01	-1.09	4.08E-02
Phvul.004G013900	PTHR32212:SF126 - EMB (1 of 24)	-0.69	3.46E-01	-1.88	1.67E-04	-0.63	4.32E-01	-1.48	4.65E-03
Phvul.003G197900	PTHR24072:SF149 - RAC-LIKE GTP-BINDING PROTEIN ARAC10-RELATED (1 of 2)	-0.69	5.50E-01	-2.28	5.40E-03	-0.26	8.71E-01	-1.29	1.21E-01
Phvul.001G043500	PTHR27007:SF24 - L-TYPE LECTIN-DOMAIN CONTAINING RECEPTOR KINASE IX.1-RELATED (1 of 16)	-0.69	6.81E-01	-2.27	4.35E-02	-0.57	7.75E-01	-1.10	3.45E-01
Phvul.002G095900	PTHR34545:SF2 - CLAVATA3/ESR (CLE)-RELATED PROTEIN 16-RELATED (1 of 1)	-0.69	6.29E-01	-2.40	4.25E-03	-0.68	6.62E-01	-2.68	2.60E-03
Phvul.001G181300	PTHR24073:SF536 - RAS-RELATED PROTEIN RABC2A (1 of 3)	-0.69	1.73E-01	-0.91	2.33E-02	-0.51	4.04E-01	-0.48	2.78E-01
Phvul.009G108700	PTHR10374//PTHR10374:SF21 - LACTOYLGLUTATHIONE LYASE GLYOXALASE I // SUBFAMILY NOT NAMED (1 of 1)	-0.69	3.96E-02	-1.02	2.72E-04	-0.68	5.87E-02	-0.68	2.24E-02
Phvul.011G141500	PTHR24115:SF520 - KINESIN-2-RELATED (1 of 2)	-0.69	1.12E-01	-0.93	1.23E-02	-0.43	4.31E-01	-0.48	2.34E-01
Phvul.008G111300	no data	-0.69	2.51E-02	-0.64	3.20E-02	-0.32	4.36E-01	-0.65	3.19E-02
Phvul.003G096500	no data	-0.69	6.36E-02	-0.79	1.00E-02	-0.60	1.53E-01	-0.78	1.50E-02
Phvul.004G086300	K13103 - tuftelin-interacting protein 11 (TFIP11) (1 of 1)	-0.69	1.46E-01	-1.22	1.95E-03	-0.61	2.65E-01	-1.08	7.64E-03
Phvul.005G099100	PTHR24072:SF167 - RAC-LIKE GTP-BINDING PROTEIN ARAC2 (1 of 2)	-0.69	2.28E-01	-1.21	1.32E-02	-0.50	4.57E-01	-0.91	6.95E-02
Phvul.002G279700	no data	-0.69	1.18E-01	-0.91	8.92E-03	-0.69	1.57E-01	-0.80	2.97E-02
Phvul.009G030900	PTHR14791 - BOMB/KIRA PROTEINS (1 of 3)	-0.70	2.89E-01	-0.94	5.17E-02	-0.53	4.93E-01	-1.00	4.50E-02
Phvul.001G193700	no data	-0.70	1.63E-01	-0.91	1.74E-02	-0.59	3.11E-01	-0.81	4.40E-02
Phvul.011G150200	PTHR27002:SF9 - CYSTEINE-RICH RECEPTOR-LIKE PROTEIN KINASE 27-RELATED (1 of 4)	-0.70	6.54E-01	2.06	1.37E-02	0.24	9.10E-01	0.95	3.61E-01
Phvul.003G188901	PTHR31889:SF2 - FUCOSYLTRANSFERASE 2-RELATED (1 of 2)	-0.70	2.36E-01	-1.80	4.95E-05	-0.81	1.94E-01	-1.07	2.15E-02
Phvul.001G140200	PTHR10024:SF243 - ANTHRANILATE PHOSPHORIBOSYLTRANSFERASE-LIKE PROTEIN (1 of 3)	-0.70	1.72E-01	-1.04	7.73E-03	-0.67	2.42E-01	-0.81	5.00E-02
Phvul.008G026300	PF01190 - Pollen proteins Ole e I like (Pollen Ole e I) (1 of 29)	-0.70	2.99E-01	-1.53	1.96E-03	-0.54	5.04E-01	-0.90	9.04E-02
Phvul.006G063401	no data	-0.70	2.53E-01	-1.33	3.23E-03	-0.76	2.54E-01	-1.51	1.29E-03
Phvul.004G005400	PTHR24296:SF1 - ALKANE HYDROXYLASE CYP96A15-RELATED (1 of 11)	-0.70	1.15E-01	-0.78	2.95E-02	-0.53	3.20E-01	-0.57	1.39E-01
Phvul.003G088700	PTHR22814//PTHR22814:SF94 - COPPER TRANSPORT PROTEIN ATOX1-RELATED // SUBFAMILY NOT NAMED (1 of 2)	-0.70	2.94E-01	-1.44	1.08E-02	-1.16	7.89E-02	-0.46	4.29E-01
Phvul.011G047600	PTHR24073:SF375 - RAS-RELATED PROTEIN RABG3F (1 of 1)	-0.71	4.27E-01	-1.58	8.80E-03	-0.80	3.82E-01	-1.48	1.74E-02
Phvul.002G210300	K14396 - polyadenylate-binding protein 2 (PABPN1, PABP2) (1 of 4)	-0.71	3.16E-01	-1.09	3.59E-02	-0.52	5.30E-01	-1.26	1.84E-02
Phvul.009G012700	no data	-0.71	3.16E-01	-1.37	6.81E-03	-0.77	3.06E-01	-0.96	7.20E-02
Phvul.006G056000	no data	-0.71	4.48E-01	0.98	1.60E-01	-0.42	7.15E-01	1.34	3.63E-02
Phvul.009G200200	PTHR31727:SF2 - PALMITOYL-ACYL CARRIER PROTEIN THIOESTERASE, CHLOROPLASTIC (1 of 2)	-0.71	4.15E-02	-0.75	1.04E-02	-0.57	1.61E-01	-0.48	1.31E-01
Phvul.001G194200	K03386 - peroxiredoxin (alkyl hydroperoxide reductase subunit C) [EC:1.11.1.15] (E1.11.1.15, PRDX, ahpC) (1 of 2)	-0.71	4.87E-02	-1.14	8.42E-05	-0.57	1.86E-01	-0.92	2.72E-03
Phvul.007G206400	PTHR11654//PTHR11654:SF110 - OLIGOPEPTIDE TRANSPORTER-RELATED // SUBFAMILY NOT NAMED (1 of 2)	-0.71	7.81E-02	-0.71	3.61E-02	-0.63	1.64E-01	-0.55	1.26E-01
Phvul.001G215700	PTHR11453:SF44 - BORON TRANSPORTER 1-RELATED (1 of 2)	-0.71	1.08E-01	-0.77	2.93E-02	-0.42	4.65E-01	-0.49	2.03E-01
Phvul.008G207200	PTHR24177:SF42 - ANKYRIN REPEAT FAMILY PROTEIN (1 of 4)	-0.71	2.23E-01	-0.56	2.67E-01	-0.79	2.07E-01	-0.96	4.87E-02
Phvul.004G174000	PTHR31079:SF3 - NAC DOMAIN CONTAINING PROTEIN 75-RELATED (1 of 2)	-0.71	2.53E-02	0.02	9.74E-01	-0.27	5.59E-01	0.28	3.89E-01
Phvul.008G207800	PTHR10641:SF475 - MYB TRANSCRIPTION FACTOR-RELATED (1 of 3)	-0.71	5.45E-01	-2.03	8.58E-03	-0.50	7.31E-01	-1.59	4.42E-02
Phvul.002G258300	PTHR31263:SF0 - CELLULASE (GLYCOSYL HYDROLASE FAMILY 5) PROTEIN-RELATED (1 of 3)	-0.72	1.61E-01	-0.88	3.52E-02	-0.37	5.80E-01	-0.40	3.95E-01
Phvul.011G058200	PTHR10024:SF243 - ANTHRANILATE PHOSPHORIBOSYLTRANSFERASE-LIKE PROTEIN (1 of 3)	-0.72	5.02E-01	-1.94	4.93E-03	-1.10	2.90E-01	-1.77	1.67E-02
Phvul.002G129100	2.1.1.41 - Sterol 24-C-methyltransferase / Zymosterol-24-methyltransferase (1 of 13)	-0.72	2.03E-01	-1.45	3.53E-04	-0.59	3.63E-01	-1.08	1.20E-02
Phvul.001G239200	PF00139 - Legume lectin domain (Lectin legB) (1 of 53)	-0.72	3.05E-01	-1.28	1.49E-02	-0.38	6.76E-01	-0.37	5.51E-01
Phvul.009G052200	PTHR32295:SF33 - PROTEIN IQ-DOMAIN 21 (1 of 2)	-0.72	1.57E-01	-1.22	1.51E-03	-0.64	2.70E-01	-0.88	3.28E-02
Phvul.002G251900	PTHR31747:SF3 - PROTEIN LSD1 (1 of 1)	-0.72	4.23E-01	1.10	4.00E-02	-0.08	9.61E-01	-0.01	9.94E-01
Phvul.007G093800	PTHR13377 - PLACENTAL PROTEIN 6 (1 of 2)	-0.72	1.72E-01	-1.22	3.17E-03	-0.70	2.28E-01	-1.31	2.22E-03
Phvul.007G257800	PTHR10024:SF243 - ANTHRANILATE PHOSPHORIBOSYLTRANSFERASE-LIKE PROTEIN (1 of 3)	-0.72	2.78E-01	-1.35	4.85E-03	-0.65	3.79E-01	-1.13	2.55E-02
Phvul.001G146800	PTHR27001:SF54 - RECEPTOR-LIKE CYTOPLASMIC KINASE VIA5 (1 of 2)	-0.72	6.86E-01	-1.98	7.37E-02	-1.18	5.02E-01	-2.41	3.68E-02
Phvul.006G000066	PF02298 - Plastocyanin-like domain (Cu bind like) (1 of 53)	-0.72	3.45E-01	-1.34	1.32E-02	-0.69	4.08E-01	-0.95	9.68E-02
Phvul.008G166500	PTHR11728:SF8 - GLYCEROL-3-PHOSPHATE DEHYDROGENASE [NAD(+)], CYTOPLASMIC Pathogenesis-related	-0.72	1.21E-01	-1.06	1.65E-02	-0.52	3.45E-01	-0.27	5.96E-01
Phvul.003G237300	PTHR10795//PTHR10795:SF388 - PROPROTEIN CONVERTASE SUBTILISIN/KEXIN // SUBFAMILY NOT NAMED (1 of 5)	-0.72	1.06E-01	-0.83	1.89E-02	-0.52	3.44E-01	-0.79	3.25E-02
Phvul.002G263900	PF01535//PF13041 - PPR repeat (PPR) // PPR repeat family (PPR 2) (1 of 197)	-0.72	3.00E-01	-1.09	2.87E-02	-0.49	5.63E-01	-1.36	7.71E-03
Phvul.008G288800	PTHR32382:SF4 - FASCICLIN-LIKE ARABINOGALACTAN PROTEIN 1 (1 of 2)	-0.72	3.31E-01	-1.18	2.36E-02	-0.42	6.67E-01	-1.15	3.46E-02
Phvul.001G256900	PTHR31683:SF26 - PECTATE LYASE 5-RELATED (1 of 2)	-0.72	5.93E-01	-2.67	1.47E-03	-0.78	5.78E-01	-1.68	5.54E-02
Phvul.002G292800	PTHR22814//PTHR22814:SF127 - COPPER TRANSPORT PROTEIN ATOX1-RELATED // SUBFAMILY NOT NAMED (1 of 1)	-0.73	3.96E-02	-0.88	2.44E-03	-0.69	7.29E-02	-0.79	9.69E-03
Phvul.007G217100	PTHR13778:SF8 - GALACTURONOSYLTRANSFERASE-LIKE 6-RELATED (1 of 2)	-0.73	2.97E-01	-1.01	4.95E-02	-0.25	8.16E-01	-0.61	2.71E-01
Phvul.009G219300	PTHR27001:SF69 - PROTEIN KINASE FAMILY PROTEIN (1 of 2)	-0.73	3.53E-01	-1.06	5.97E-02	-0.70	4.16E-01	-1.21	3.47E-02
Phvul.009G083500	PF07911 - Protein of unknown function (DUF1677) (DUF1677) (1 of 10)	-0.73	3.24E-01	-1.54	2.75E-03	-0.22	8.42E-01	-1.25	2.15E-02
Phvul.002G051100	K02969 - small subunit ribosomal protein S20e (RP-S20e, RPS20) (1 of 3)	-0.73	5.65E-02	-0.73	2.08E-02	-0.61	1.67E-01	-1.08	7.55E-04
Phvul.001G137700	PTHR31344:SF4 - NUCLEOLAR PROTEIN GAR2-LIKE PROTEIN (1 of 1)	-0.73	4.69E-02	-0.67	3.33E-02	-0.41	4.05E-01	-0.48	1.51E-01
Phvul.011G059900	PF03100 - CcmE (CcmE) (1 of 1)	-0.73	2.11E-01	-1.22	4.51E-03	-0.47	5.18E-01	-1.23	5.78E-03
Phvul.006G084300	PF11955 - Plant organelle RNA recognition domain (PORR) (1 of 16)	-0.73	4.88E-01	-1.59	2.53E-02	-0.74	5.10E-01	-1.15	1.10E-01
Phvul.008G217700	no data	-0.73	2.57E-02	-0.28	4.37E-01	-0.47	2.62E-01	-0.18	6.44E-01
Phvul.001G063600	PTHR13856:SF78 - ENTH/VHS/GAT FAMILY PROTEIN (1 of 2)	-0.73	1.05E-01	-0.68	6.92E-02	-0.82	7.93E-02	-0.82	3.26E-02
Phvul.001G067700	PTHR34194:SF3 - F14J8.16 PROTEIN-RELATED (1 of 1)	-0.73	4.45E-02	-0.98	1.18E-03	-0.62	1.38E-01	-0.76	1.87E-02

Phvul.004G032800	PTHR32212:SF126 - EMB (1 of 24)	-0.73	1.21E-01	-1.03	5.00E-03	-0.61	2.70E-01	-0.98	1.05E-02
Phvul.002G018200	PTHR33136:SF7 - PROTEIN RALF-LIKE 26-RELATED (1 of 1)	-0.74	3.15E-01	-1.54	2.51E-03	-0.51	5.64E-01	-1.01	6.32E-02
Phvul.003G054801	K03248 - translation initiation factor 3 subunit G (EIF3G) (1 of 2)	-0.74	1.76E-01	-1.13	5.11E-03	-0.75	2.07E-01	-0.99	2.02E-02
Phvul.009G075801	no data	-0.74	8.52E-02	-0.99	3.49E-03	-0.55	2.86E-01	-0.89	1.22E-02
Phvul.001G194500	PTHR13683:SF340 - ASPARTYL PROTEASE FAMILY PROTEIN (1 of 1)	-0.74	4.78E-01	-2.14	2.01E-03	-0.32	8.33E-01	-1.38	5.83E-02
Phvul.005G123300	PTHR23177:SF20 - RHO GTPASE-ACTIVATING PROTEIN 1-RELATED (1 of 2)	-0.74	2.87E-02	-1.01	4.00E-04	-0.41	3.74E-01	-0.65	3.39E-02
Phvul.003G138100	PTHR32285:SF8 - PROTEIN TRICHOME BIREFRINGENCE-LIKE 5 (1 of 1)	-0.74	1.44E-01	-1.13	1.14E-02	-0.35	6.11E-01	-0.98	3.35E-02
Phvul.007G147900	PTHR36738:SF1 - EXPRESSED PROTEIN (1 of 1)	-0.74	1.69E-01	-1.30	2.02E-03	-0.49	4.71E-01	-1.06	1.59E-02
Phvul.006G068500	K07300 - Ca2+:H+ antiporter (chaA, CAX) (1 of 6)	-0.74	6.86E-02	-0.73	3.74E-02	-0.56	2.52E-01	-0.87	1.45E-02
Phvul.003G132600	PTHR14155//PTHR14155:SF93 - RING FINGER DOMAIN-CONTAINING // SUBFAMILY NOT NAMED (1 of 2)	-0.74	1.27E-01	-0.85	4.11E-02	-0.79	1.27E-01	-0.62	1.49E-01
Phvul.009G124400	PTHR23354:SF75 - LYSM DOMAIN-CONTAINING GPI-ANCHORED PROTEIN 1 (1 of 2)	-0.74	2.27E-01	-1.11	2.12E-02	-0.57	4.29E-01	-1.09	2.99E-02
Phvul.003G068300	K11662 - actin-related protein 6 (ACTR6, ARP6) (1 of 1)	-0.75	1.18E-01	-0.87	2.57E-02	-0.57	3.15E-01	-0.58	1.64E-01
Phvul.003G279300	PTHR10795//PTHR10795:SF382 - PROPROTEIN CONVERTASE SUBTILISIN/KEXIN // SUBFAMILY NOT NAMED (1 of 1)	-0.75	2.56E-01	-1.44	2.34E-03	-0.52	5.16E-01	-1.15	2.16E-02
Phvul.003G142700	PTHR31734:SF11 - AUXIN-RESPONSIVE PROTEIN IAA1-RELATED (1 of 2)	-0.75	5.92E-02	-0.60	8.17E-02	-0.81	4.66E-02	-0.47	1.94E-01
Phvul.007G052800	PTHR31062:SF1 - XYLOGLUCAN ENDOTRANSGLUCOSYLASE/HYDROLASE PROTEIN 8-RELATED (1 of 2)	-0.75	3.51E-01	-1.48	5.93E-03	-0.79	3.56E-01	-1.36	1.65E-02
Phvul.009G171300	PF14577 - Sieve element occlusion C-terminus (SEO C) (1 of 14)	-0.75	3.08E-02	-0.55	8.17E-02	-0.38	4.21E-01	-0.32	3.61E-01
Phvul.006G153400	PTHR22814:SF33 - HEAVY METAL TRANSPORT/DETOXIFICATION DOMAIN-CONTAINING PROTEIN (1 of 2)	-0.75	1.21E-01	-1.42	1.63E-04	-0.84	9.42E-02	-1.19	2.33E-03
Phvul.005G043100	PF06136 - Domain of unknown function (DUF966) (DUF966) (1 of 6)	-0.75	1.18E-01	-1.08	4.74E-03	-0.44	4.80E-01	-0.83	4.09E-02
Phvul.003G152900	PF00069//PF08263//PF13855 - Protein kinase domain (Pkinase) // Leucine rich repeat N-terminal domain (LRRNT 2) //	-0.76	1.00E-02	-0.15	6.55E-01	-0.33	4.33E-01	0.06	8.72E-01
Phvul.011G110300	no data	-0.76	3.55E-01	-1.26	2.81E-02	-0.74	4.17E-01	-1.10	6.57E-02
Phvul.009G082100	Pathogenesis-related protein p14a	-0.76	2.41E-01	-1.73	1.89E-04	-0.65	3.79E-01	-1.44	2.73E-03
Phvul.003G238200	PTHR37209:SF2 - LYSINE-RICH ARABINOGALACTAN PROTEIN 17-RELATED (1 of 2)	-0.76	4.22E-01	-1.78	4.05E-03	-0.40	7.55E-01	-1.37	3.60E-02
Phvul.009G190698	PTHR23023:SF86 - FLAVIN-CONTAINING MONOOXYGENASE FMO GS-OX-LIKE 8-RELATED (1 of 4)	-0.76	1.00E-01	-1.09	1.38E-02	-0.73	1.53E-01	-0.31	5.39E-01
Phvul.009G021000	PTHR11709:SF90 - MULTI-COPPER OXIDASE TYPE I FAMILY PROTEIN-RELATED (1 of 2)	-0.76	6.26E-01	-2.30	1.44E-02	-0.52	7.90E-01	-2.03	3.88E-02
Phvul.001G185400	PF00069//PF08263 - Protein kinase domain (Pkinase) // Leucine rich repeat N-terminal domain (LRRNT 2) (1 of 19)	-0.76	2.13E-01	-1.86	2.27E-04	-0.85	1.86E-01	-1.36	7.39E-03
Phvul.007G196700	PTHR23213:SF177 - FORMIN-LIKE PROTEIN 11 (1 of 3)	-0.76	6.08E-01	-2.47	7.56E-03	-0.88	5.67E-01	-1.91	4.85E-02
Phvul.001G218400	PF12708 - Pectate lyase superfamily protein (Pectate lyase 3) (1 of 32)	-0.76	1.80E-01	-1.08	1.48E-02	-0.86	1.49E-01	-0.98	3.45E-02
Phvul.007G026900	3.2.1.177 - Alpha-D-xyloside xylohydrolase / Alpha-xylosidase (1 of 2)	-0.76	2.69E-01	-1.69	4.96E-03	-0.78	2.97E-01	-0.94	1.23E-01
Phvul.003G088400	no data	-0.76	3.76E-02	-0.81	1.54E-02	-0.69	9.14E-02	-0.44	2.24E-01
Phvul.L001667	PTHR12801:SF56 - EXONUCLEASE FAMILY PROTEIN (1 of 1)	-0.76	5.74E-01	-2.17	1.36E-02	-0.04	9.86E-01	-1.26	1.73E-01
Phvul.001G095100	no data	-0.76	2.49E-01	-1.19	5.09E-02	-0.39	6.45E-01	-1.21	4.99E-02
Phvul.010G010200	PTHR31867:SF9 - EXPANSIN-A6 (1 of 6)	-0.76	4.26E-01	-3.54	2.41E-04	-0.63	5.49E-01	-2.15	1.27E-02
Phvul.004G149100	PTHR27008:SF19 - INACTIVE RECEPTOR KINASE RLK902-RELATED (1 of 2)	-0.77	9.37E-02	-1.20	1.15E-03	-0.57	3.03E-01	-0.99	1.04E-02
Phvul.001G131700	PTHR11977:SF46 - VILLIN-2-RELATED (1 of 2)	-0.77	1.14E-02	-0.83	1.98E-03	-0.61	8.23E-02	-0.51	8.06E-02
Phvul.004G002400	K02864 - large subunit ribosomal protein L10 (RP-L10, MRPL10, rplJ) (1 of 1)	-0.77	7.34E-02	-0.89	1.04E-02	-0.49	3.59E-01	-0.52	1.74E-01
Phvul.008G184600	PTHR31066:SF2 - GB (1 of 3)	-0.77	2.57E-01	-1.12	3.07E-02	-0.49	5.61E-01	-1.06	4.99E-02
Phvul.004G035200	no data	-0.77	5.21E-01	-2.08	7.46E-03	-1.08	3.55E-01	-2.30	4.59E-03
Phvul.001G202400	PF07765 - KIP1-like protein (KIP1) (1 of 16)	-0.77	9.60E-02	-0.31	5.24E-01	-0.95	4.26E-02	-0.15	7.78E-01
Phvul.007G040400	PTHR27008:SF42 - LEUCINE-RICH REPEAT PROTEIN KINASE-LIKE PROTEIN (1 of 1)	-0.77	2.94E-01	-1.66	1.60E-03	-0.76	3.39E-01	-1.29	1.98E-02
Phvul.010G036900	PTHR11926:SF360 - UDP-GLYCOSYLTRANSFERASE 91A1-RELATED (1 of 2)	-0.77	2.16E-01	-1.44	2.54E-03	-0.24	8.08E-01	-0.74	1.52E-01
Phvul.002G273700	K08065 - nuclear transcription Y subunit beta (NFYB) (1 of 3)	-0.77	1.61E-01	-1.17	5.36E-03	-0.74	2.30E-01	-0.88	4.90E-02
Phvul.007G053100	PTHR23177 - MKIAA1688 PROTEIN (1 of 13)	-0.77	2.69E-01	-1.06	4.00E-02	-0.43	6.40E-01	-1.12	3.52E-02
Phvul.003G197000	K03501 - 16S rRNA (guanine527-N7)-methyltransferase [EC:2.1.1.170] (gidB, rsmG) (1 of 1)	-0.77	4.02E-01	-0.49	5.57E-01	-1.68	4.74E-02	-0.29	7.42E-01
Phvul.001G172000	PTHR21229//PTHR21229:SF26 - LUNG SEVEN TRANSMEMBRANE RECEPTOR // SUBFAMILY NOT NAMED (1 of 2)	-0.77	1.65E-01	-1.36	1.46E-03	-0.62	3.38E-01	-1.13	1.19E-02
Phvul.007G094600	PTHR19139:SF167 - AQUAPORIN PIP2-1-RELATED (1 of 5)	-0.77	7.72E-02	-0.88	1.22E-02	-0.62	2.34E-01	-0.91	1.27E-02
Phvul.006G087300	PTHR22870:SF177 - F12P19.9 PROTEIN (1 of 2)	-0.77	1.39E-01	-1.19	6.37E-03	-0.59	3.47E-01	-0.87	5.54E-02
Phvul.001G237100	PTHR24209:SF7 - PROTEIN DA1-RELATED 2 (1 of 2)	-0.78	2.86E-03	-0.58	2.51E-02	-0.71	1.17E-02	-0.38	1.71E-01
Phvul.009G077000	PTHR11034:SF23 - N-MYC DOWNREGULATED-LIKE 3 PROTEIN (1 of 4)	-0.78	2.88E-01	-1.42	5.77E-03	-0.58	5.00E-01	-1.27	1.89E-02
Phvul.008G006300	no data	-0.78	1.93E-01	-0.96	2.94E-02	-0.82	2.05E-01	-0.72	1.18E-01
Phvul.011G061400	PTHR10283:SF76 - DICARBOXYLATE TRANSPORTER 1, CHLOROPLASTIC (1 of 1)	-0.78	2.72E-02	-0.91	6.96E-03	-0.61	1.42E-01	-1.04	2.73E-03
Phvul.003G090500	PTHR12277:SF58 - ALPHA/BETA-HYDROLASES SUPERFAMILY PROTEIN (1 of 3)	-0.78	1.17E-02	-0.86	1.31E-03	-0.54	1.60E-01	-0.61	3.49E-02
Phvul.009G153100	PTHR23421:SF67 - BETA-GALACTOSIDASE 10 (1 of 1)	-0.78	2.53E-01	-1.39	4.30E-03	-0.59	4.70E-01	-1.08	3.70E-02
Phvul.007G241300	PTHR22884:SF378 - HISTONE-LYSINE N-METHYLTRANSFERASE MES-4-RELATED (1 of 3)	-0.78	1.42E-01	0.25	6.48E-01	-1.03	4.87E-02	-0.01	9.89E-01
Phvul.001G045900	PTHR33184:SF11 - BETA-1,3-N-ACETYLGLUCOSAMINYLTRANSFERASE FAMILY PROTEIN (1 of 9)	-0.78	3.21E-01	-1.66	3.12E-03	-0.40	7.11E-01	-1.29	2.88E-02
Phvul.006G160900	PTHR10809:SF59 - VESICLE-ASSOCIATED PROTEIN 2-2 (1 of 3)	-0.78	2.27E-01	-1.22	1.21E-02	-0.51	5.20E-01	-0.92	7.64E-02
Phvul.002G084400	PTHR11214:SF129 - BETA-1,3-GALACTOSYLTRANSFERASE 15 (1 of 1)	-0.78	1.36E-02	-0.57	6.38E-02	-0.35	4.29E-01	-0.64	4.07E-02
Phvul.001G151400	PTHR32241:SF5 - INACTIVE PATATIN-LIKE PROTEIN 9-RELATED (1 of 2)	-0.78	3.92E-01	-1.93	2.83E-03	-0.24	8.59E-01	-1.44	3.38E-02
Phvul.007G231300	no data	-0.78	2.83E-01	-2.34	9.35E-06	-0.89	2.50E-01	-1.96	2.19E-04
Phvul.001G008000	PTHR31580:SF4 - FILAMENT-LIKE PLANT PROTEIN 4-RELATED (1 of 3)	-0.78	1.72E-01	-1.34	1.79E-03	-0.70	2.80E-01	-0.89	5.45E-02
Phvul.006G160800	PTHR20863:SF29 - ACYL CARRIER PROTEIN 1, CHLOROPLASTIC-RELATED (1 of 3)	-0.78	6.55E-02	-1.54	8.57E-06	-0.55	2.90E-01	-1.23	5.01E-04
Phvul.001G210500	K10406 - kinesin family member C2/C3 (KIFC2 3) (1 of 13)	-0.78	4.87E-02	-0.67	5.72E-02	-0.67	1.42E-01	-0.33	3.96E-01
Phvul.010G159700	KOG0324 - Uncharacterized conserved protein (1 of 12)	-0.79	8.49E-02	-1.13	2.36E-03	-0.35	5.68E-01	-0.79	4.07E-02
Phvul.010G116500	PTHR24073:SF345 - RAS-RELATED PROTEIN RABA3 (1 of 2)	-0.79	3.54E-01	-1.64	9.01E-03	-0.92	3.02E-01	-1.63	1.24E-02
Phvul.010G152800	PTHR32370:SF16 - NON-PHOTOTROPIC HYPOCOTYL-LIKE PROTEIN (1 of 2)	-0.79	2.88E-01	-1.62	2.04E-03	-0.39	7.05E-01	-1.32	1.76E-02
Phvul.010G124400	PTHR10795:SF424 - SUBTILASE FAMILY PROTEIN-RELATED (1 of 2)	-0.79	1.10E-01	-0.81	4.75E-02	-0.61	3.03E-01	-0.81	5.47E-02
Phvul.011G014100	PTHR32295:SF35 - PROTEIN IQ-DOMAIN 11 (1 of 2)	-0.79	2.34E-01	-1.16	2.20E-02	-0.62	4.23E-01	-0.88	9.98E-02
Phvul.010G054000	no data	-0.79	1.33E-01	-0.94	4.50E-02	0.20	7.81E-01	-0.22	6.75E-01
Phvul.L004037	PF14577 - Sieve element occlusion C-terminus (SEO C) (1 of 14)	-0.79	4.39E-02	-0.35	3.87E-01	-0.62	1.87E-01	-0.09	8.57E-01
Phvul.003G067800	PTHR12565:SF148 - PROTEIN BANQUO 3 (1 of 3)	-0.79	4.00E-02	-0.96	6.99E-03	-0.86	3.14E-02	-0.67	7.07E-02
Phvul.001G028200	PTHR24206:SF35 - TRANSCRIPTION FACTOR-RELATED (1 of 2)	-0.79	1.65E-01	-1.17	7.10E-03	-0.64	3.40E-01	-0.87	6.11E-02
Phvul.001G055100	PTHR10209//PTHR10209:SF119 - OXIDOREDUCTASE, 2OG-FE II OXYGENASE FAMILY PROTEIN // SUBFAMILY NOT NAME	-0.80	2.69E-01	-1.43	1.04E-02	-0.39	6.95E-01	-1.22	3.38E-02
Phvul.007G064100	PTHR10795:SF322 - SUBTILISIN SERINE PROTEASE-RELATED (1 of 2)	-0.80	5.67E-01	-2.99	1.20E-03	-1.08	4.30E-01	-1.97	3.49E-02
Phvul.003G200600	PF02701 - Dof domain, zinc finger (zf-Dof) (1 of 41)	-0.80	1.18E-01	-1.30	1.98E-03	-0.36	6.07E-01	-0.72	9.93E-02
Phvul.009G025600	KOG2922 - Uncharacterized conserved protein (1 of 11)	-0.80	7.96E-02	-0.93	1.39E-02	-0.28	6.75E-01	-1.03	7.99E-03
Phvul.003G185300	PTHR32227:SF75 - GLUCAN ENDO-1,3-BETA-GLUCOSIDASE-LIKE PROTEIN 2-RELATED (1 of 2)	-0.80	7.32E-02	-1.32	1.97E-04	-0.67	2.01E-01	-1.18	1.27E-03
Phvul.005G135933	3.2.1.145//3.2.1.23 - Beta-galactosidase / Lactase (1 of 2)	-0.80	3.58E-01	-2.19	2.34E-04	-0.72	4.62E-01	-1.45	2.08E-02
Phvul.007G269100	PTHR11638//PTHR11638:SF69 - ATP-DEPENDENT CLP PROTEASE // SUBFAMILY NOT NAMED (1 of 1)	-0.80	4.76E-02	-0.66	6.75E-02	-0.52	3.04E-01	-0.46	2.21E-01
Phvul.001G085600	K14822 - rRNA-processing protein CGR1 (CGR1) (1 of 1)	-0.80	2.07E-01	-1.31	5.78E-03	-0.96	1.42E-01	-1.35	5.98E-03
Phvul.005G086300	no data	-0.81	1.61E-01	-1.60	5.13E-04	-0.65	3.41E-01	-0.69	1.60E-01
Phvul.009G067300	PTHR12411:SF344 - CYSTEINE PROTEASE COMPONENT OF PROTEASE-INHIBITOR COMPLEX-RELATED (1 of 2)	-0.81	2.16E-01	-1.35	1.75E-02	-0.22	8.29E-01	-0.73	2.16E-01
Phvul.001G028800	no data	-0.81	3.03E-01	-1.54	5.08E-03	-0.89	2.86E-01	-1.38	1.76E-02
Phvul.009G152700	PTHR13683:SF227 - ASPARTYL PROTEASE FAMILY PROTEIN (1 of 2)	-0.81	1.33E-01	-0.96	3.69E-02	-0.37	6.11E-01	-0.57	2.40E-01
Phvul.002G229200	PTHR21349:SF0 - 39S RIBOSOMAL PROTEIN L21, MITOCHONDRIAL (1 of 1)	-0.81	1.65E-02	-0.98	1.12E-03	-0.53	2.20E-01	-0.72	2.31E-02
Phvul.004G053500	PTHR10641:SF473 - MYB TRANSCRIPTION FACTOR (1 of 5)	-0.81	2.56E-01	-1.35	1.94E-02	-0.57	4.99E-01	-1.08	6.82E-02
Phvul.006G067900	no data	-0.81	8.04E-02	-0.84	4.15E-02	-0.38	5.26E-01	-0.32	4.86E-01
Phvul.007G110800	PTHR31371:SF2 - GENOMIC DNA, CHROMOSOME 3, TAC CLONE:K14B15 (1 of 2)	-0.82	1.10E-01	-0.88	3.81E-02	-0.42	5.34E-01	-0.59	1.98E-01
Phvul.005G042400	no data	-0.82	6.42E-02	-0.92	2.27E-02	-0.53	3.30E-01	-0.94	2.47E-02
Phvul.003G076600	K17263 - cullin-associated NEDD8-dissociated protein 1 (CAND1) (1 of 1)	-0.82	3.37E-01	-2.73	9.35E-06	-0.94	2.94E-01	-1.88	2.33E-03
Phvul.008G097600	PTHR10353:SF29 - BETA-GLUCOSIDASE 1-RELATED (1 of 9)	-0.82	7.38E-01	-4.37	3.86E-03	-3.19	1.12E-01	-2.72	4.40E-02
Phvul.003G147600	PTHR31062:SF32 - XYLOGLUCAN ENDOTRANSGLUCOSYLASE/HYDROLASE PROTEIN 25-RELATED (1 of 5)	-0.82	2.83E-01	-1.18	4.47E-02	-0.40	6.96E-01	-0.76	2.20E-01
Phvul.007G100800	no data	-0.82	1.18E-01	-1.56	7.95E-05	-0.86	1.22E-01	-0.85	4.83E-02
Phvul.008G261100	PTHR10336:SF105 - PHOSPHOINOSITIDE PHOSPHOLIPASE C 1-RELATED (1 of 1)	-0.82	2.94E-01	-0.82	1.97E-01	-1.11	1.73E-01	-1.65	1.09E-02
Phvul.007G139200	PF07795 - Protein of unknown function (DUF1635) (DUF1635) (1 of 6)	-0.82	3.16E-01	-1.42	4.16E-02	-0.68	4.64E-01	-1.00	1.63E-01
Phvul.008G100400	PTHR12321//PTHR12321:SF46 - CPG BINDING PROTEIN // SUBFAMILY NOT NAMED (1 of 3)	-0.82	3.60E-01	-1.43	4.50E-02	-0.46	6.91E-01	-1.39	6.17E-02
Phvul.002G291800	PF00651 - BTB/POZ domain (BTB) (1 of 51)	-0.83	2.68E-01	-2.01	1.17E-03	-0.32	7.56E-01	-0.82	1.84E-01
Phvul.003G052000	1.14.13.136 - 2-hydroxyisoflavanone synthase / Isoflavonoid synthase (1 of 4)	-0.83	4.16E-01	-1.72	1.06E-02	-0.57	6.48E-01	-1.09	1.32E-01
Phvul.009G067600	PTHR10177//PTHR10177:SF242 - CYCLINE // SUBFAMILY NOT NAMED (1 of 2)	-0.83	3.65E-01	-1.89	4.21E-03	-1.12	2.26E-01	-1.30	5.96E-02
Phvul.002G232100	PTHR11527:SF158 - 17.6 KDA CLASS I HEAT SHOCK PROTEIN 1-RELATED (1 of 11)	-0.84	9.01E-02	-1.34	7.13E-04	-0.58	3.32E-01	-0.92	2.82E-02
Phvul.008G157200	no data	-0.84	1.21E-01	-1.51	1.21E-03	-0.72	2.45E-01	-1.01	3.44E-02
Phvul.008G161400	6.2.1.3 - Long-chain-fatty-acid--CoA ligase / Lignoceroyl-CoA synthase (1 of 14)	-0.84	1.11E-01	-0.77	1.10E-01	-0.12	9.03E-01	-1.02	3.63E-02
Phvul.004G073600	6.1.1.9 - Valine--tRNA ligase / Valyl-tRNA synthetase (1 of 2)	-0.84	1.46E-01	-1.38	1.46E-02	-0.79	2.19E-01	-0.76	1.91E-01
Phvul.008G023501	no data	-0.84	3.70E-01	-2.54	1.13E-04	-1.14	2.24E-01	-2.07	2.07E-03
Phvul.006G127700	PF07712 - Stress up-regulated Nod 19 (SURNod19) (1 of 15)	-0.84	2.85E-01	-1.85	1.15E-03	-0.87	3.02E-01	-1.76	2.78E-03

Phvul.001G074300	K14304 - nuclear pore complex protein Nup85 (NUP85) (1 of 1)	-0.84	1.33E-01	-1.45	1.57E-03	-0.67	3.15E-01	-1.13	1.66E-02
Phvul.003G250400	no data	-0.84	1.57E-02	-0.66	3.71E-02	-0.45	3.29E-01	-0.40	2.40E-01
Phvul.008G019200	2.7.10.1//2.7.11.1 - Receptor protein-tyrosine kinase / Receptor protein tyrosine kinase // Non-specific serine/threonine	-0.84	1.43E-02	-0.81	1.47E-02	-0.38	4.22E-01	-0.66	5.62E-02
Phvul.009G028800	PTHR33059:SF4 - F28K19.24-RELATED (1 of 3)	-0.84	2.26E-02	-0.54	1.15E-01	-0.88	2.26E-02	-0.36	3.23E-01
Phvul.007G009800	PTHR11260:SF171 - GLUTATHIONE S-TRANSFERASE DHAR3, CHLOROPLASTIC (1 of 2)	-0.85	3.82E-02	-1.19	4.46E-04	-0.59	2.45E-01	-0.95	7.91E-03
Phvul.007G270800	3.1.3.2 - Acid phosphatase / Phosphomonoesterase (1 of 34)	-0.85	5.20E-01	-2.57	7.03E-03	-1.10	4.23E-01	-3.68	6.69E-04
Phvul.008G280300	1.3.1.80 - Red chlorophyll catabolite reductase / Red Chl catabolite reductase (1 of 2)	-0.85	1.45E-02	-0.40	2.81E-01	-0.62	1.36E-01	-0.17	6.87E-01
Phvul.006G139700	PTHR22814//PTHR22814:SF94 - COPPER TRANSPORT PROTEIN ATOX1-RELATED // SUBFAMILY NOT NAMED (1 of 2)	-0.85	3.43E-01	-1.90	1.86E-03	-0.68	5.09E-01	-1.86	3.19E-03
Phvul.002G292100	PTHR33403:SF2 - PROTEIN SPIRAL1-LIKE 5 (1 of 2)	-0.85	3.42E-01	-1.65	1.08E-02	-0.71	4.92E-01	-1.32	5.34E-02
Phvul.006G182700	PTHR35274:SF2 - EXPRESSED PROTEIN-RELATED (1 of 2)	-0.85	2.49E-01	-1.40	9.66E-03	-0.82	3.20E-01	-1.10	5.48E-02
Phvul.006G107100	PTHR13168 - ASSOCIATE OF C-MYC AMY-1 (1 of 1)	-0.85	5.59E-01	-2.31	1.26E-02	-0.68	6.85E-01	-2.08	3.06E-02
Phvul.009G148200	PTHR22849//PTHR22849:SF20 - WDSAM1 PROTEIN // SUBFAMILY NOT NAMED (1 of 2)	-0.85	2.13E-01	-1.11	3.57E-02	-0.61	4.44E-01	-0.89	1.02E-01
Phvul.003G086400	no data	-0.86	1.84E-01	-1.37	4.40E-03	-0.77	2.94E-01	-1.43	4.94E-03
Phvul.001G141000	PF03110 - SBP domain (SBP) (1 of 23)	-0.86	1.12E-01	-0.89	4.25E-02	-0.71	2.60E-01	-0.97	3.23E-02
Phvul.007G181600	PTHR27001:SF92 - PROTEIN KINASE DOMAIN-CONTAINING PROTEIN (1 of 1)	-0.86	2.85E-01	-1.83	1.18E-03	-0.91	2.87E-01	-1.52	1.00E-02
Phvul.009G110500	PF08670 - MEKHLA domain (MEKHLA) (1 of 6)	-0.86	1.79E-02	-0.64	5.67E-02	-0.53	2.54E-01	-0.55	1.20E-01
Phvul.011G184300	PTHR11527//PTHR11527:SF144 - SMALL HEAT-SHOCK PROTEIN HSP20 FAMILY // SUBFAMILY NOT NAMED (1 of 2)	-0.86	3.54E-02	-0.78	5.12E-02	-0.61	2.22E-01	-0.85	3.63E-02
Phvul.002G181000	no data	-0.86	3.24E-01	-2.65	5.19E-05	-0.97	2.92E-01	-1.98	2.73E-03
Phvul.006G174100	PTHR27000:SF131 - RECEPTOR LIKE PROTEIN 55 (1 of 2)	-0.86	2.92E-01	-1.18	5.48E-02	-0.56	5.86E-01	-1.55	1.34E-02
Phvul.009G110600	PF07714//PF08263//PF13855 - Protein tyrosine kinase (Pkinase Tyr) // Leucine rich repeat N-terminal domain (LRRNT 2	-0.86	7.39E-03	-0.57	6.66E-02	-0.57	1.55E-01	-0.36	2.78E-01
Phvul.003G192500	PTHR10024:SF201 - C2 CALCIUM/LIPID-BINDING PLANT PHOSPHORIBOSYLTRANSFERASE-LIKE PROTEIN (1 of 1)	-0.86	2.26E-02	-0.79	2.11E-02	-0.29	6.18E-01	-0.53	1.46E-01
Phvul.002G250600	PTHR33021:SF9 - BASIC BLUE PROTEIN (1 of 3)	-0.87	1.89E-01	-1.52	4.93E-03	-0.53	5.20E-01	-0.68	2.35E-01
Phvul.009G224200	3.1.1.11 - Pectinesterase / Pectin methylesterase (1 of 92)	-0.87	3.79E-01	-1.65	1.87E-02	-1.06	2.96E-01	-1.51	4.13E-02
Phvul.008G219100	no data	-0.87	8.52E-02	-1.32	1.13E-03	-1.25	6.74E-03	-1.49	2.66E-04
Phvul.003G090300	no data	-0.87	3.06E-01	-1.54	1.06E-02	-0.74	4.44E-01	-0.96	1.42E-01
Phvul.008G079000	PF06697 - Protein of unknown function (DUF1191) (DUF1191) (1 of 7)	-0.87	1.73E-01	-1.15	1.99E-02	-0.74	3.20E-01	-1.06	3.93E-02
Phvul.011G045800	PTHR10743 - PROTEIN RER1 (1 of 4)	-0.87	2.85E-01	-2.61	1.15E-03	-1.16	1.67E-01	-1.72	2.39E-02
Phvul.002G061800	PTHR10972:SF88 - OXYSTEROL-BINDING PROTEIN-RELATED PROTEIN 2A-RELATED (1 of 1)	-0.87	3.10E-02	-1.05	4.93E-03	-1.03	1.06E-02	-0.61	1.24E-01
Phvul.011G105600	PTHR22880:SF156 - TRANSCRIPTION FACTOR GTE10 (1 of 4)	-0.87	2.61E-01	-1.95	1.17E-03	-0.52	5.90E-01	-1.65	7.07E-03
Phvul.005G168000	PTHR35461:SF1 - GENOMIC DNA, CHROMOSOME 3, P1 CLONE: MSL1 (1 of 2)	-0.87	3.92E-01	-1.62	2.63E-02	-0.82	4.62E-01	-1.84	1.32E-02
Phvul.009G226900	PTHR10795:SF434 - SUBTILISIN SERINE PROTEASE-RELATED (1 of 2)	-0.88	1.04E-01	-1.20	9.30E-03	-0.45	5.22E-01	-1.10	2.04E-02
Phvul.005G111300	PTHR31062:SF11 - XYLOGLUCAN ENDOTRANSGLUCOSYLASE/HYDROLASE PROTEIN 9 (1 of 2)	-0.88	4.18E-01	-1.58	2.93E-02	-0.70	5.85E-01	-1.23	1.06E-01
Phvul.009G232700	PTHR10071//PTHR10071:SF191 - TRANSCRIPTION FACTOR GATA GATA BINDING FACTOR // SUBFAMILY NOT NAMED (1	-0.88	8.59E-02	-1.07	8.90E-03	-0.89	1.06E-01	-0.95	2.86E-02
Phvul.005G117500	PTHR32499:SF3 - FASCICLIN-LIKE ARABINOGALACTAN PROTEIN 15-RELATED (1 of 3)	-0.88	3.45E-01	-2.15	8.73E-04	-0.53	6.54E-01	-1.68	1.23E-02
Phvul.001G011500	PTHR31898:SF1 - TRANSMEMBRANE PROTEIN 136 (1 of 1)	-0.89	3.29E-01	-2.54	5.99E-05	-0.90	3.50E-01	-1.77	6.44E-03
Phvul.003G047700	K02960 - small subunit ribosomal protein S16e (RP-S16e, RPS16) (1 of 2)	-0.89	9.25E-02	-1.15	9.01E-03	-0.90	1.12E-01	-0.83	7.20E-02
Phvul.009G074300	K09285 - AP2-like factor, ANT lineage (OVM, ANT) (1 of 17)	-0.89	2.51E-01	-1.95	1.83E-03	-0.76	3.89E-01	-1.27	4.50E-02
Phvul.011G182900	PF00407 - Pathogenesis-related protein Bet v I family (Bet v 1) (1 of 2)	-0.89	2.05E-02	-1.05	3.12E-03	-0.82	5.06E-02	-0.60	1.16E-01
Phvul.004G123200	PTHR21726:SF51 - GENOMIC DNA, CHROMOSOME 5, P1 CLONE:MTG10 (1 of 2)	-0.90	1.24E-01	-1.48	2.79E-03	-0.32	7.15E-01	-0.42	4.60E-01
Phvul.008G153300	PTHR10217:SF479 - CYCLIC NUCLEOTIDE-GATED ION CHANNEL 16-RELATED (1 of 2)	-0.90	3.00E-01	-1.51	1.75E-02	-0.85	3.68E-01	-1.33	4.40E-02
Phvul.002G152700	K05277 - leucoanthocyanidin dioxygenase (E1.14.11.19) (1 of 1)	-0.90	1.72E-01	-0.65	2.32E-01	-0.45	6.16E-01	-1.05	4.47E-02
Phvul.003G201900	PF13947 - Wall-associated receptor kinase galacturonan-binding (GUB WAK bind) (1 of 65)	-0.90	4.04E-02	-1.27	2.71E-03	-0.79	1.07E-01	-0.82	5.70E-02
Phvul.007G216700	no data	-0.90	5.29E-01	-3.17	2.09E-03	-1.26	3.82E-01	-1.99	5.10E-02
Phvul.010G047000	KOG0513 - Ca2+-independent phospholipase A2 (1 of 16)	-0.90	3.95E-01	-0.69	5.04E-01	-0.76	5.25E-01	-1.86	4.35E-02
Phvul.007G029900	PF00560//PF07714//PF08263 - Leucine Rich Repeat (LRR 1) // Protein tyrosine kinase (Pkinase Tyr) // Leucine rich repe	-0.90	3.88E-01	-1.55	4.25E-02	-0.34	8.23E-01	-1.52	5.54E-02
Phvul.003G131400	PTHR31238:SF21 - GERMIN-LIKE PROTEIN SUBFAMILY 3 MEMBER 3 (1 of 3)	-0.91	6.38E-01	-3.57	3.47E-03	-1.40	4.45E-01	-2.60	3.87E-02
Phvul.001G022700	PTHR31471:SF13 - REMORIN-LIKE PROTEIN (1 of 1)	-0.91	1.54E-01	-1.06	4.82E-02	-0.86	2.29E-01	-0.90	1.08E-01
Phvul.006G125000	PTHR32448:SF25 - FAD-BINDING BERBERINE FAMILY PROTEIN-RELATED (1 of 5)	-0.91	3.00E-01	-1.28	7.54E-02	-0.60	5.87E-01	-1.53	3.66E-02
Phvul.010G123066	PTHR31707:SF55 - PECTINESTERASE 2-RELATED (1 of 2)	-0.91	8.64E-02	-0.99	2.14E-02	-0.60	3.71E-01	-0.84	6.68E-02
Phvul.001G259100	PTHR24198//PTHR24198:SF77 - ANKYRIN REPEAT AND PROTEIN KINASE DOMAIN-CONTAINING PROTEIN // SUBFAMILY	-0.92	2.36E-01	-1.75	1.09E-02	-1.33	7.86E-02	-1.49	3.38E-02
Phvul.001G016700	PTHR31048:SF10 - PATHOGENESIS-RELATED PROTEIN 5-RELATED	-0.93	2.16E-01	-2.26	1.62E-04	-0.64	4.84E-01	-1.27	3.64E-02
Phvul.002G135800	PTHR24177:SF42 - ANKYRIN REPEAT FAMILY PROTEIN (1 of 4)	-0.93	5.31E-01	-2.73	4.36E-03	-0.92	5.60E-01	-2.07	3.86E-02
Phvul.007G138900	PTHR11638//PTHR11638:SF106 - ATP-DEPENDENT CLP PROTEASE // SUBFAMILY NOT NAMED (1 of 2)	-0.93	2.42E-02	-0.90	1.20E-02	-0.75	1.17E-01	-0.92	1.32E-02
Phvul.003G231000	PTHR23042//PTHR23042:SF57 - CIRCADIAN PROTEIN CLOCK/ARNT/BMAL/PAS // SUBFAMILY NOT NAMED (1 of 13)	-0.93	1.10E-01	-1.30	7.48E-03	-0.87	1.85E-01	-0.77	1.39E-01
Phvul.008G075000	PTHR32382:SF5 - FASCICLIN-LIKE ARABINOGALACTAN PROTEIN 10-RELATED (1 of 1)	-0.93	4.11E-01	-2.02	8.17E-03	-0.31	8.56E-01	-1.55	5.20E-02
Phvul.003G080100	1.8.4.10 - Adenylyl-sulfate reductase (thioredoxin) / Thioredoxin-dependent 5'-adenylylsulfate reductase (1 of 2)	-0.93	5.33E-01	-3.01	1.24E-03	-0.97	5.44E-01	-2.39	1.24E-02
Phvul.003G189400	no data	-0.94	3.08E-01	-0.56	5.20E-01	-0.42	7.27E-01	-1.62	4.78E-02
Phvul.008G203900	K14409 - protein SMG7 (SMG7, EST1C) (1 of 3)	-0.94	8.64E-02	-0.90	6.87E-02	-1.33	1.24E-02	-0.64	2.09E-01
Phvul.001G083100	2.3.1.74 - Naringenin-chalcone synthase / Flavonone synthase (1 of 14)	-0.94	1.57E-01	-1.85	2.12E-04	-0.77	3.23E-01	-1.32	1.23E-02
Phvul.008G091400	PTHR23324:SF57 - PATELLIN-6 (1 of 2)	-0.94	2.35E-01	-1.49	1.21E-02	-0.90	3.01E-01	-1.30	3.59E-02
Phvul.011G127500	K01595 - phosphoenolpyruvate carboxylase (ppc) (1 of 6)	-0.94	7.82E-02	-1.44	7.24E-04	-0.93	1.06E-01	-1.43	1.09E-03
Phvul.009G060200	PTHR13878:SF60 - CYTOKININ DEHYDROGENASE 5 (1 of 1)	-0.95	3.54E-01	-2.45	1.94E-03	-1.18	2.64E-01	-1.92	1.55E-02
Phvul.003G284500	PTHR11969:SF22 - TRANSCRIPTION FACTOR BHLH71-RELATED (1 of 1)	-0.95	5.11E-01	-2.57	5.61E-03	-0.76	6.55E-01	-2.54	8.28E-03
Phvul.005G066000	PTHR32444:SF13 - COMITIN (1 of 3)	-0.96	3.60E-01	-1.95	1.26E-02	-0.98	3.86E-01	-1.33	9.29E-02
Phvul.009G228000	PF08879//PF08880 - WRC (WRC) // QLQ (QLQ) (1 of 10)	-0.96	1.43E-01	-1.74	4.91E-03	-1.06	1.27E-01	-1.54	1.54E-02
Phvul.001G001500	PTHR11709:SF90 - MULTI-COPPER OXIDASE TYPE I FAMILY PROTEIN-RELATED (1 of 2)	-0.96	2.26E-01	-1.79	2.01E-03	-1.09	1.89E-01	-1.50	1.41E-02
Phvul.001G164000	PTHR31284:SF15 - HAD SUPERFAMILY, SUBFAMILY IIIB ACID PHOSPHATASE (1 of 1)	-0.96	1.34E-01	-2.07	2.85E-05	-1.02	1.39E-01	-1.30	1.23E-02
Phvul.006G064800	PTHR11977:SF25 - VILLIN-1 (1 of 1)	-0.96	1.72E-02	-0.95	9.23E-03	-0.60	2.47E-01	-0.70	6.85E-02
Phvul.003G056000	1.11.1.7 - Peroxidase / Lactoperoxidase (1 of 96)	-0.97	2.46E-01	-1.88	3.00E-03	-0.85	3.72E-01	-0.79	2.53E-01
Phvul.002G070800	no data	-0.97	3.34E-02	-1.30	2.08E-03	-0.60	2.92E-01	-0.61	1.63E-01
Phvul.001G198300	K03363 - cell division cycle 20, cofactor of APC complex (CDC20) (1 of 4)	-0.97	6.77E-02	-0.80	6.21E-02	-0.64	3.12E-01	-1.07	1.46E-02
Phvul.001G138700	no data	-0.97	3.80E-01	-0.48	6.63E-01	-1.95	4.66E-02	-0.67	5.24E-01
Phvul.003G258300	PTHR23421:SF79 - BETA-GALACTOSIDASE 3 (1 of 2)	-0.98	2.54E-01	-2.23	2.82E-04	-0.85	3.78E-01	-1.71	7.00E-03
Phvul.011G034200	PTHR23180:SF244 - ADP-RIBOSYLATION FACTOR GTPASE-ACTIVATING PROTEIN AGD2-RELATED Pathogenesis-related	-0.98	3.90E-01	-2.19	6.17E-03	-0.60	6.76E-01	-1.48	7.49E-02
Phvul.008G229500	PTHR11709//PTHR11709:SF80 - MULTI-COPPER OXIDASE // SUBFAMILY NOT NAMED (1 of 1)	-0.98	7.04E-01	-3.24	2.24E-02	-0.73	8.10E-01	-3.40	1.94E-02
Phvul.007G212000	K01051 - pectinesterase (E3.1.1.11) (1 of 52)	-0.98	2.50E-01	-1.95	2.63E-03	-0.68	5.19E-01	-1.18	8.25E-02
Phvul.009G121700	no data	-0.98	6.83E-02	-1.56	4.40E-04	-0.84	1.72E-01	-1.27	6.29E-03
Phvul.009G120000	PTHR10795//PTHR10795:SF442 - PROPROTEIN CONVERTASE SUBTILISIN/KEXIN // SUBFAMILY NOT NAMED (1 of 2)	-0.99	1.73E-01	-1.61	1.07E-02	-0.68	4.38E-01	-0.87	1.83E-01
Phvul.003G157100	PTHR16223:SF28 - TRANSCRIPTION FACTOR BHLH66-RELATED (1 of 2)	-0.99	1.85E-02	-0.27	5.83E-01	-0.49	3.96E-01	-0.21	6.73E-01
Phvul.008G181800	K10395 - kinesin family member 4/21/27 (KIF4 21 27) (1 of 5)	-0.99	1.00E-01	-0.62	3.40E-01	-1.21	4.68E-02	-0.74	2.37E-01
Phvul.009G070600	PTHR31807:SF8 - QWRF MOTIF-CONTAINING PROTEIN 9 (1 of 2)	-0.99	5.36E-03	-0.87	3.39E-02	-0.57	2.09E-01	-0.25	5.89E-01
Phvul.008G163500	PTHR31636:SF25 - SCARECROW-LIKE PROTEIN 26 (1 of 2)	-0.99	2.37E-01	-1.91	3.08E-03	-1.16	1.79E-01	-1.36	4.39E-02
Phvul.011G062500	PF01535//PF12854//PF13041 - PPR repeat (PPR) // PPR repeat (PPR 1) // PPR repeat family (PPR 2) (1 of 29)	-0.99	1.58E-01	-2.02	6.73E-04	-0.71	4.08E-01	-1.08	7.61E-02
Phvul.005G046201	1.11.1.7 - Peroxidase / Lactoperoxidase (1 of 96)	-1.00	1.95E-01	-2.02	1.15E-03	-1.26	1.10E-01	-1.32	3.89E-02
Phvul.009G237700	PF04782 - Protein of unknown function (DUF632) (DUF632) (1 of 15)	-1.00	1.38E-02	-0.61	1.17E-01	-0.50	3.46E-01	-0.45	2.65E-01
Phvul.003G193100	PF00069//PF00560//PF08263 - Protein kinase domain (Pkinase) // Leucine Rich Repeat (LRR 1) // Leucine rich repeat N	-1.00	1.06E-01	-1.36	1.12E-02	-0.99	1.40E-01	-1.35	1.42E-02
Phvul.L002446	PTHR33044:SF7 - NON-SPECIFIC LIPID TRANSFER PROTEIN GPI-ANCHORED 1 (1 of 2)	-1.00	3.08E-01	-2.55	9.39E-04	-0.72	5.32E-01	-2.40	2.39E-03
Phvul.008G123000	no data	-1.01	8.90E-03	-1.09	2.64E-03	-1.12	3.85E-03	-0.89	1.96E-02
Phvul.001G027700	PTHR23083//PTHR23083:SF431 - TETRATRICOPEPTIDE REPEAT PROTEIN, TPR // SUBFAMILY NOT NAMED (1 of 2)	-1.01	1.85E-02	-0.55	3.12E-01	-0.78	1.14E-01	-0.43	4.41E-01
Phvul.007G063500	PTHR11062:SF52 - GLYCOSYLTRANSFERASE 18 (1 of 1)	-1.01	1.60E-01	-1.29	2.25E-02	-0.80	3.45E-01	-1.31	2.46E-02
Phvul.002G131500	PTHR22870//PTHR22870:SF190 - REGULATOR OF CHROMOSOME CONDENSATION // SUBFAMILY NOT NAMED (1 of 4)	-1.01	2.48E-02	-0.79	5.68E-02	-0.53	3.62E-01	-0.26	5.88E-01
Phvul.001G266100	PTHR11062:SF110 - EXOSTOSIN FAMILY PROTEIN (1 of 1)	-1.02	1.95E-01	-2.40	8.89E-04	-0.95	2.78E-01	-1.67	1.77E-02
Phvul.008G013300	PTHR10795:SF387 - SUBTILISIN-LIKE SERINE PROTEASE-RELATED (1 of 2)	-1.02	1.68E-02	-2.24	3.53E-04	-0.96	3.67E-02	-1.47	1.22E-02
Phvul.008G287700	PTHR32077:SF3 - FASCICLIN-LIKE ARABINOGALACTAN PROTEIN 7 (1 of 1)	-1.02	1.04E-01	-1.75	3.45E-04	-0.89	2.13E-01	-1.35	8.16E-03
Phvul.006G113100	PF13639//PF13947//PF14380 - Ring finger domain (zf-RING 2) // Wall-associated receptor kinase galacturonan-binding	-1.02	1.63E-02	-0.84	6.39E-02	-0.62	2.45E-01	-0.02	9.76E-01
Phvul.003G079900	4.2.1.51//4.2.1.91 - Arogenate dehydratase / Carboxycyclohexadienyl dehydratase (1 of 5)	-1.02	3.21E-01	-2.27	1.73E-03	-0.72	5.69E-01	-1.71	2.39E-02
Phvul.011G142200	PTHR27003:SF26 - CALCIUM/CALMODULIN-REGULATED RECEPTOR-LIKE KINASE (1 of 3)	-1.03	4.87E-02	-1.33	2.51E-03	-0.92	1.12E-01	-1.32	3.96E-03
Phvul.001G249100	PTHR33257:SF5 - EXPRESSED PROTEIN (1 of 5)	-1.03	4.35E-01	-2.81	4.09E-03	-0.61	7.37E-01	-2.85	5.18E-03
Phvul.002G145900	PTHR11926//PTHR11926:SF220 - GLUCOSYL/GLUCURONOSYL TRANSFERASES // SUBFAMILY NOT NAMED (1 of 6)	-1.04	1.33E-02	-0.64	1.41E-01	-0.76	1.26E-01	-0.49	2.69E-01
Phvul.009G134800	PF14416 - PMR5 N terminal Domain (PMR5N) (1 of 64)	-1.04	6.80E-02	-1.26	7.69E-03	-0.62	3.98E-01	-0.87	8.50E-02
Phvul.007G242300	PTHR24361:SF403 - PROTEIN KINASE FAMILY PROTEIN-RELATED (1 of 1)	-1.04	3.71E-01	-2.22	1.05E-02	-0.64	6.67E-01	-1.23	1.61E-01
Phvul.007G056100	PTHR11558//PTHR11558:SF30 - SPERMIDINE/SPERMINE SYNTHASE // SUBFAMILY NOT NAMED (1 of 2)	-1.04	5.90E-02	-1.05	2.42E-02	-0.83	2.02E-01	-0.87	7.62E-02
Phvul.009G096900	PTHR10209//PTHR10209:SF119 - OXIDOREDUCTASE, 2OG-FE II OXYGENASE FAMILY PROTEIN // SUBFAMILY NOT NAME	-1.04	2.67E-01	-1.64	4.65E-02	-0.94	3.67E-01	-0.92	2.91E-01

Phvul.004G118100	K10664 - E3 ubiquitin-protein ligase ATL6/9/15/31/42/55 [EC:6.3.2.19] (ATL6S) (1 of 10)	-1.05	1.67E-02	-0.73	6.74E-02	-0.60	3.02E-01	-0.54	2.10E-01
Phvul.004G132000	PTHR31235:SF22 - PEROXIDASE 47 (1 of 2)	-1.05	3.21E-01	-2.13	4.04E-03	-0.86	4.84E-01	-1.61	3.91E-02
Phvul.006G183700	PF14383 - DUF761-associated sequence motif (VARLMGL) (1 of 22)	-1.05	1.35E-01	-1.90	2.09E-03	-0.33	7.35E-01	-0.84	1.55E-01
Phvul.003G086500	1.1.1.35//4.2.1.17//4.2.1.55//5.1.2.3//5.3.3.8 - 3-hydroxyacyl-CoA dehydrogenase / Beta-keto-reductase // Enoyl-CoA h	-1.05	1.10E-02	-1.03	4.52E-03	-0.76	1.26E-01	-0.70	7.47E-02
Phvul.002G158300	no data	-1.05	8.27E-02	-1.27	2.74E-02	-1.12	7.93E-02	-1.22	3.62E-02
Phvul.L004500	PTHR32227:SF29 - GLUCAN ENDO-1,3-BETA-GLUCOSIDASE 10-RELATED (1 of 1)	-1.05	1.23E-01	-1.52	1.85E-02	-0.50	5.71E-01	-0.79	2.23E-01
Phvul.001G024900	PTHR10177//PTHR10177:SF242 - CYCLINE // SUBFAMILY NOT NAMED (1 of 2)	-1.05	1.69E-01	-1.34	2.78E-02	-0.65	5.07E-01	-0.66	3.27E-01
Phvul.011G107000	PF03000 - NPH3 family (NPH3) (1 of 37)	-1.05	3.91E-01	-2.37	3.96E-03	-0.45	8.02E-01	-1.71	4.87E-02
Phvul.003G209000	PTHR33595:SF3 - EMB (1 of 3)	-1.05	8.04E-02	-2.46	5.67E-04	-0.71	3.20E-01	-0.40	5.64E-01
Phvul.008G022700	PTHR27001:SF217 - PROTEIN NSP-INTERACTING KINASE 1 (1 of 2)	-1.05	2.54E-02	-1.30	1.23E-03	-0.84	1.29E-01	-1.01	1.79E-02
Phvul.009G050700	no data	-1.06	3.45E-01	-2.05	9.56E-03	-0.58	7.00E-01	-2.16	7.25E-03
Phvul.005G117200	PTHR24115:SF388 - KINESIN FAMILY MEMBER C1 (1 of 1)	-1.06	2.16E-02	0.23	6.97E-01	-0.66	2.47E-01	0.08	9.07E-01
Phvul.005G097600	PTHR32227:SF91 - GLUCAN ENDO-1,3-BETA-GLUCOSIDASE-LIKE PROTEIN 1 (1 of 2)	-1.07	1.85E-02	-0.92	1.10E-01	-0.28	6.85E-01	0.07	9.23E-01
Phvul.009G227800	PTHR31238:SF21 - GERMIN-LIKE PROTEIN SUBFAMILY 3 MEMBER 3 (1 of 3)	-1.07	5.94E-01	-2.94	1.97E-02	-1.08	6.17E-01	-2.13	1.13E-01
Phvul.006G160600	PTHR35725:SF2 - CLASSICAL ARABINOGALACTAN PROTEIN 25 (1 of 1)	-1.07	3.73E-01	-1.68	6.73E-02	-0.87	5.23E-01	-2.08	2.87E-02
Phvul.006G041300	PF01535//PF12854//PF13041//PF14432 - PPR repeat (PPR) // PPR repeat (PPR 1) // PPR repeat family (PPR 2) // DYW	-1.07	3.73E-03	-1.12	6.18E-03	-0.73	1.02E-01	-0.44	3.21E-01
Phvul.001G163600	no data	-1.07	2.63E-01	-1.57	3.49E-02	-0.44	7.54E-01	-1.40	7.07E-02
Phvul.002G250700	PTHR10071:SF159 - GATA TRANSCRIPTION FACTOR 26-RELATED (1 of 3)	-1.08	3.75E-01	-2.00	2.02E-02	-1.08	4.03E-01	-1.59	7.55E-02
Phvul.007G007200	PTHR18937//PTHR18937:SF221 - STRUCTURAL MAINTENANCE OF CHROMOSOMES SMC FAMILY MEMBER // SUBFAMIL	-1.09	1.98E-02	-0.39	4.86E-01	-0.82	1.32E-01	-0.14	8.29E-01
Phvul.002G188600	PTHR13301:SF59 - CELLULOSE SYNTHASE A CATALYTIC SUBUNIT 7 [UDP-FORMING] (1 of 3)	-1.09	1.38E-01	-2.58	1.86E-04	-1.18	1.30E-01	-1.16	7.86E-02
Phvul.009G077600	no data	-1.09	4.54E-01	-3.54	5.00E-03	-0.62	7.33E-01	-4.32	1.86E-03
Phvul.003G149300	PTHR33228:SF5 - PROTEIN GLUTAMINE DUMPER 1-RELATED (1 of 1)	-1.09	1.68E-02	-0.25	6.30E-01	-0.57	3.43E-01	-0.08	8.91E-01
Phvul.003G028600	PTHR12385:SF36 - PLASMA-MEMBRANE CHOLINE TRANSPORTER-LIKE PROTEIN (1 of 2)	-1.10	1.70E-03	-1.37	3.41E-04	-0.85	3.45E-02	-1.24	1.40E-03
Phvul.002G220800	no data	-1.10	6.60E-01	-2.88	5.75E-02	-0.46	8.94E-01	-3.37	3.16E-02
Phvul.008G228400	no data	-1.10	1.73E-01	-1.54	2.89E-02	-1.05	2.37E-01	-0.93	1.98E-01
Phvul.009G067200	PF00069//PF00560//PF13855 - Protein kinase domain (Pkinase) // Leucine Rich Repeat (LRR 1) // Leucine rich repeat (L	-1.11	1.71E-01	-1.99	2.08E-03	-0.73	4.66E-01	-2.02	2.47E-03
Phvul.001G138500	PTHR27008:SF41 - LEUCINE-RICH REPEAT PROTEIN KINASE-LIKE PROTEIN (1 of 1)	-1.11	1.33E-01	-1.63	5.90E-03	-0.64	5.12E-01	-1.35	2.93E-02
Phvul.008G186900	PF00069//PF00560//PF08263//PF13855 - Protein kinase domain (Pkinase) // Leucine Rich Repeat (LRR 1) // Leucine ric	-1.11	2.52E-01	-1.87	3.94E-02	-1.88	3.80E-02	-1.27	1.61E-01
Phvul.003G232600	K09285 - AP2-like factor, ANT lineage (OVM, ANT) (1 of 17)	-1.12	2.07E-03	-0.94	1.77E-02	-0.87	3.45E-02	-0.55	1.90E-01
Phvul.007G224300	PF11961 - Domain of unknown function (DUF3475) (DUF3475) (1 of 15)	-1.12	7.83E-02	-1.68	1.48E-03	-0.61	4.62E-01	-0.79	1.61E-01
Phvul.010G153700	PTHR23111//PTHR23111:SF34 - ZINC FINGER PROTEIN // SUBFAMILY NOT NAMED (1 of 1)	-1.12	4.36E-04	-0.99	2.05E-03	-0.35	4.81E-01	-0.65	5.46E-02
Phvul.010G071300	PTHR31636:SF11 - SCARECROW-LIKE PROTEIN 22-RELATED (1 of 3)	-1.13	4.17E-02	0.04	9.63E-01	-0.83	2.04E-01	0.24	6.86E-01
Phvul.007G161600	no data	-1.13	9.10E-02	-1.28	2.73E-02	-1.08	1.38E-01	-0.74	2.31E-01
Phvul.002G086300	1.14.19.4 - Delta(8)-fatty-acid desaturase / SLD (1 of 3)	-1.14	4.01E-02	-1.80	1.24E-03	-1.05	8.34E-02	-1.67	3.38E-03
Phvul.005G165900	PTHR27001:SF102 - LYSM DOMAIN RECEPTOR-LIKE KINASE 3 (1 of 2)	-1.14	8.58E-02	-1.13	1.13E-01	-1.34	4.63E-02	-1.95	8.89E-03
Phvul.002G011500	K11492 - condensin-2 complex subunit G2 (NCAPG2, LUZP5) (1 of 1)	-1.14	1.59E-01	-0.78	2.91E-01	-1.91	1.48E-02	-1.82	1.41E-02
Phvul.009G053500	PTHR24056:SF181 - PLASTID-LIPID-ASSOCIATED PROTEIN 14, CHLOROPLASTIC-RELATED (1 of 1)	-1.15	1.55E-02	-1.22	2.97E-03	-1.09	3.45E-02	-1.03	1.78E-02
Phvul.008G123600	PTHR27008:SF28 - KINASE-LIKE PROTEIN TMKL1-RELATED (1 of 1)	-1.15	9.63E-02	-1.13	4.97E-02	-1.01	2.06E-01	-0.63	3.16E-01
Phvul.001G031400	PTHR33088:SF9 - HYDROXYPROLINE-RICH GLYCOPROTEIN FAMILY PROTEIN (1 of 1)	-1.15	1.85E-02	-0.84	1.44E-01	-0.74	2.20E-01	-0.71	2.14E-01
Phvul.003G000900	PTHR10641:SF538 - LD18233P (1 of 1)	-1.16	1.26E-01	-1.75	1.05E-02	-0.79	3.98E-01	-1.13	1.03E-01
Phvul.005G109600	K18812 - cyclin D6, plant (CYCD6) (1 of 2)	-1.16	1.21E-02	-1.21	8.19E-03	-0.66	2.60E-01	-0.43	3.89E-01
Phvul.001G265300	PTHR31062:SF34 - XYLOGLUCAN ENDOTRANSGLUCOSYLASE/HYDROLASE PROTEIN 15-RELATED (1 of 2)	-1.16	6.83E-02	-1.30	1.49E-02	-0.82	2.90E-01	-0.98	8.31E-02
Phvul.009G104850	no data	-1.16	4.97E-01	-2.52	4.79E-02	-1.51	3.62E-01	-1.97	1.29E-01
Phvul.003G038900	K13100 - pre-mRNA-splicing factor CWC22 (CWC22) (1 of 4)	-1.17	2.66E-02	-1.31	1.04E-02	-1.05	7.07E-02	-0.78	1.39E-01
Phvul.010G013600	PTHR27004:SF37 - DISEASE RESISTANCE FAMILY PROTEIN/LRR FAMILY PROTEIN-RELATED (1 of 10)	-1.17	5.62E-01	1.89	3.05E-02	-0.47	8.58E-01	1.45	1.29E-01
Phvul.002G014100	PTHR37209:SF2 - LYSINE-RICH ARABINOGALACTAN PROTEIN 17-RELATED (1 of 2)	-1.17	4.54E-01	-3.05	3.71E-03	-0.59	7.84E-01	-2.77	1.07E-02
Phvul.007G166400	PTHR35725:SF1 - CLASSICAL ARABINOGALACTAN PROTEIN 26 (1 of 1)	-1.17	7.98E-02	-1.52	8.74E-03	-0.86	2.85E-01	-1.06	7.70E-02
Phvul.009G029500	PTHR17985 - SER/THR-RICH PROTEIN T10 IN DGCR REGION (1 of 2)	-1.18	7.55E-02	-1.69	1.87E-03	-0.28	7.81E-01	-1.24	2.55E-02
Phvul.005G099500	PTHR32077:SF6 - FASCICLIN-LIKE ARABINOGALACTAN PROTEIN 13-RELATED (1 of 1)	-1.18	1.61E-01	-1.58	1.21E-02	-0.65	5.67E-01	-0.88	2.04E-01
Phvul.008G239300	PTHR10766//PTHR10766:SF58 - TRANSMEMBRANE 9 SUPERFAMILY PROTEIN // SUBFAMILY NOT NAMED (1 of 3)	-1.19	4.23E-02	-0.74	2.11E-01	-1.35	2.33E-02	-1.15	4.67E-02
Phvul.005G081200	PTHR12956//PTHR12956:SF30 - ALKALINE CERAMIDASE-RELATED // SUBFAMILY NOT NAMED (1 of 1)	-1.19	2.24E-02	-0.61	2.50E-01	-1.12	4.74E-02	-0.73	1.59E-01
Phvul.006G057400	PTHR32382:SF4 - FASCICLIN-LIKE ARABINOGALACTAN PROTEIN 1 (1 of 2)	-1.19	3.57E-01	-3.24	3.29E-04	-0.72	6.66E-01	-2.36	1.00E-02
Phvul.002G251800	PTHR10694:SF41 - JUMONJI DOMAIN-CONTAINING PROTEIN 18-RELATED (1 of 2)	-1.19	2.44E-02	-0.75	1.83E-01	-0.96	1.12E-01	-0.86	1.22E-01
Phvul.003G293500	K10400 - kinesin family member 15 (KIF15) (1 of 6)	-1.19	1.39E-03	-0.89	3.92E-02	-1.35	4.36E-04	-0.52	2.48E-01
Phvul.004G066300	PTHR11614:SF86 - HYDROLASE, ALPHA/BETA FOLD FAMILY PROTEIN (1 of 2)	-1.19	5.83E-02	-1.96	2.72E-04	-1.26	5.40E-02	-1.97	3.22E-04
Phvul.009G188251	PTHR23042:SF51 - TRANSCRIPTION FACTOR BHLH47 (1 of 2)	-1.19	1.83E-01	-1.68	1.41E-02	-0.91	3.94E-01	-1.18	1.00E-01
Phvul.002G211800	no data	-1.20	4.25E-01	-3.22	1.33E-03	-1.45	3.27E-01	-2.55	1.25E-02
Phvul.001G177400	PTHR31223:SF17 - CYTOKININ RIBOSIDE 5'-MONOPHOSPHATE PHOSPHORIBOHYDROLASE LOG3-RELATED (1 of 3)	-1.20	1.68E-02	-0.71	2.33E-01	-0.40	5.76E-01	-0.19	7.76E-01
Phvul.007G185701	PTHR11079//PTHR11079:SF90 - CYTOSINE DEAMINASE // SUBFAMILY NOT NAMED (1 of 4)	-1.20	4.80E-02	-1.20	4.63E-02	-1.13	9.50E-02	-1.59	9.68E-03
Phvul.002G314600	PTHR23180:SF200 - ARF-GAP DOMAIN AND FG REPEAT-CONTAINING PROTEIN 1 (1 of 2)	-1.21	3.44E-01	-2.22	2.43E-02	-0.98	4.87E-01	-1.07	2.81E-01
Phvul.002G333000	PF14383 - DUF761-associated sequence motif (VARLMGL) (1 of 22)	-1.21	3.96E-02	-0.54	3.70E-01	-0.45	5.95E-01	-0.23	7.31E-01
Phvul.001G183900	PTHR24012:SF976 - POLYADENYLATE-BINDING PROTEIN 7 (1 of 2)	-1.21	8.95E-02	-1.43	3.41E-02	-0.91	2.73E-01	-1.24	6.85E-02
Phvul.008G232200	PTHR24073:SF582 - P-LOOP CONTAINING NUCLEOSIDE TRIPHOSPHATE HYDROLASES SUPERFAMILY PROTEIN-RELATED (	-1.21	2.19E-03	-0.85	3.15E-02	-0.51	3.36E-01	-0.42	3.15E-01
Phvul.007G081100	no data	-1.21	1.38E-01	-2.10	2.11E-03	-1.18	1.87E-01	-1.77	1.01E-02
Phvul.001G010100	PTHR31218:SF53 - PROTEIN WALLS ARE THIN 1 (1 of 3)	-1.21	2.69E-02	-0.24	7.05E-01	-0.95	1.44E-01	-0.25	6.85E-01
Phvul.004G019900	PTHR23201//PTHR23201:SF18 - EXTENSIN, PROLINE-RICH PROTEIN // SUBFAMILY NOT NAMED (1 of 4)	-1.21	2.49E-01	-1.82	2.47E-02	-1.67	9.25E-02	-1.40	1.05E-01
Phvul.011G020100	PTHR13301:SF72 - CELLULOSE SYNTHASE-LIKE PROTEIN D3 (1 of 2)	-1.23	8.73E-02	-1.33	3.28E-02	-1.24	1.05E-01	-1.64	1.10E-02
Phvul.011G073900	PTHR35460:SF1 - TRANSLATION ELONGATION FACTOR EF-1 ALPHA (1 of 1)	-1.23	4.07E-02	-0.68	3.08E-01	-0.68	3.53E-01	-0.58	3.84E-01
Phvul.002G063400	PTHR31589:SF19 - CARBOXYL-TERMINAL PEPTIDASE-RELATED (1 of 1)	-1.23	9.65E-03	-1.31	2.30E-03	-0.86	1.36E-01	-0.57	2.22E-01
Phvul.002G287500	PTHR23270:SF7 - NUCLEIC ACID-BINDING, OB-FOLD-LIKE PROTEIN (1 of 1)	-1.23	5.02E-02	-1.23	3.81E-02	-0.59	4.84E-01	-1.04	8.76E-02
Phvul.007G091400	no data	-1.23	2.88E-02	-1.03	5.24E-02	-1.19	5.25E-02	-1.05	5.09E-02
Phvul.006G130400	PTHR31388:SF22 - PEROXIDASE 22-RELATED (1 of 10)	-1.23	2.45E-01	-2.39	1.87E-03	-1.07	3.60E-01	-1.62	3.92E-02
Phvul.002G239500	K14502 - protein brassinosteroid insensitive 2 (BIN2) (1 of 5)	-1.24	9.88E-03	-1.14	2.97E-02	-1.08	4.11E-02	-1.01	5.83E-02
Phvul.006G124500	K02935 - large subunit ribosomal protein L7/L12 (RP-L7, MRPL12, rplL) (1 of 7)	-1.24	2.99E-02	-1.59	4.52E-03	-1.00	1.27E-01	-1.26	2.82E-02
Phvul.003G089001	PF01535//PF12854//PF13041 - PPR repeat (PPR) // PPR repeat (PPR 1) // PPR repeat family (PPR 2) (1 of 29)	-1.24	4.80E-02	-0.64	3.44E-01	-0.60	4.56E-01	-0.31	6.72E-01
Phvul.009G157200	PTHR23086:SF33 - PHOSPHATIDYLINOSITOL 4-PHOSPHATE 5-KINASE 1-RELATED (1 of 3)	-1.24	5.97E-02	-1.58	3.42E-03	-0.93	2.38E-01	-1.33	1.89E-02
Phvul.002G307200	PTHR31079:SF3 - NAC DOMAIN CONTAINING PROTEIN 75-RELATED (1 of 2)	-1.25	1.39E-01	-1.64	2.82E-02	-0.54	6.49E-01	-1.63	3.32E-02
Phvul.008G271700	PF14368 - Probable lipid transfer (LTP 2) (1 of 66)	-1.25	2.68E-02	-1.38	3.76E-03	-0.90	1.90E-01	-1.18	1.90E-02
Phvul.002G100200	1.3.5.1 - Succinate dehydrogenase (quinone) / Succinic dehydrogenase (1 of 7)	-1.25	3.74E-01	-2.07	4.06E-02	-1.75	2.26E-01	-3.03	4.10E-03
Phvul.006G035100	no data	-1.26	1.96E-02	-1.01	6.88E-02	-1.18	4.34E-02	-0.39	5.25E-01
Phvul.010G032300	PTHR11771//PTHR11771:SF59 - LIPOXYGENASE // SUBFAMILY NOT NAMED (1 of 1)	-1.26	4.20E-03	-0.48	3.32E-01	-0.34	6.23E-01	-0.14	8.13E-01
Phvul.001G212200	PTHR33052:SF19 - AGAA.5 (1 of 2)	-1.26	3.21E-01	-2.51	3.88E-02	-1.62	1.87E-01	-2.27	5.56E-02
Phvul.003G029700	PTHR31042:SF10 - CORE-2/I-BRANCHING BETA-1,6-N-ACETYLGLUCOSAMINYLTRANSFERASE-LIKE PROTEIN (1 of 2)	-1.26	1.05E-01	-1.58	1.24E-02	-0.71	4.80E-01	-1.27	5.59E-02
Phvul.003G160300	no data	-1.27	3.16E-01	-2.29	1.28E-02	-0.75	6.31E-01	-1.46	1.21E-01
Phvul.008G037500	PF01357//PF03330 - Pollen allergen (Pollen allerg 1) // Rare lipoprotein A (RlpA)-like double-psi beta-barrel (DPBB 1) (1	-1.27	4.38E-01	-3.23	3.54E-03	-1.22	4.89E-01	-2.87	1.20E-02
Phvul.008G212700	1.2.3.1 - Aldehyde oxidase / Retinal oxidase (1 of 2)	-1.27	2.87E-01	-4.13	1.21E-05	-1.09	4.20E-01	-2.95	1.18E-03
Phvul.003G128900	PTHR10772:SF0 - 20 KDA CHAPERONIN, CHLOROPLASTIC (1 of 2)	-1.27	1.36E-02	-1.80	7.98E-05	-1.18	3.60E-02	-1.42	2.70E-03
Phvul.009G029300	PTHR30620:SF8 - BETA-D-XYLOSIDASE 7-RELATED (1 of 3)	-1.28	2.74E-03	-1.62	2.10E-04	-1.04	3.22E-02	-0.99	2.53E-02
Phvul.008G128400	PTHR10825:SF5 - E3 UBIQUITIN-PROTEIN LIGASE RING1 (1 of 1)	-1.28	1.46E-02	-1.41	2.16E-03	-0.79	2.47E-01	-1.03	3.50E-02
Phvul.002G083800	PTHR10641:SF473 - MYB TRANSCRIPTION FACTOR (1 of 5)	-1.28	1.12E-01	-1.83	2.51E-02	-0.25	8.40E-01	-1.02	2.05E-01
Phvul.006G080200	PTHR11260//PTHR11260:SF16 - GLUTATHIONE S-TRANSFERASE, GST, SUPERFAMILY, GST DOMAIN CONTAINING // SUBF	-1.29	8.51E-02	-1.79	3.66E-03	-1.11	1.99E-01	-2.03	1.38E-03
Phvul.005G038700	PTHR12452 - 42-9-9 PROTEIN-RELATED (1 of 2)	-1.30	1.98E-02	-1.57	1.12E-03	-1.22	4.46E-02	-1.00	5.04E-02
Phvul.003G242100	PTHR31580:SF3 - FILAMENT-LIKE PLANT PROTEIN 7 (1 of 2)	-1.30	2.08E-02	-1.29	1.53E-02	-1.01	1.27E-01	-0.46	4.53E-01
Phvul.002G179900	PTHR11629//PTHR11629:SF69 - VACUOLAR PROTON ATPASES // SUBFAMILY NOT NAMED (1 of 2)	-1.30	8.32E-02	-1.48	3.02E-02	-0.96	2.68E-01	-0.95	1.74E-01
Phvul.001G064300	1.14.19.2 - Stearoyl-[acyl-carrier-protein] 9-desaturase / Stearyl-ACP desaturase (1 of 4)	-1.32	4.25E-02	-2.29	2.91E-04	-1.27	6.79E-02	-1.41	2.28E-02
Phvul.005G110250	no data	-1.33	4.68E-02	0.05	9.49E-01	-1.01	1.88E-01	-0.35	6.03E-01
Phvul.002G132600	no data	-1.33	1.44E-04	-0.77	5.02E-02	-0.72	1.04E-01	-0.35	4.23E-01
Phvul.001G171800	no data	-1.34	2.83E-01	-4.76	6.59E-05	-1.57	2.27E-01	-1.77	6.08E-02
Phvul.002G157600	6.3.2.2 - Glutamate--cysteine ligase / Gamma-glutamylcysteine synthetase (1 of 2)	-1.35	1.00E-01	-1.44	3.21E-02	-1.11	2.39E-01	-0.93	1.95E-01
Phvul.002G028500	PTHR23500:SF154 - POLYOL TRANSPORTER 3-RELATED (1 of 5)	-1.37	6.00E-02	1.25	9.92E-03	0.36	6.91E-01	0.41	5.19E-01
Phvul.002G261800	PTHR31083:SF6 - PROTEIN UPSTREAM OF FLC (1 of 1)	-1.38	4.45E-03	-0.42	5.25E-01	-0.72	2.31E-01	-0.02	9.83E-01
Phvul.009G023900	PTHR27001:SF156 - PROTEIN KINASE FAMILY PROTEIN (1 of 3)	-1.39	4.05E-02	-1.03	2.09E-01	-0.73	3.98E-01	-0.15	8.79E-01
Phvul.005G025900	PTHR13683:SF258 - ASPARTYL PROTEASE FAMILY PROTEIN-RELATED (1 of 2)	-1.39	2.56E-01	-2.59	2.86E-03	-1.15	4.07E-01	-2.40	7.47E-03

Phvul.001G096200	no data	-1.40	6.98E-02	-1.09	1.01E-01	-1.17	1.83E-01	-1.58	2.00E-02
Phvul.007G165900	PTHR10168:SF57 - GLUTAREDOXIN-C13-RELATED (1 of 3)	-1.40	3.08E-01	-3.02	2.12E-03	-1.70	1.69E-01	-1.94	4.40E-02
Phvul.005G104600	PTHR31692:SF5 - EXPANSIN-B1-RELATED (1 of 2)	-1.41	1.80E-01	-2.40	2.90E-03	-1.27	2.64E-01	-2.76	8.14E-04
Phvul.007G078000	PTHR24223:SF192 - ABC TRANSPORTER C FAMILY MEMBER 10 (1 of 4)	-1.43	1.05E-01	-2.53	2.94E-03	-0.77	4.63E-01	-1.63	4.07E-02
Phvul.002G092400	PF04669 - Polysaccharide biosynthesis (Polysacc synt 4) (1 of 9)	-1.45	2.80E-02	-1.37	3.68E-02	-0.97	2.46E-01	-1.08	1.03E-01
Phvul.005G174700	3.1.1.5 - Lysophospholipase / Phospholipase B (1 of 5)	-1.45	4.32E-02	-2.07	2.14E-02	-1.68	2.72E-02	-2.85	3.96E-03
Phvul.008G112800	PTHR27005:SF11 - WALL-ASSOCIATED RECEPTOR KINASE-LIKE 15-RELATED (1 of 1)	-1.45	1.54E-01	-0.63	5.32E-01	-2.11	3.86E-02	-1.57	1.04E-01
Phvul.009G157900	PTHR23324:SF48 - PATELLIN-3-RELATED (1 of 3)	-1.46	1.16E-02	-1.87	1.84E-04	-1.03	1.53E-01	-1.49	4.39E-03
Phvul.003G078900	PTHR31235:SF69 - PEROXIDASE 42 (1 of 1)	-1.47	2.69E-02	-0.35	6.83E-01	-1.46	3.90E-02	-0.69	3.73E-01
Phvul.010G144200	3.1.3.2 - Acid phosphatase / Phosphomonoesterase (1 of 34)	-1.47	2.40E-01	-3.14	4.90E-03	-0.81	5.82E-01	-1.62	1.31E-01
Phvul.006G207800	K17470 - sulfate transporter 1, high-affinity (SULTR1) (1 of 3)	-1.48	4.20E-02	-1.57	4.50E-02	-1.50	4.88E-02	-2.30	6.30E-03
Phvul.002G032500	PTHR23172:SF19 - AUXILLIN, ISOFORM A (1 of 2)	-1.49	1.49E-03	-0.84	1.88E-01	-1.48	2.73E-03	-1.20	5.64E-02
Phvul.009G179500	PF03181 - BURP domain (BURP) (1 of 11)	-1.49	7.05E-03	-0.75	2.36E-01	-0.50	5.15E-01	-0.55	3.94E-01
Phvul.004G174500	PTHR24361:SF336 - MITOGEN-ACTIVATED PROTEIN KINASE KINASE 6 (1 of 1)	-1.49	1.57E-02	-1.37	9.01E-02	-1.16	9.16E-02	-0.51	5.39E-01
Phvul.005G121200	no data	-1.51	1.44E-01	-2.28	7.56E-03	-1.37	2.22E-01	-2.07	1.74E-02
Phvul.001G026400	no data	-1.52	2.79E-02	-2.07	3.47E-03	-1.32	8.29E-02	-1.50	3.62E-02
Phvul.009G086700	PTHR33021:SF8 - EARLY NODULIN-LIKE PROTEIN 1-RELATED (1 of 3)	-1.52	6.18E-02	-2.67	4.33E-03	-0.74	4.74E-01	-2.23	1.53E-02
Phvul.007G038400	PTHR10177:SF251 - CYCLIN-D3-1 (1 of 1)	-1.53	2.00E-01	-3.02	1.63E-03	-1.13	4.16E-01	-2.73	5.34E-03
Phvul.011G119900	PTHR10641//PTHR10641:SF574 - MYB-LIKE DNA-BINDING PROTEIN MYB // SUBFAMILY NOT NAMED (1 of 1)	-1.53	3.38E-02	-1.57	1.66E-02	-0.39	6.91E-01	-0.55	4.11E-01
Phvul.007G189200	PTHR23107:SF6 - GRF1-INTERACTING FACTOR 1 (1 of 2)	-1.54	2.63E-02	-0.85	2.00E-01	-0.79	3.52E-01	-0.83	2.10E-01
Phvul.005G113300	PTHR31717:SF13 - CCT MOTIF FAMILY PROTEIN-RELATED (1 of 1)	-1.55	7.32E-02	-2.10	8.30E-03	-1.22	2.46E-01	-1.45	7.30E-02
Phvul.005G064400	PTHR31044:SF19 - CARBOHYDRATE-BINDING X8 DOMAIN-CONTAINING PROTEIN-RELATED (1 of 2)	-1.55	8.73E-04	-1.35	7.65E-02	-1.54	1.54E-03	-1.20	1.13E-01
Phvul.006G057200	no data	-1.56	4.68E-02	-1.30	1.78E-01	-1.69	4.50E-02	-0.78	4.21E-01
Phvul.010G064300	K02728 - 20S proteasome subunit alpha 3 (PSMA4) (1 of 2)	-1.57	4.87E-02	-2.23	2.24E-03	-1.70	3.67E-02	-2.21	2.72E-03
Phvul.011G125500	PTHR31280:SF1 - F19P19.6 PROTEIN (1 of 3)	-1.57	1.39E-02	-1.19	6.53E-02	-1.38	4.68E-02	-1.15	7.79E-02
Phvul.009G158000	PTHR33147:SF3 - DEFENSIN-LIKE PROTEIN 6 (1 of 4)	-1.58	2.29E-01	-3.96	1.62E-04	-0.61	7.41E-01	-1.48	1.54E-01
Phvul.010G025800	PTHR11017//PTHR11017:SF163 - LEUCINE-RICH REPEAT-CONTAINING PROTEIN // SUBFAMILY NOT NAMED (1 of 62)	-1.58	2.43E-02	-1.98	1.25E-03	-0.99	2.70E-01	-2.21	4.27E-04
Phvul.001G119700	PTHR33646:SF2 - F20H23.8 PROTEIN-RELATED (1 of 2)	-1.59	2.99E-02	-0.28	7.46E-01	-1.52	4.74E-02	-1.59	2.92E-02
Phvul.001G221700	PTHR21659:SF19 - HYDROPHOBIC PROTEIN RCI2B (1 of 2)	-1.59	2.14E-02	-2.16	5.67E-04	-1.43	6.26E-02	-1.60	1.46E-02
Phvul.003G131000	PTHR39113:SF1 - MEMBRANE LIPOPROTEIN-RELATED (1 of 1)	-1.59	1.08E-02	-1.43	6.69E-02	-0.82	2.76E-01	-0.53	5.06E-01
Phvul.002G296700	PTHR22950:SF229 - GABA TRANSPORTER 2-RELATED (1 of 1)	-1.59	4.33E-02	-0.51	5.98E-01	-1.25	1.73E-01	-0.50	5.97E-01
Phvul.002G009900	PTHR31311:SF3 - GLYCOSYLTRANSFERASE 6-RELATED (1 of 1)	-1.59	9.38E-03	-1.64	3.65E-03	-0.82	3.19E-01	-1.31	2.58E-02
Phvul.007G179900	PTHR24115:SF449 - MICROTUBULE BASED KINESIN MOTOR PROTEIN (1 of 1)	-1.60	4.83E-02	-0.92	4.18E-01	-1.41	1.12E-01	-0.87	4.33E-01
Phvul.010G087500	PTHR11945//PTHR11945:SF222 - MADS BOX PROTEIN // SUBFAMILY NOT NAMED (1 of 2)	-1.60	1.72E-02	-1.21	4.94E-02	-1.00	2.45E-01	-0.64	3.45E-01
Phvul.001G265600	1.1.1.3//2.7.2.4 - Aspartate kinase / Aspartokinase (1 of 2)	-1.61	9.00E-02	-2.32	1.24E-02	-1.42	1.89E-01	-1.56	9.60E-02
Phvul.003G077600	K08744 - cardiolipin synthase (CRLS) (1 of 1)	-1.63	7.08E-02	-1.73	3.84E-02	-0.89	4.23E-01	-1.55	6.74E-02
Phvul.006G041900	K15077 - elongin-A (ELA1) (1 of 4)	-1.63	3.38E-02	-0.69	4.14E-01	-1.33	1.19E-01	-0.08	9.38E-01
Phvul.001G229800	PTHR32080:SF5 - CYSTEINE-RICH REPEAT SECRETORY PROTEIN 60 (1 of 2)	-1.63	2.52E-02	-1.01	1.51E-01	-1.11	2.21E-01	-1.05	1.38E-01
Phvul.005G039200	PTHR10353//PTHR10353:SF44 - GLYCOSYL HYDROLASE // SUBFAMILY NOT NAMED (1 of 2)	-1.66	1.69E-02	-2.23	5.30E-04	-1.30	1.10E-01	-2.51	1.20E-04
Phvul.006G107400	PF14365 - Domain of unknown function (DUF4409) (DUF4409) (1 of 17)	-1.66	1.49E-01	-2.28	1.76E-02	-1.49	2.47E-01	-2.36	1.83E-02
Phvul.002G071800	PF06075 - Plant protein of unknown function (DUF936) (DUF936) (1 of 7)	-1.69	4.32E-02	-1.17	1.90E-01	-1.57	8.32E-02	-0.48	6.17E-01
Phvul.009G110700	PF07911 - Protein of unknown function (DUF1677) (DUF1677) (1 of 10)	-1.70	1.36E-02	-0.42	5.06E-01	-0.95	2.41E-01	-1.37	1.67E-02
Phvul.011G021300	no data	-1.70	5.60E-03	-1.22	1.85E-01	-1.43	3.08E-02	-0.05	9.61E-01
Phvul.011G117500	3.5.2.3 - Dihydroorotase / DHOase (1 of 2)	-1.71	2.77E-03	-1.96	2.87E-03	-1.35	3.57E-02	-1.67	1.20E-02
Phvul.002G004300	3.1.3.58 - Sugar-terminal-phosphatase / Xylitol-5-phosphatase (1 of 3)	-1.71	3.01E-01	-3.11	8.53E-03	-1.40	4.63E-01	-2.94	1.67E-02
Phvul.001G223900	KOG3195 - Uncharacterized membrane protein NPD008/CGI-148 (1 of 2)	-1.73	1.53E-02	-2.65	6.91E-04	-0.64	4.98E-01	-1.99	8.28E-03
Phvul.009G257600	1.1.3.37 - D-arabinono-1,4-lactone oxidase / D-arabinono-gamma-lactone oxidase (1 of 4)	-1.73	4.26E-02	-1.03	2.33E-01	-1.51	1.10E-01	-0.65	4.74E-01
Phvul.009G093400	PTHR18896:SF11 - PHOSPHOLIPASE D ALPHA 1-RELATED (1 of 5)	-1.76	2.00E-03	-0.55	4.30E-01	-0.73	3.25E-01	-0.69	3.01E-01
Phvul.009G103900	PF04654 - Protein of unknown function, DUF599 (DUF599) (1 of 10)	-1.78	1.04E-01	-1.69	7.58E-02	-1.95	9.20E-02	-1.91	4.93E-02
Phvul.002G240800	PTHR13194:SF19 - NAD(P)-BINDING ROSSMANN-FOLD SUPERFAMILY PROTEIN (1 of 1)	-1.79	1.59E-01	-3.60	4.91E-04	-0.81	6.51E-01	-3.56	9.75E-04
Phvul.008G213400	PTHR13018:SF46 - ERD (EARLY-RESPONSIVE TO DEHYDRATION STRESS) FAMILY PROTEIN-RELATED (1 of 1)	-1.80	6.80E-02	-2.56	2.33E-03	-1.66	1.27E-01	-1.85	3.32E-02
Phvul.007G061300	PTHR33109:SF4 - EPIDERMAL PATTERNING FACTOR-LIKE PROTEIN 5 (1 of 2)	-1.80	1.38E-02	-0.82	2.83E-01	-0.43	6.61E-01	-0.54	4.92E-01
Phvul.005G005000	PTHR32472:SF12 - P-LOOP CONTAINING NUCLEOSIDE TRIPHOSPHATE HYDROLASES SUPERFAMILY PROTEIN (1 of 1)	-1.81	3.97E-02	-1.50	1.40E-01	-2.54	4.87E-03	-3.78	2.70E-03
Phvul.006G189500	PF01344 - Kelch motif (Kelch 1) (1 of 34)	-1.81	1.57E-02	-2.40	5.44E-03	-2.71	5.41E-04	-2.44	6.76E-03
Phvul.007G090100	2.1.1.154 - Isoliquiritigenin 2'-O-methyltransferase / CHMT (1 of 8)	-1.83	1.40E-01	-2.84	9.89E-03	-2.49	5.78E-02	-2.45	2.41E-02
Phvul.L002544	no data	-1.85	3.82E-03	-2.09	7.04E-03	-1.51	2.97E-02	-1.17	1.12E-01
Phvul.008G122400	no data	-1.89	3.60E-02	-1.09	1.65E-01	-0.96	3.90E-01	-1.44	6.34E-02
Phvul.006G140300	PTHR31246:SF5 - MICROTUBULE-ASSOCIATED PROTEIN 70-5 (1 of 2)	-1.89	2.63E-02	-2.70	6.10E-04	-1.30	2.11E-01	-3.16	1.28E-04
Phvul.011G097900	PTHR31415:SF2 - LATE EMBRYOGENESIS ABUNDANT HYDROXYPROLINE-RICH GLYCOPROTEIN-RELATED (1 of 4)	-1.89	5.52E-04	-1.33	2.65E-02	-1.52	1.12E-02	-0.51	4.31E-01
Phvul.003G212200	PTHR31636:SF16 - SCARECROW-LIKE PROTEIN 28 (1 of 1)	-1.90	2.45E-02	-1.55	6.94E-02	-1.38	1.64E-01	-0.82	3.53E-01
Phvul.001G169100	PTHR30509:SF9 - F3L24.34 PROTEIN (1 of 1)	-1.90	2.87E-02	-1.79	1.33E-01	-1.51	1.30E-01	-0.84	4.76E-01
Phvul.005G018500	PTHR24078:SF177 - PROTEIN DNJ-23-RELATED (1 of 12)	-1.91	8.39E-02	-2.32	9.62E-03	-1.47	2.51E-01	-2.90	1.65E-03
Phvul.008G158600	3.2.1.78 - Mannan endo-1,4-beta-mannosidase / Endo-1,4-mannanase (1 of 12)	-1.96	2.05E-02	-2.37	1.70E-02	-1.43	1.47E-01	-3.09	4.92E-03
Phvul.001G000500	K07760 - cyclin-dependent kinase [EC:2.7.11.22] (CDK) (1 of 2)	-1.97	2.25E-02	-2.08	1.86E-02	-1.76	5.59E-02	-1.58	7.03E-02
Phvul.005G121300	no data	-2.00	1.13E-01	-4.33	1.46E-03	-1.88	1.64E-01	-2.33	4.20E-02
Phvul.010G069638	PTHR15672//PTHR15672:SF16 - CAMP-REGULATED PHOSPHOPROTEIN 21 RELATED R3H DOMAIN CONTAINING PROTEIN	-2.03	4.77E-02	-1.74	1.02E-01	-0.73	5.96E-01	-1.55	1.51E-01
Phvul.004G077900	PTHR19957:SF91 - SYNTAXIN-RELATED PROTEIN KNOLLE (1 of 1)	-2.03	3.46E-03	-0.83	3.61E-01	-1.16	1.63E-01	-1.68	6.12E-02
Phvul.002G115100	PTHR10177:SF250 - CYCLIN-B2-3-RELATED (1 of 2)	-2.06	1.16E-02	-1.15	2.50E-01	-1.46	1.22E-01	-1.05	2.88E-01
Phvul.008G274700	PTHR24115:SF529 - KINESIN MOTOR FAMILY PROTEIN (1 of 2)	-2.07	1.94E-02	-1.12	2.44E-01	-1.68	9.47E-02	-1.04	2.85E-01
Phvul.008G051600	PTHR34124:SF2 - F16B3.27 PROTEIN (1 of 1)	-2.09	1.86E-04	-1.09	1.34E-01	-1.07	1.35E-01	-0.80	2.81E-01
Phvul.003G262100	no data	-2.11	2.88E-05	-0.19	8.37E-01	-1.06	6.88E-02	-0.68	4.05E-01
Phvul.011G023800	PF02140//PF02449 - Galactose binding lectin domain (Gal Lectin) // Beta-galactosidase (Glyco hydro 42) (1 of 2)	-2.26	4.24E-03	-1.11	2.14E-01	-1.50	1.02E-01	-1.26	1.52E-01
Phvul.010G158500	PTHR23042//PTHR23042:SF57 - CIRCADIAN PROTEIN CLOCK/ARNT/BMAL/PAS // SUBFAMILY NOT NAMED (1 of 13)	-2.28	3.50E-03	-1.82	1.43E-02	-1.44	1.32E-01	-1.09	1.61E-01
Phvul.008G196700	PTHR22807:SF34 - METHYLTRANSFERASE NSUN6-RELATED (1 of 1)	-2.49	4.68E-02	-4.19	7.95E-05	-2.17	1.11E-01	-3.20	2.73E-03
Phvul.007G033200	K18753 - butyrate response factor 1 (ZFP36L) (1 of 1)	-2.55	2.20E-04	-2.21	1.39E-03	-2.21	1.22E-03	-2.05	3.09E-03
Phvul.L001946	PF05755 - Rubber elongation factor protein (REF) (REF) (1 of 4)	-2.58	7.82E-05	-0.84	2.30E-01	-1.34	6.34E-02	-1.51	3.26E-02
Phvul.001G232800	PTHR19918:SF7 - PROTEIN FIZZY-RELATED 3 (1 of 1)	-2.73	9.47E-07	-1.19	5.16E-02	-1.20	5.59E-02	-1.08	8.68E-02
Phvul.011G209700	PTHR13683:SF298 - ASPARTIC PROTEINASE CDR1-RELATED (1 of 13)	-2.77	1.98E-02	-5.39	1.23E-03	-2.69	2.97E-02	-4.15	5.78E-03
Phvul.001G141700	PTHR31314:SF7 - MYB-LIKE HTH TRANSCRIPTIONAL REGULATOR FAMILY PROTEIN (1 of 4)	-2.94	1.23E-02	-1.04	3.80E-01	-2.44	6.03E-02	-1.10	3.19E-01
Phvul.003G222400	PTHR10641//PTHR10641:SF558 - MYB-LIKE DNA-BINDING PROTEIN MYB // SUBFAMILY NOT NAMED (1 of 2)	-3.08	9.02E-05	-2.08	6.32E-03	-1.42	7.33E-02	-1.40	5.85E-02
Phvul.001G168900	no data	-3.14	7.00E-03	-1.62	1.93E-01	-2.08	1.19E-01	-2.37	5.79E-02
Phvul.011G208200	PTHR24078:SF219 - DNAJ HEAT SHOCK N-TERMINAL DOMAIN-CONTAINING PROTEIN (1 of 3)	-3.21	3.26E-02	-3.80	1.19E-02	-2.40	1.34E-01	-3.82	1.49E-02
Phvul.006G033900	PTHR33210:SF6 - PROTODERMAL FACTOR 1 (1 of 2)	-3.49	1.35E-02	-4.66	2.07E-03	-2.65	1.10E-01	-4.10	6.82E-03

Pvalue

0.00 0.00 0.05 0.00

LogRatio

-2.50 -1.50 -1.00 0.00 1.00 1.50
